# PLOD1 Catalytic Activity Stabilizes ENO1 by Limiting FBXW7‐dependent Degradation to Promote Glycolysis and TMZ Resistance in Glioblastoma

**DOI:** 10.1002/advs.78070

**Published:** 2026-09-27

**Authors:** Fen Xue, Dingkong Liang, Xin Chen, Fei Fan, Xiayun He

**Affiliations:** ^1^ Department of Radiation Oncology Fudan University Shanghai Cancer Center Shanghai China; ^2^ Department of Oncology Shanghai Medical College Fudan University Shanghai China; ^3^ Shanghai Clinical Research Center for Radiation Oncology Shanghai China; ^4^ Shanghai Key Laboratory of Radiation Oncology Shanghai China; ^5^ Department of General Surgery Longhua Hospital Shanghai University of Traditional Chinese Medicine Shanghai China; ^6^ Department of Neurosurgery Fudan University Shanghai Cancer Center Shanghai China; ^7^ Department of Neurosurgery Union Hospital Tongji Medical College Huazhong University of Science and Technology Wuhan China

**Keywords:** ENO1, glioblastoma, metabolic reprogramming, PLOD1, temozolomide resistance

## Abstract

Acquired resistance to temozolomide (TMZ) remains a major therapeutic challenge in glioblastoma (GBM), with metabolic reprogramming emerging as a critical driver of treatment failure. Procollagen lysyl hydroxylase 1 (PLOD1) is identified as a key regulator of adaptive metabolic remodeling in TMZ‐resistant GBM. Elevated PLOD1 promotes a hyper‐glycolytic phenotype by maintaining the stability of the glycolytic enzyme alpha‐enolase (ENO1). Mechanistically, PLOD1 catalytic activity increases an ENO1‐associated hydroxylation signal, promotes ENO1 stability, and limits FBXW7‐dependent ubiquitination and proteasomal degradation. Structural and functional analyses reveal that the central Ndst region of PLOD1 mediates substrate interaction, whereas its catalytic domain is required for ENO1 stabilization and downstream metabolic regulation. Clinically, PLOD1 and ENO1 expression levels are positively correlated in GBM specimens, and their co‐expression is associated with unfavorable patient outcomes. In xenograft models, inhibition of the PLOD1‐ENO1 axis suppresses tumor progression and enhances TMZ responsiveness. These findings establish the PLOD1‐FBXW7‐ENO1 regulatory axis as a critical mediator of GBM metabolic adaptation and therapeutic resistance, highlighting its potential as a prognostic biomarker and therapeutic target.

## Graphical abstract

PLOD1 interacts with ENO1 and limits its FBXW7‐dependent ubiquitination and proteasomal degradation through a mechanism requiring PLOD1 catalytic activity. ENO1 stabilization sustains glycolysis, promoting glioblastoma cell proliferation, migration, and temozolomide resistance. Targeting the PLOD1‐ENO1 axis suppresses tumor progression and enhances temozolomide responsiveness in preclinical models, highlighting a potential therapeutic strategy for resistant glioblastoma.

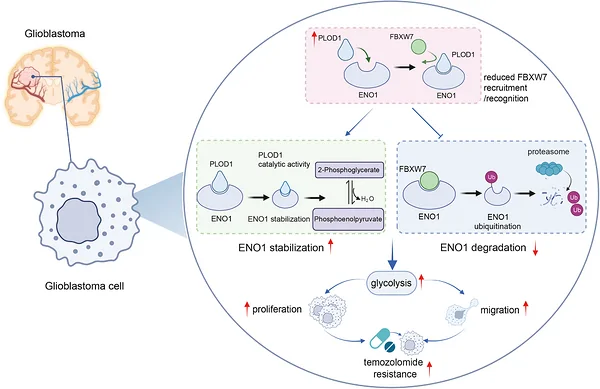

## 1 Introduction

Glioblastoma (GBM) represents a formidable clinical challenge, characterized by inevitable recurrence and a devastating median survival of less than 15 months [1, 2, 3]. Although the Stupp protocol remains the standard of care, the rapid emergence of resistance to temozolomide (TMZ) constitutes the principal driver of treatment failure [4, 5, 6]. While classical biomarkers such as O^6^‐methylguanine‐DNA methyltransferase (MGMT) promoter methylation offer some prognostic value, they fail to fully capture the dynamic adaptive mechanisms that enable tumor cells to survive therapeutic stress [7, 8, 9, 10, 11, 12]. This gap underscores the urgent need to decipher the molecular machinery that drives adaptive resistance [13, 14].

Metabolic reprogramming has emerged as a hallmark of TMZ resistance in GBM [3, 15, 16, 17]. To withstand drug‐induced DNA damage and oxidative stress, tumor cells shift their metabolism to supply the ATP, biosynthetic precursors, and redox buffering capacity required for survival [18, 19, 20, 21, 22]. High‐grade gliomas are prototypical Warburg tumors, exhibiting a profound dependency on aerobic glycolysis [23, 24, 25, 26]. Alpha‐enolase (ENO1), a core glycolytic enzyme that converts 2‐phosphoglycerate to phosphoenolpyruvate, is consistently upregulated in GBM and correlates with poor clinical outcomes [27, 28, 29]. However, despite its pivotal role in sustaining glycolytic flux, the mechanisms that maintain ENO1 protein stability under chemotherapeutic pressure remain poorly understood [30, 31, 32, 33, 34].

Accumulating evidence suggests that post‐translational modifications (PTMs) play a crucial role in rapidly fine‐tuning metabolic enzymes in response to cellular stress [35, 36, 37, 38, 39]. Phosphorylation, acetylation, and ubiquitination have all been shown to regulate the activity, stability, and stress adaptability of metabolic enzymes, thereby facilitating metabolic rewiring and therapy resistance in multiple cancers [40, 41]. However, whether lysine hydroxylation serves as an additional regulatory layer controlling metabolic enzyme stability remains largely unexplored.

In this context, the procollagen lysine hydroxylase 1 (PLOD1) has attracted attention for its non‐canonical intracellular roles that extend beyond extracellular matrix remodeling [42, 43, 44]. Conversely, the E3 ubiquitin ligase FBXW7 serves as a major tumor‐suppressive regulator that targets numerous oncoproteins and metabolic enzymes for proteasomal degradation [45, 46, 47]. The paradoxical persistence of high ENO1 expression in TMZ‐resistant GBM, despite the presence of FBXW7, suggests that an upstream PTM‐dependent mechanism might protect ENO1 from ubiquitin‐mediated turnover [30, 48, 49, 50].

Here, we identify PLOD1 as an important mediator of metabolic reprogramming and chemoresistance in GBM. We demonstrate that PLOD1 physically interacts with ENO1 and that its catalytic activity is associated with ENO1 modification and stabilization, thereby reducing FBXW7 recognition and proteasomal degradation. This PLOD1‐ENO1 signaling axis enforces a hyper‐glycolytic state essential for tumor survival and therapeutic tolerance, revealing a previously unrecognized metabolic vulnerability and a promising target to overcome TMZ resistance.

## 2 Materials and Methods

### 2.1 Cell Culture and Reagents

Human GBM cell lines U87 MG (ATCC, #HTB‐14; RRID: CVCL_0022) and U251 MG (Sigma‐Aldrich, #09063001; RRID: CVCL_0021), and the HEK293T cell line (ATCC, #CRL‐3216; RRID: CVCL_0063) were obtained from the American Type Culture Collection (ATCC) or Sigma‐Aldrich. The cell lines were authenticated by short tandem repeat (STR) profiling and confirmed to be free of mycoplasma contamination. Detailed information regarding cell‐line provenance, acquisition dates, passage numbers, STR authentication, and mycoplasma testing is provided in the Supporting Information (Cell Line Authentication and Quality Control). Cells were authenticated by short tandem repeat (STR) profiling and routinely tested for mycoplasma contamination. Cells were cultured in Dulbecco's Modified Eagle Medium (DMEM, Gibco, #11995065) supplemented with 10% Fetal Bovine Serum (FBS, Gibco, #10270106) and 1% Penicillin‐Streptomycin (Gibco, #15140122) at 37°C in a humidified atmosphere with 5% CO_2_.

Temozolomide (TMZ, #S1237) and 2‐Deoxy‐D‐glucose (2‐DG, S4701) were purchased from Selleck Chemicals; The activator of glucokinase GKA50 (SML0849) and Cycloheximide (CHX, C7698) were from Sigma–Aldrich.

### 2.2 Establishment of Tmz‐Resistant Cell Lines

TMZ‐resistant sublines (U87‐R and U251‐R) were established by exposing parental cells to gradually increasing concentrations of TMZ. Briefly, cells were initially treated with low‐dose TMZ, and the concentration was gradually increased after cells recovered and resumed stable growth. The TMZ concentration was sequentially increased from 0.125 µg/mL to 16 µg/mL over approximately 9 months. Cells capable of maintaining stable growth under 16 µg/mL TMZ were considered TMZ‐resistant and designated as U251‐R, U87‐R. Parental cells were cultured in parallel without TMZ exposure.

### 2.3 Tandem Mass Tags (TMT)‐Based Quantitative Proteomic Analysis

To identify global protein expression changes associated with TMZ resistance, parental U251 cells and TMZ‐resistant U251‐R cells were harvested and lysed in 8 M urea buffer supplemented with protease inhibitors. Protein concentrations were determined using the BCA Protein Assay Kit (Thermo Fisher, #23225). A total of 100 µg protein per sample was reduced with 5 mM dithiothreitol (DTT) at 56°C for 30 min and alkylated with 11 mM iodoacetamide (IAA) at room temperature in the dark for 15 min. Samples were diluted and digested with sequencing‐grade trypsin (Promega) at a 1:50 (enzyme: protein) ratio overnight at 37°C. Resulting peptides were desalted and labeled using the TMTpro 16‐plex Isobaric Labeling Reagent Set (Thermo Fisher, #A44522) according to the manufacturer's instructions. Labeled peptides were pooled and fractionated by high‐pH reversed‐phase liquid chromatography, followed by LC‐MS/MS analysis on an Orbitrap Fusion Lumos mass spectrometer (Thermo Fisher Scientific) in data‐dependent acquisition mode. Raw spectra were processed using Proteome Discoverer software (v2.5) with the SEQUEST HT search engine against the UniProt human database [51]. Proteins quantified with at least two unique peptides were retained for further analysis. Differentially expressed proteins (DEPs) were strictly defined using a bidirectional threshold: fold change (FC) > 1.3 (up‐regulated) or FC < 1/1.3 (down‐regulated) (equivalent to |log_2_FC| > 0.379), accompanied by a Student's t‐test p < 0.05. KEGG pathway enrichment analysis was performed using Metascape (http://metascape.org) and the ClusterProfiler package in R.

### 2.4 ENO1 Immunoprecipitation and LC‐MS/MS Analysis

To identify potential ENO1 lysine modification sites regulated by PLOD1, ENO1 was immunoprecipitated from U251R cells transfected with either vector control or PLOD1 overexpression plasmids. Cell lysates were incubated with anti‐ENO1 antibody and Protein A/G magnetic beads overnight at 4°C. Immunoprecipitated proteins were separated by SDS‐PAGE, and the band corresponding to ENO1 (∼47 kDa) was excised and subjected to in‐gel tryptic digestion. Peptide samples were analyzed using a Q Exactive HF‐X mass spectrometer (Thermo Fisher Scientific) coupled to nanoLC. Raw data were processed using Proteome Discoverer 2.4 against the UniProt human protein database. Carbamidomethylation of cysteine was set as a fixed modification, whereas oxidation (M/K) and N‐terminal acetylation were set as variable modifications. Because oxidation of lysine (+15.9949 Da) is indistinguishable in mass from hydroxylysine, oxidation (K) was used as a surrogate search parameter for hydroxylysine‐containing peptides. Candidate ENO1 modification sites were subsequently prioritized by integrating proteomic coverage, motif analysis, and structural accessibility.

### 2.5 Candidate Lysine Site Prioritization and Structural Analysis

Candidate lysine residues were prioritized by integrating multiple independent criteria, including sequence motif characteristics, ENO1‐IP LC‐MS/MS peptide coverage, evolutionary conservation, and structural accessibility. Lysine residues located within reported PLOD‐compatible sequence motifs, including canonical (X‐K‐G) or extended (X‐K‐A/S) patterns, were considered for initial screening. Candidate sites were mapped onto both the experimentally determined human ENO1 crystal structure (PDB ID: 3B97) and the full‐length AlphaFold‐predicted ENO1 structure retrieved from the AlphaFold Protein Structure Database (UniProt ID: P06733) using PyMOL software (Schrödinger, LLC). Solvent accessibility was evaluated based on SASA proxies of the lysine side‐chain nitrogen (NZ) from both structural models. Structural information from both models was integrated with proteomic evidence, motif characteristics, and evolutionary conservation to prioritize candidate residues. Based on the combined evidence, K28, K60, and K126 were selected for subsequent experimental validation.

### 2.6 Plasmids, shRNAs, and Lentiviral Transduction

#### 2.6.1 Mammalian Expression Plasmids

The full‐length human coding sequences for PLOD1 (N‐terminal Flag‐tagged), ENO1 (N‐terminal HA‐tagged), and FBXW7 (N‐terminal Myc‐tagged) were cloned into the pLVX‐puro lentiviral expression vector. A plasmid expressing His‐tagged Ubiquitin (Addgene, #31815) was used for in vitro ubiquitination assays.

#### 2.6.2 Mutagenesis

Site‐directed mutagenesis was performed using the Q5 Site‐Directed Mutagenesis Kit (NEB, E0554S) to generate the following constructs, all based on the pLVX‐Flag‐PLOD1 or pLVX‐HA‐ENO1 backbones:

PLOD1‐CD:A catalytically dead PLOD1 mutant

PLOD1‐MUT1:A PLOD1 mutant with a mutation in aa304‐638 to abrogate ENO1 binding.

PLOD1‐MUT2:A PLOD1 mutant with mutations in aa 639–727 to abrogate enzymatic activity.

ENO1‐MUT:The ENO1‐MUT construct was generated by introducing S263A/S268A substitutions into the predicted FBXW7‐binding region (SPDDPS, residues 263–268) of wild‐type ENO1.

ENO1 K28R, K60R, and K126R: Lysine‐to‐arginine substitution mutants generated by site‐directed mutagenesis.

#### 2.6.3 Truncation Constructs

For mammalian expression: A series of Flag‐tagged PLOD1 truncation constructs (fragments 1–638, 304–638, 639–727, and 304–727) were cloned into the pLVX‐puro vector for functional analyses in glioblastoma cells.

For bacterial expression: The same PLOD1 fragments were cloned into the pGEX‐4T‐1 vector to generate glutathione S‐transferase (GST)‐tagged fusion proteins for GST pull‐down assays.

All constructs were verified by Sanger sequencing.

#### 2.6.4 ShRNAs

Short hairpin RNAs (shRNAs) targeting human PLOD1 and FBXW7 were cloned into the pLKO.1‐puro lentiviral vector. The target sequences were:

shPLOD1#1: 5'‐CCTGGCACTGATGGAGGAGAT‐3'

shPLOD1#2: 5'‐GCTTATCAGCAAGAAGCGCTA‐3'

shFBXW7#1: 5'‐CCTAAAGAGTTGGCACTCTAT‐3'

shFBXW7#2: 5'‐CCAGTCGTTAACAAGTGGAAT‐3'

A non‐targeting shRNA (shNC, Addgene, #1864) was used as a control.

#### 2.6.5 Lentiviral Production and Transduction

Lentiviral particles were produced in HEK293T cells by co‐transfecting the expression or shRNA vectors with packaging plasmids psPAX2 (Addgene, #12260) and pMD2.G (Addgene, #12259) using Lipofectamine 3000 (Invitrogen, #L3000015). Viral supernatants were harvested at 48 and 72 h post‐transfection, filtered (0.45 µm), and used to infect target cells in the presence of 8 µg/mL polybrene (Sigma‐Aldrich, #H9268). Stably transduced cells were selected and maintained in medium containing 2 µg/mL puromycin (Gibco, #A1113803). The efficiency of knockdown or overexpression was validated by qRT‐PCR and/or Western blotting, with representative results shown in Figure S1.

### 2.7 Western Blotting and Antibodies

Cells were lysed in radioimmunoprecipitation assay (RIPA) buffer (Thermo Fisher, #89900) supplemented with protease and phosphatase inhibitor cocktail (Sigma‐Aldrich, #PPC1010). Protein concentrations were determined using the BCA Protein Assay Kit (Thermo Fisher, #23225). Equal amounts of protein were separated by sodium dodecyl sulfate‐polyacrylamide gel electrophoresis (SDS‐PAGE), transferred to polyvinylidene difluoride (PVDF) membranes (Millipore, #IPVH00010), and blocked with 5% non‐fat milk in Tris‐buffered saline with Tween 20 (TBST). Membranes were incubated overnight at 4°C with primary antibodies, followed by incubation with HRP‐conjugated secondary antibodies. Detailed information on the antibodies used is provided in Table S1. Blots were visualized using SuperSignal West Pico PLUS Chemiluminescent Substrate (Thermo Fisher, #34580).

### 2.8 RNA Extraction and Quantitative Real‐Time PCR (qRT‐PCR)

Total RNA was extracted using the RNeasy Mini Kit (Qiagen, #74104). cDNA was synthesized using the iScript cDNA Synthesis Kit (Bio‐Rad, #1708891). qRT‐PCR was performed on a CFX96 Real‐Time PCR Detection System (Bio‐Rad) using iTaq Universal SYBR Green Supermix (Bio‐Rad, #1725121). Relative gene expression was calculated using the 2^−^ΔΔCt method with ACTB as the endogenous reference gene. The stability of ACTB expression across experimental groups was confirmed before normalization. Primer sequences are listed in Table S2.

### 2.9 Cycloheximide (CHX) Chase Assay

To assess protein stability, a cycloheximide (CHX) chase assay was performed [52]. Cells were treated with 100 µg/mL CHX (Sigma, #C7698) to block de novo protein synthesis. At the indicated time points (0, 2, 4, and 8 h) post‐treatment, cells were harvested and lysed. The remaining levels of the target proteins were then analyzed by Western blotting. Protein band intensities were quantified using ImageJ software, normalized to the corresponding ACTIN loading control, and plotted as line graphs to determine the protein half‐life.

### 2.10 Immunofluorescence (IF) Staining

U87‐R and U251‐R cells were grown on glass coverslips placed in 24‐well plates. For staining, cells were fixed with 4% paraformaldehyde (PFA) in PBS for 15 min and then permeabilized with 0.2% Triton X‐100 in PBS for 10 min at room temperature. After blocking with 5% bovine serum albumin (BSA) for 1 h, the cells were incubated overnight at 4°C with primary antibodies against PLOD1 and ENO1. After washing, cells were incubated for 1 h at room temperature in the dark with corresponding secondary antibodies. Nuclei were counterstained with 4′,6‐diamidino‐2‐phenylindole (DAPI; Beyotime, #C1002). The coverslips were then mounted on microscope slides using an anti‐fade mounting medium (Beyotime, #P0126). All primary and secondary antibodies used in this study are listed in Table S1.

Fluorescence images were acquired using a Leica TCS SP8 LIGHTNING super‐resolution confocal microscope (Leica Microsystems, Wetzlar, Germany) equipped with a 100× oil‐immersion objective lens. Fluorescence intensity was quantified using ImageJ software (NIH) [53, 54]. All imaging and analysis parameters, including exposure time, were maintained consistently for all samples.

To quantitatively evaluate the spatial association between PLOD1 and ENO1, co‐localization analysis was performed using the Coloc2 plugin in Fiji/ImageJ. Pearson's correlation coefficients (PCCs) were calculated from five randomly selected cells per group using identical thresholding parameters after background subtraction. PCC values were used as a quantitative measure of PLOD1–ENO1 co‐localization.

### 2.11 Subcellular Fractionation Assay

Subcellular fractionation was performed using a Cell Fractionation Kit (Thermo Fisher Scientific, Cat. No. 78840) according to the manufacturer's instructions. Briefly, U87, U87‐R, U251, and U251‐R cells were harvested and separated into nuclear (N), cytoplasmic (C), and membrane/endoplasmic reticulum‐associated (M/ER) fractions. Equal amounts of protein from each fraction were subjected to western blot analysis. Lamin B1, ACTIN, and Calnexin were used as markers for nuclear, cytoplasmic, and ER‐associated fractions, respectively.

### 2.12 In Vitro Ubiquitination Assay

For in vitro ubiquitination assays, U87‐R and U251‐R cells were seeded in 6‐well plates. Cells were then co‐transfected with various plasmid constructs, including those expressing His‐Ubiquitin, HA‐ENO1 (wild‐type or mutant), Myc‐FBXW7, and Flag‐PLOD1 (wild‐type or domain mutants), as specified in the figure legends. For knockdown experiments, cells were infected with lentiviruses carrying shRNA against PLOD1 (shPLOD1) or a non‐coding control (shNC) prior to plasmid transfection. At 48 h post‐transfection (or 72 h post‐infection), cells were treated with 20 µM MG132 for 6 h to inhibit proteasomal degradation. Cells were subsequently harvested and lysed on ice with IP lysis buffer (50 mM Tris‐HCl pH 7.4, 150 mM NaCl, 1% NP‐40) supplemented with a protease inhibitor cocktail and 10 mM N‐ethylmaleimide (NEM).

Cell lysates were subjected to immunoprecipitation overnight at 4°C using an anti‐ENO1 antibody (Abcam, ab227978). The immunocomplexes were captured with Protein A/G agarose beads, washed three times with lysis buffer, and eluted by boiling in 2x SDS loading buffer. The eluted proteins (IP samples) and whole‐cell lysates (Input samples) were analyzed by Western blotting. Ubiquitinated ENO1 was detected using an anti‐His tag antibody. The expression of relevant proteins (e.g., PLOD1, Myc‐FBXW7, Flag‐PLOD1, HA‐ENO1) and the loading control (ACTIN) in the input samples were confirmed using the corresponding primary antibodies. All antibodies used are detailed in Table S1.

### 2.13 Structural Modeling and Docking Analysis

To explore the structural basis underlying ENO1 regulation by PLOD1 and FBXW7, protein–protein docking analyses were performed using experimentally resolved and AlphaFold‐predicted structures. The crystal structure of human ENO1 (PDB ID: 3B97) was obtained from the Protein Data Bank, whereas the structures of human PLOD1 (UniProt: Q02809) and FBXW7 (UniProt: Q969H0) were retrieved from the AlphaFold Protein Structure Database.

Docking simulations were carried out using a blind docking strategy without predefined interaction constraints. Predicted complexes were ranked according to interface compatibility and interaction scores. Candidate interaction interfaces, residue contacts, and spatial relationships among ENO1, PLOD1, and FBXW7 were analyzed and visualized using PyMOL (version 3.1). The resulting models were used to identify putative PLOD1‐associated lysine residues and to evaluate the spatial relationship between the predicted PLOD1‐ and FBXW7‐binding surfaces on ENO1.

### 2.14 Immunoprecipitation (IP) and Mass Spectrometry (MS)

For co‐immunoprecipitation, cells were lysed in non‐denaturing IP Lysis Buffer (Thermo Fisher, #87787). Lysates were pre‐cleared and incubated with the indicated primary antibody or control IgG overnight at 4°C, followed by incubation with Protein A/G Magnetic Beads (Thermo Fisher, #88802) for 2 h. Beads were washed extensively, and bound proteins were eluted and analyzed by Western blotting. For IP‐MS, immunoprecipitated proteins were resolved by SDS‐PAGE, stained with Coomassie Blue, and the specific bands were excised and analyzed by LC‐MS/MS at the Fudan University Proteomics Core Facility.

### 2.15 GST Pull‐Down Assay

GST‐tagged PLOD1 fragments were expressed in E. coli BL21 (DE3) and purified using Glutathione Sepharose 4B beads (Cytiva, #17075601). HA‐ENO1 was expressed in HEK293T cells. Cell lysates containing HA‐ENO1 were incubated with GST or GST‐fusion proteins immobilized on beads. After extensive washing, bound proteins were eluted and analyzed by Western blotting.

### 2.16 Cell‐Based Functional Assays

Cell viability was assessed using the Cell Counting Kit‐8 (CCK‐8, Dojindo, CK04) according to the manufacturer's instructions. For TMZ sensitivity assays, parental and TMZ‐resistant cells were exposed to a range of TMZ concentrations under standard culture conditions, and cell viability was measured after treatment. The IC50 values were calculated using GraphPad Prism software.

Cell proliferation‐related changes were assessed using the Cell Counting Kit‐8 (CCK‐8, Dojindo, CK04) according to the manufacturer's instructions.

Cell migration was evaluated using 24‐well Transwell inserts (Corning, #3422; 8.0 µm pore size). Cells were seeded in the upper chamber in serum‐free medium, and the lower chamber was filled with medium containing 30% FBS. After 24 h, migrated cells on the lower surface were fixed, stained with crystal violet, and counted.

### 2.17 Metabolic Assays and Seahorse XF Analysis

Glucose consumption and lactate production in the culture medium were measured using the Glucose‐Glo Assay (Promega, #J6021) and Lactate‐Glo Assay (Promega, #J5021), respectively. Cellular ATP levels were quantified using the ATP Assay Kit (Beyotime, #S0026). All results were normalized to total protein concentration.

Real‐time metabolic flux was measured using a Seahorse XFe96 Analyzer (Agilent). The Glycolysis Stress Test was performed by sequential injection of glucose (10 mM), oligomycin (1 µM), and 2‐DG (50 mM). The Cell Mito Stress Test was performed by sequential injection of oligomycin (1 µM), carbonyl cyanide‐4‐(trifluoromethoxy) phenylhydrazone (FCCP, 1 µM), and rotenone/antimycin A (0.5 µM). Data was normalized to cell number.

### 2.18 Animal Studies

All animal experiments were performed in accordance with the protocols authorized by the Committee on the Ethics of Animal Experiments of Fudan University Shanghai Cancer Center (approval No. FUSCC‐IACUC‐S2025‐0236). Four‐to‐six‐week‐old male BALB/c nude mice (Charles River Laboratories) were used. For xenograft models, 5 × 10^6^ cells were suspended in PBS/Matrigel (Corning, #354234) and injected subcutaneously into the right flank of each mouse. When tumors reached approximately 50–100 mm^3^, mice were randomly assigned into different groups and treated with TMZ (50 mg/kg, i.p., every 3 days). Tumor volumes were measured every 3–4 days at the indicated time points, and tumor growth was calculated using the formula (length × width^2^)/2. Mouse body weights were monitored throughout the subcutaneous xenograft experiments, and the corresponding longitudinal body‐weight data are presented in Figure S10.

#### 2.18.1 Orthotopic GBM Xenograft Model

To evaluate tumor growth and TMZ resistance within a physiologically relevant brain microenvironment, an orthotopic glioblastoma xenograft model was established. Four‐to‐six‐week‐old male BALB/c nude mice were used for intracranial transplantation. Mice were anesthetized and placed in a stereotactic apparatus, and TMZ‐resistant U87R cells stably expressing shNC or shPLOD1 were suspended in PBS (3 × 10^5^ cells in 3 µL) and stereotactically injected into the right striatum. The injection coordinates relative to the bregma were set as follows: 1.0 mm posterior, 2.0 mm lateral, and 3.5 mm ventral. To minimize cell reflux, the needle was advanced to a depth of 4.0 mm and then withdrawn to 3.5 mm before slow injection of the cell suspension.

Following tumor establishment, mice were randomly assigned to different treatment groups and administered TMZ (10 mg/kg, intraperitoneally, once daily for 5 consecutive days) or vehicle as indicated. Tumor progression was monitored by bioluminescence imaging (BLI). At the endpoint, mice were euthanized, and brains were collected, fixed, and subjected to hematoxylin and eosin (H&E) staining and immunohistochemical (IHC) analysis.

#### 2.18.2 Measurement of Intratumoral Metabolites

Fresh tumor tissues were weighed and homogenized in ice‐cold PBS. Following centrifugation, the supernatants were collected for metabolite analysis. Intratumoral glucose, lactate, and ATP levels were quantified using a Glucose Assay Kit (Abcam, ab65333), Lactate Assay Kit (Abcam, ab65331), and ATP Assay Kit (Colorimetric/Fluorometric; Abcam, ab83355), respectively, according to the manufacturers' instructions. Metabolite levels were normalized to tumor wet weight and expressed as relative levels.

### 2.19 Human Tissue Specimens and Immunohistochemistry (IHC)

A cohort of glioma specimens and adjacent normal brain tissues was obtained from the Fudan University Shanghai Cancer Center Tissue Bank. The study was approved by the Medical Ethics Committee of Fudan University Shanghai Cancer Center (approval No. SZRYS2025257 and No. 1612167‐18), and written informed consent was obtained from all patients. For IHC, formalin‐fixed, paraffin‐embedded (FFPE) sections were deparaffinized, rehydrated, and subjected to antigen retrieval. Sections were then incubated overnight with primary antibodies against PLOD1, ENO1, and Ki67 (detailed information is provided in Table S1), followed by incubation with a biotinylated secondary antibody and visualization using the VECTASTAIN Elite ABC Kit and DAB Substrate (Vector Laboratories, #SK‐4100). IHC staining was quantified using QuPath software (v0.6.0). Tumor regions were manually annotated, followed by automated cell detection and classification according to staining intensity. Cells were categorized as negative, weak positive (1+), moderate positive (2+), or strong positive (3+). The H‐score was calculated as follows: H‐score = (% of 1+ cells ×1) + (% of 2+ cells × 2) + (% of 3+ cells × 3), with values ranging from 0 to 300. For survival and correlation analyses, patients were divided into high‐ and low‐expression groups based on the median H‐score value. Image analysis was performed using standardized parameters in QuPath [55], and investigators performing digital image quantification were blinded to clinical information and outcome data.

### 2.20 Statistical Analysis

No data transformation was applied unless otherwise specified. For quantitative analyses, data were normalized to the corresponding control or reference group where indicated in the individual figure legends, and the normalization procedures were performed according to the specific experimental design. No experimental data were excluded from statistical analysis. The number of biological replicates or samples (n) for each experiment is indicated in the corresponding figure legends, and all in vitro experiments were independently repeated at least three times unless otherwise stated.

Quantitative data are presented as mean ± standard deviation (SD). Statistical analyses were performed using GraphPad Prism 9 (GraphPad Software, San Diego, CA, USA). Comparisons between two groups were conducted using a two‐tailed Student's t‐test. Comparisons among three or more groups were performed using one‐way or two‐way analysis of variance (ANOVA), as appropriate, followed by Tukey's post hoc multiple‐comparisons test. Kaplan–Meier survival analyses were performed using the log‐rank (Mantel–Cox) test. Correlations between PLOD1 and ENO1 expression were evaluated using Pearson correlation analysis for the IHC cohort and Spearman correlation analysis for the TCGA‐GBMLGG and CGGA mRNAseq_325 datasets, as specified in the corresponding figure legends.

All statistical tests were two‐sided, and a two‐sided *p* value < 0.05 was considered statistically significant. Statistical significance is denoted as ^*^ *p* < 0.05, ^**^ *p* < 0.01, and ^***^ *p* < 0.001, while ns indicates no statistically significant difference.

## 3 Results

### 3.1 PLOD1 Is Upregulated in TMZ‐Resistant GBM and Contributes to Chemoresistance

**FIGURE 1:**
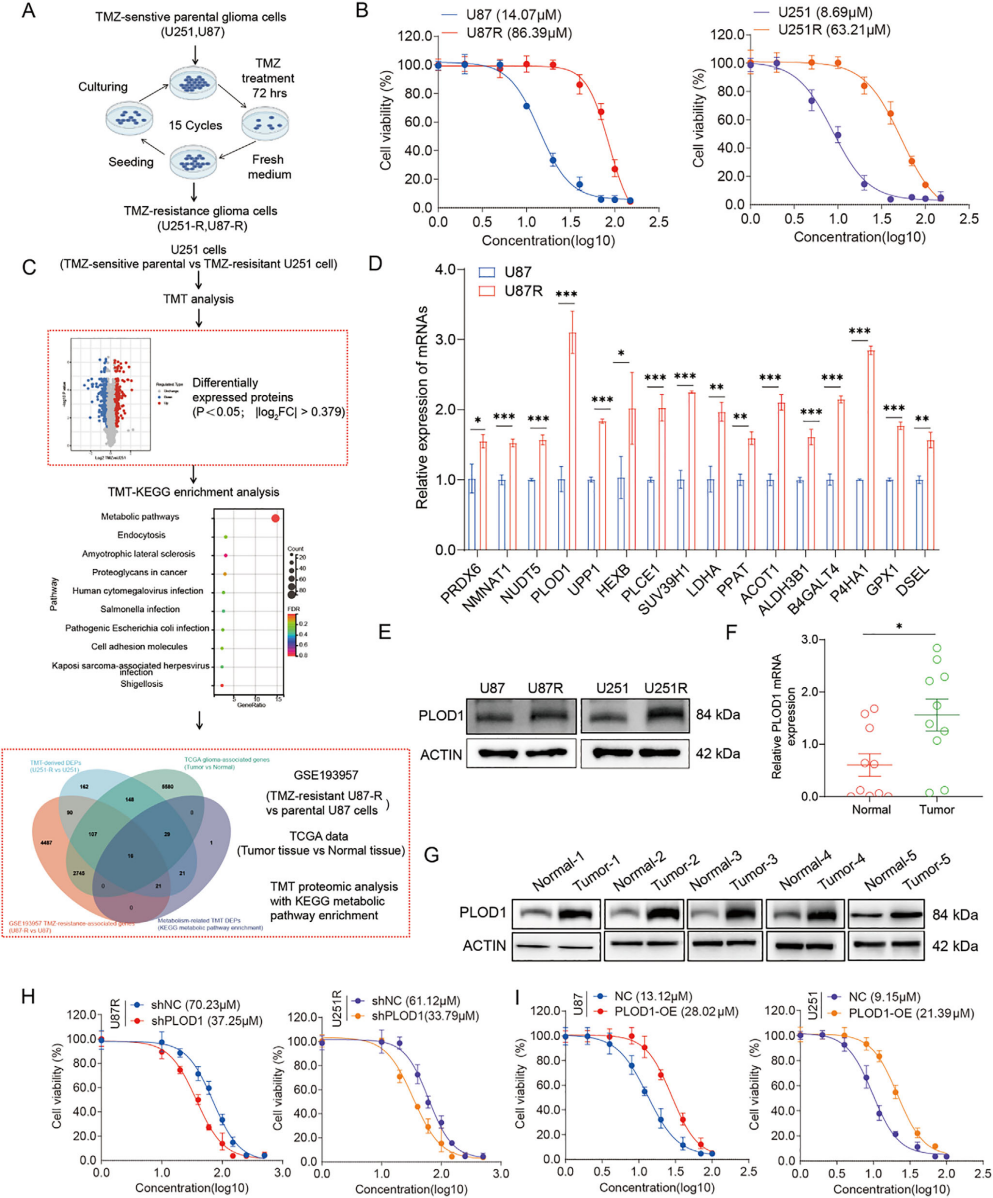
Upregulation of PLOD1 mediates acquired TMZ resistance in glioblastoma. (A) Schematic depicting the generation of TMZ‐resistant U87‐R and U251‐R cells through 15 cycles of dose‐escalating TMZ treatment. (B) Representative dose‐response curves of parental and TMZ‐resistant (U87‐R, U251‐R) cells treated with TMZ for 72 h. Cell viability was determined by CCK‐8 assay. IC_50_ values are indicated. (C) Schematic of the multi‐omic strategy to identify metabolic drivers of TMZ resistance by integrating TMT‐based proteomics, KEGG metabolic pathway enrichment, and public GSE193957 and TCGA datasets. (D) qRT‐PCR validation of the 16 metabolism‐related candidate genes identified by integrative analysis of TMT proteomics, GSE193957, and TCGA datasets. Compared between parental U87 and TMZ‐resistant U87‐R cells. (E) Western blot analysis of PLOD1 protein levels in parental and TMZ‐resistant cell lines. (F) Relative PLOD1 mRNA expression in human glioma tumor tissues versus adjacent normal brain tissues (n = 10). (G) Representative Western blot of PLOD1 protein levels in five pairs of patient‐matched tumors and normal tissues (*n* = 5 pairs). (H) Dose‐response curves of TMZ‐resistant cells following stable knockdown of PLOD1 (shPLOD1). (I) Dose‐response curves of parental cells following stable overexpression of PLOD1. For in vitro quantitative experiments, data are presented as mean ± SD from three independent experiments (n = 3), unless otherwise indicated. Statistical significance for two‐group comparisons was determined using a two‐tailed Student's t‐test. All statistical tests were two‐sided, with ^*^ *p* < 0.05, ^**^ *p* < 0.01, and ^***^ *p* < 0.001.

Acquired resistance to TMZ remains a major clinical obstacle in the treatment of GBM. To mechanistically dissect this clinical challenge, we first established TMZ‐resistant GBM cell lines. Parental U87 and U251 cells were subjected to 15 cycles of escalating TMZ treatment to generate their resistant counterparts, U87‐R and U251‐R (Figure 1A). The resultant resistant cell lines exhibited a marked reduction in TMZ sensitivity. The IC50 value for U87‐R cells increased 6.1‐fold to 86.39 µM compared to 14.07 µM in parental cells. Similarly, U251‐R cells demonstrated a 7.3‐fold increase in resistance, with an IC_50_ of 63.21 µM versus 8.69 µM in the parental line (Figure 1B).

To identify molecular alterations associated with the TMZ‐resistant phenotype, we performed quantitative TMT‐based proteomic analysis on parental U251 cells cultured with vehicle (DMSO) and established U251‐R cells maintained under TMZ selection conditions (Figure 1C). This analysis revealed numerous differentially expressed proteins (DEPs) (Figure 1C, volcano plot), with KEGG enrichment analysis identifying “Metabolic pathways” as the most significantly enriched category (Figure 1C, bubble plot). To prioritize robust and clinically relevant metabolic candidates, DEPs identified by TMT proteomics were first filtered according to KEGG metabolic pathway enrichment and subsequently integrated with two independent transcriptomic datasets (GSE193957 and TCGA). This four‐way integrative analysis identified 16 metabolism‐related candidate genes overlapping among the TMT proteomic and transcriptomic datasets (Figure 1C, Venn diagram). The expression of these 16 candidates was then experimentally evaluated by qRT‐PCR in TMZ‐resistant U87‐R cells. The full list of these 16 candidate proteins is now provided in Table S3.

Among the 16 candidates, PLOD1 showed consistent elevation across the integrated datasets and exhibited the largest increase among candidates experimentally validated by qRT‐PCR in TMZ‐resistant U87‐R cells (Figure 1D). We therefore selected PLOD1 for subsequent functional validation. Western blotting further confirmed increased PLOD1 protein abundance in both U87‐R and U251‐R cells compared with their parental counterparts (Figure 1E). To ascertain its clinical relevance, we examined PLOD1 expression in human glioma specimens. In agreement with these observations, both PLOD1 mRNA and protein levels were significantly elevated in glioma tissues relative to adjacent normal brain tissues (Figure 1F,G), underscoring its potential role in GBM pathology.

To functionally validate PLOD1 as a driver of TMZ resistance, we performed gain‐ and loss‐of‐function studies. Knockdown of PLOD1 using a specific shRNA in resistant U87‐R and U251‐R cells significantly re‐sensitized them to TMZ, as evidenced by a nearly two‐fold reduction in their respective IC_50_ values (Figure 1H). Conversely, ectopic overexpression of PLOD1 in parental, TMZ‐sensitive U87 and U251 cells was sufficient to confer TMZ resistance, more than doubling the IC_50_ values in both cell lines (Figure 1I). In addition to its central role in chemoresistance, PLOD1 also governed core malignant phenotypes; its overexpression enhanced cell proliferation and migration, whereas its depletion had the opposite effects (Figure S2). Taken together, these results indicate that PLOD1 is upregulated in TMZ‐resistant glioma cells and functionally contributes to acquired TMZ resistance and malignant phenotypes.

### 3.2 PLOD1 Promotes a Glycolytic Phenotype That Is Essential for Its Oncogenic Functions

**FIGURE 2:**
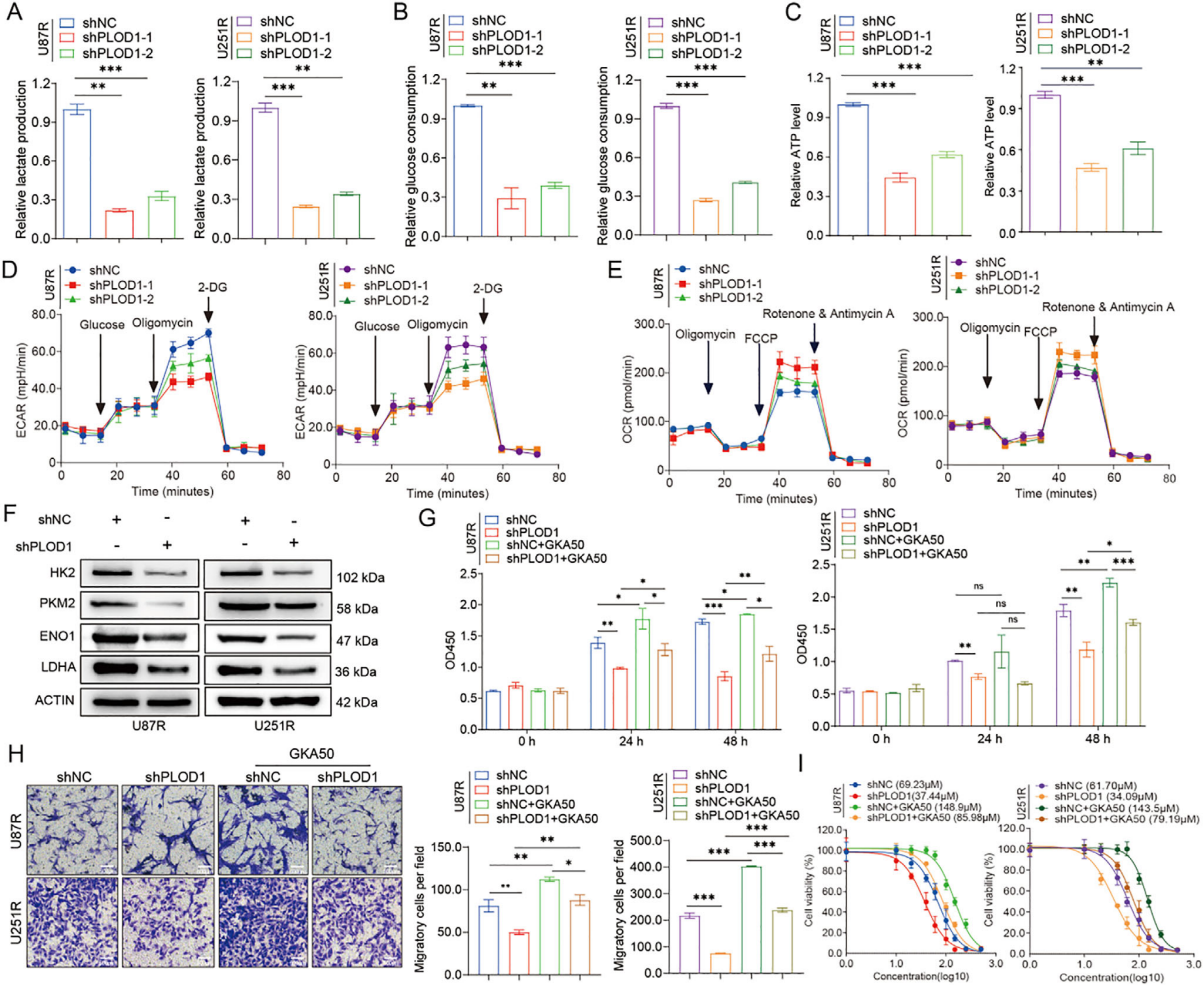
PLOD1 drives malignancy by enforcing a hyper‐glycolytic state. (A–C) Quantification of relative lactate production (A), glucose consumption (B), and intracellular ATP levels (C) in TMZ‐resistant U87R and U251R cells following stable knockdown of PLOD1. (D,E) Real‐time Seahorse metabolic flux analysis showing extracellular acidification rate (ECAR) (D) and oxygen consumption rate (OCR) (E) in TMZ‐resistant cells following PLOD1 knockdown. (F) Western blot analysis of HK2, PKM2, ENO1, and LDHA protein expression in resistant cells following PLOD1 knockdown, with ACTIN as the loading control. (G–I) Rescue experiments in PLOD1‐knockdown resistant cells treated with or without the glycolytic activator GKA50. (G) Cell proliferation determined by CCK‐8 assay. (H) Cell migration determined by Transwell assay. Scale bar = 500 µm (I) TMZ sensitivity determined by CCK‐8 assay, with IC50 values indicated. For quantitative in vitro experiments, data are presented as mean ± SD from three independent experiments (*n* = 3). Statistical significance for comparisons among three or more groups was determined using one‐way or two‐way ANOVA, followed by Tukey's multiple‐comparisons test. All statistical tests were two‐sided. ^*^ *p* < 0.05, ^**^ *p* < 0.01, and ^***^ *p* < 0.001; ns indicates no statistically significant difference.

To elucidate the mechanism by which PLOD1 drives chemoresistance, we first investigated its role in the metabolic reprogramming of GBM cells. We began by confirming that TMZ‐resistant (U87‐R, U251‐R) cells indeed exhibit a hyper‐glycolytic phenotype, characterized by increased glucose consumption and lactate production compared to their parental counterparts (Figure S3). We then tested whether PLOD1 directly regulates this metabolic state. Indeed, shRNA‐mediated depletion of PLOD1 in resistant cells suppressed their glycolytic activity, as evidenced by reduced lactate production, glucose consumption, and cellular ATP levels (Figure 2A–C). Real‐time metabolic flux analysis confirmed this, showing a decrease in the extracellular acidification rate (ECAR) and a concomitant increase in the oxygen consumption rate (OCR), indicating a shift away from glycolysis toward mitochondrial respiration (Figure 2D,E). Conversely, ectopic expression of PLOD1 in parental, TMZ‐sensitive cells were sufficient to induce a hyper‐glycolytic state, mirroring the phenotype of resistant cells (Figure S4A–E). This metabolic shift was driven by the expression regulation of key glycolytic enzymes representing distinct critical steps of the pathway, including HK2 (the initial rate‐limiting enzyme), PKM2 (the final rate‐limiting enzyme), ENO1 (responsible for phosphoenolpyruvate synthesis), and LDHA (essential for lactate production), all of which were downregulated upon PLOD1 depletion (Figure 2F). Together, these bidirectional genetic manipulations establish PLOD1 as a critical upstream regulator that enforces a glycolytic phenotype in GBM. In addition to its effects on glycolytic metabolism, we examined whether PLOD1 influences cellular redox status and mitochondrial integrity. DCFH‐DA staining revealed that PLOD1 overexpression markedly reduced intracellular ROS accumulation, whereas PLOD1 depletion produced the opposite effect in both U87R and U251R cells (Figure S7A). Consistently, JC‐1 staining demonstrated an increased proportion of JC‐1 aggregates and a concomitant reduction in JC‐1 monomers following PLOD1 overexpression, indicative of preserved mitochondrial membrane potential. In contrast, PLOD1 silencing promoted mitochondrial depolarization, as evidenced by reduced aggregate formation and increased monomer fluorescence (Figure S7B). Together, these findings suggest that PLOD1 not only promotes glycolytic reprogramming but also contributes to the maintenance of redox balance and mitochondrial homeostasis in GBM cells.

Next, we sought to determine whether this PLOD1‐driven glycolytic reprogramming was functionally essential for its oncogenic effects. To test this dependency, we pharmacologically manipulated glycolysis downstream of PLOD1. In PLOD1‐depleted cells, which exhibited reduced proliferation and migration, reactivation of glycolysis using the glucokinase activator GKA50 was sufficient to substantially rescue these malignant phenotypes (Figure 2G,H and Figure S5). Critically, restoring the glycolytic flux also reinstated chemoresistance, significantly increasing the TMZ IC50 in PLOD1‐knockdown cells (Figure 2I). In the reciprocal experiment, we sought to reverse PLOD1's effects by inhibiting glycolysis with 2‐Deoxy‐D‐glucose (2‐DG). We first confirmed that while 2‐DG treatment partially reduced PLOD1 mRNA levels, its overexpression was maintained compared to control cells (Figure S4F). Under these conditions, 2‐DG treatment abrogated the pro‐proliferative and pro‐migratory effects conferred by PLOD1 (Figures S4G,H and S6). Furthermore, blocking glycolysis re‐sensitized these cells to TMZ, effectively reversing PLOD1‐mediated chemoresistance (Figure S4I). These results provide compelling evidence that the oncogenic functions of PLOD1 are causally dependent on its ability to orchestrate a state of enhanced glycolysis.

### 3.3 PLOD1 Stabilizes the Glycolytic Enzyme ENO1 Through Its Catalytic Activity

**FIGURE 3:**
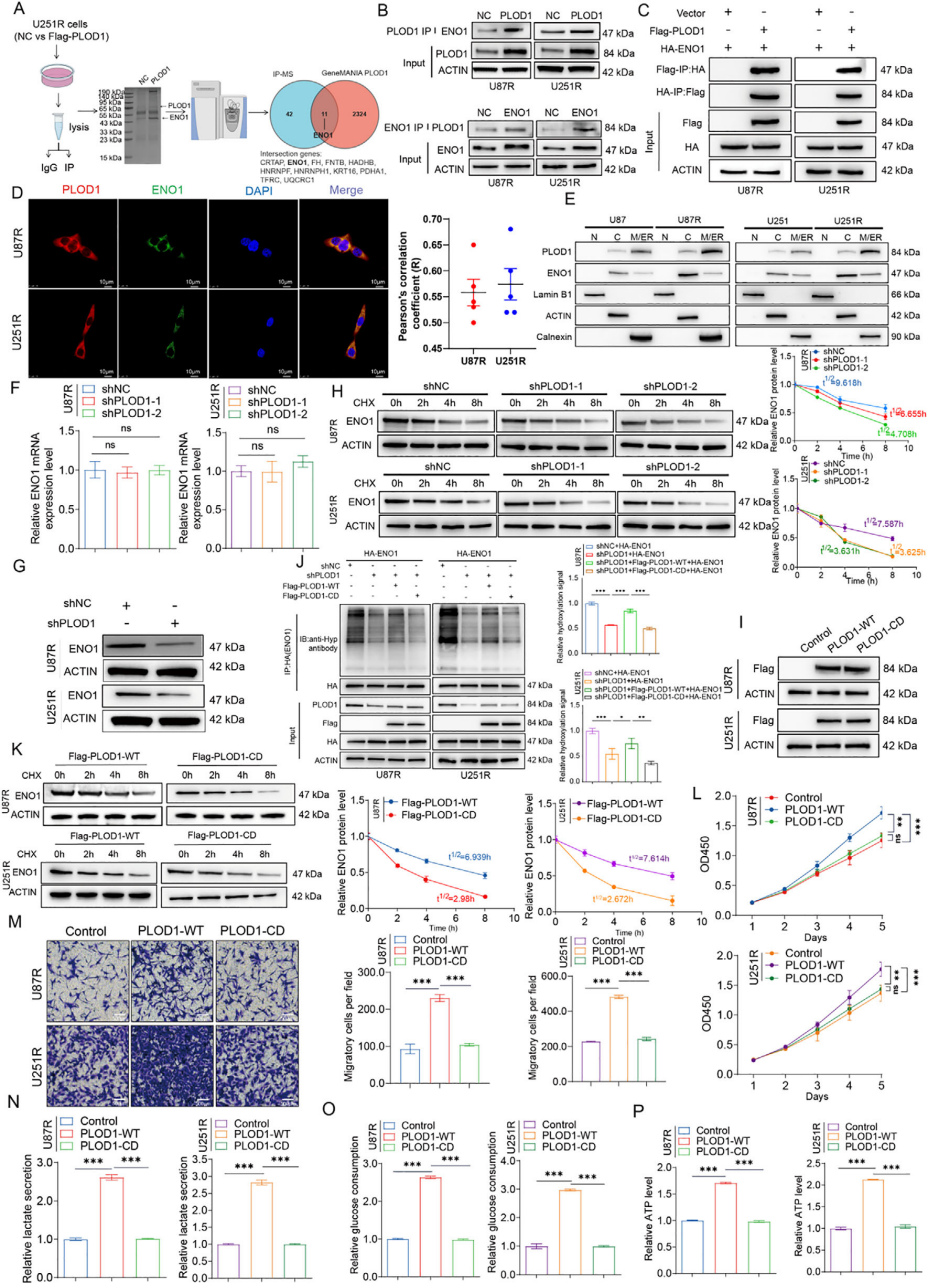
PLOD1 Stabilizes the Glycolytic Enzyme ENO1 through Its Catalytic Activity. (A) Schematic of the IP‐MS screen used to identify ENO1 as a key PLOD1‐interacting protein. (B, C) Reciprocal co‐IP validates the interaction between PLOD1 and ENO1 at the endogenous level (B) and with exogenously expressed proteins (C) in glioblastoma cells. (D) Representative immunofluorescence images showing the intracellular co‐localization of PLOD1 (red) and ENO1 (green) in U87‐R and U251‐R cells. Nuclei were counterstained with DAPI (blue). Pearson's correlation coefficients (PCCs) were quantified to evaluate the degree of co‐localization. Scale bar, 10 µm. (E) Subcellular fractionation followed by western blot analysis showing that PLOD1 is predominantly enriched in the membrane/endoplasmic reticulum‐associated (M/ER) fraction, while a detectable cytosolic pool co‐fractionates with ENO1. Lamin B1, ACTIN, and Calnexin were used as markers for the nuclear (N), cytoplasmic (C), and membrane/ER‐associated fractions, respectively. (F, G) qRT‐PCR (F) and western blot (G) analyses showing that PLOD1 knockdown does not alter ENO1 mRNA levels but reduces ENO1 protein expression. (H) Cycloheximide (CHX) chase assay and corresponding quantification showing that PLOD1 knockdown accelerates ENO1 protein degradation. (I) Western blot analysis confirming comparable expression levels of exogenously expressed Flag‐tagged PLOD1‐WT and PLOD1‐CD constructs in U87‐R and U251‐R cells after rescue expression. (J) Exogenously expressed HA‐ENO1 was immunoprecipitated and subjected to immunoblotting using an anti‐Hyp antibody to assess ENO1‐associated hydroxylation signals. Although this antibody detects hydroxylation‐associated epitopes rather than a specific lysine hydroxylation site, PLOD1 depletion reduced the hydroxylation‐associated signal detected on immunoprecipitated HA‐ENO1, which was restored by re‐expression of PLOD1‐WT but not the catalytically inactive PLOD1‐CD mutant. The anti‐Hyp signal corresponding to immunoprecipitated HA‐ENO1 (∼47 kDa) was quantified and normalized to the amount of HA‐ENO1 recovered in the immunoprecipitation. (K) CHX chase assays and corresponding quantification showing that the catalytically inactive PLOD1‐CD mutant fails to maintain ENO1 protein stability compared with PLOD1‐WT. (L‐P) Re‐expression of PLOD1‐WT, but not the catalytically inactive PLOD1‐CD, successfully promoted cellular proliferation (L), Representative Transwell images and quantitative analysis of cell migration are shown in (M) Scale bar = 500 µm, lactate secretion (N), glucose consumption (O), and ATP production (P). For quantitative in vitro experiments, data are presented as mean ± SD from three independent experiments (*n* = 3), unless otherwise indicated. Statistical significance was determined using one‐way or two‐way ANOVA, followed by Tukey's multiple‐comparisons test. All statistical tests were two‐sided. ^*^ *p* < 0.05, ^**^ *p* < 0.01, and ^***^ *p* < 0.001; ns indicates no statistically significant difference.

To identify a candidate downstream effector linking PLOD1 to this hyper‐glycolytic phenotype, we performed an unbiased immunoprecipitation‐mass spectrometry (IP‐MS) screen to map the PLOD1 interactome in U251R cells. To maximize the confidence of candidate selection, we intersected our IP‐MS dataset with the GeneMANIA‐predicted PLOD1 interaction network, yielding 11 high‐confidence overlapping proteins (CRTAP, ENO1, FH, FNTB, HADHB, HNRNPF, HNRNPH1, KRT16, PDHA1, TFRC, and UQCRC1) (Figure 3A). Among these candidates, ENO1 was the only enzyme directly executing the classical glycolytic pathway, whereas the others were primarily involved in mitochondrial metabolism, RNA processing, or structural organization. Given our strong functional evidence that PLOD1 predominantly drives glycolytic reprogramming, we prioritized ENO1 as the most biologically relevant downstream target for in‐depth mechanistic investigation. We validated this physical association through a series of experiments. Reciprocal co‐IP assays in both U87R and U251‐R cells confirmed a robust interaction between PLOD1 and ENO1 (Figure 3B,C). Furthermore, high‐resolution confocal immunofluorescence analysis revealed significant co‐localization of PLOD1 and ENO1 within the cytoplasm, which we quantitatively confirmed using Pearson's correlation coefficient (Figure 3D). Because PLOD1 is canonically recognized as an endoplasmic reticulum (ER)‐resident protein, we sought to definitively confirm its physical presence in the cytosol where glycolysis occurs. Notably, recent evidence has begun to uncover non‐canonical cytosolic roles for PLOD1, such as its interaction with the cytoskeletal protein Septin2 [56]. To biochemically validate this cytosolic pool in our model, we performed subcellular fractionation assays. As expected, PLOD1 was abundantly detected in the membrane/ER fraction (marked by Calnexin). However, crucially, a substantial pool of PLOD1 was also clearly present in the cytosolic fraction (marked by Actin), where it co‐fractionated with ENO1 across multiple GBM cell lines (Figure 3E). This biochemical evidence substantiates a non‐canonical, cytosolic localization for PLOD1, which is compatible with the observed interaction with ENO1.

We next determined that this physical interaction leads to the post‐transcriptional regulation of ENO1. While depletion of PLOD1 did not significantly alter ENO1 mRNA levels (Figure 3F), it triggered a marked reduction in ENO1 protein expression (Figure 3G). Critically, CHX chase assays utilizing two independent shRNAs and subsequent quantitative analysis, revealed that the half‐life of the ENO1 protein was significantly shortened in PLOD1‐knockdown cells, demonstrating that PLOD1 is required for ENO1 protein stability (Figure 3H).

Given that PLOD1 is a lysyl hydroxylase [57], we generated a catalytically dead (CD) PLOD1 mutant and re‐expressed it alongside its wild‐type (WT) counterpart in PLOD1‐depleted glioblastoma cells to determine whether its enzymatic activity is required for ENO1 regulation. Western blot analysis confirmed that both the Flag‐tagged PLOD1‐WT and PLOD1‐CD constructs were successfully overexpressed at comparable levels in these cells (Figure 3I). We next examined whether PLOD1 catalytic activity affects ENO1‐associated hydroxylation modification. HA‐tagged ENO1 was immunoprecipitated and subjected to immunoblotting using an anti‐Hyp antibody to detect hydroxylation‐associated signals. Although this antibody recognizes hydroxylation‐related epitopes rather than a specific lysine hydroxylation event, PLOD1 depletion markedly reduced the hydroxylation‐associated signal detected on immunoprecipitated ENO1. Importantly, re‐expression of wild‐type PLOD1 restored this signal, whereas the catalytically inactive PLOD1‐CD mutant failed to rescue the modification (Figure 3J). These findings indicate that PLOD1 catalytic activity contributes to regulating ENO1‐associated hydroxylation modification. Consistently, a CHX assay revealed that the half‐life of ENO1 was markedly shortened when a catalytically dead (CD) PLOD1 mutant was expressed, whereas wild‐type PLOD1 preserved ENO1 stability (Figure 3K). Crucially, this loss of enzymatic function completely abrogated the oncogenic effects of PLOD1. In stark contrast to its WT counterpart, the PLOD1‐CD mutant was unable to promote cell proliferation (Figure 3L), enhance migration (Figure 3M), or drive the glycolytic phenotype, as evidenced by its failure to increase lactate secretion, glucose consumption, or cellular ATP levels (Figure 3N–P). Collectively, these data support a model in which PLOD1 catalytic activity regulates ENO1‐associated hydroxylation signals and stabilizes ENO1, thereby enabling glycolysis and malignant progression in glioblastoma.

### 3.4 PLOD1 Catalytic Activity Shields ENO1 From FBXW7‐Targeted Ubiquitination and Proteasomal Degradation

**FIGURE 4:**
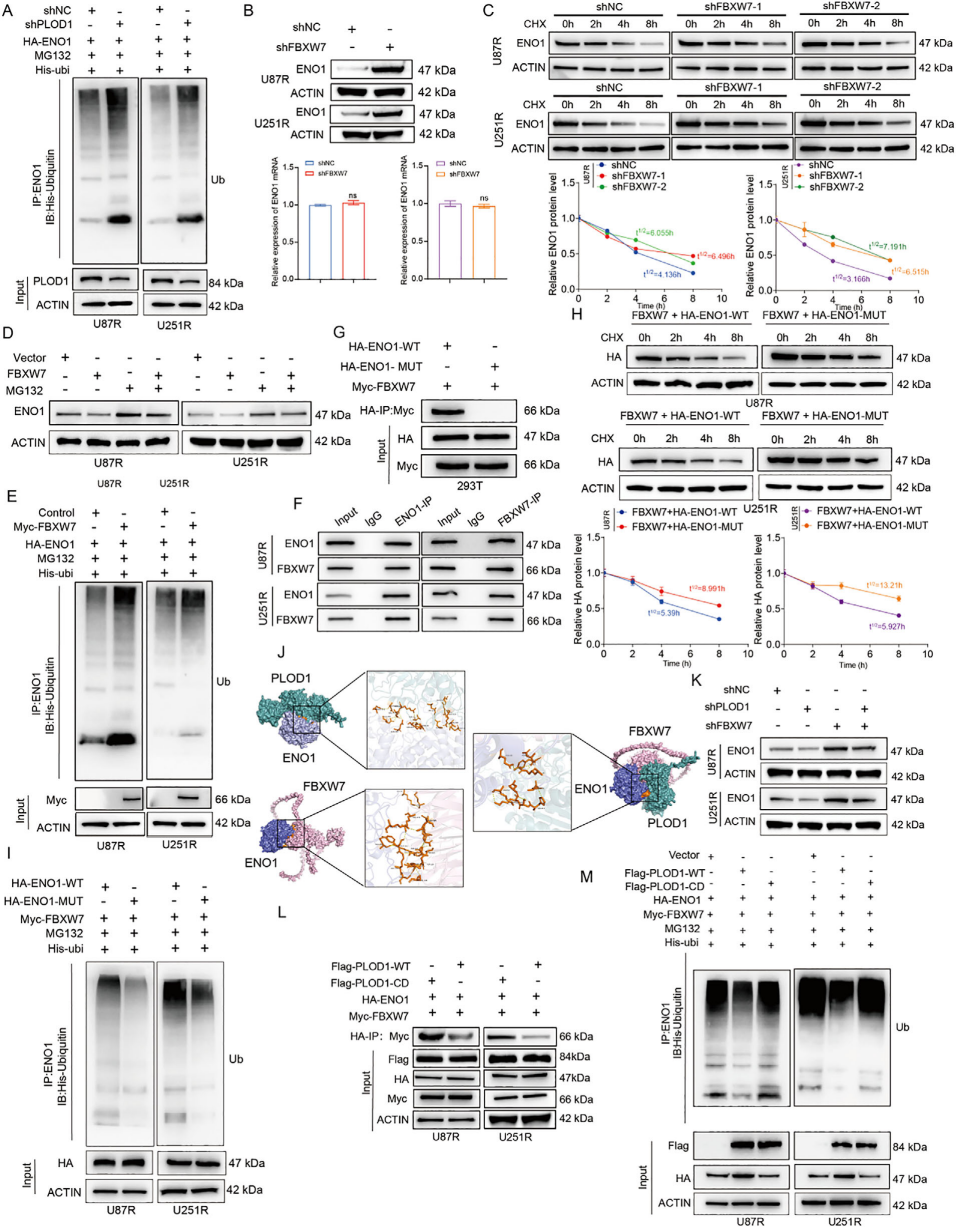
PLOD1 protects ENO1 from FBXW7‐mediated degradation. (A) In vitro ubiquitination assay demonstrates that knockdown of PLOD1 enhances the ubiquitination of HA‐ENO1 in U87‐R and U251‐R cells. (B) Western blot analysis showing that knockdown of the E3 ligase FBXW7 leads to an increase in endogenous ENO1 protein levels without affecting ENO1 mRNA abundance (below panel). ns, not significant (quantified below). (C) Cycloheximide (CHX) chase assay reveals that knockdown of FBXW7 significantly increases the stability of the ENO1 protein. (D) Overexpression of FBXW7 reduces ENO1 protein levels, an effect that is reversed by treatment with the proteasome inhibitor MG132. (E) In vitro ubiquitination assay showing that overexpression of Myc‐FBXW7 markedly promotes the ubiquitination of HA‐ENO1. (F) Endogenous co‐immunoprecipitation assays demonstrating physiological interactions between ENO1 and FBXW7 in GBM cells. (G) Exogenous co‐immunoprecipitation assays in 293T cells showing that mutation of the predicted FBXW7‐recognition motif markedly weakens the interaction between HA‐ENO1 and Myc‐FBXW7. (H) CHX chase assays demonstrating that ENO1‐MUT exhibits enhanced protein stability compared with ENO1‐WT in both U87R and U251R cells. (I) In vitro ubiquitination assays showing that ENO1‐MUT displays substantially reduced polyubiquitination compared with ENO1‐WT following FBXW7 overexpression. (J) Structural modeling illustrating the spatial relationship between the candidate PLOD1‐regulated region and the predicted FBXW7‐recognition interface on ENO1. (K) Western blot analysis showing that simultaneous depletion of FBXW7 largely rescues the reduction in ENO1 protein levels induced by PLOD1 knockdown. (L) Co‐immunoprecipitation assays demonstrating that wild‐type PLOD1 (PLOD1‐WT), but not the catalytic‐dead mutant (PLOD1‐CD), disrupts the interaction between ENO1 and FBXW7. (M) In vitro ubiquitination assays showing that PLOD1‐WT, but not PLOD1‐CD, suppresses FBXW7‐mediated polyubiquitination of ENO1. For quantitative in vitro experiments, data are presented as mean ± SD from three independent experiments (*n* = 3), unless otherwise indicated. Statistical significance between two groups was determined using a two‐tailed Student's t‐test. All statistical tests were two‐sided. ns indicates no statistically significant difference.

Having identified PLOD1 as an upstream stabilizer of ENO1, we sought to dissect the underlying post‐translational mechanism. We hypothesized that PLOD1‐mediated hydroxylation might protect ENO1 from degradation via the ubiquitin‐proteasome system. To test this, we first performed an in vitro ubiquitination assay. Results revealed that genetic depletion of PLOD1 markedly increased the polyubiquitination levels of ENO1, suggesting that PLOD1 acts as a shield against ubiquitin‐mediated degradation (Figure 4A). We next aimed to identify the specific E3 ligase responsible for this process, prioritizing FBXW7 due to its known role in regulating metabolic enzymes. Knockdown of FBXW7 significantly increased steady‐state ENO1 protein levels without altering ENO1 mRNA abundance, pointing to post‐translational regulation (Figure 4B). This observation was substantiated by cycloheximide (CHX) chase assays, which demonstrated that FBXW7 depletion significantly extended the half‐life of ENO1 (Figure 4C). Crucially, this regulation was confirmed to be proteasome‐dependent, as the reduction in ENO1 levels driven by FBXW7 overexpression was effectively rescued by the proteasome inhibitor MG132 (Figure 4D). Consistent with this, overexpression of FBXW7 promoted the robust polyubiquitination of ENO1 (Figure 4E). To determine whether FBXW7 directly interacts with ENO1, endogenous co‐immunoprecipitation assays were performed in GBM cells. ENO1 efficiently co‐precipitated with FBXW7, confirming the existence of a physiological interaction between the two proteins (Figure 4F). Previous studies have implicated residues S263 and S268 of ENO1 in FBXW7‐mediated recognition [58]. Guided by these observations, together with sequence motif analysis and structural docking predictions, we identified the SPDDPS‐containing region (residues 263–268) as the most likely FBXW7‐binding interface. We therefore generated an ENO1 mutant (ENO1‐MUT) carrying S263A/S268A substitutions within this region to disrupt the predicted ENO1–FBXW7 interaction. Co‐immunoprecipitation assays revealed that mutation of this motif markedly weakened FBXW7 binding compared with wild‐type ENO1 (Figure 4G), indicating that this region is required for efficient substrate recognition. Consequently, the ENO1‐MUT variant displayed significantly enhanced protein stability (Figure 4H) and reduced polyubiquitination levels (Figure 4I), confirming that this specific interface is required for FBXW7‐mediated targeting. We next investigated how PLOD1 antagonizes FBXW7‐dependent ENO1 turnover. Structural modeling revealed that the predicted PLOD1‐binding interface is located in close spatial proximity to the putative FBXW7‐recognition interface on ENO1 (Figure 4J), suggesting that PLOD1‐mediated hydroxylation may interfere with FBXW7 recruitment and substrate recognition. To establish the functional hierarchy of this axis, we performed genetic epistasis experiments. While PLOD1 knockdown reduced ENO1 levels, this reduction was effectively rescued by the simultaneous knockdown of FBXW7, placing PLOD1 upstream of FBXW7 in the regulation of ENO1 stability (Figure 4K).

Finally, we examined whether this protection relied on PLOD1 enzymatic activity. Co‐IP assays demonstrated that wild‐type (WT) PLOD1, but not a catalytically dead (CD) mutant, potently disrupted the physical interaction between ENO1 and FBXW7 (Figure 4L). This enzymatic dependency was further validated by in vitro ubiquitination assays, which showed that only WT PLOD1 was capable of inhibiting FBXW7‐mediated polyubiquitination of ENO1, whereas the CD mutant failed to do so (Figure 4M). Collectively, these findings support a model in which PLOD1 catalytic activity reduces FBXW7 recruitment to ENO1, thereby preventing ubiquitin‐dependent proteasomal degradation and maintaining ENO1 protein stability.

### 3.5 ENO1 and a K60‐Dependent Stabilization Mechanism Mediate the Oncogenic Functions of PLOD1

To achieve deeper mechanistic insight, we sought to identify the precise lysine residue(s) on ENO1 targeted by PLOD1. We first attempted to map the exact modification site using high‐resolution LC‐MS/MS on ENO1 immunoprecipitated from PLOD1‐overexpressing cells. Although lysine oxidation‐containing peptides were readily detected elsewhere in the dataset, no ENO1‐derived peptide carrying confidently localized lysine oxidation was identified, likely reflecting the low abundance of the modified peptide and the incomplete sequence coverage inherent to discovery‐based DDA proteomics. (Figure S8A–C). As a complementary functional approach, we prioritized candidate lysine residues for mutational analysis. We cataloged all lysine residues on ENO1 matching the canonical (X‐K‐G) or extended (X‐K‐A/S) PLOD recognition motifs and structurally mapped them onto the ENO1 crystal surface (Figure S8D).

**FIGURE 5:**
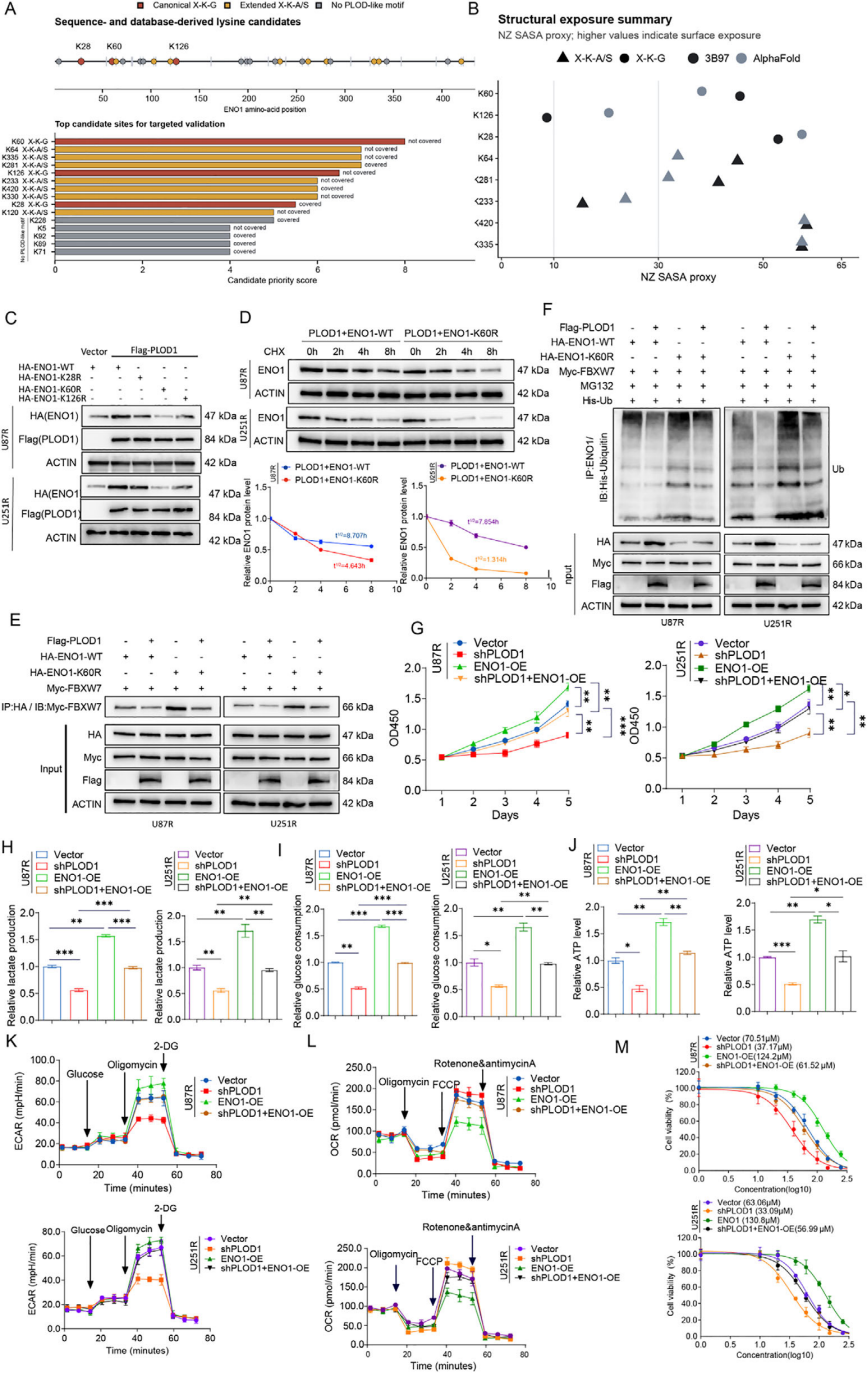
K60 is a functionally critical candidate residue required for PLOD1‐dependent stabilization of ENO1 and downstream metabolic reprogramming. (A) Schematic overview of candidate lysine residue prioritization. Candidate lysine residues were ranked by integrating LC‐MS/MS peptide coverage, PLOD1 substrate motif prediction, evolutionary conservation, and structural accessibility analyses. K60 emerged as the highest‐priority candidate for experimental validation. (B) Structural exposure analysis of candidate lysine residues based on the ENO1 crystal structure (PDB ID:3B97) and an AlphaFold‐predicted model (UniProt ID: P06733). Solvent accessibility and structural localization were evaluated to identify residues prioritized for functional evaluation. (C) Immunoblot analysis of ENO1 protein expression following ectopic expression of wild‐type ENO1 or individual lysine‐to‐arginine mutants in the presence or absence of PLOD1 overexpression. (D) Cycloheximide (CHX) chase assays demonstrating that the K60R mutation impaired PLOD1‐mediated stabilization of ENO1 in U87R and U251R cells. (E) Co‐immunoprecipitation assays showing that mutation of K60 restores FBXW7 binding despite PLOD1 overexpression. (F)In vitro ubiquitination assays demonstrating that the K60R mutation impaired the ability of PLOD1 to suppress FBXW7‐mediated polyubiquitination of ENO1. (G) CCK‐8 assays showing that ENO1 re‐expression rescues the proliferation defect induced by PLOD1 depletion in TMZ‐resistant GBM cells. (H‐J) Quantification of lactate production (H), glucose consumption (I), and intracellular ATP levels (J), demonstrating that restoration of ENO1 reverses the metabolic defects caused by PLOD1 silencing. (K) Extracellular acidification rate (ECAR) analysis showing that ENO1 re‐expression restores glycolytic activity suppressed by PLOD1 knockdown. (L) Oxygen consumption rate (OCR) analysis demonstrating that ENO1 restoration reverses the increase in mitochondrial respiration induced by PLOD1 depletion. (M) Dose‐response curves and IC50 analyses of TMZ sensitivity. ENO1 re‐expression partially restores TMZ resistance in PLOD1‐depleted GBM cells. Data are presented as mean ± SD from three independent experiments (*n* = 3). Statistical significance for (G‐J) was determined using one‐way ANOVA followed by Tukey's multiple‐comparisons test. IC50 values in (M) were calculated by nonlinear regression of the dose‐response curves. All statistical tests were two‐sided. ^*^ *p* < 0.05, ^**^ *p* < 0.01, and ^***^ *p* < 0.001; ns indicates no statistically significant difference.

Among the candidate residues, K28, K60, and K126 emerged as the highest‐priority sites because they were located within canonical PLOD‐like recognition motifs and exhibited favorable structural accessibility (Figure 5A,B). To experimentally determine which residue mediates PLOD1‐dependent ENO1 stabilization, lysine‐to‐arginine mutants (K28R, K60R, and K126R) were generated and co‐expressed with PLOD1. While PLOD1 markedly increased the abundance of wild‐type ENO1, mutation of K60 substantially impaired this stabilizing effect. In contrast, K28R and K126R only partially attenuated PLOD1‐mediated ENO1 accumulation (Figure 5C). These findings suggest that K60 is the most functionally relevant candidate residue involved in PLOD1‐dependent ENO1 stabilization. To further assess the contribution of K60 to ENO1 protein turnover, cycloheximide chase assays were performed. Consistent with the protein expression results, PLOD1 markedly prolonged the half‐life of wild‐type ENO1, whereas the ENO1‐K60R mutant exhibited accelerated degradation despite comparable PLOD1 expression levels (Figure 5D).

Because our previous findings identified FBXW7 as the E3 ubiquitin ligase responsible for ENO1 degradation, we next investigated whether K60 regulates the interaction between ENO1 and FBXW7. Co‐immunoprecipitation assays demonstrated that PLOD1 markedly reduced the association between FBXW7 and wild‐type ENO1. In contrast, mutation of K60 substantially restored FBXW7 binding despite equivalent PLOD1 expression levels (Figure 5E), indicating that K60 is required for the ability of PLOD1 to disrupt the ENO1–FBXW7 interaction. These findings indicated that K60 is required for the PLOD1‐dependent reduction in ENO1‐FBXW7 association. Consistent with this observation, ubiquitination assays revealed that PLOD1 markedly suppressed polyubiquitination of wild‐type ENO1. However, this protective effect was largely abolished in the ENO1‐K60R mutant, which displayed substantially increased ubiquitin accumulation under identical experimental conditions (Figure 5F). Together, these data indicate that K60 is essential for PLOD1‐mediated protection of ENO1 from FBXW7‐dependent ubiquitin‐proteasome degradation.

Having shown that PLOD1 promotes ENO1 stability, we next examined whether ENO1 mediates the downstream effects of PLOD1. Indeed, re‐expression of ENO1 was sufficient to substantially rescue the proliferative defects (Figure 5G). This functional rescue extended to the metabolic program; ENO1 overexpression restored lactate production, glucose consumption, and cellular ATP to levels comparable to or exceeding those of control cells (Figure 5H–J). Consistent with this, metabolic flux analysis confirmed that ENO1 re‐expression prompted a complete metabolic reversal in PLOD1‐depleted cells. It not only normalized their suppressed extracellular acidification rate (ECAR), reviving glycolysis (Figure 5K), but also concurrently suppressed their compensatory increase in the oxygen consumption rate (OCR), thereby reducing their reliance on mitochondrial respiration (Figure 5L).

Most critically, re‐expression of ENO1 was sufficient to reinstate chemoresistance. While PLOD1 depletion sensitized U87‐R and U251‐R cells to TMZ, concomitant overexpression of ENO1 substantially restored their resistance, as evidenced by a marked increase in their IC_50_ values (Figure 5M). Collectively, these findings establish ENO1 as a major downstream effector of the PLOD1 signaling axis. Although definitive site‐level hydroxylation evidence remains to be obtained, our mutational and functional analyses identify K60 as a critical residue required for PLOD1‐mediated ENO1 stabilization and protection from FBXW7‐dependent ubiquitination, thereby sustaining glycolytic reprogramming and TMZ resistance in glioblastoma cells.

### 3.6 A Dual‐Domain Mechanism Governs PLOD1's Interaction With and Stabilization of ENO1

**FIGURE 6:**
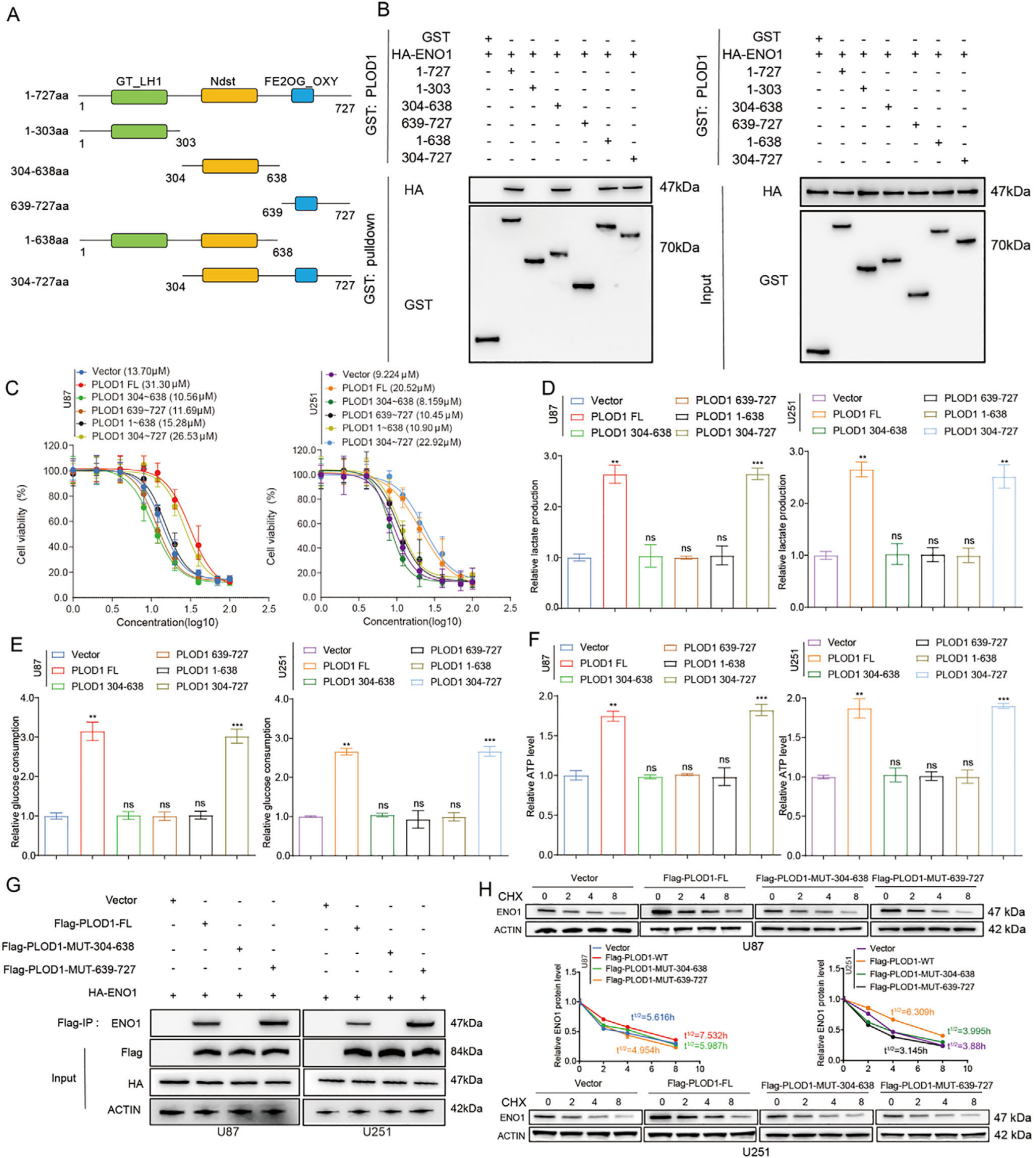
Coordinated action of the Ndst and catalytic domains is essential for PLOD1 function. (A, B) A schematic of PLOD1 truncation mutants (A) was used in GST pull‐down assays (B) to map the essential ENO1‐binding site to the Ndst domain (aa 304–638). (C‐F) Structure‐function analyses in U87 and U251 cells demonstrated that the coordinated action of both the Ndst and catalytic domains is required to enhance chemoresistance to TMZ (C), as well as to increase lactate secretion (D), glucose consumption (E), and cellular ATP levels (F). (G) Co‐immunoprecipitation confirmed that mutation of the Ndst domain (MUT‐304‐638) dramatically weakens the PLOD1‐ENO1 interaction in cells. (H) A subsequent CHX chase assay revealed that wild‐type PLOD1 stabilizes the ENO1 protein. This stabilization was completely abolished by mutations in either the Ndst domain (aa 304–638) or the catalytic domain region (aa 639–727), indicating that both domains are essential for this function. Data in D‐F are presented as mean ± SD (*n* = 3). Statistical significance was determined using one‐way ANOVA followed by Tukey's multiple‐comparisons test. ns, not significant; ^**^ *p* < 0.01; ^***^ *p* < 0.001.

To delineate the functional domains of PLOD1 governing its interaction with and stabilization of ENO1, we first analyzed its domain architecture, identifying an N‐terminal GT_LH1 domain (aa 1–303), a central Ndst domain (aa 304–638), and a C‐terminal FE2OG_OXY catalytic domain (aa 639–727) (Figure 6A). Through GST pull‐down assays using a series of truncated mutants, we determined that the Ndst domain (aa 304–638) was the primary region responsible for binding to ENO1 (Figure 6B). However, functional assays revealed that the ability to bind ENO1 alone was insufficient for PLOD1's oncogenic activity. In glioblastoma cells, only full‐length PLOD1 (PLOD1‐FL) and a construct containing both the Ndst and catalytic domains (aa 304–727) could confer chemoresistance and enhance metabolic activity, whereas constructs lacking the catalytic domain were functionally inactive (Figure 6C–F). These findings led us to hypothesize a two‐step mechanism: PLOD1 first recognizes and binds ENO1 via its Ndst domain, and then its FE2OG_OXY domain catalyzes a modification that leads to ENO1 stabilization and subsequent oncogenic effects.

To validate this hypothesis, we generated mutants within the Ndst and catalytic domains. Co‐immunoprecipitation experiments confirmed that a mutation in the Ndst domain (aa 304–638) abrogated the interaction with ENO1. In contrast, a mutation in the catalytic domain (aa 639–727) did not prevent PLOD1 from binding to ENO1, confirming the Ndst domain's role as the primary binding interface (Figure 6G). Crucially, a CHX chase assay demonstrated that both mutants failed to prolong the half‐life of ENO1. The binding‐deficient mutant could not engage its substrate, while the catalysis‐deficient mutant, despite binding, was unable to confer stability (Figure 6H). Thus, PLOD1 acquires the ability to promote glioblastoma malignancy, depending on the coordinated action of two distinct domains: the Ndst domain for substrate recognition and binding, and the C‐terminal catalytic domain for subsequent stabilization of ENO1.

### 3.7 The PLOD1‐ENO1 Axis Drives Glioma Progression and Chemoresistance In Vivo

**FIGURE 7:**
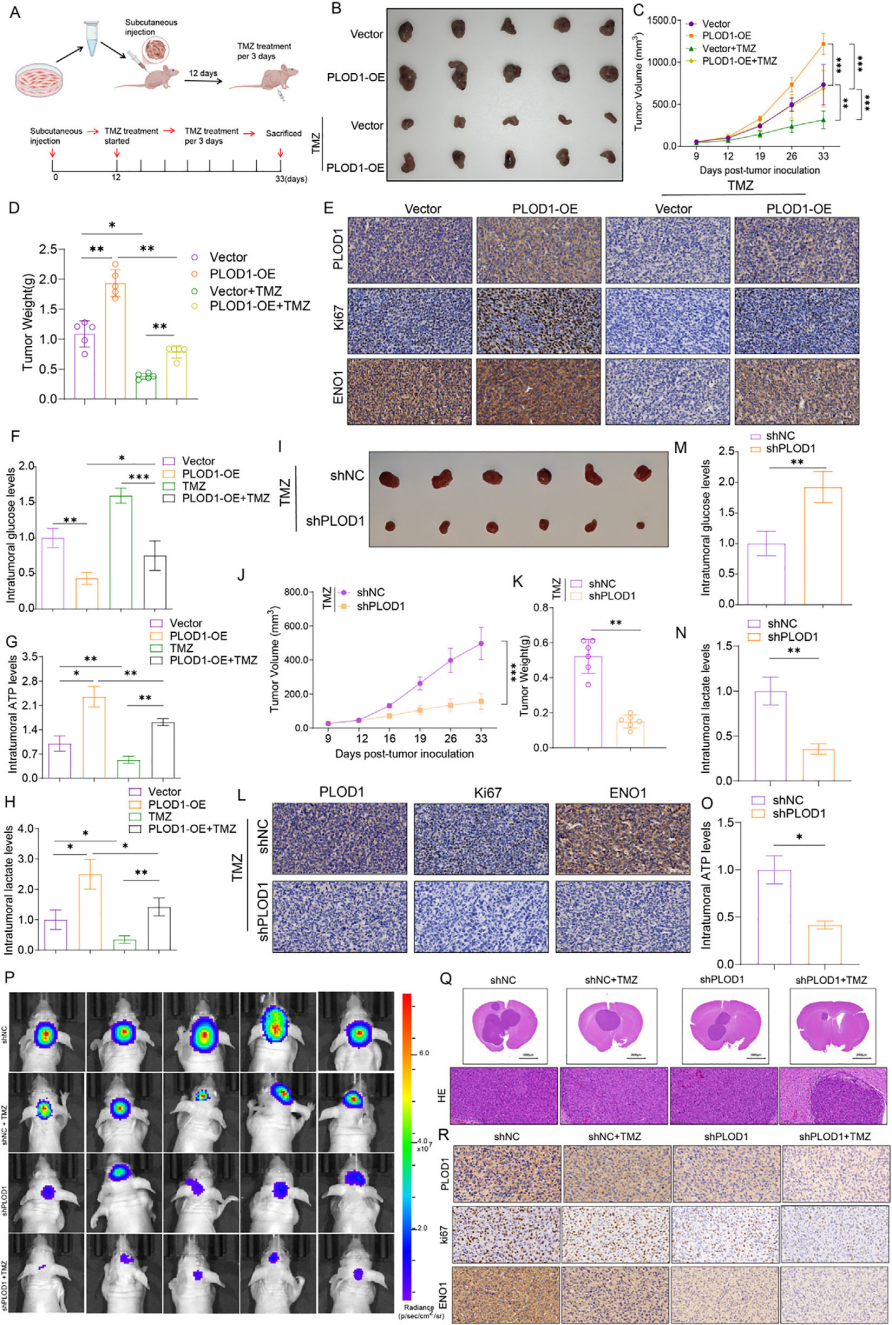
The PLOD1‐ENO1 axis drives glioma progression and chemoresistance in vivo. (A) Schematic illustration of the subcutaneous xenograft model and TMZ treatment schedule. (B‐H) In vivo characterization of xenografts established with parental U87 cells stably overexpressing PLOD1 or vector control (n = 5 per group). (B) Representative images of excised xenograft tumors. (C) Tumor growth curves during TMZ treatment. (D) Quantification of tumor weights at endpoint. (E) Representative immunohistochemical staining of xenograft sections for PLOD1, Ki67, and ENO1. Scale bar, 50 µm. (F–H) Quantification of intratumoral glucose levels (F), ATP levels (G), and lactate levels (H) in tumor tissue homogenates. Metabolic measurements were normalized to tumor wet weight. (I‐O) In vivo evaluation of TMZ‐resistant U87R xenografts with stable PLOD1 depletion (shPLOD1) or control shRNA (shNC) (n = 6 per group). (I) Representative images of excised xenograft tumors. (J) Tumor growth curves following TMZ treatment. (K) Quantification of tumor weights at endpoint. (L) Representative immunohistochemical staining for PLOD1, Ki67, and ENO1 in xenograft tissues. Scale bar, 50 µm. (M–O) Analysis of intratumoral glucose levels (M), lactate levels (N), and ATP levels (O) in tumor tissue homogenates. Metabolic measurements were normalized to tumor wet weight. (P‐R) Validation of the PLOD1‐ENO1 axis in an orthotopic intracranial GBM xenograft model. TMZ‐resistant U87R cells expressing shNC or shPLOD1 were stereotactically implanted into the mouse striatum (*n* = 5 per group). (P) Representative bioluminescence imaging (BLI) images showing intracranial tumor progression under different treatment conditions. (Q) Representative hematoxylin and eosin (H&E) staining of brain sections demonstrating intracranial tumor burden. Scale bar, 2 mm. (R) Representative immunohistochemical staining of intracranial tumors for PLOD1, Ki67, and ENO1. Scale bar, 50 µm. Data are presented as mean ± SD. Statistical significance was determined using two‐way ANOVA followed by Tukey's multiple‐comparisons test for the tumor growth curves in (C, J), one‐way ANOVA followed by Tukey's multiple‐comparisons test for the endpoint comparisons in (D, F‐H), and a two‐tailed Student's t‐test for the two‐group comparisons in (K, M‐O). All statistical tests were two‐sided. ^*^ *p* < 0.05, ^**^ *p* < 0.01, and ^***^ *p* < 0.001; ns indicates no statistically significant difference.

To determine the in vivo relevance of our findings, we established a subcutaneous glioma xenograft model using cells stably overexpressing PLOD1. Consistent with our in vitro results, tumors derived from PLOD1‐overexpressing cells exhibited markedly accelerated growth. Critically, these tumors also displayed marked attenuation of TMZ efficacy, which effectively suppressed the growth of control tumors (Figure 7A–D). IHC analysis of resected tumors confirmed the in vivo relevance of our proposed mechanism. PLOD1‐overexpressing tumors displayed elevated levels of the proliferation marker Ki67 and, importantly, showed robust accumulation of ENO1 protein (Figure 7E). This was accompanied by a glycolysis‐associated metabolic profile, reflected by decreased intratumoral glucose levels together with increased intratumoral lactate and ATP levels (Figure 7F–H).

In a complementary model, we found that targeting PLOD1 could overcome chemoresistance. Genetic depletion of PLOD1 in chemoresistant xenografts not only suppressed tumor growth but, critically, re‐sensitized them to TMZ therapy (Figure 7I–K). This therapeutic response was associated with a marked reduction in Ki67 staining and a striking loss of ENO1 protein (Figure 7L). Consistently, suppression of the PLOD1‐ENO1 axis resulted in profound attenuation of tumor glycolytic activity, as reflected by increased intratumoral glucose levels and decreased intratumoral lactate and ATP levels (Figure 7M–O). Given the limitations of subcutaneous models in recapitulating the brain tumor microenvironment, we further validated the role of the PLOD1‐ENO1 axis using an orthotopic intracranial GBM xenograft model established with TMZ‐resistant U87R cells expressing shNC or shPLOD1. Bioluminescence imaging revealed that PLOD1 depletion significantly suppressed intracranial tumor progression, and this inhibitory effect was further enhanced following TMZ treatment (Figure 7P). Consistently, hematoxylin and eosin staining demonstrated reduced intracranial tumor burden in mice bearing shPLOD1 tumors (Figure 7Q). Immunohistochemical analysis further confirmed decreased PLOD1 expression accompanied by reduced Ki67 positivity and diminished ENO1 accumulation after PLOD1 depletion (Figure 7R), providing additional evidence that the PLOD1‐ENO1 axis sustains malignant growth and therapeutic resistance within the brain tumor microenvironment.

Taken together, these in vivo data demonstrate that the PLOD1‐ENO1 axis is a critical driver of glioma progression and chemoresistance, which it achieves by reprogramming tumor metabolism.

### 3.8 The PLOD1‐ENO1 Axis is Clinically Relevant and Prognostic for Poor Survival in Glioma Patients

**FIGURE 8:**
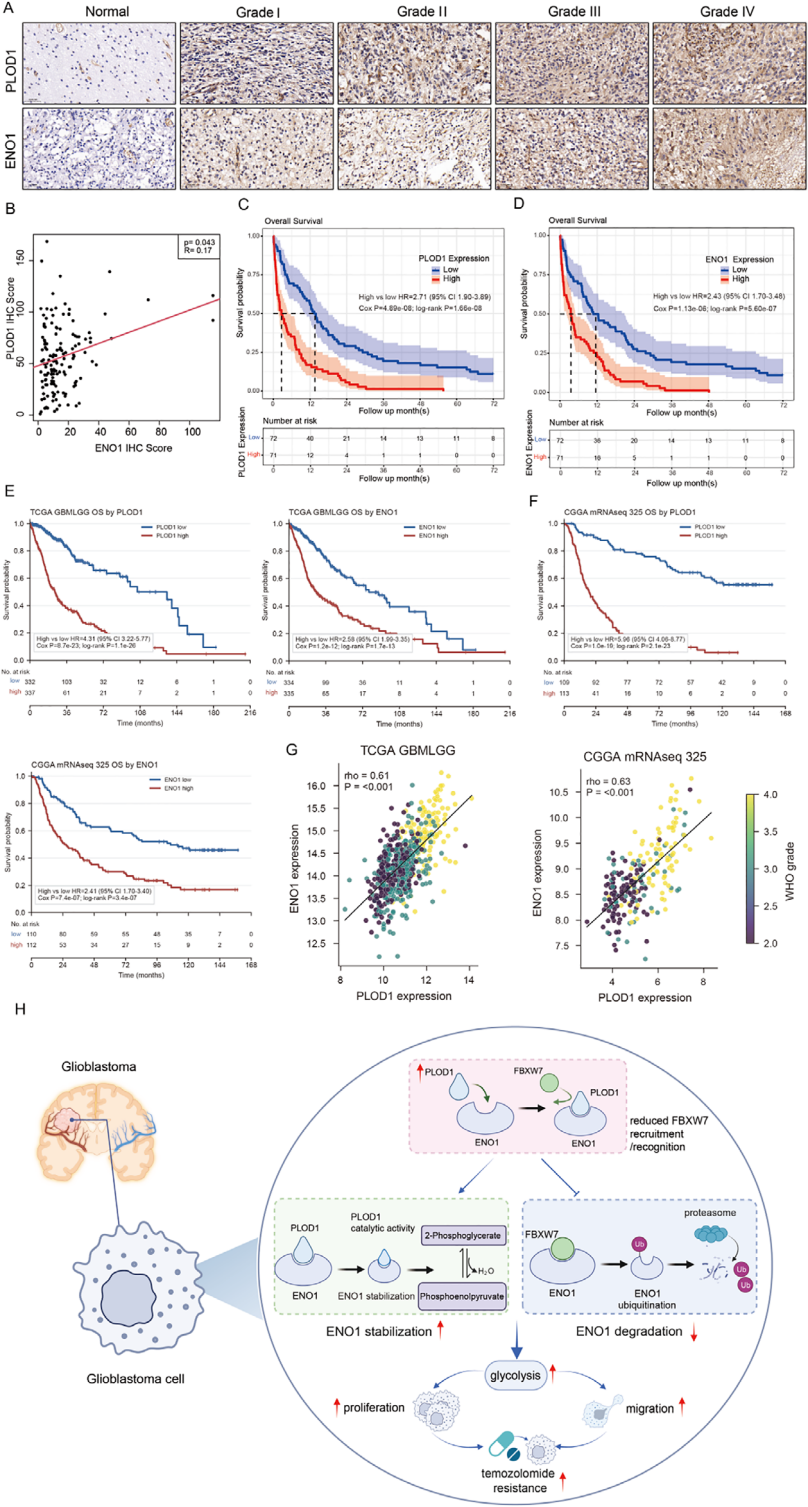
The PLOD1‐ENO1 axis is clinically relevant and predicts poor survival in glioma. (A) Representative IHC images of PLOD1 and ENO1 expression in adjacent normal brain and glioma tissues of WHO grade I‐IV. Scale bar, 100 µm. (B) Scatter plot showing a significant positive correlation (Pearson r = 0.17, *p* = 0.043) between PLOD1 and ENO1 IHC scores in a cohort of 143 glioma patients. (C, D) Kaplan‐Meier survival analysis of glioma patients stratified by median expression of PLOD1 (C) or ENO1 (D). *P*‐values were determined by the log‐rank test. (E) Validation of the prognostic significance of PLOD1 and ENO1 in the TCGA‐GBMLGG cohort. Kaplan–Meier analyses demonstrate significantly shorter overall survival in patients with high PLOD1 or ENO1 expression. (F) Validation of the prognostic significance of PLOD1 and ENO1 in the CGGA mRNAseq_325 cohort. Elevated expression of either gene is associated with unfavorable overall survival. (G) Correlation analyses of PLOD1 and ENO1 mRNA expression in the TCGA‐GBMLGG and CGGA mRNAseq_325 datasets. Spearman correlation coefficients (rho) and corresponding *p* values are shown. Dot colors indicate WHO tumor grade. (H) Schematic model illustrating how PLOD1 stabilizes ENO1, promotes glycolytic reprogramming, and drives glioma progression and TMZ resistance. Created in BioRender. chang, W. (2026). [https://BioRender.com/9w64yej].

To establish the clinical relevance of our mechanistic findings, we examined the expression of PLOD1 and ENO1 in human glioma specimens. IHC analysis revealed that both proteins were markedly elevated in glioma tissues compared to adjacent normal brain and that their expression progressively increased with advancing tumor grade (Figure 8A). IHC analysis revealed a statistically significant but modest positive correlation between PLOD1 and ENO1 protein expression in our clinical cohort (Figure 8B). Furthermore, Kaplan–Meier survival analysis demonstrated that high expression of either PLOD1 or ENO1 was associated with significantly shorter overall survival (Figure 8C,D). To further strengthen the clinical relevance of the PLOD1‐ENO1 axis and address the limited size of our in‐house cohort, we performed independent validation using the TCGA‐GBMLGG and CGGA mRNAseq_325 datasets. In both cohorts, PLOD1 expression showed a strong positive correlation with ENO1 expression (TCGA: rho = 0.61, p < 0.001; CGGA: rho = 0.63, p < 0.001) (Figure 8G). Moreover, elevated expressions of either PLOD1 or ENO1 were consistently associated with significantly poorer overall survival across both datasets (Figure 8E,F), further supporting the prognostic significance of the PLOD1‐ENO1 axis in glioma.

Taken together, our findings support a cohesive molecular model for how GBM cells acquire chemoresistance through metabolic reprogramming (Figure 8H). In this model, upregulated PLOD1 acts as an important metabolic checkpoint. PLOD1 interacts with ENO1, and its catalytic activity promotes ENO1 stability. Crucially, this PLOD1‐dependent regulatory effect reduces binding of the E3 ubiquitin ligase FBXW7, thereby shielding ENO1 from ubiquitination and subsequent proteasomal degradation. The resulting accumulation of stable ENO1 protein enforces a hyper‐glycolytic state. This metabolic rewiring, in turn, fuels a cascade of malignant phenotypes, including enhanced proliferation, migration, and the critical development of resistance to TMZ therapy.

## 4 Discussion

In this study, we identify the lysyl hydroxylase PLOD1 as an important mediator of acquired TMZ resistance in GBM. We demonstrate that PLOD1 interacts with ENO1, and its catalytic activity promotes ENO1 stability, thereby shielding it from FBXW7‐mediated proteasomal degradation. This axis fuels a hyper‐glycolytic state essential for malignancy and chemoresistance. Our findings delineate a signaling cascade establishing the PLOD1‐FBXW7‐ENO1 axis as a pivotal regulator of the tumor's metabolic landscape and a candidate prognostic biomarker.

Our work significantly expands the functional repertoire of PLOD1. Although PLOD1 has traditionally been characterized as an ER‐resident collagen‐modifying enzyme linked primarily to extracellular matrix remodeling [59, 60], recent studies have expanded its functional scope. Notably, PLOD1 has been shown to stabilize the cytosolic cytoskeletal protein Septin2 [56], supporting the concept that PLOD1 can engage non‐collagenous substrates outside the classical ER‐collagen axis. Consistent with these observations, our subcellular fractionation analysis identified a detectable cytosolic pool of PLOD1, where it co‐localizes with ENO1. Building on these findings, we identify the glycolytic enzyme ENO1 as a previously unrecognized PLOD1‐interacting protein, providing a plausible mechanistic framework through which PLOD1 catalytic activity contributes to ENO1 stabilization and metabolic adaptation in GBM cells.

Mechanistically, we address a critical gap regarding ENO1 stability in GBM [32, 61]. While FBXW7 canonically targets metabolic enzymes for degradation [47, 62, 63, 64, 65], our findings suggest that PLOD1 catalytic activity protects ENO1 from FBXW7‐dependent ubiquitination by reducing FBXW7 recruitment or substrate recognition. Furthermore, through an integrated strategy combining LC‐MS/MS‐guided candidate prioritization, structural accessibility analysis, and site‐directed mutagenesis, we identified K60 as a functionally critical candidate residue required for PLOD1‐dependent ENO1 stabilization. Mutation of K60 restored FBXW7 association, increased ENO1 ubiquitination, and accelerated ENO1 turnover, further supporting a model in which PLOD1‐dependent modification of ENO1 reduces its susceptibility to FBXW7 recognition. Our domain mapping supports a “clamp‐and‐regulate” model, where the Ndst domain mediates binding, and the catalytic domain is required for subsequent ENO1 stabilization. This structural insight provides a rationale for designing inhibitors that disrupt the PLOD1‐ENO1 interface or block PLOD1 enzymatic activity to restore chemosensitivity.

By identifying the PLOD1‐ENO1 axis as a post‐translational enforcer of the Warburg effect, our study complements traditional transcriptional models involving HIF‐1α and MYC [66, 67, 68]. Although the upstream events responsible for PLOD1 induction in TMZ‐resistant GBM cells were not directly investigated in the present study, several plausible mechanisms may account for this observation. Chronic TMZ exposure is known to induce persistent DNA damage, oxidative stress, and adaptive hypoxia‐associated signaling, all of which can trigger extensive transcriptional reprogramming [69, 70]. Given that PLOD1 is a well‐established hypoxia‐responsive gene, it is conceivable that stress‐adaptive pathways contribute to its upregulation during the acquisition of TMZ resistance [71]. Future studies will be required to define the precise regulatory mechanisms governing PLOD1 expression in resistant GBM cells. Importantly, our findings support an emerging model in which metabolic reprogramming functions as a critical complementary layer to established TMZ‐resistance mechanisms. Canonical pathways, including MGMT‐mediated DNA repair and mismatch repair deficiency, primarily determine how tumor cells respond to TMZ‐induced DNA lesions [69]. In contrast, enhanced glycolytic activity provides the bioenergetic and biosynthetic resources required to sustain these adaptive responses. Increased glycolytic flux can support ATP production, facilitate nucleotide biosynthesis through interconnected metabolic pathways, and contribute to cellular redox homeostasis, thereby improving tolerance to TMZ‐induced stress. Within this framework, the PLOD1–FBXW7–ENO1 axis may act as a metabolic facilitator that cooperates with, rather than replaces, established resistance pathways. By reinforcing the metabolic fitness of GBM cells, this signaling axis creates a permissive environment for the execution of canonical resistance programs, ultimately promoting therapeutic failure. The ability of either glycolytic inhibition or PLOD1 depletion to restore TMZ sensitivity further highlights the therapeutic vulnerability of metabolically adapted GBM cells and supports the rationale for combining TMZ with metabolism‐targeted interventions [72].

The clinical and therapeutic implications of our findings are substantial. In our cohort, higher PLOD1 and ENO1 expressions were associated with poorer survival. Preclinically, targeting the PLOD1‐ENO1 axis in xenografts not only suppressed tumor growth but achieved profound re‐sensitization to TMZ. The observed downregulation of Ki67 and ENO1 in treated tumors offers candidate pharmacodynamic biomarkers for future clinical trials. These results validate the “metabolic‐proteostasis” paradigm as a viable therapeutic strategy, moving beyond purely genetic determinants like MGMT [13, 73].

From a translational perspective, identifying the PLOD1‐ENO1 axis uncovers a potentially actionable vulnerability in chemoresistant GBM. The requirement of PLOD1 enzymatic activity for ENO1 stabilization suggests that pharmacological inhibition of PLOD1 hydroxylase activity or disruption of the PLOD1‐ENO1 regulatory interface may represent promising strategies to dismantle this metabolic dependency. Although selective and clinically approved PLOD1 inhibitors are currently unavailable, the development of PLOD1‐targeted small molecules represents an attractive direction for future therapeutic exploration. Furthermore, our findings provide a rationale for combining PLOD1‐targeted interventions with TMZ‐based therapy. Because enhanced glycolytic capacity supports the energetic, biosynthetic, and redox requirements associated with TMZ resistance, inhibition of the PLOD1‐ENO1 axis may reduce the metabolic adaptability of GBM cells and enhance their vulnerability to TMZ‐induced stress. Future studies using patient‐derived orthotopic xenografts and clinically relevant pharmacological approaches will be required to evaluate the therapeutic efficacy, pharmacokinetic properties, and blood–brain barrier penetrance of such combination strategies.

We acknowledge certain limitations. First, although the correlation between PLOD1 and ENO1 protein expression in our in‐house IHC cohort was statistically significant but modest, this finding should be interpreted in the context of the relatively limited sample size and the inherent variability associated with semiquantitative immunohistochemical scoring. Importantly, analyses of multiple independent glioma cohorts (TCGA‐GBMLGG, CGGA mRNAseq_693, and CGGA mRNAseq_325) consistently demonstrated robust positive correlations between PLOD1 and ENO1 expression together with significant prognostic associations. Notably, adjusted PLOD1‐ENO1 correlations remained significant across all three public cohorts and persisted within individual WHO grade strata (Figure S9A–C). In addition, a multivariable Cox model in TCGA‐GBMLGG supported the prognostic association of the combined PLOD1–ENO1 signature (Figure S9D). These findings provide additional support for the clinical relevance of the PLOD1‐ENO1 axis in glioma progression. Second, although LC‐MS/MS analysis successfully recovered ENO1 peptides, we were unable to confidently identify a site‐specific hydroxylation event. The limited sensitivity of discovery‐based proteomics for low‐abundance modifications may have contributed to this result. To address this limitation, we integrated proteomic coverage, motif analysis, structural accessibility, and mutational validation to prioritize candidate residues. Notably, K60R exhibited the strongest effects on ENO1 stability, FBXW7 recruitment, and ubiquitination, suggesting that K60 represents a key functional residue within this regulatory mechanism. Nevertheless, whether K60 constitutes the direct hydroxylation site remains to be definitively established and will require future validation using targeted proteomic approaches.

Furthermore, while our study establishes the PLOD1‐FBXW7‐ENO1 axis as a primary driver of glycolytic reprogramming, we recognize that PLOD1 may exert broader effects on cellular homeostasis. Consistent with its reported roles in hypoxia and redox regulation, our supplemental data demonstrate that PLOD1 depletion triggers substantial intracellular ROS accumulation and mitochondrial depolarization (Figure S7). These findings suggest that the metabolic advantages conferred by PLOD1 extend beyond the Warburg effect to include the maintenance of redox balance and mitochondrial integrity. While the precise mechanistic links between PLOD1‐mediated ENO1 stabilization and these mitochondrial/redox phenotypes remain to be fully elucidated in the current study, they highlight the multifaceted role of PLOD1 in GBM survival and represent an important direction for future research.

Our findings rely primarily on established GBM cell lines and immunodeficient mouse models, which limit the assessment of tumor–immune interactions and the contribution of endogenous stromal components to PLOD1‐mediated metabolic adaptation. Although we further validated the oncogenic role of the PLOD1‐ENO1 axis using an orthotopic intracranial GBM model, this system still does not fully recapitulate the complexity of the human GBM microenvironment, including interactions between tumor cells, immune cells, and neighboring stromal populations. Future studies employing patient‐derived organoids, humanized models, and immunocompetent systems will be required to further define how PLOD1‐driven metabolic remodeling integrates with the broader tumor ecosystem. In conclusion, we position the PLOD1‐FBXW7‐ENO1 axis as a critical driver to therapeutic failure in GBM, offering a novel, druggable target to overcome chemoresistance.

## Author Contributions

F.X. contributed to conceptualization, methodology, investigation, data curation, formal analysis, visualization, and writing of the original draft. D.L. contributed to methodology, investigation, validation, data analysis, and visualization. X.C. contributed to investigation, validation, bioinformatics analysis, and data interpretation. F.F. contributed to conceptualization, supervision, project administration, resources, and critical revision of the manuscript. X.H. contributed to conceptualization, supervision, project administration, funding acquisition, and critical revision of the manuscript. All authors reviewed and approved the final manuscript.

## Conflicts of Interest

The authors declare no conflicts of interests.

## Ethics Approvals

All animal experiments were performed in accordance with the protocols authorized by the Committee on the Ethics of Animal Experiments of Fudan University Shanghai Cancer Center (approval No. FUSCC‐IACUC‐S2025‐0236). The use of human glioma specimens and adjacent normal brain tissues was reviewed and approved by the Medical Ethics Committee of Fudan University Shanghai Cancer Center (approval No. SZRYS2025257 and No. 1612167‐18). Written informed consent was obtained from all participants.

## Supporting information




**Supporting File 1**: advs78070‐sup‐0001‐SuppMat.pdf.

**Supporting File 2**: advs78070‐sup‐0002‐Figures.docx.

**Supporting File 3**: advs78070‐sup‐0003‐SuppMat.docx.

**Supporting File 4**: advs78070‐sup‐0004‐Tables.docx.

**Supporting File 5**: advs78070‐sup‐0005‐SuppMat.pdf.

## Data Availability

The datasets used and analyzed during the current study are available from the corresponding author on reasonable request. The original Western blot data are provided in Uncropped Western blots. The original microscope images are provided in original microscope images.
