# Supplementary material for: PLOD1 Catalytic Activity Stabilizes ENO1 by Limiting FBXW7‐dependent Degradation to Promote Glycolysis and TMZ Resistance in Glioblastoma
Source: Adv Sci (Weinh). 2026 Sep 27:e78070. Online ahead of print. doi: 10.1002/advs.78070 (PMC13616334; doi:10.1002/advs.78070)
Supplement: Supplementary file 1 [file ADVS-9999-e78070-s002.pdf]

FIG.1E

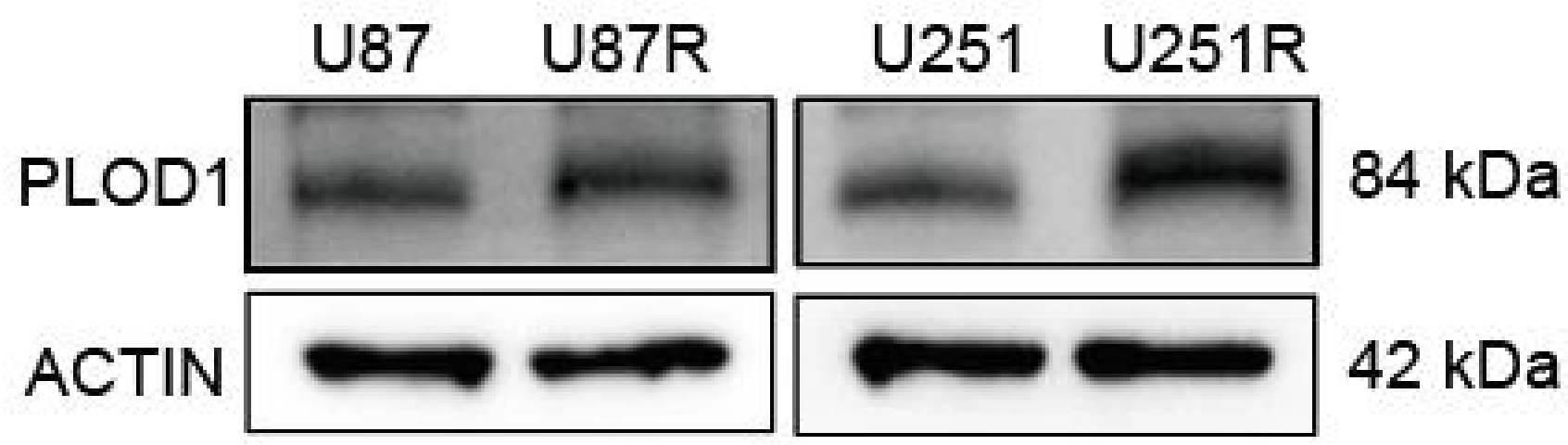

U87/U87R

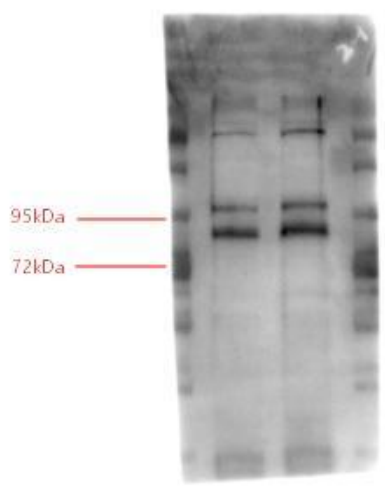

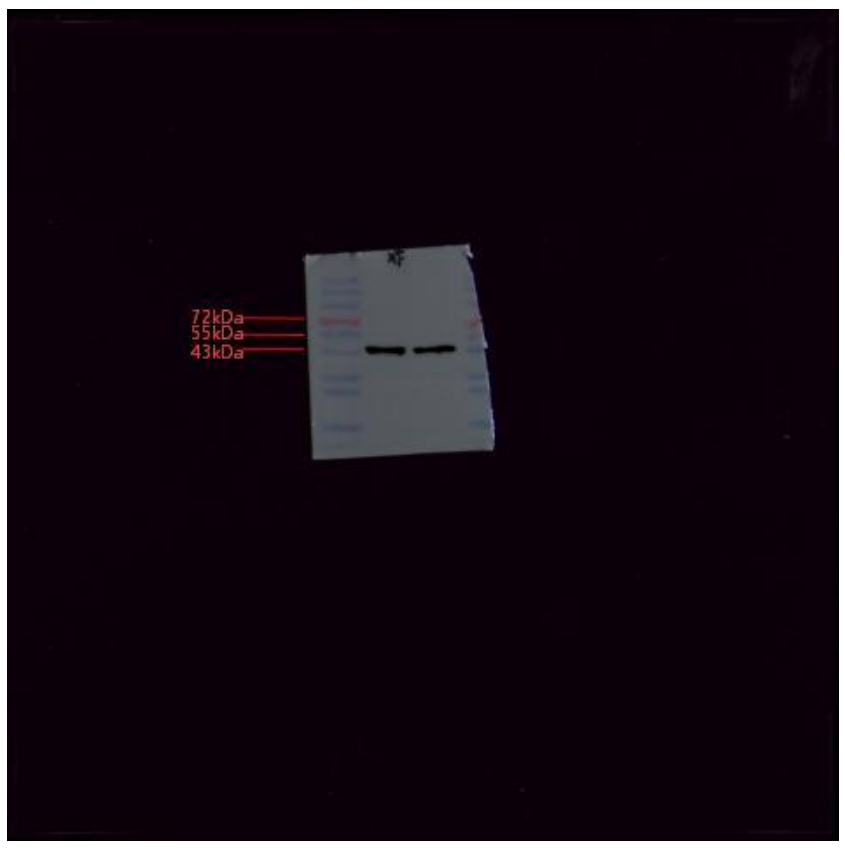

PLOD1

ACTIN

U251/U251R

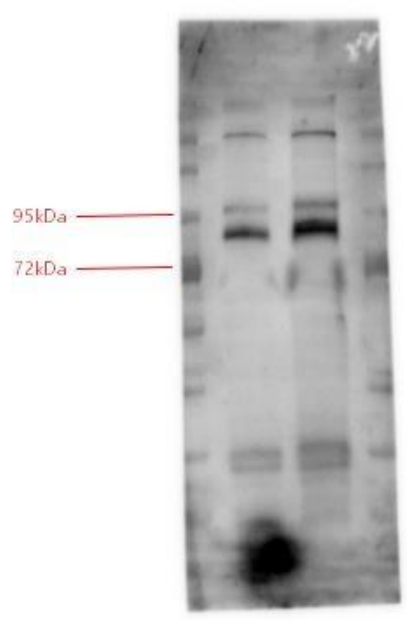

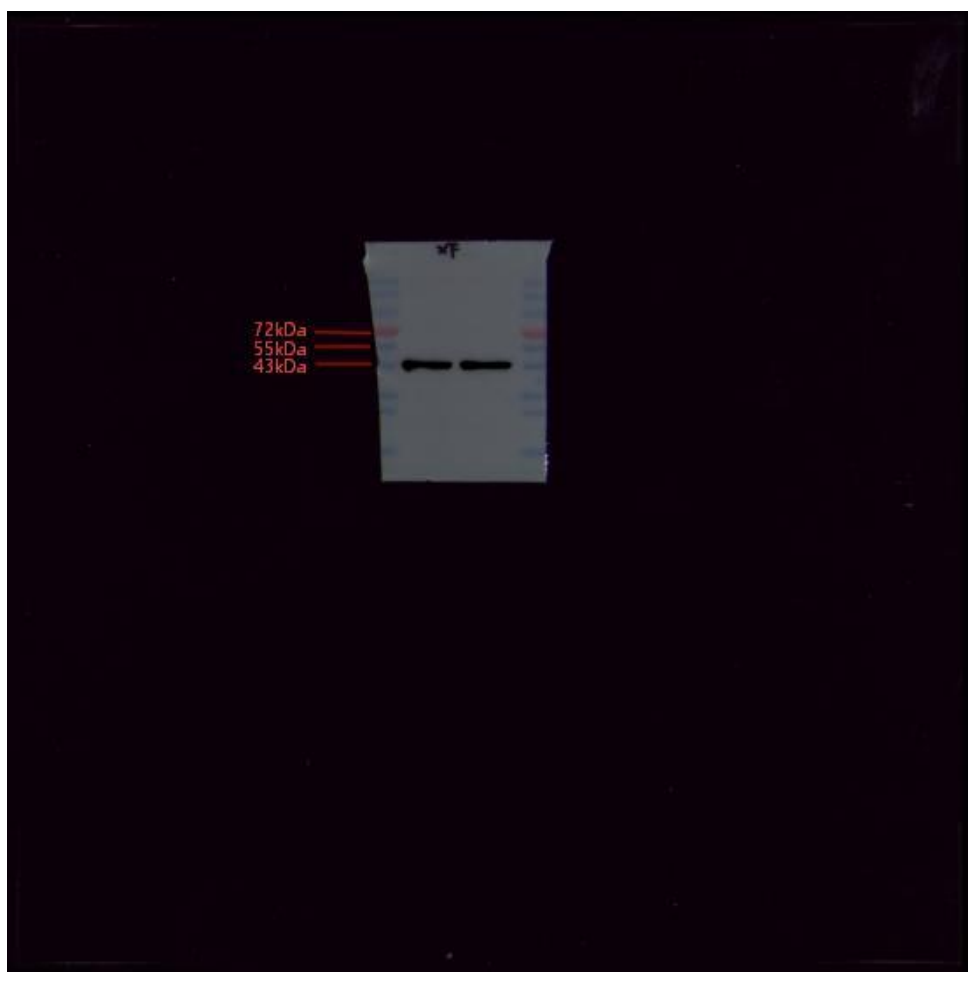

PLOD1

ACTIN

FIG.1G

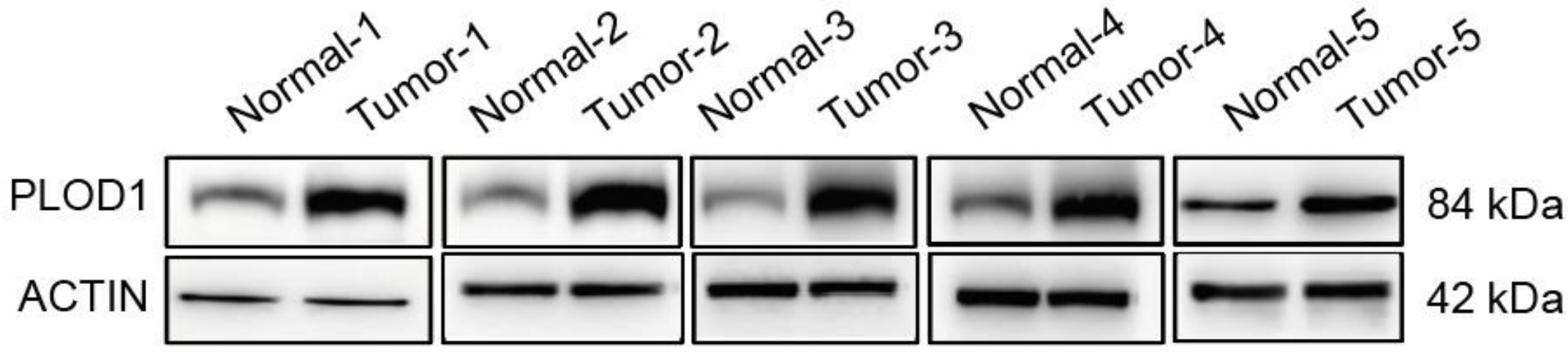

PLOD1 1 2 3

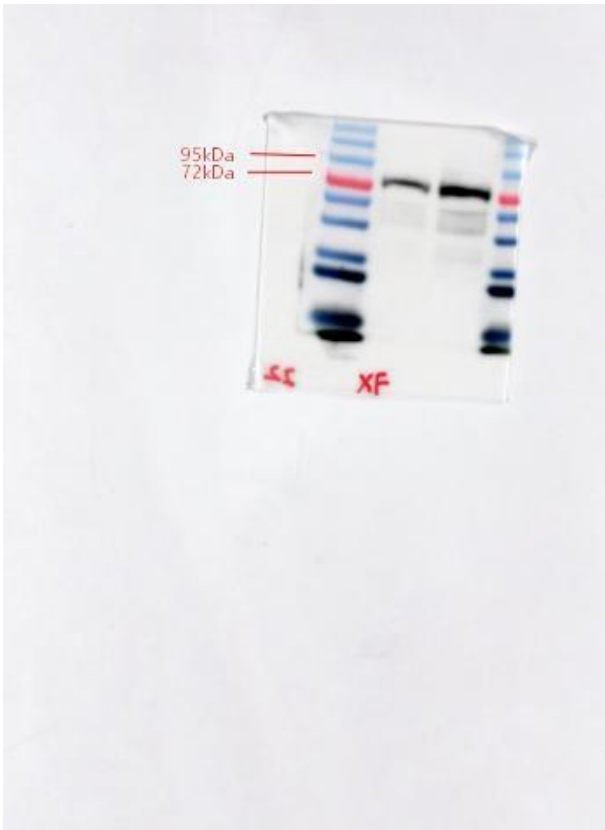

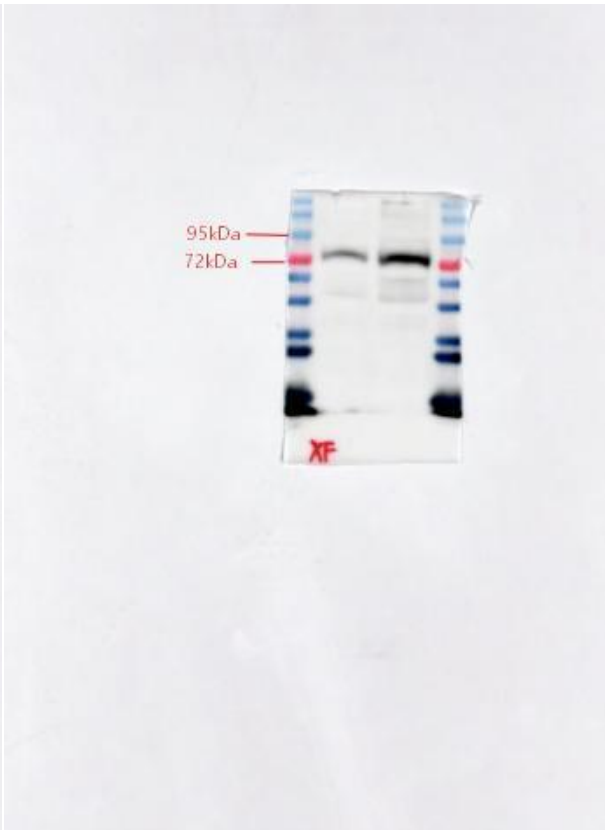

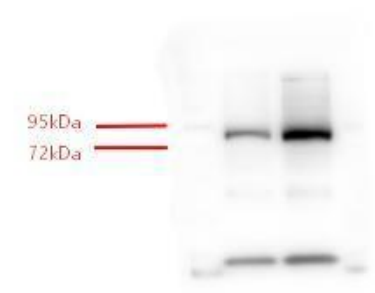

ACTIN 1 2

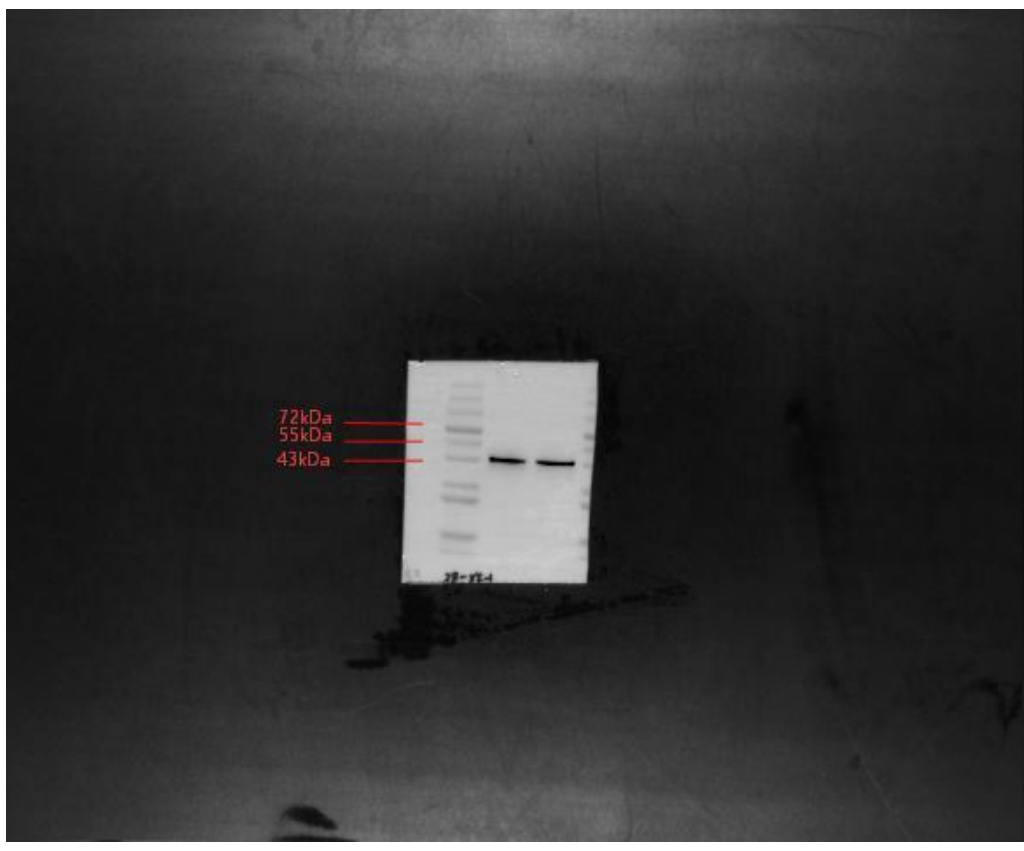

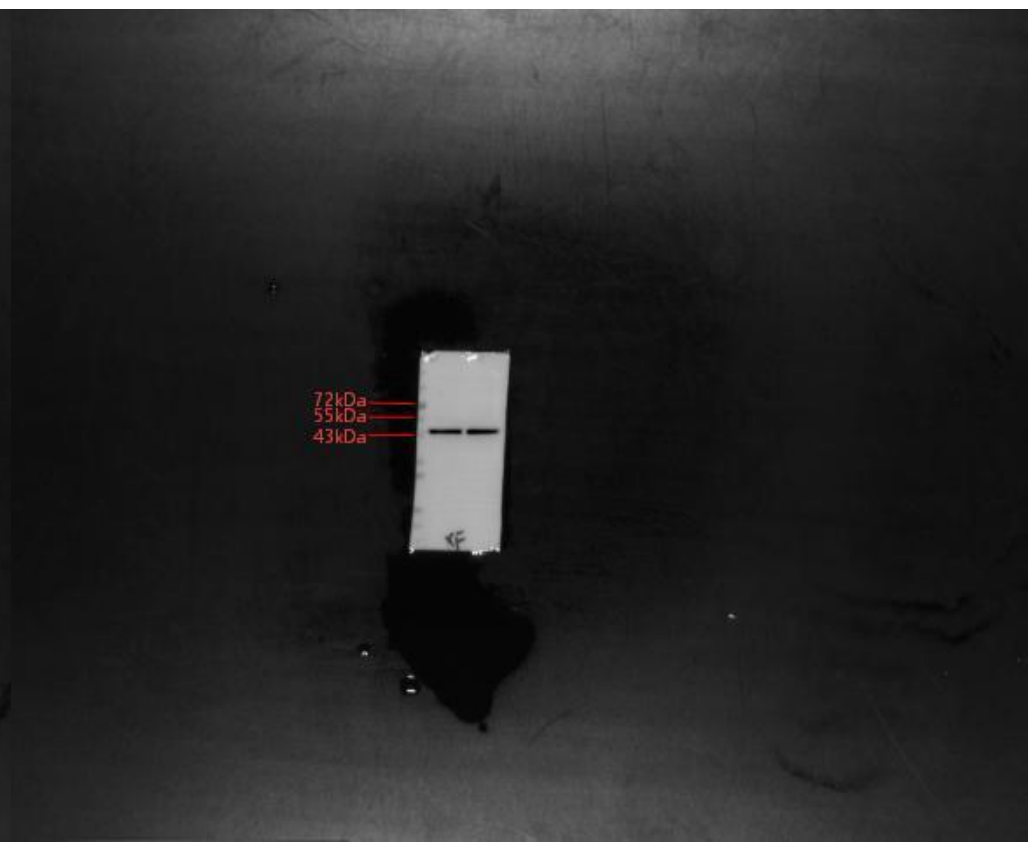

3

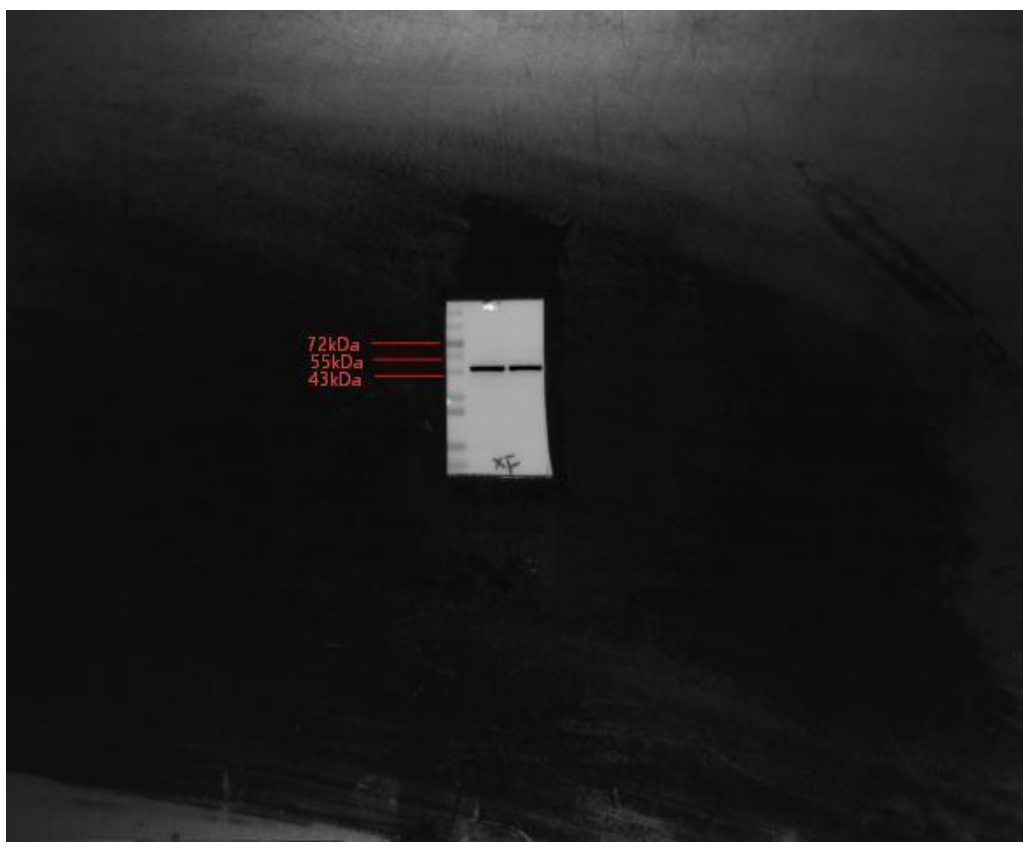

PLOD1 4

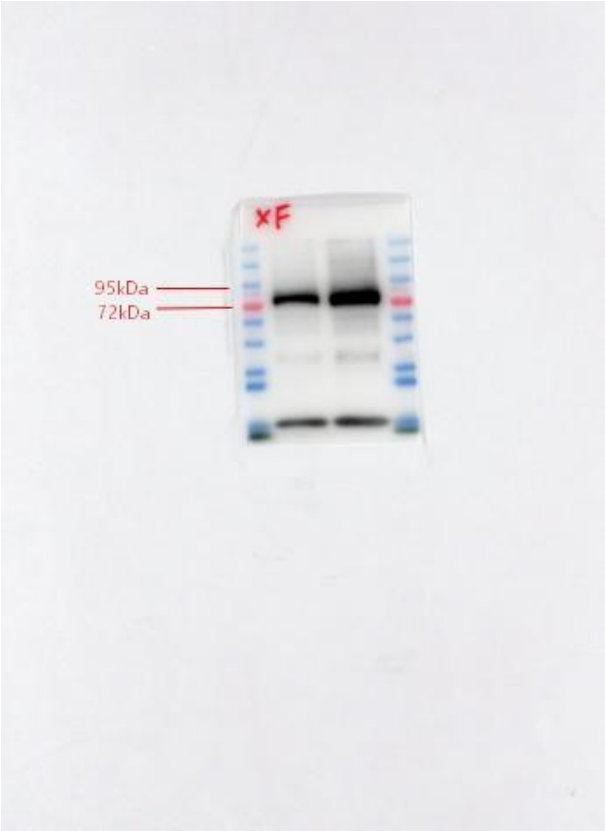

5

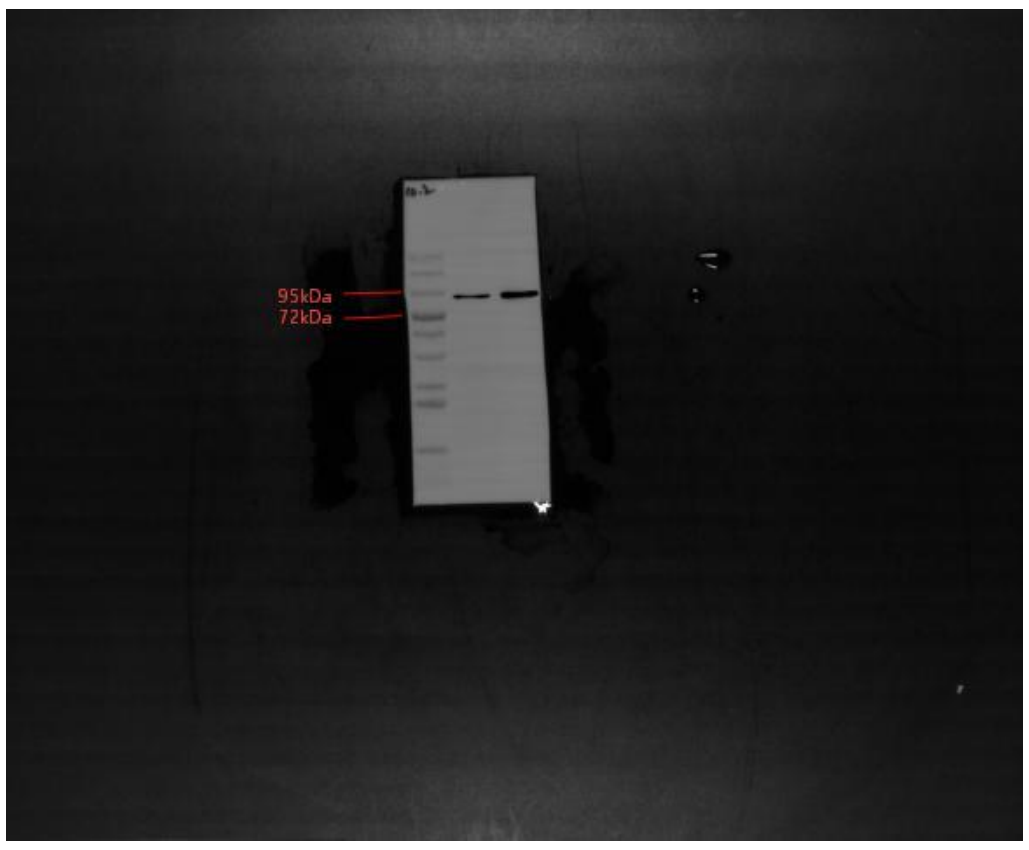

ACTIN 4

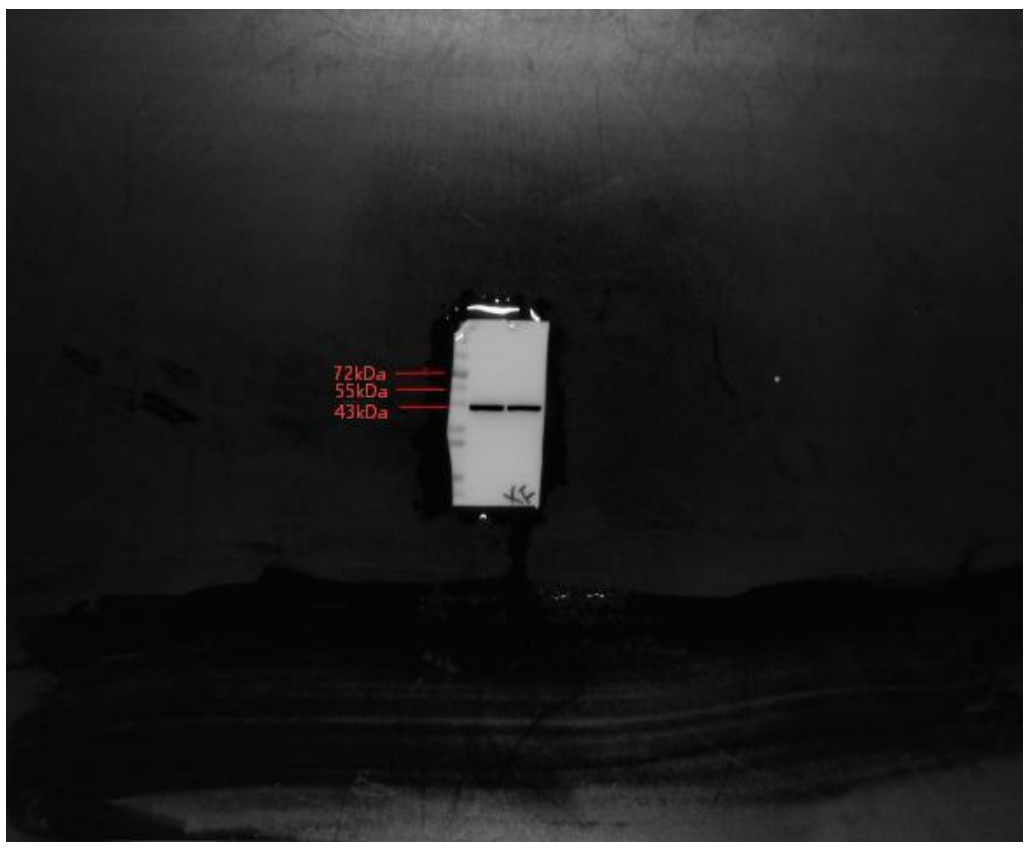

5

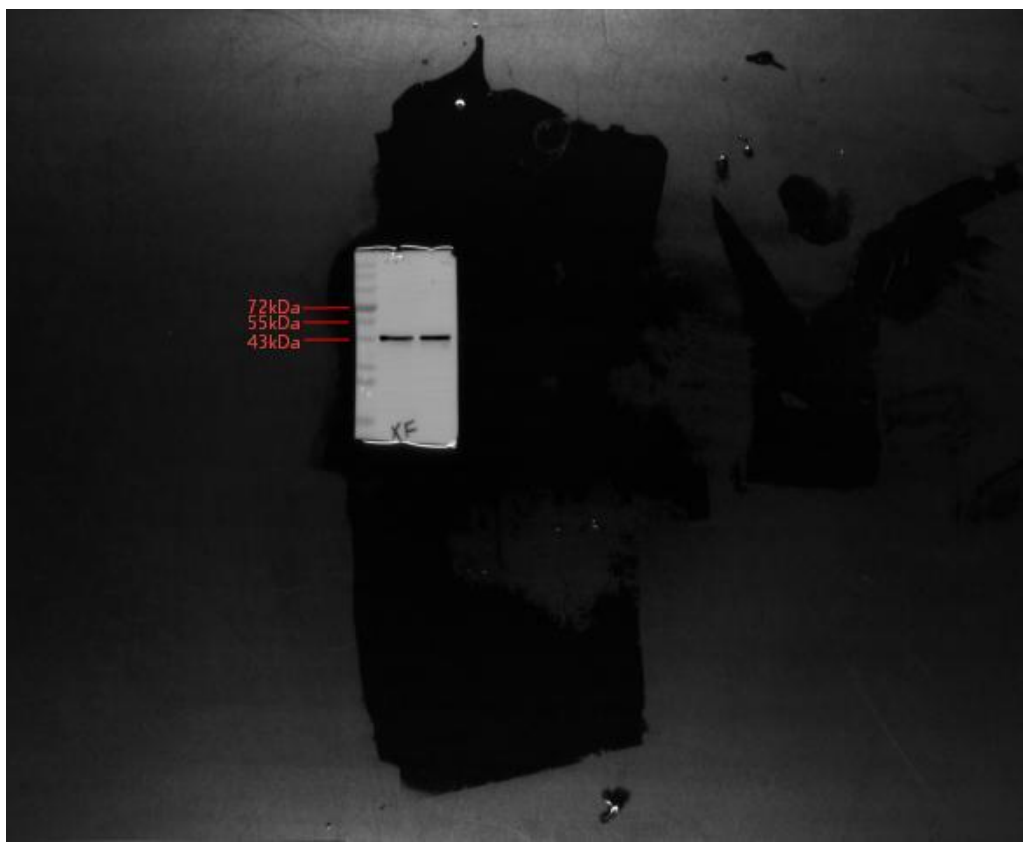

FIG.2F

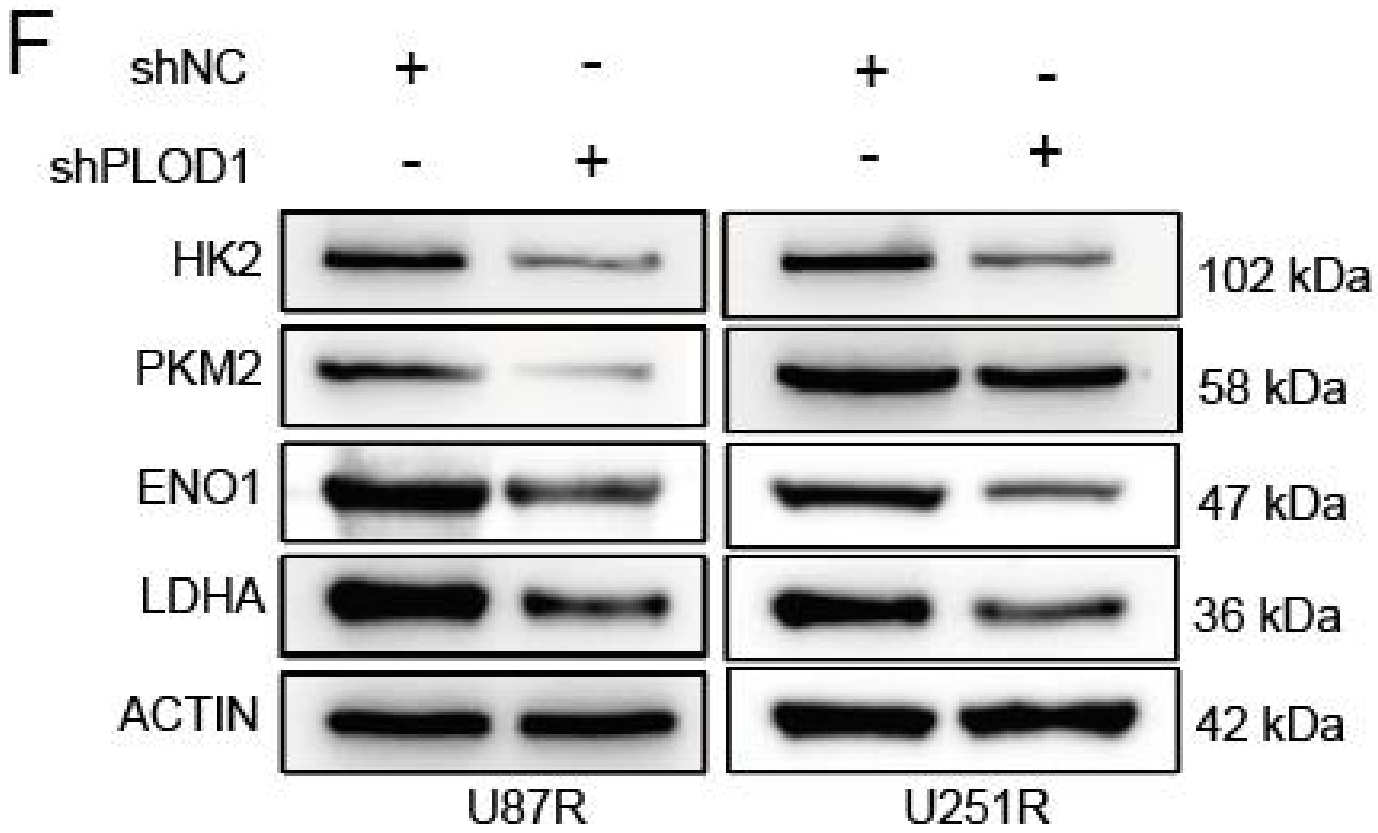

U87R

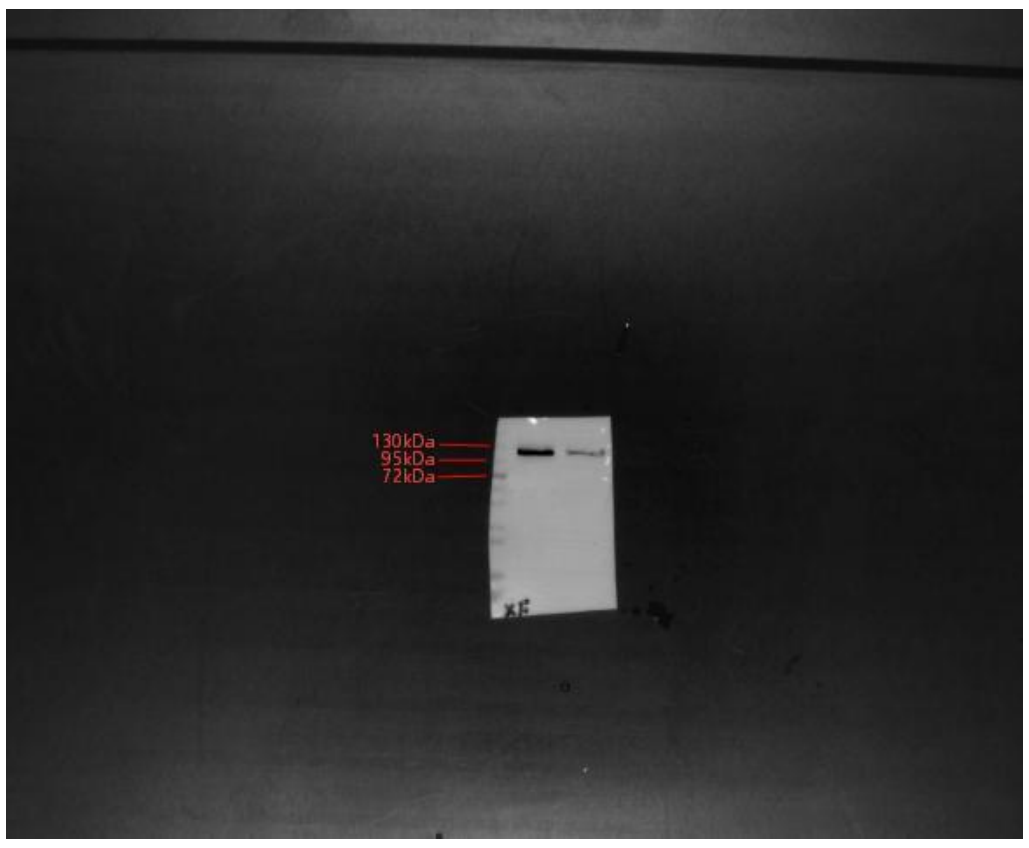

HK2

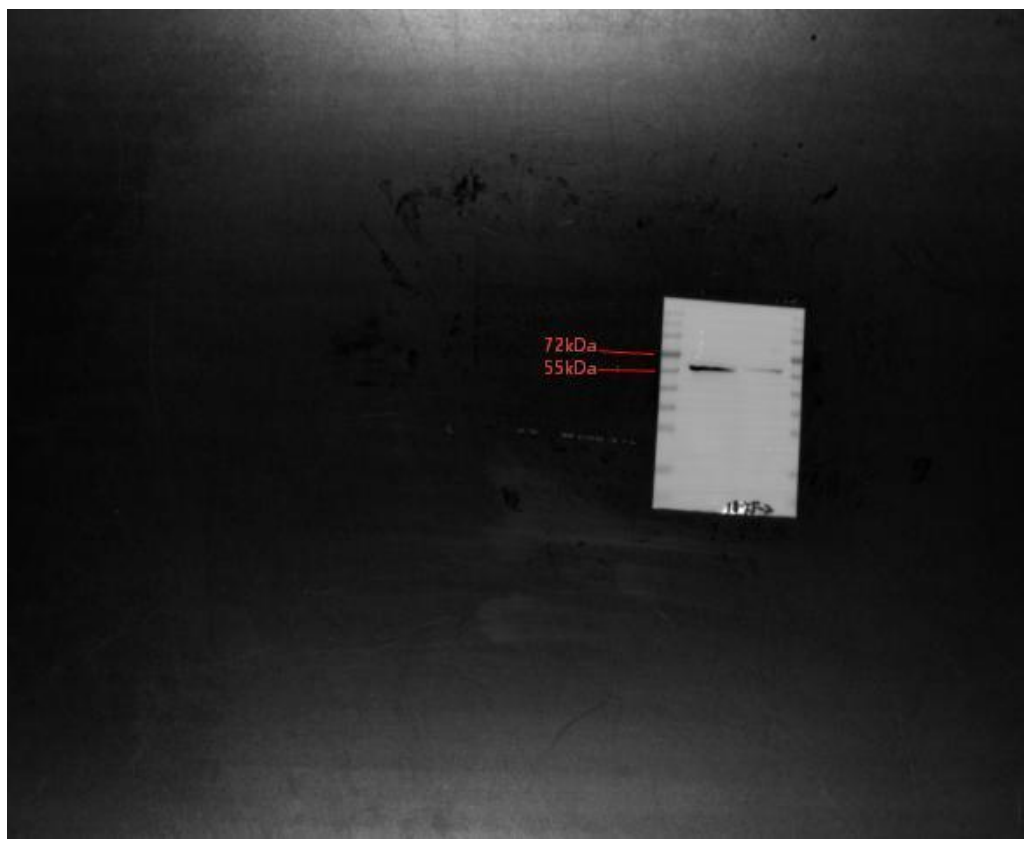

PKM2

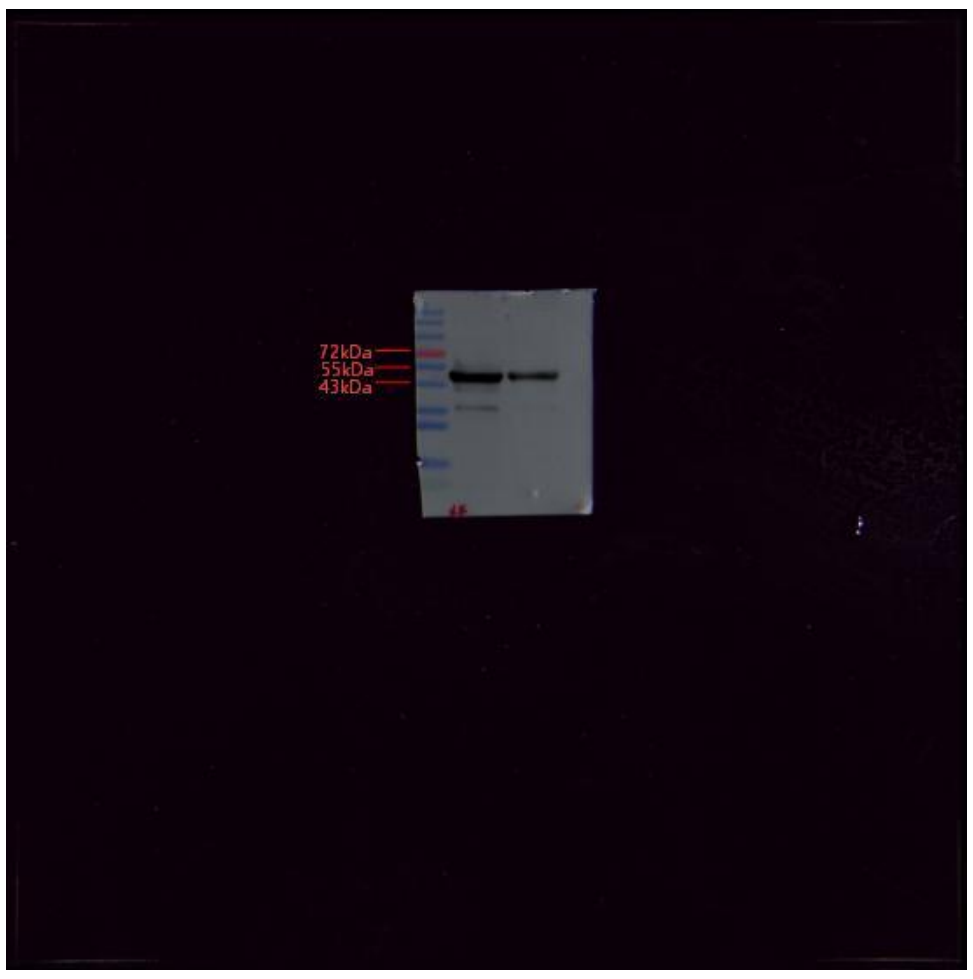

ENO1

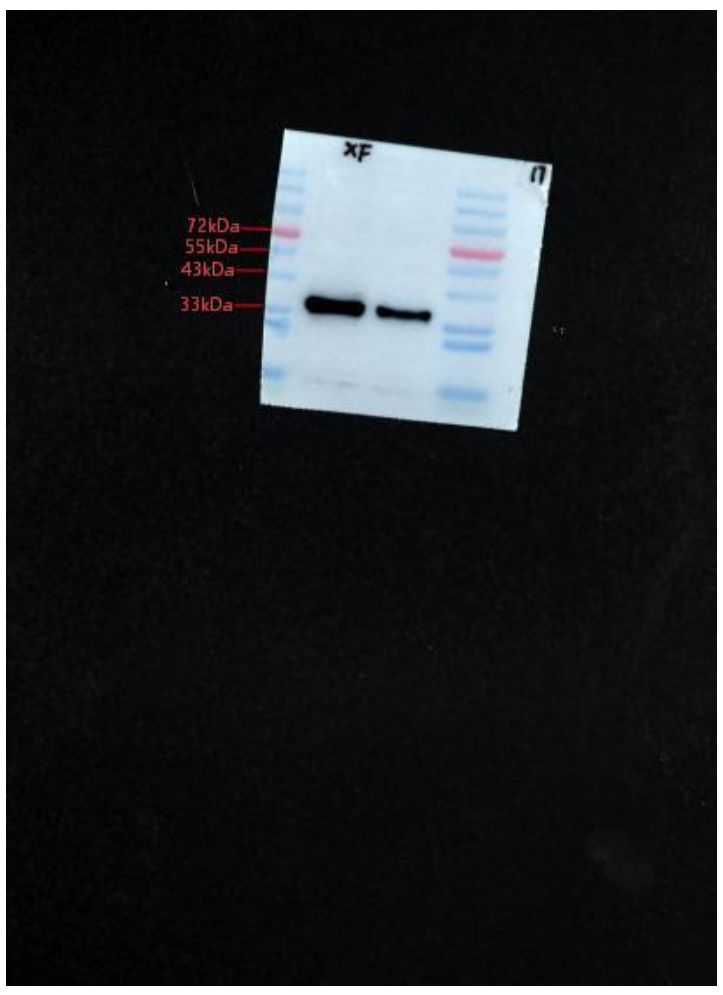

LDHA

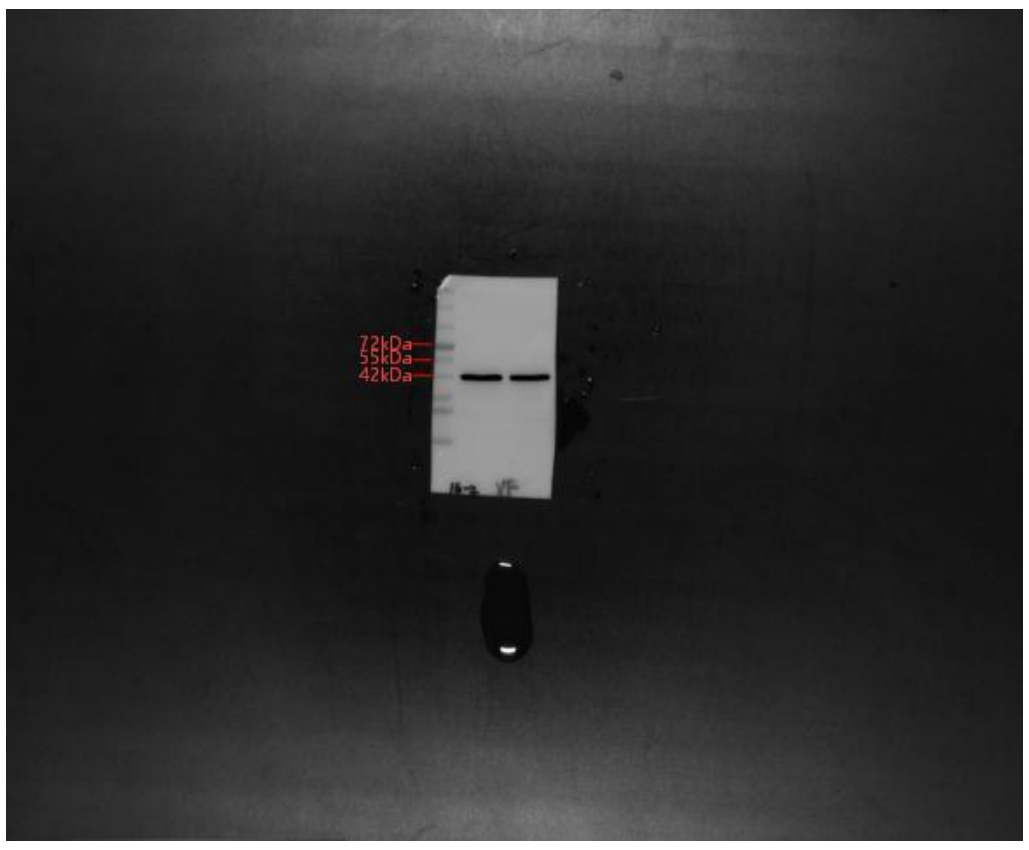

ACTIN

U251R

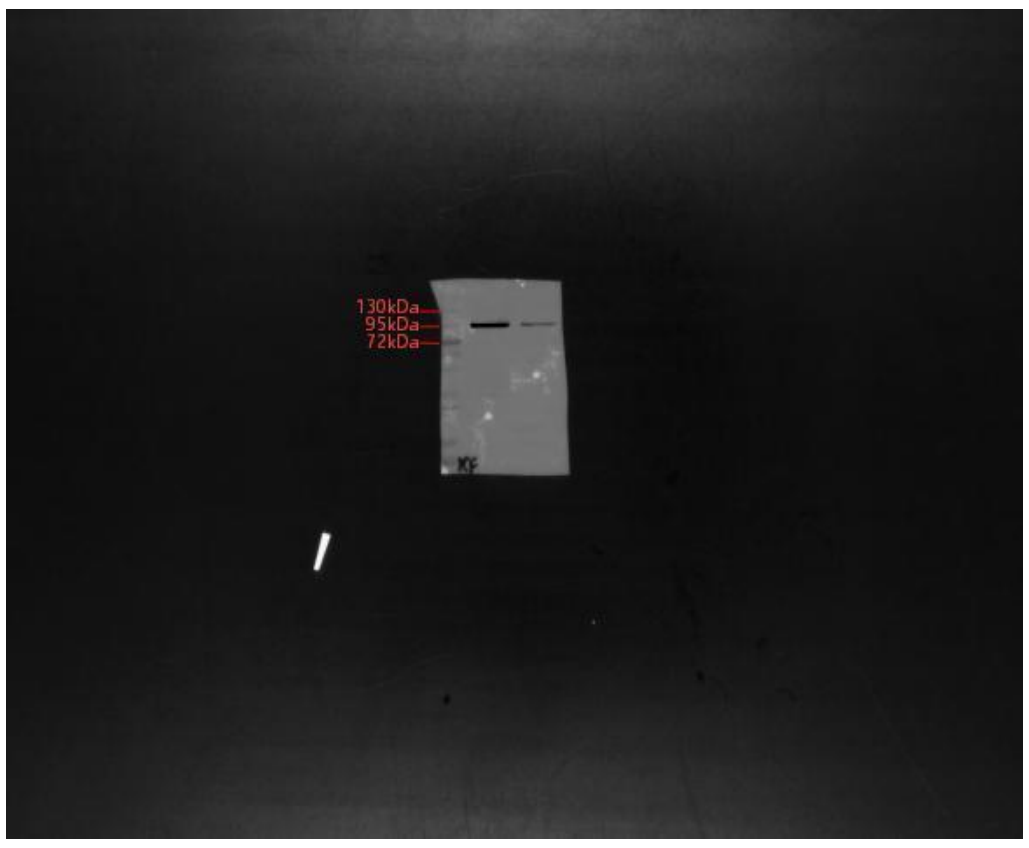

HK2

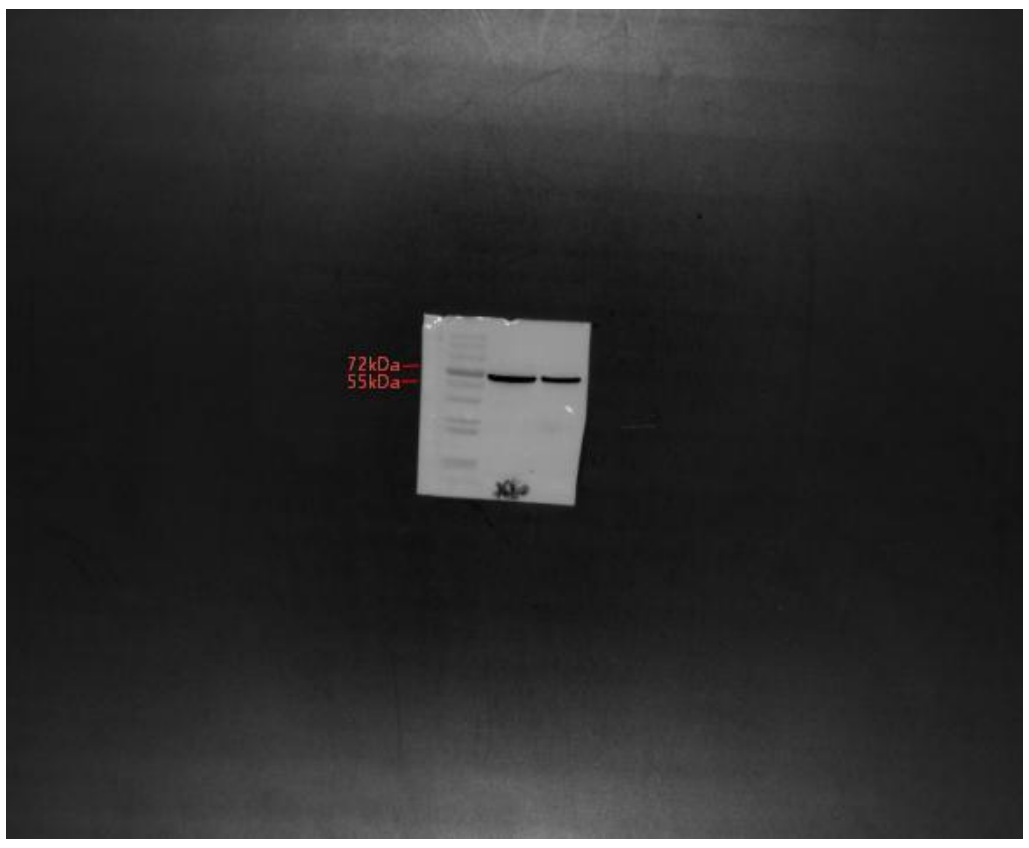

PKM2

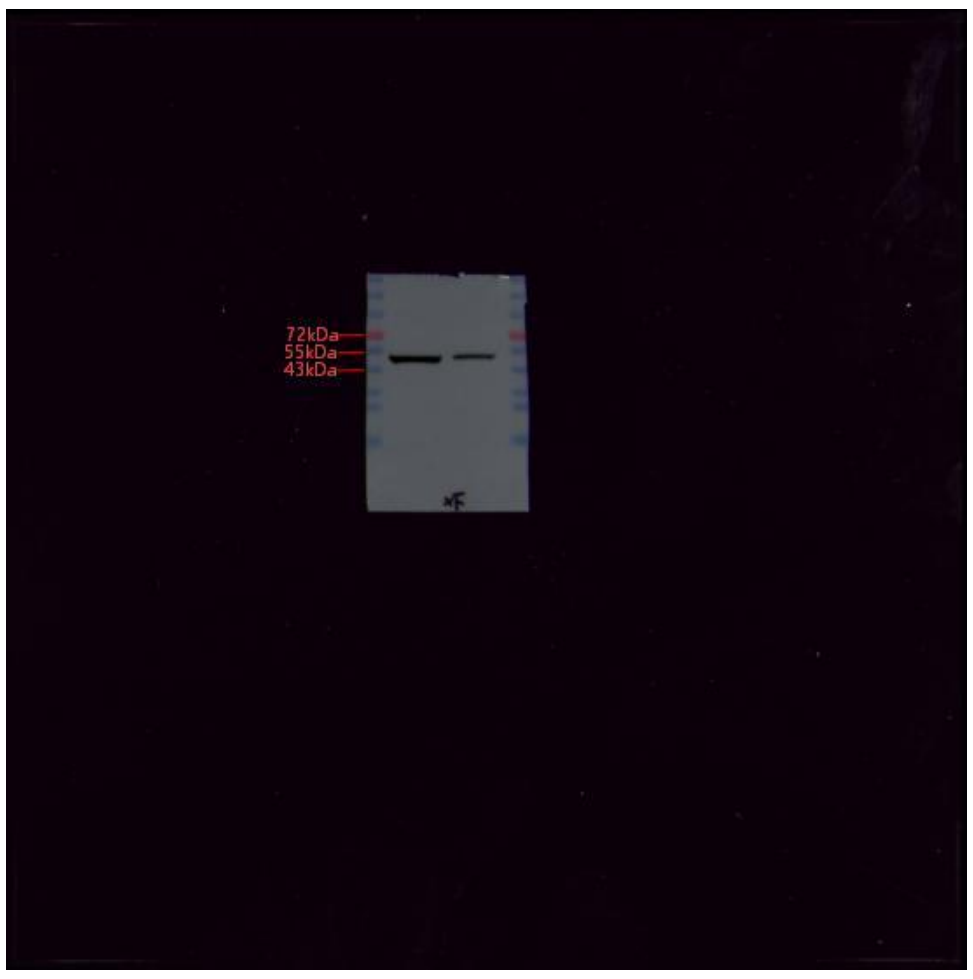

ENO1

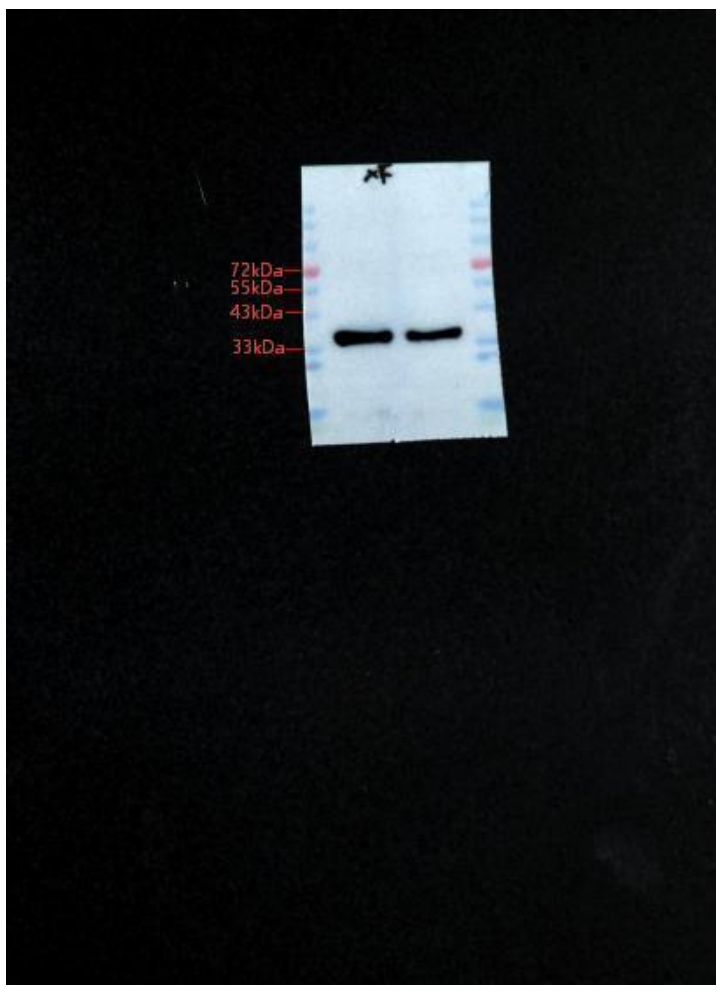

LDHA

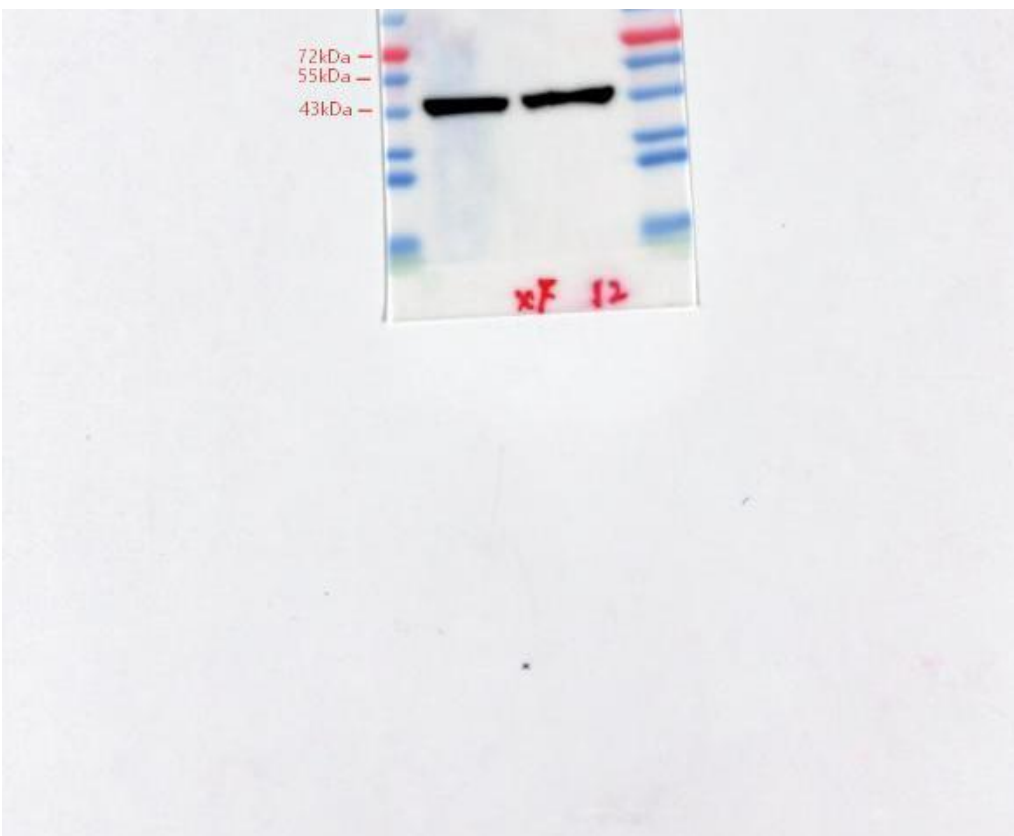

ACTIN

FIG.3B--PLOD1 IP ENO1

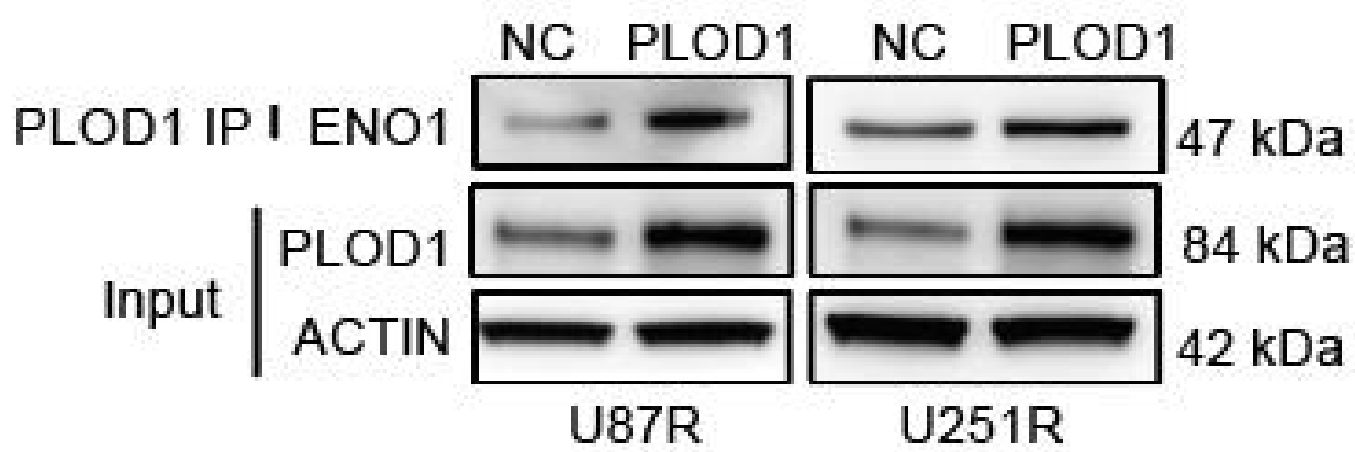

U87R

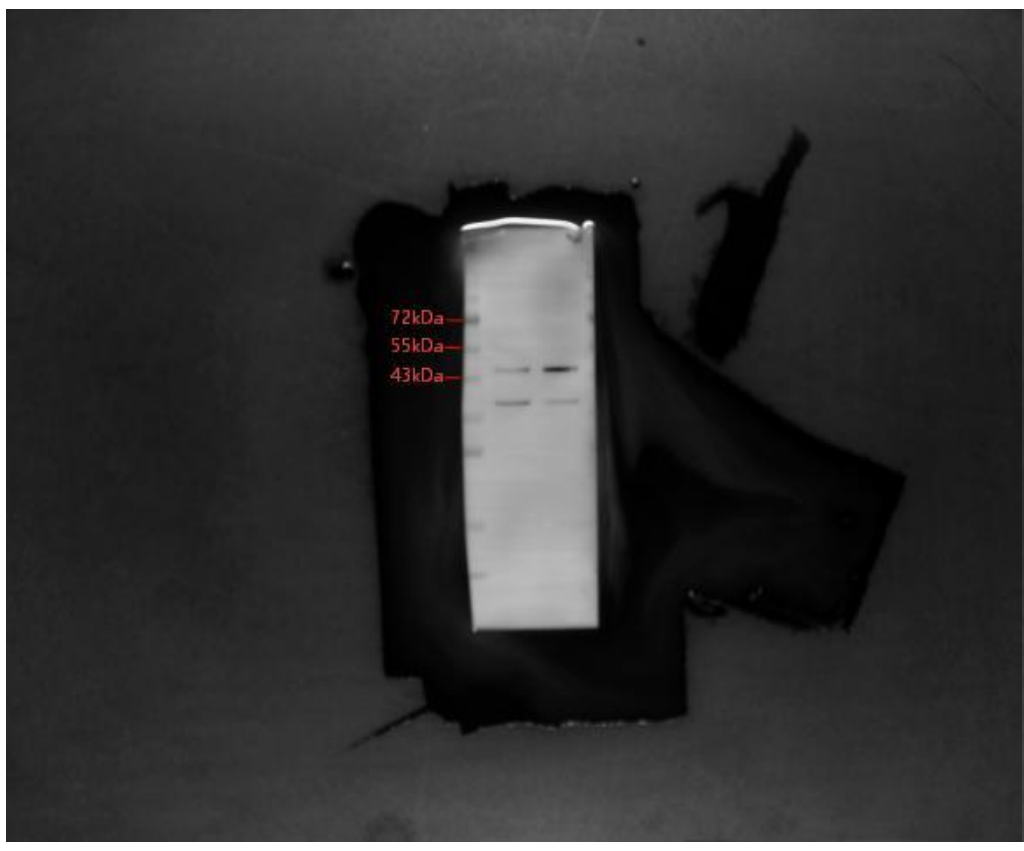

ENO1

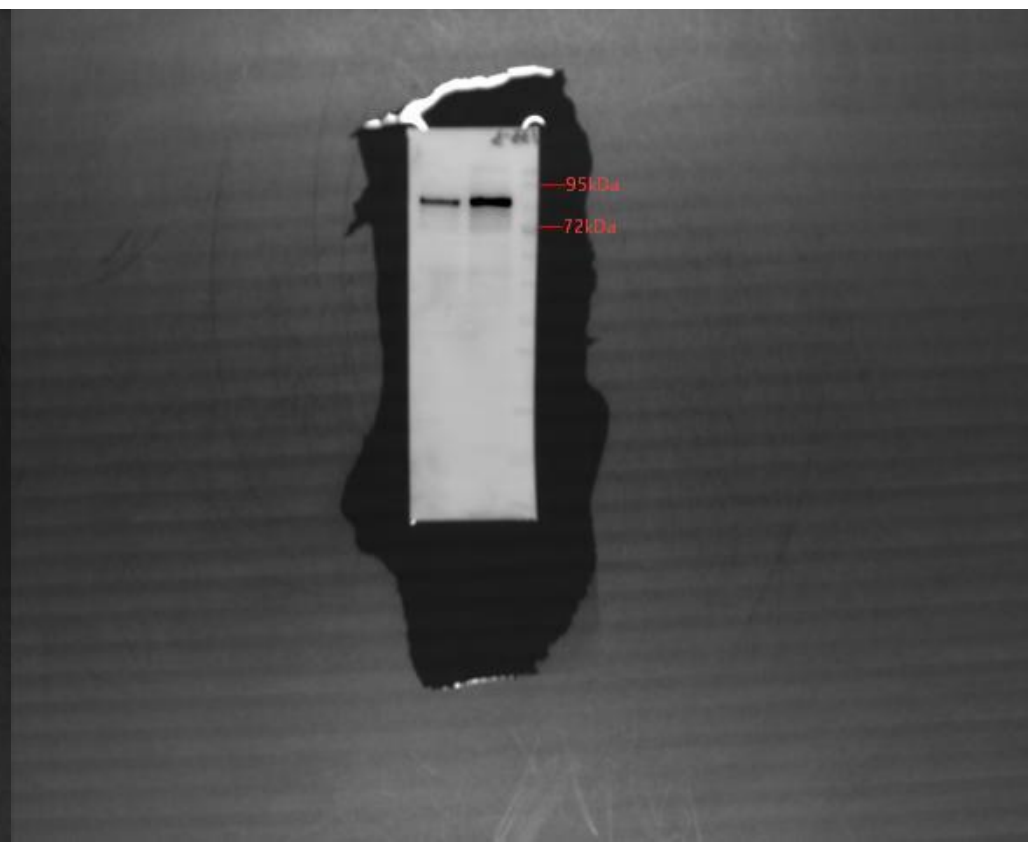

PLOD1

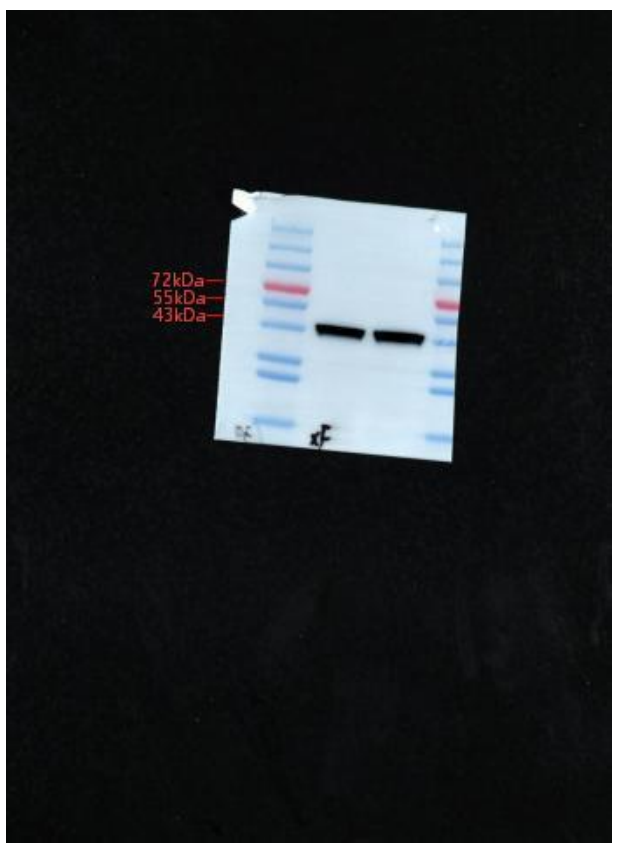

ACTIN

U251R

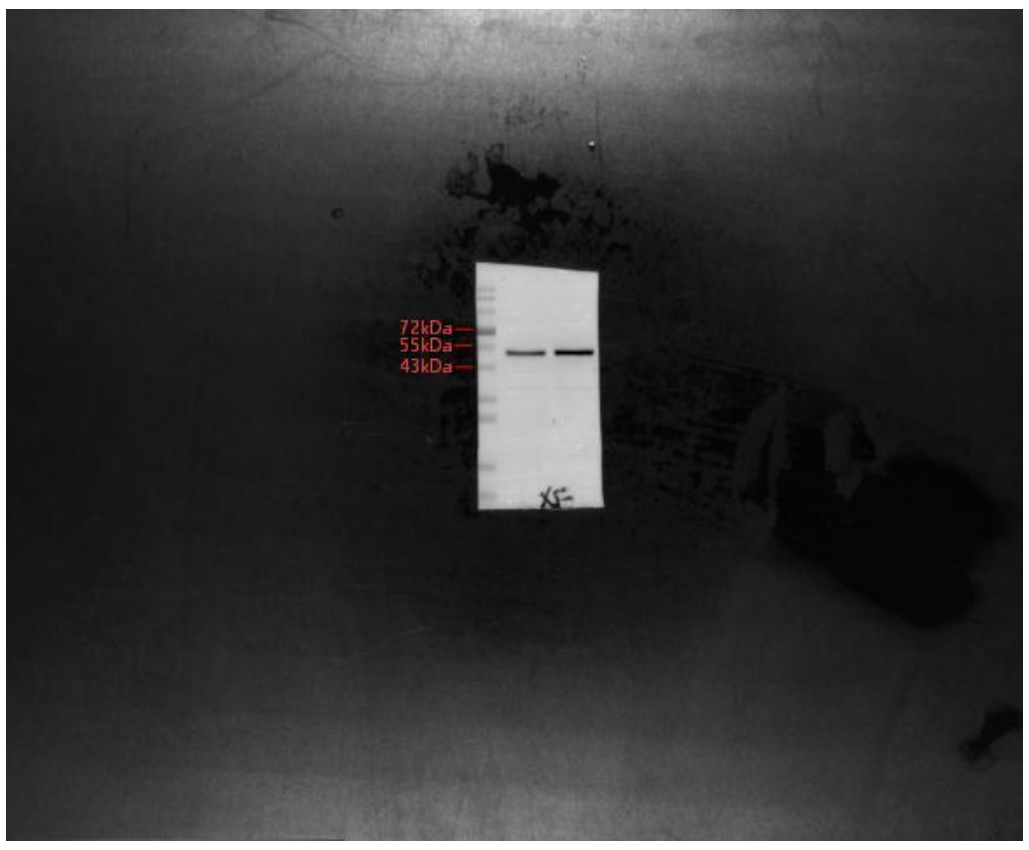

ENO1

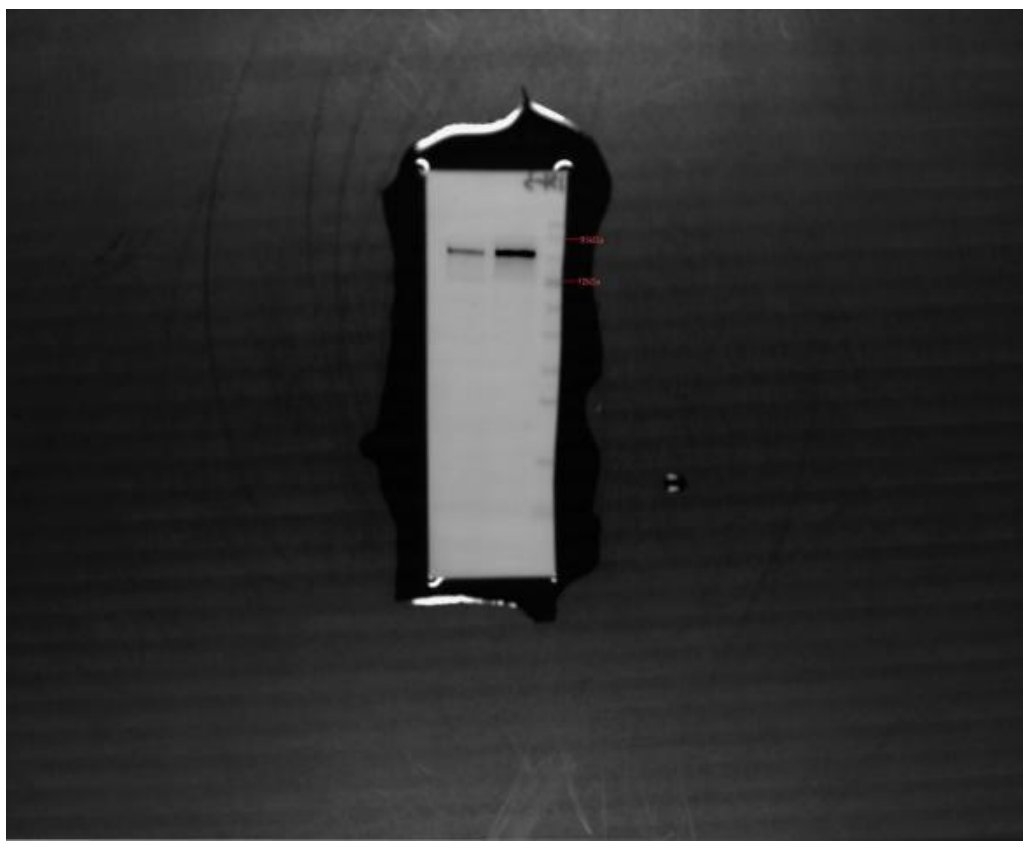

PLOD1

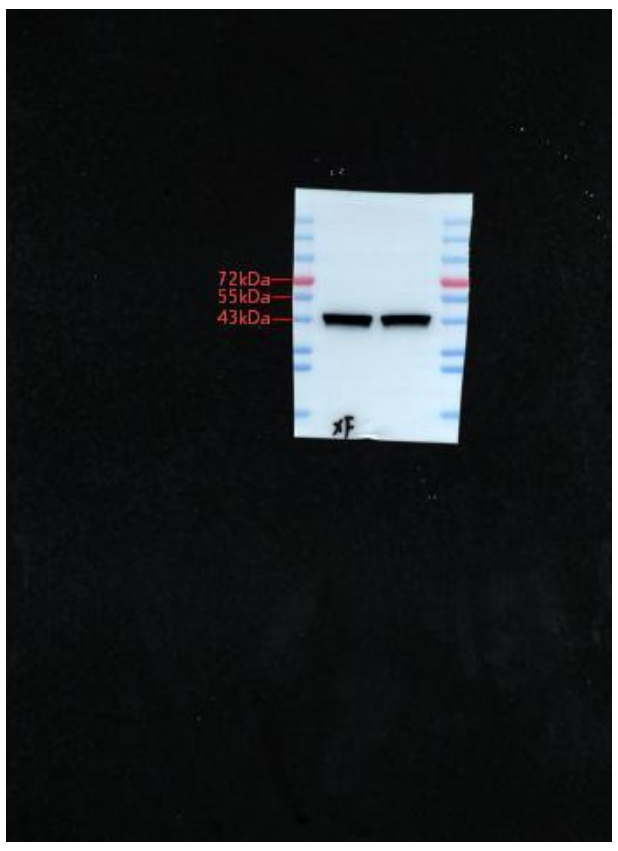

ACTIN

FIG.3B -- ENO1 IP PLOD1

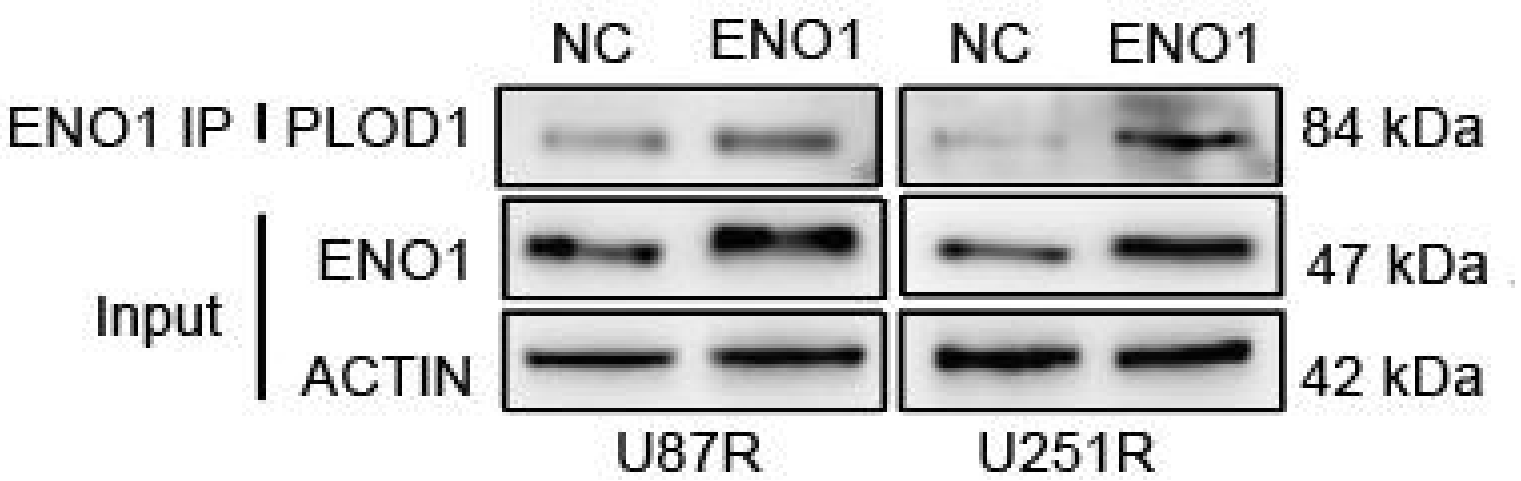

U87R

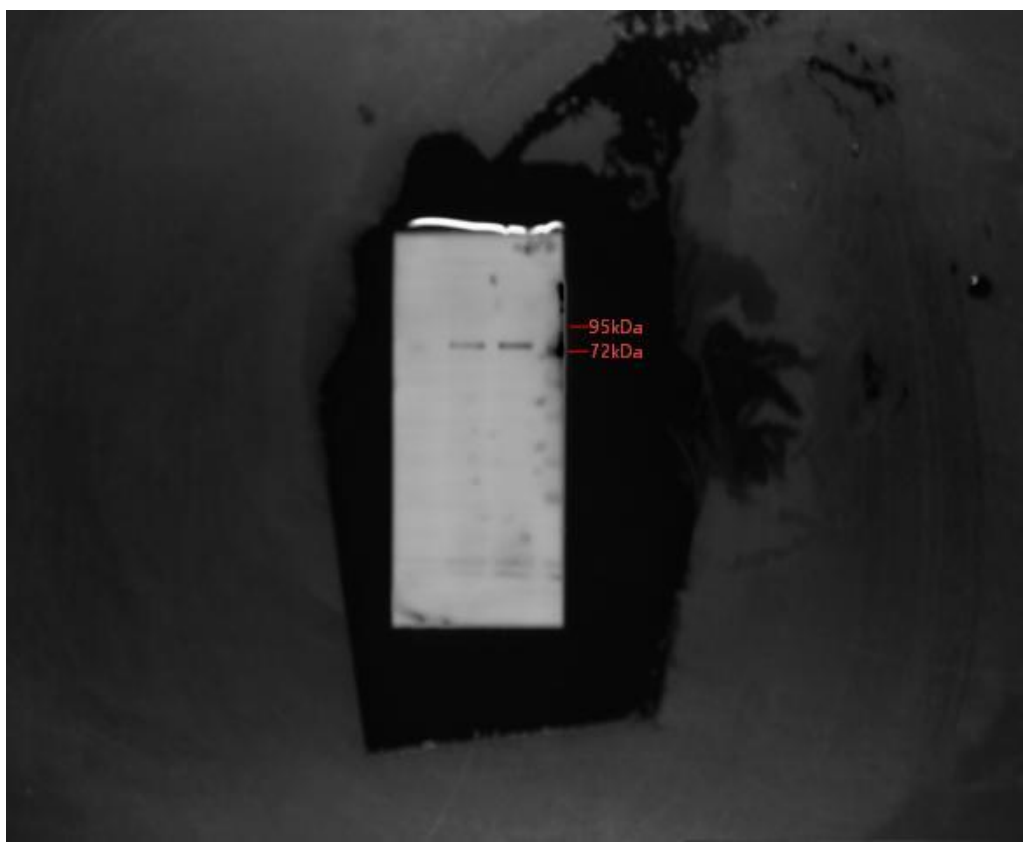

PLOD1

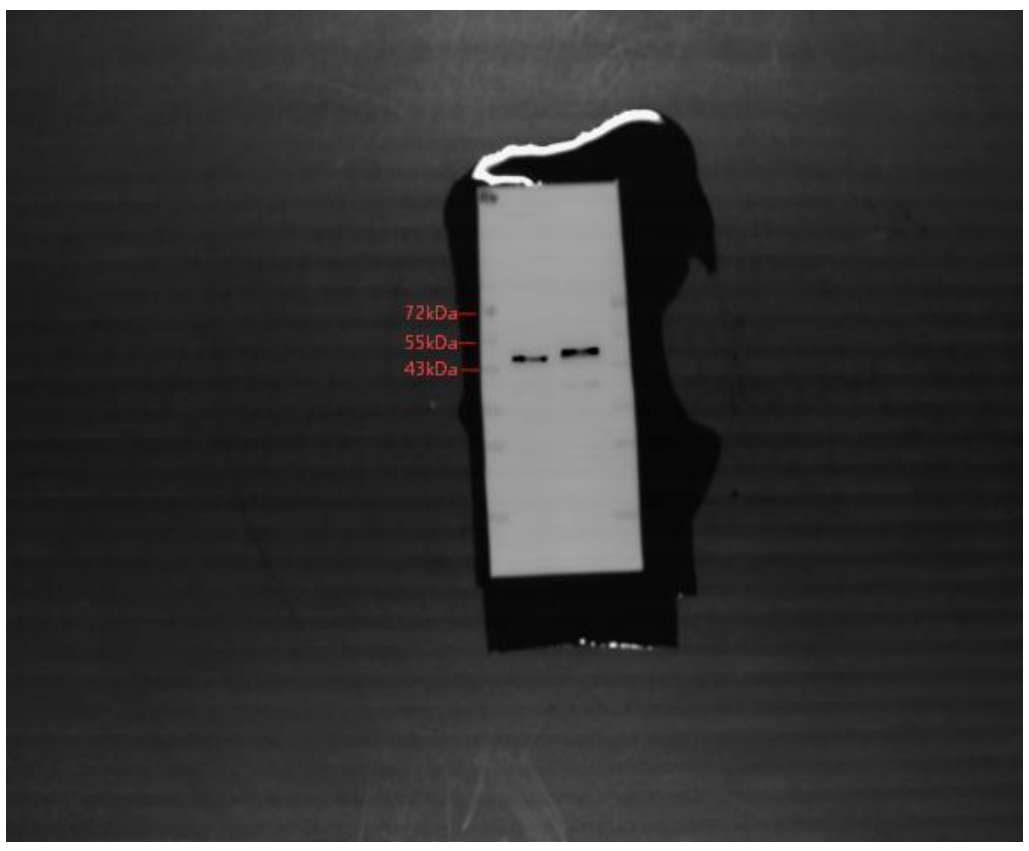

ENO1

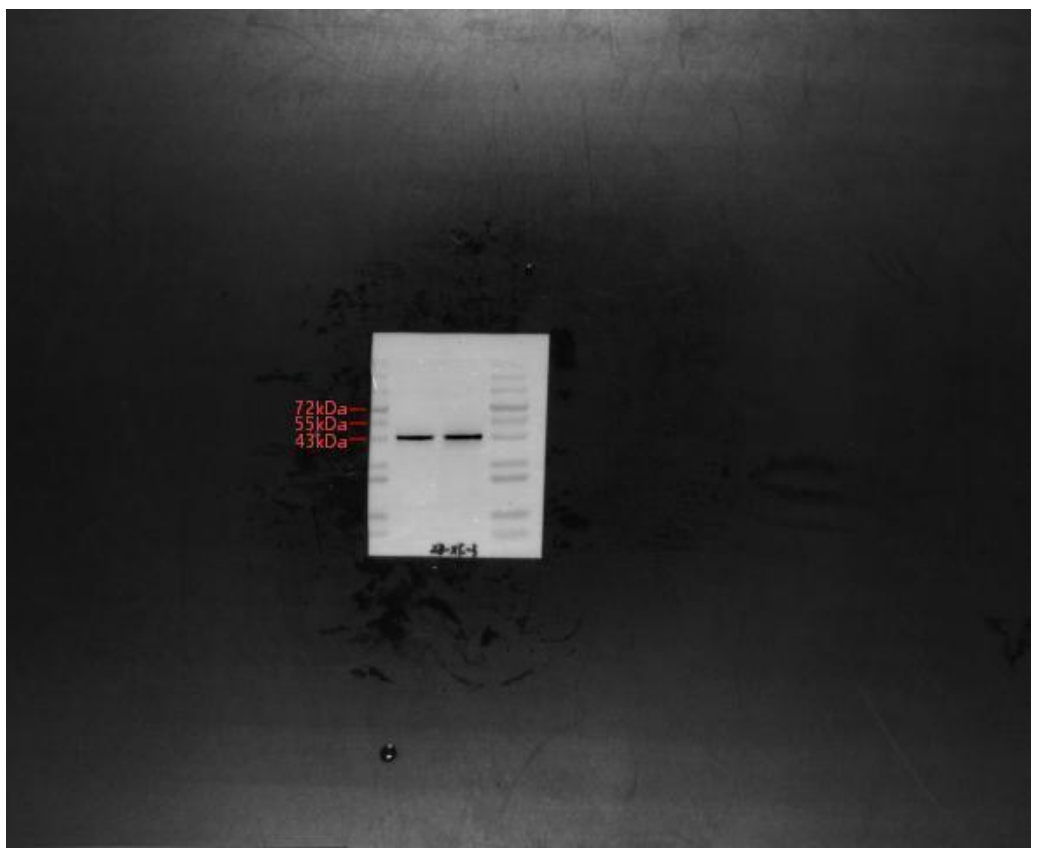

ACTIN

U251R

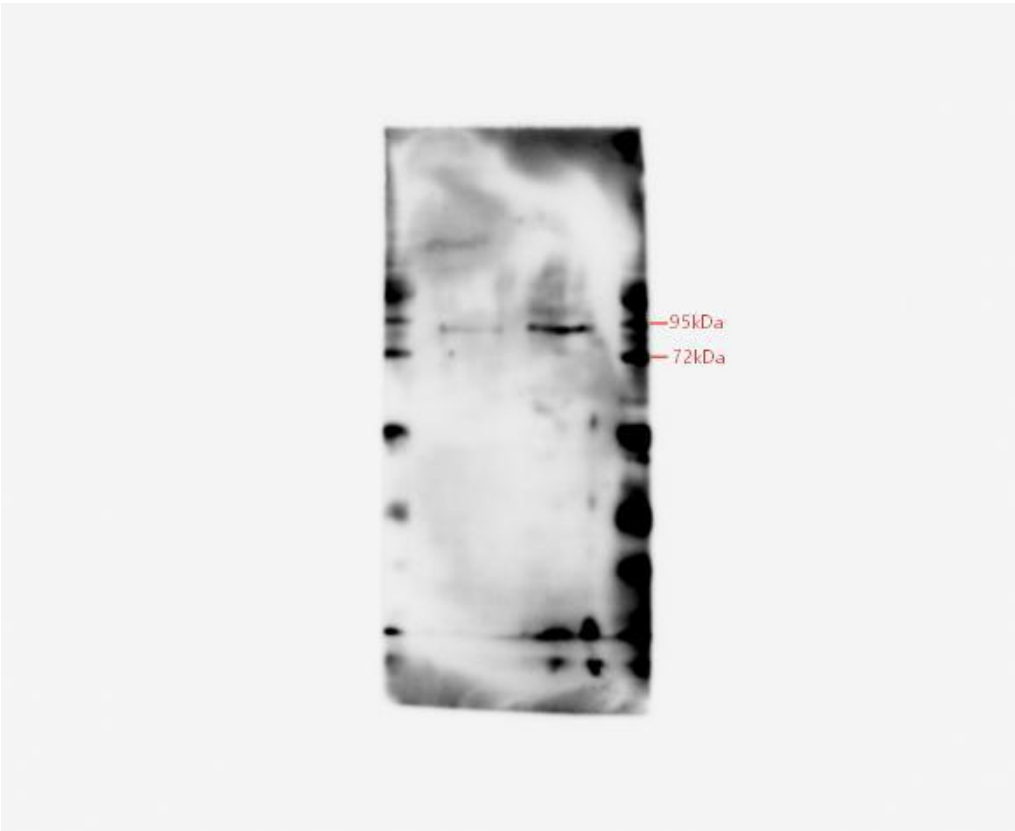

PLOD1

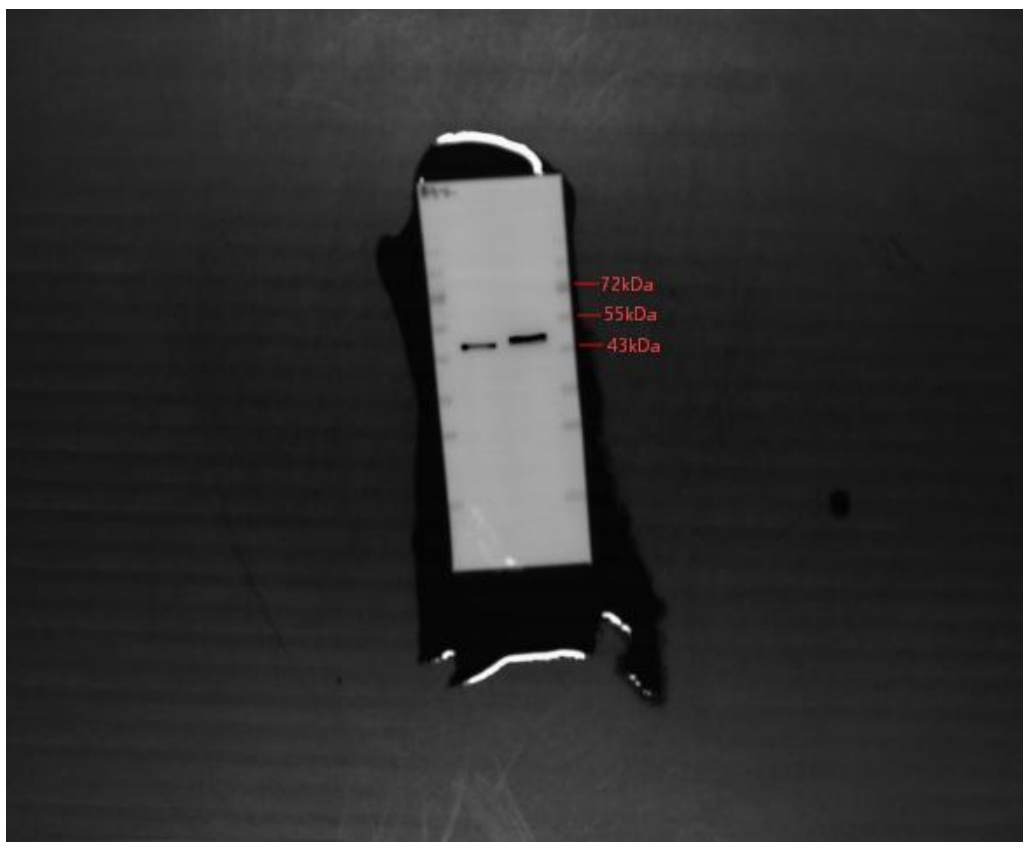

ENO1

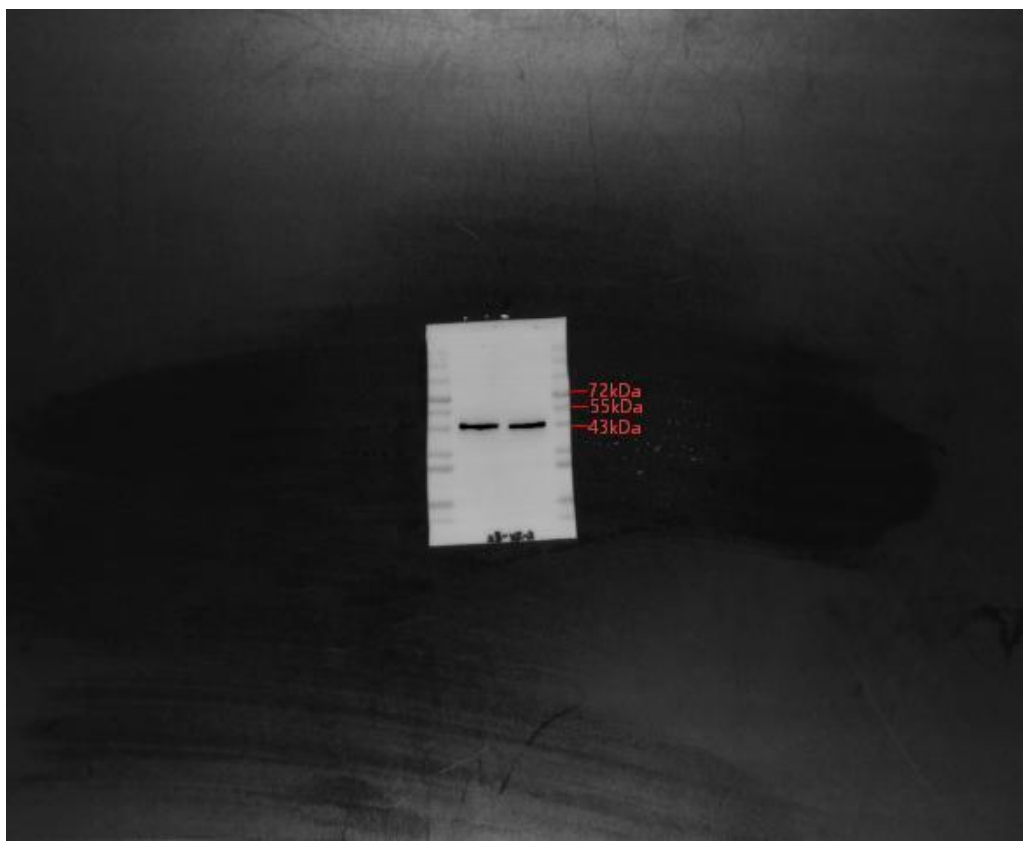

ACTIN

FIG.3C

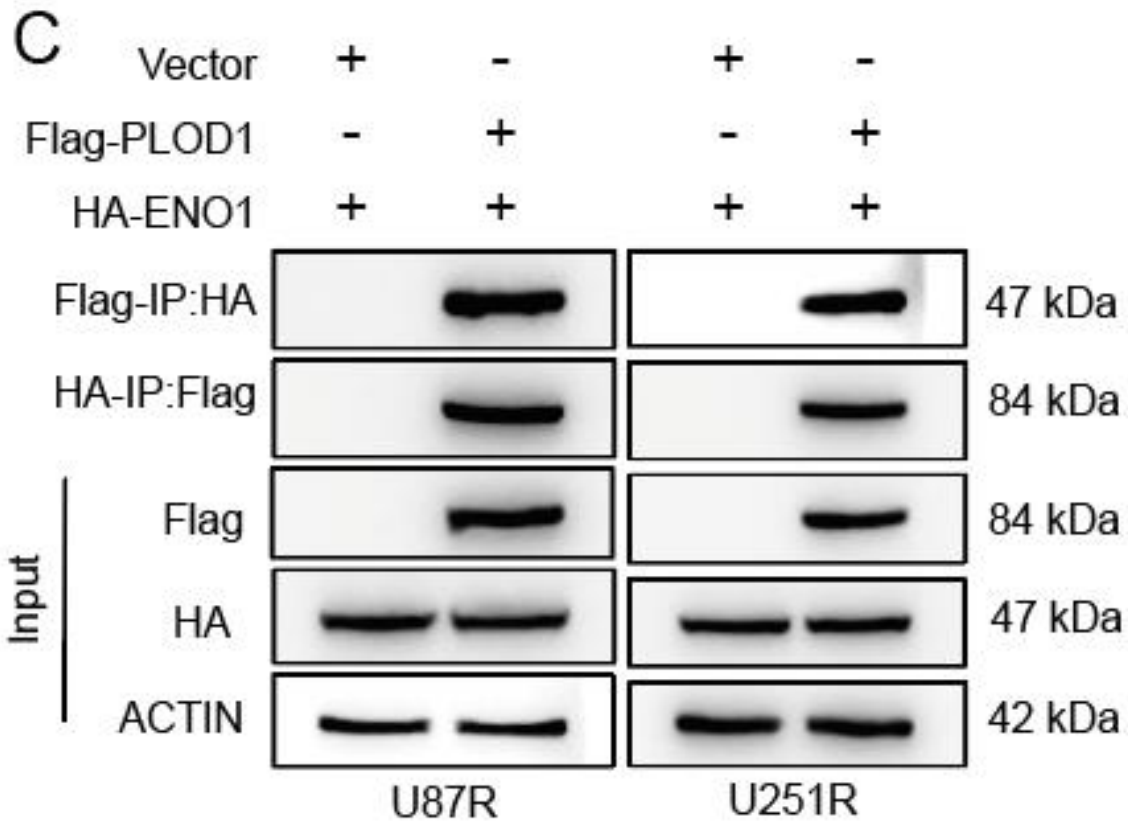

U87R

IP

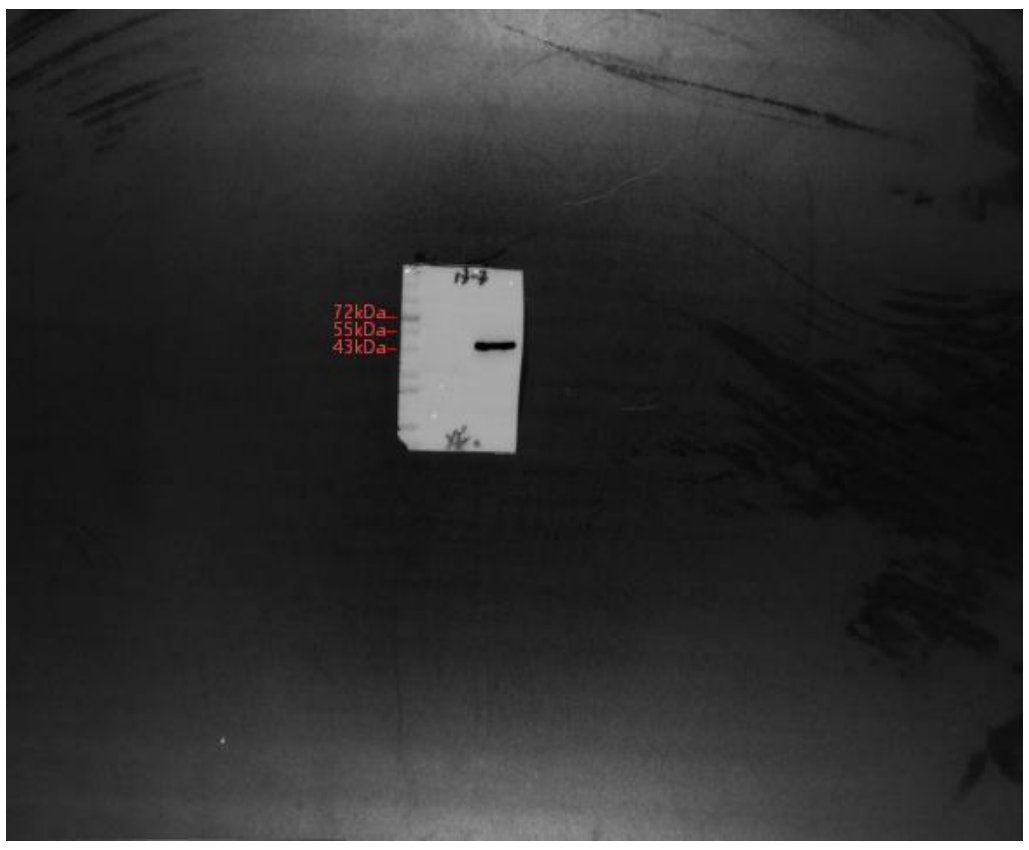

ENO1

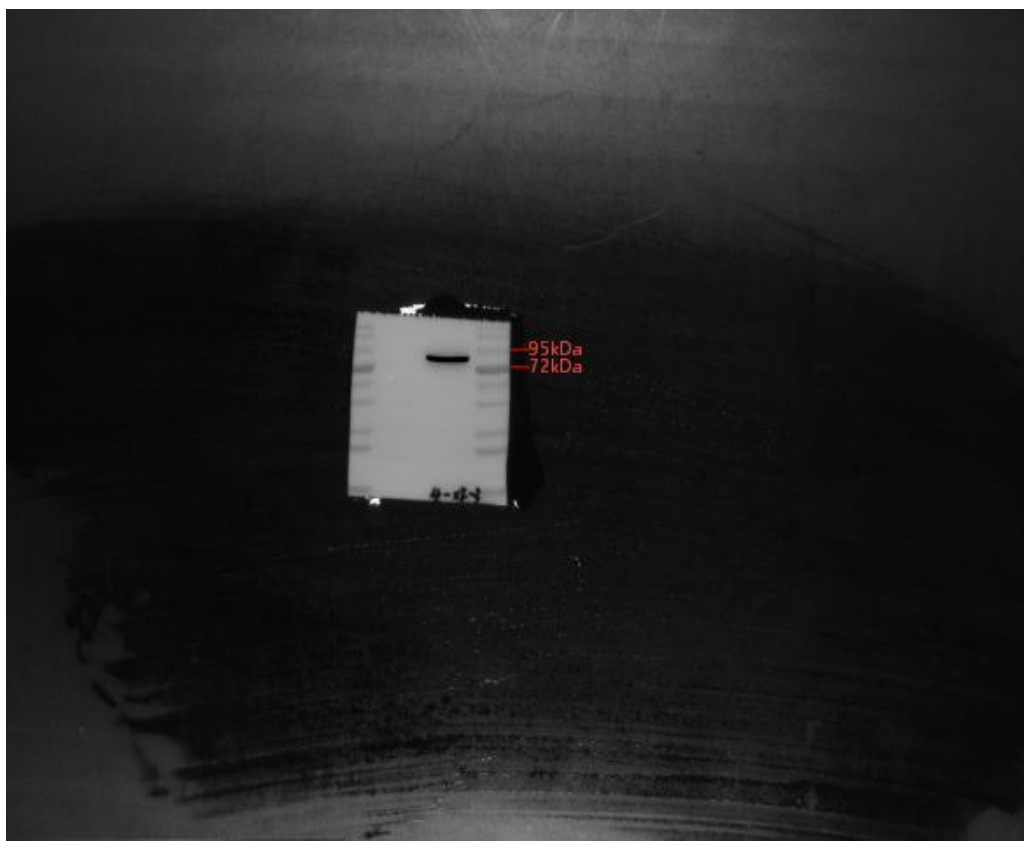

PLOD1

Input

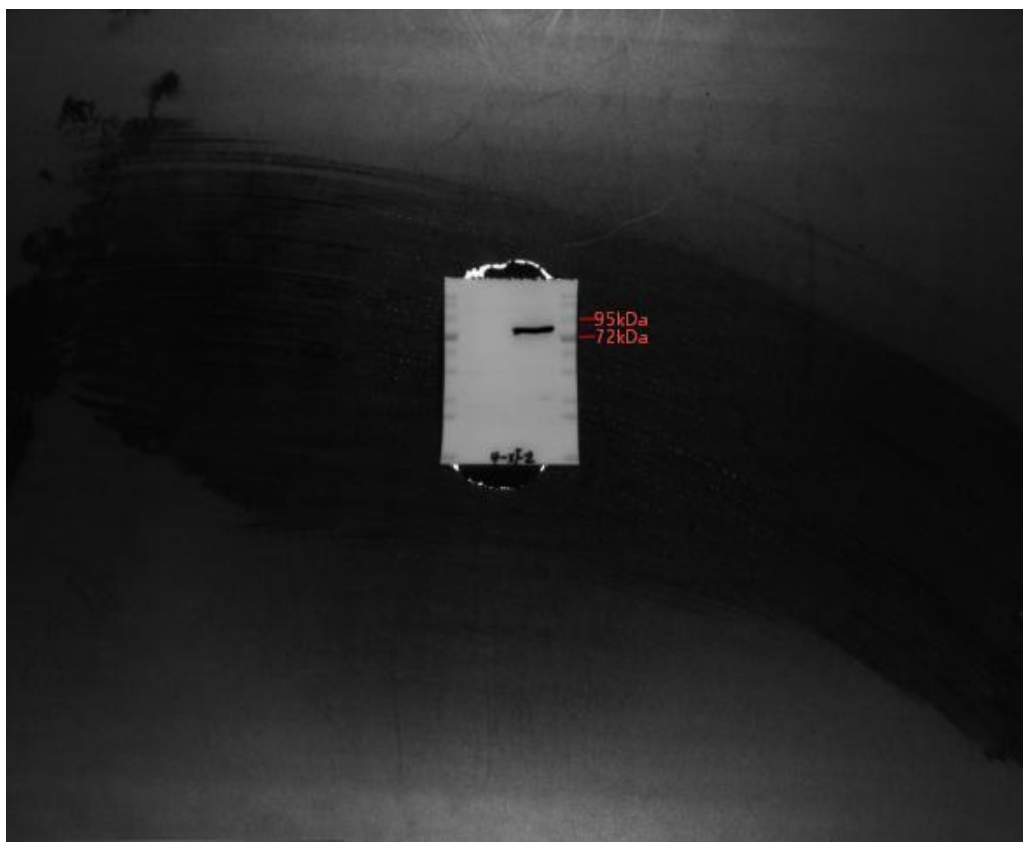

PLOD1

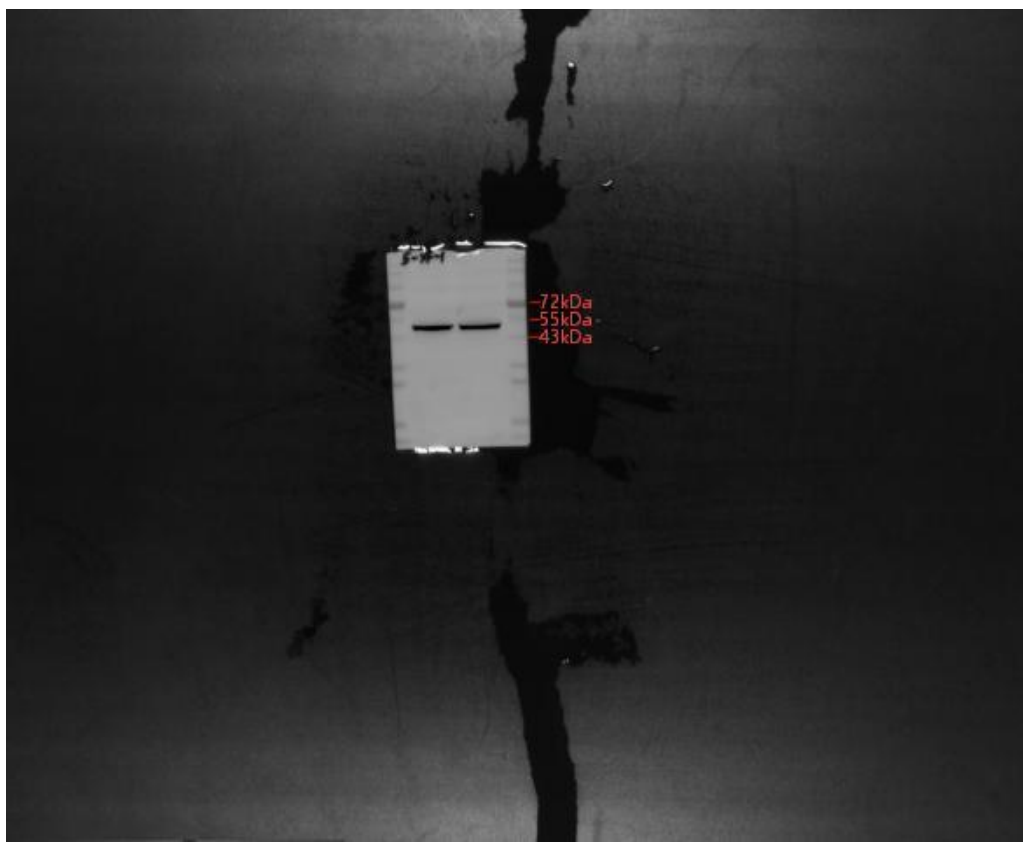

ENO1

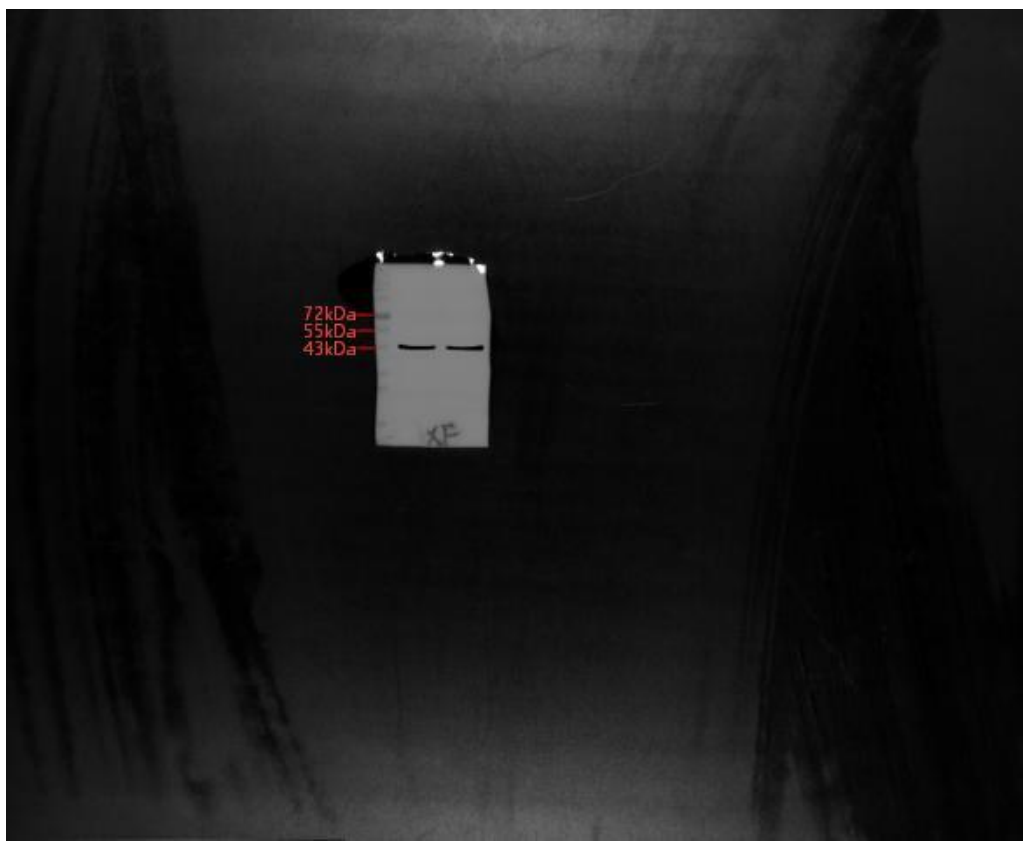

ACTIN

U251R

IP

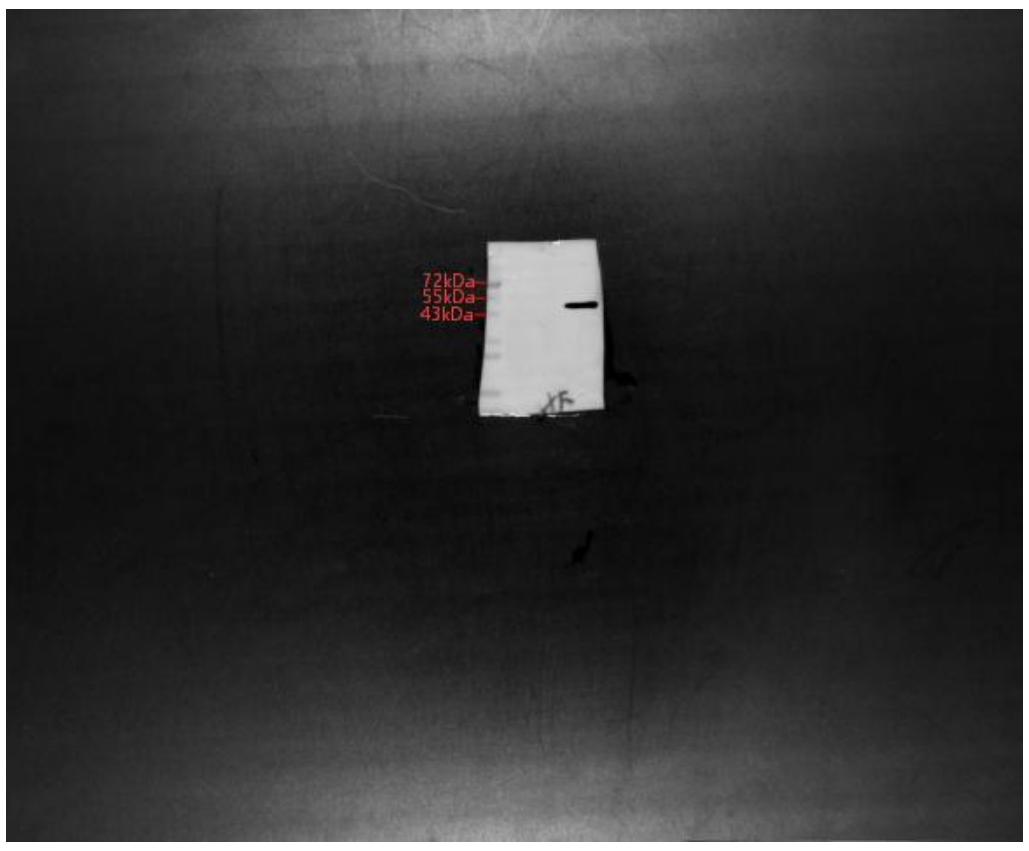

ENO1

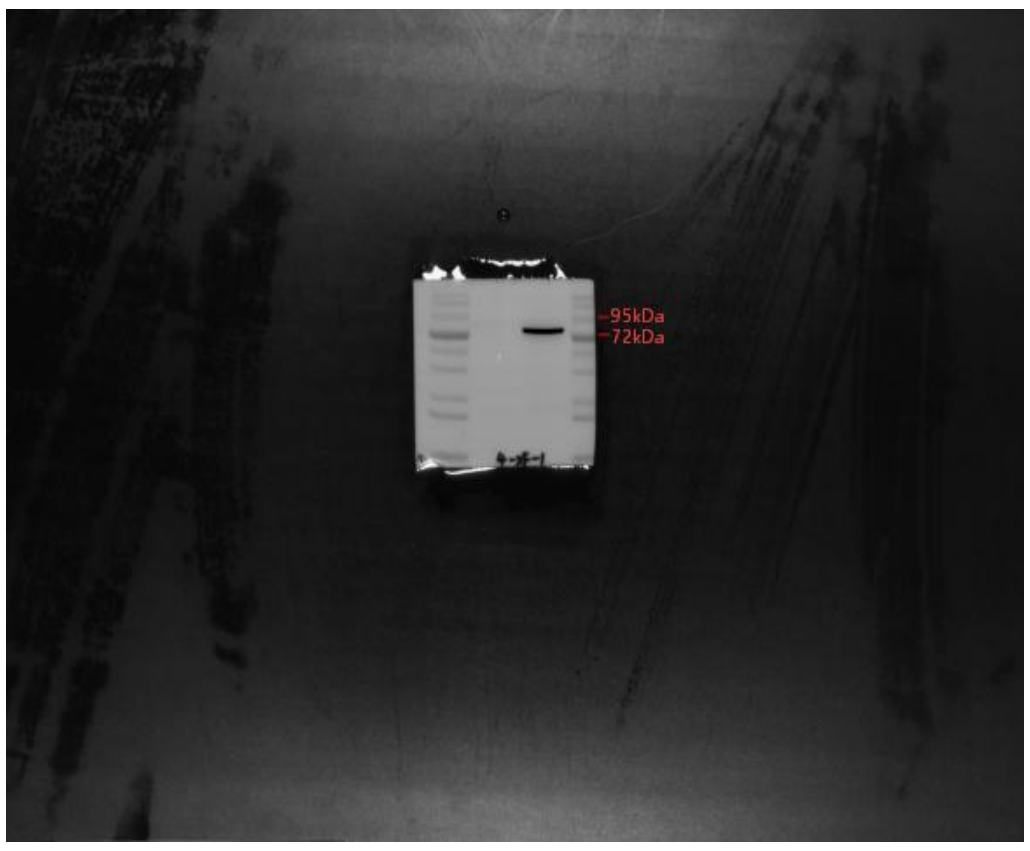

PLOD1

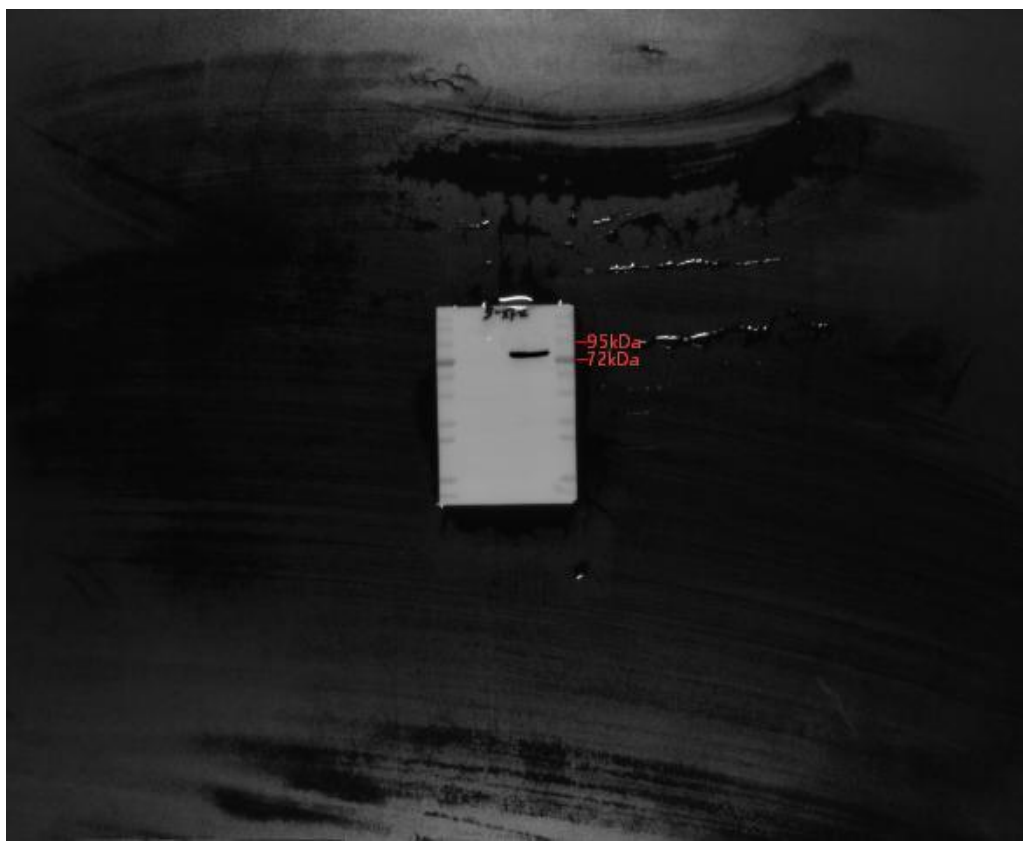

PLOD1

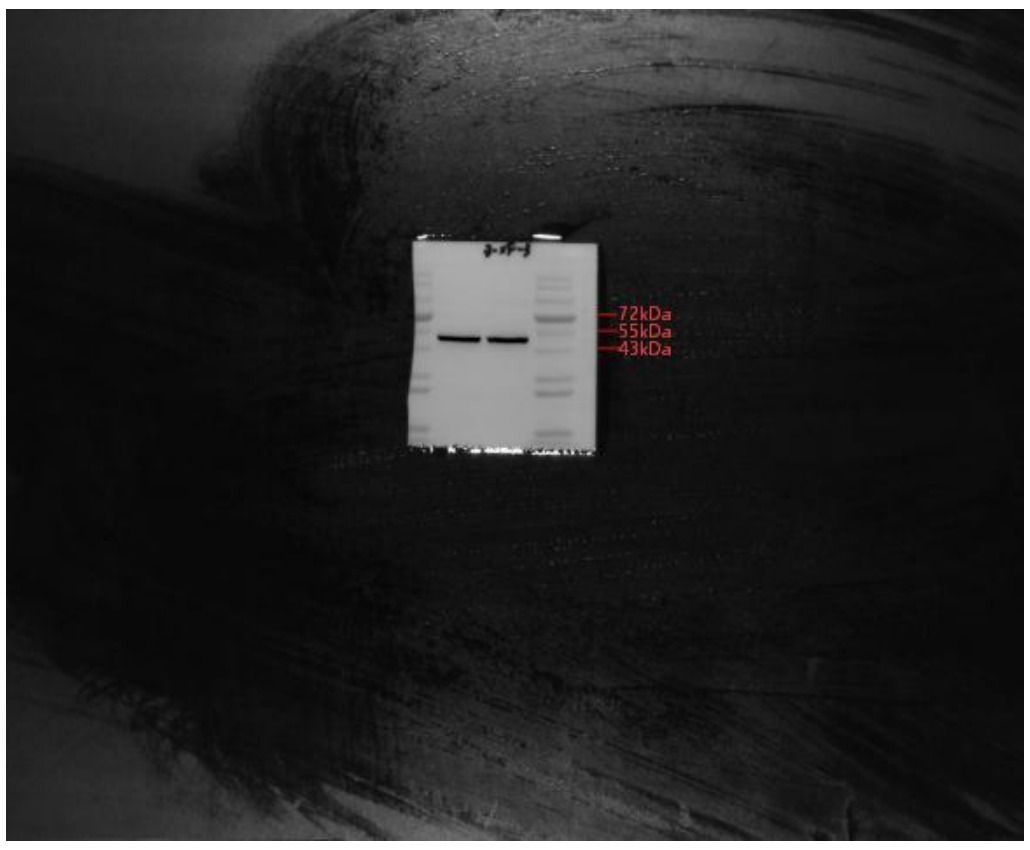

ENO1

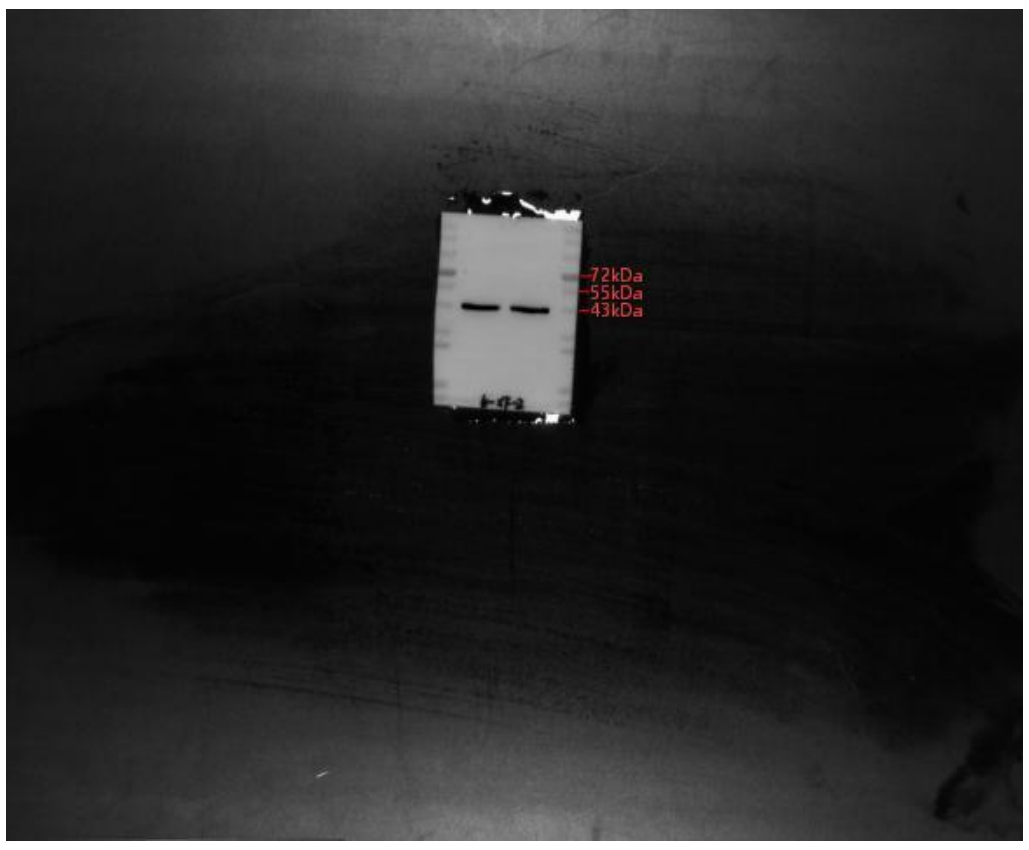

ACTIN

FIG.3E

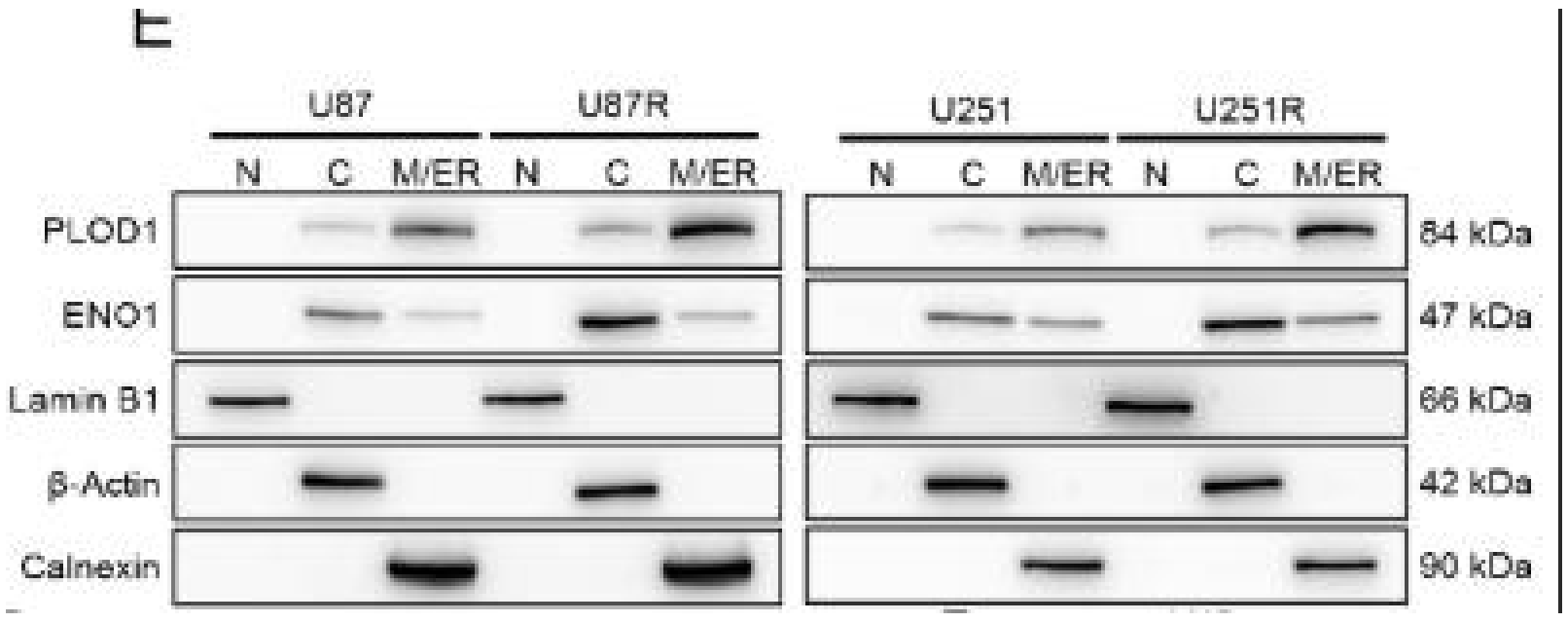

U87 vs U87R

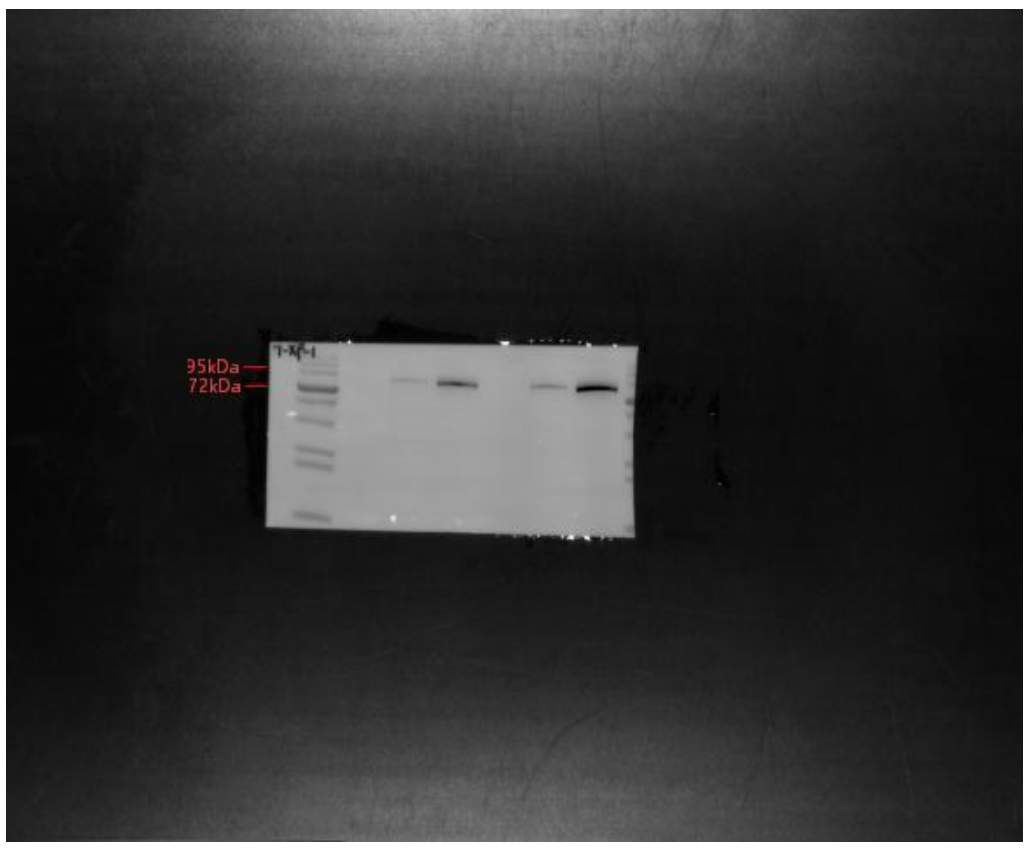

PLOD1

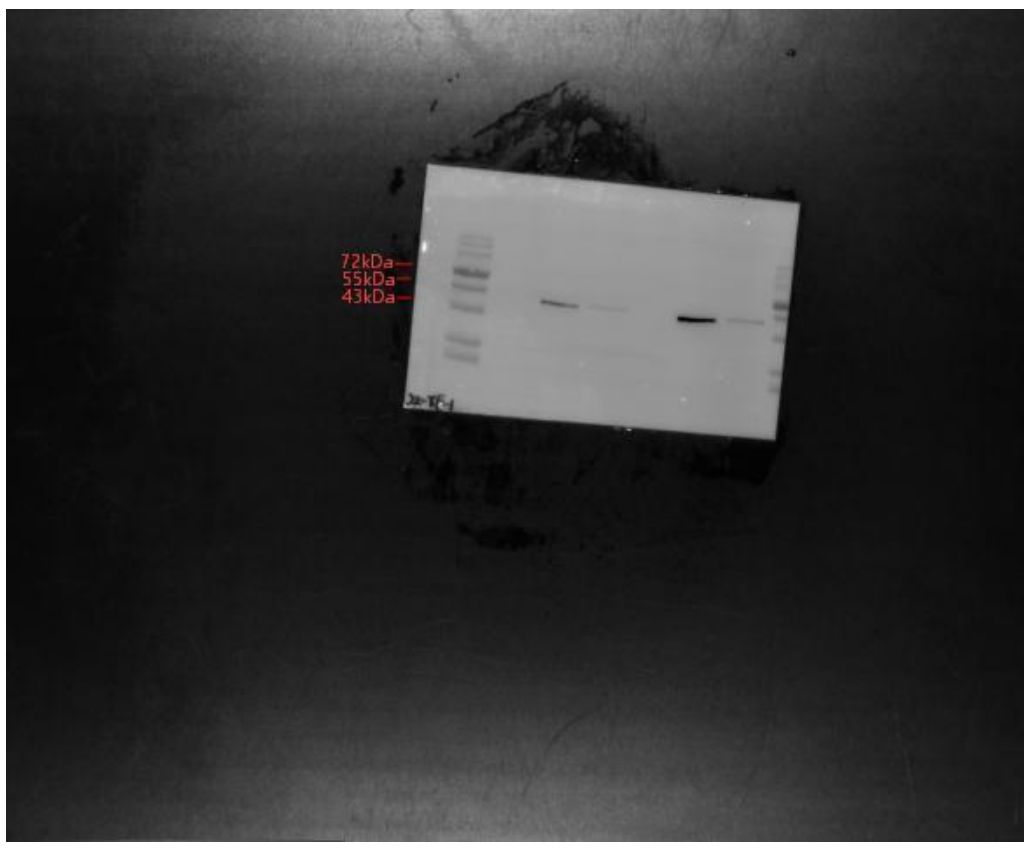

ENO1

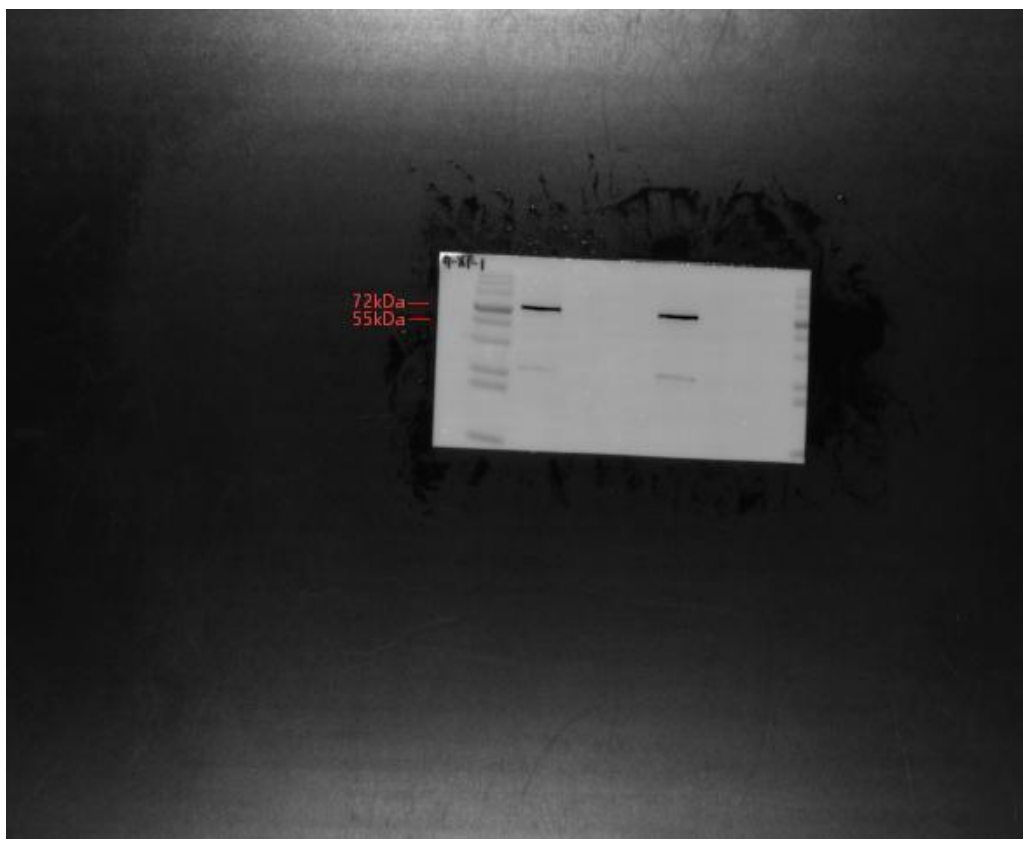

Lamin B1

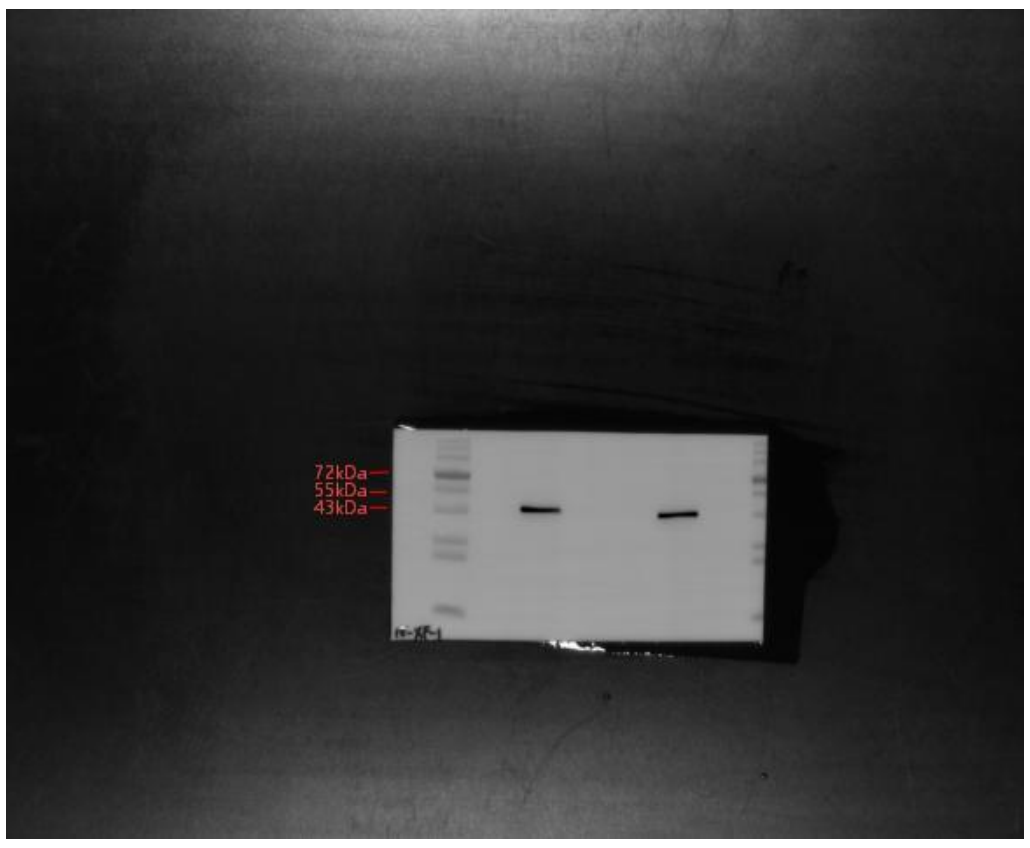

actin

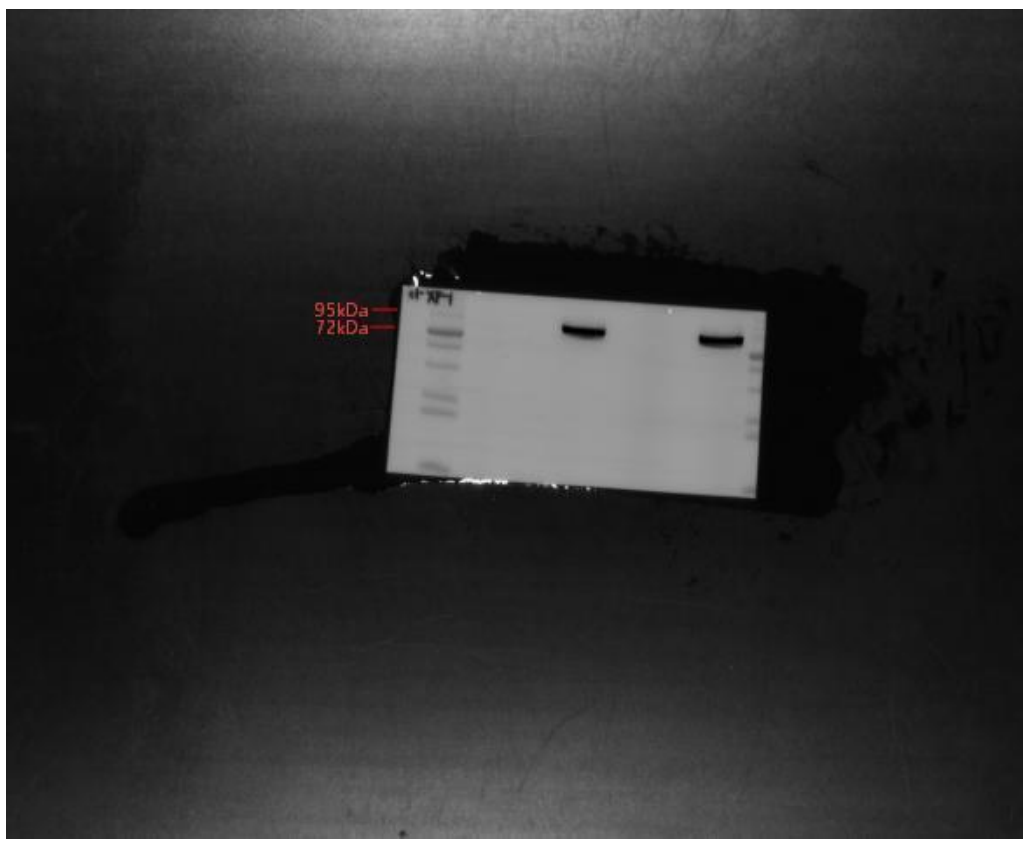

Calnexin

U251 vs U251R

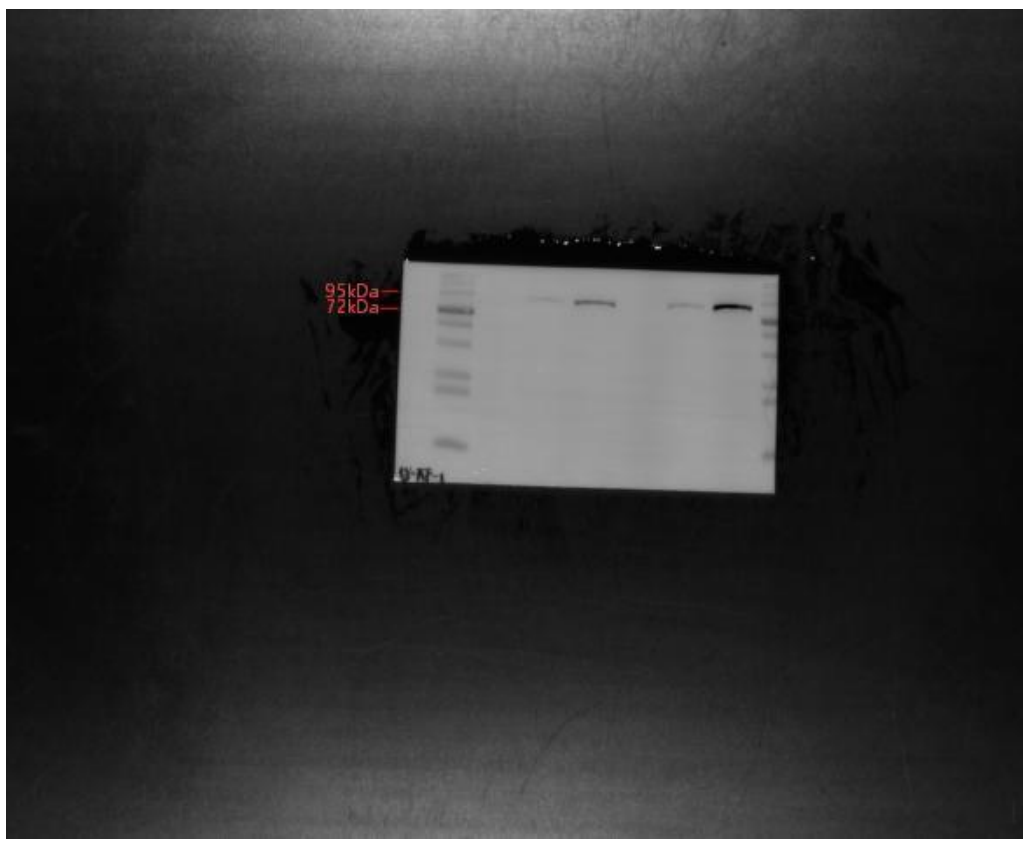

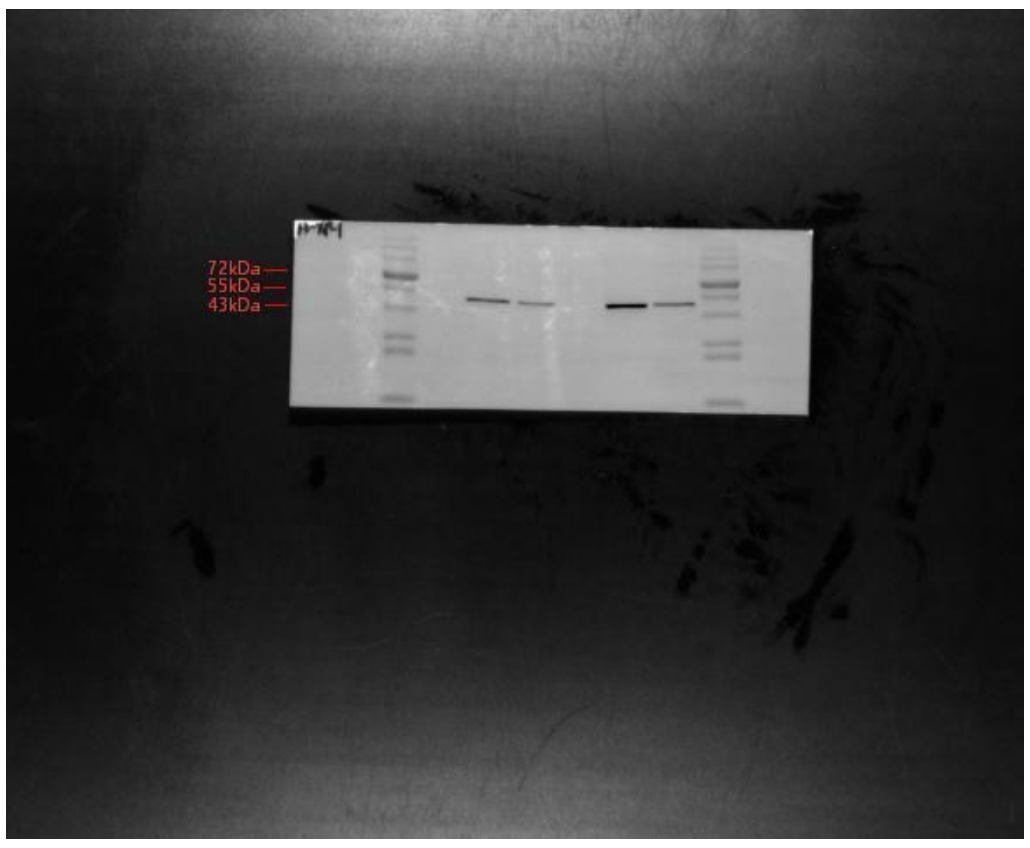

PLOD1

ENO1

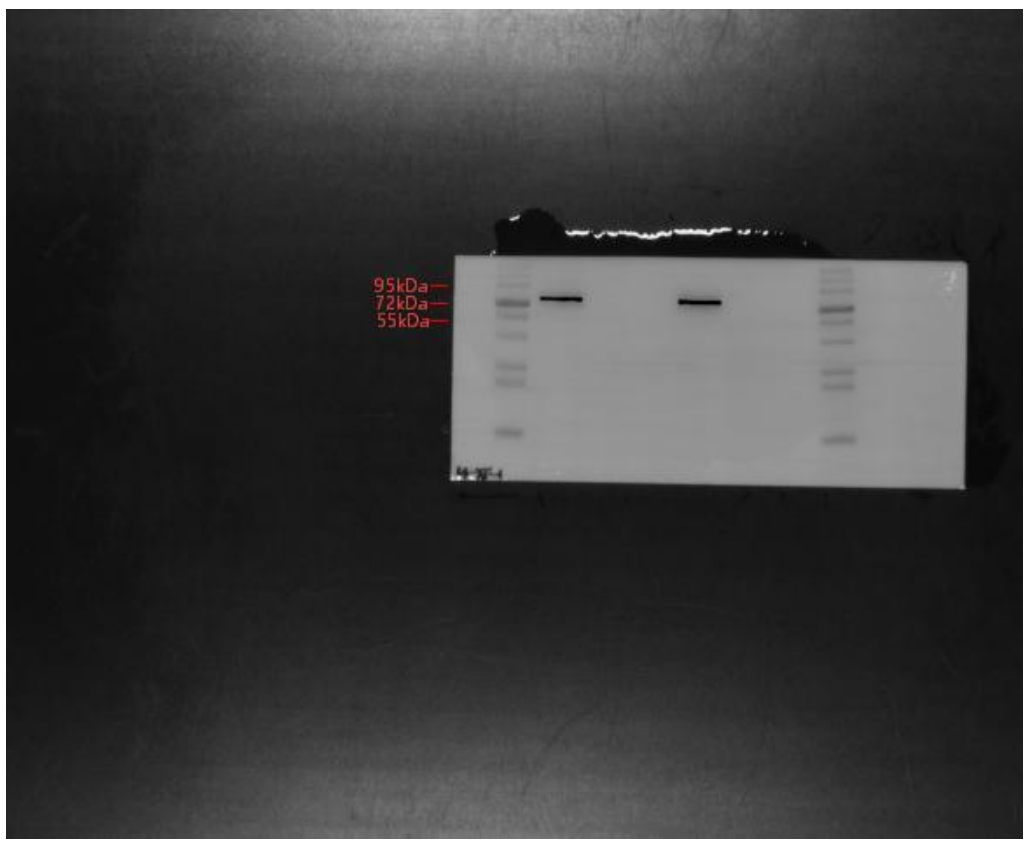

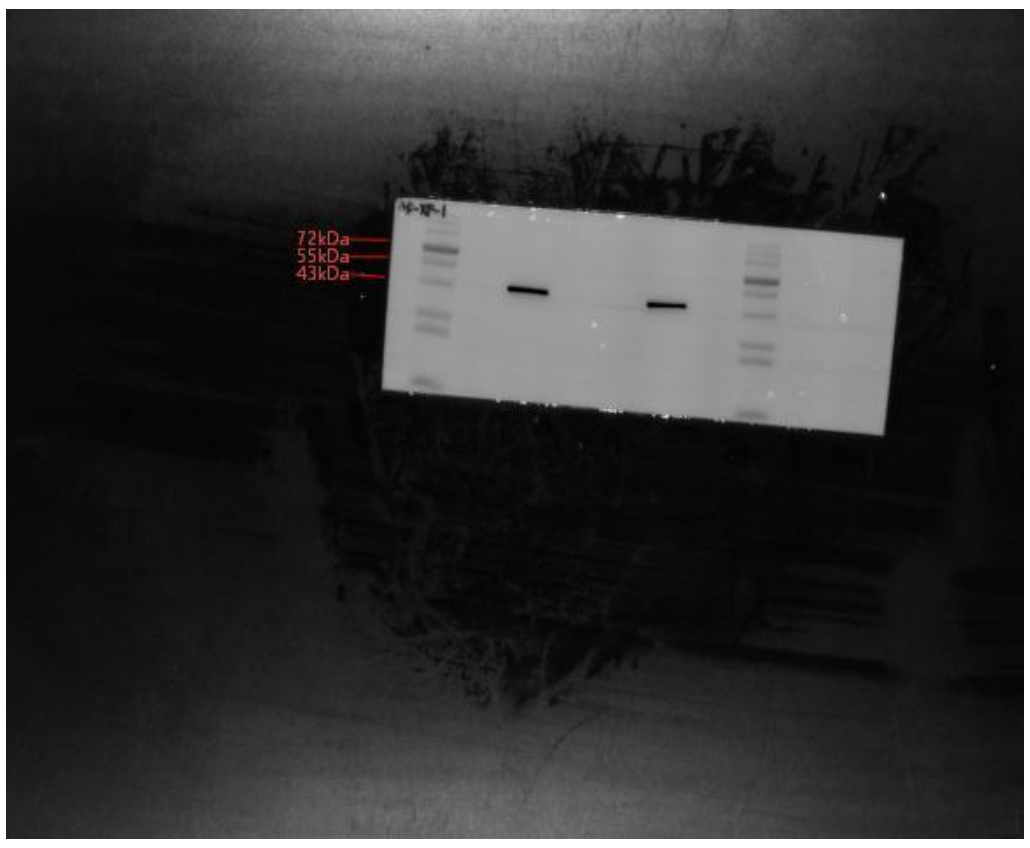

Lamin B1

actin

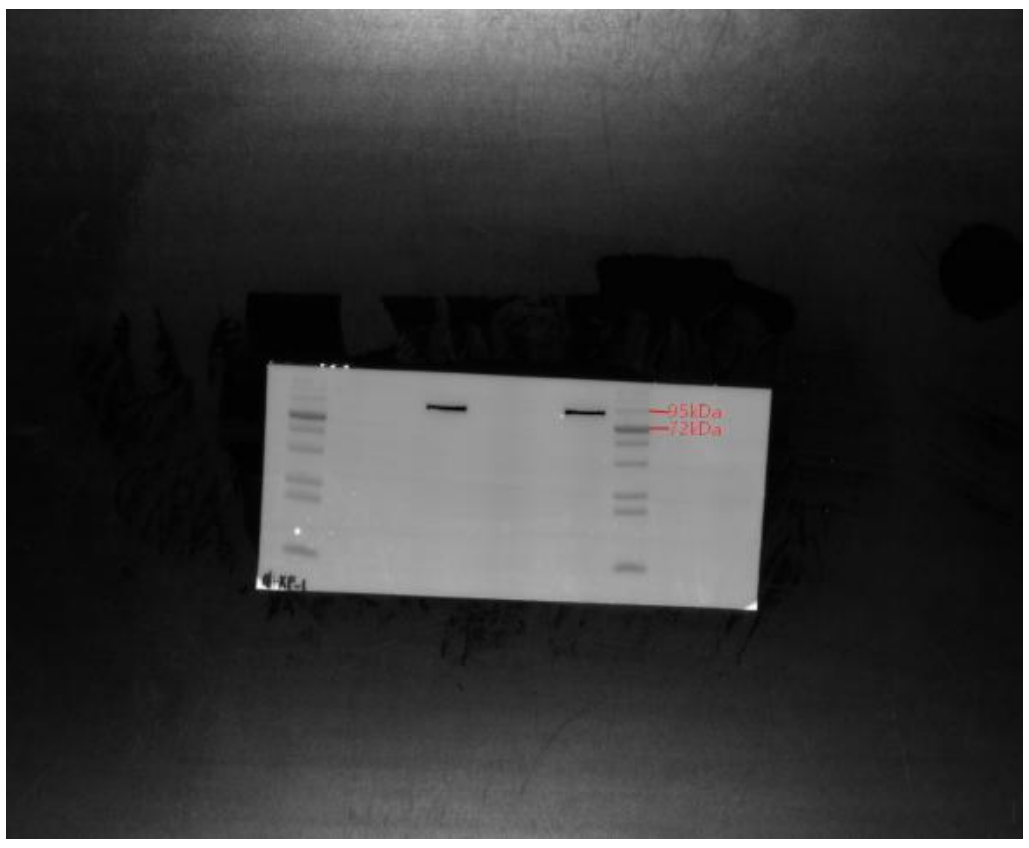

Calnexin

FIG.3G

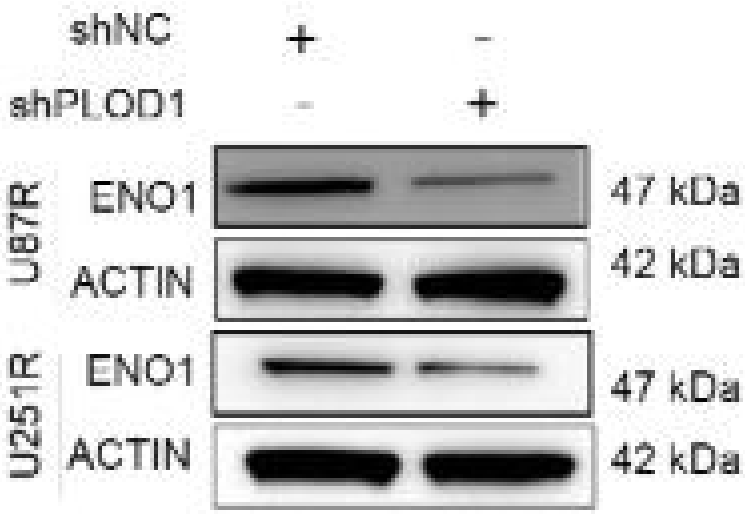

U87R

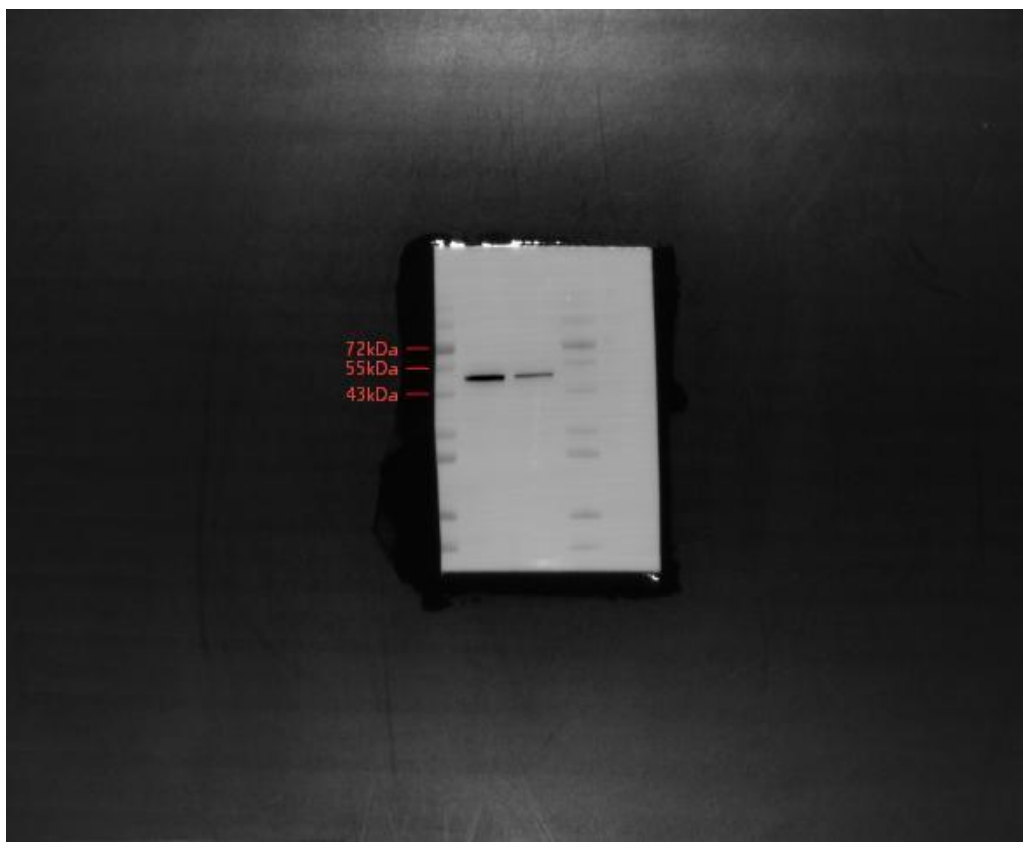

ENO1

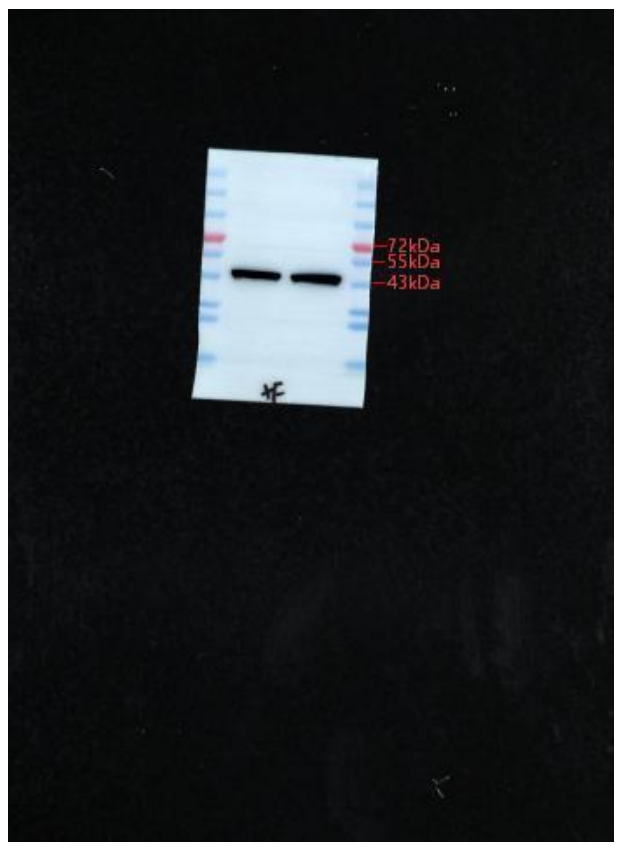

ACTIN

U251R

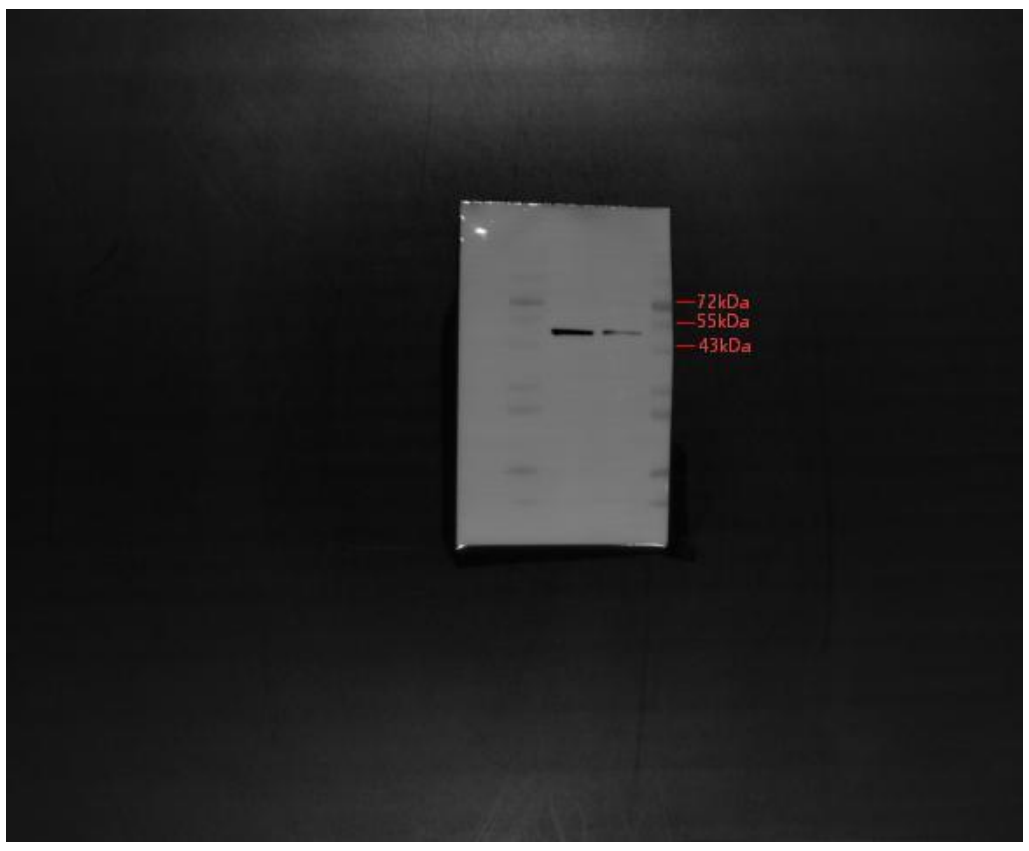

ENO1

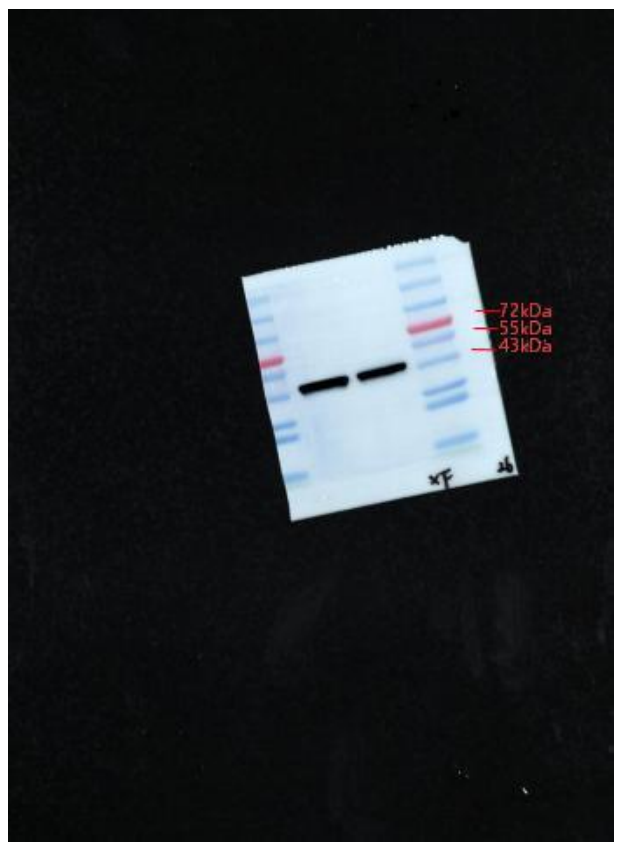

ACTIN

FIG.3H

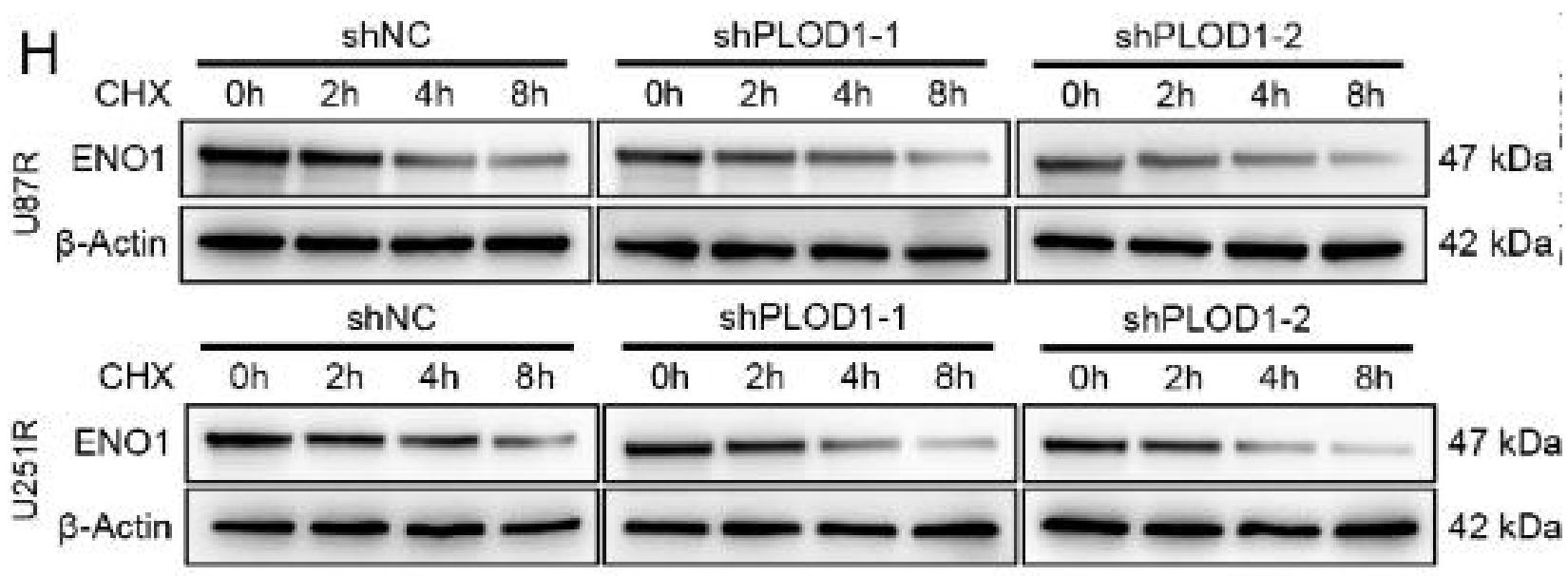

U87R

ShNC

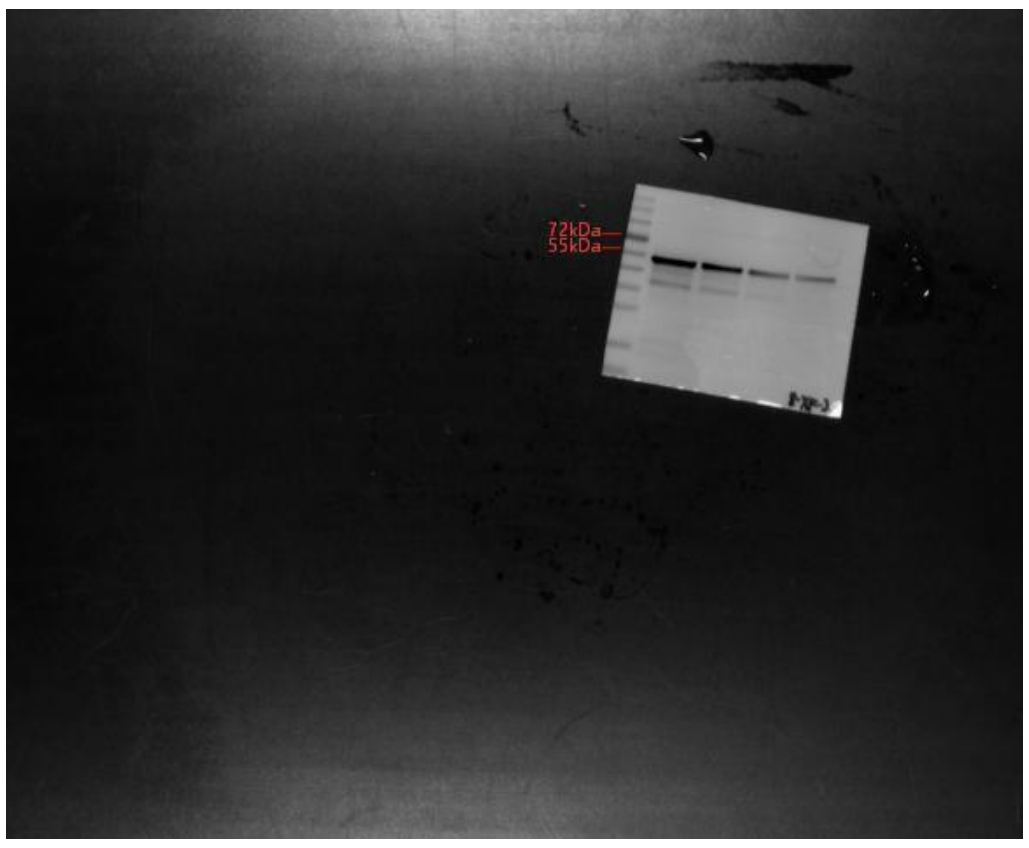

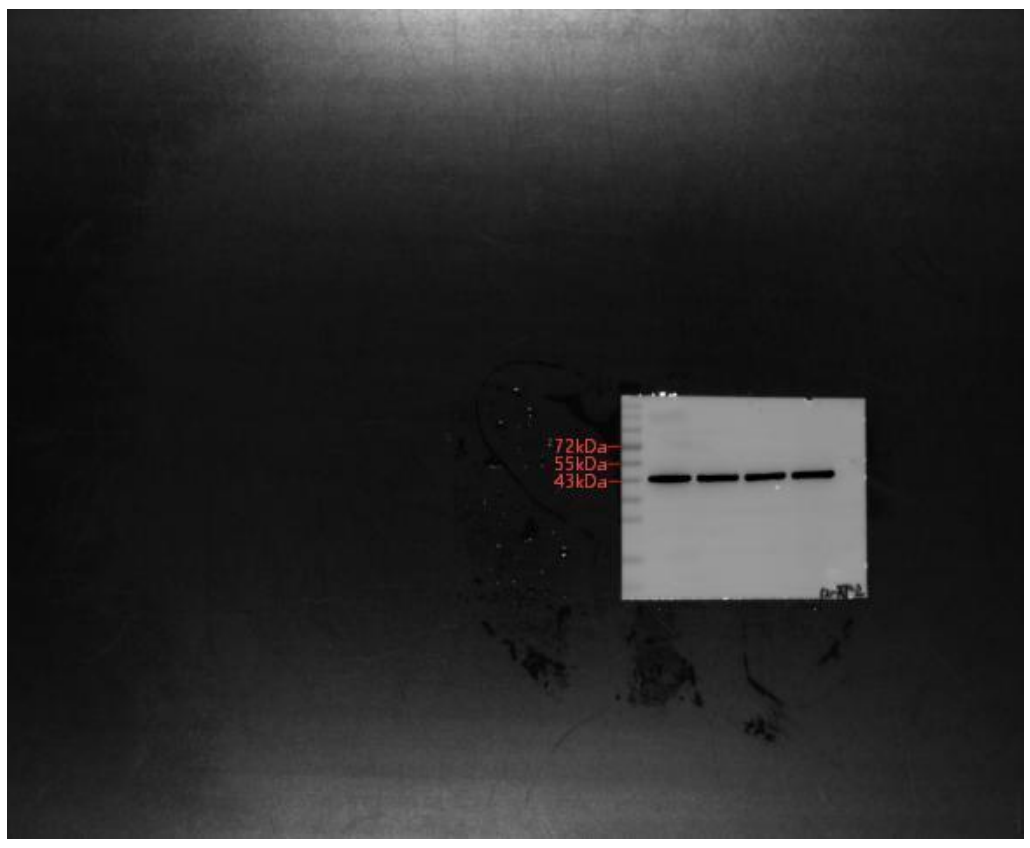

ENO1 ACTIN

ShPLOD1-1

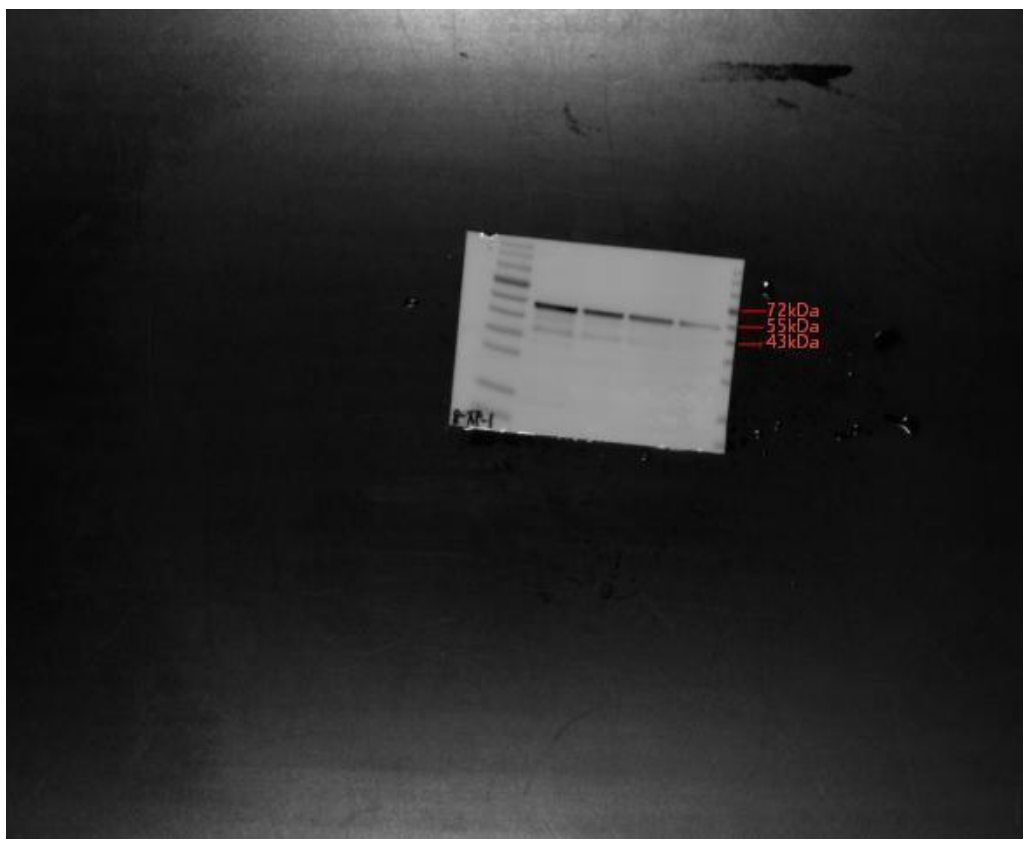

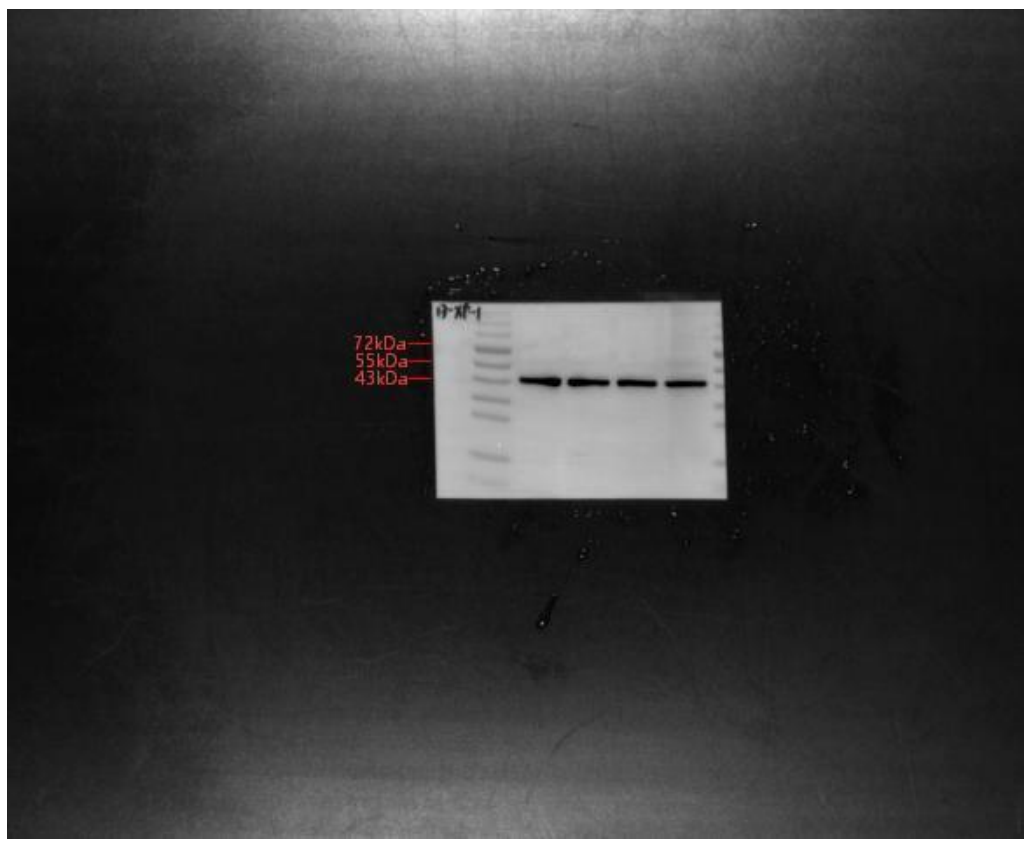

ENO1 ACTIN

ShPLOD1-2

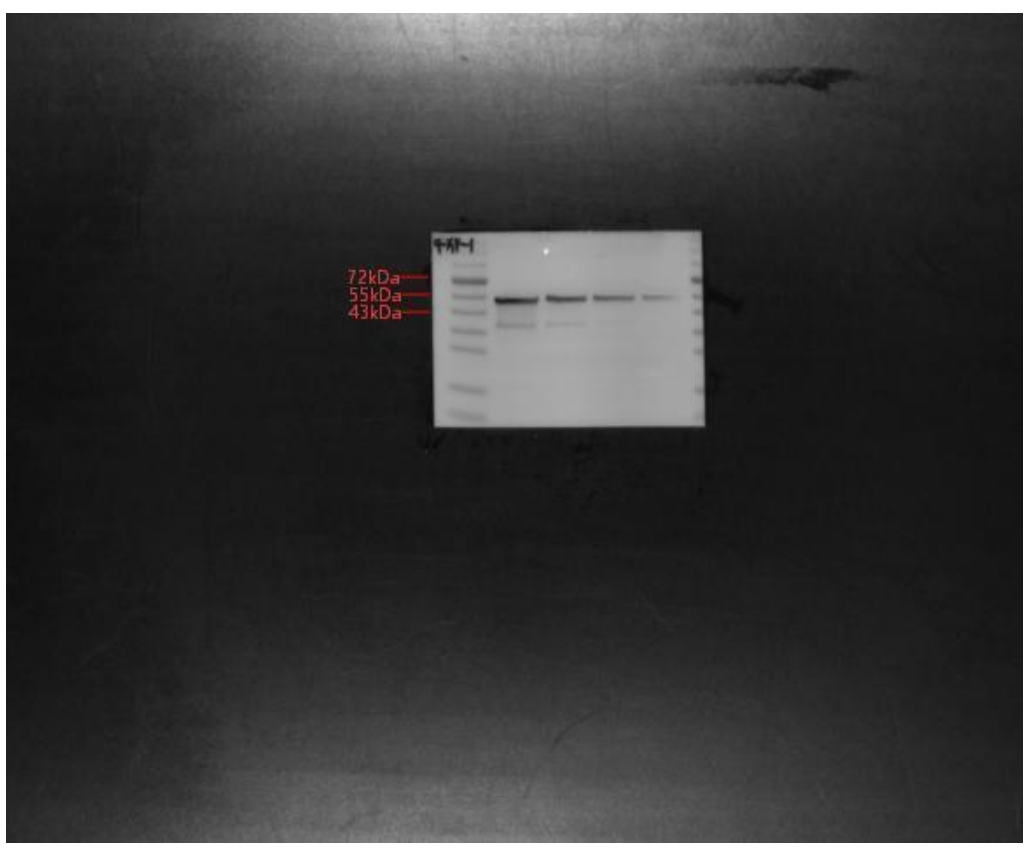

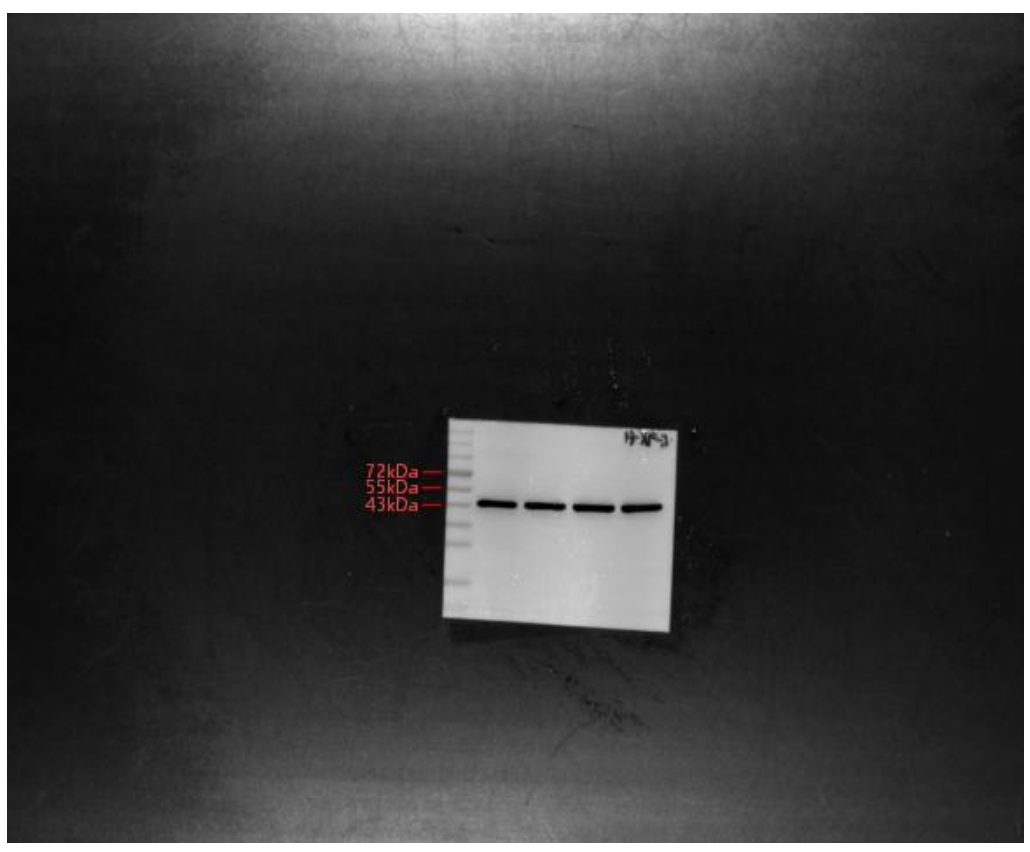

ENO1 ACTIN

U251R

ShNC

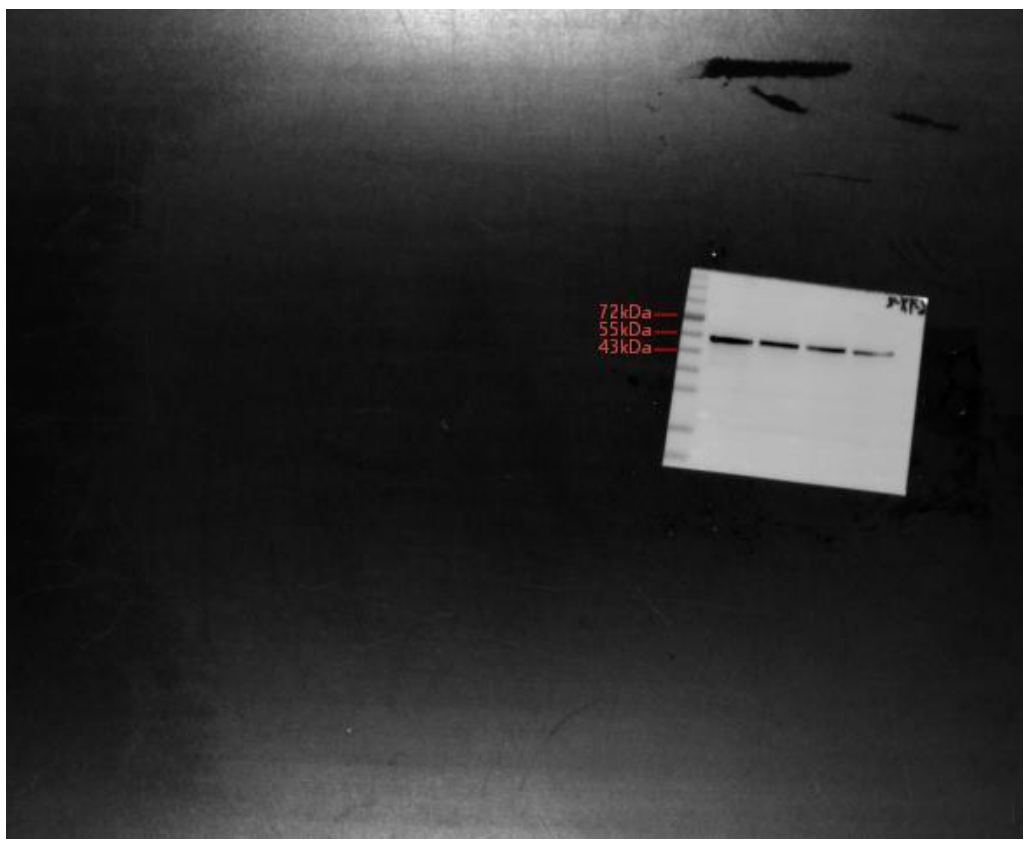

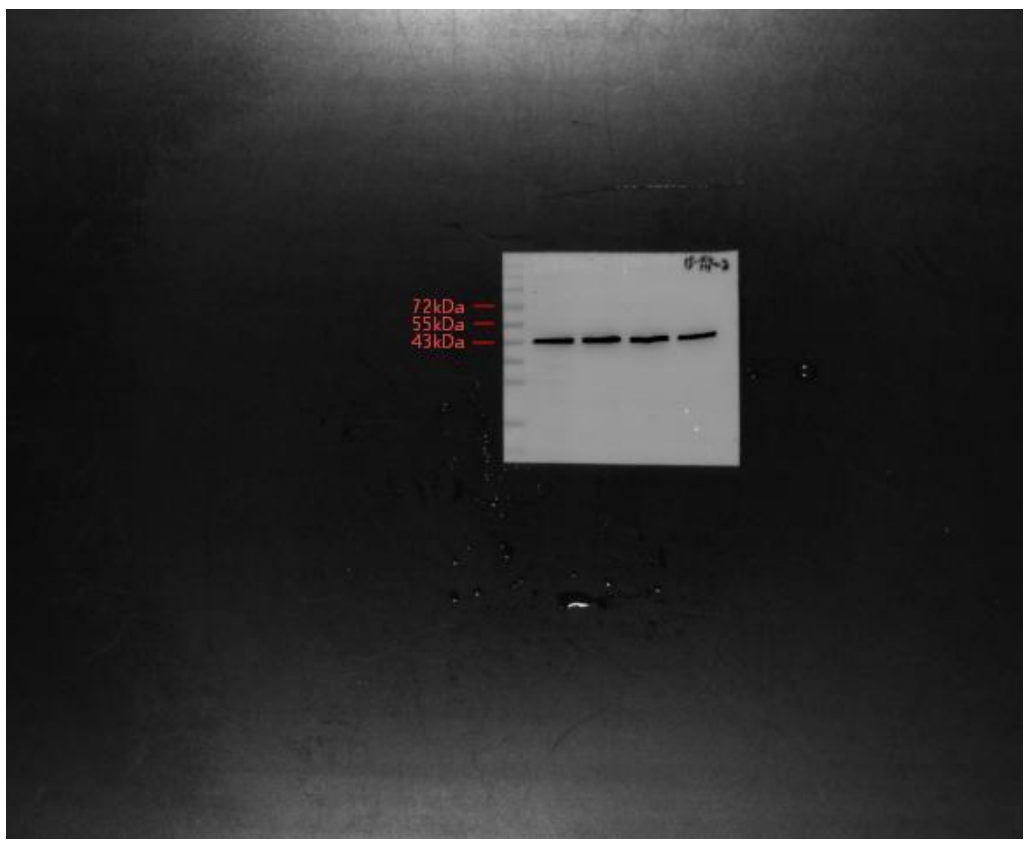

ENO1 ACTIN

ShPLOD1-1

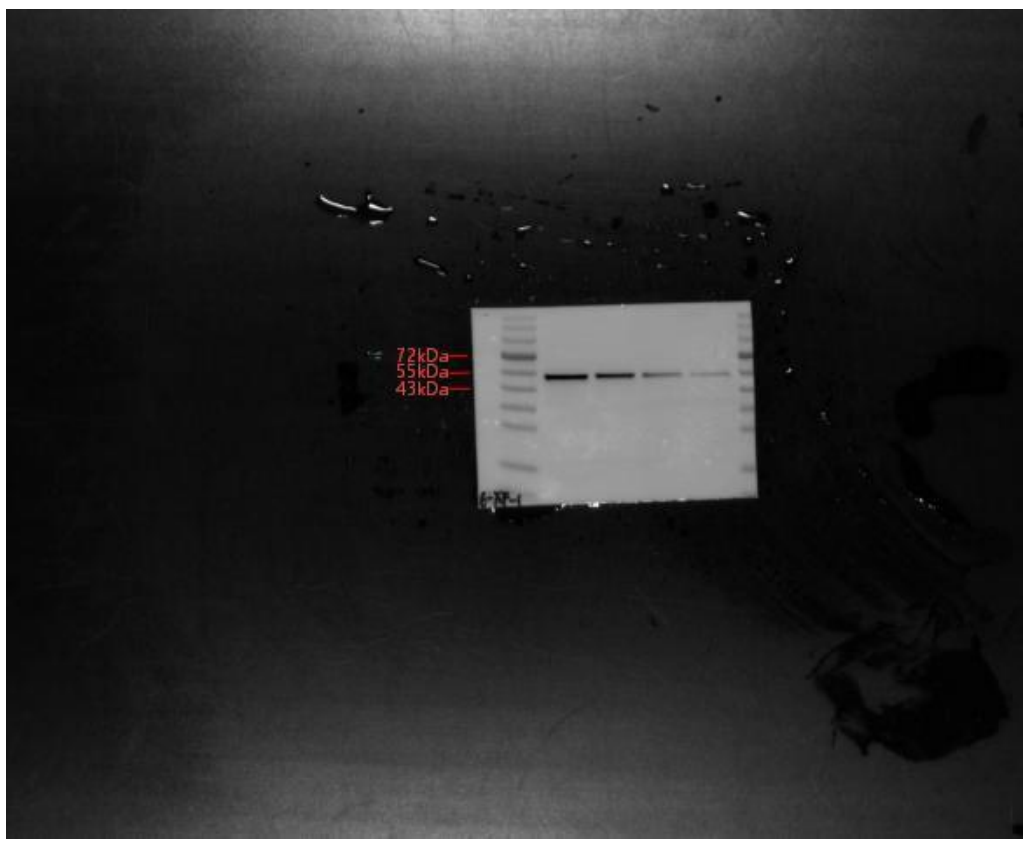

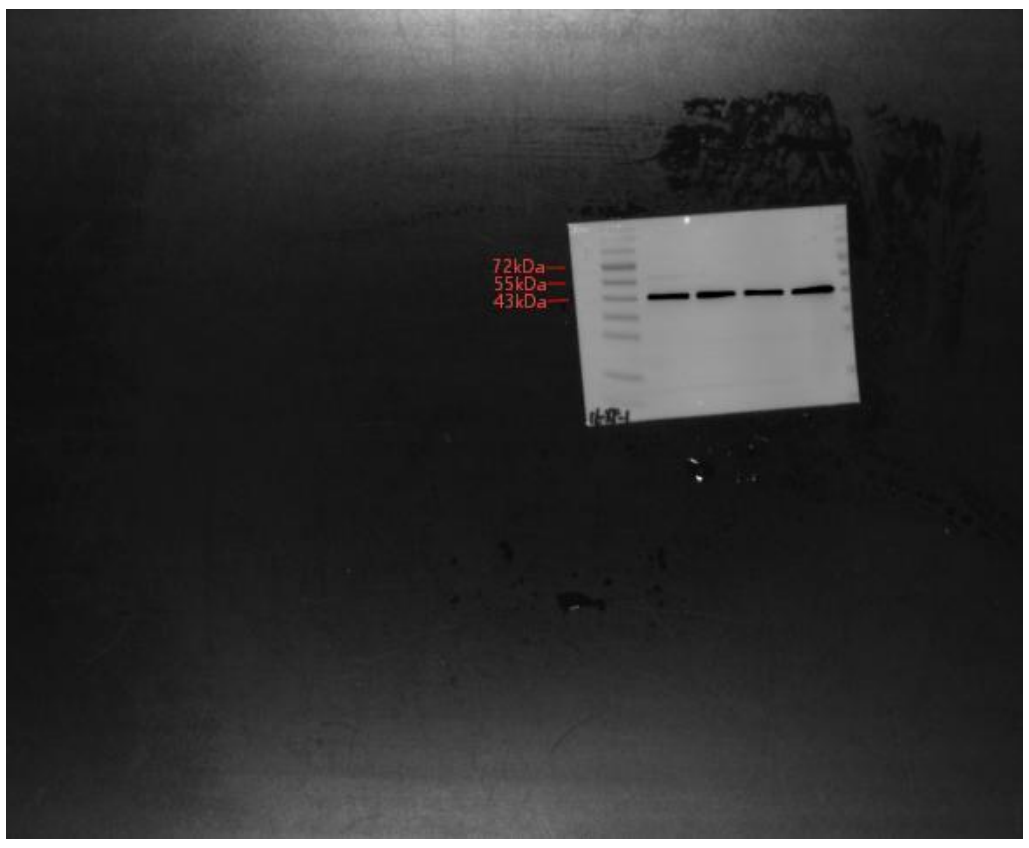

ENO1 ACTIN

ShPLOD1-2

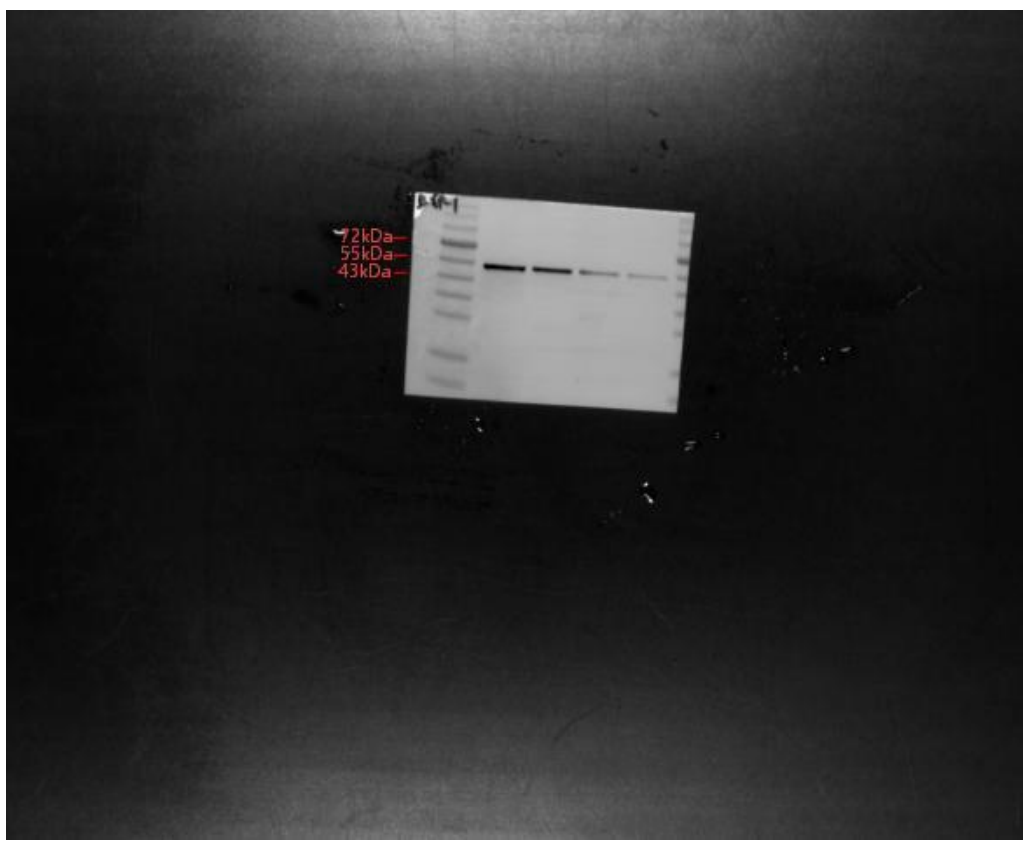

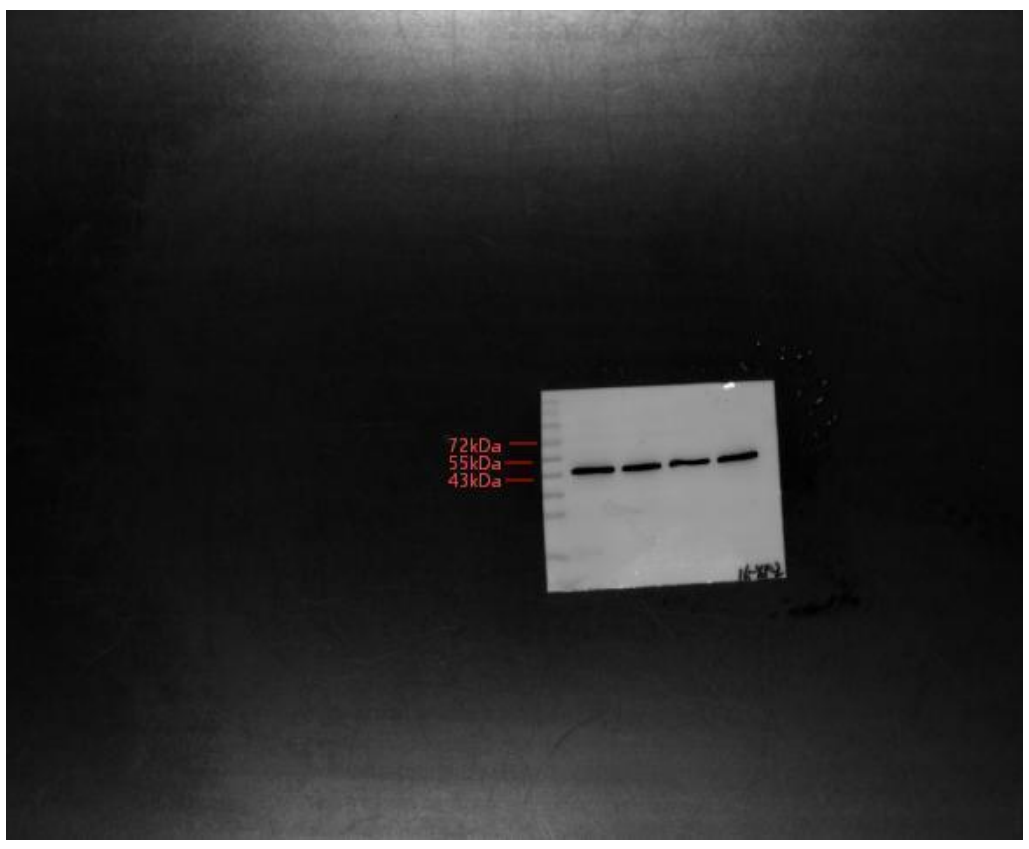

ENO1 ACTIN

FIG.3I

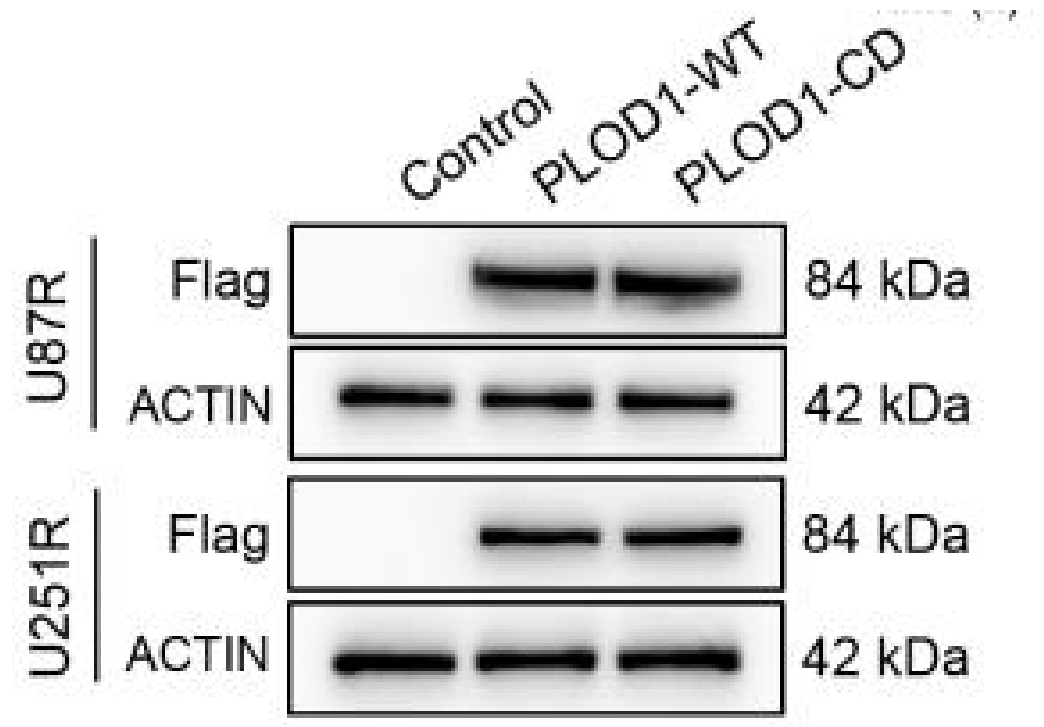

U87R

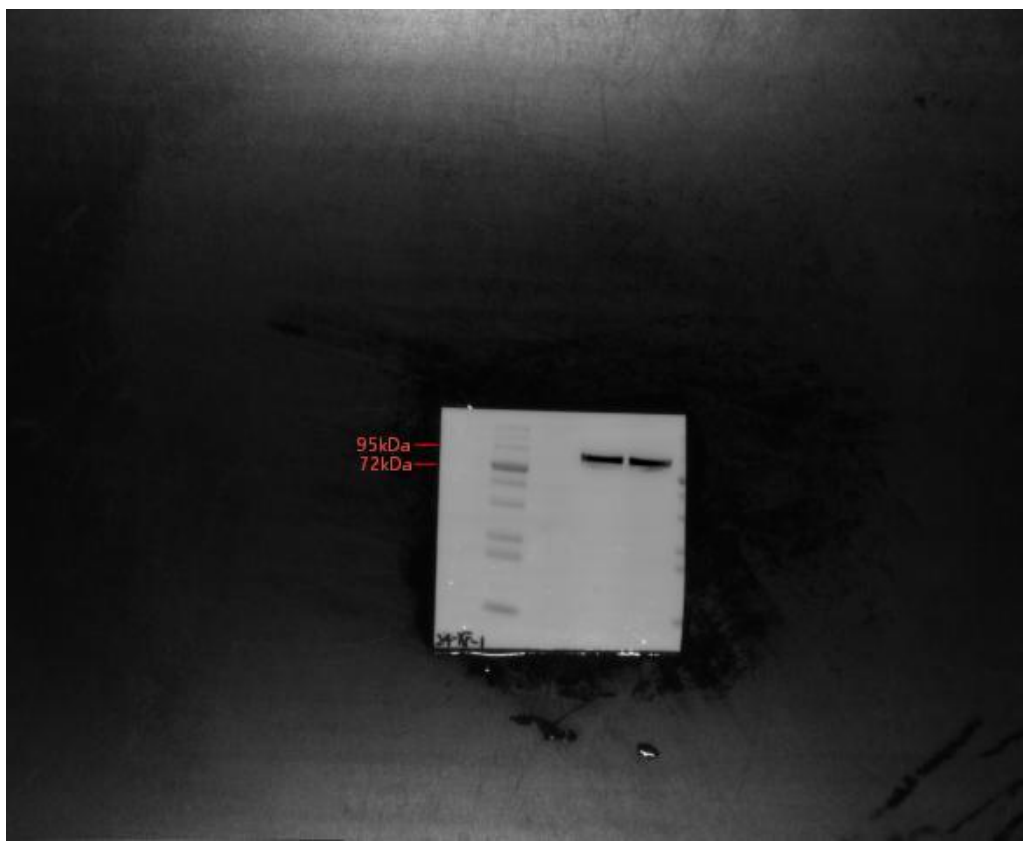

Flag-PLOD1

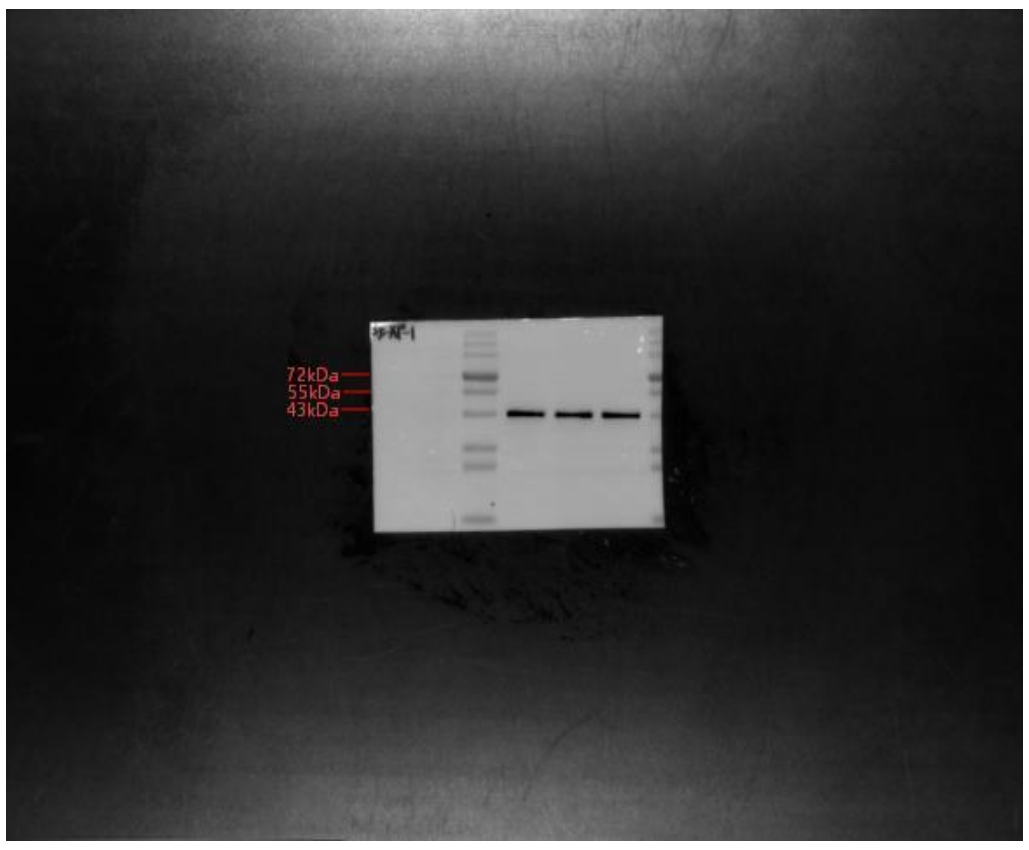

ACTIN

U251R

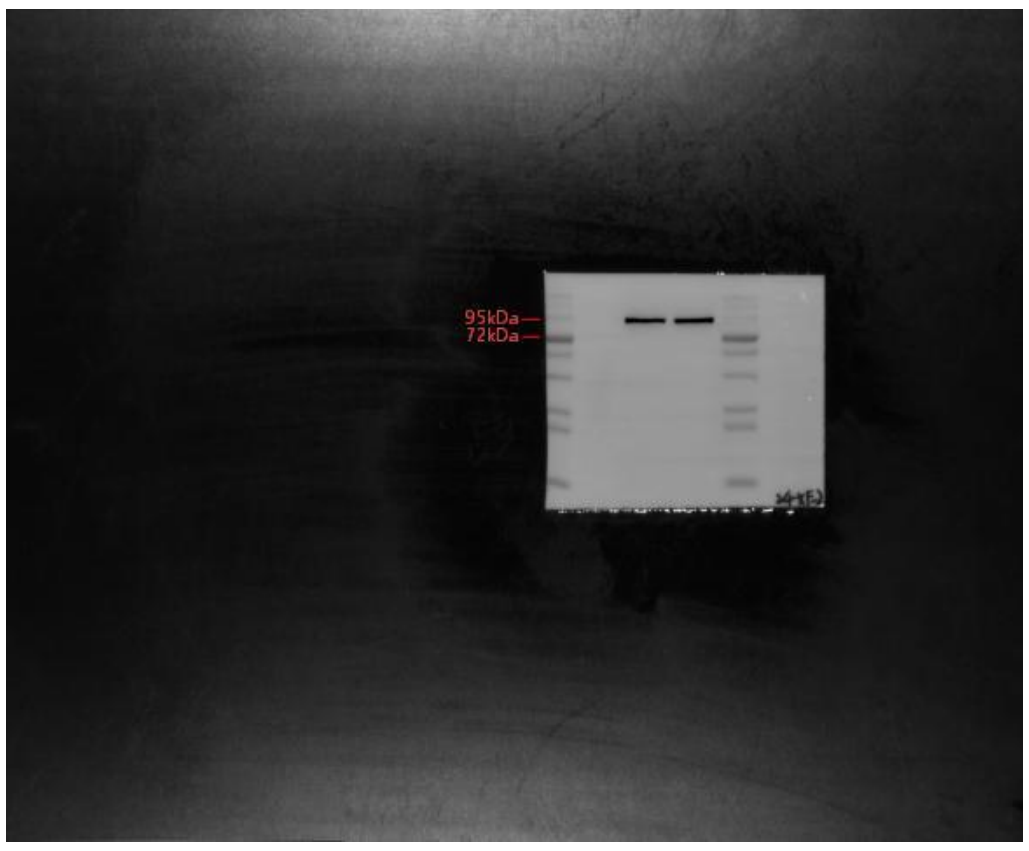

Flag-PLOD1

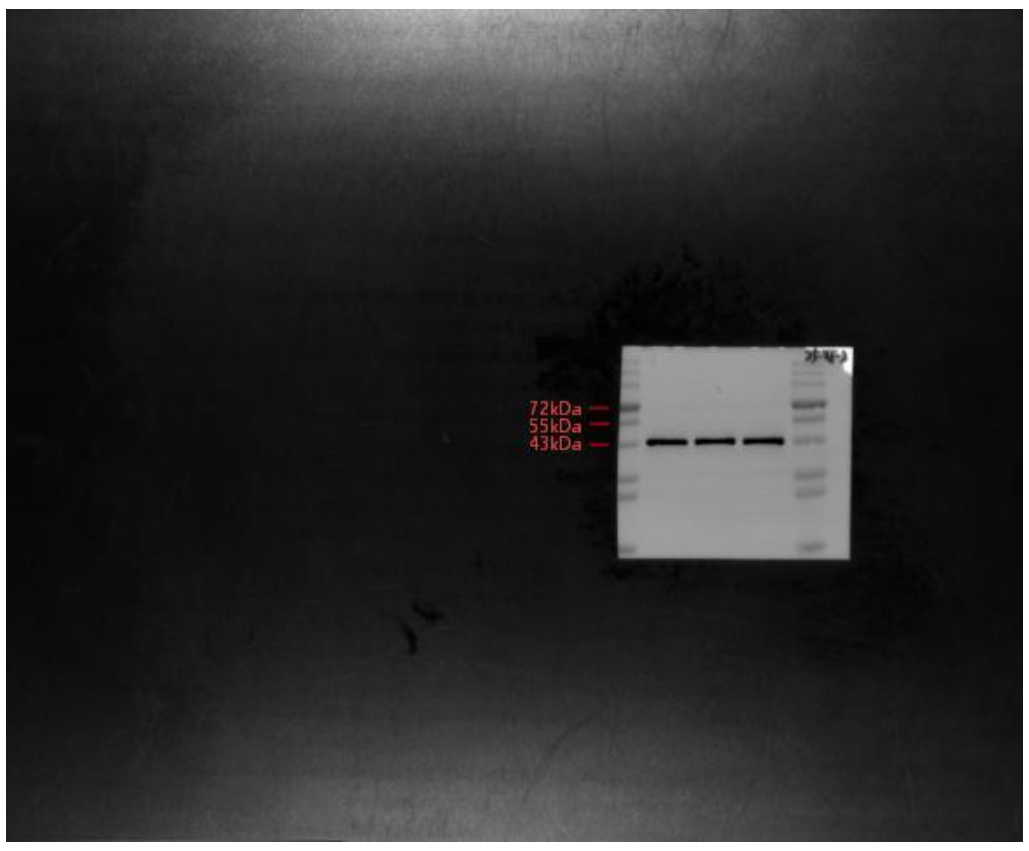

ACTIN

FIG.3J

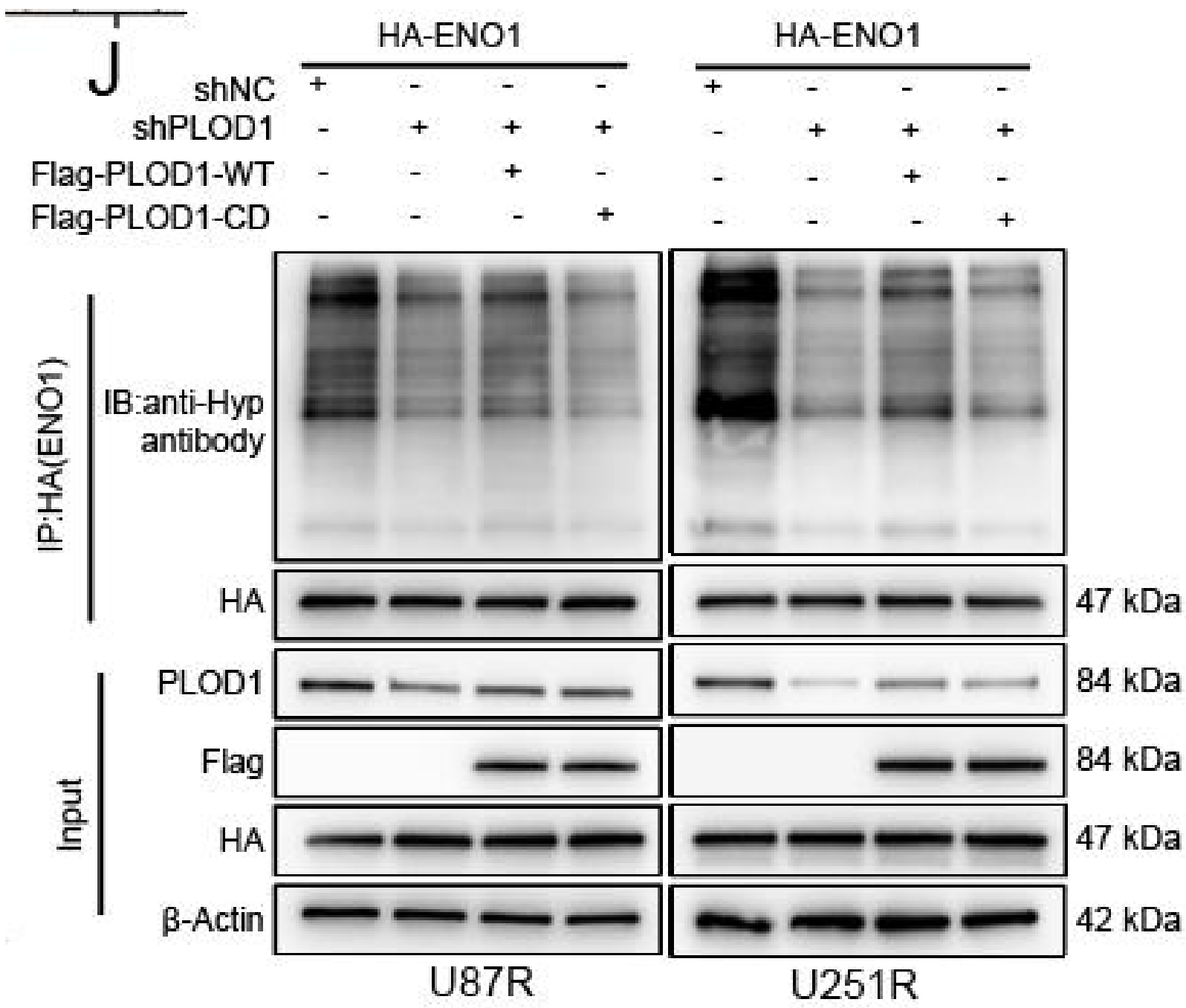

U87R

IP

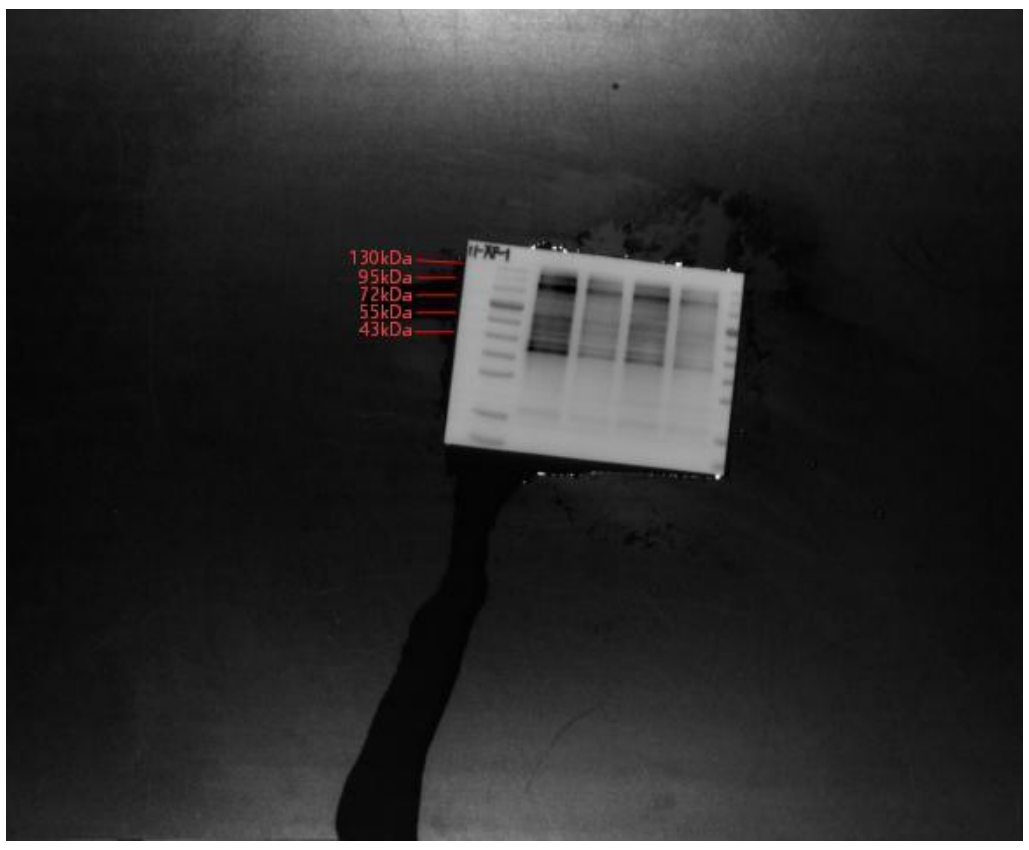

HYP

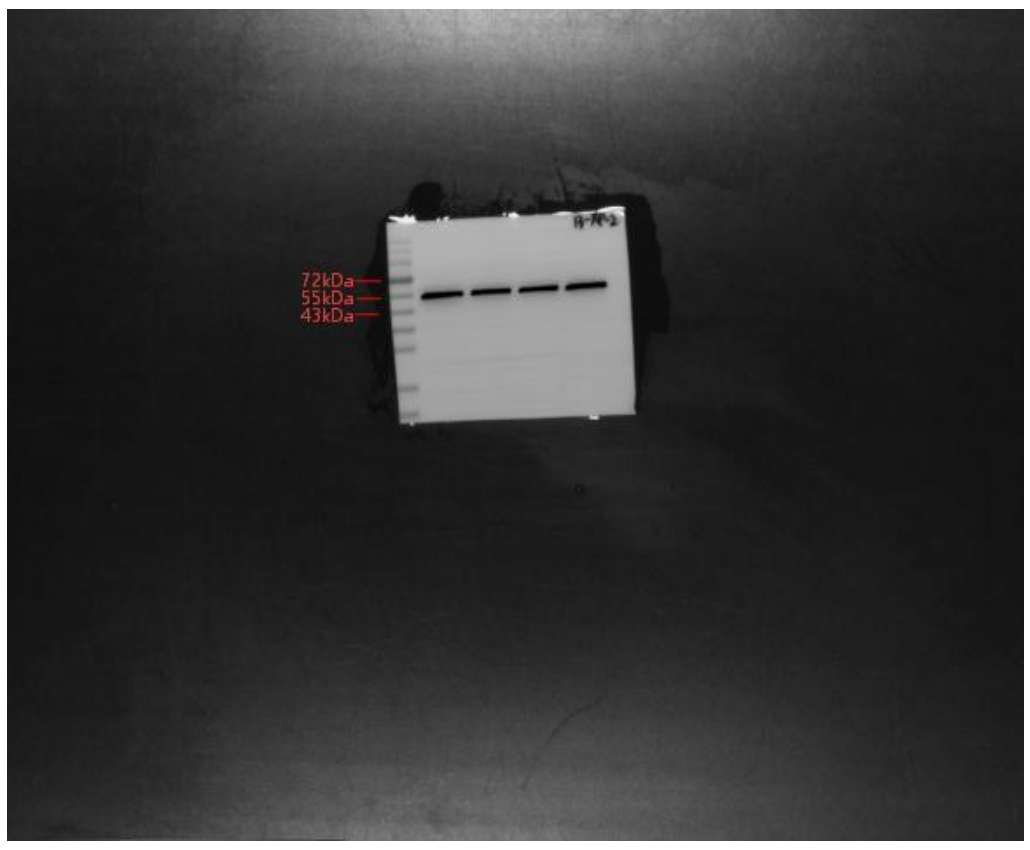

HA

INPUT

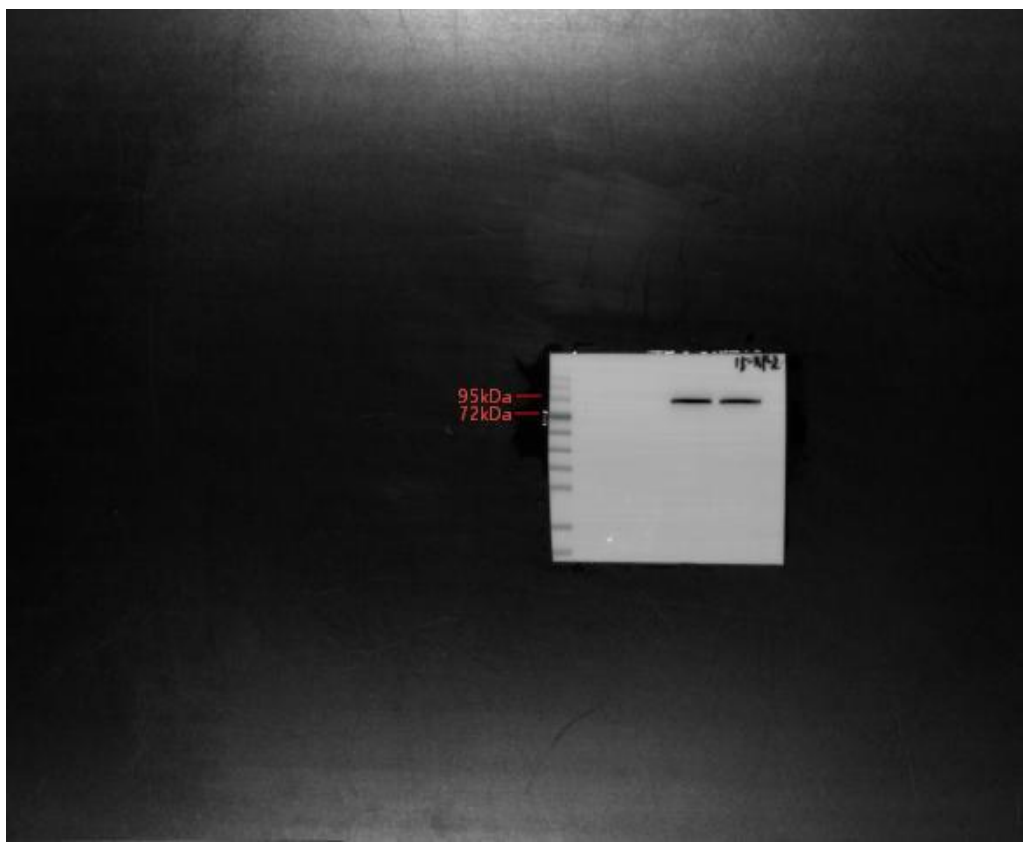

Flag

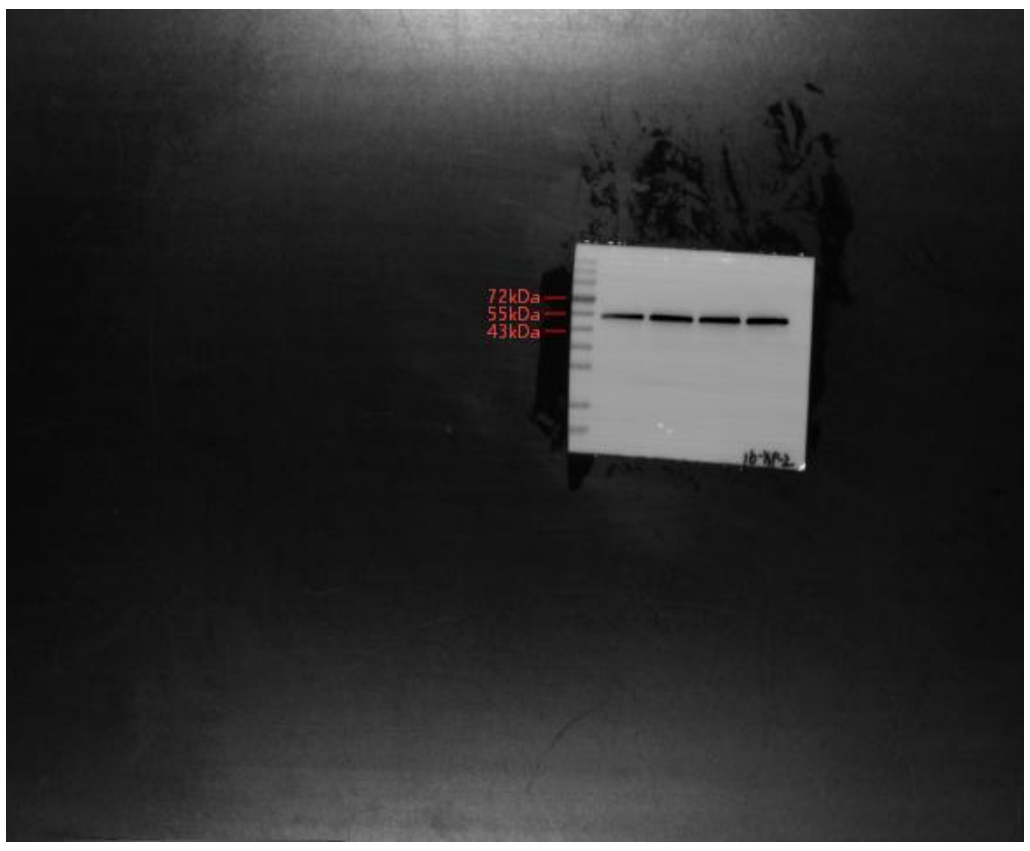

HA

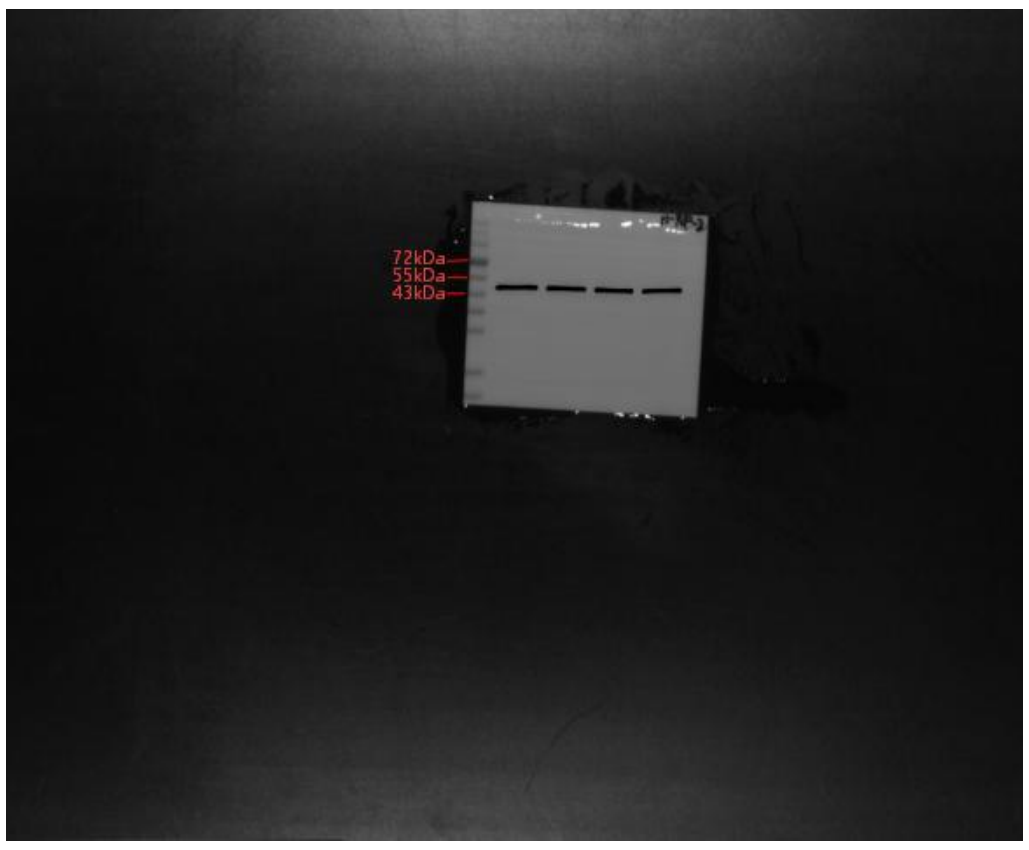

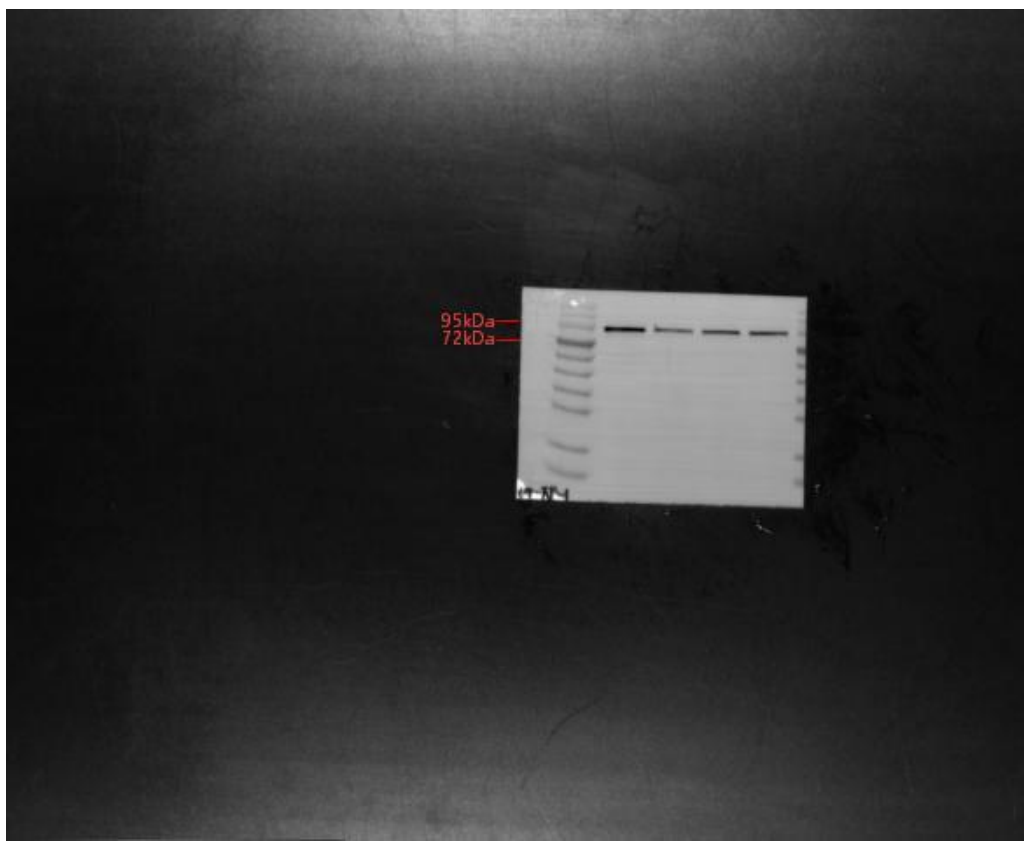

ACTIN PLOD1

U251R

IP

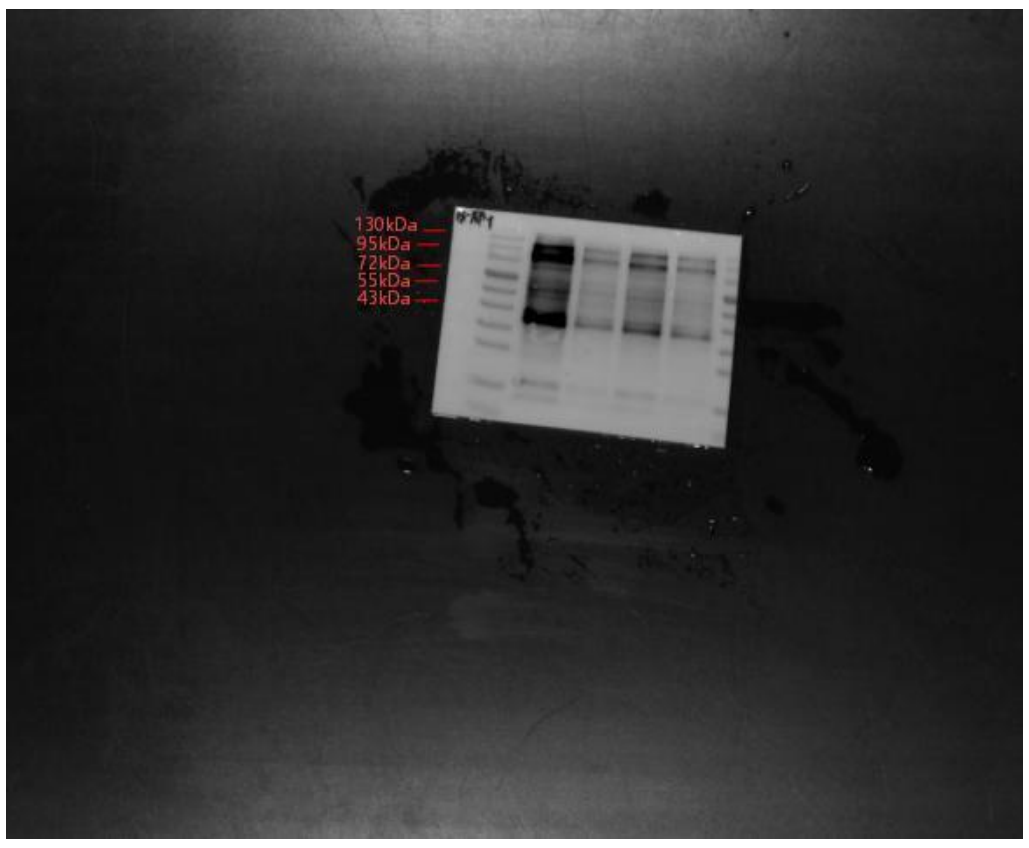

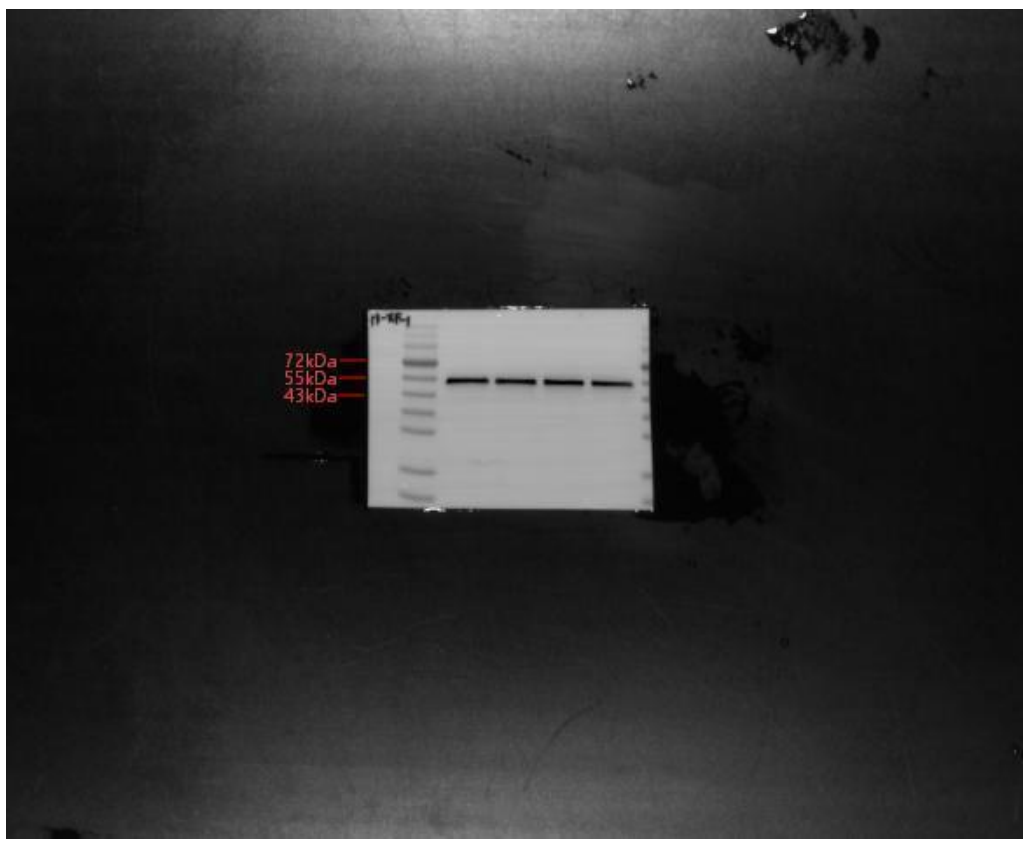

HYP HA

INPUT

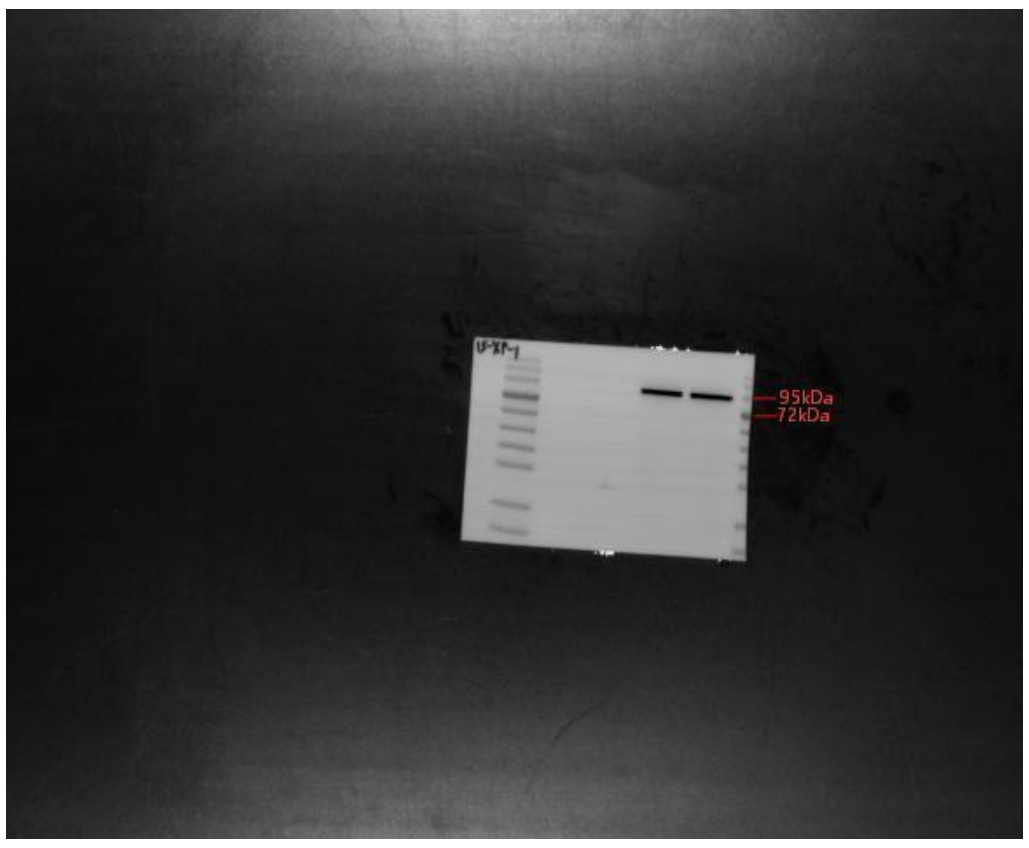

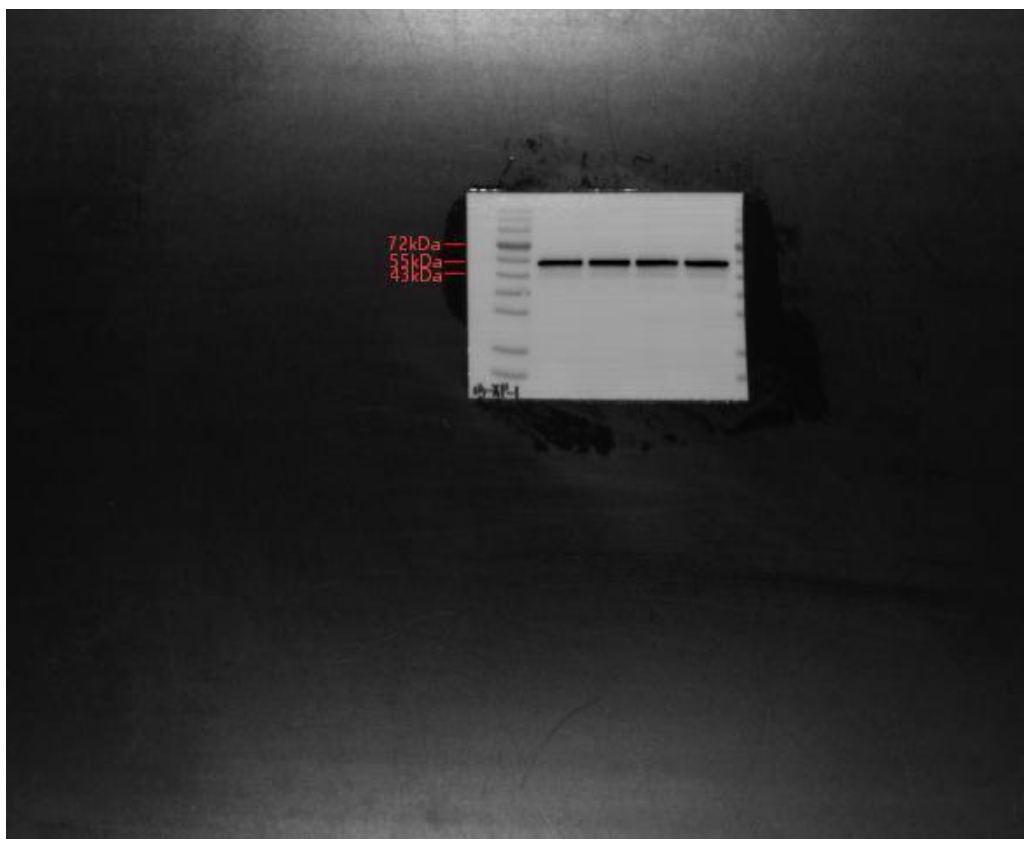

Flag HA

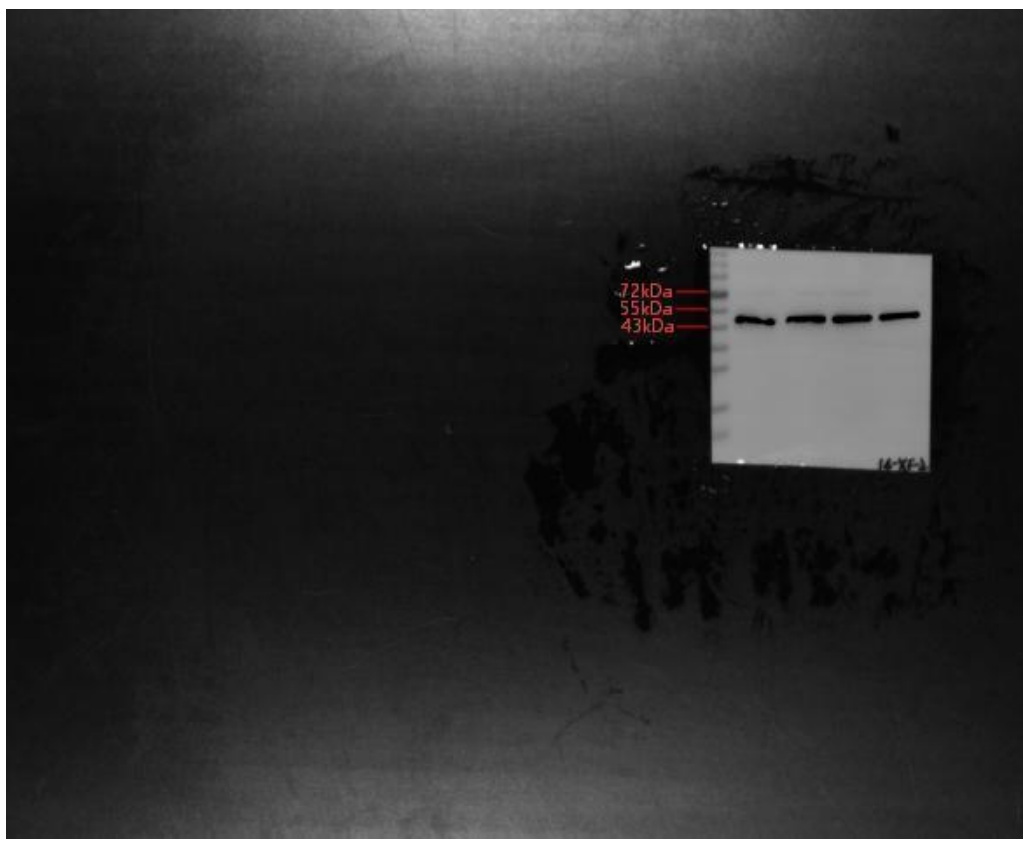

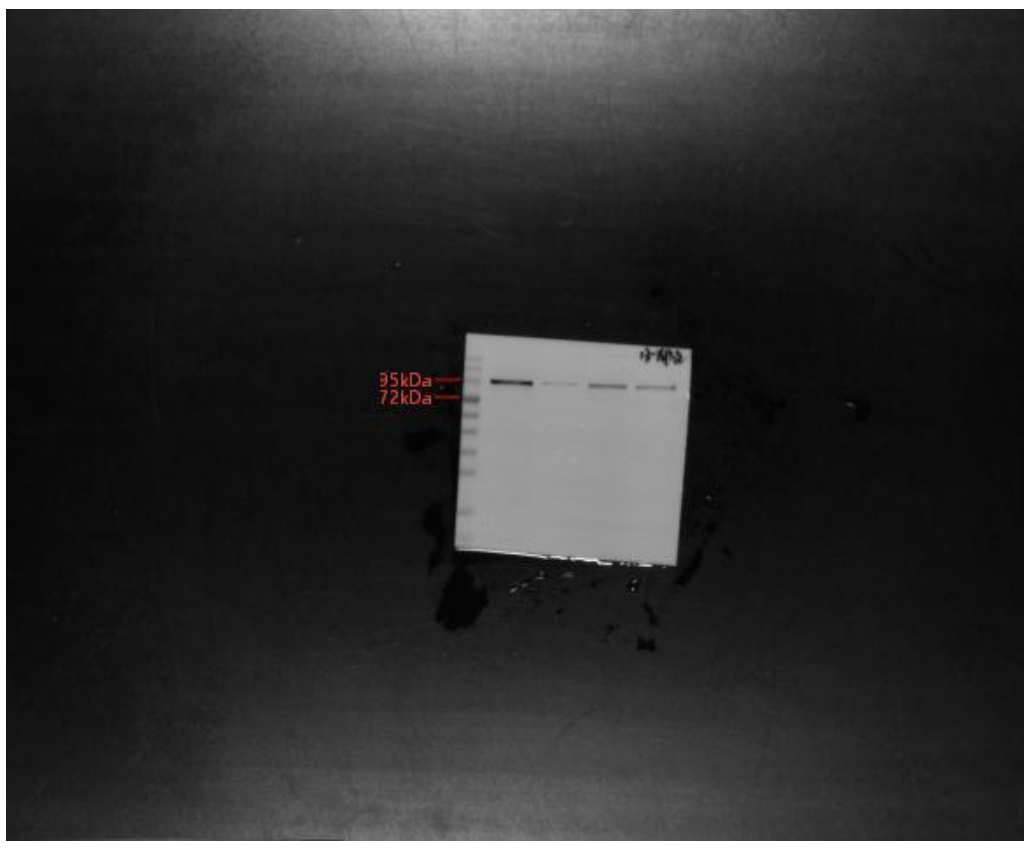

ACTIN

PLOD1

FIG.3K

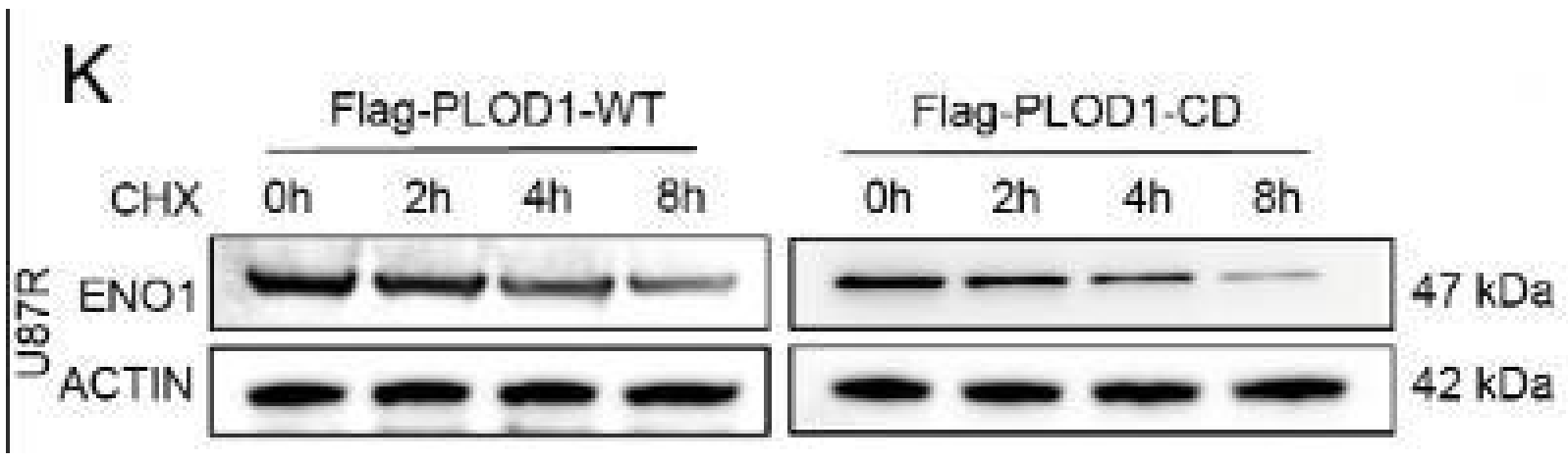

U87R

Flag-PLOD1-WT

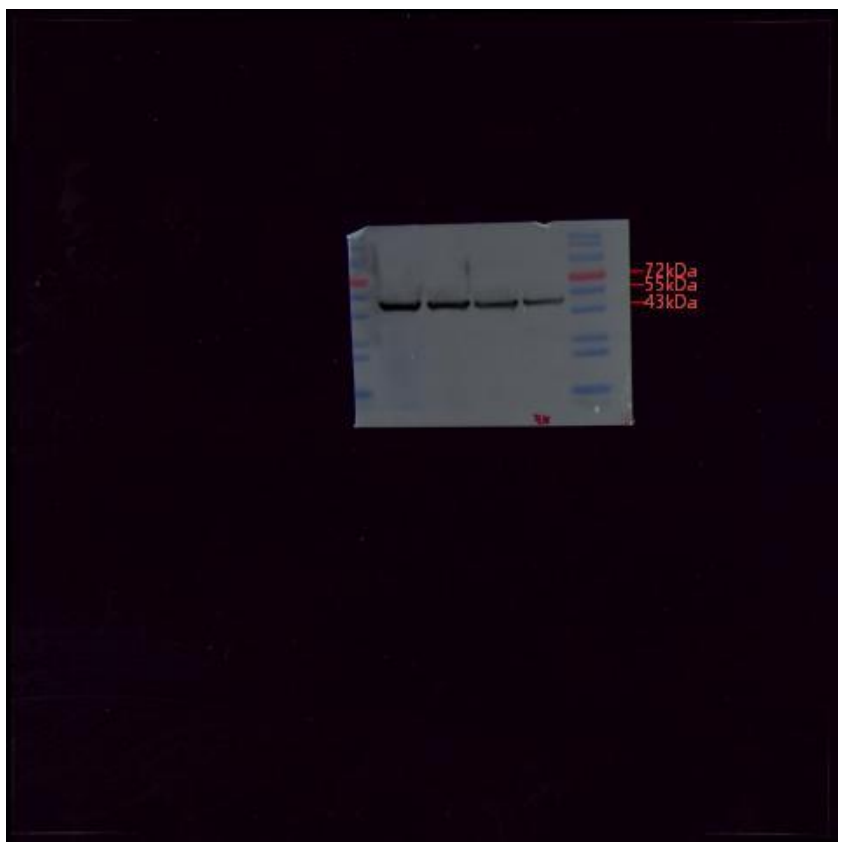

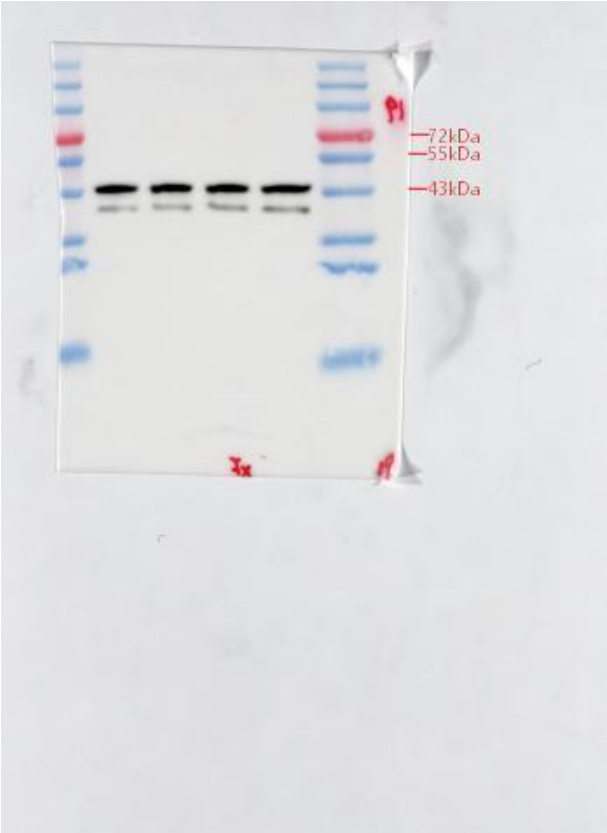

ENO1

ACTIN

Flag-PLOD1-CD

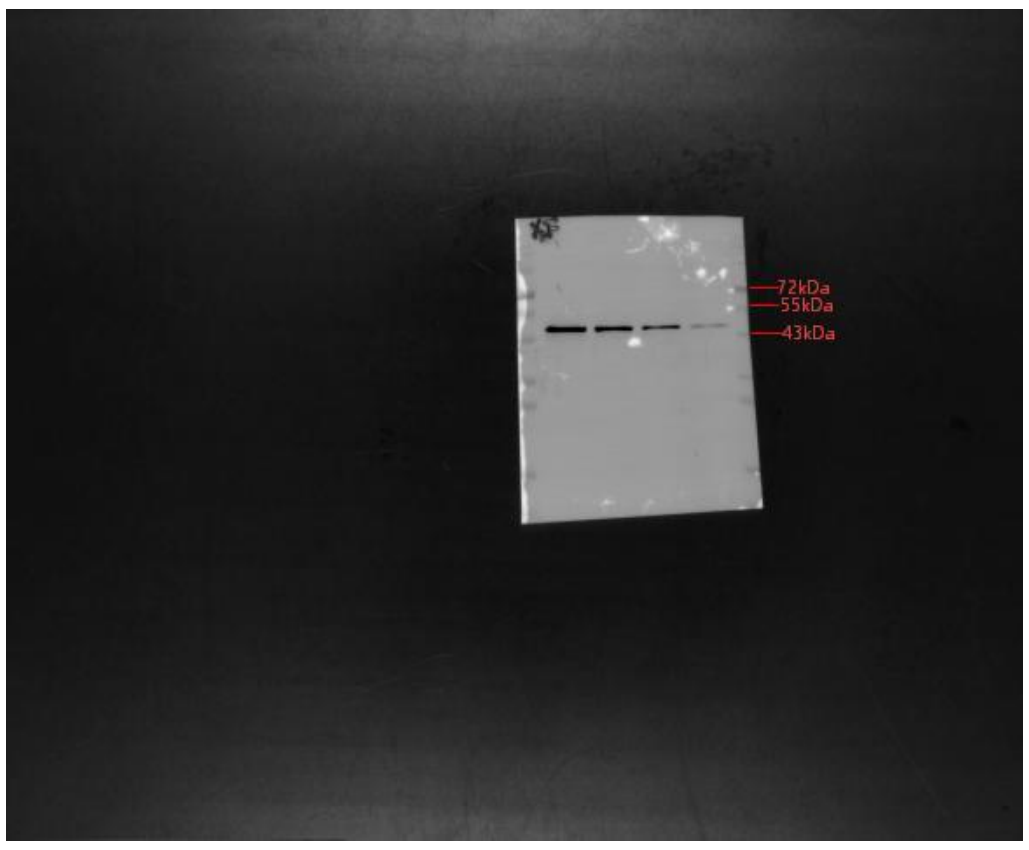

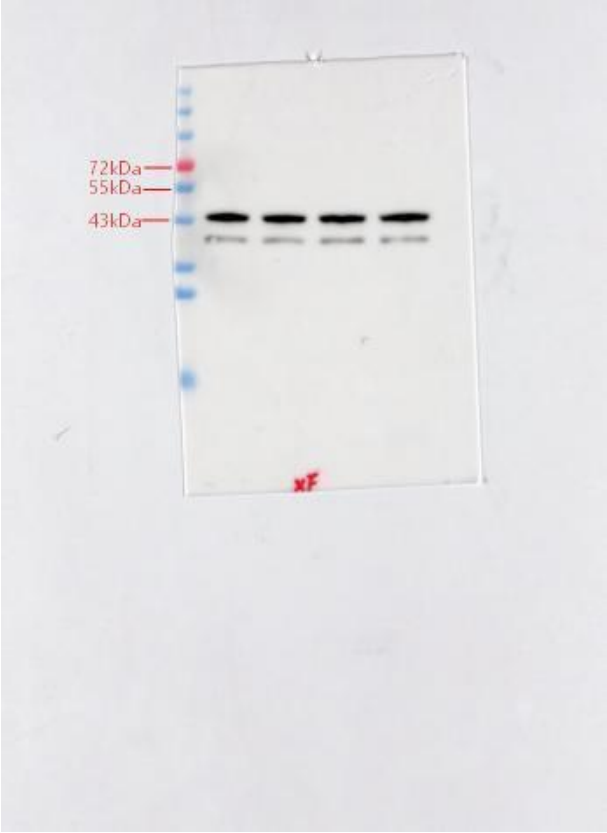

ENO1 ACTIN

U251R

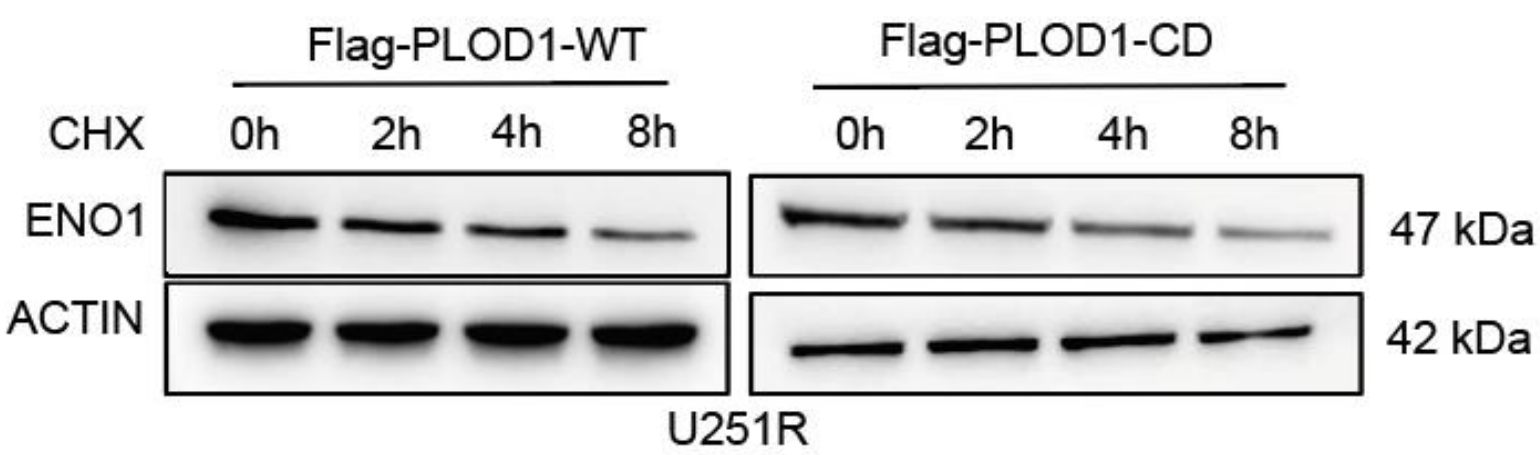

Flag-PLOD1-WT

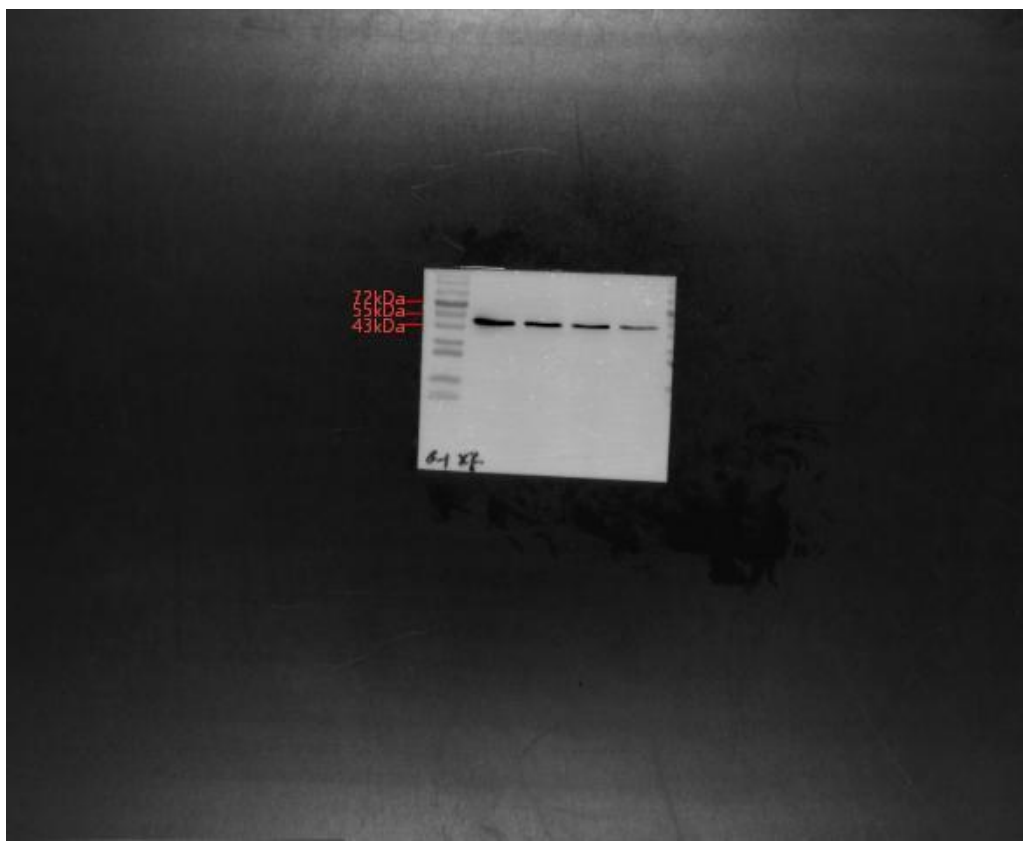

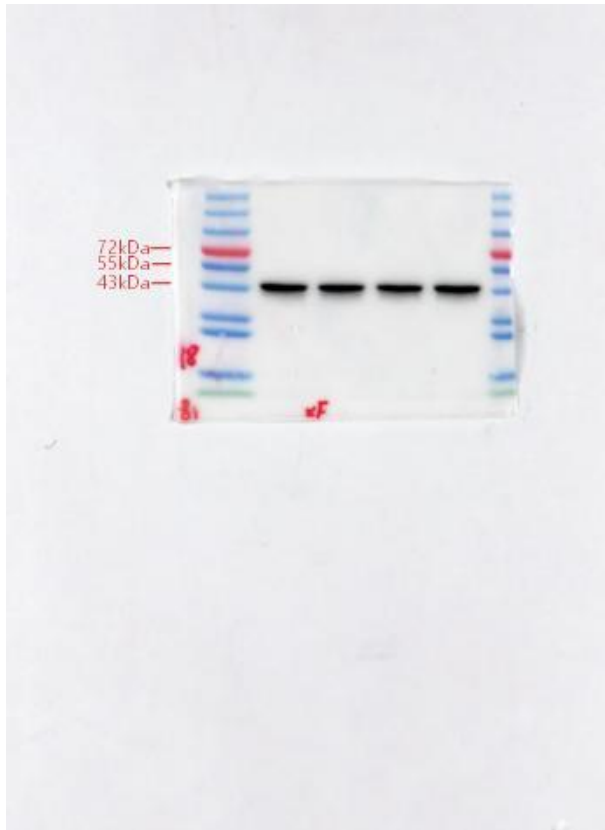

ENO1 ACTIN

Flag-PLOD1-CD

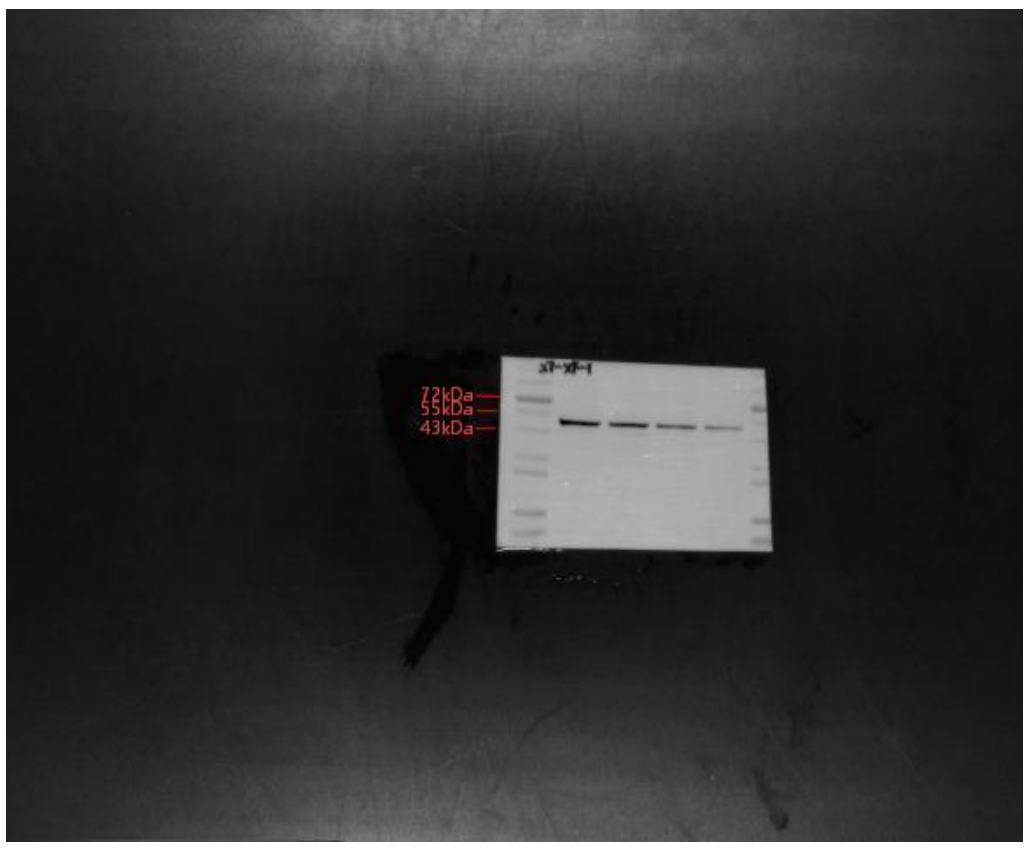

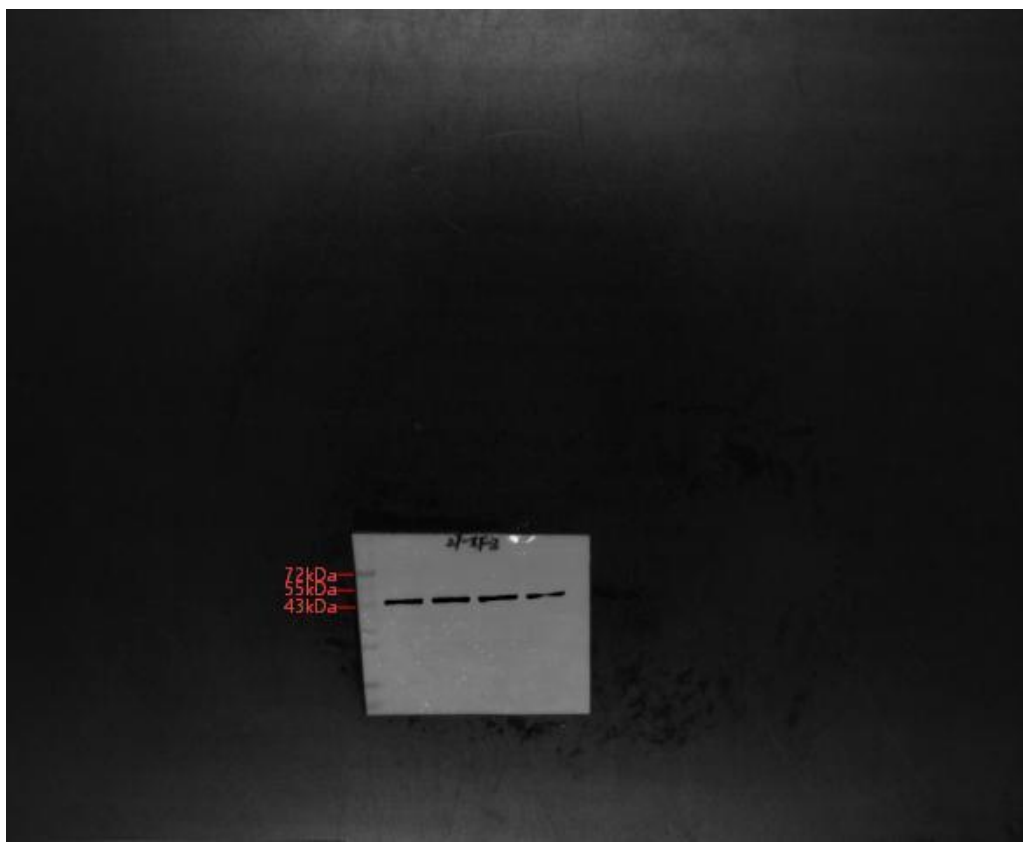

ENO1 ACTIN

FIG.4A

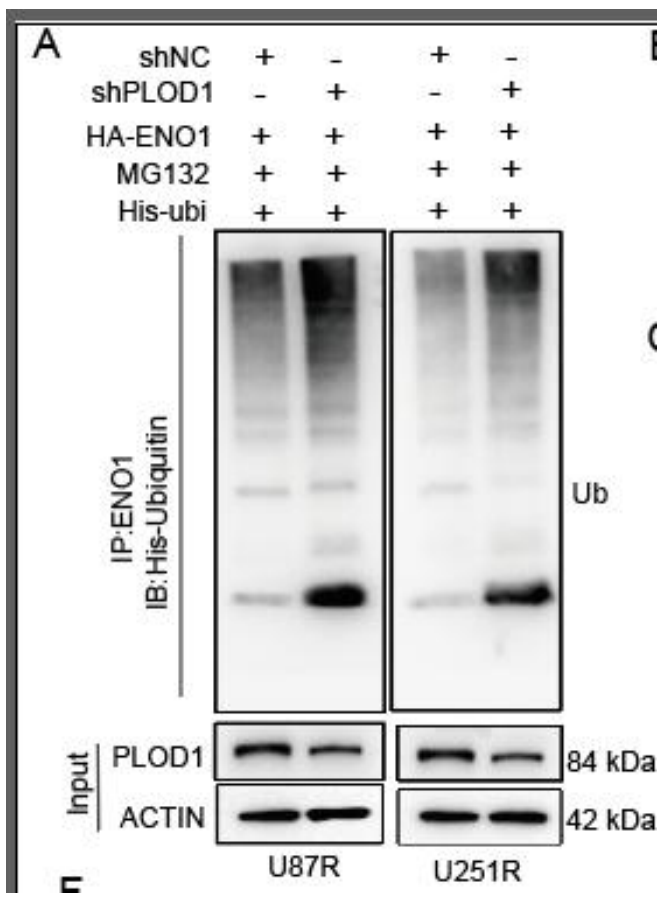

U87R

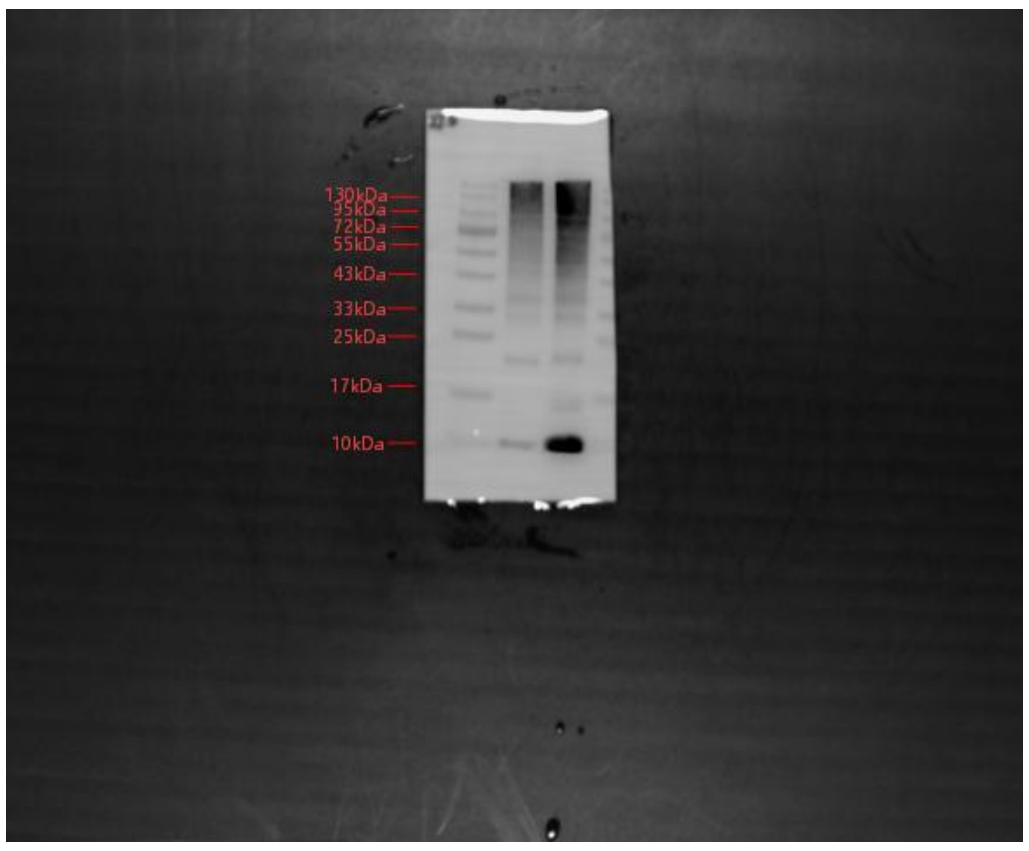

Ub

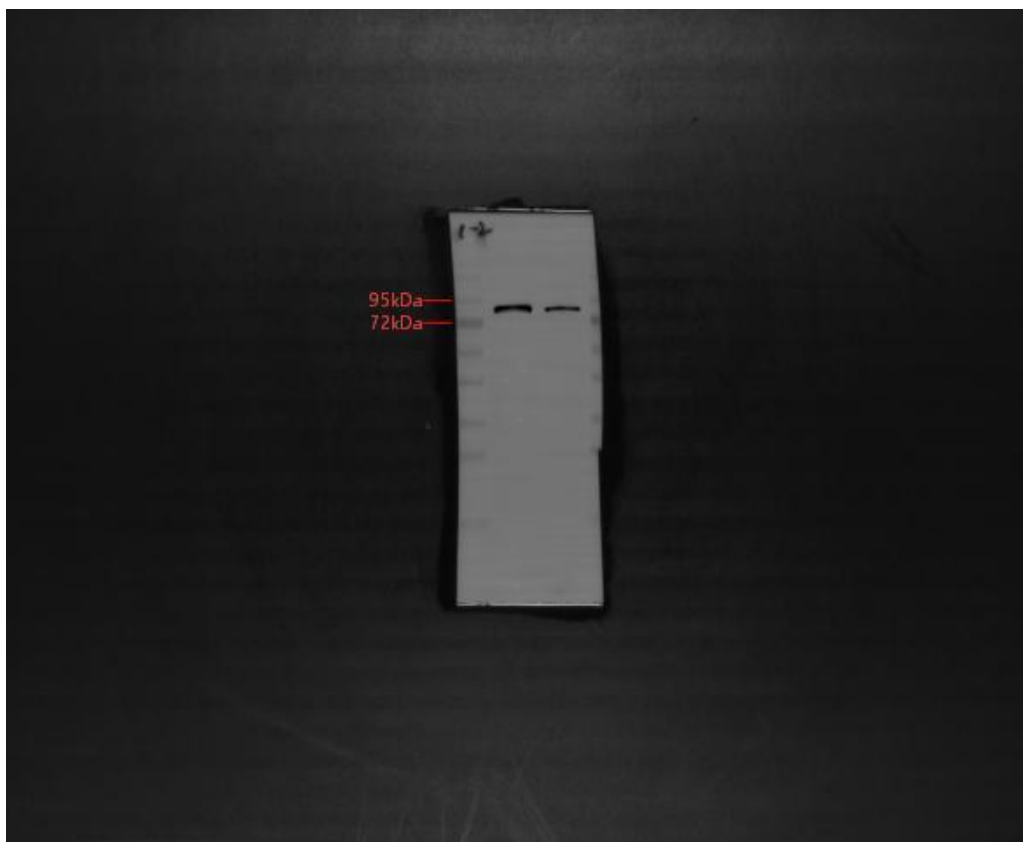

PLOD1

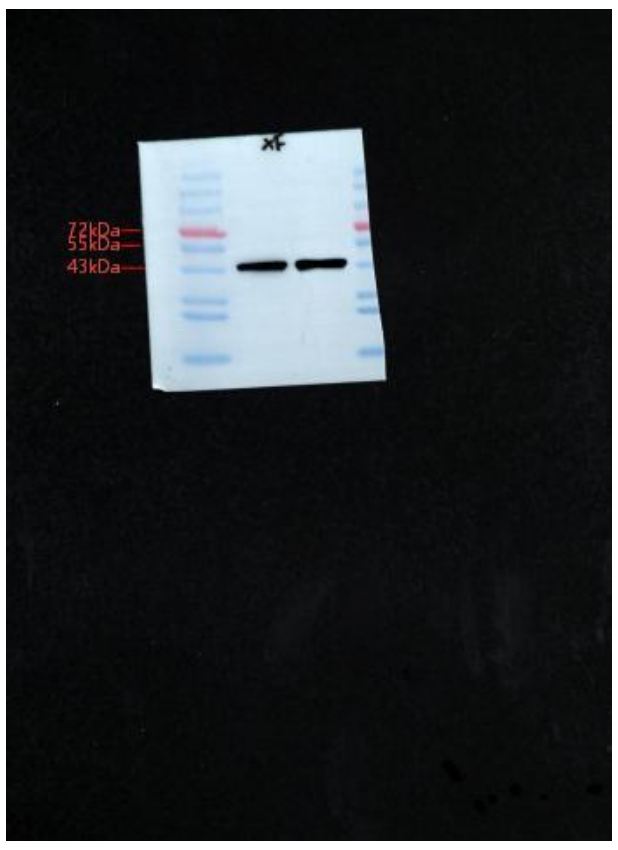

ACTIN

U251R

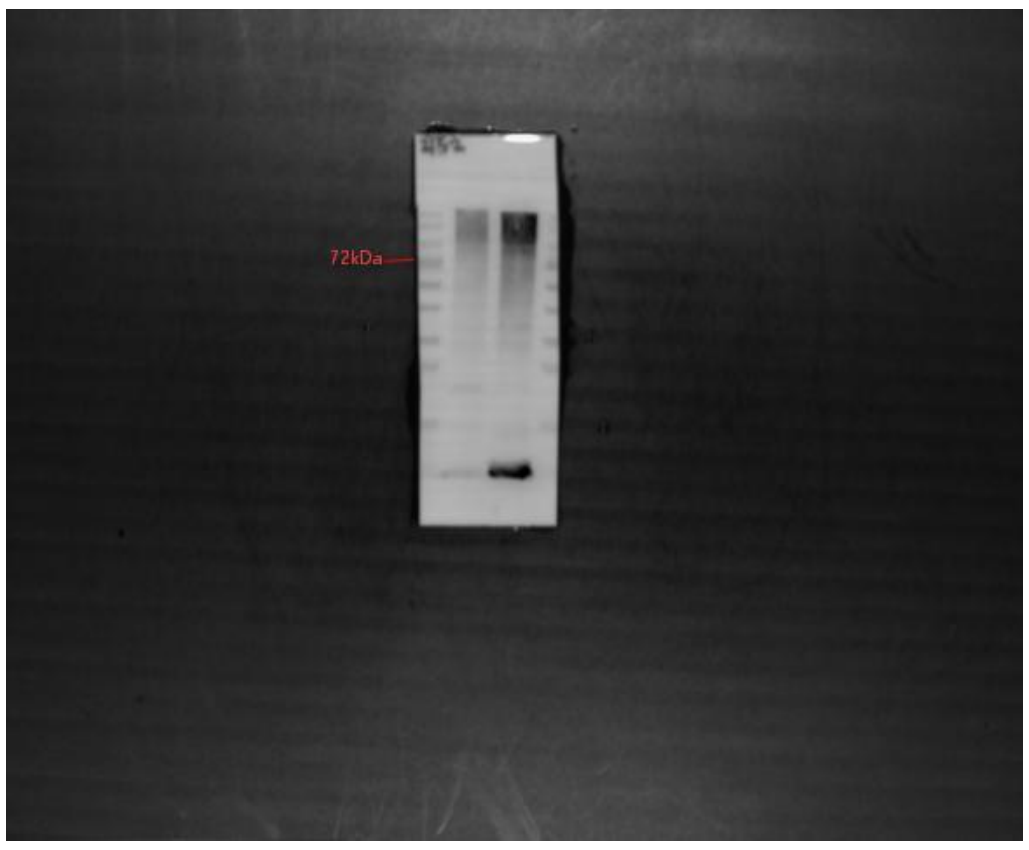

Ub

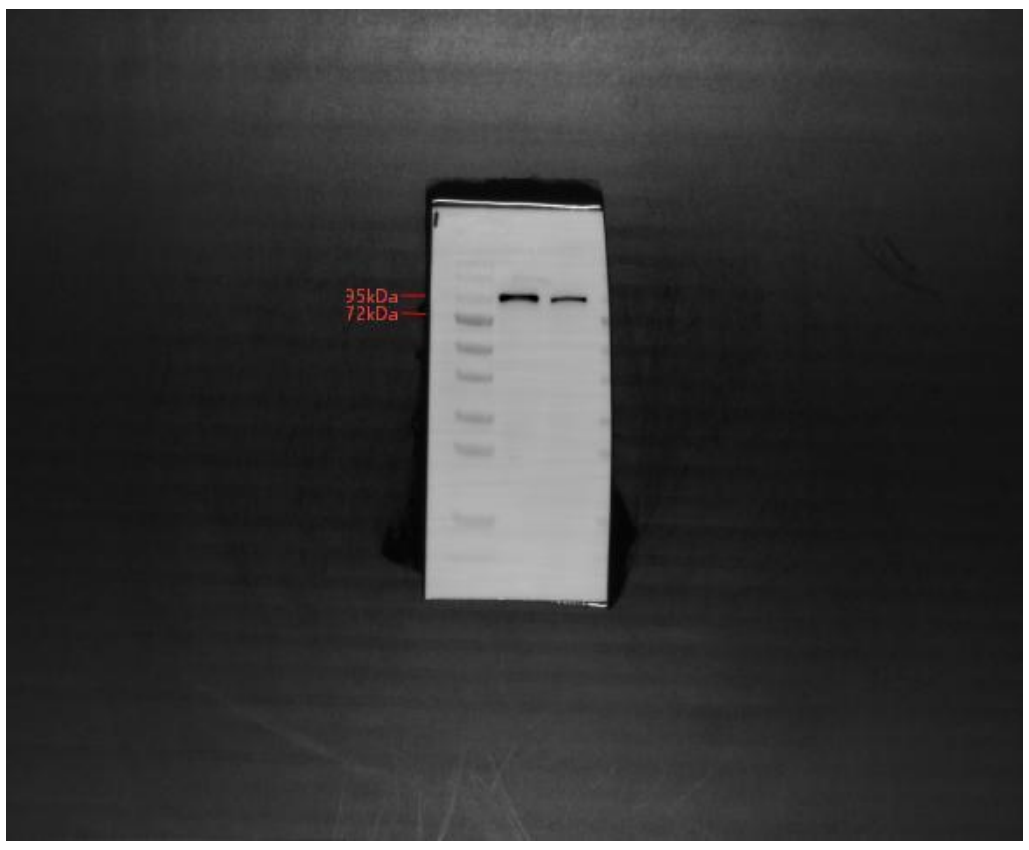

PLOD1

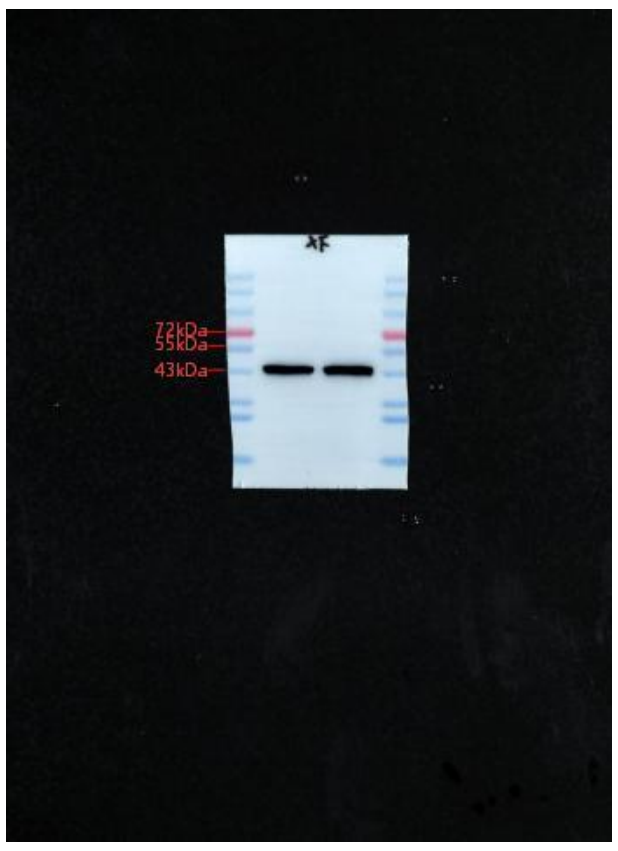

ACTIN

FIG.4B

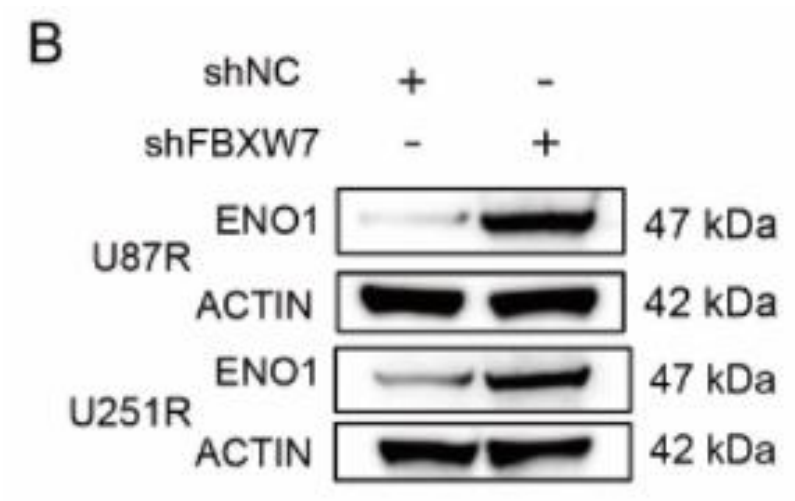

U87R

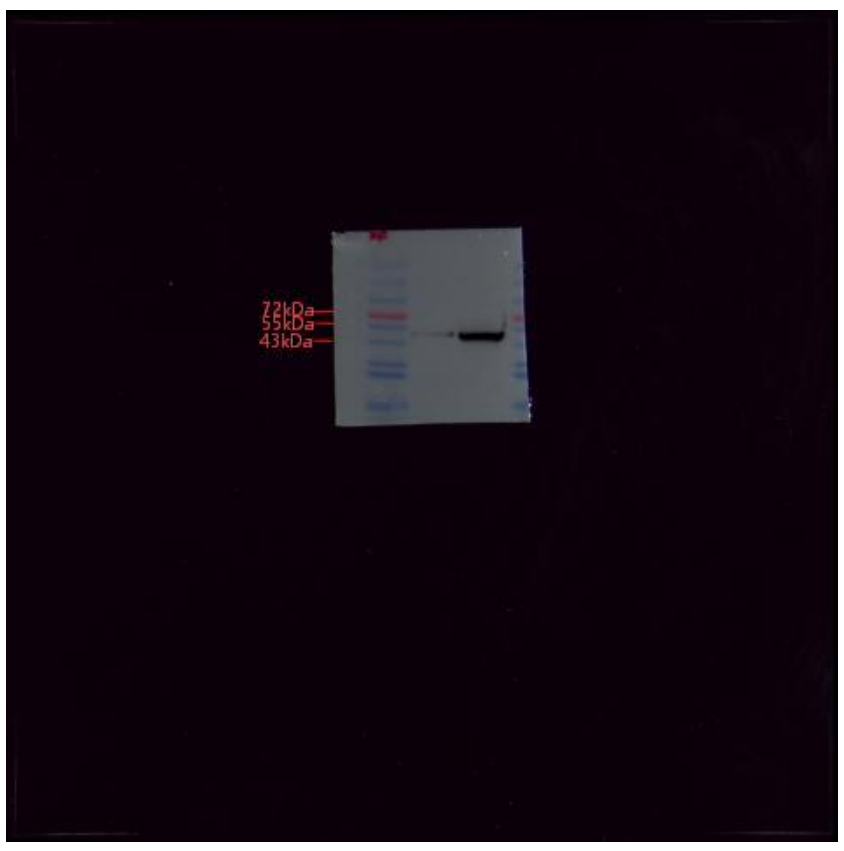

ENO1

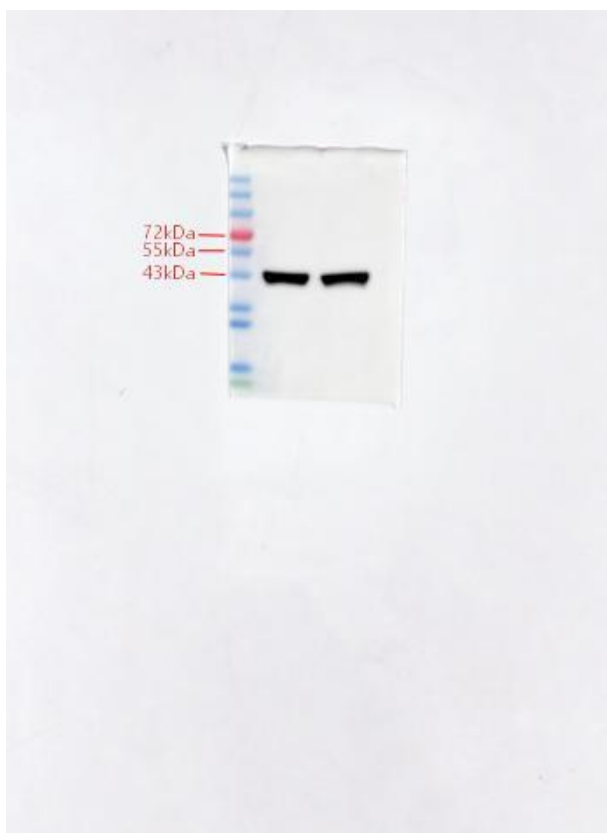

ACTIN

U251R

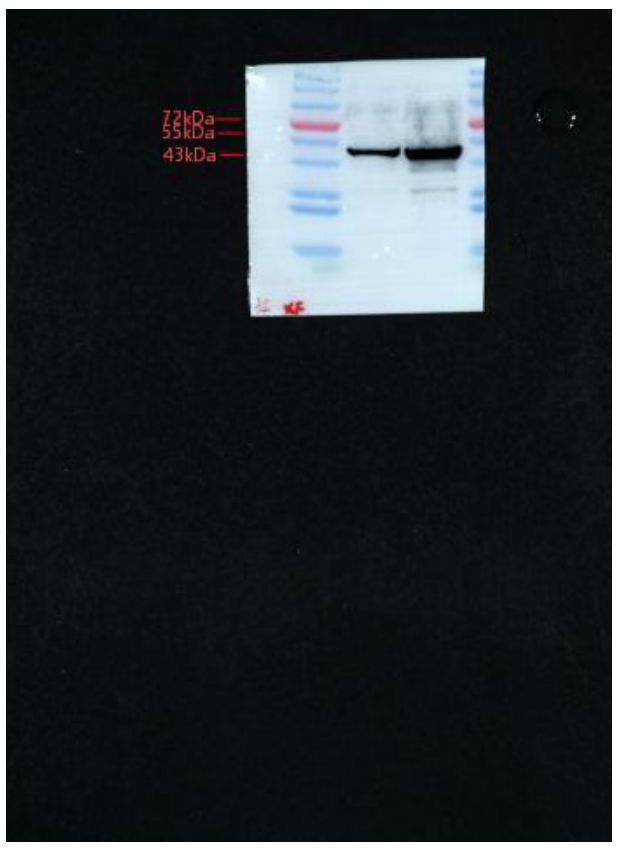

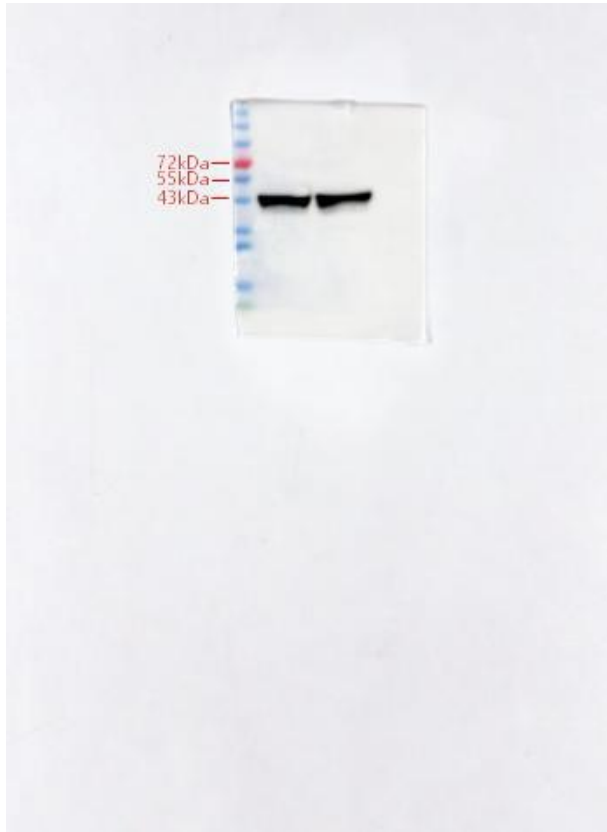

ENO1 ACTIN

FIG.4C

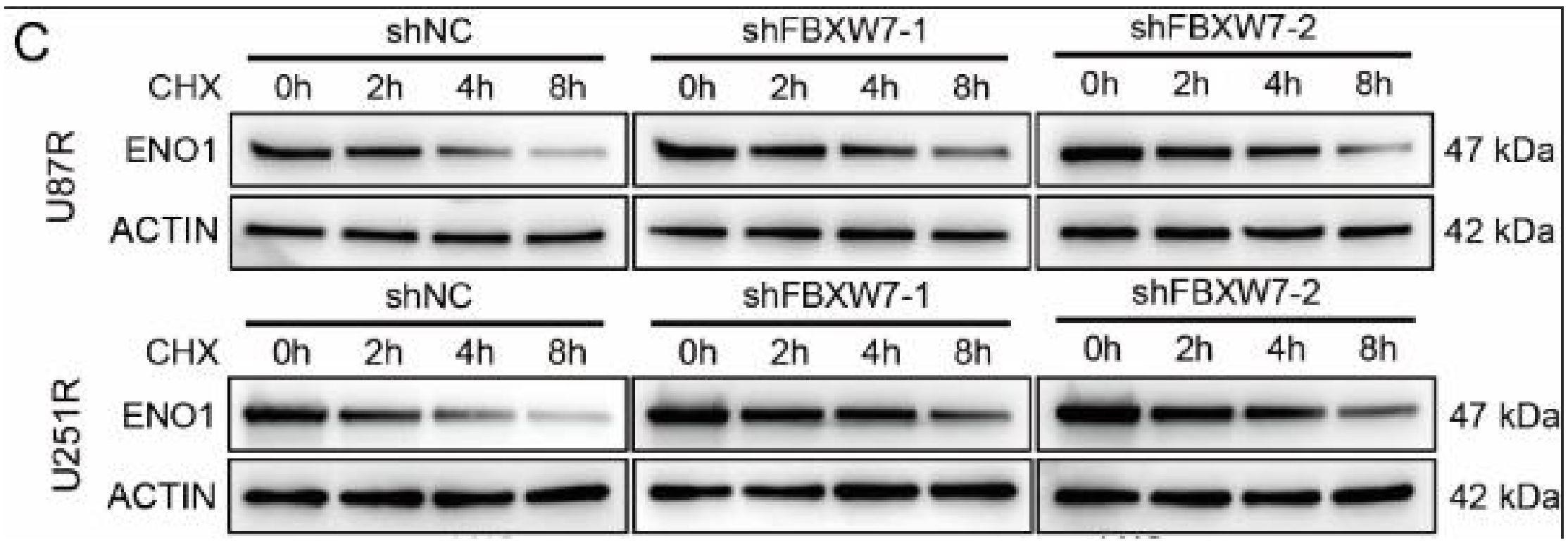

U87R

ShNC

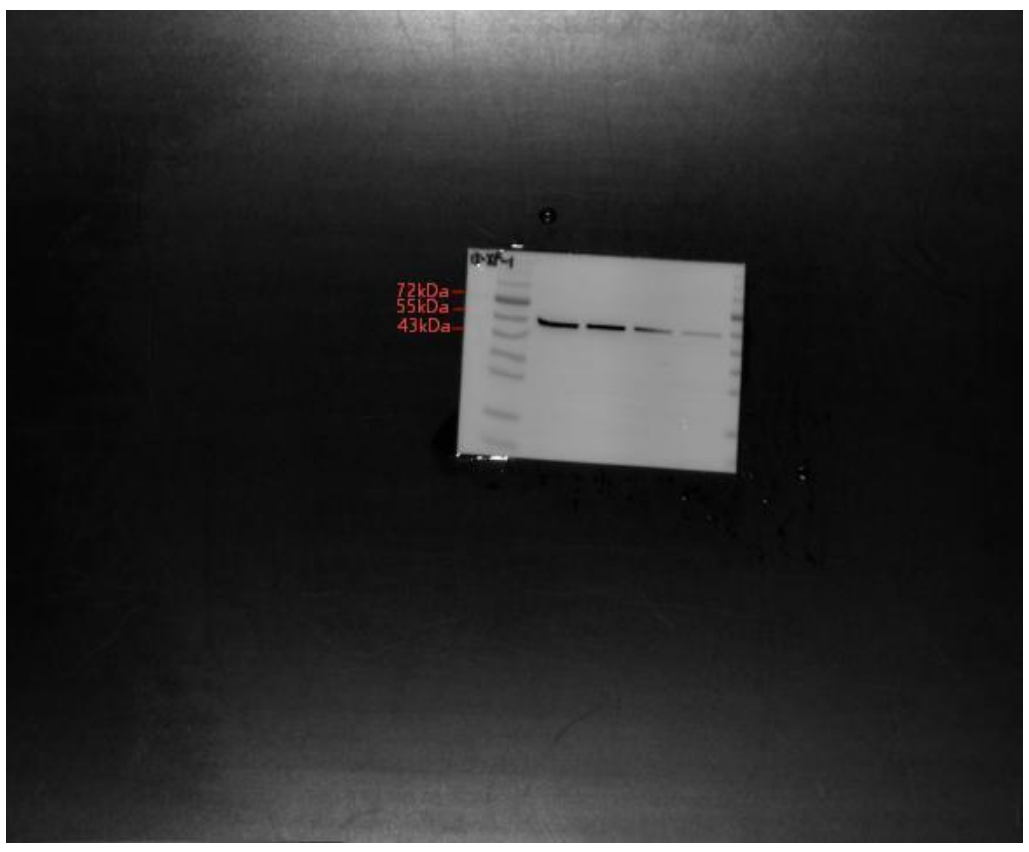

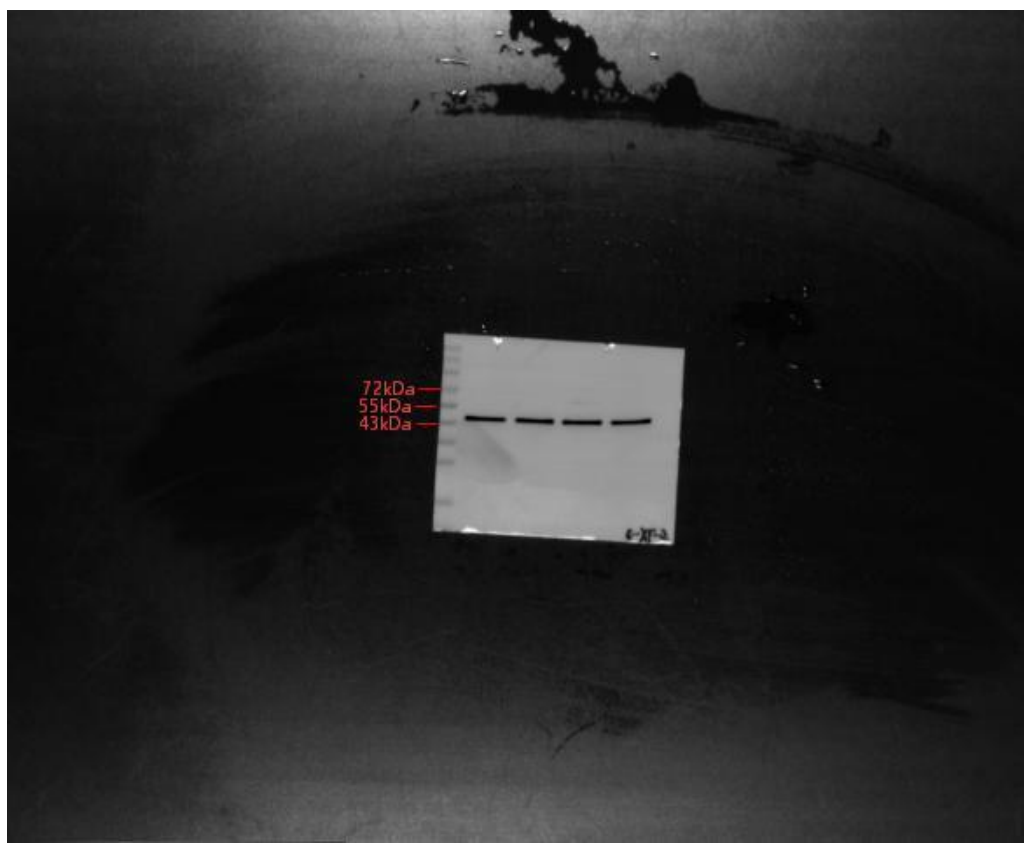

ENO1 ACTIN

ShFBXW7-1

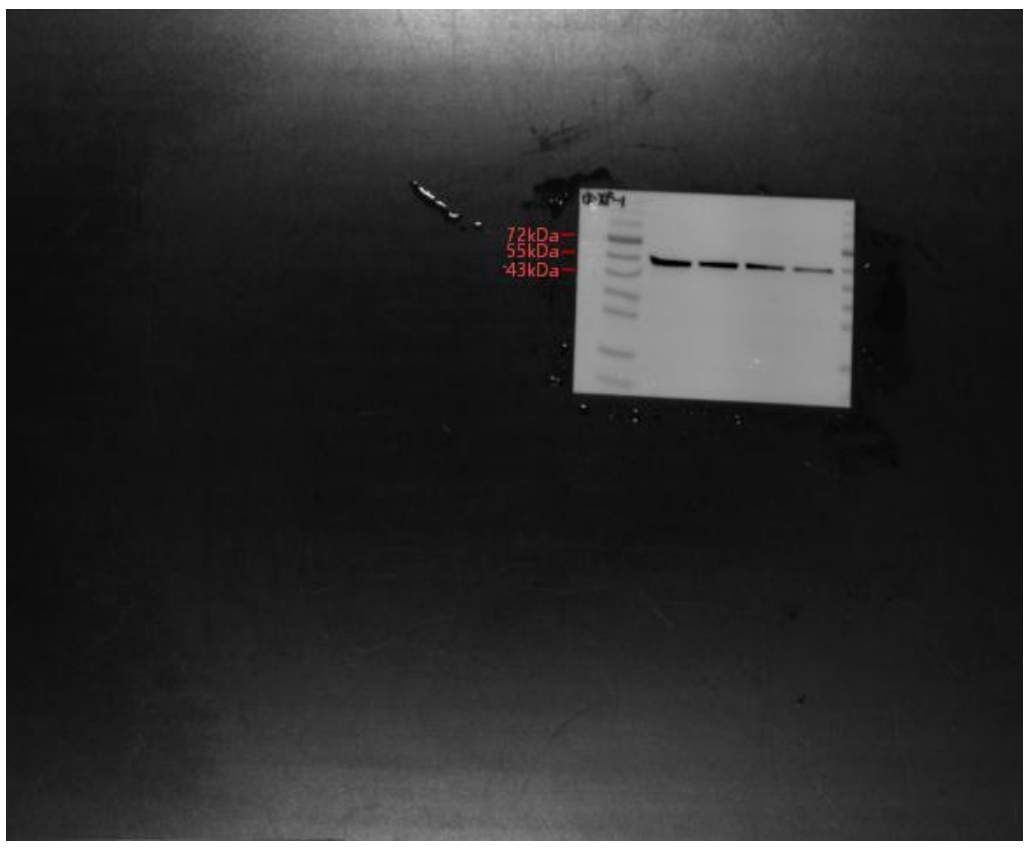

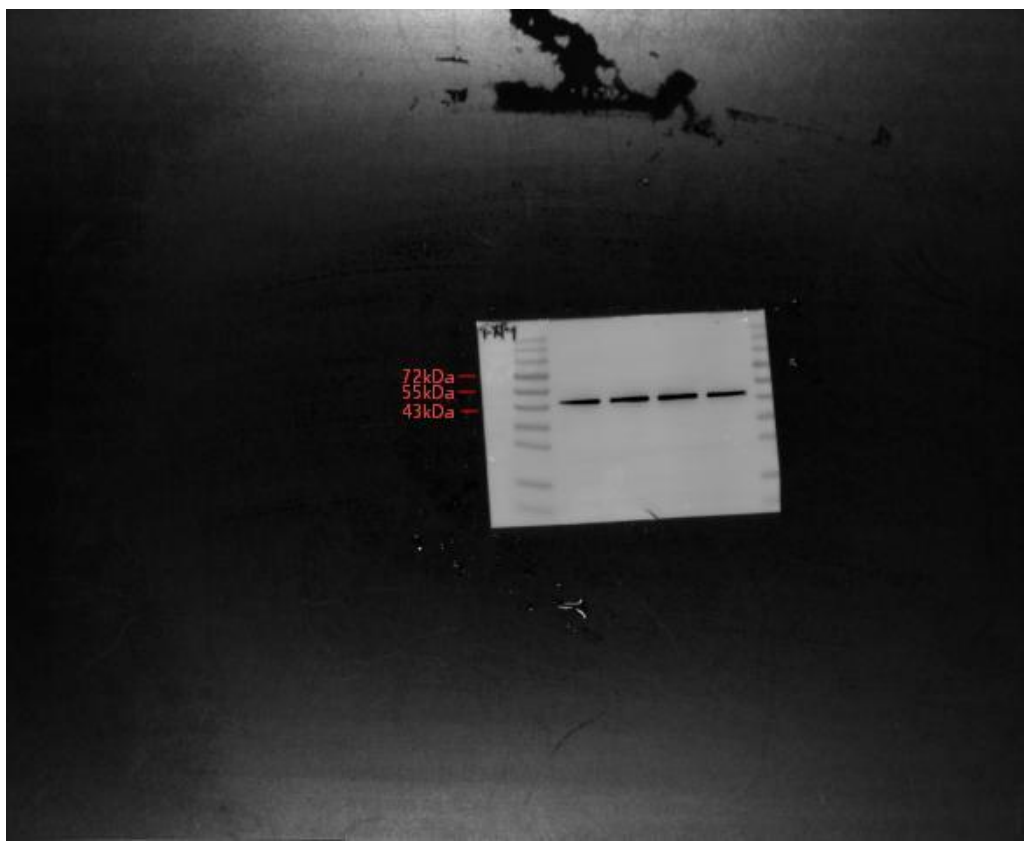

ENO1 ACTIN

ShFBXW7-2

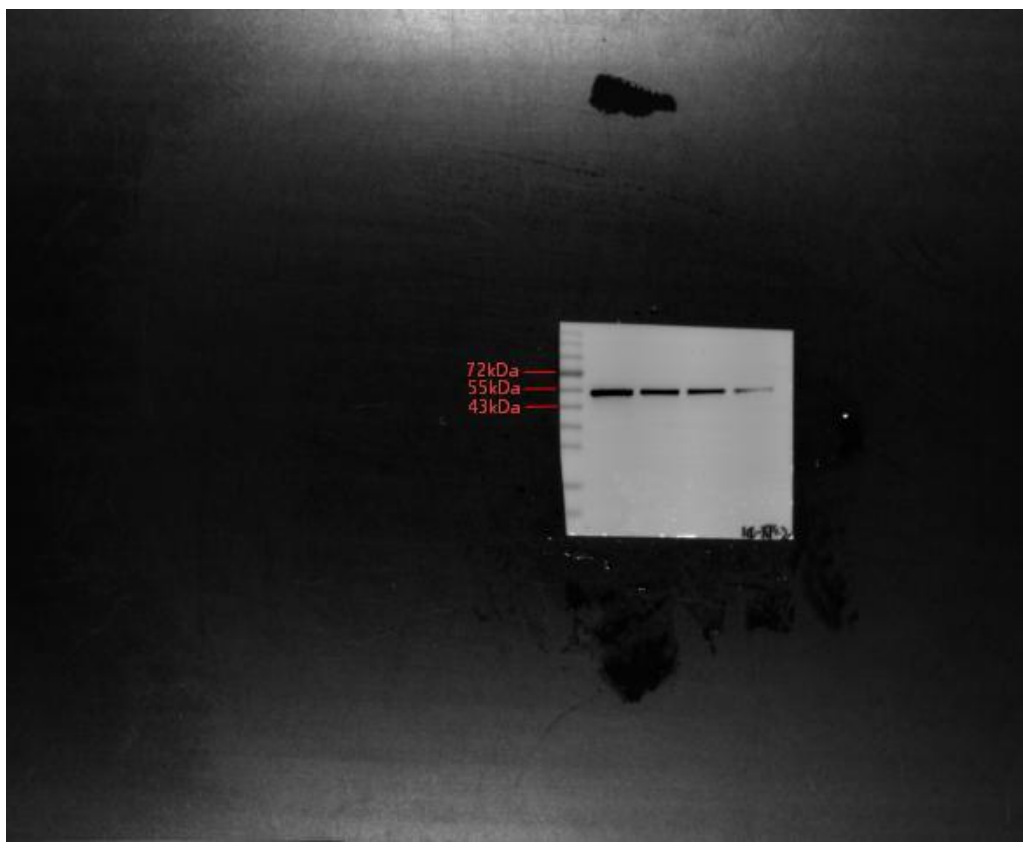

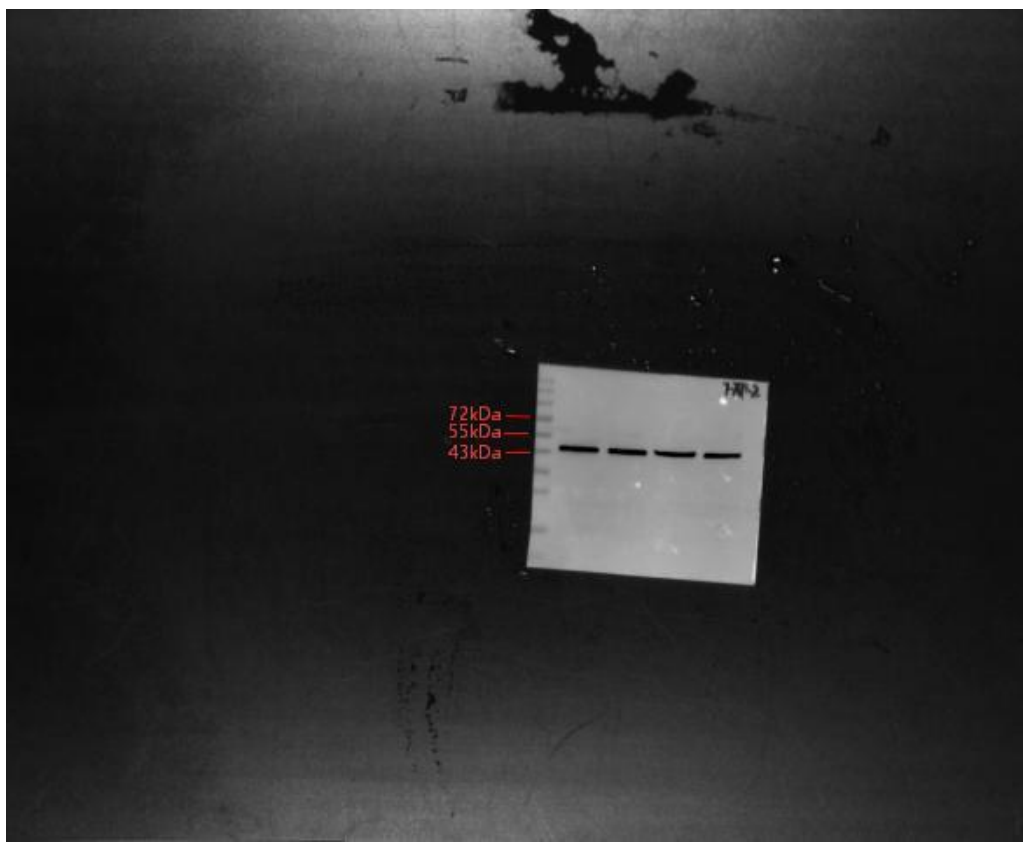

ENO1 ACTIN

U251R

ShNC

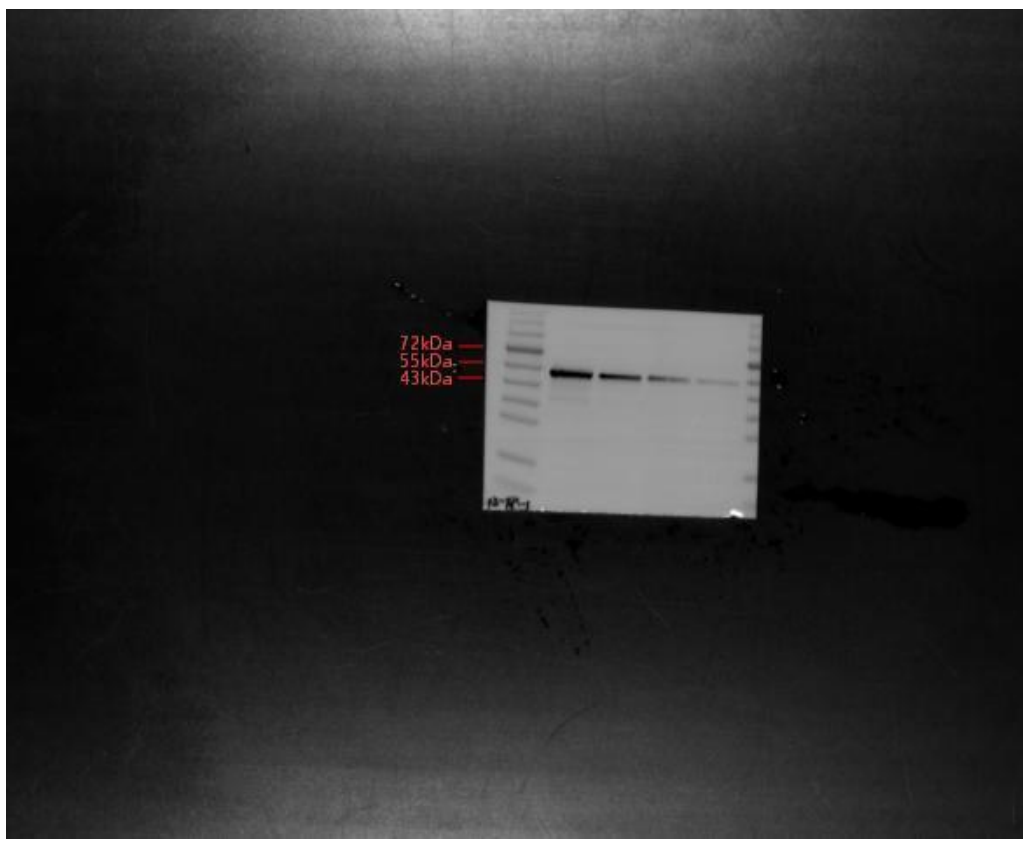

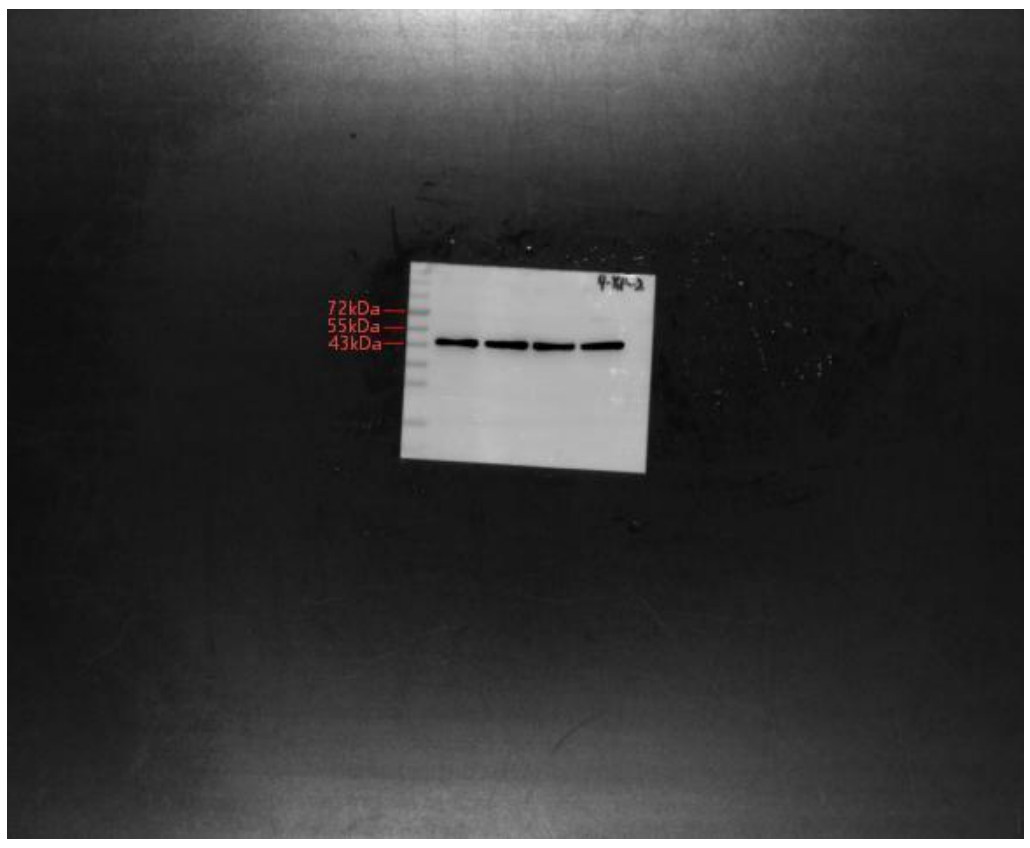

ENO1 ACTIN

ShFBXW7-1

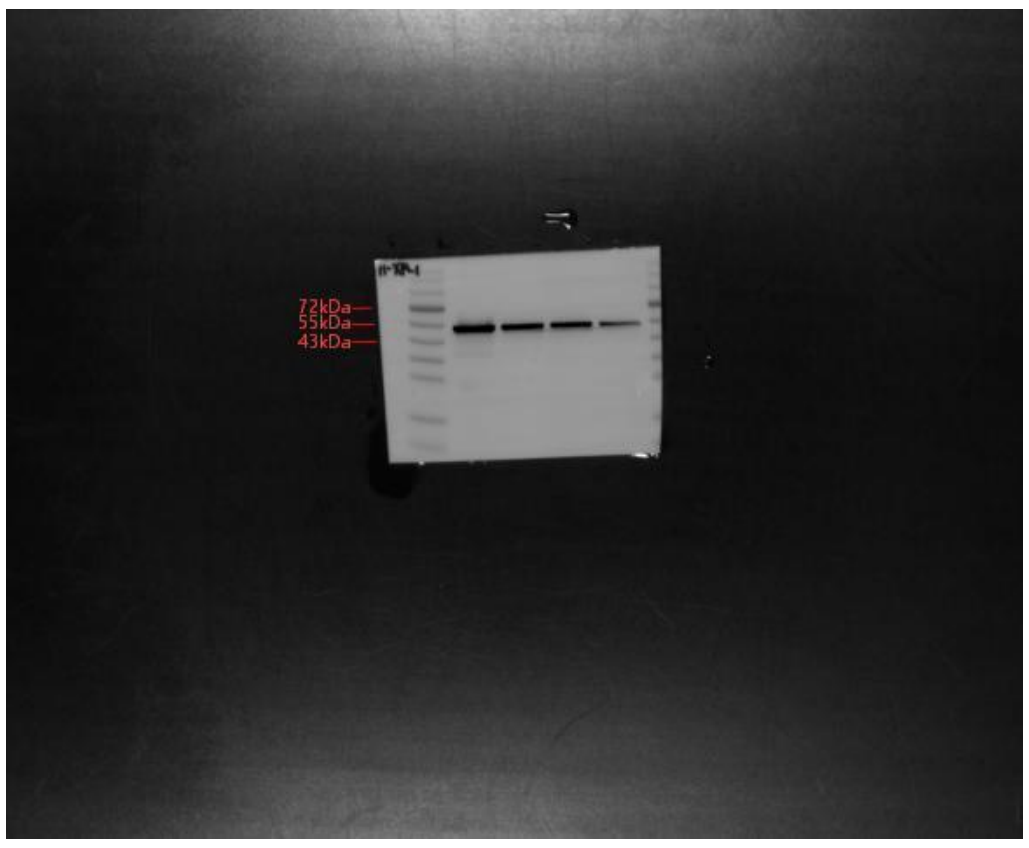

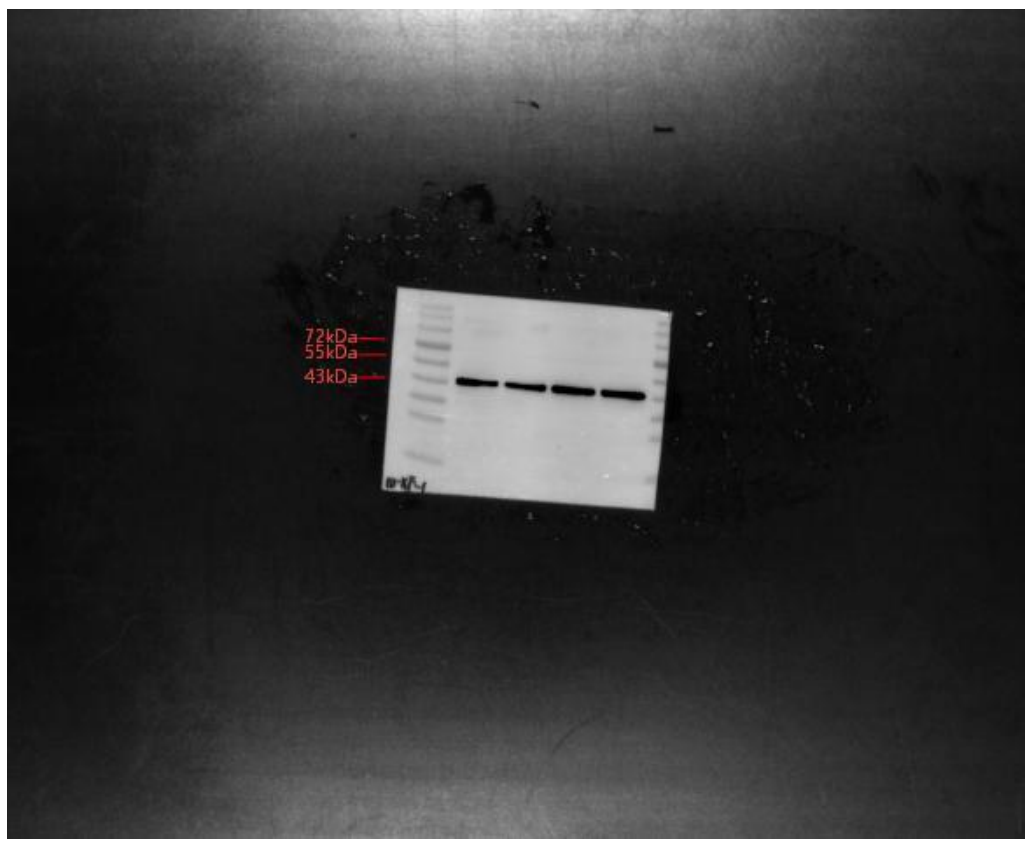

ENO1 ACTIN

ShFBXW7-2

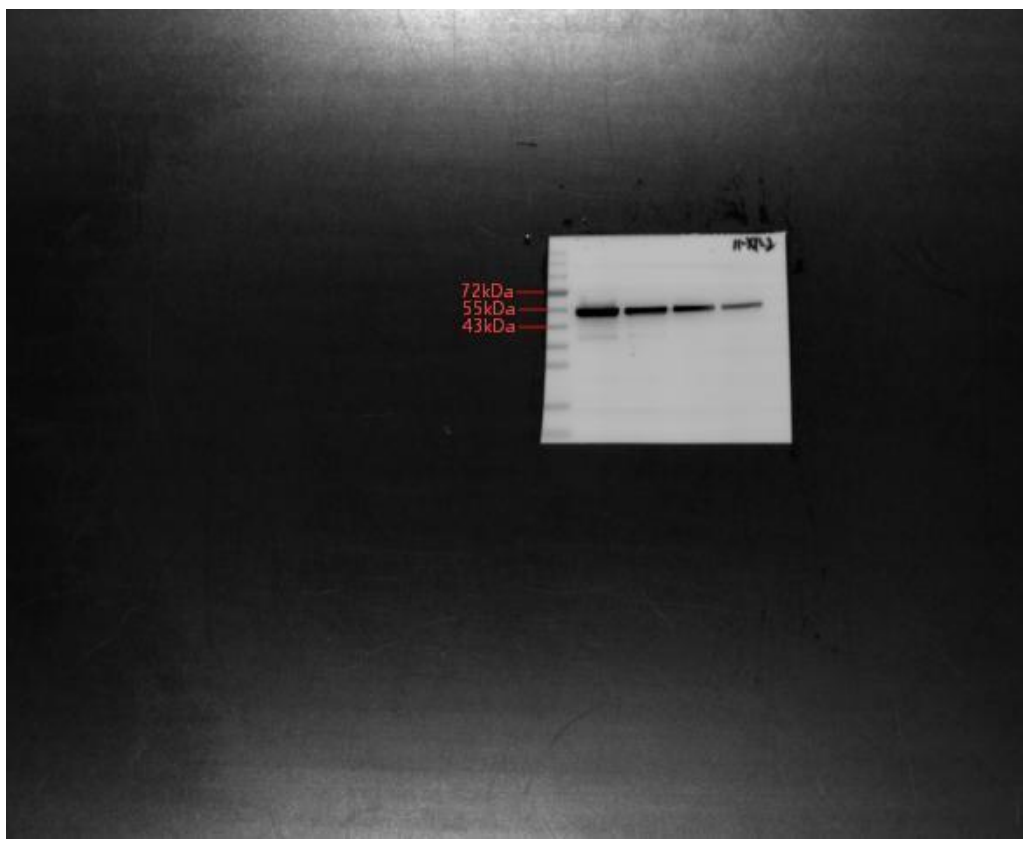

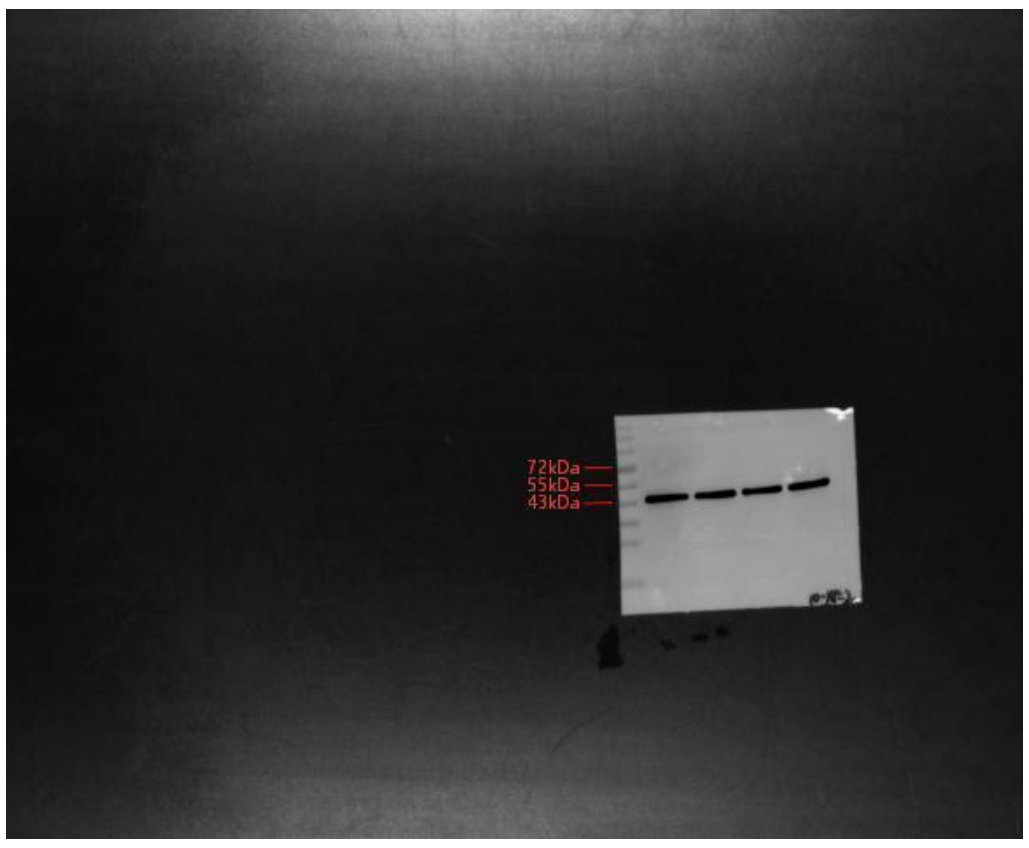

ENO1 ACTIN

FIG.4D

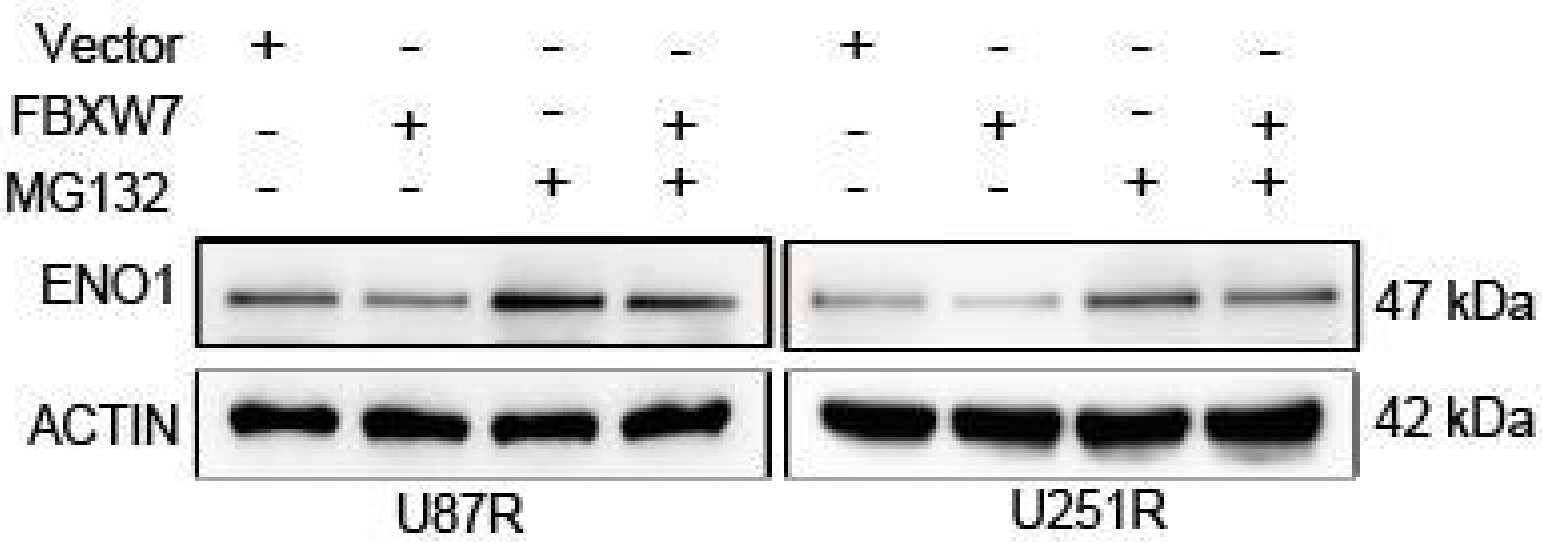

U87R

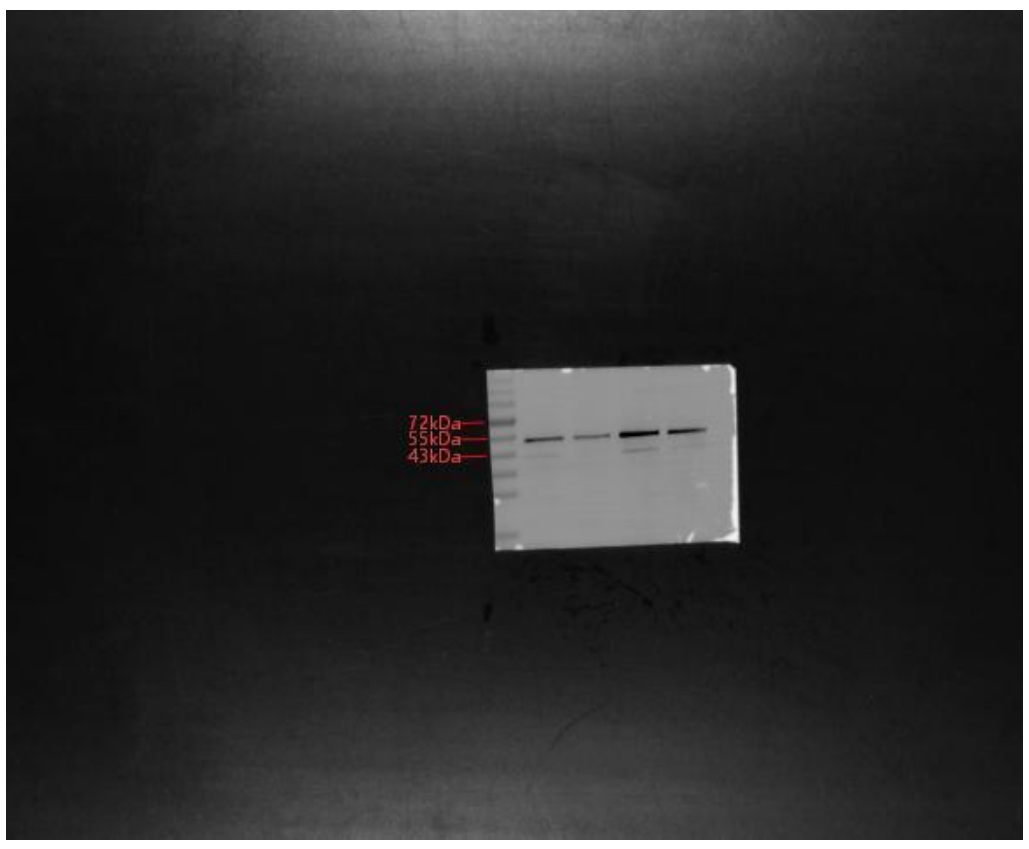

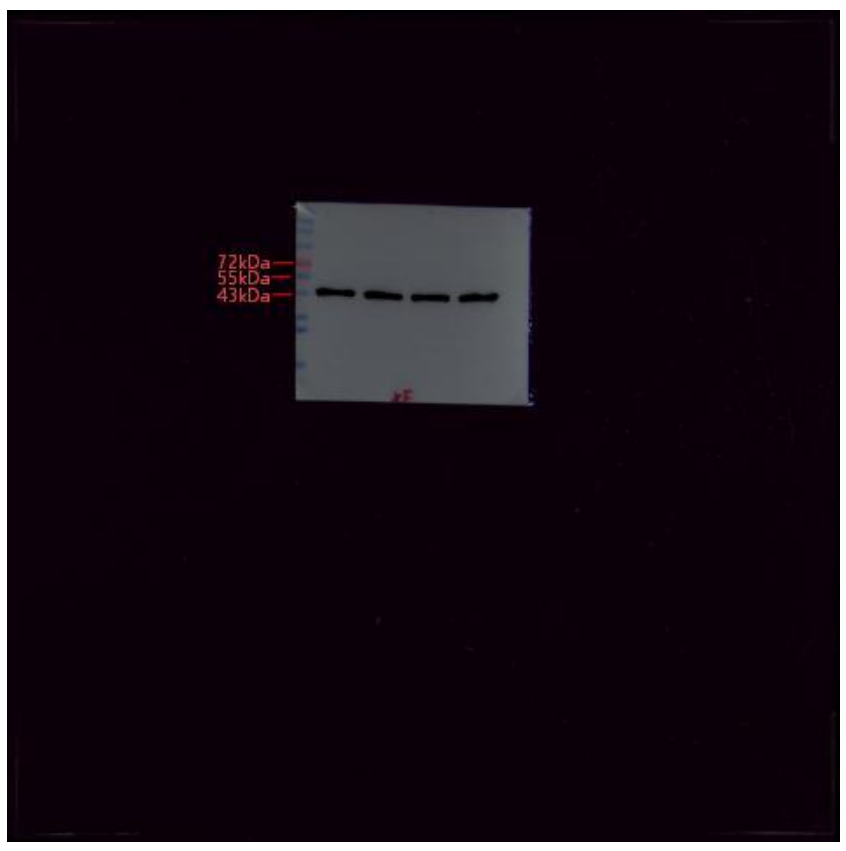

ENO1 ACTIN

U251R

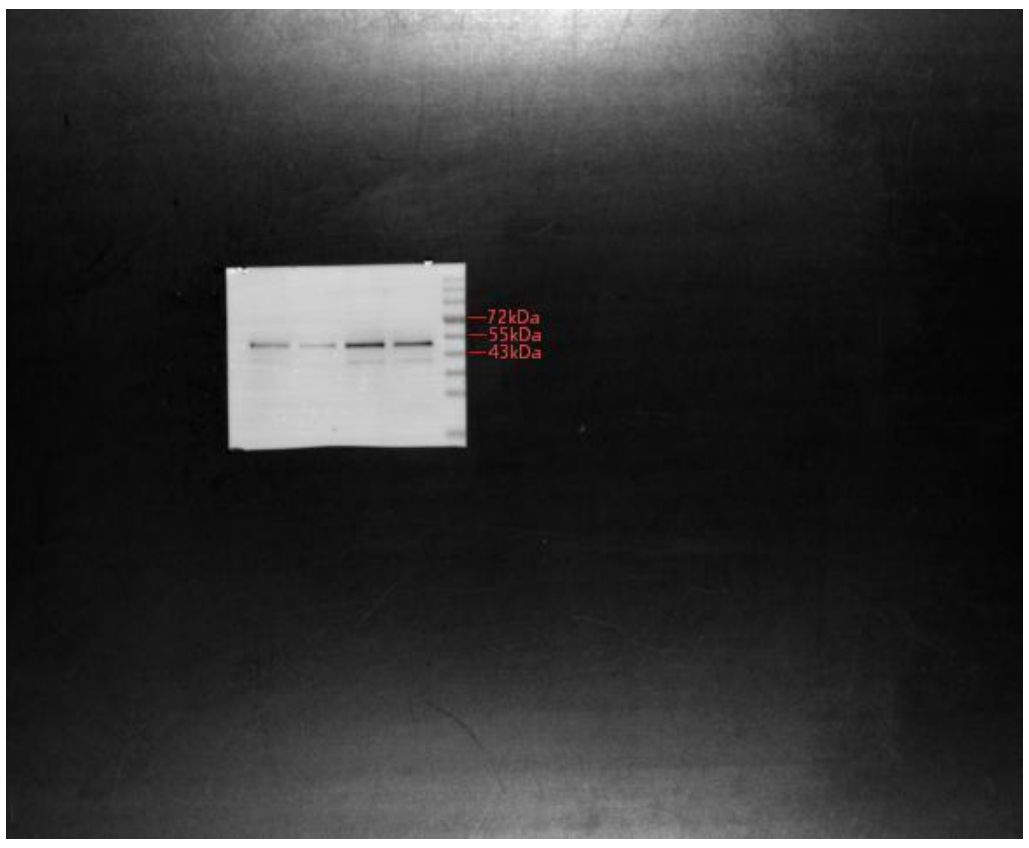

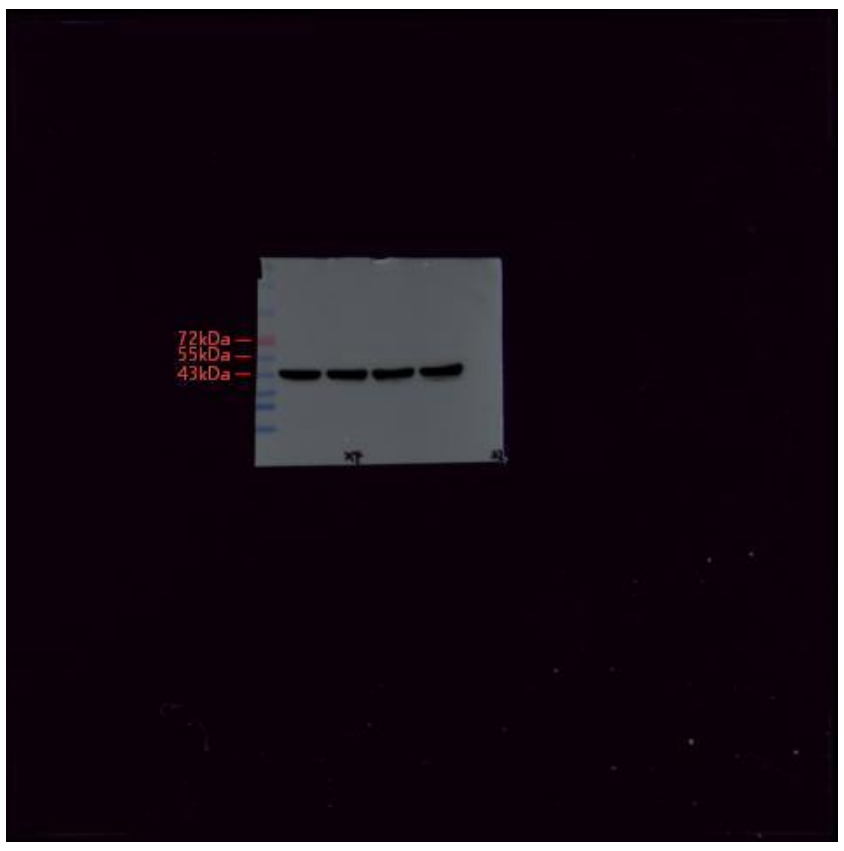

ENO1

ACTIN

FIG.4E

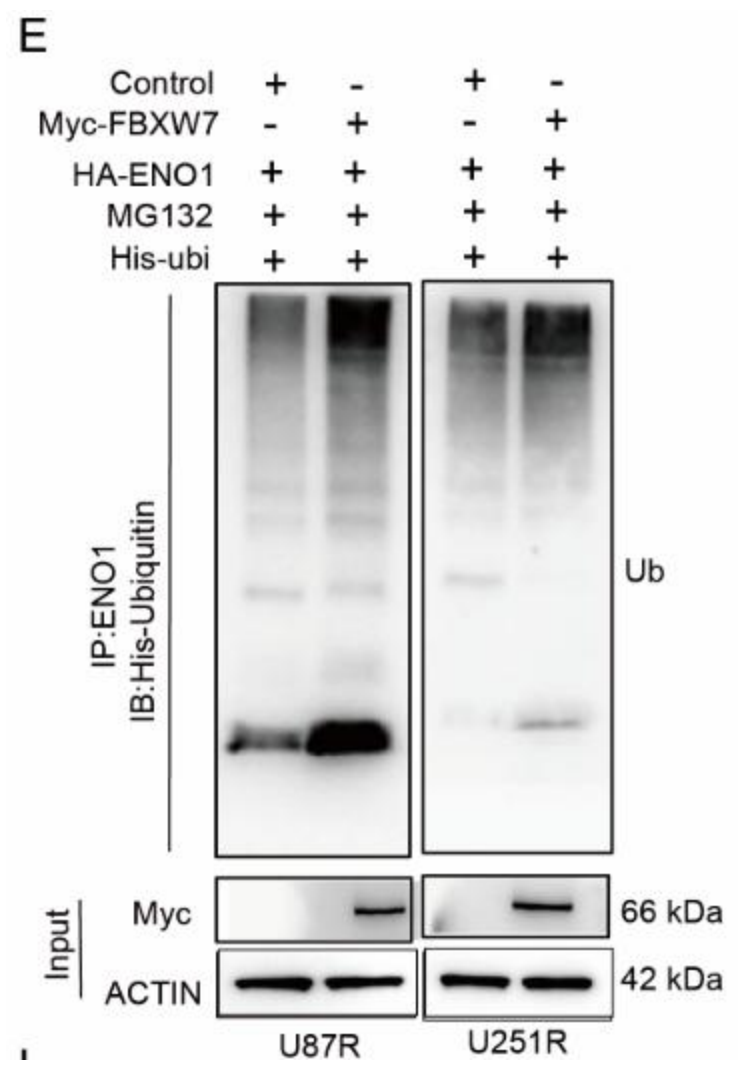

U87R

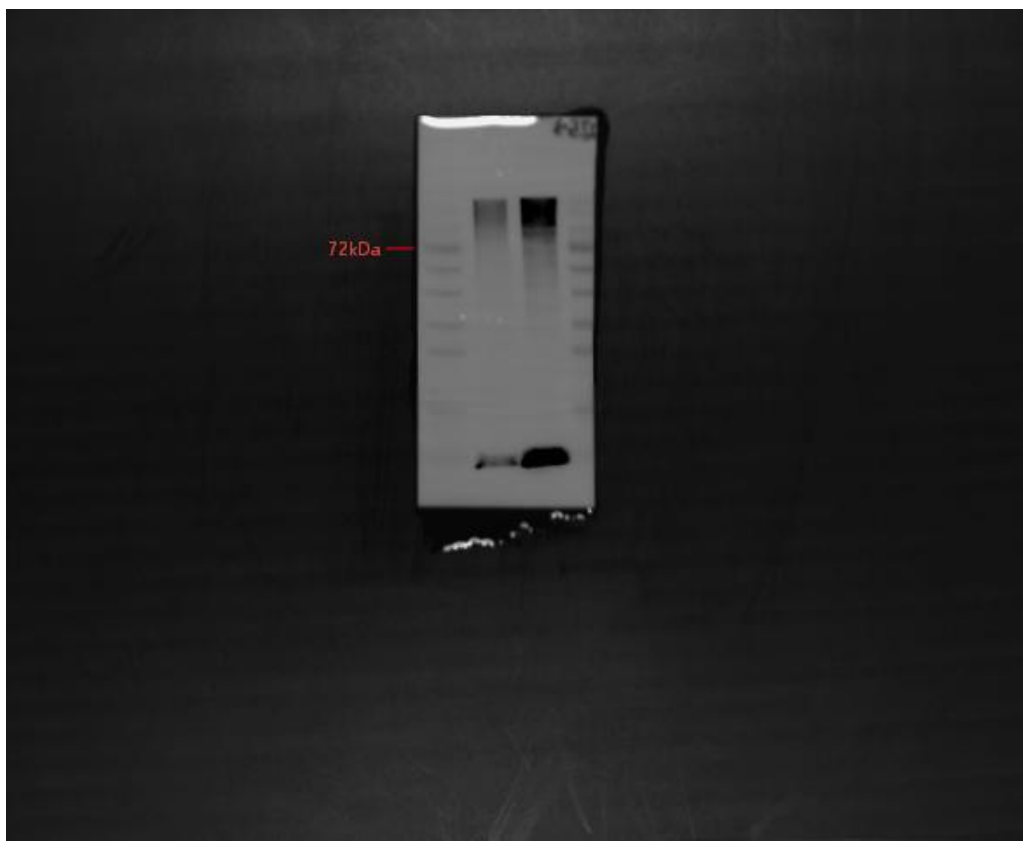

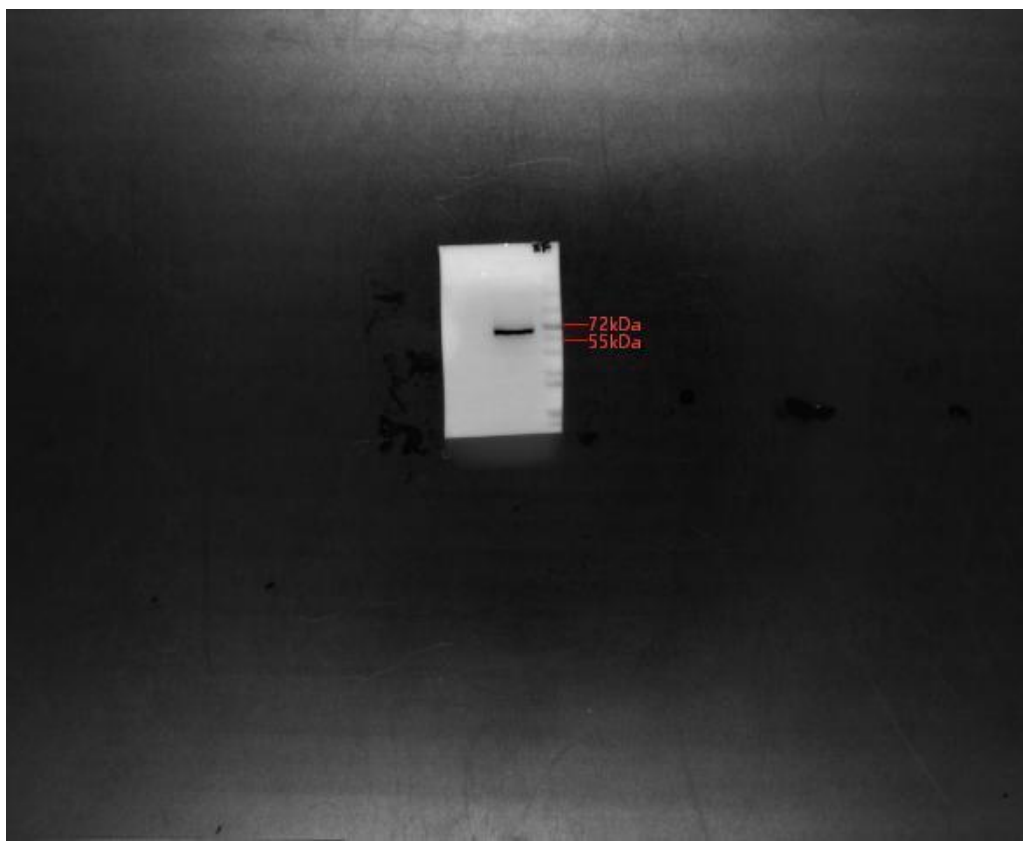

Ub

Myc

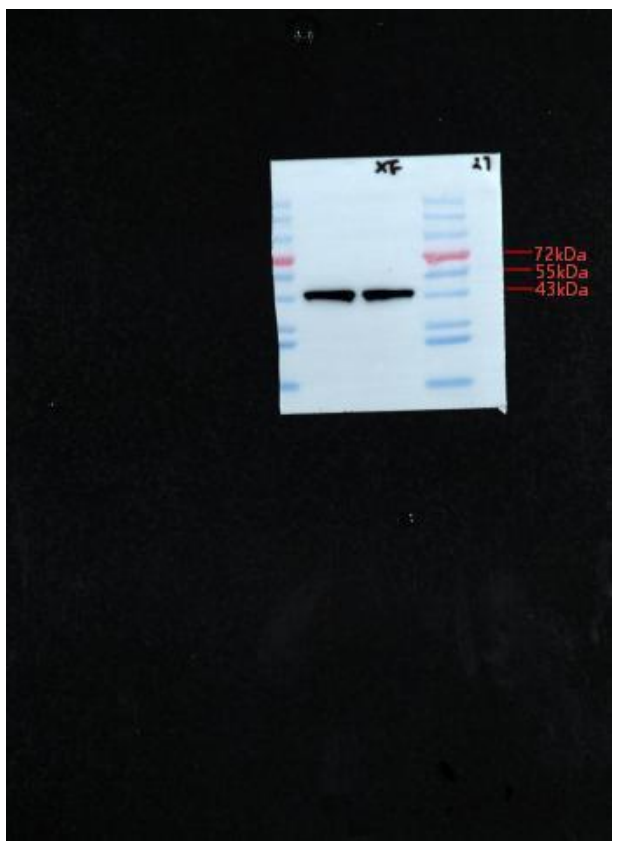

ACTIN

U251R

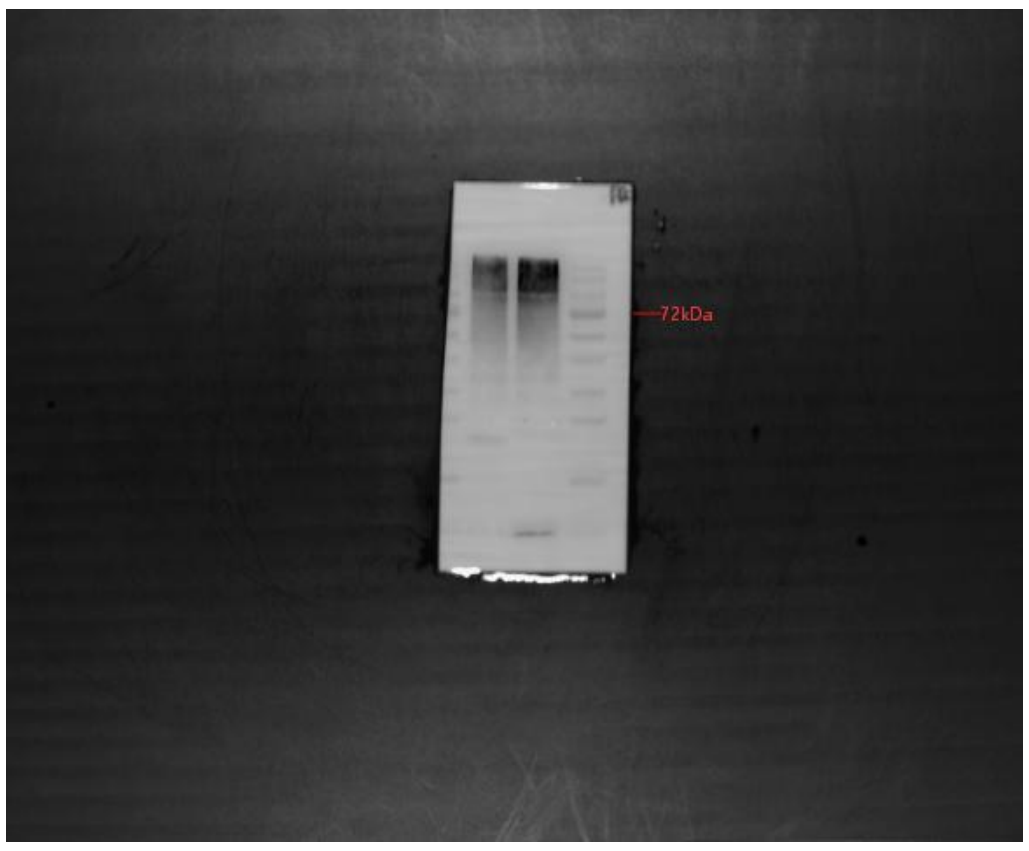

Ub

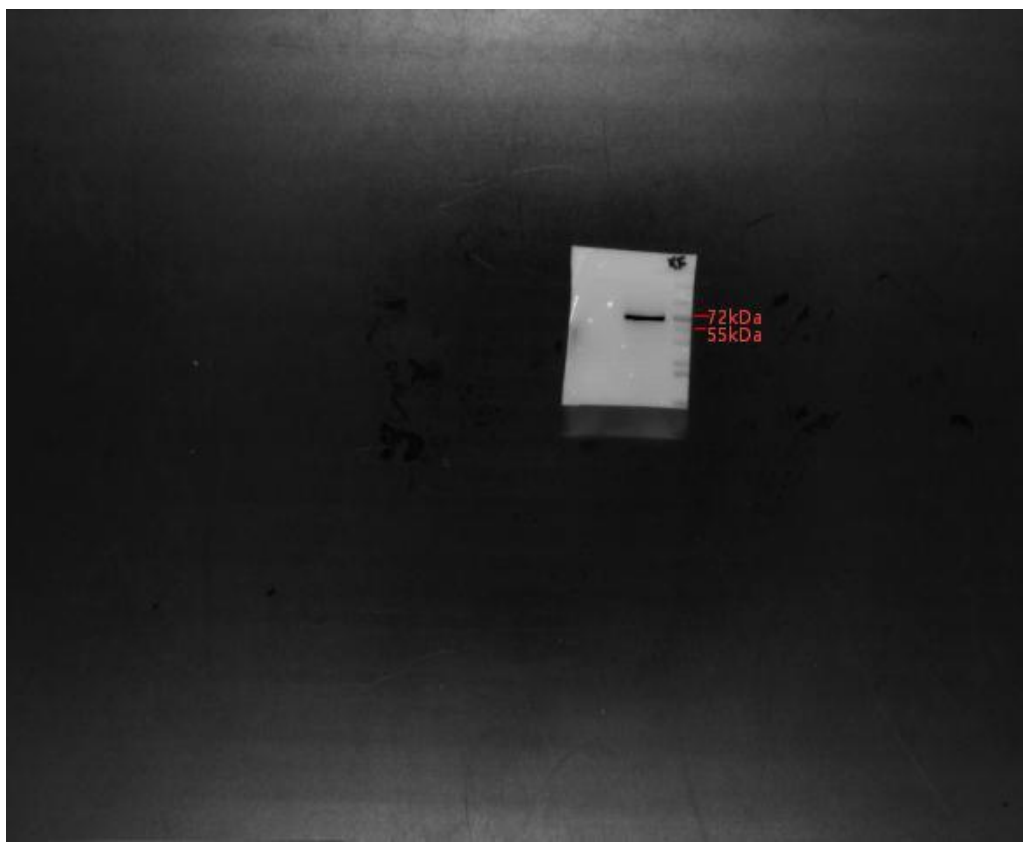

Myc

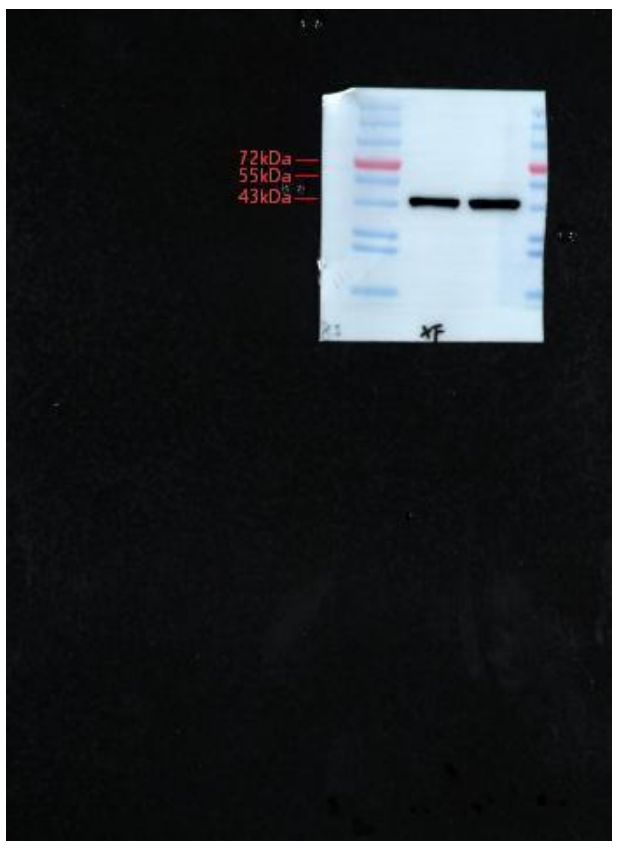

ACTIN

FIG.4F

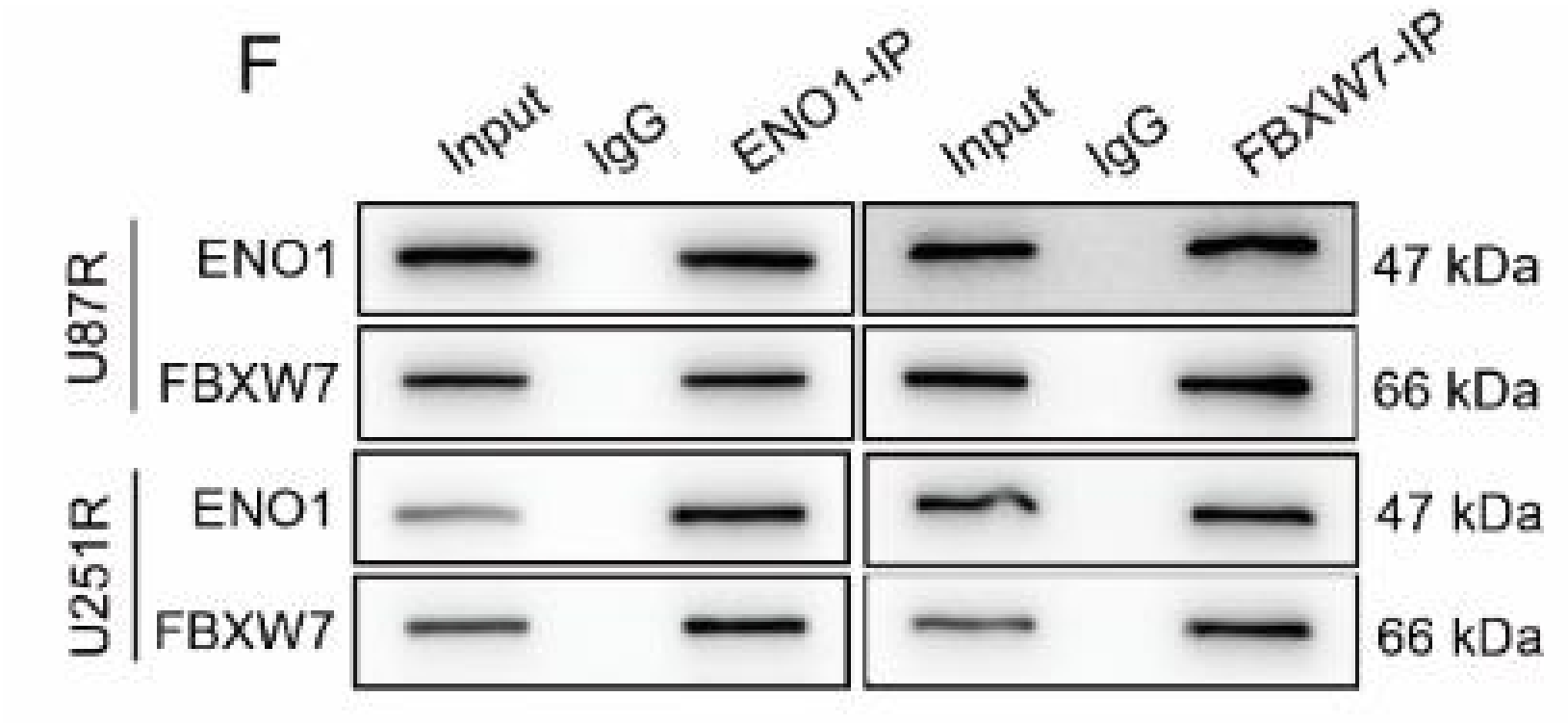

U87R

ENO1-IP

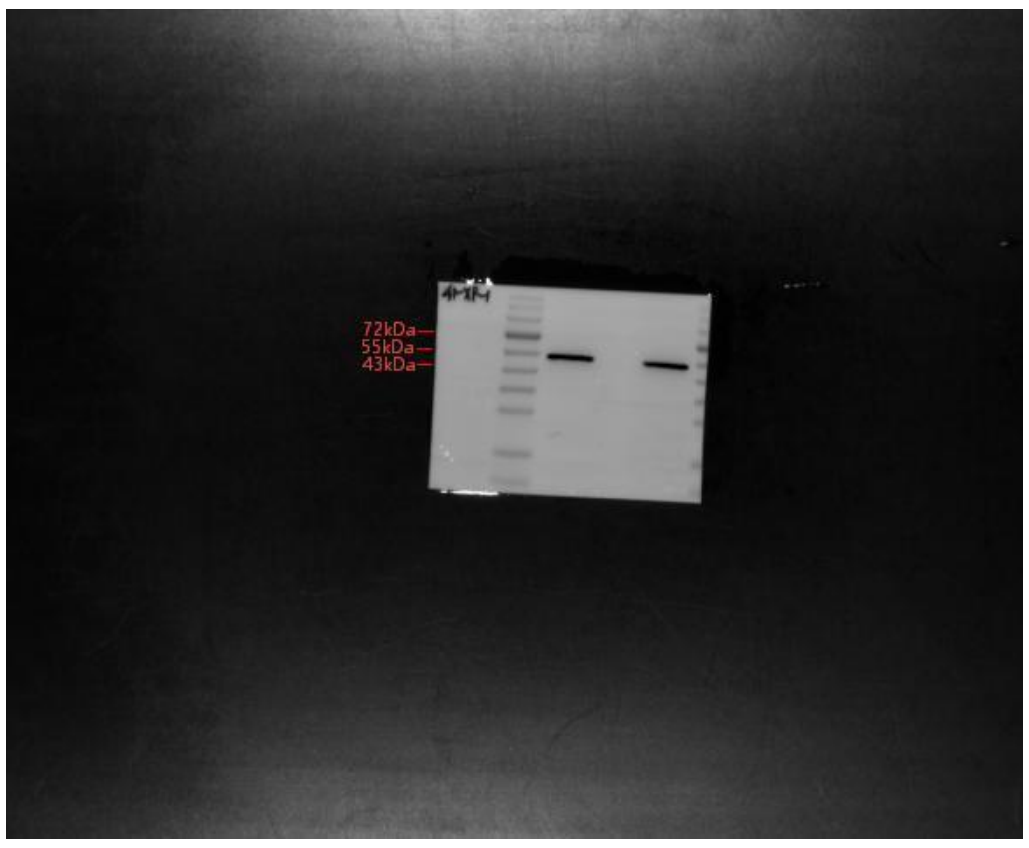

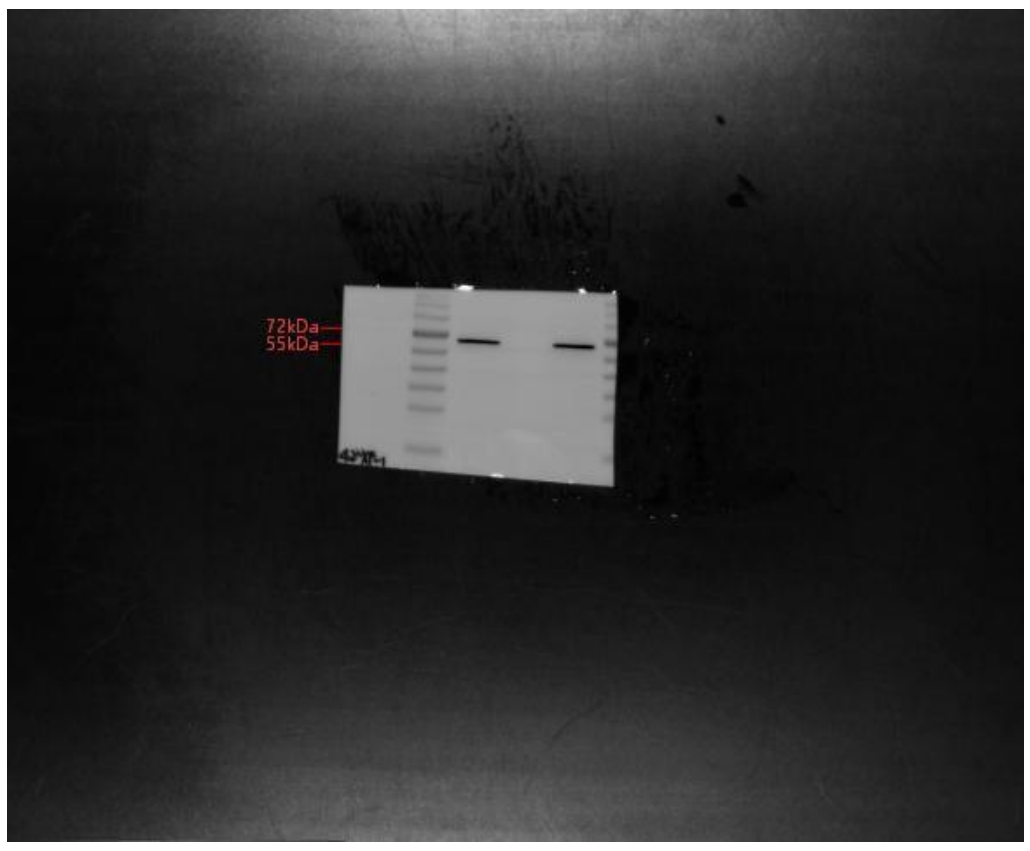

ENO1 FBXW7

FBXW7-IP

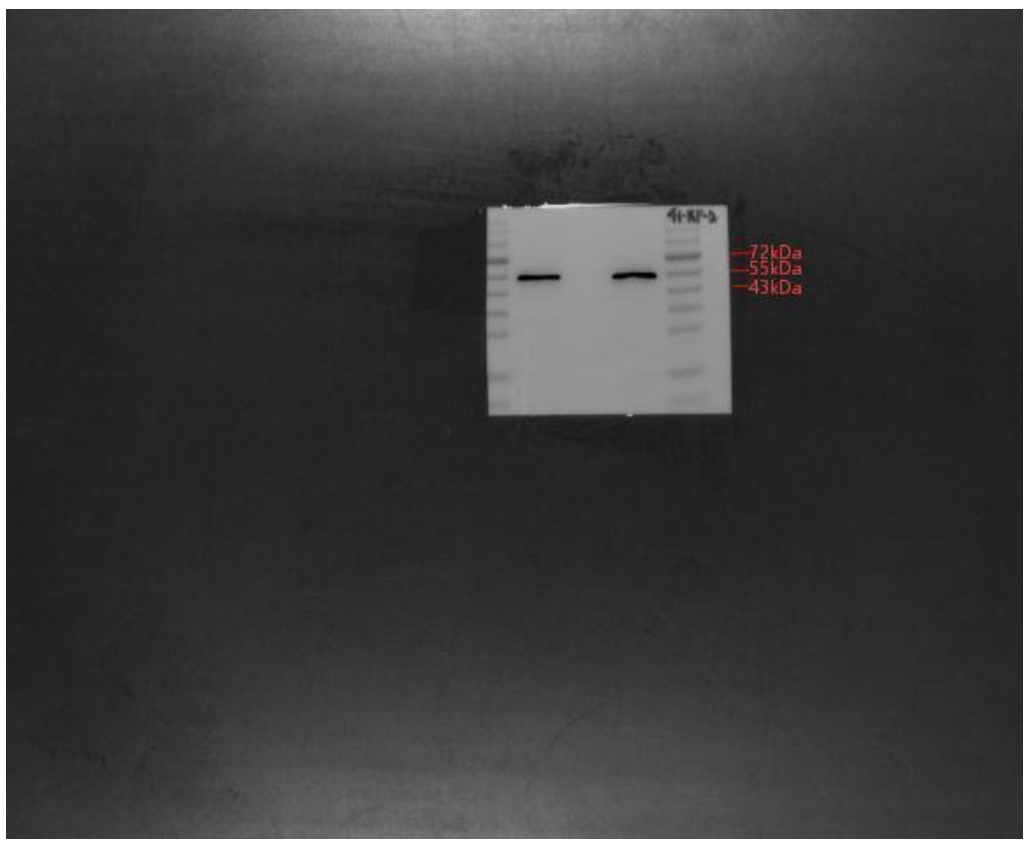

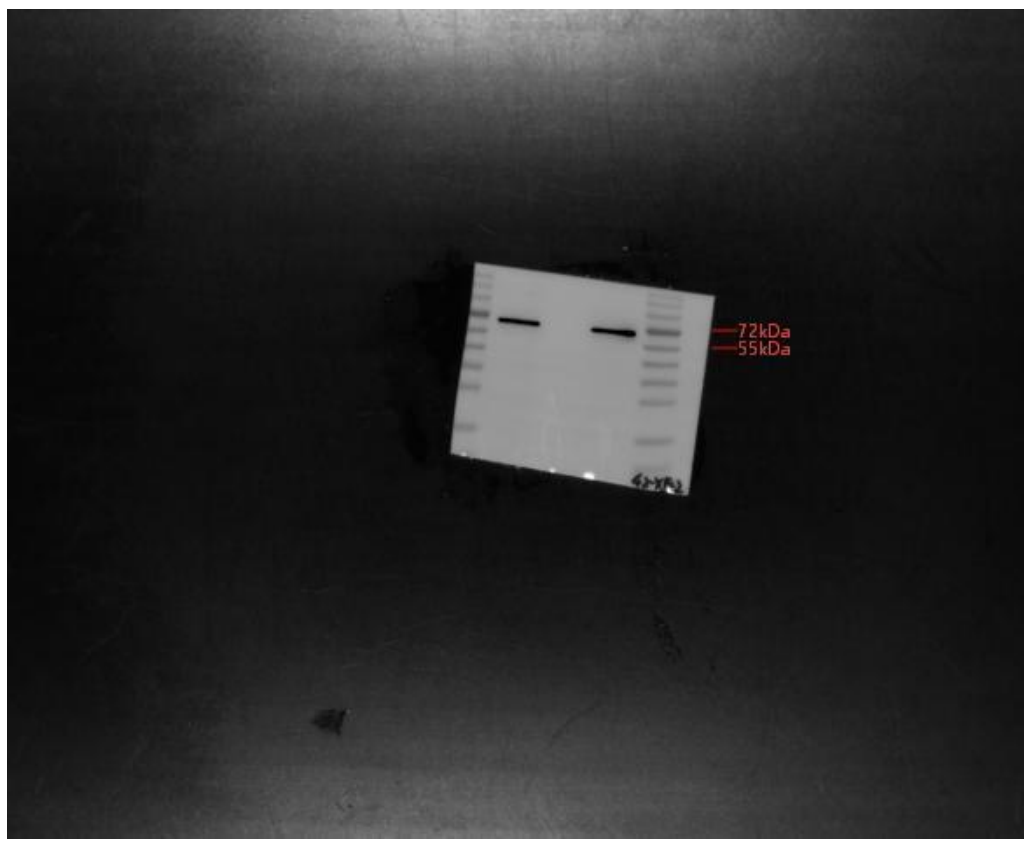

ENO1 FBXW7

U251R

ENO1-IP

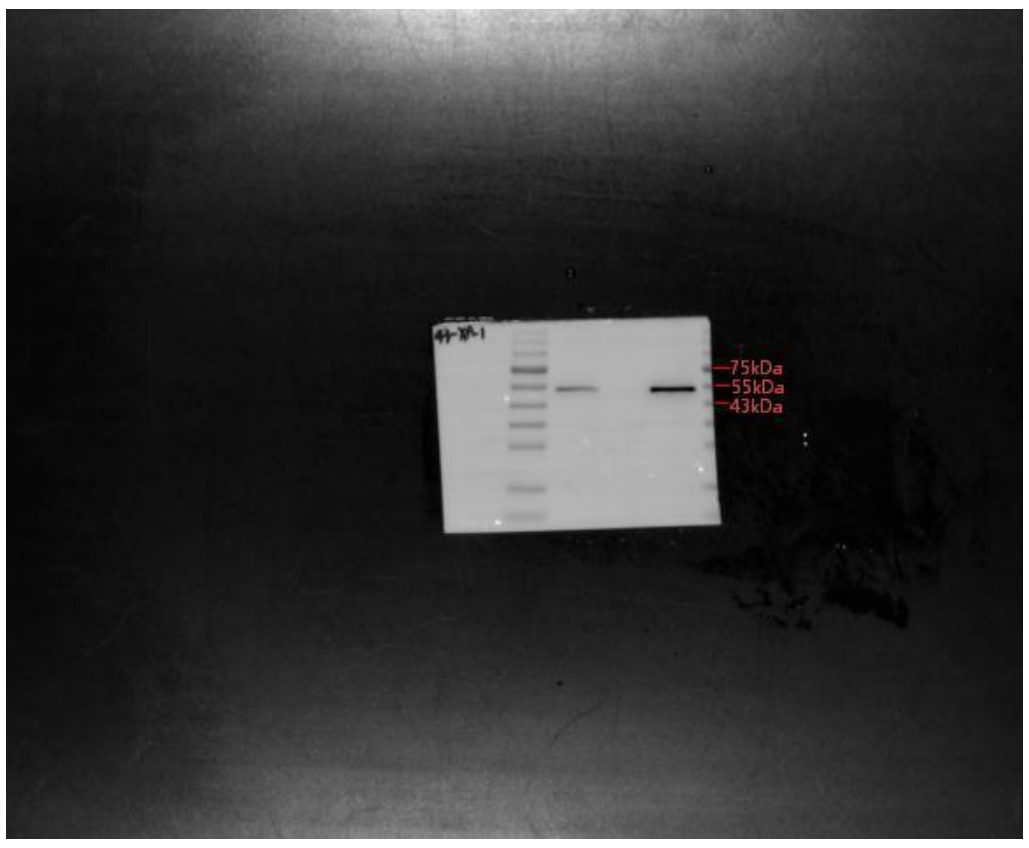

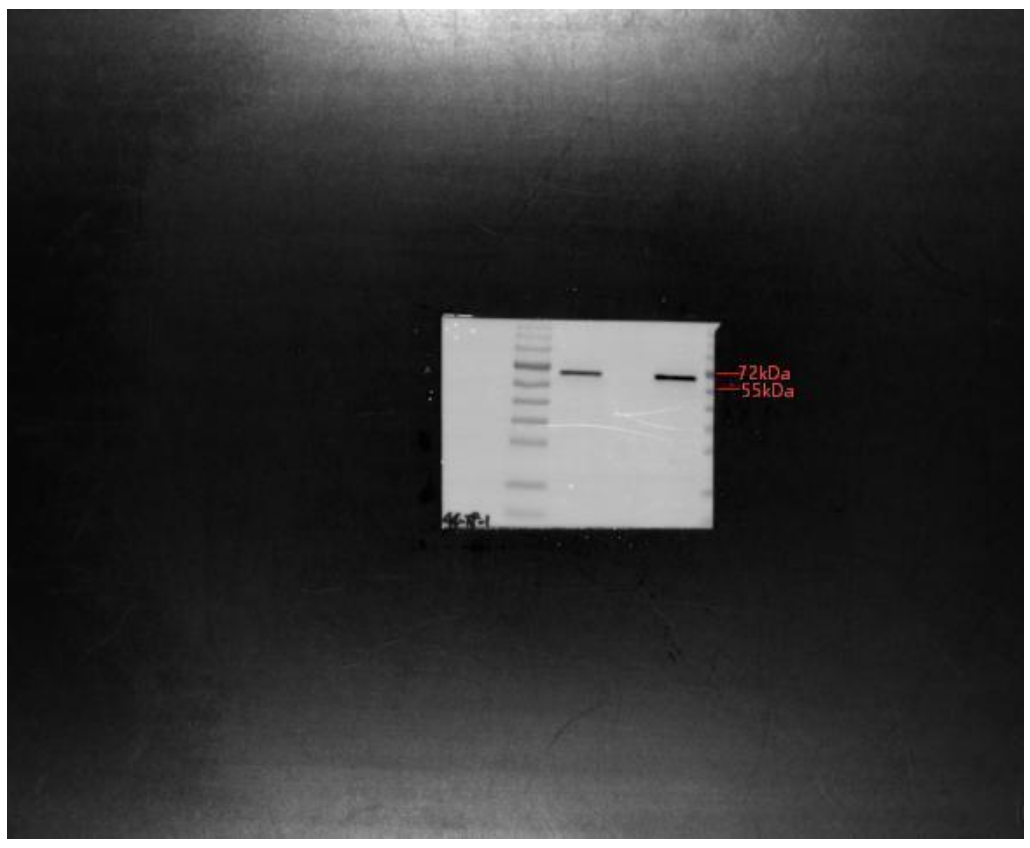

ENO1 FBXW7

FBXW7-IP

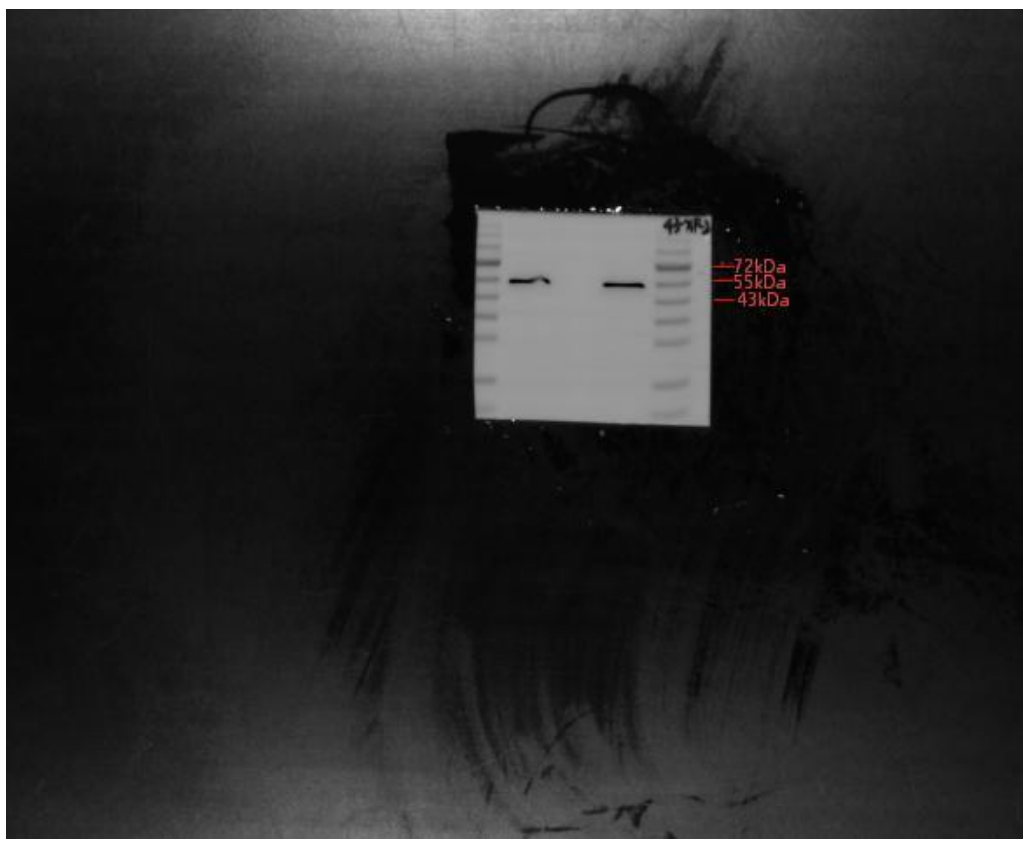

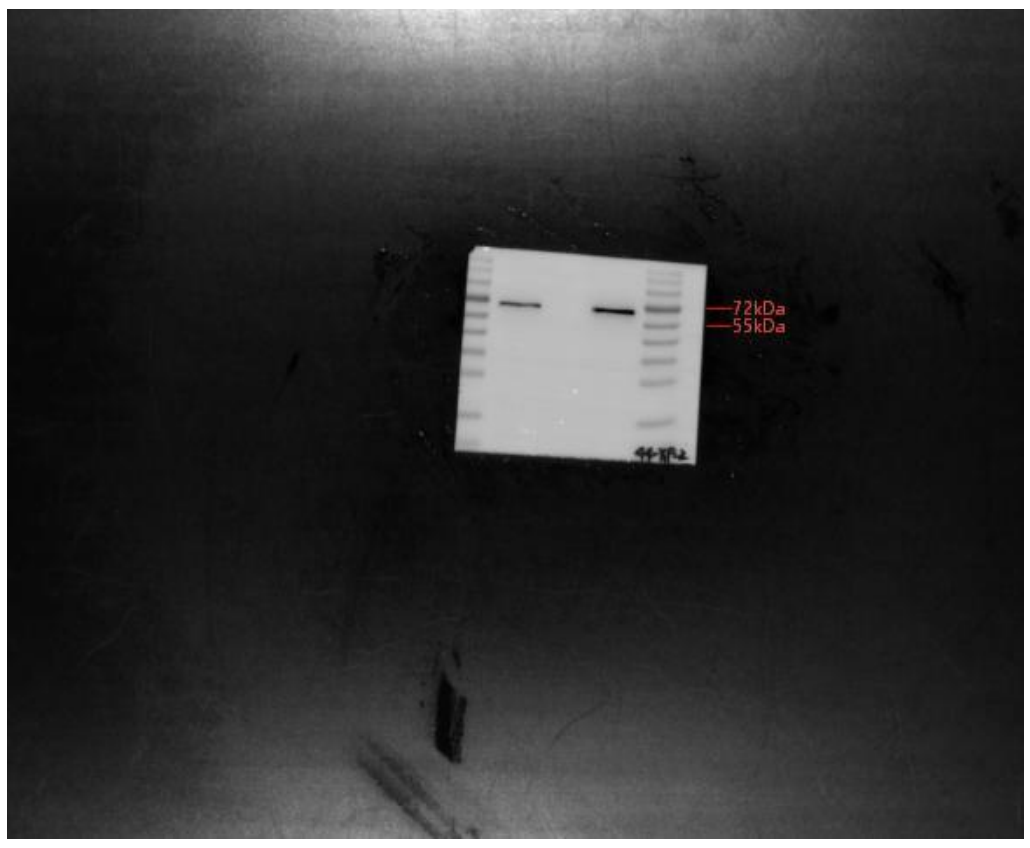

ENO1 FBXW7

FIG.4G

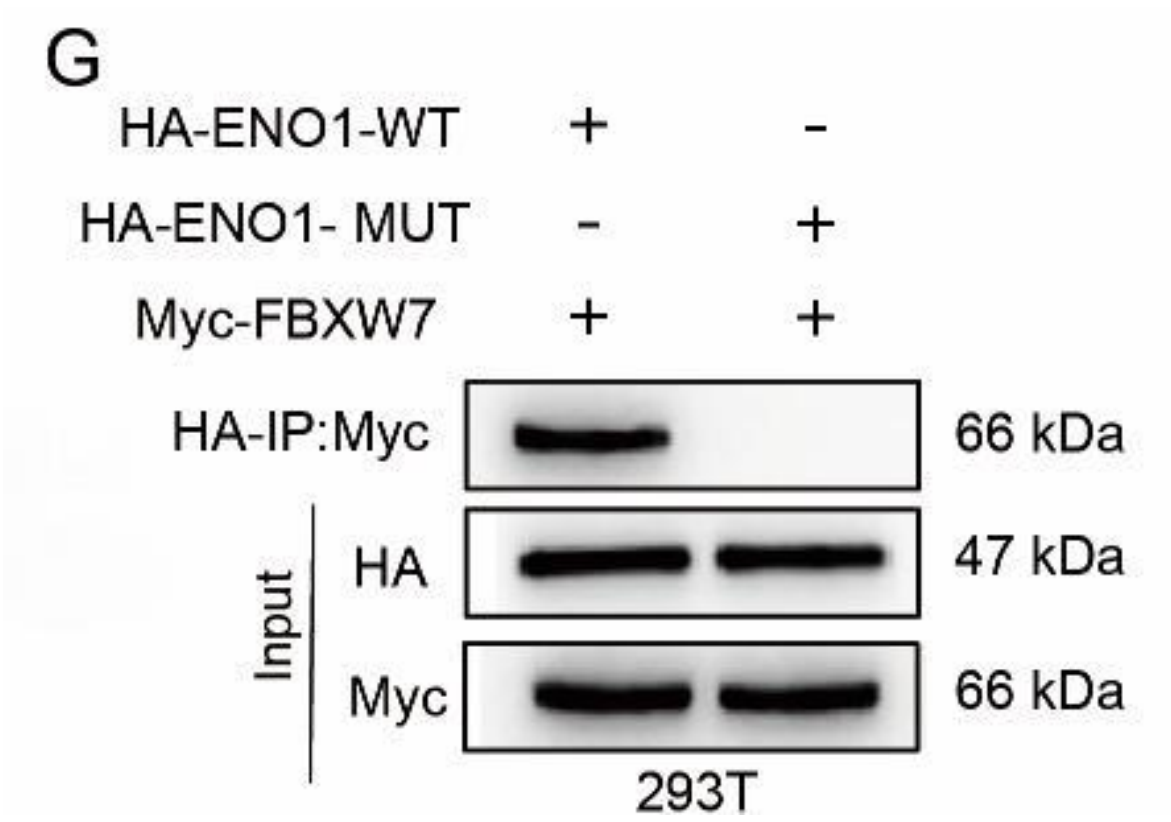

HA-IP-Myc

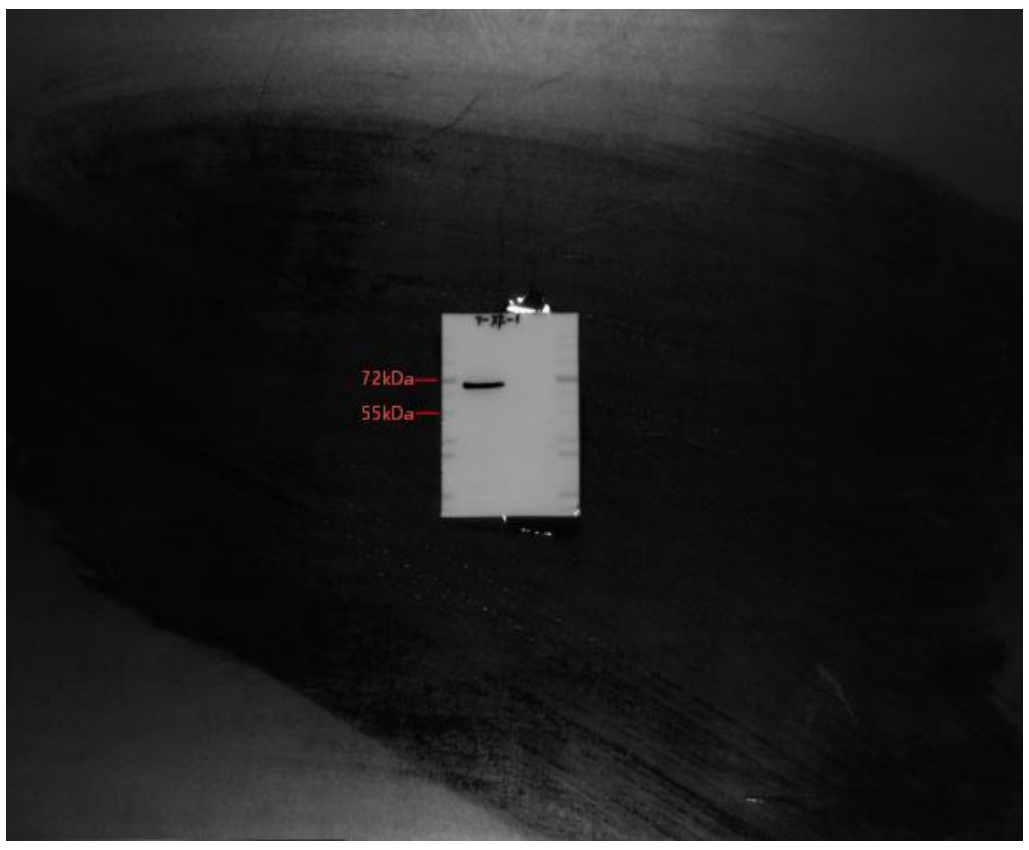

FBXW7

INPUT

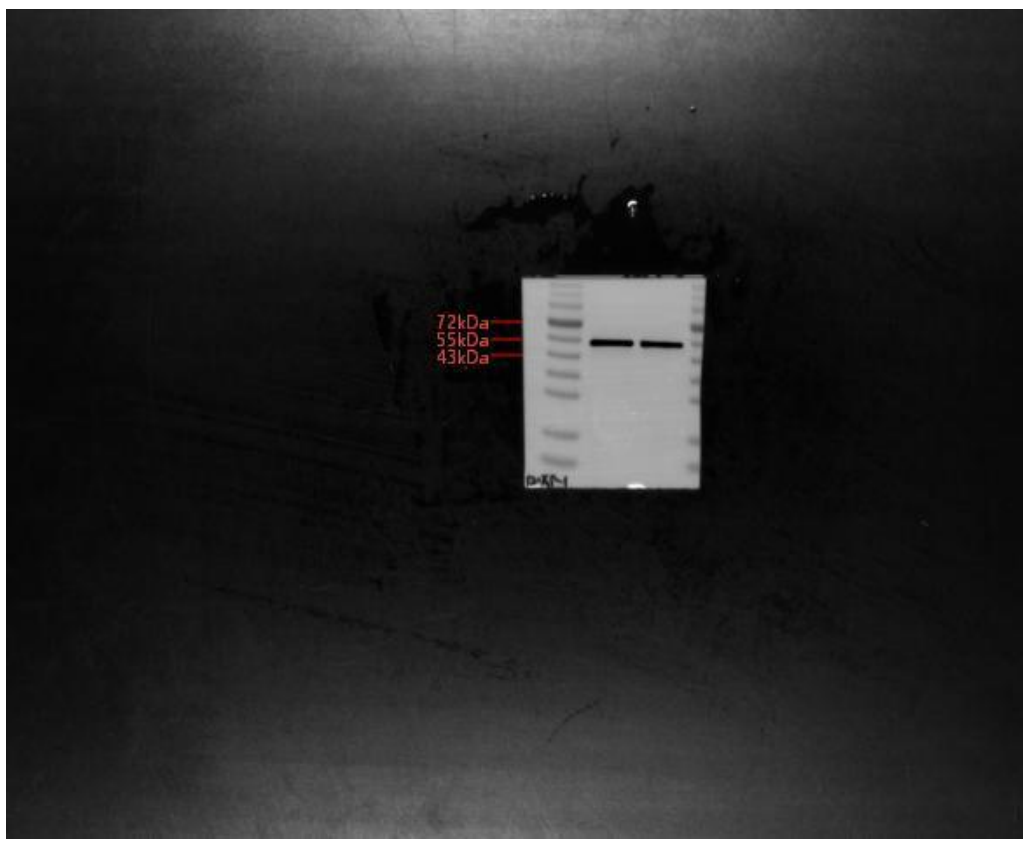

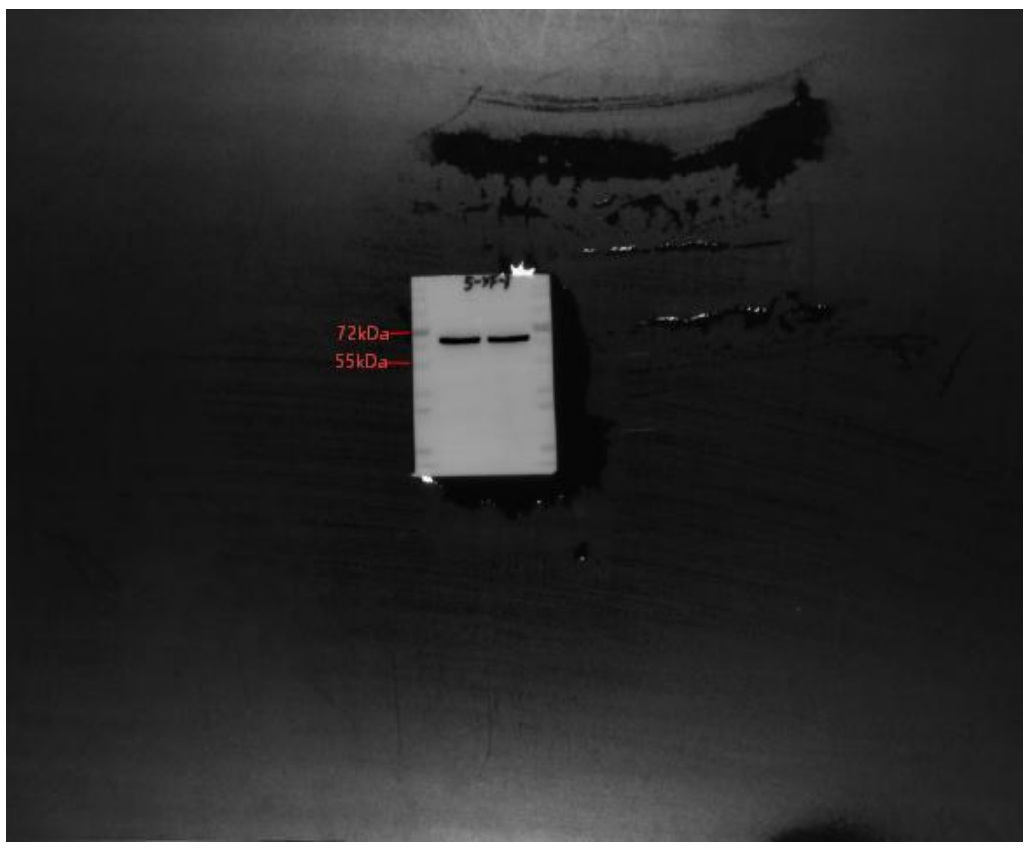

ENO1 FBXW7

FIG.4H

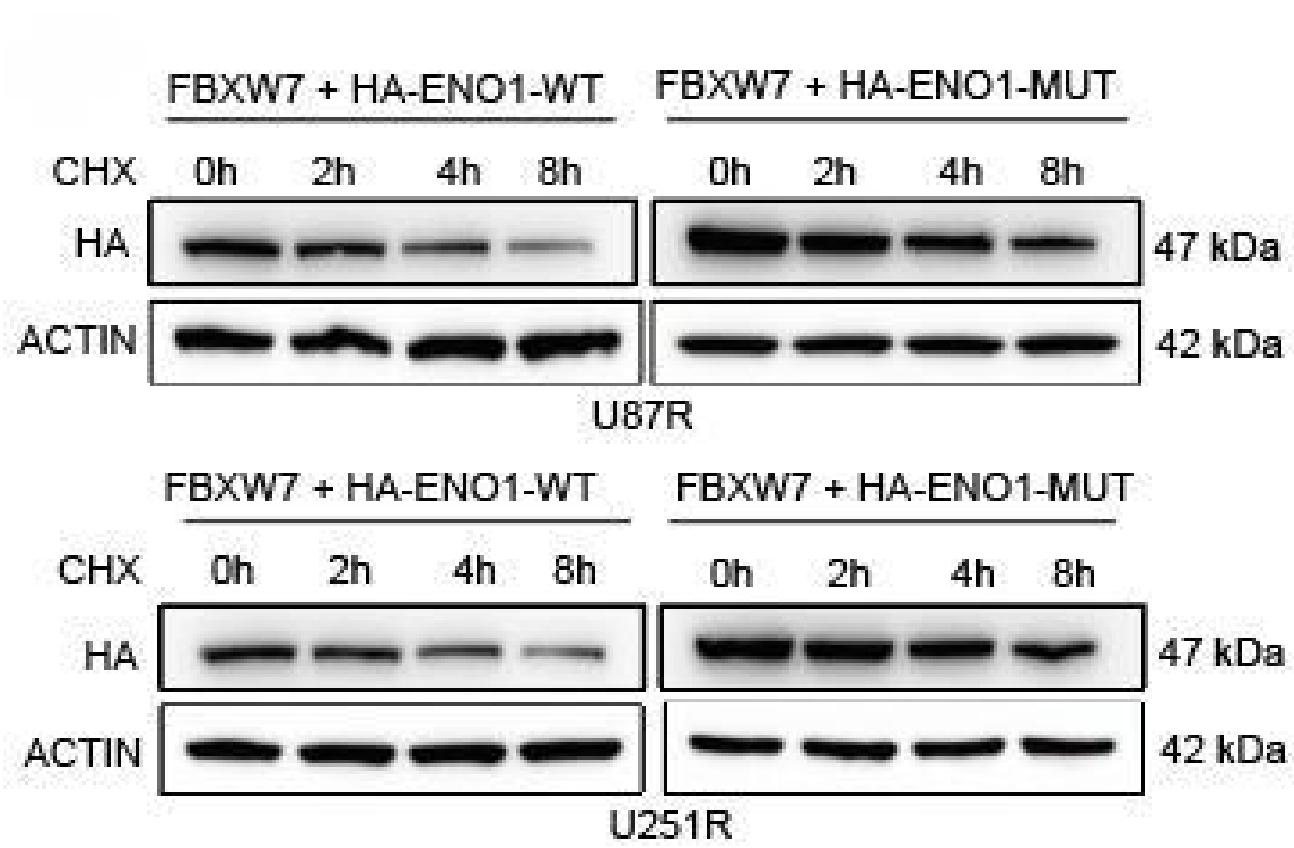

U87R

WT

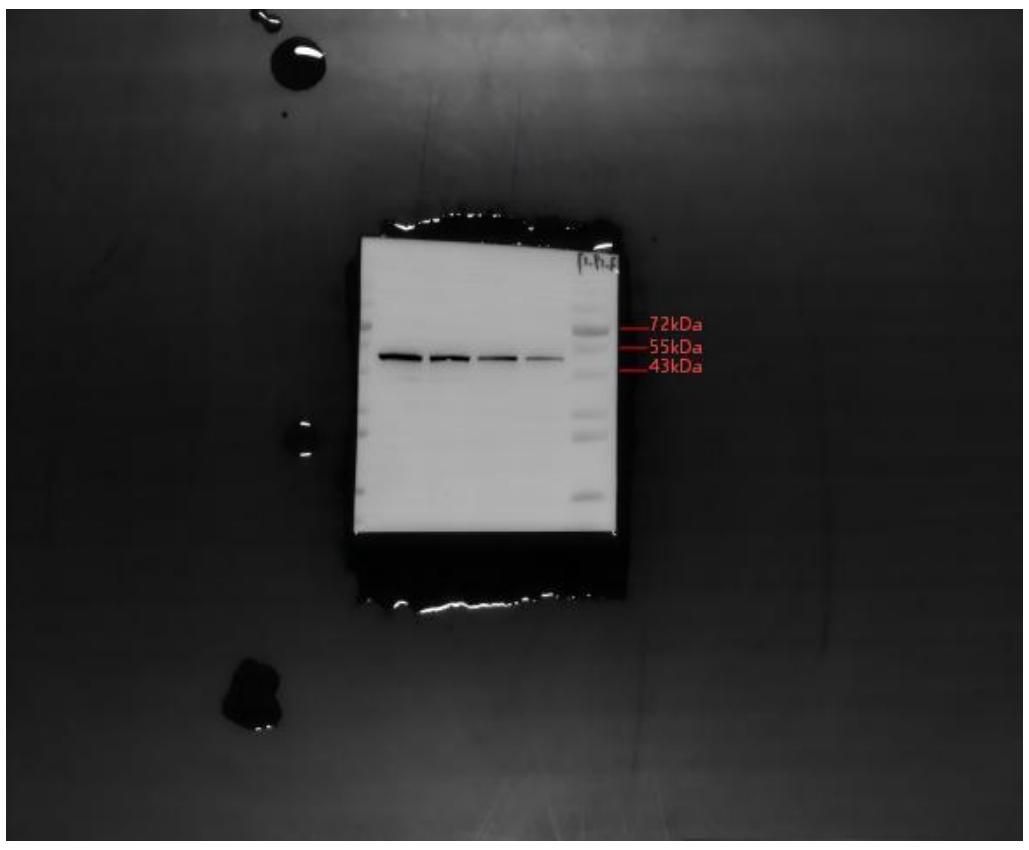

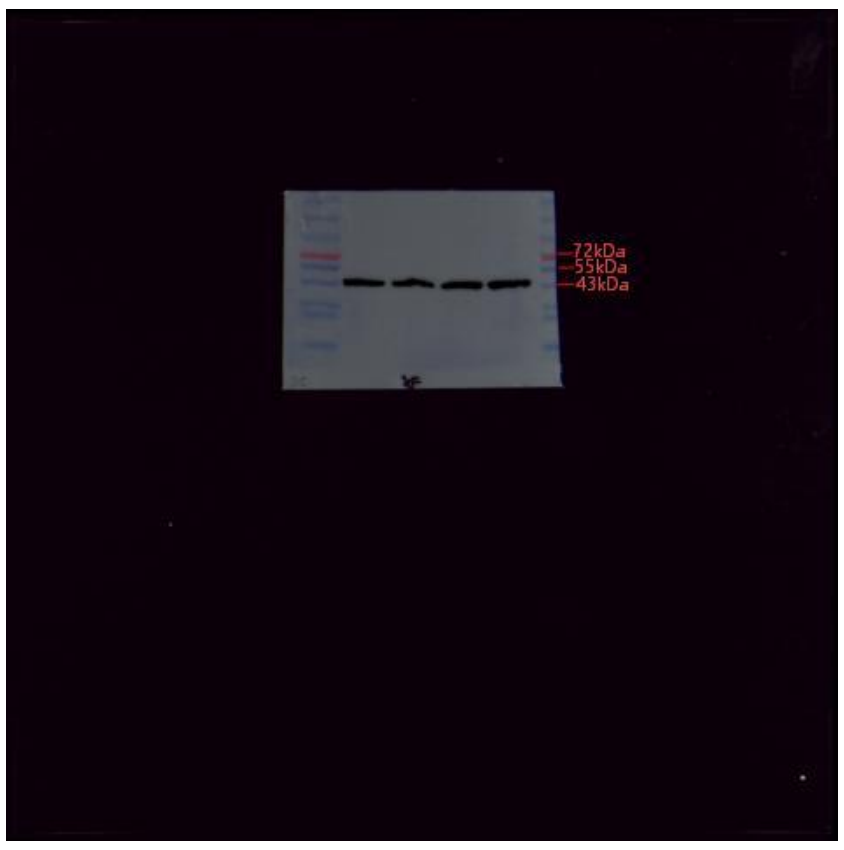

ENO1

ACTIN

MUT

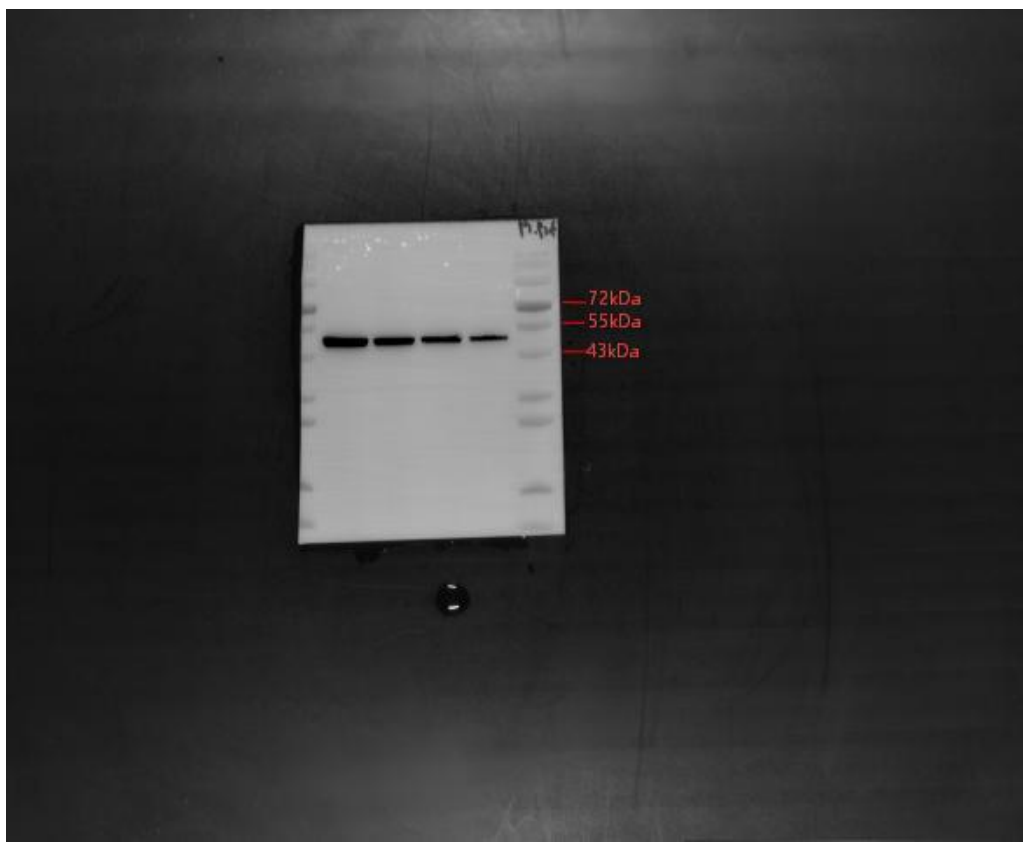

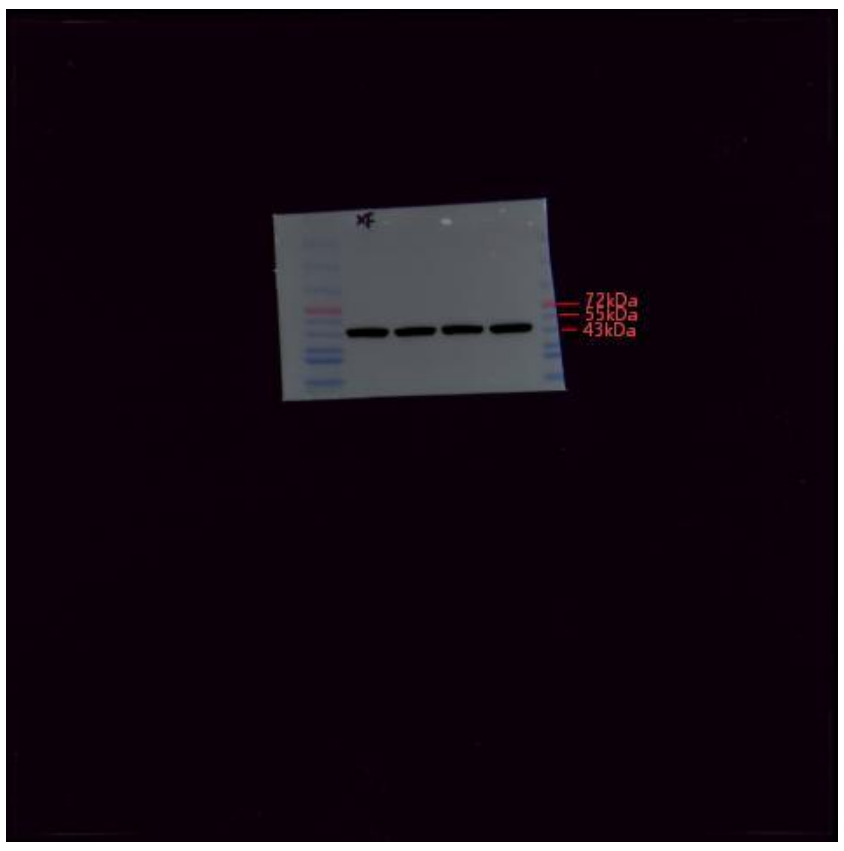

ENO1

ACTIN

U251R

WT

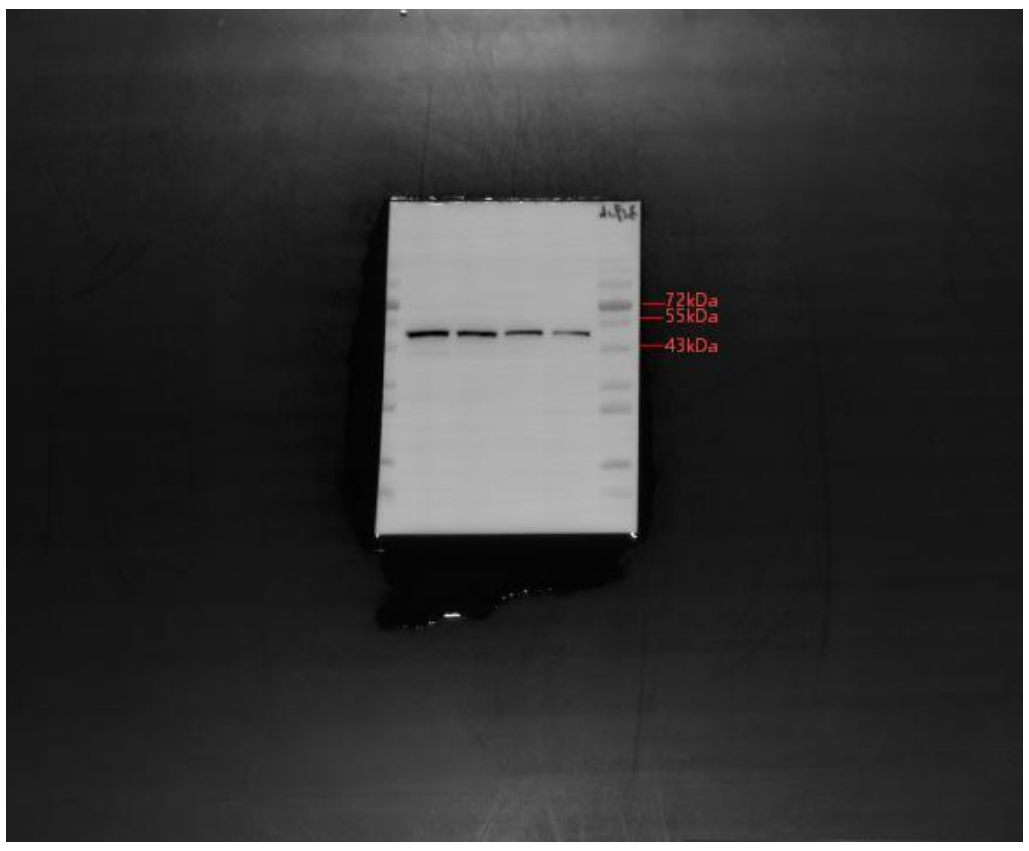

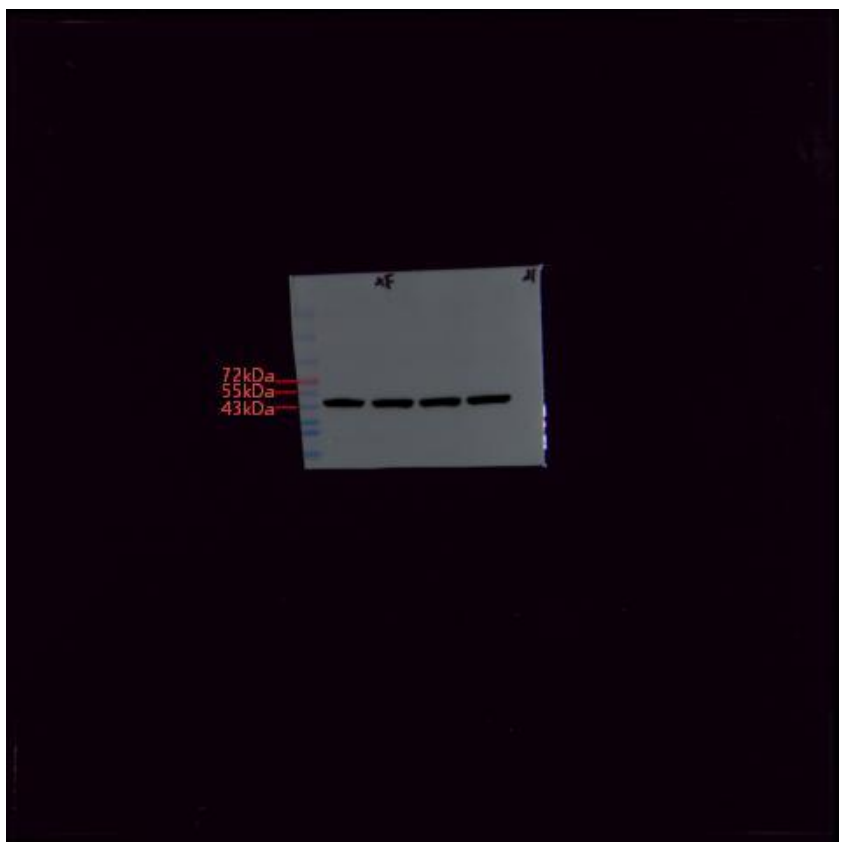

ENO1

ACTIN

MUT

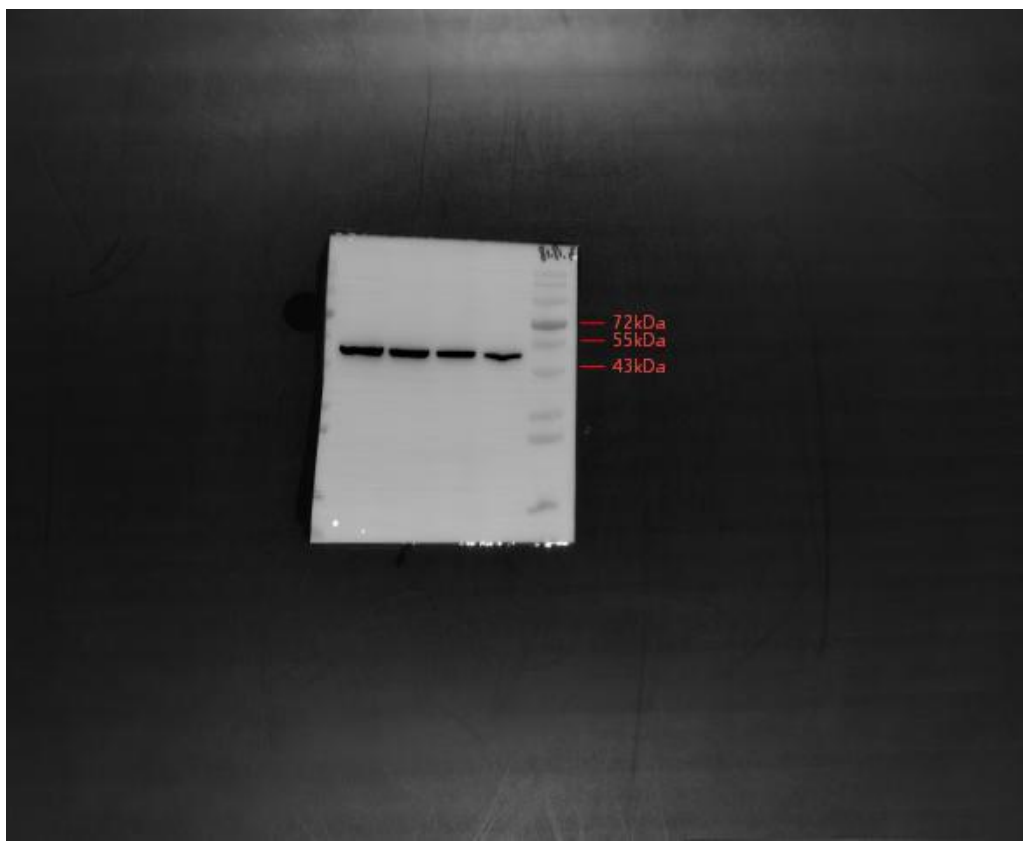

ENO1

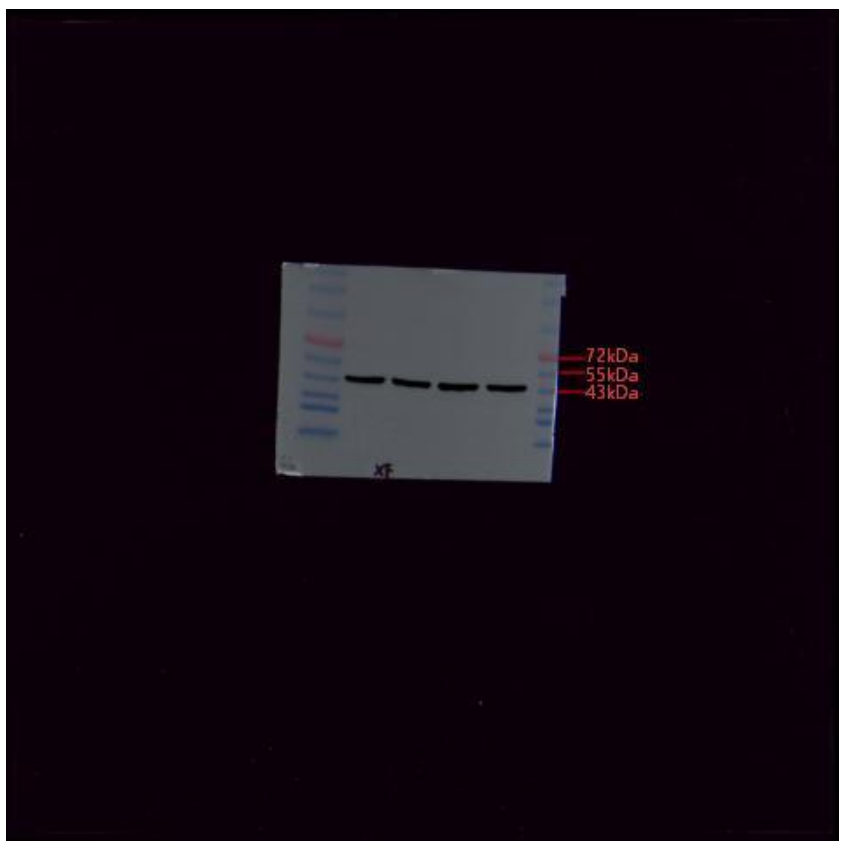

ACTIN

FIG.4I

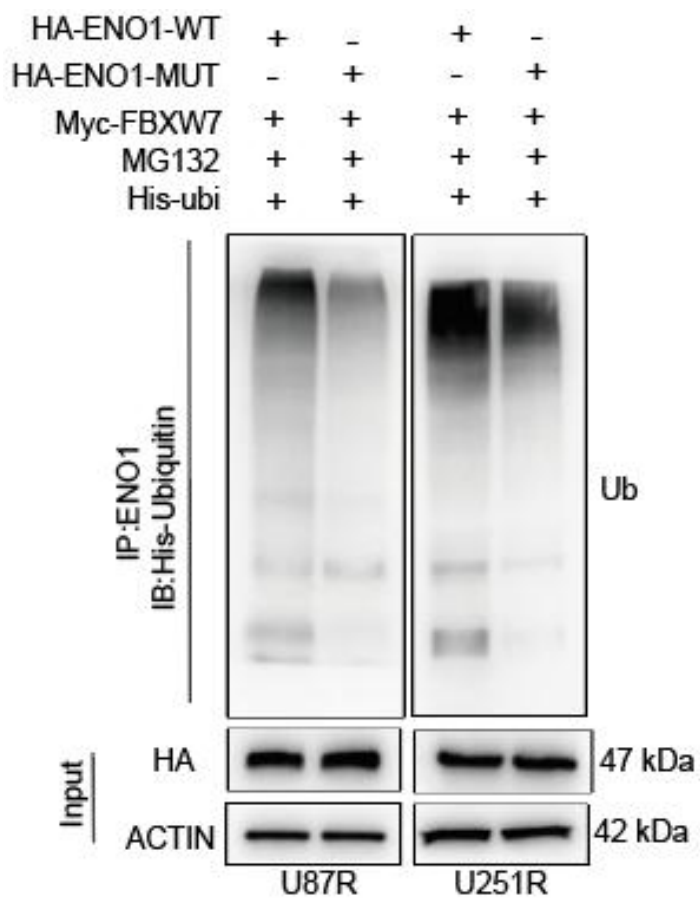

U87R

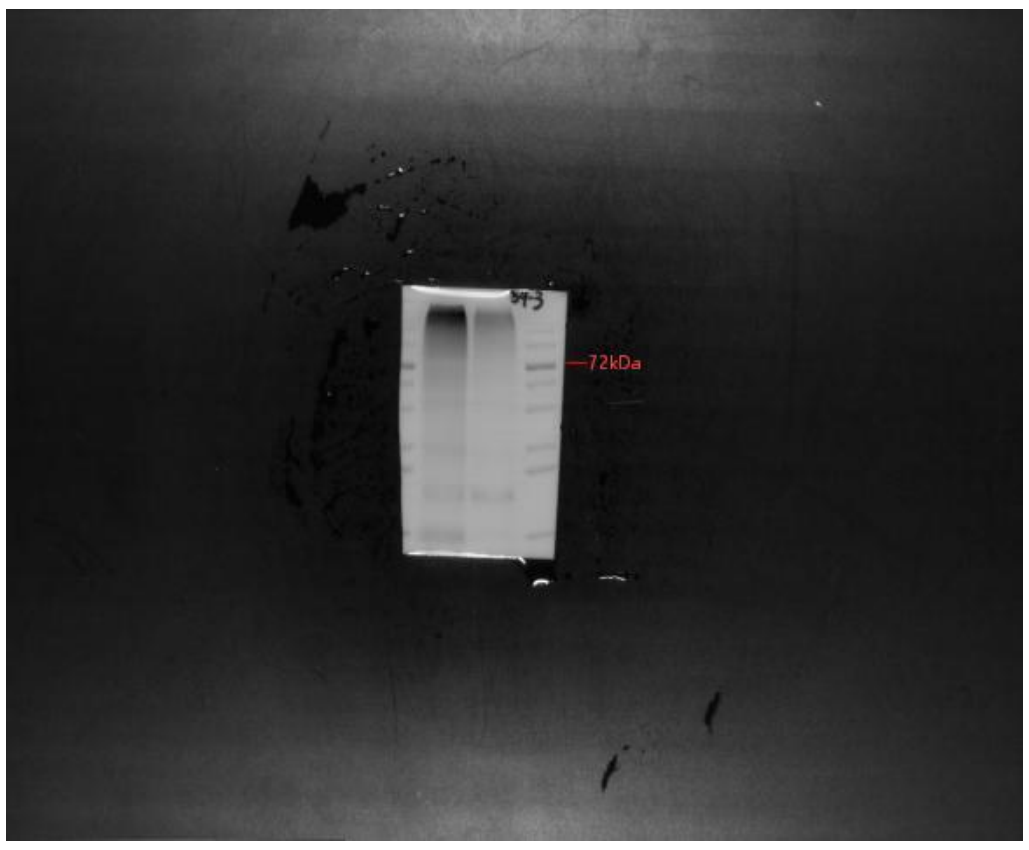

Ub

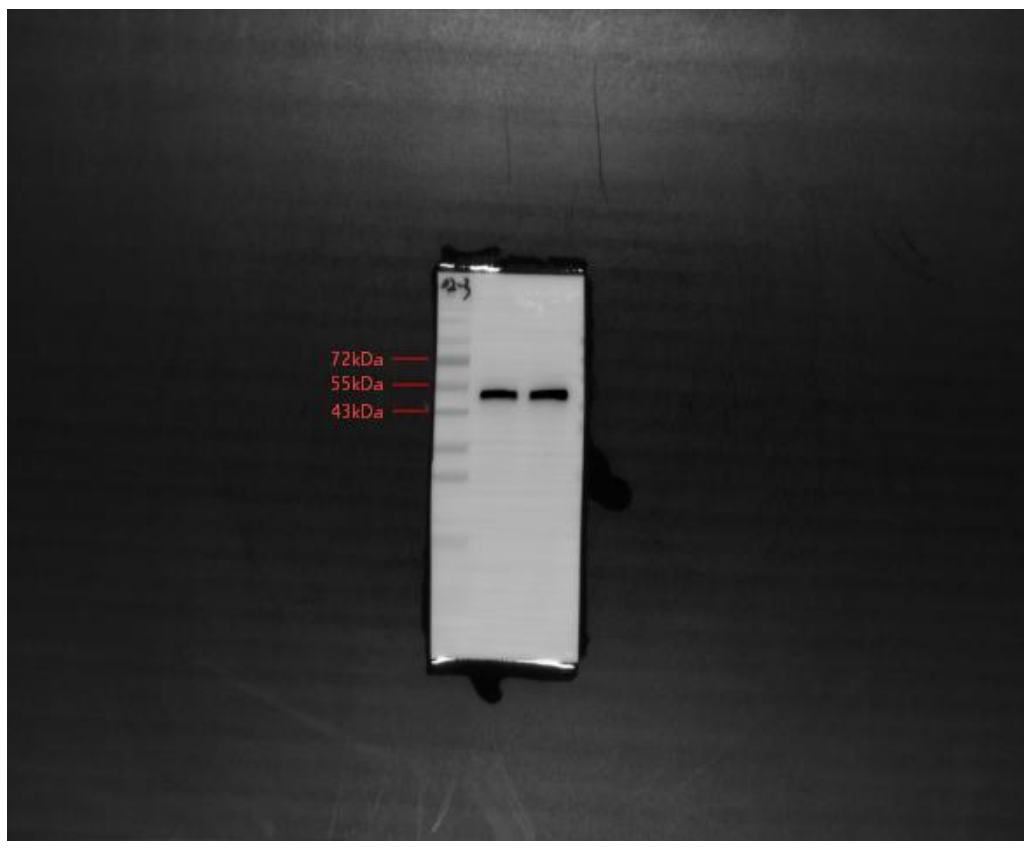

ENO1

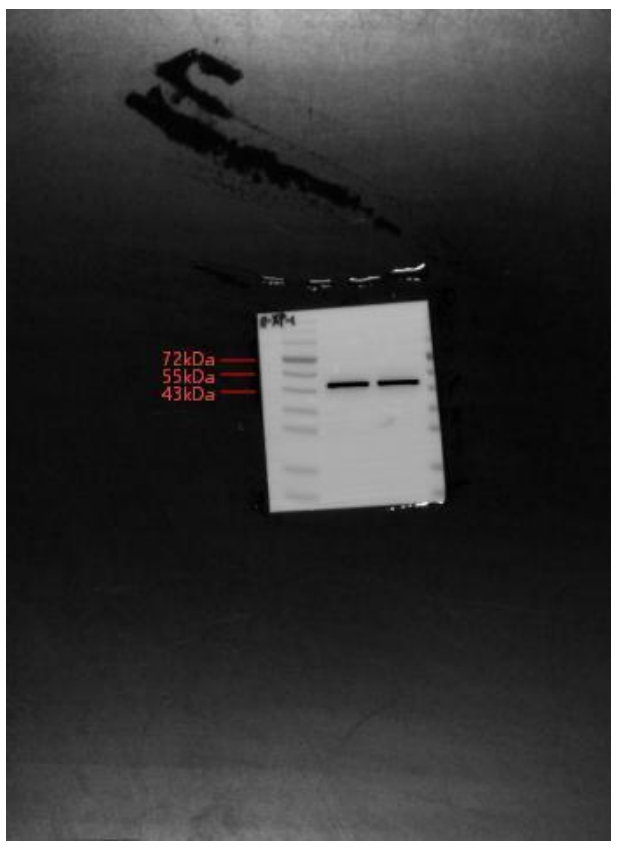

ACTIN

U251R

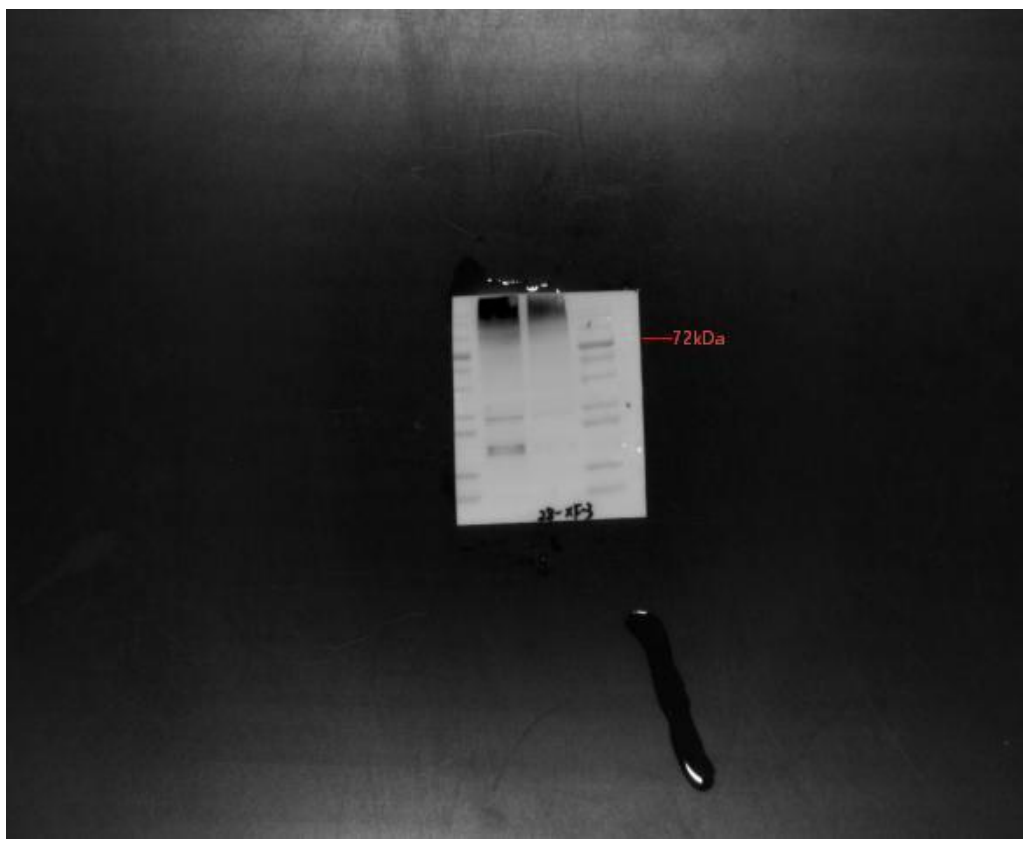

Ub

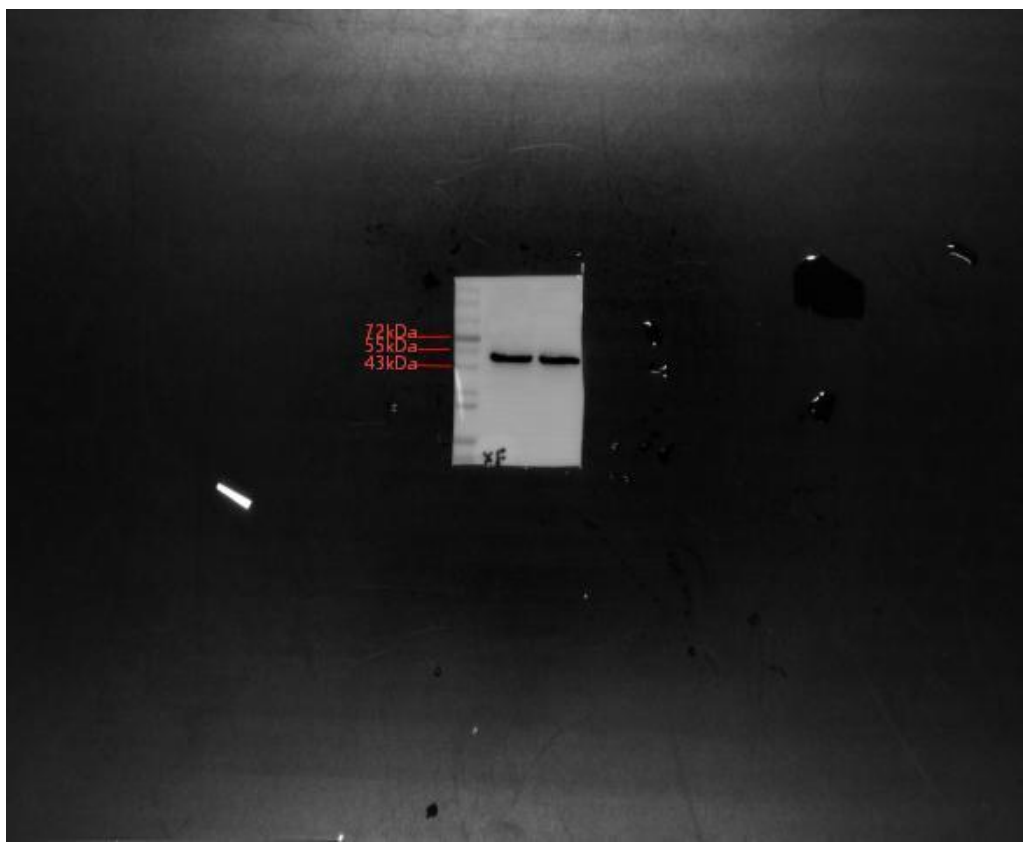

ENO1

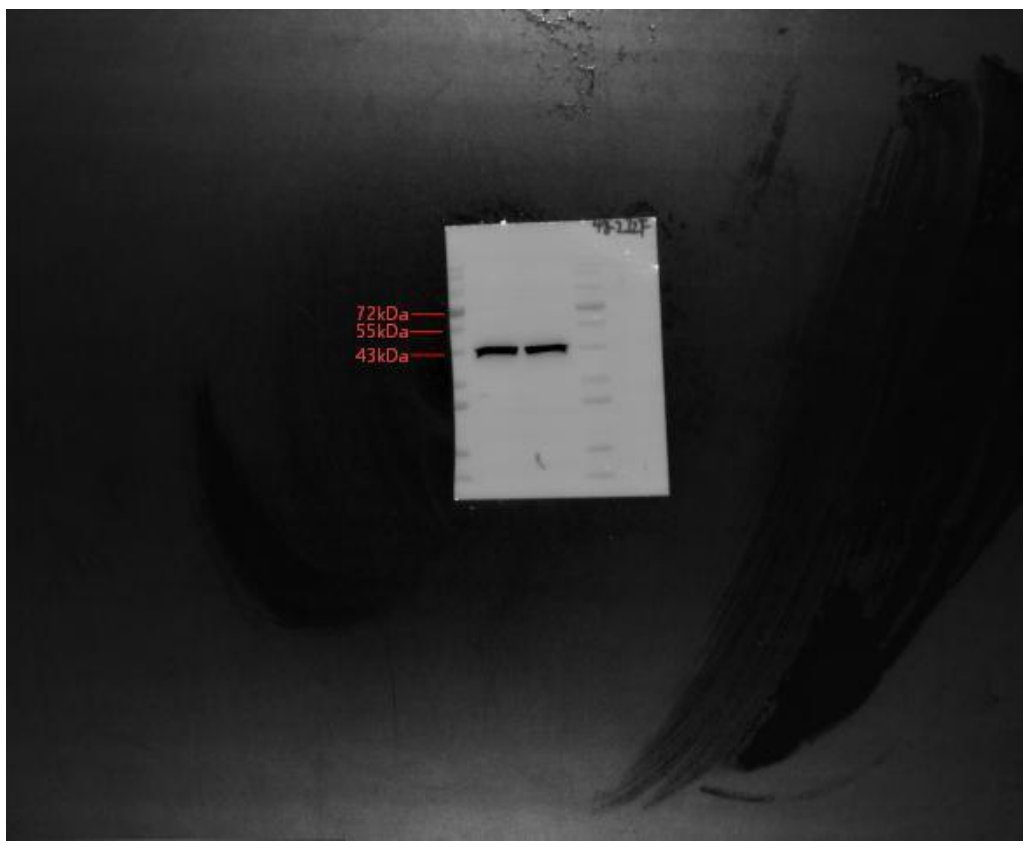

ACTIN

FIG.4K

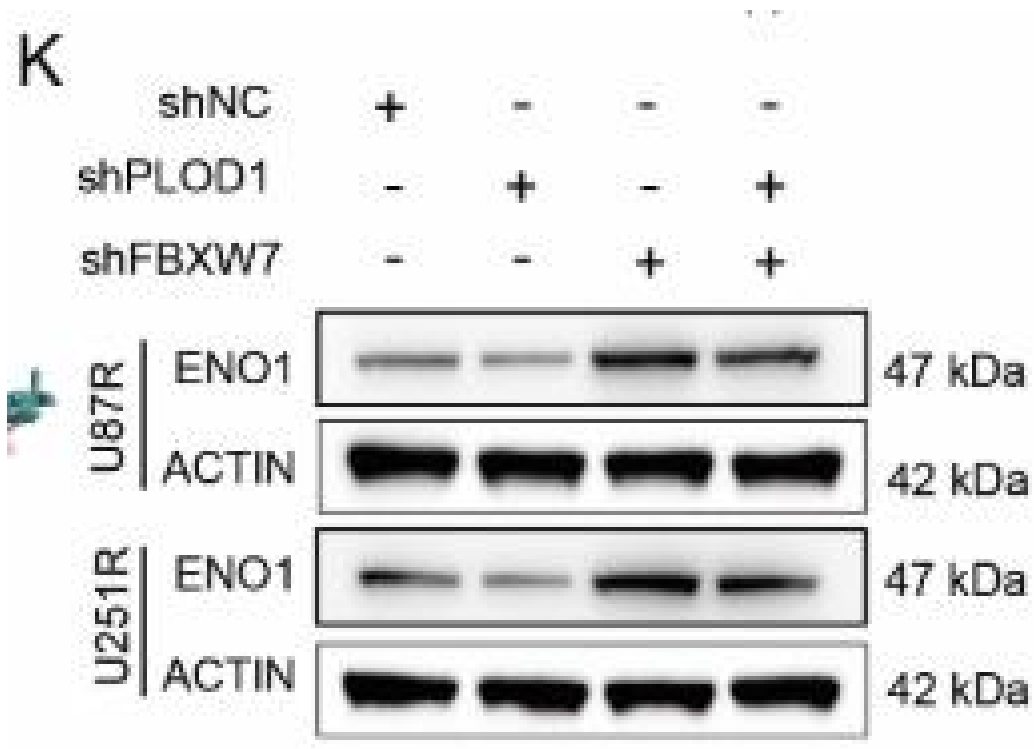

U87R

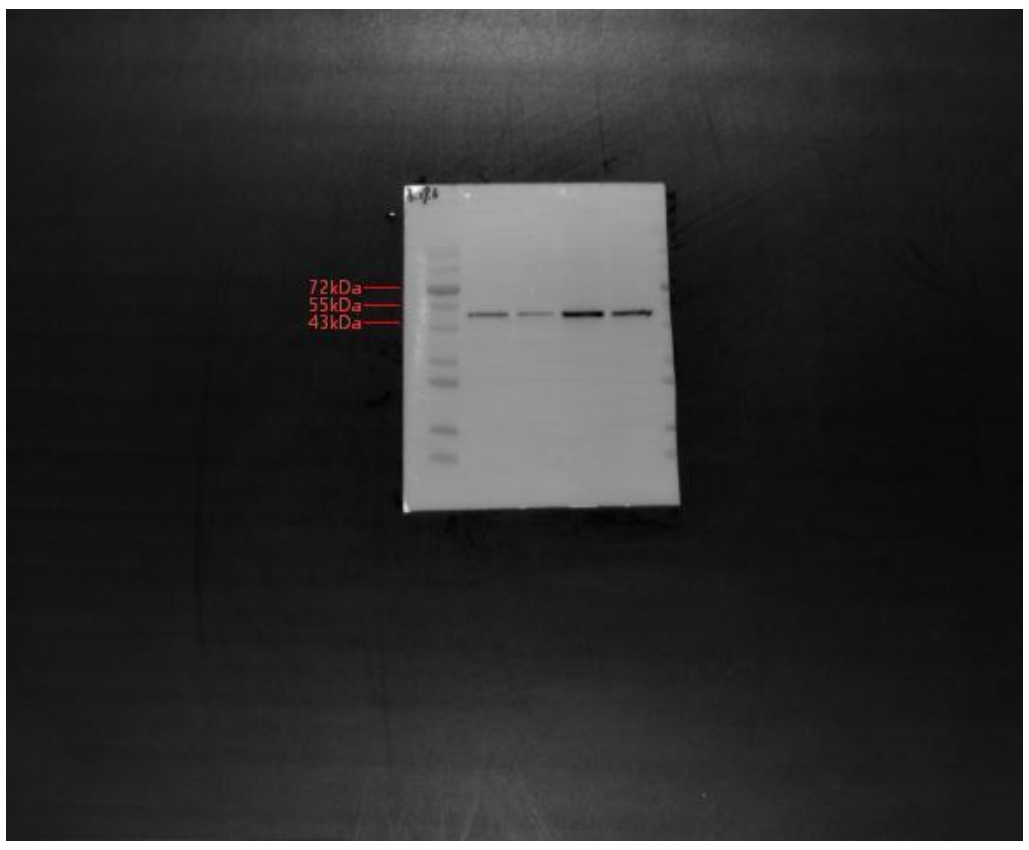

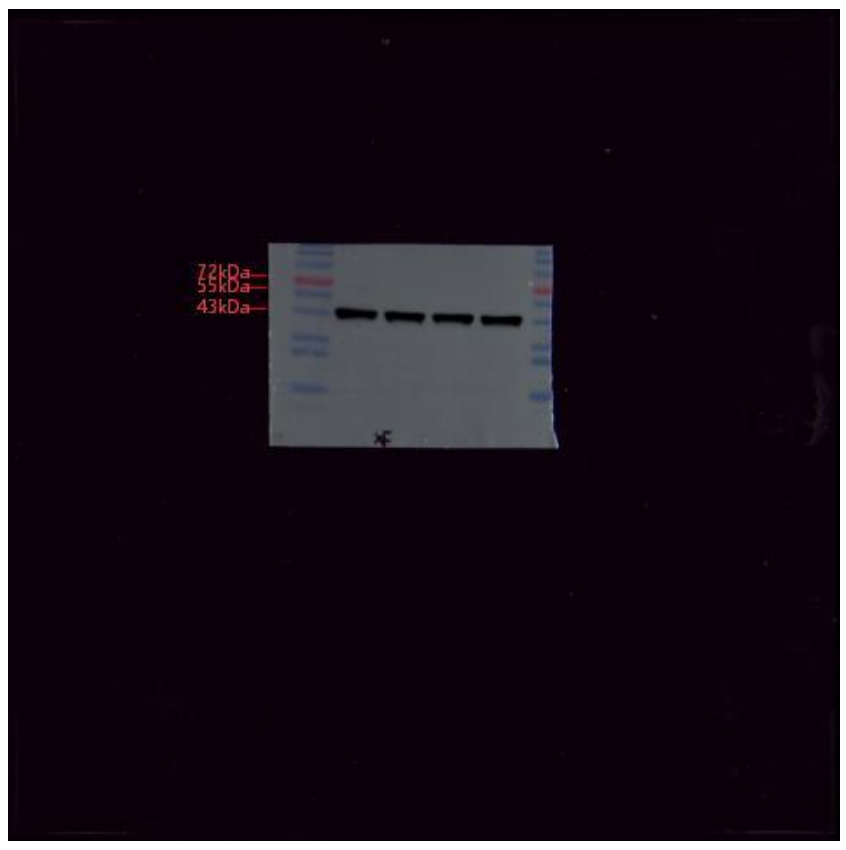

ENO1 ACTIN

U251R

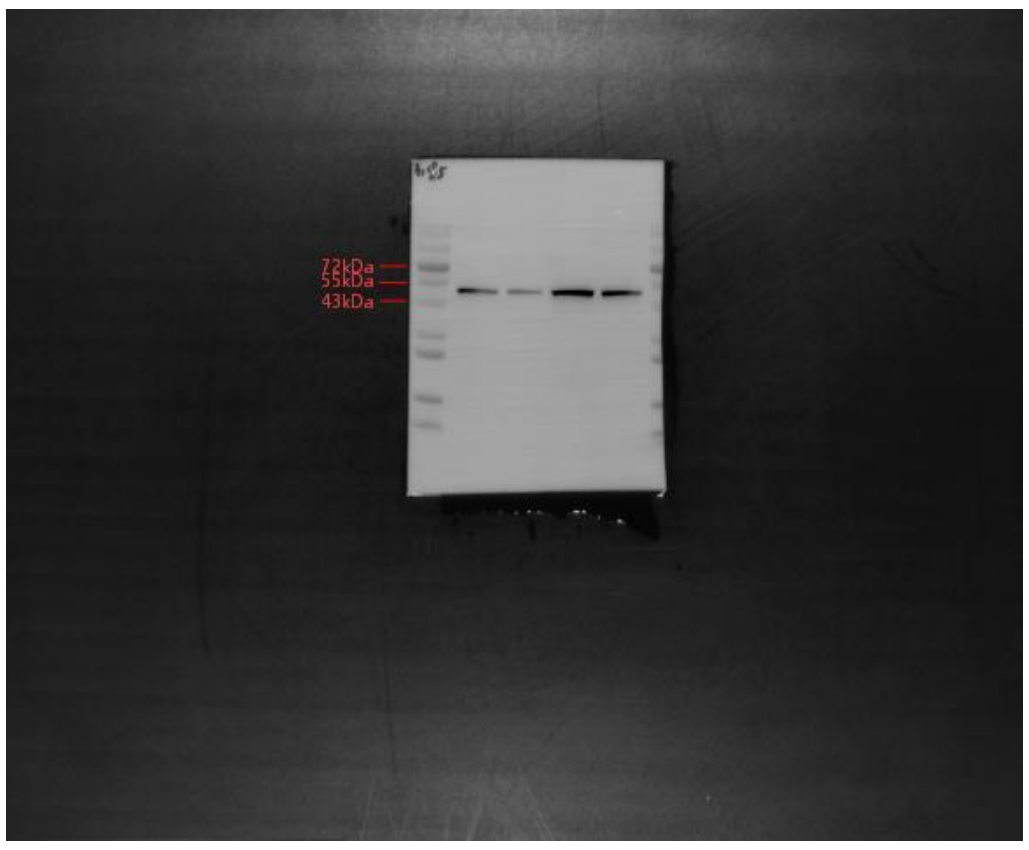

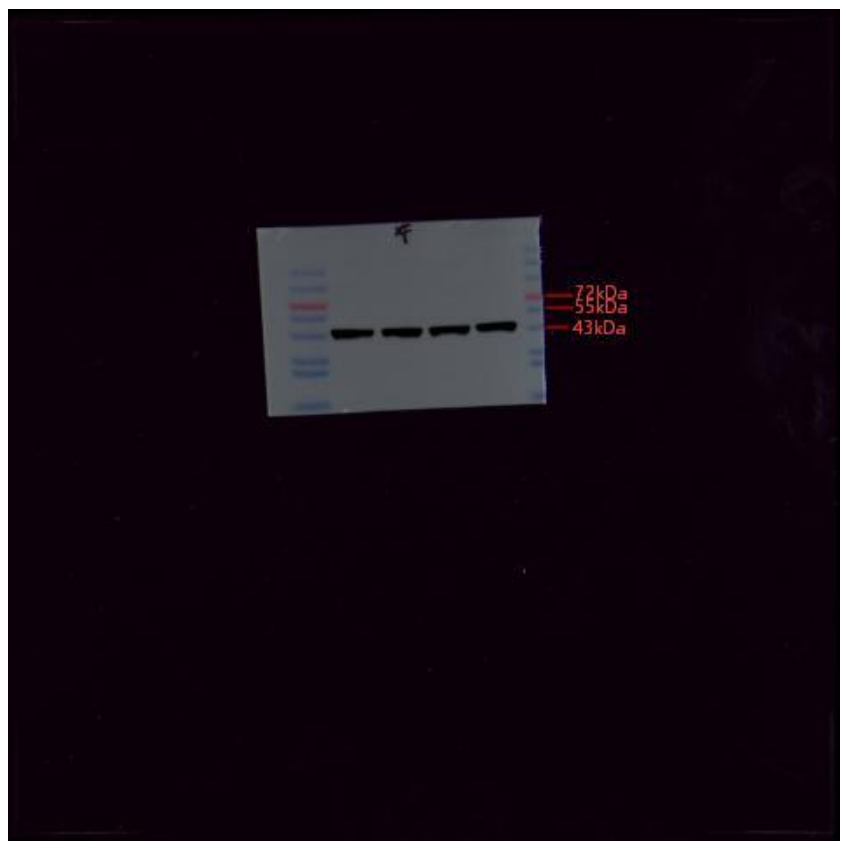

ENO1 ACTIN

FIG.4L

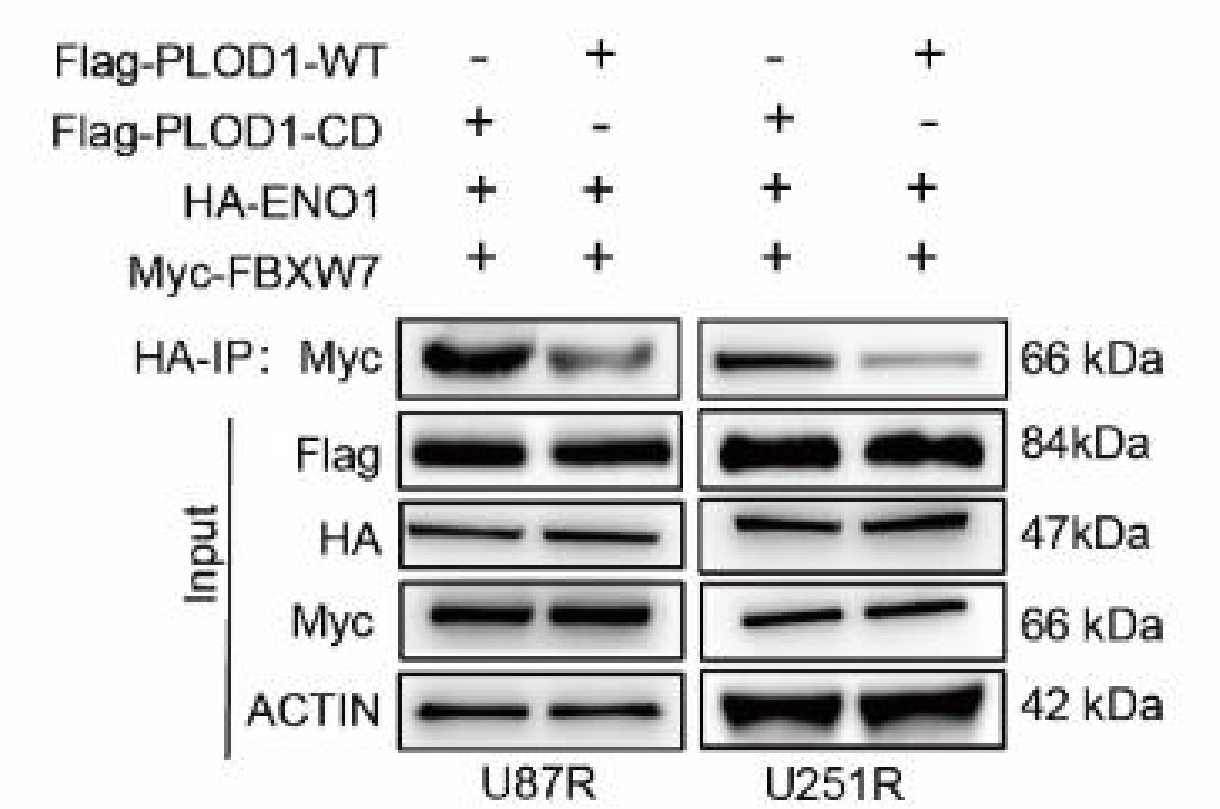

U87R

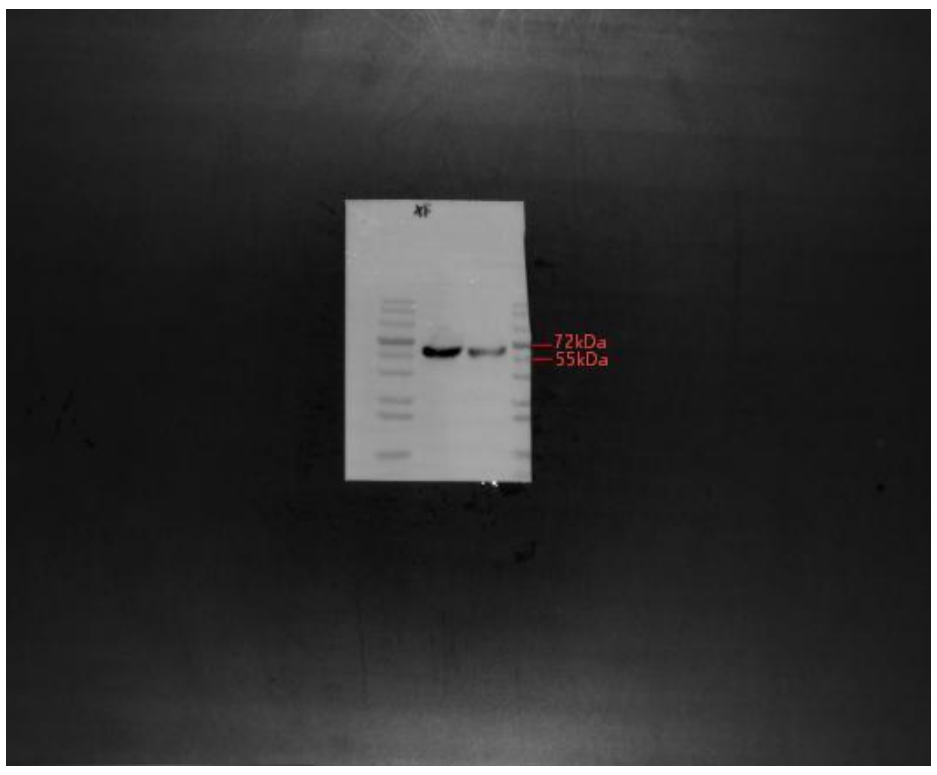

FBXW7

INPUT

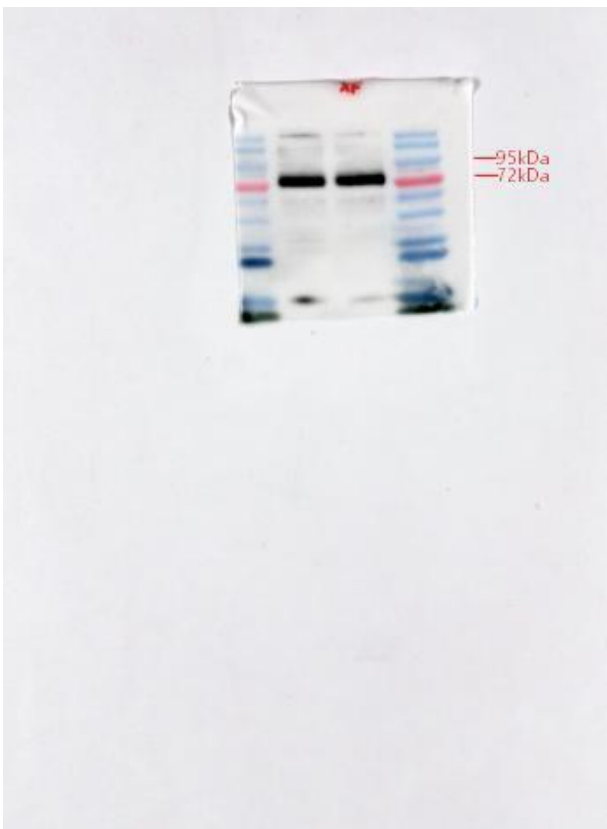

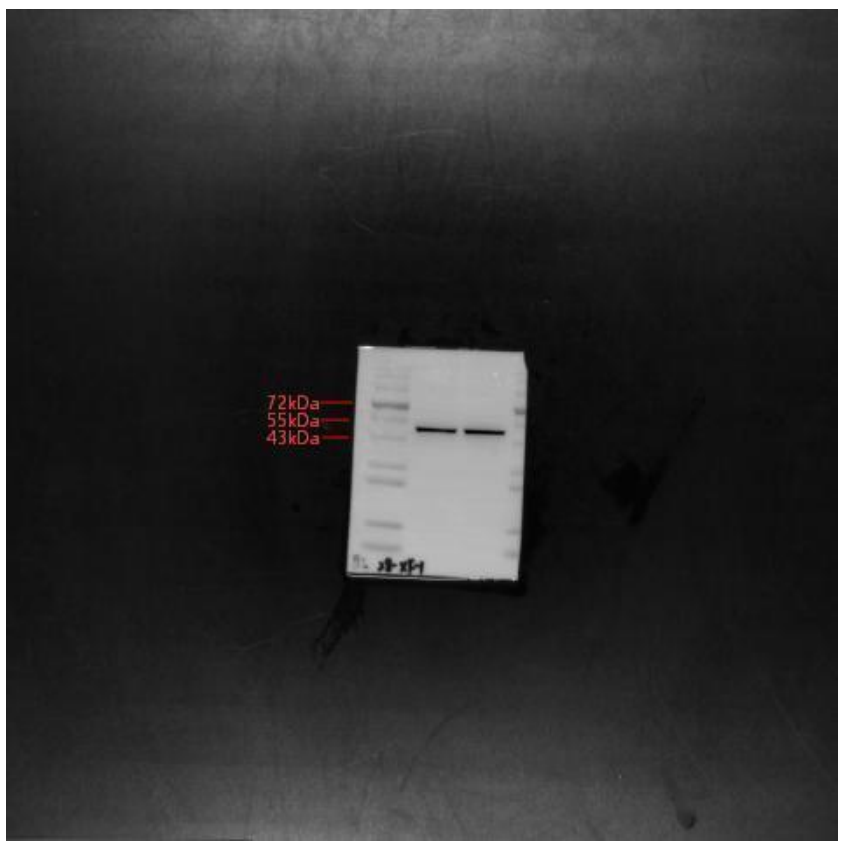

PLOD1

ENO1

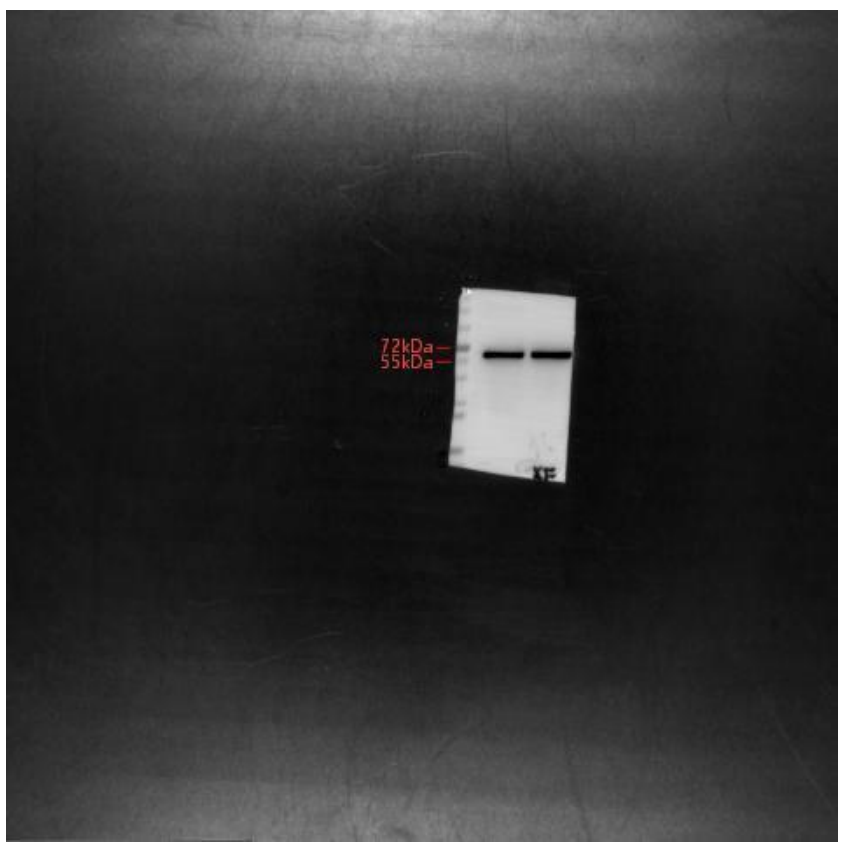

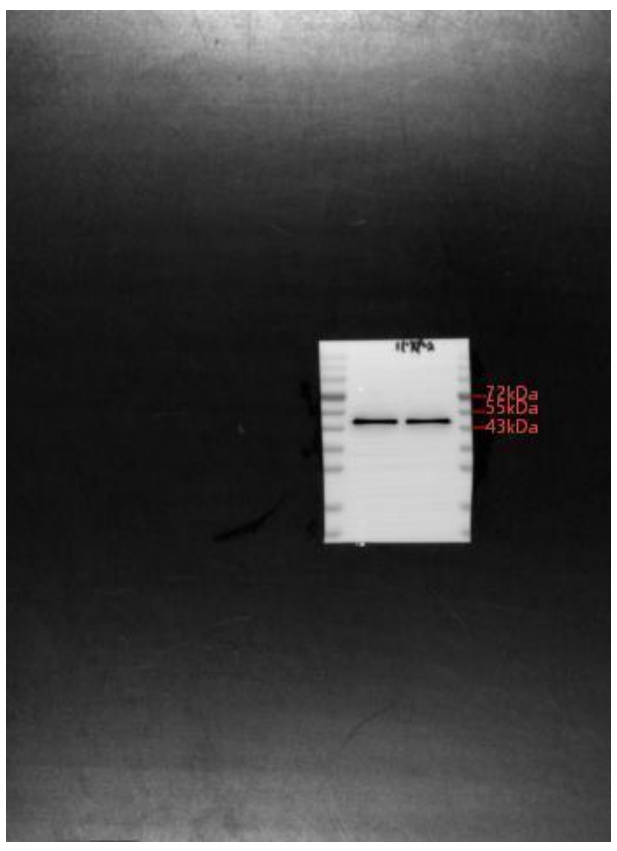

FBXW7 ACTIN

U251R

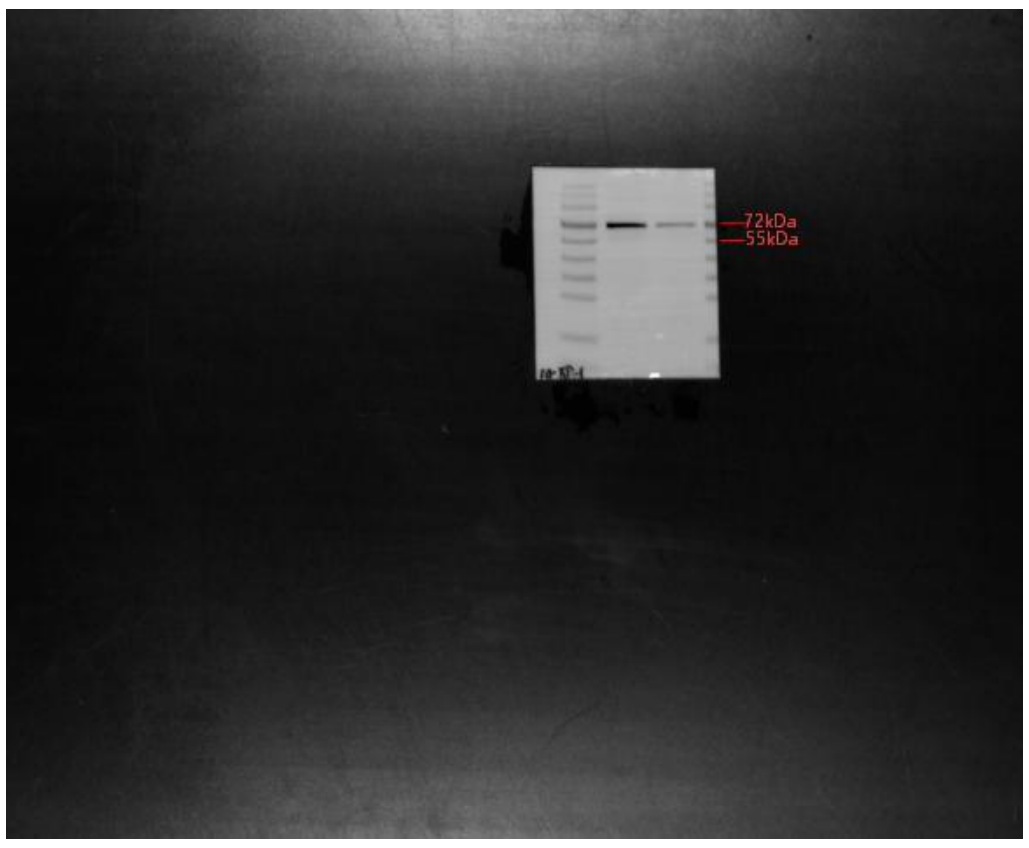

FBXW7

INPUT

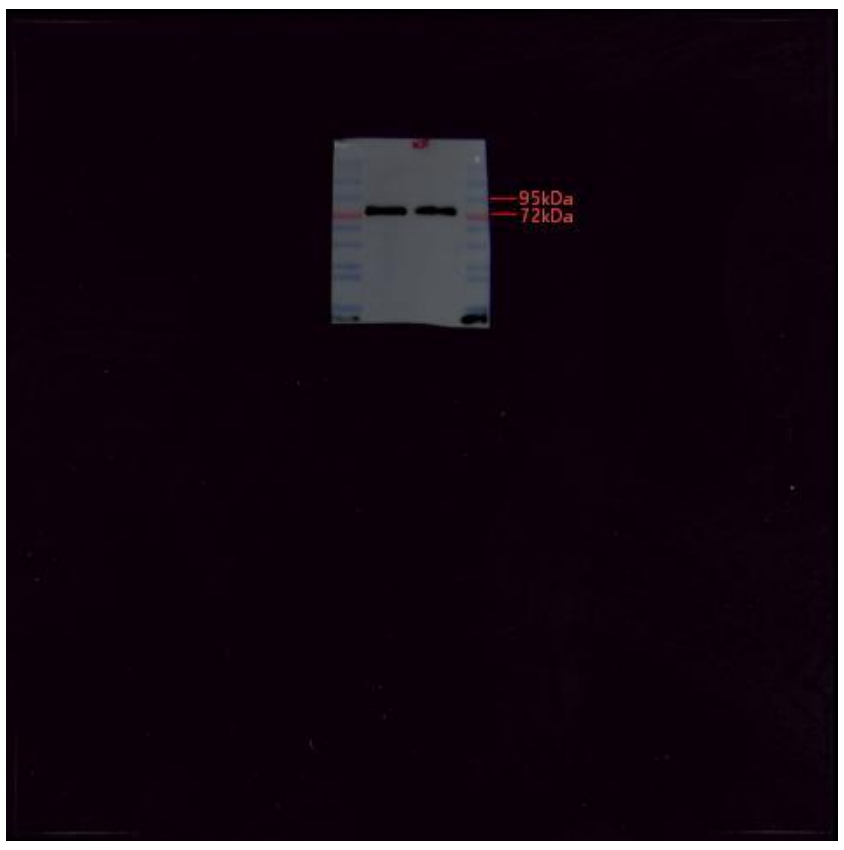

PLOD1

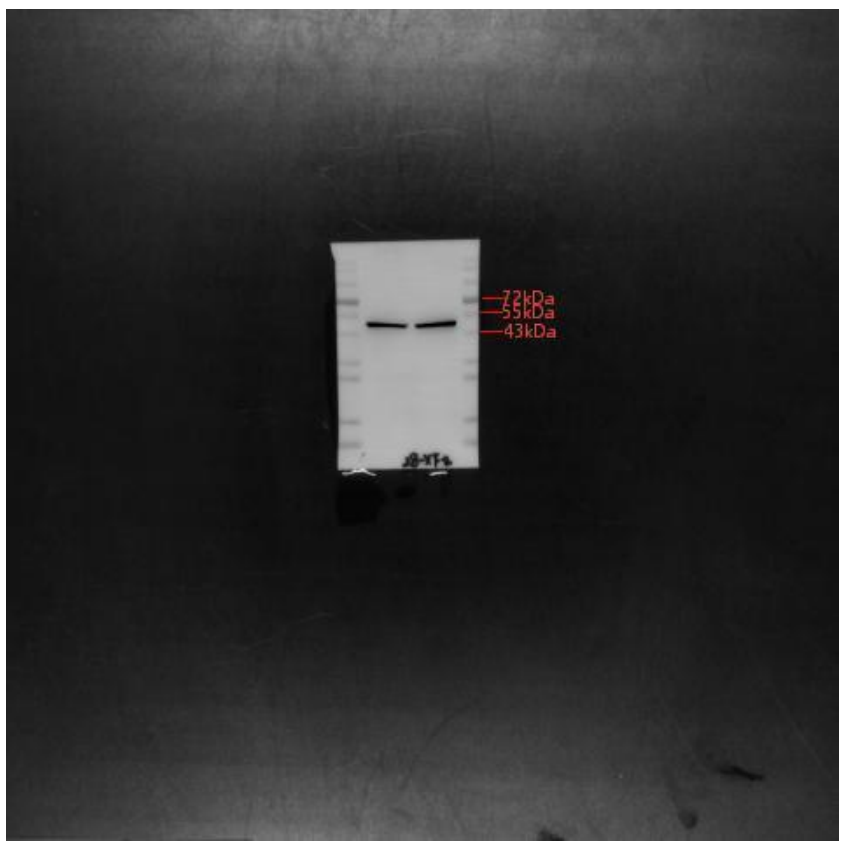

ENO1

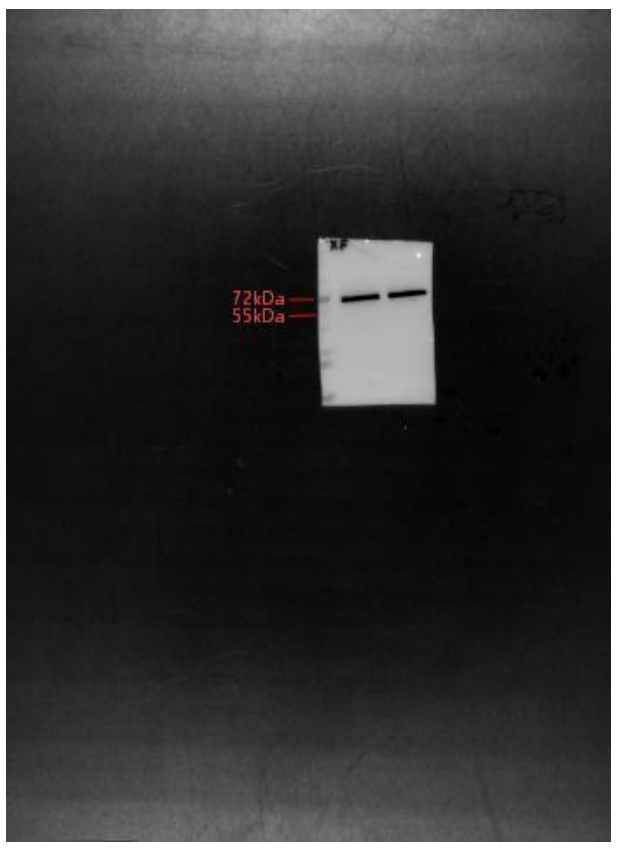

FBXW7

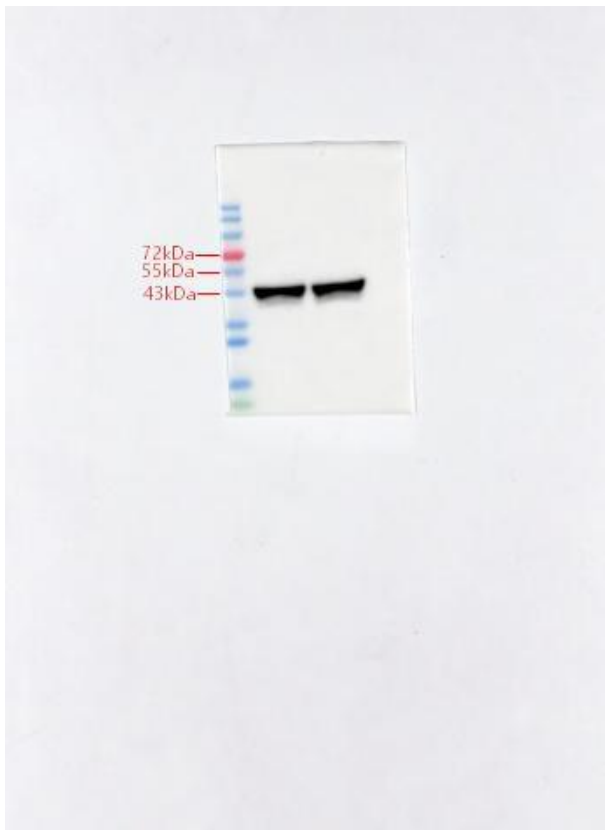

ACTIN

FIG.4M

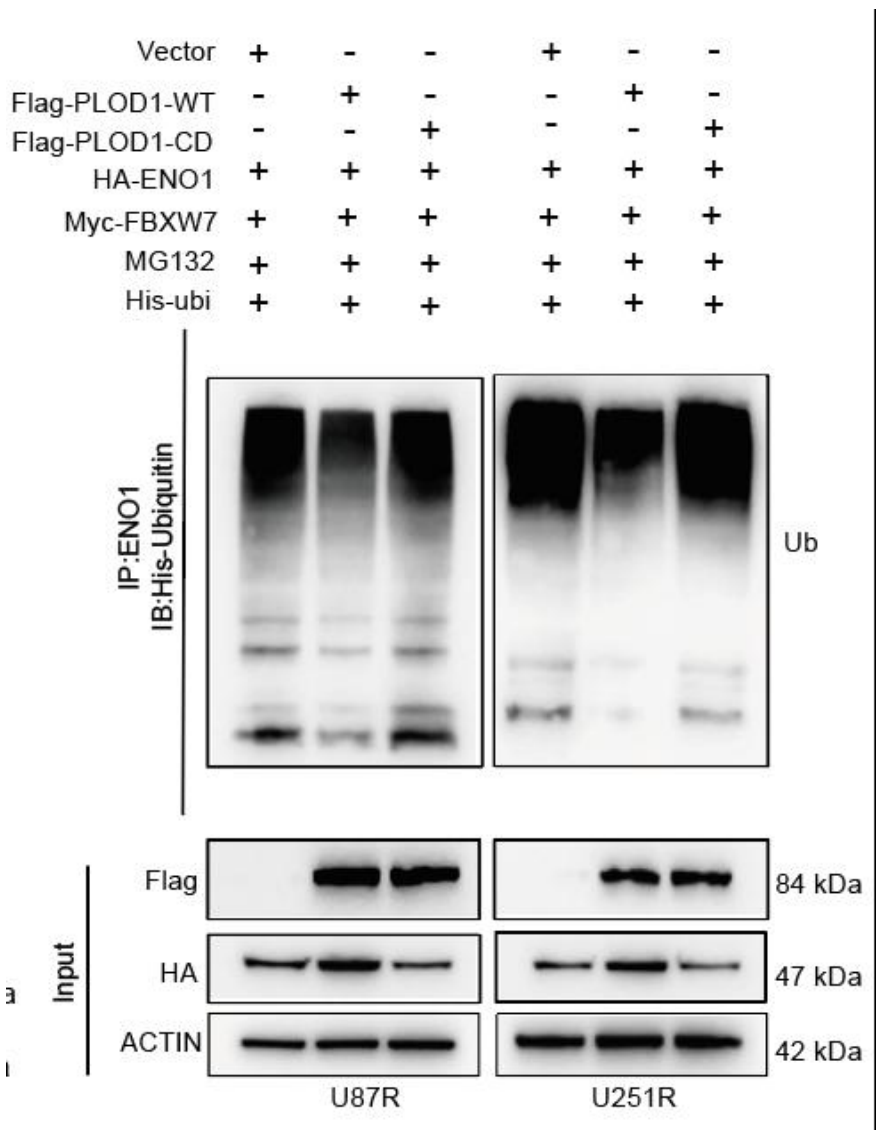

U87R

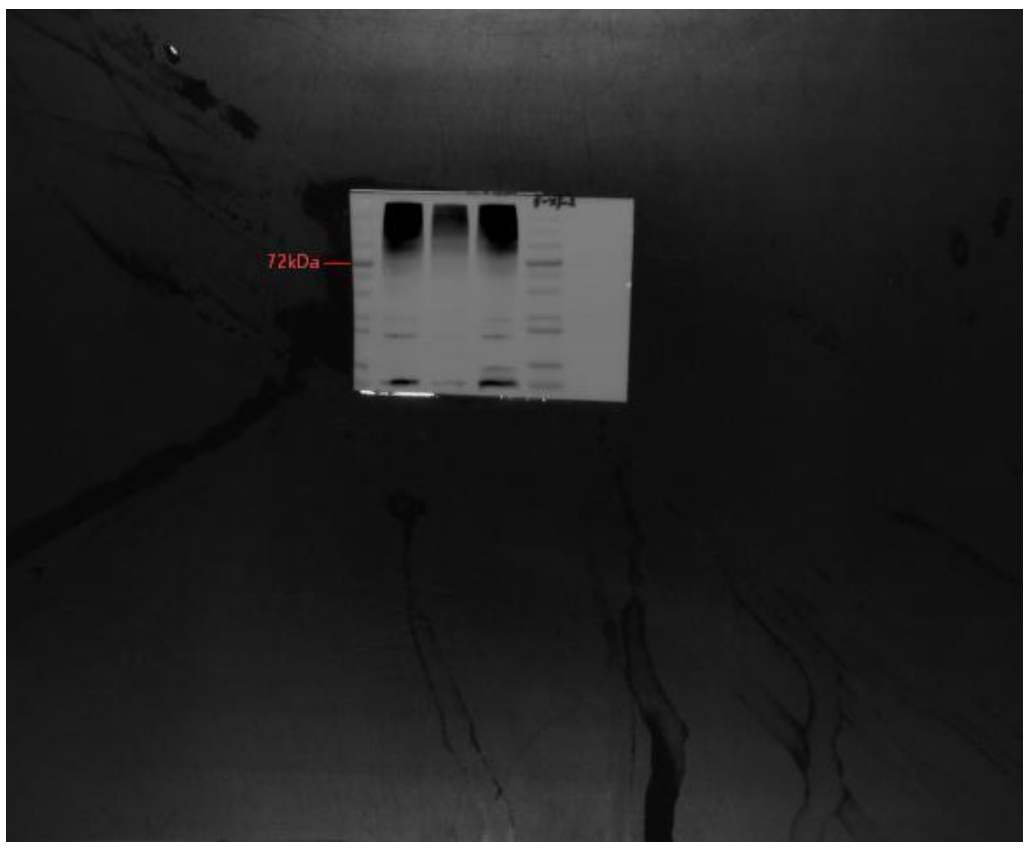

Ub

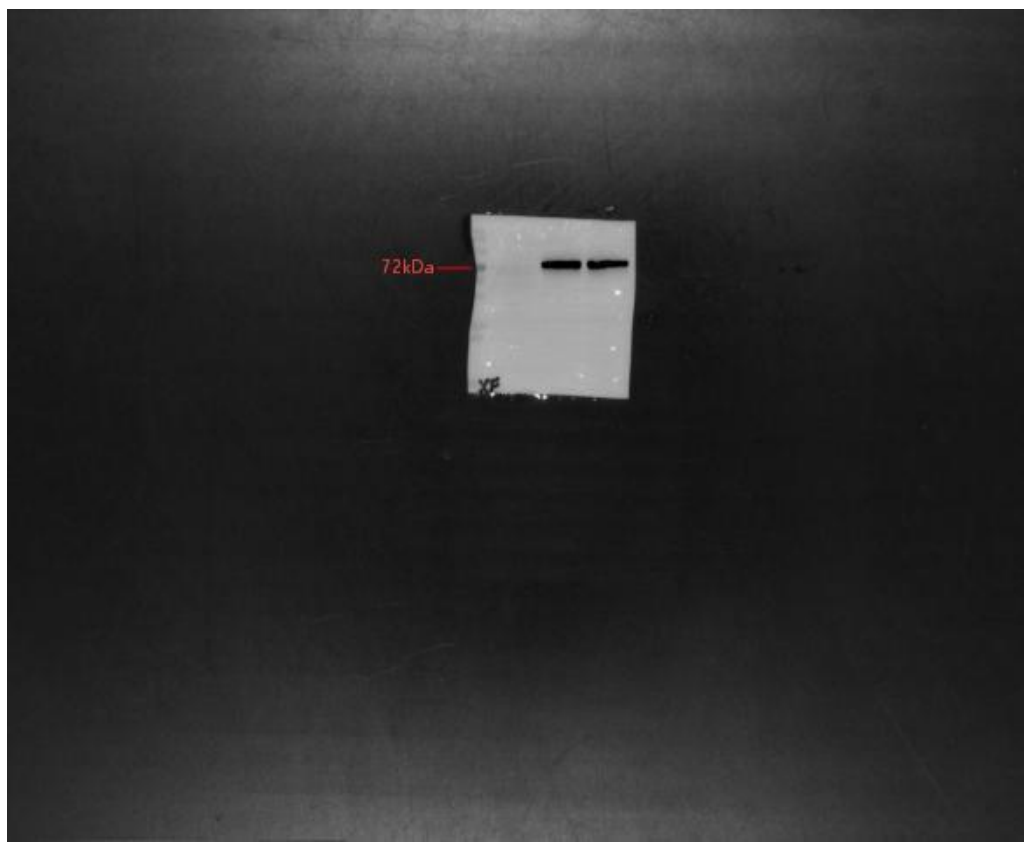

Flag

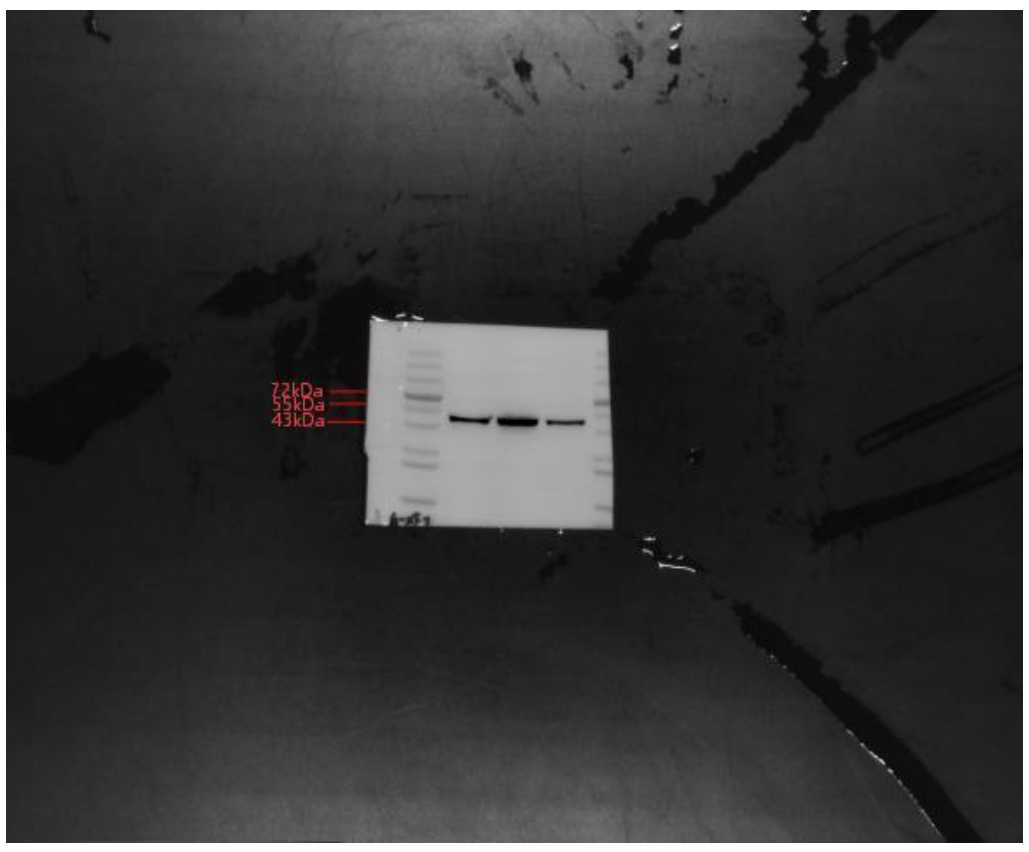

HA

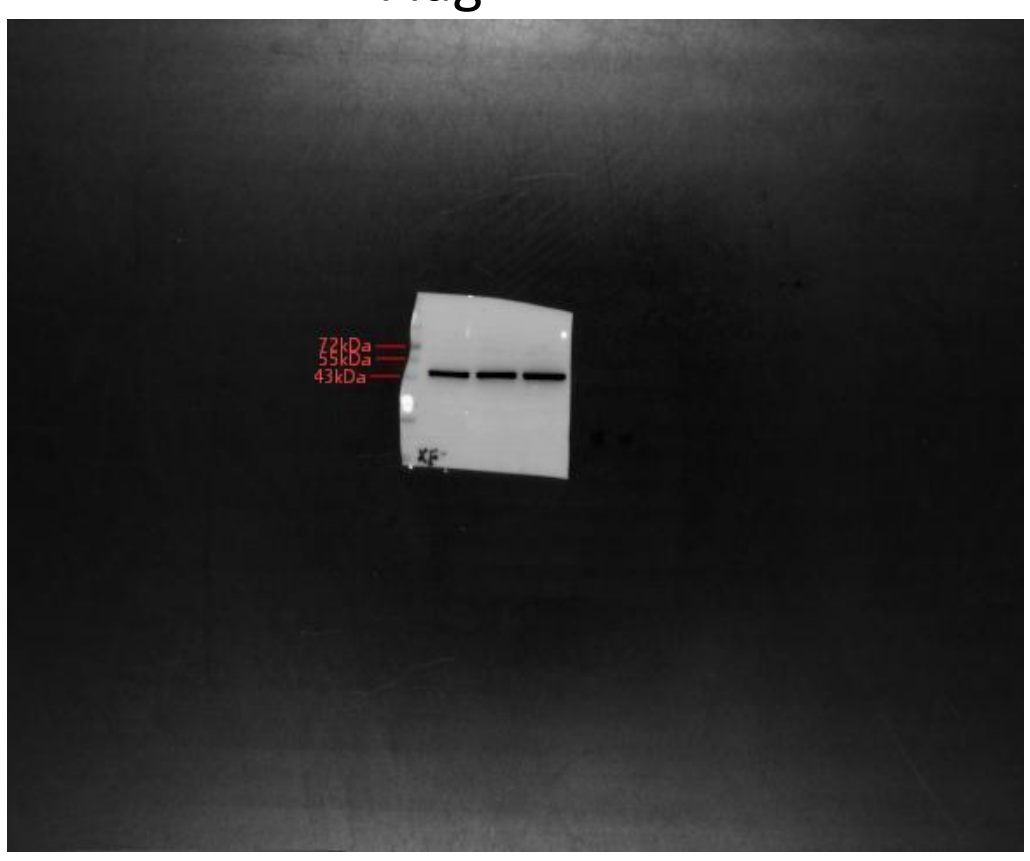

ACTIN

U251R

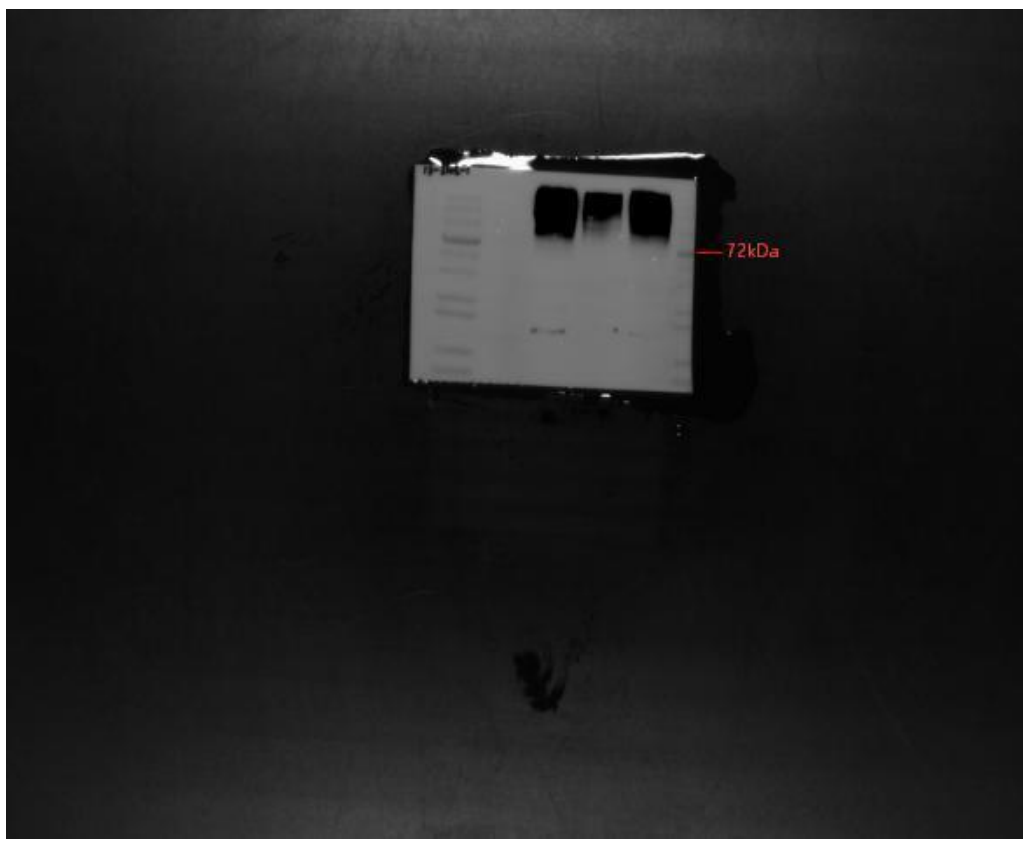

Ub

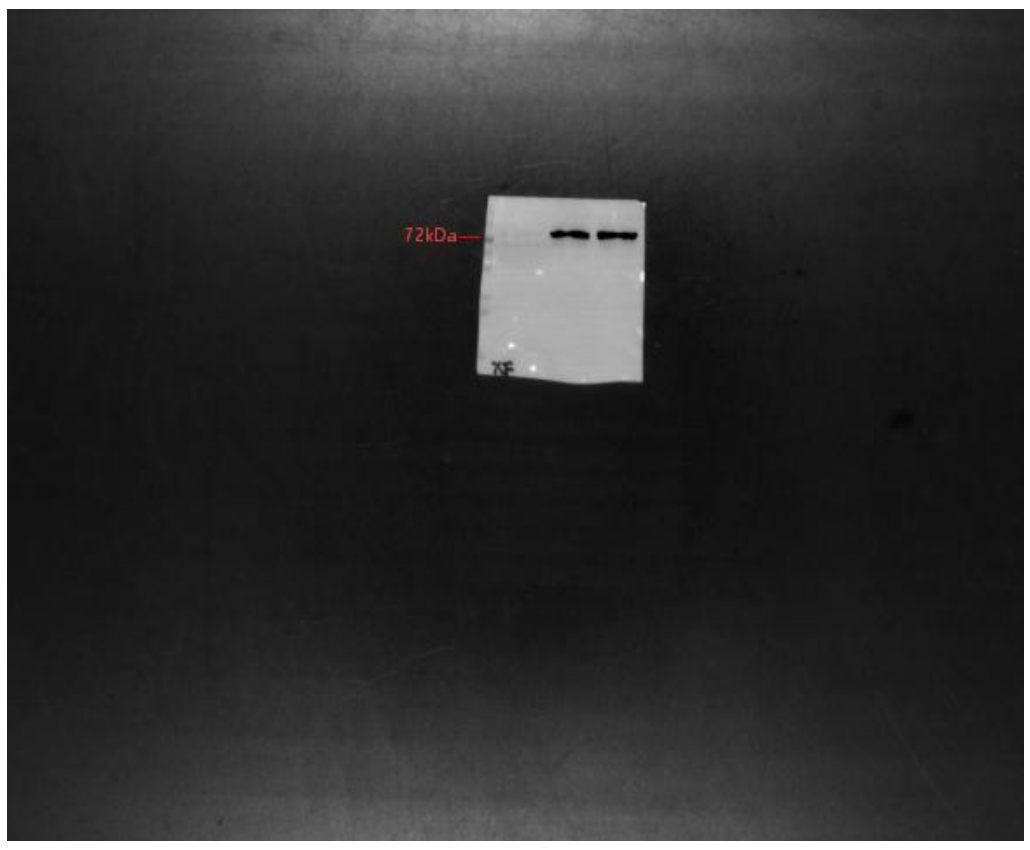

Flag

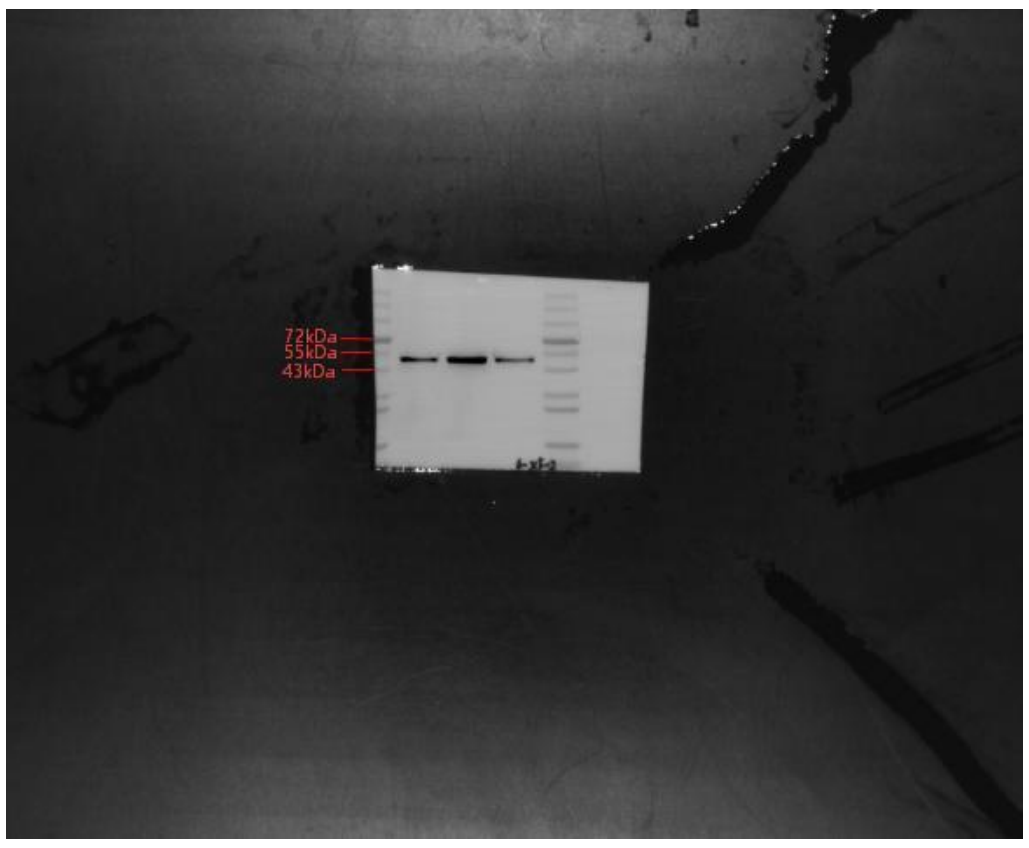

HA

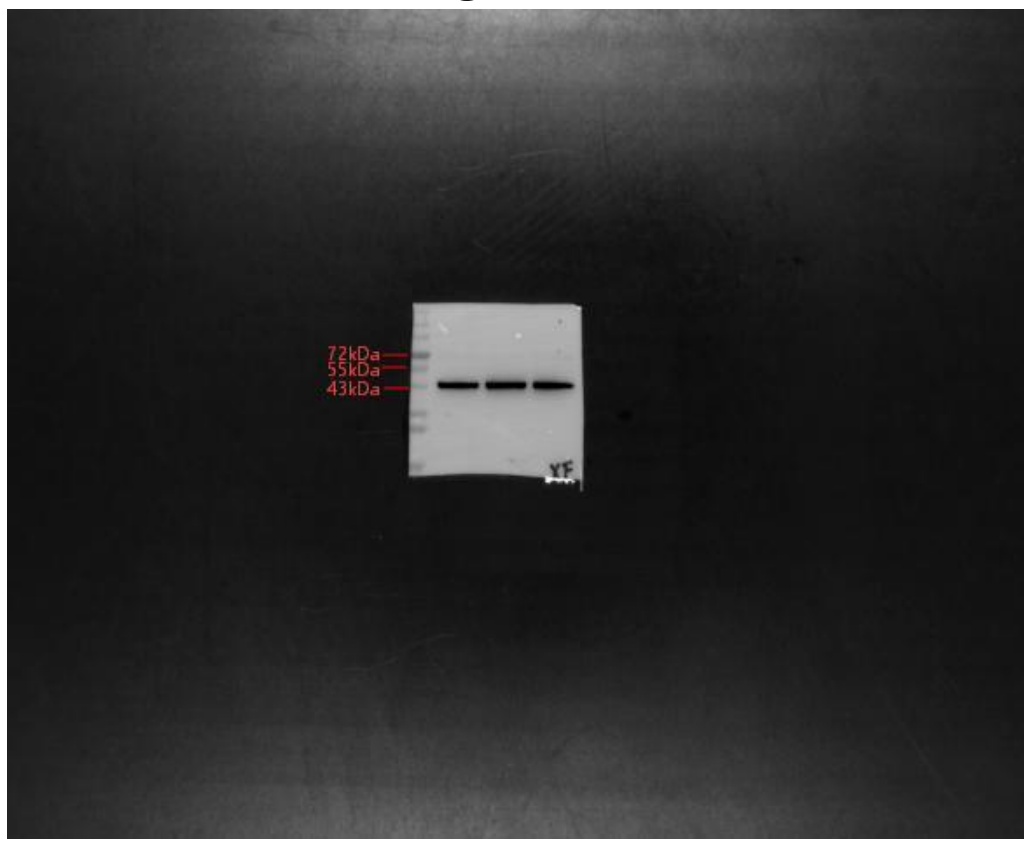

ACTIN

FIG.5C

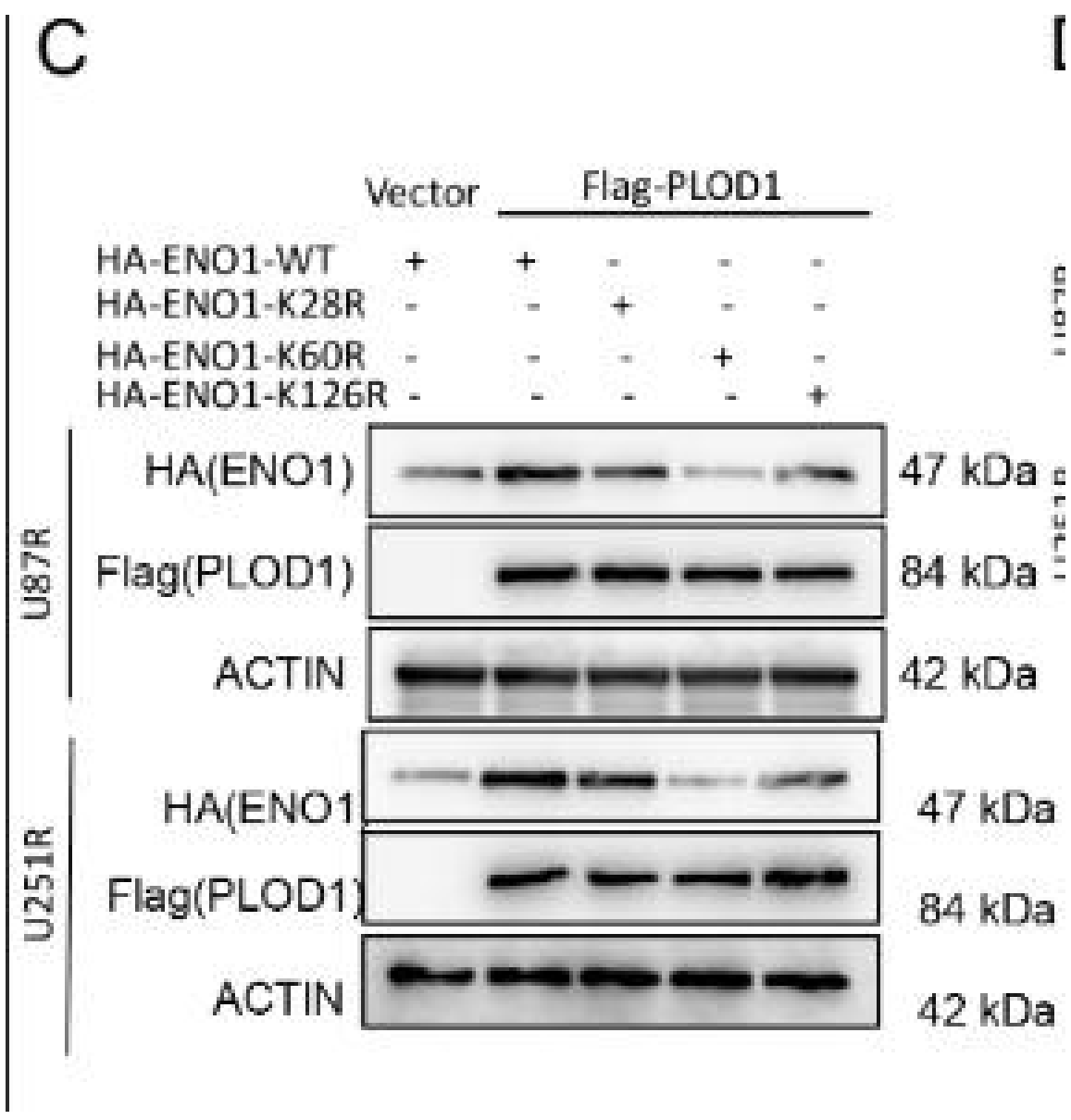

U87R

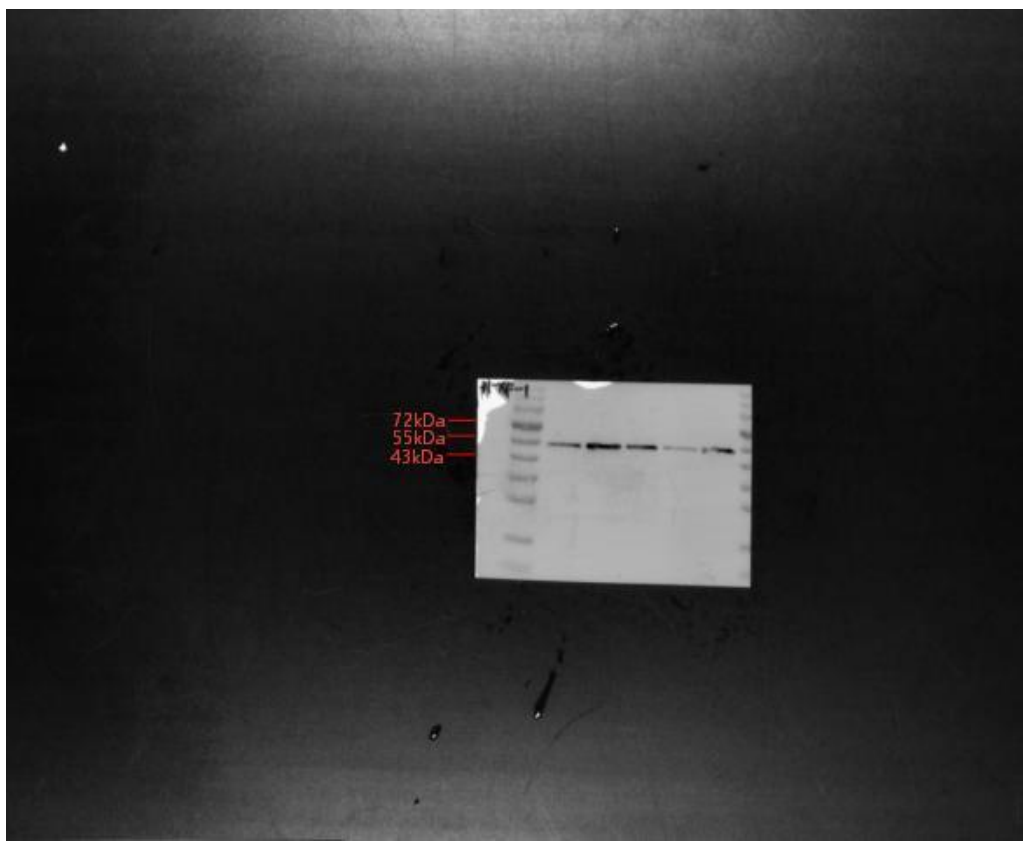

HA(ENO1)

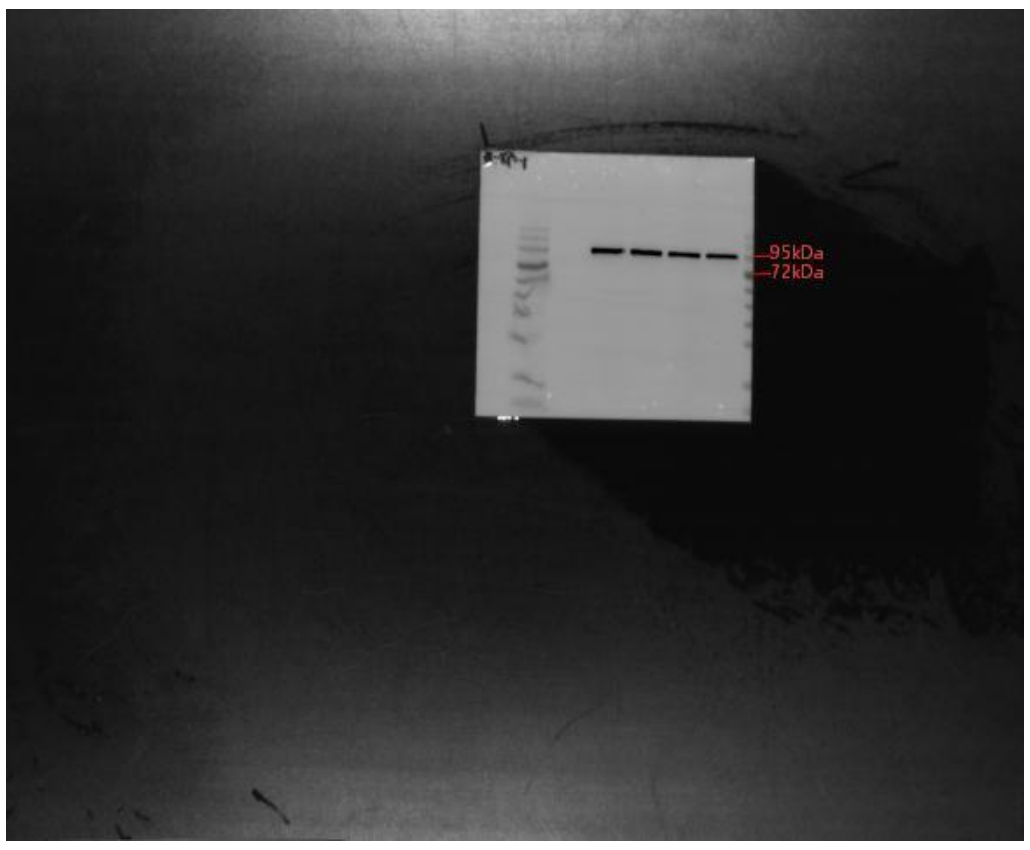

Flag(PLOD1)

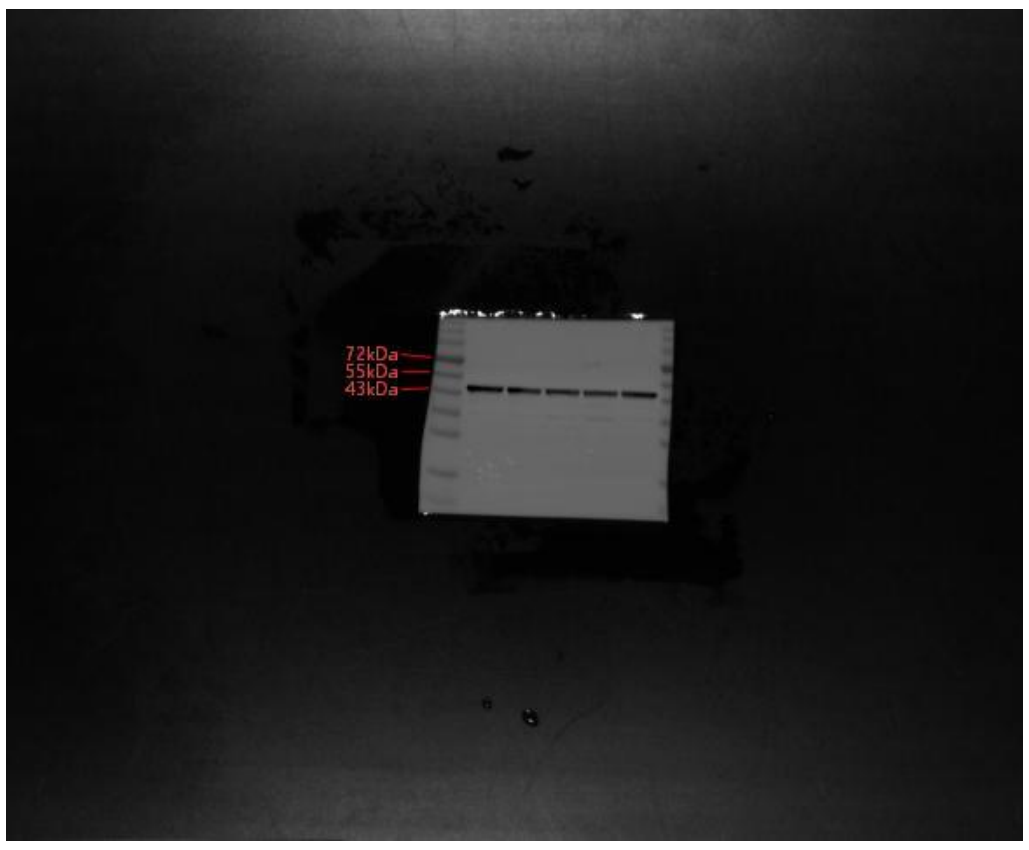

ACTIN

U251R

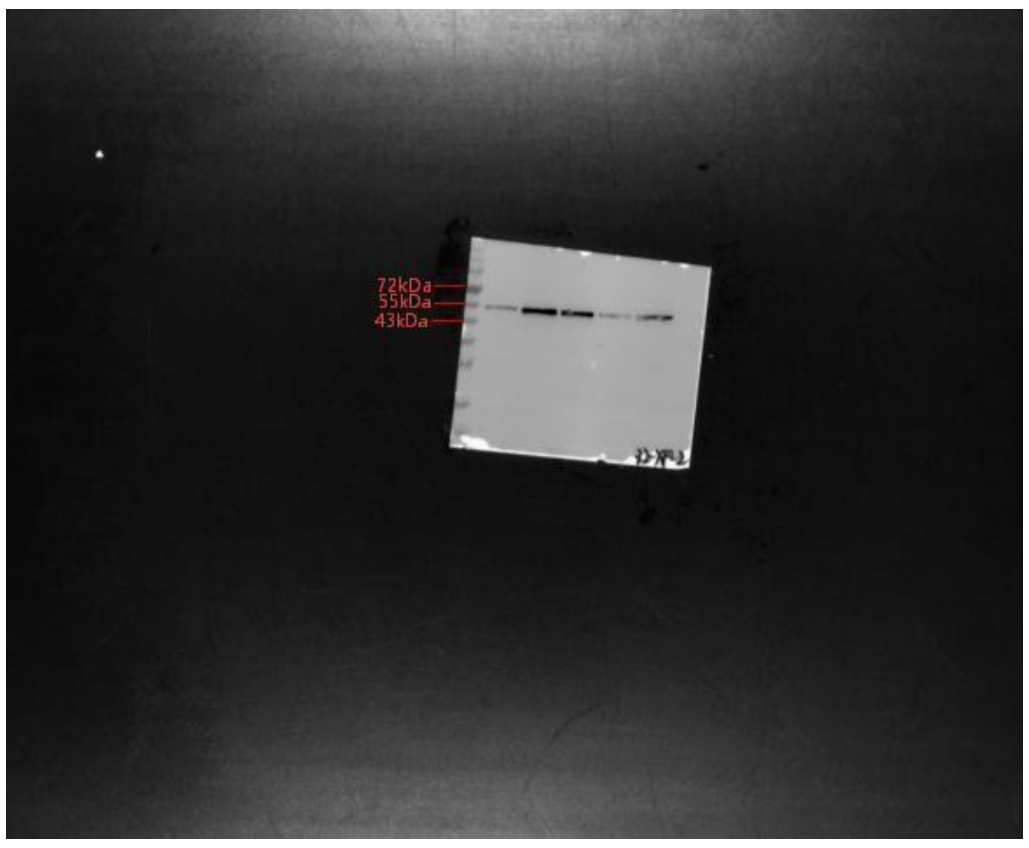

HA(ENO1)

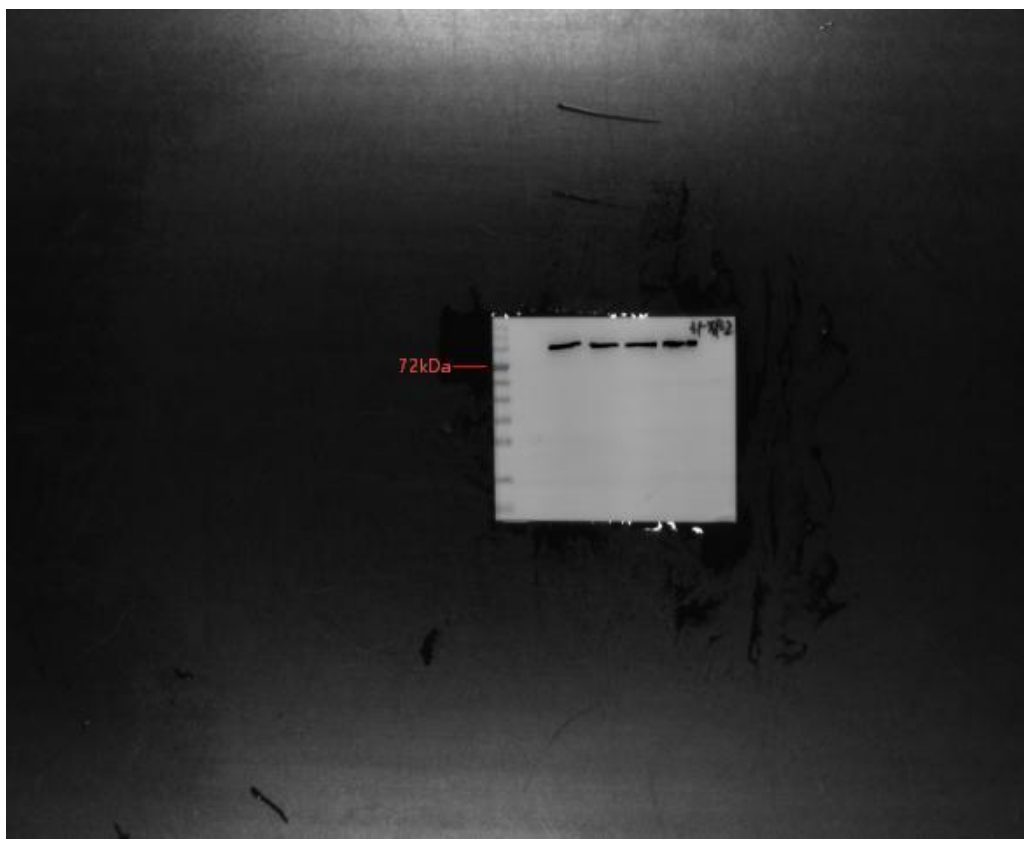

Flag(PLOD1)

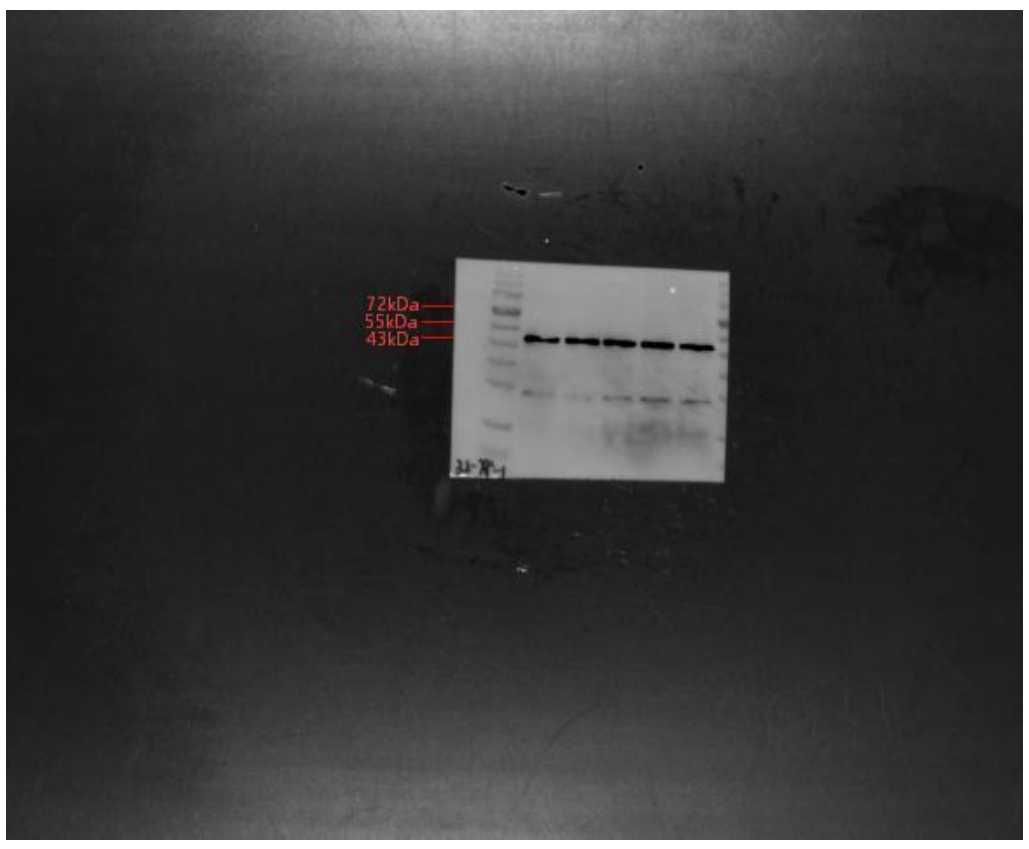

ACTIN

FIG.5D

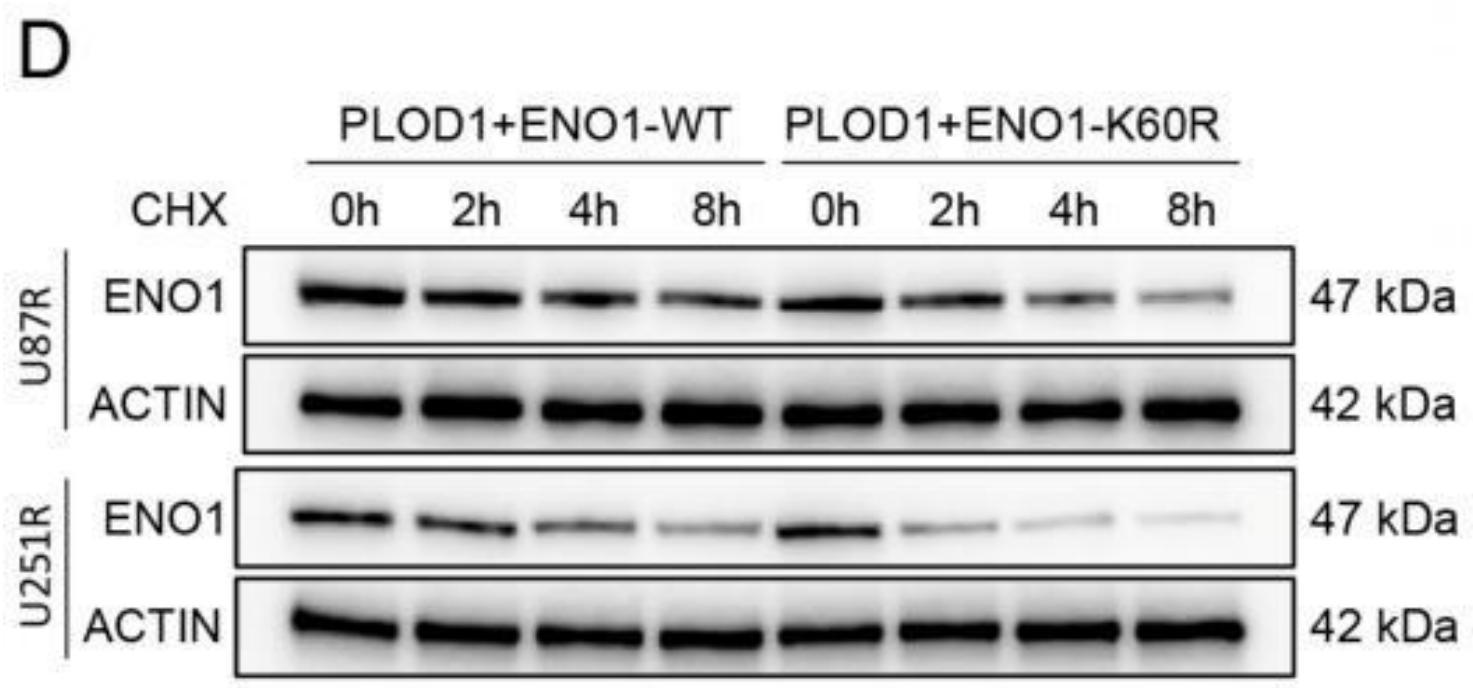

U87R

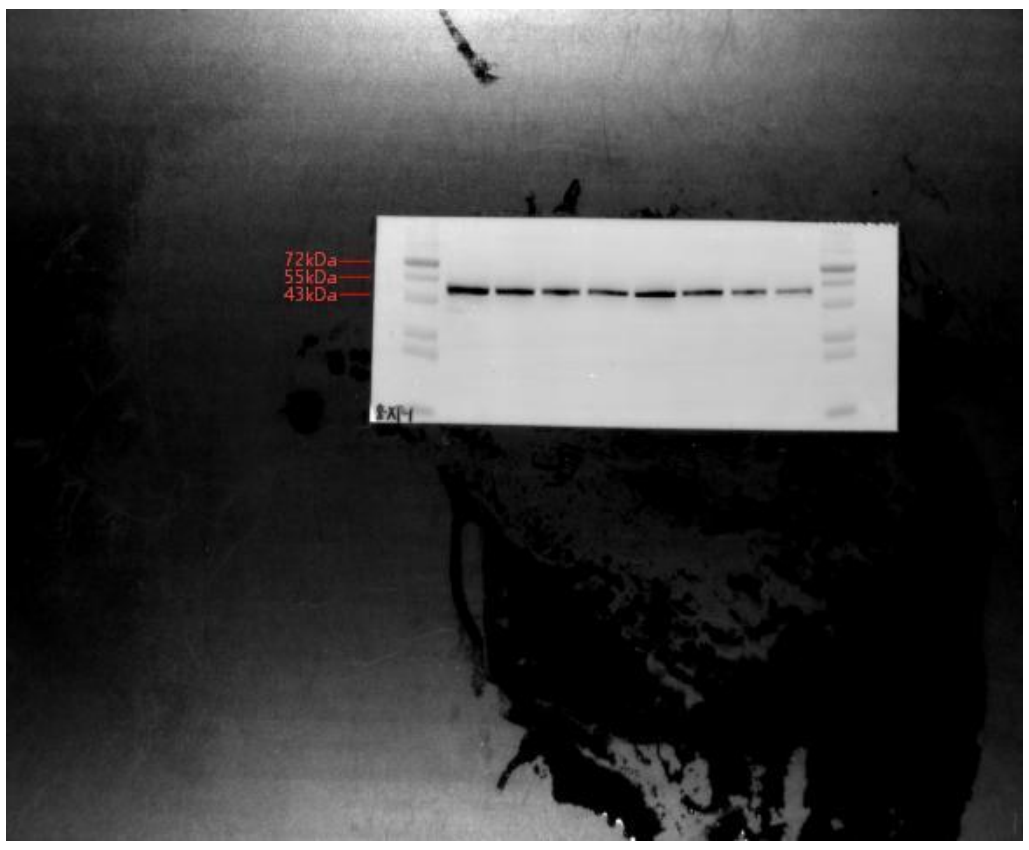

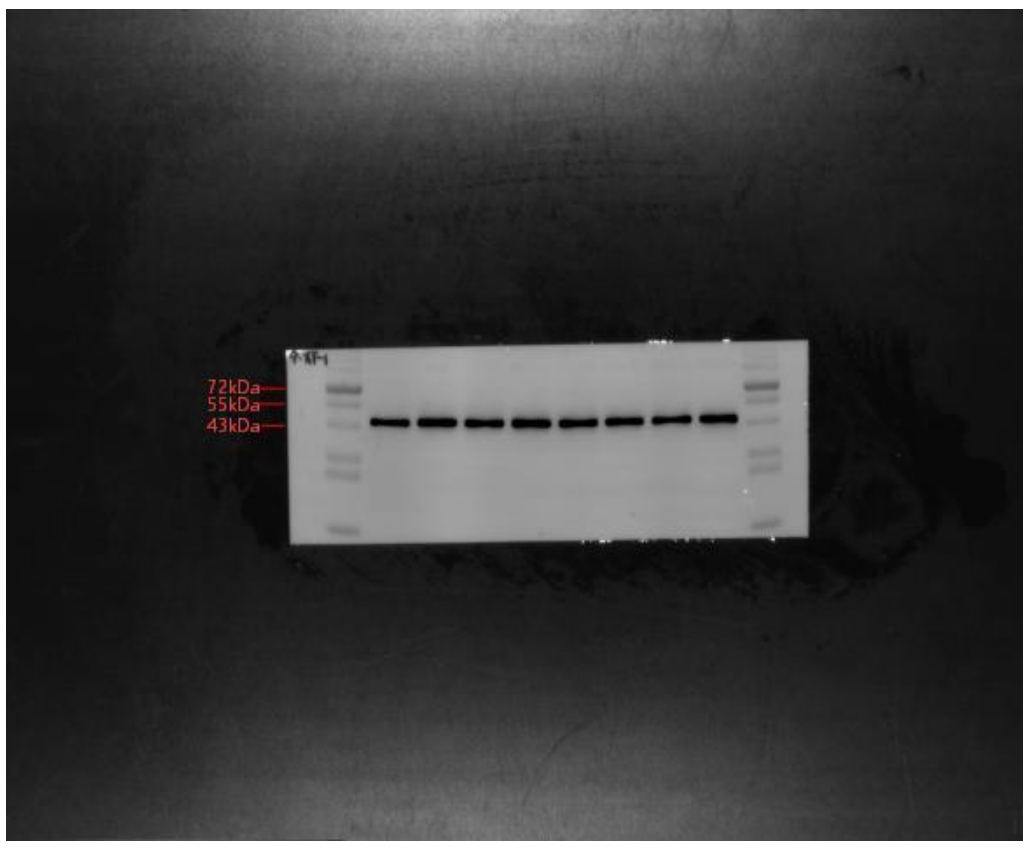

ENO1 ACTIN

U251R

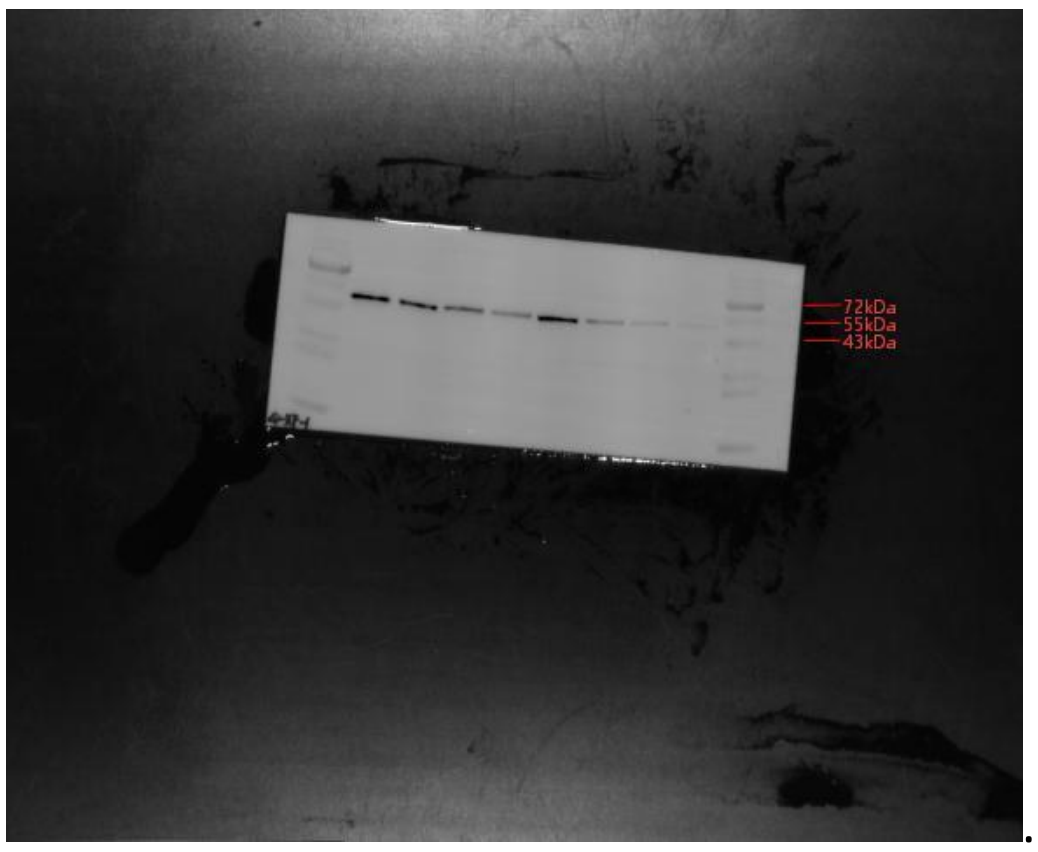

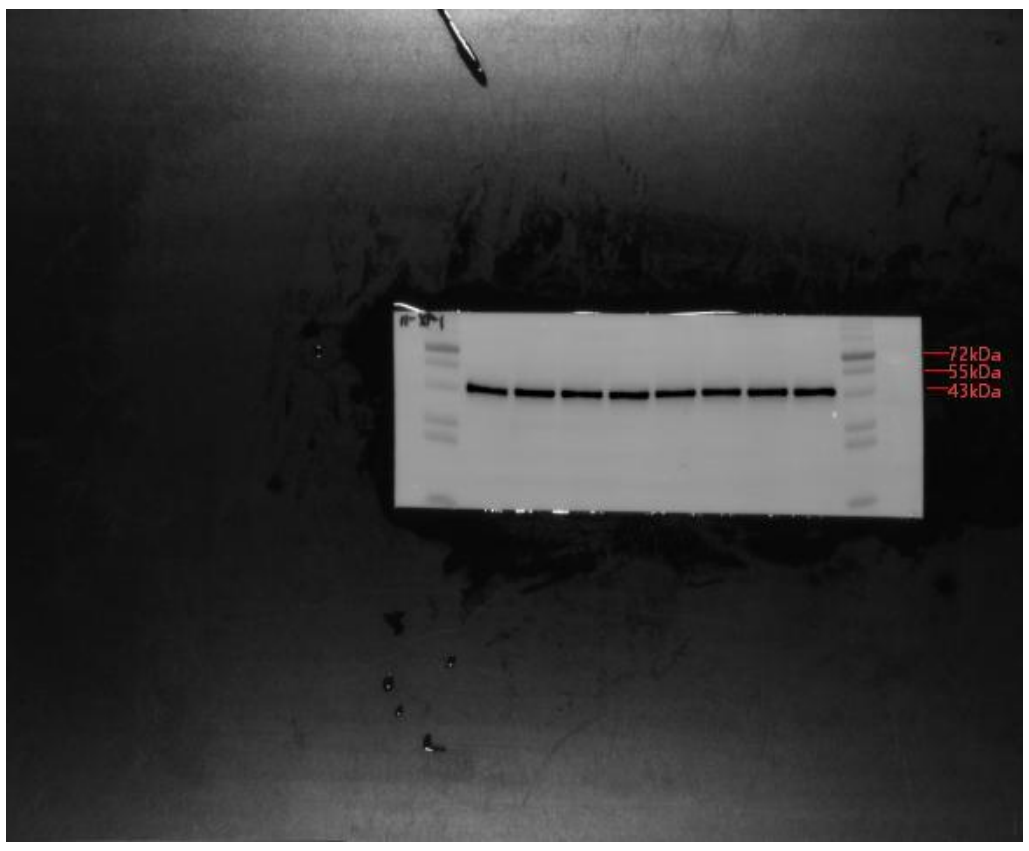

ENO1 ACTIN

FIG.5F

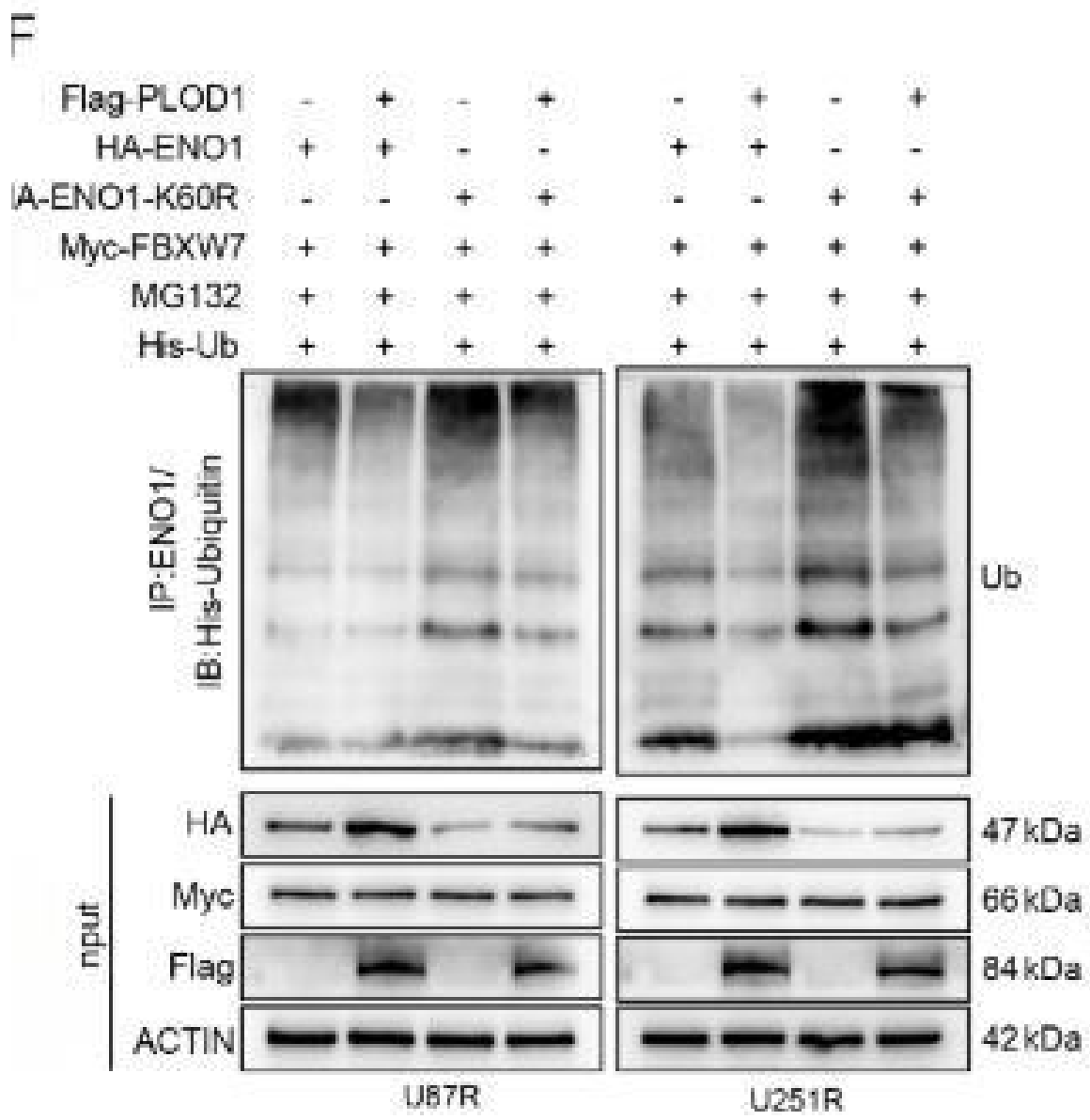

U87R

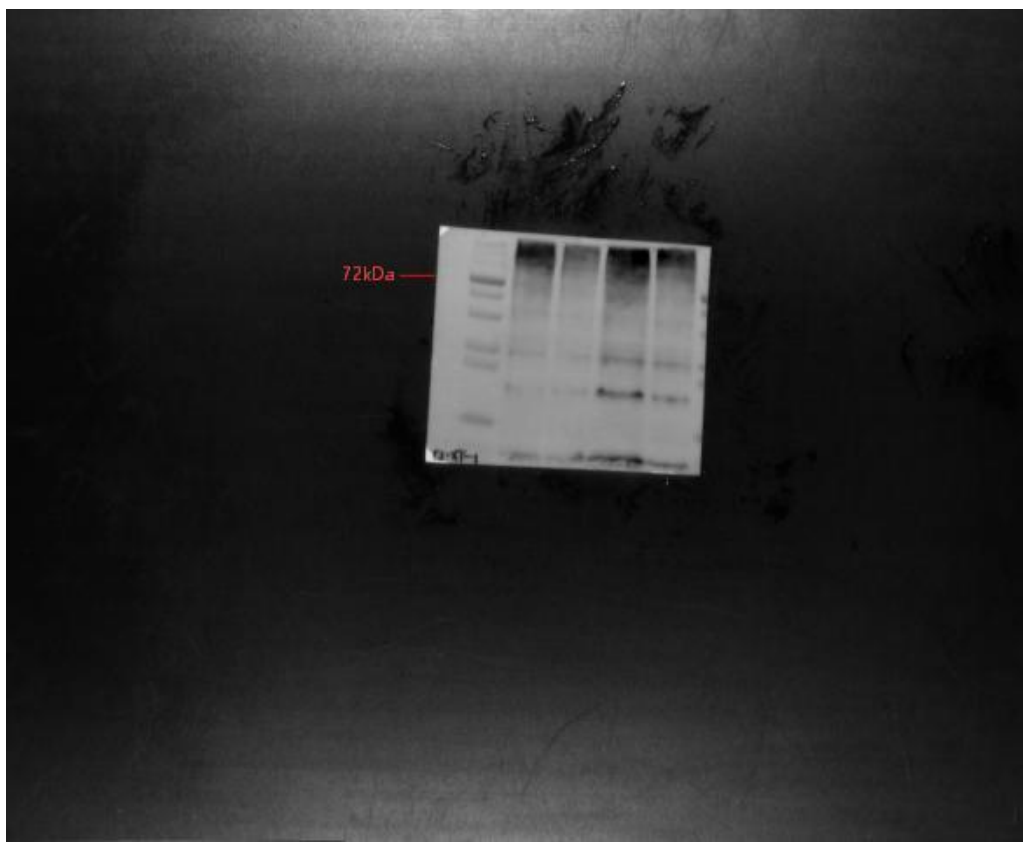

IP

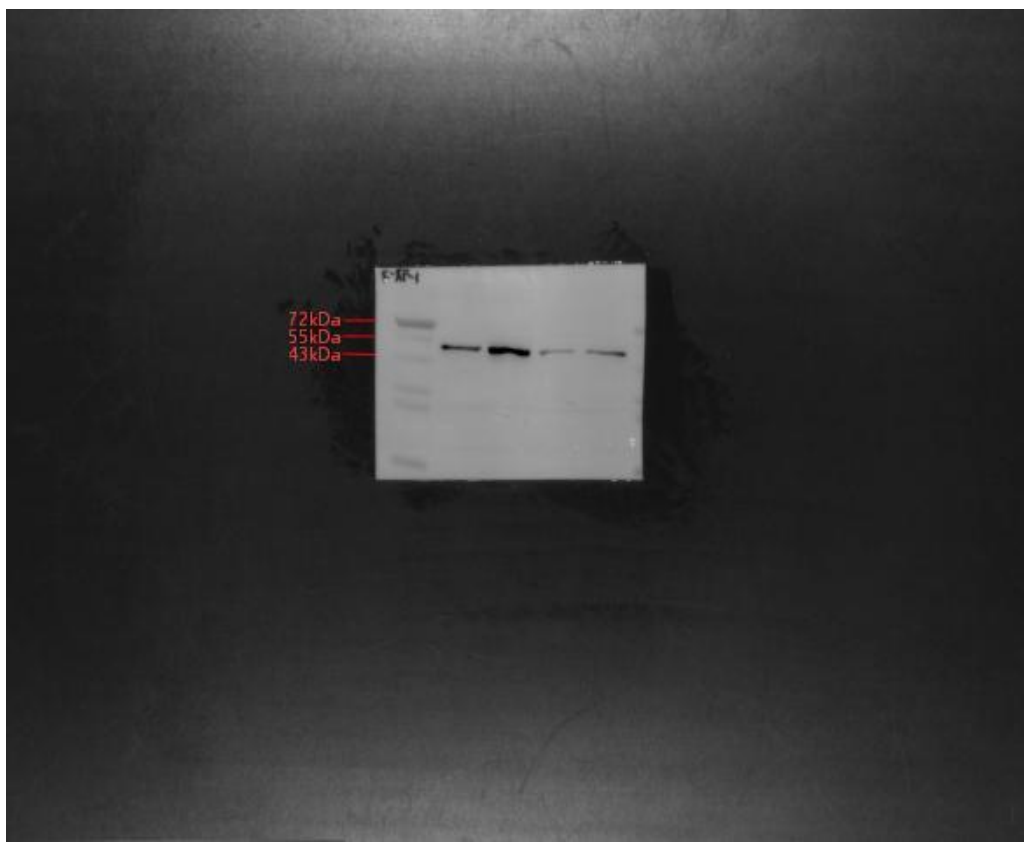

HA（ENO1）

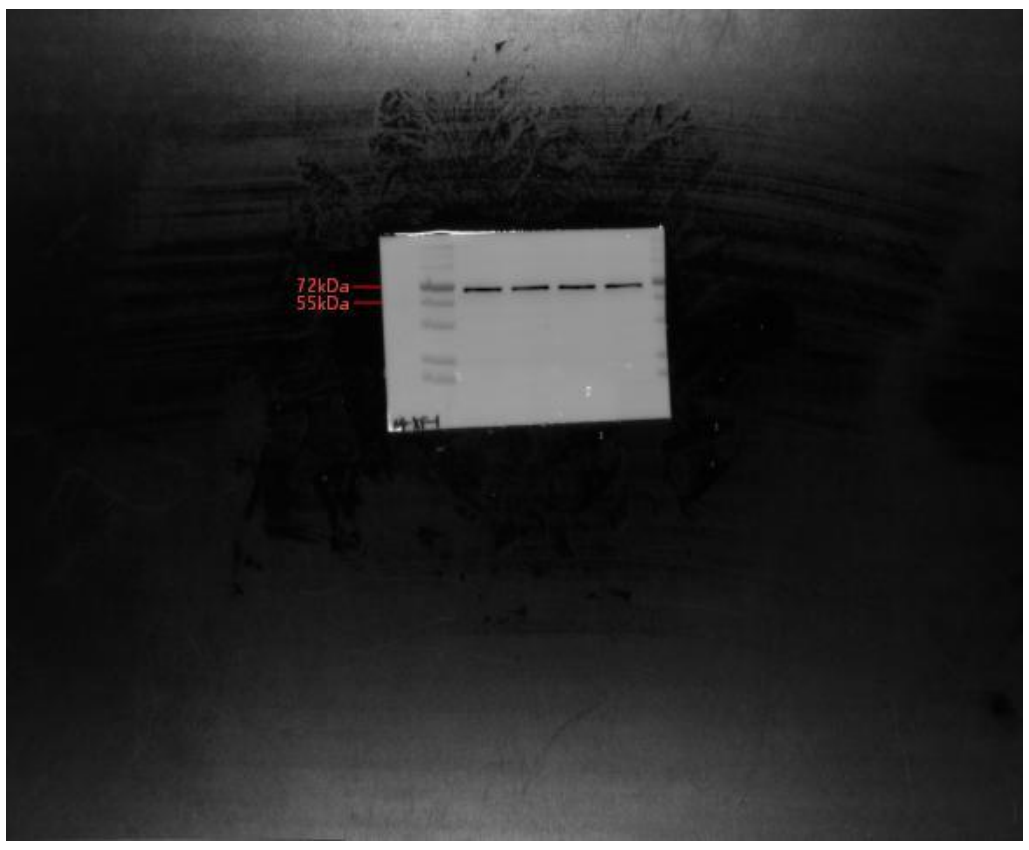

Myc（FBXW7）

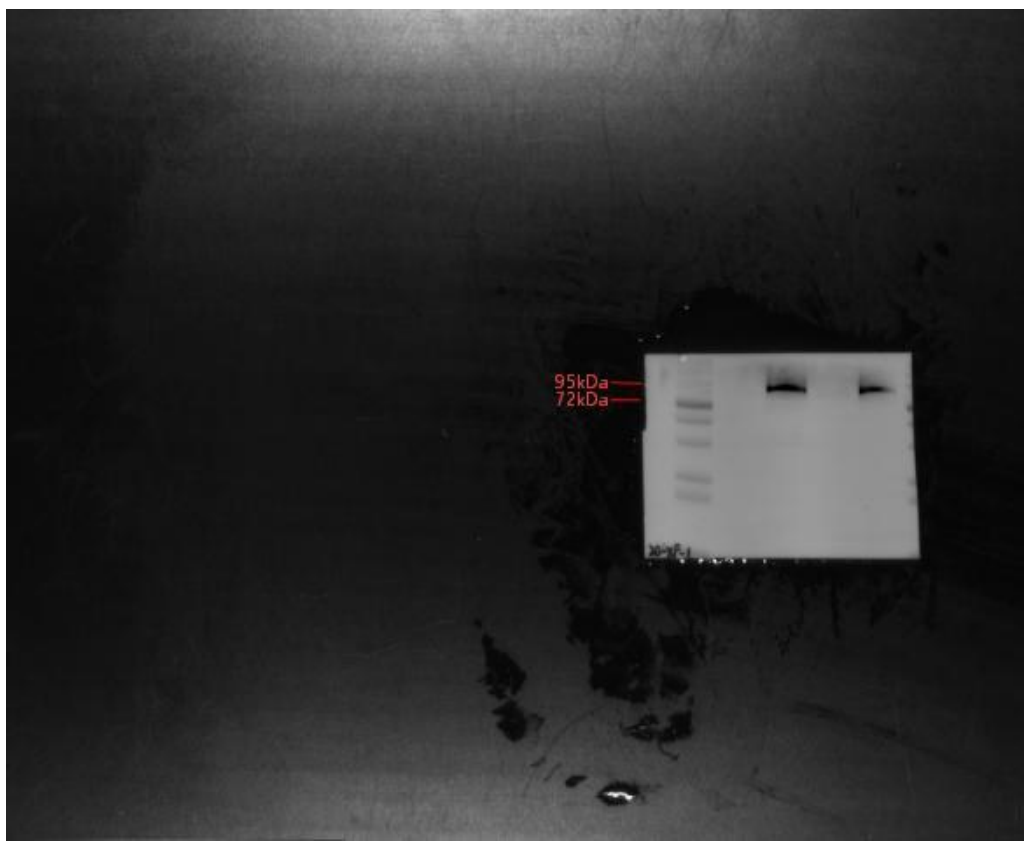

Flag（PLOD1）

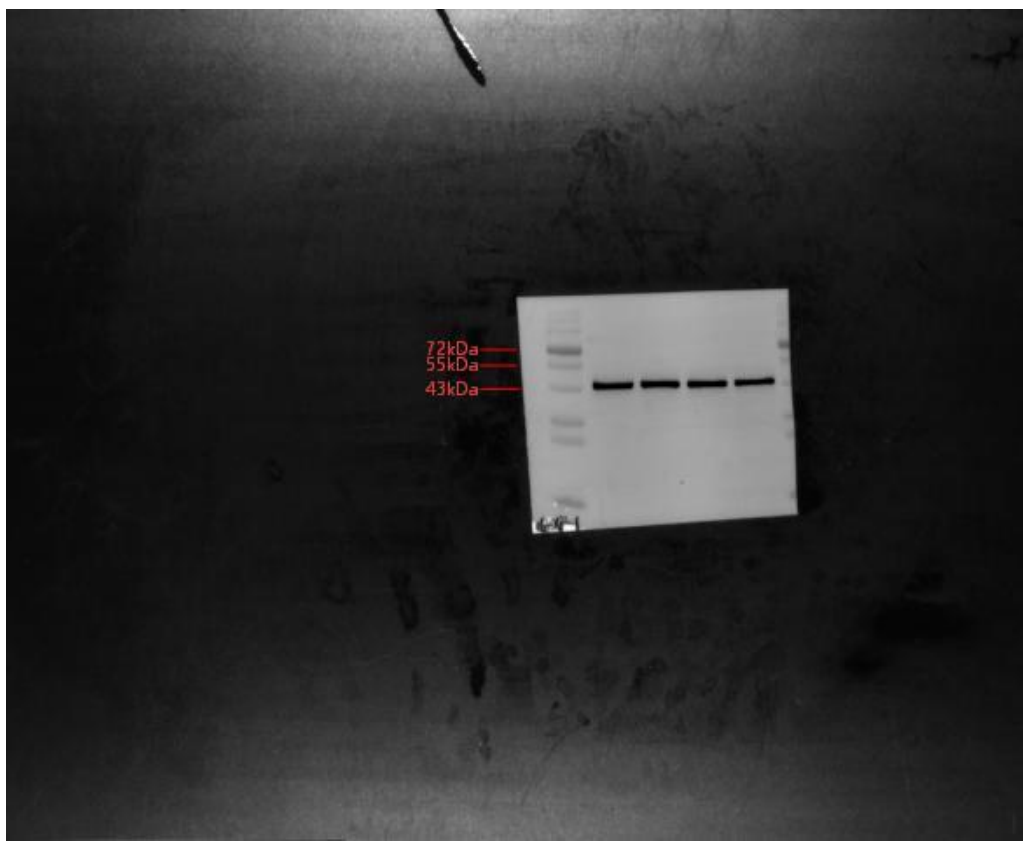

ACTIN

U251R

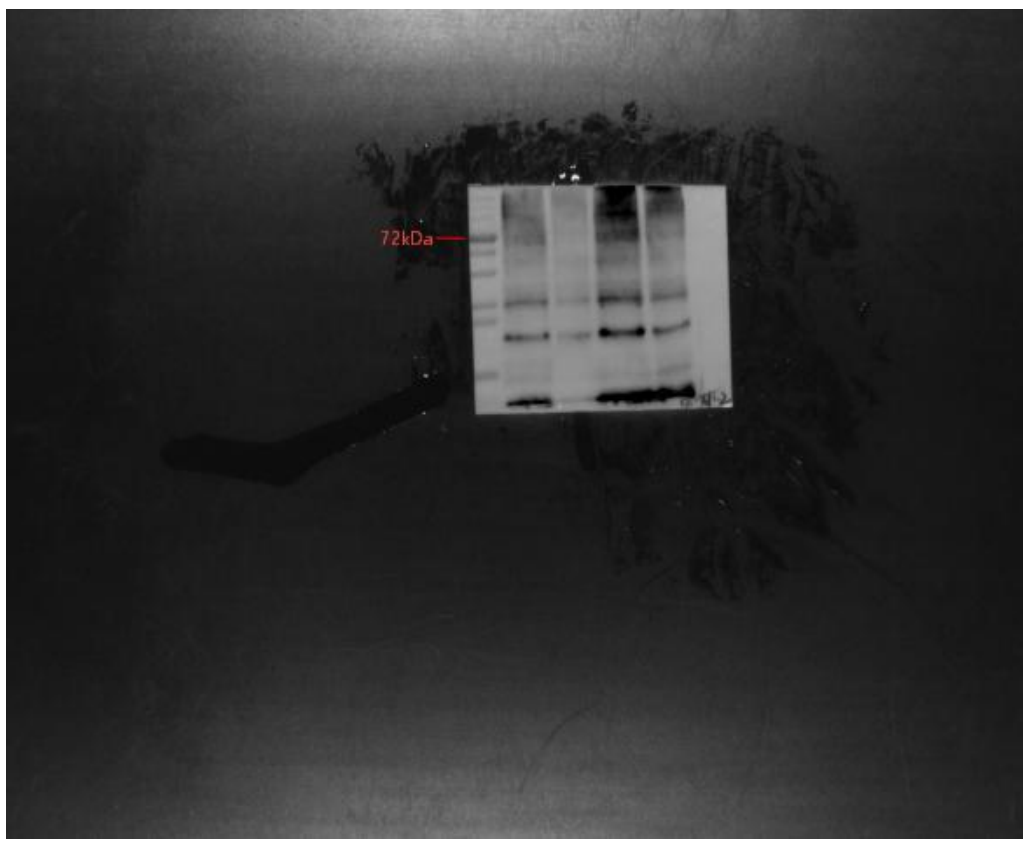

IP

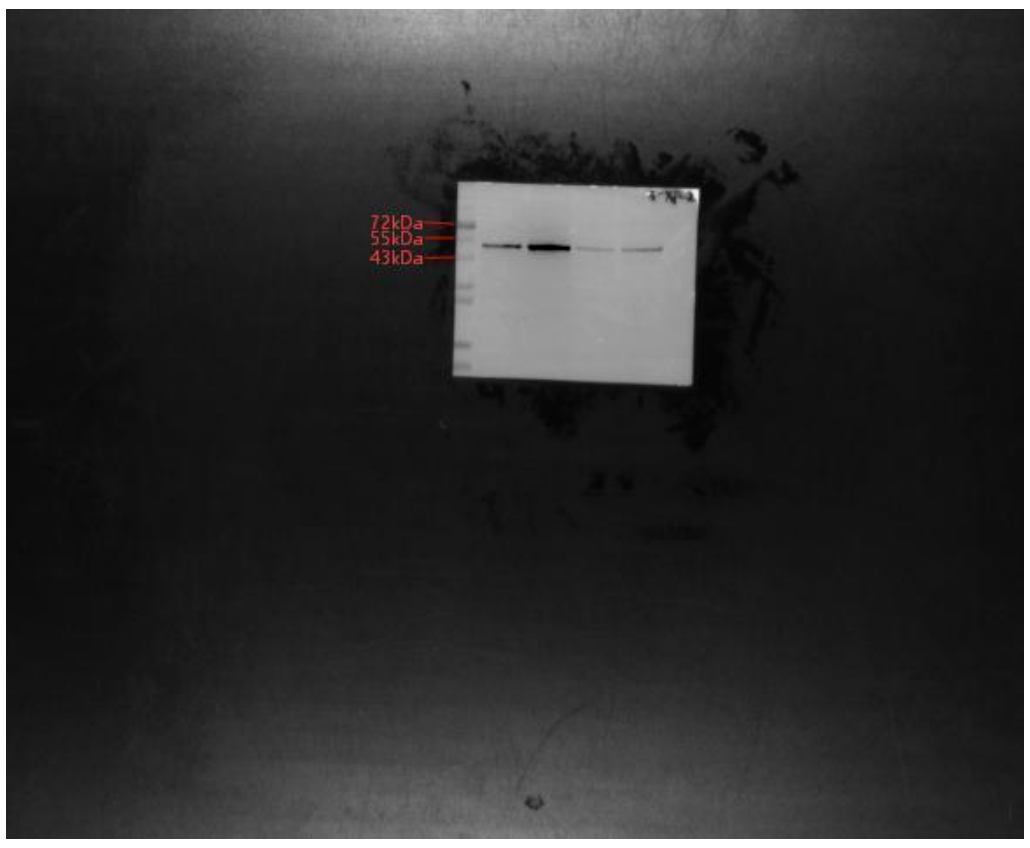

HA（ENO1）

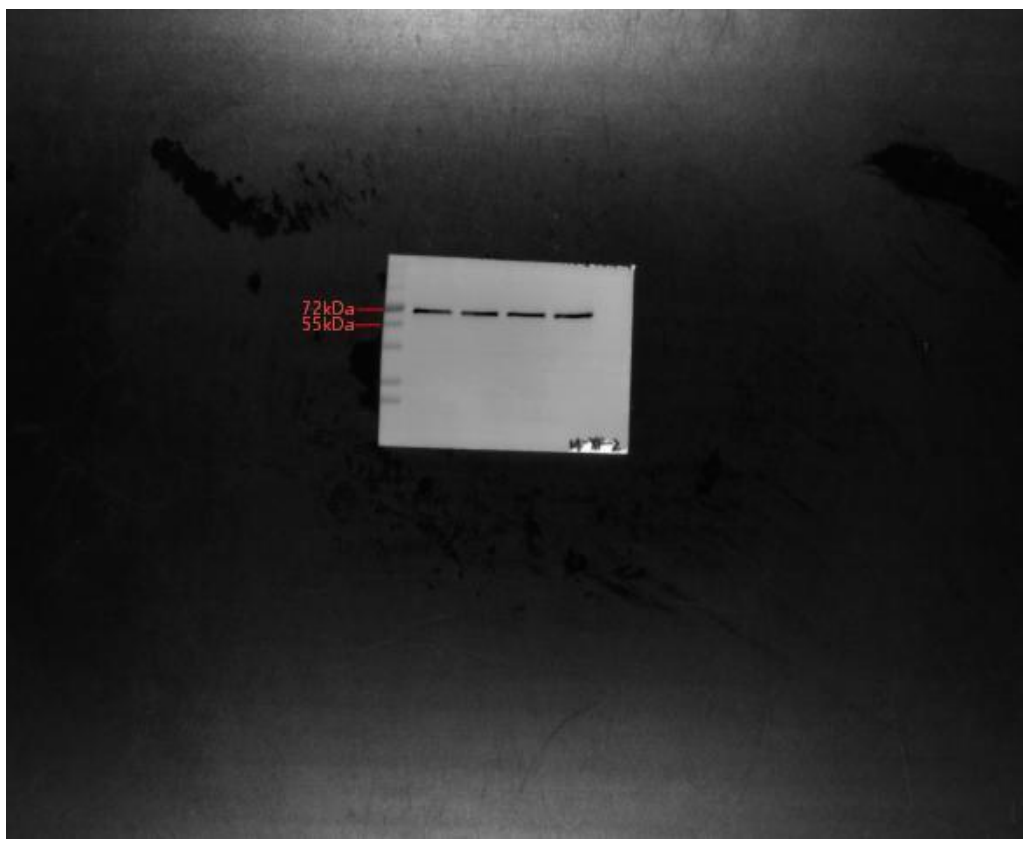

Myc（FBXW7）

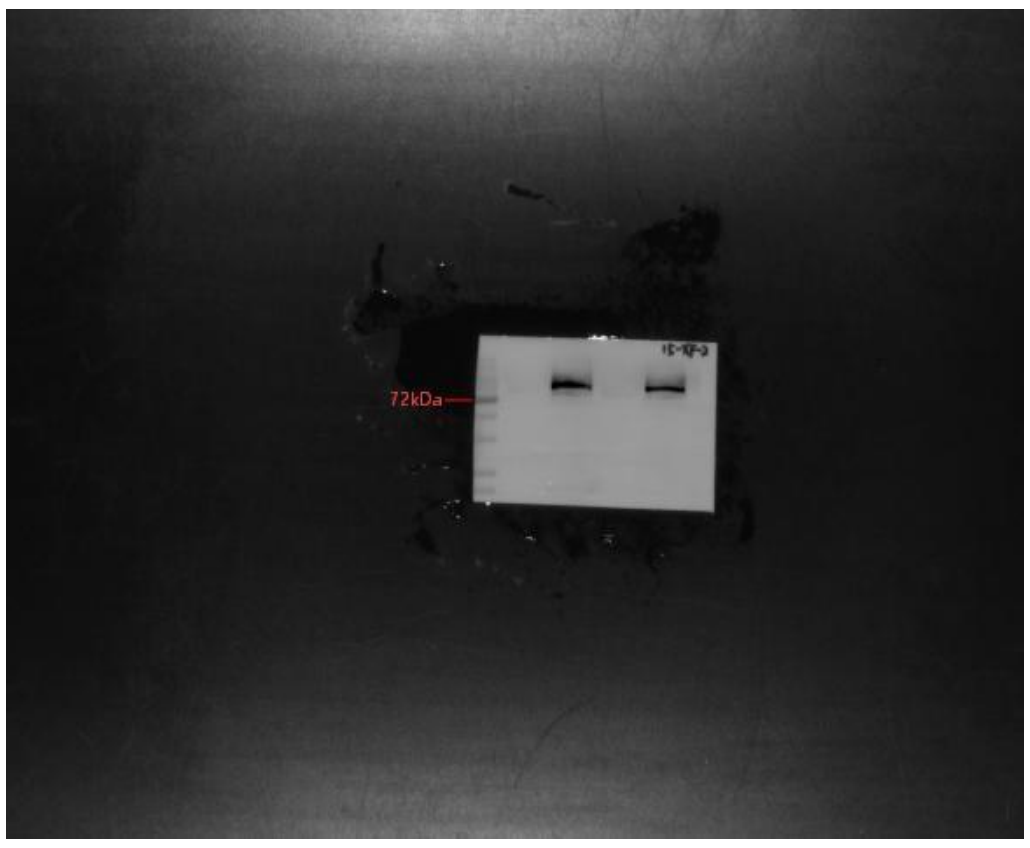

Flag（PLOD1）

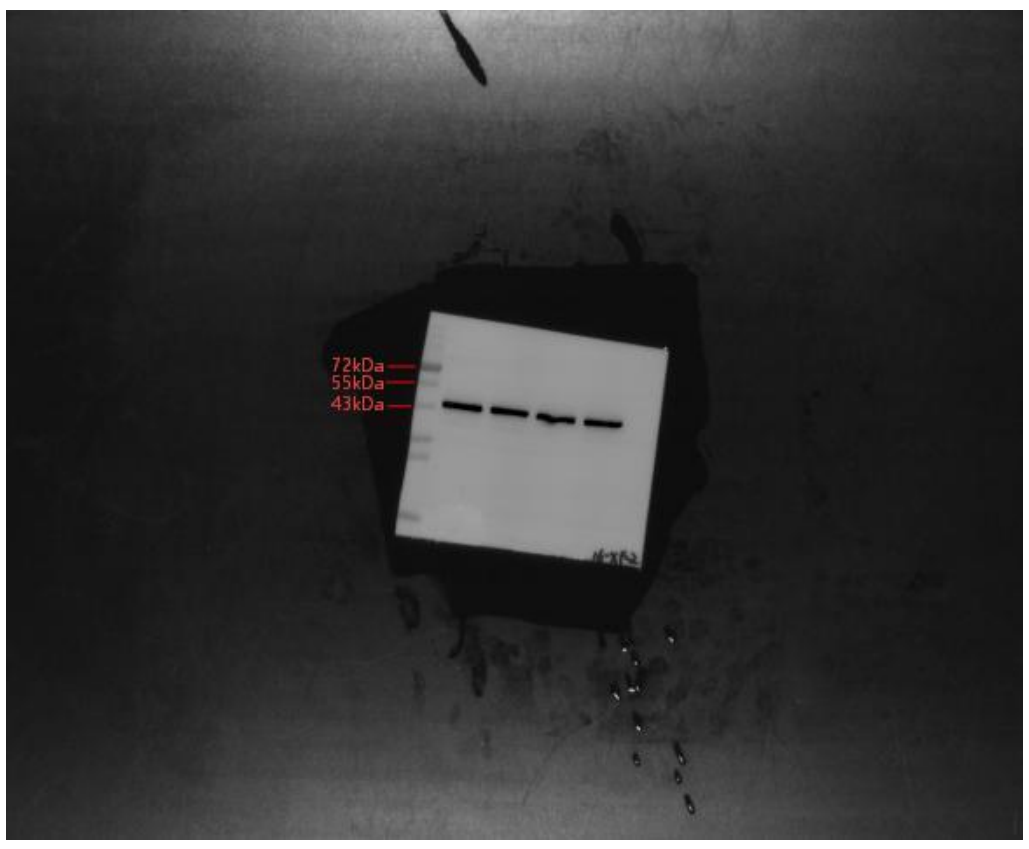

ACTIN

FIG.5E

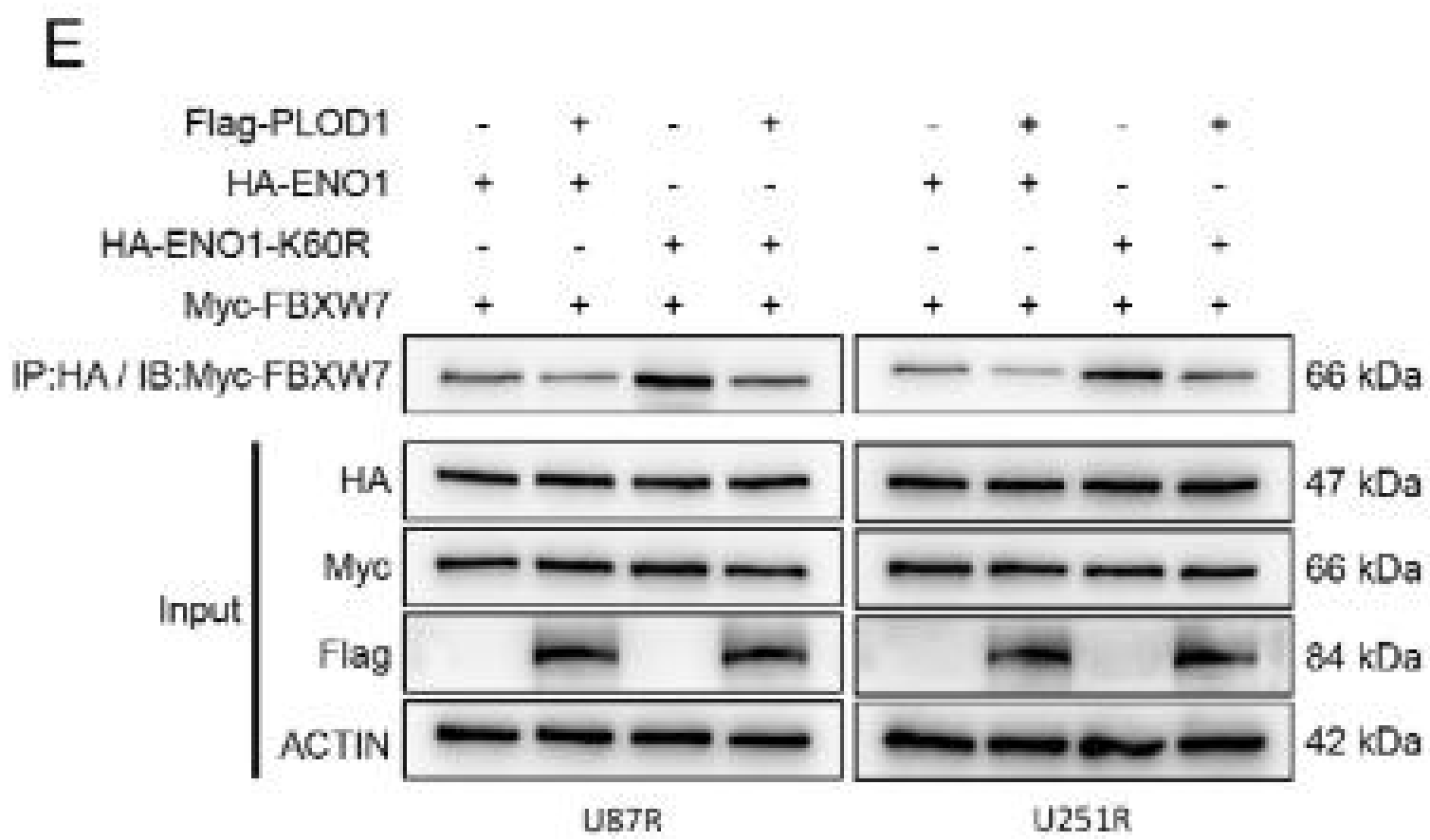

U87R

IP

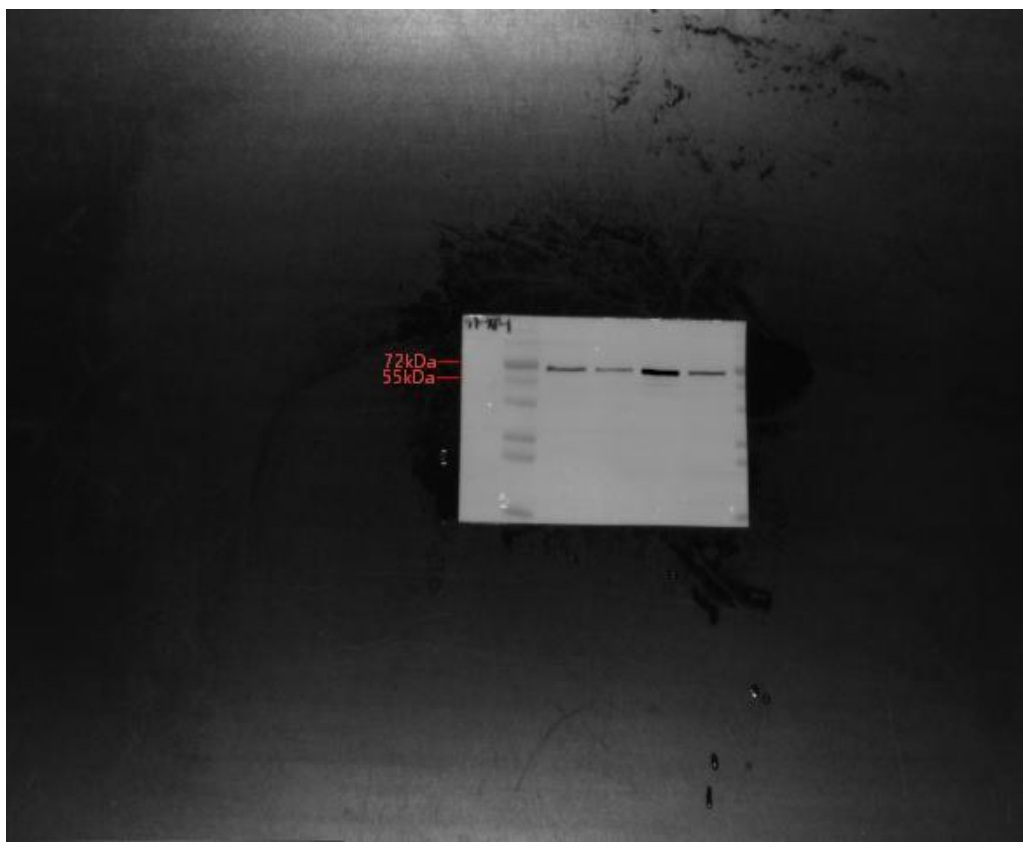

FBXW7

Input

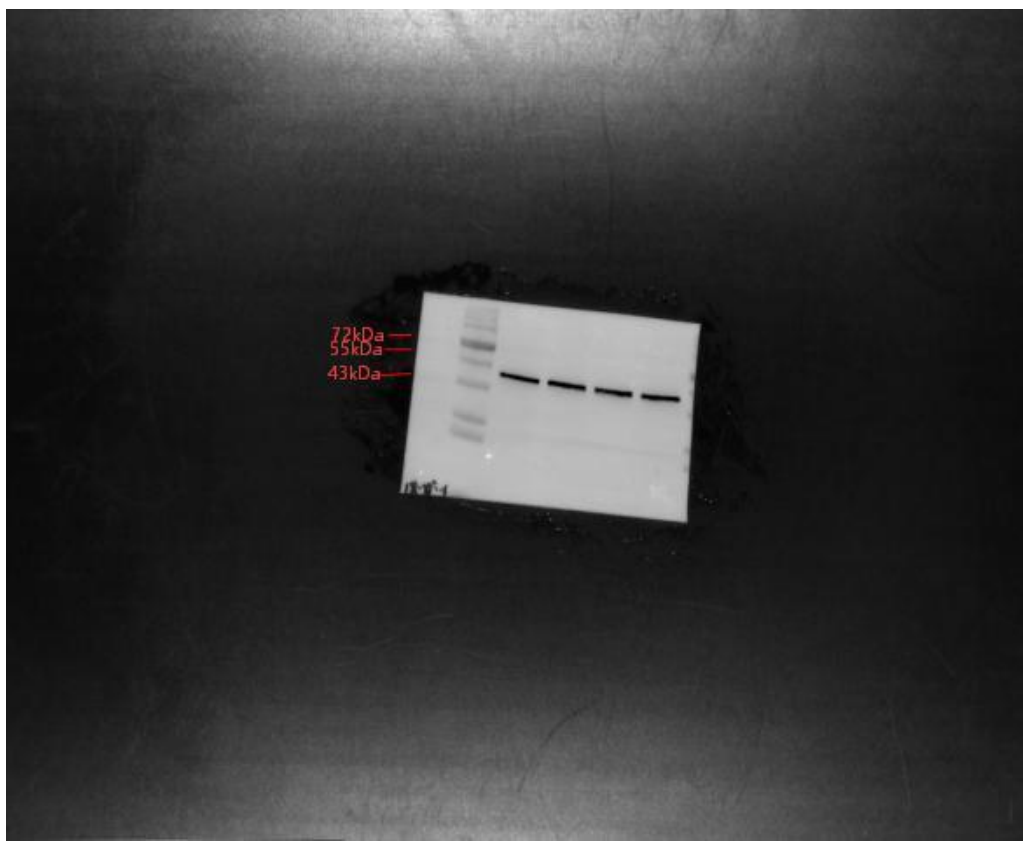

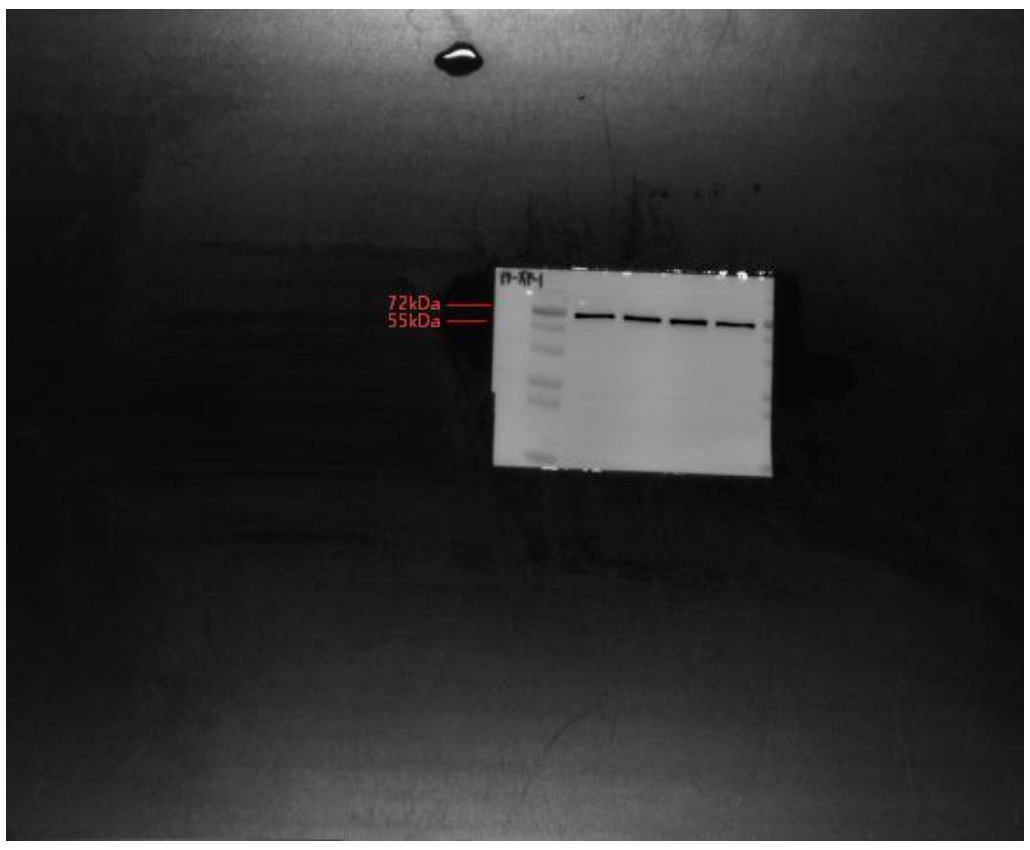

ENO1

FBXW7

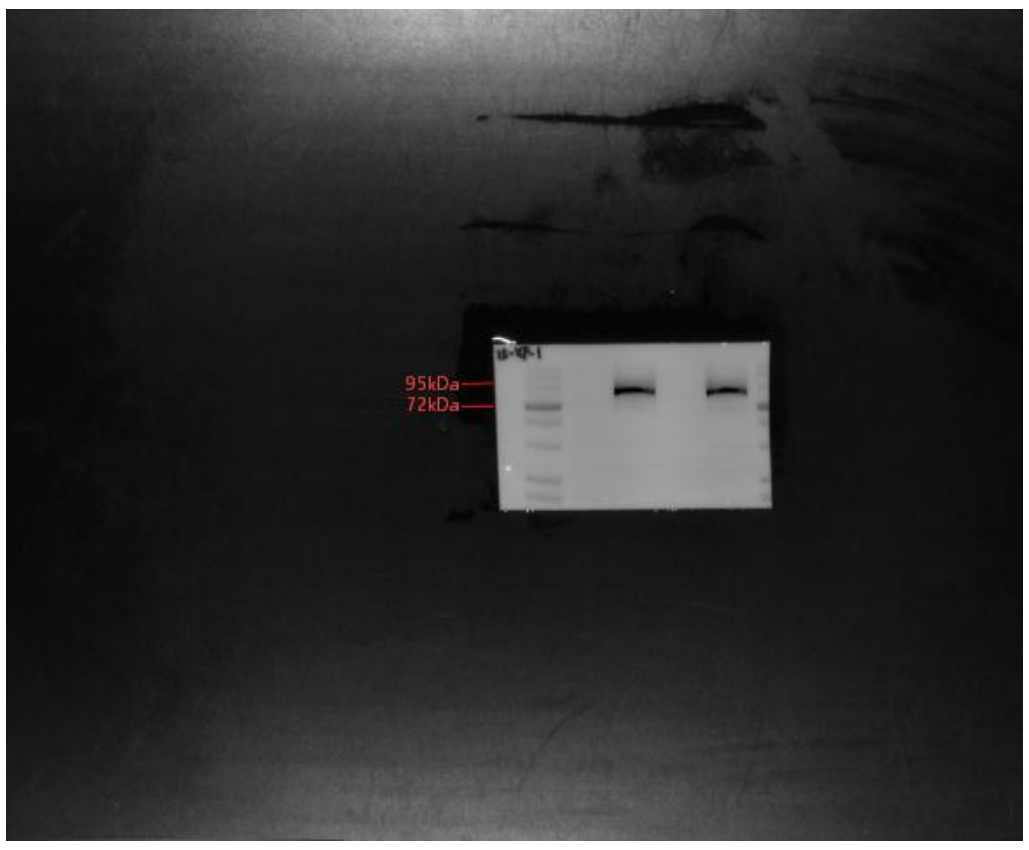

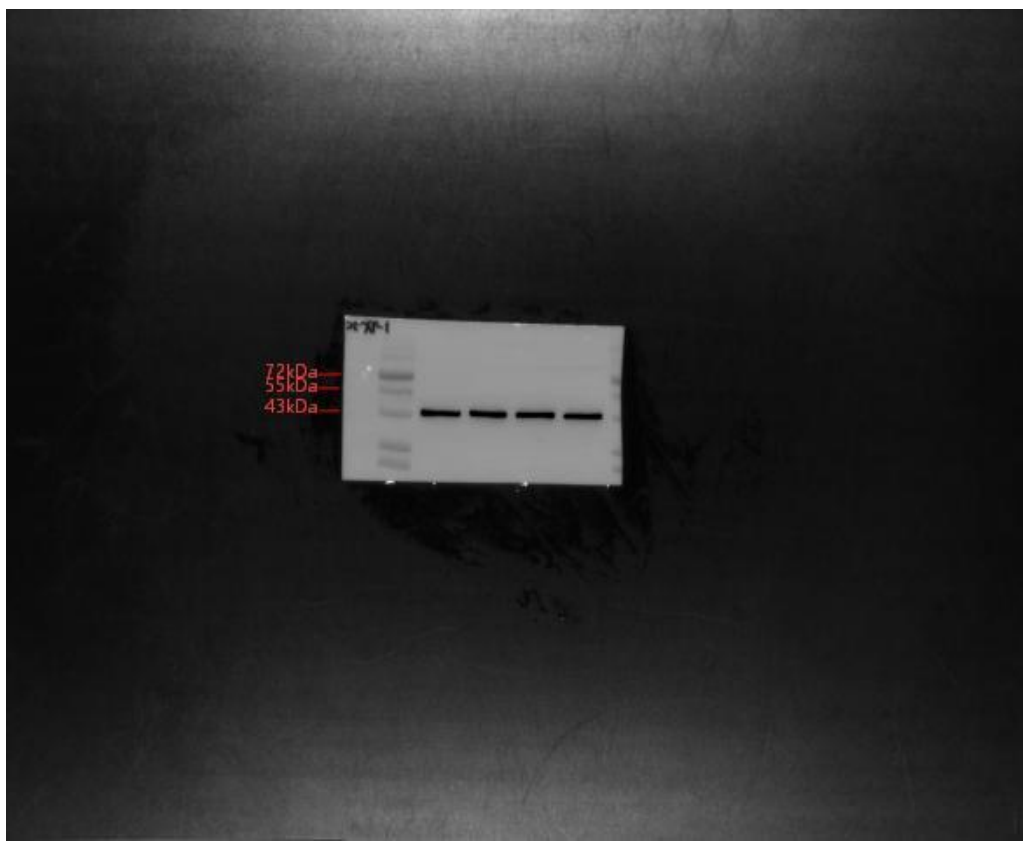

PLOD1 ACTIN

U251R

IP

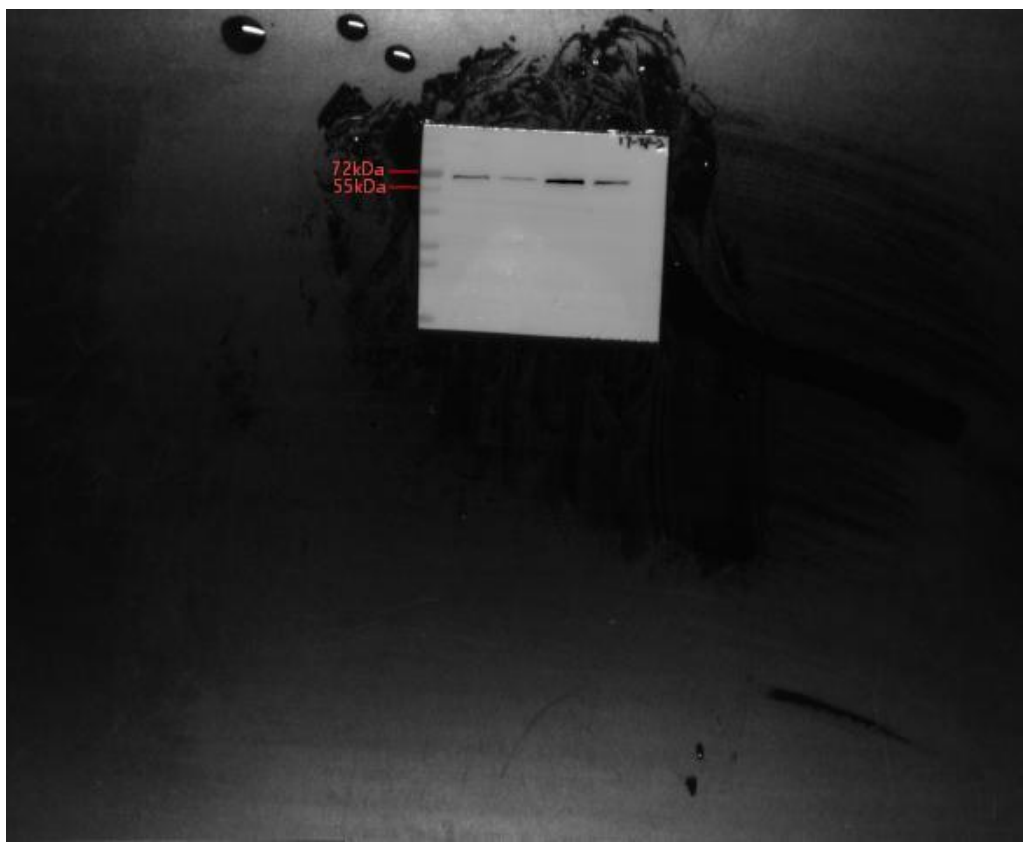

FBXW7

Input

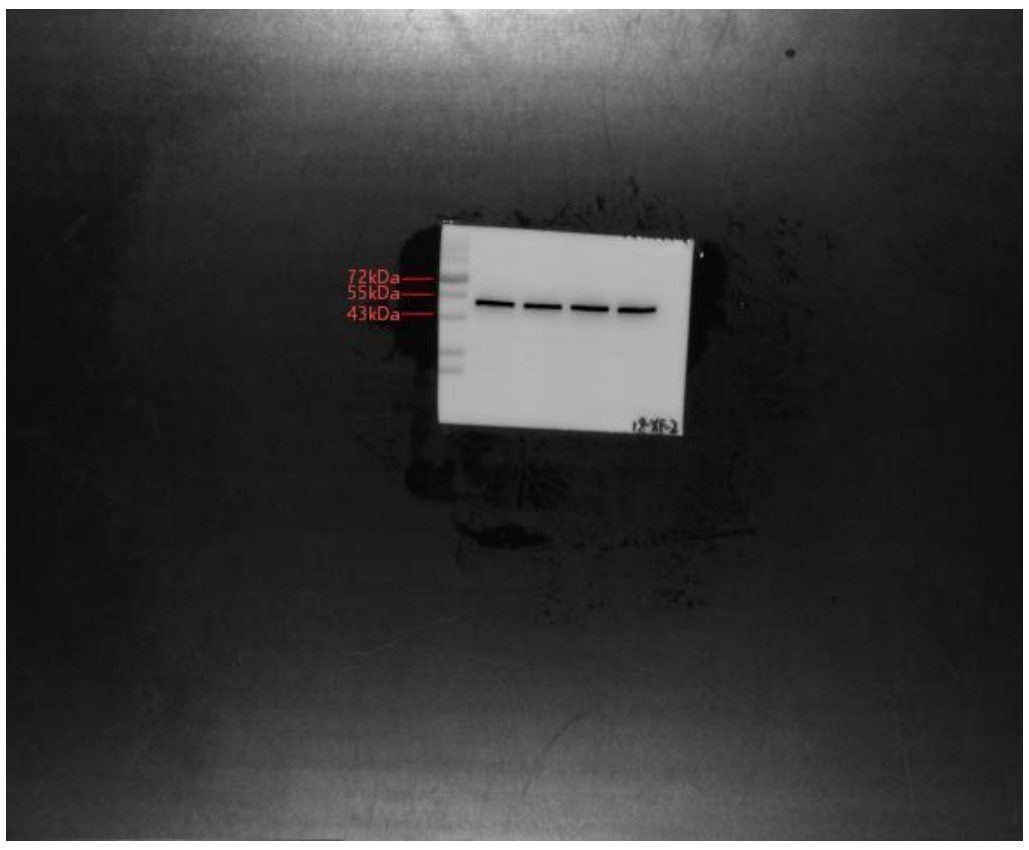

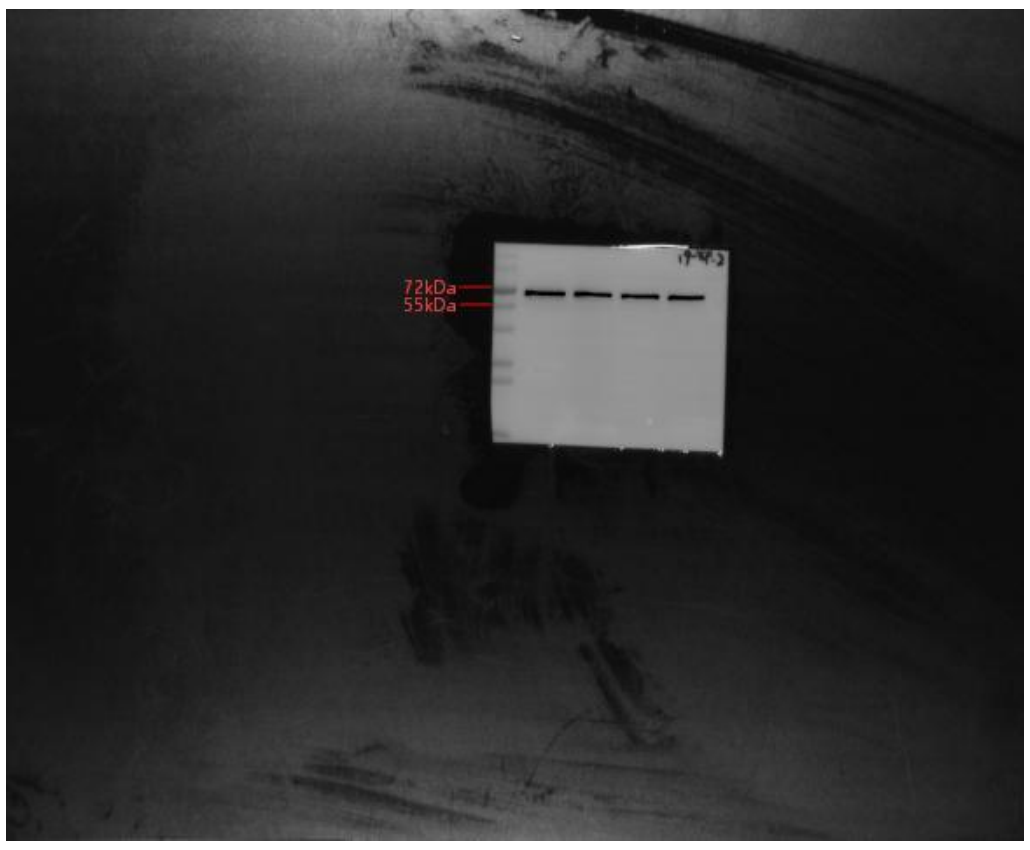

ENO1 FBXW7

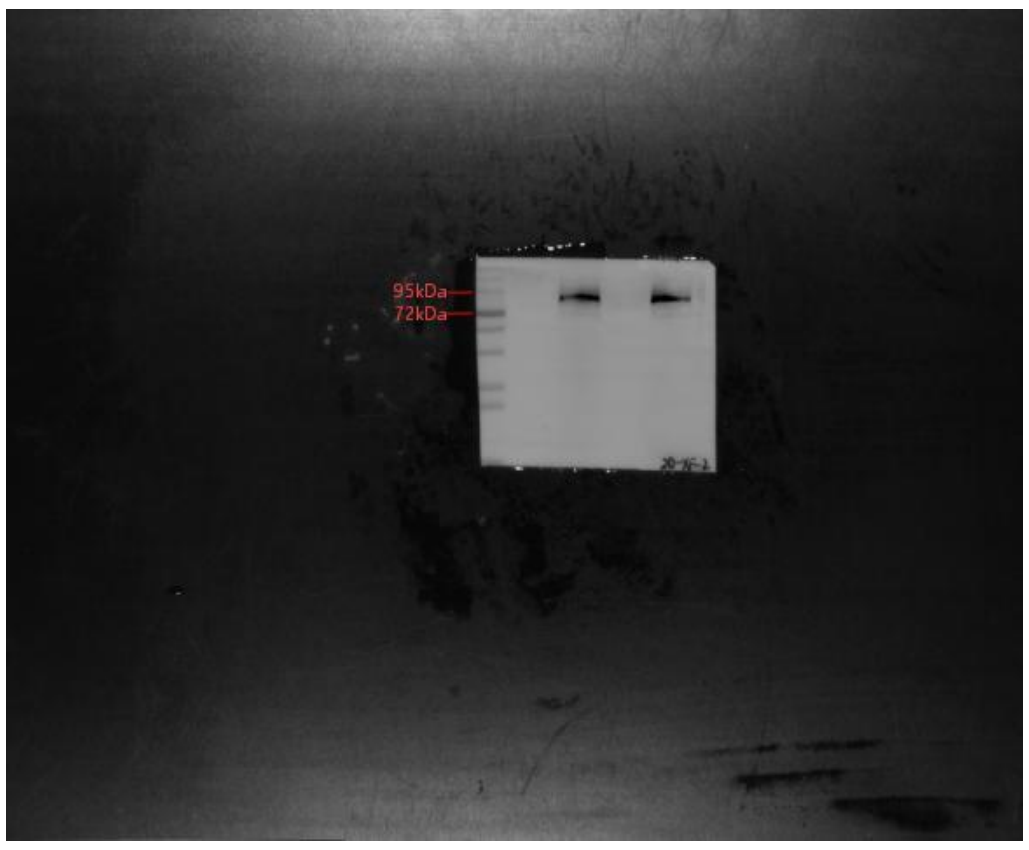

PLOD1

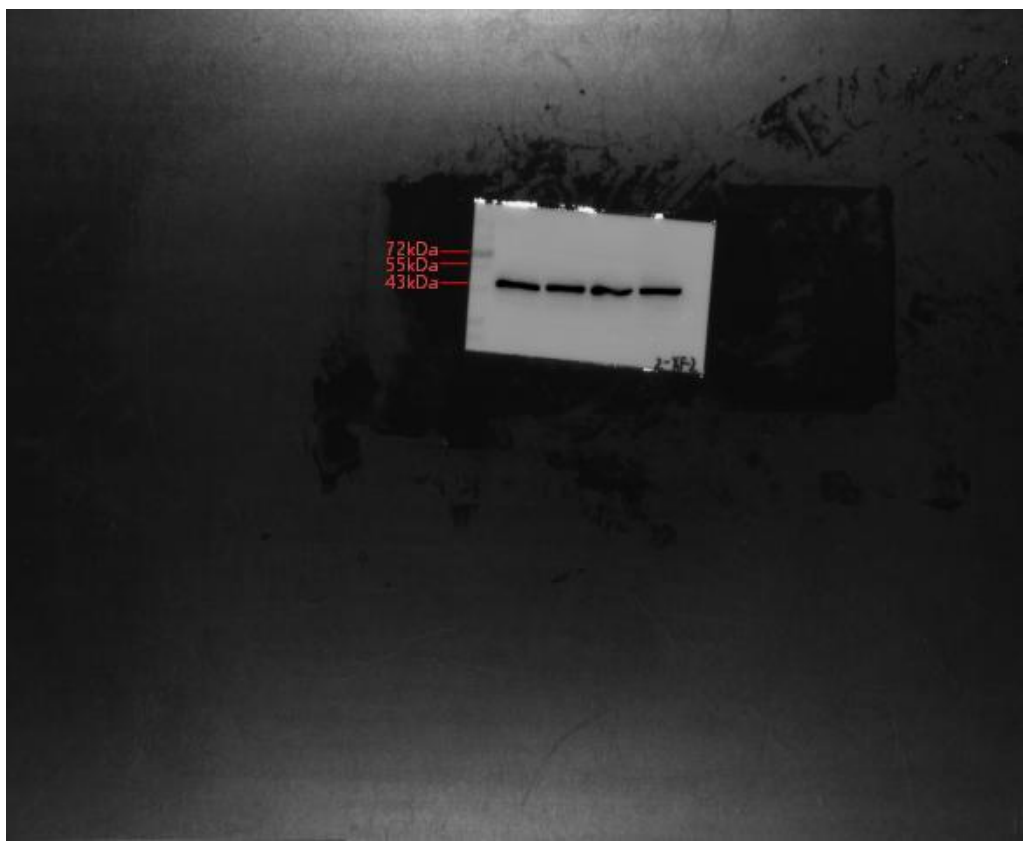

ACTIN

FIG.6B

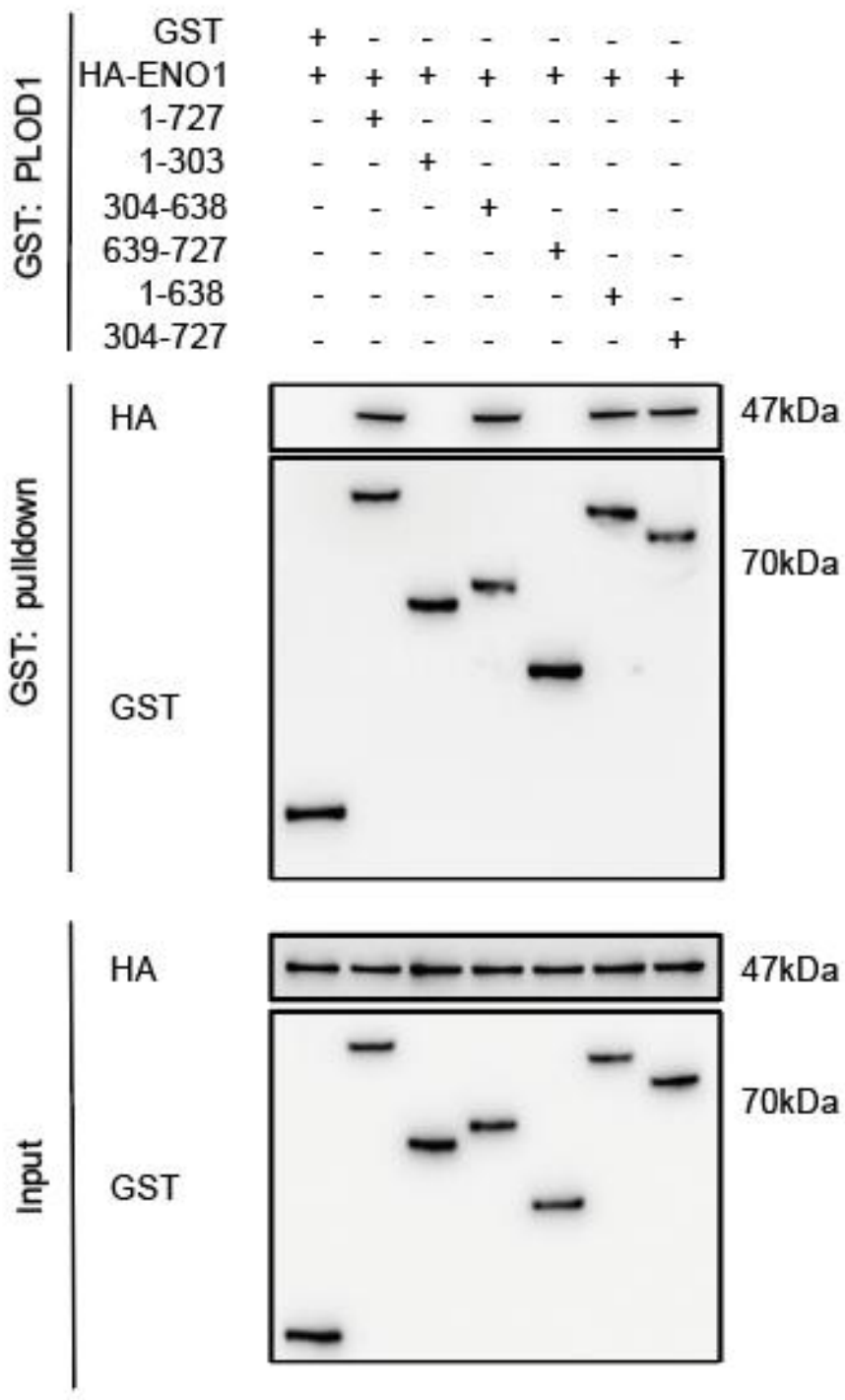

GST pulldown

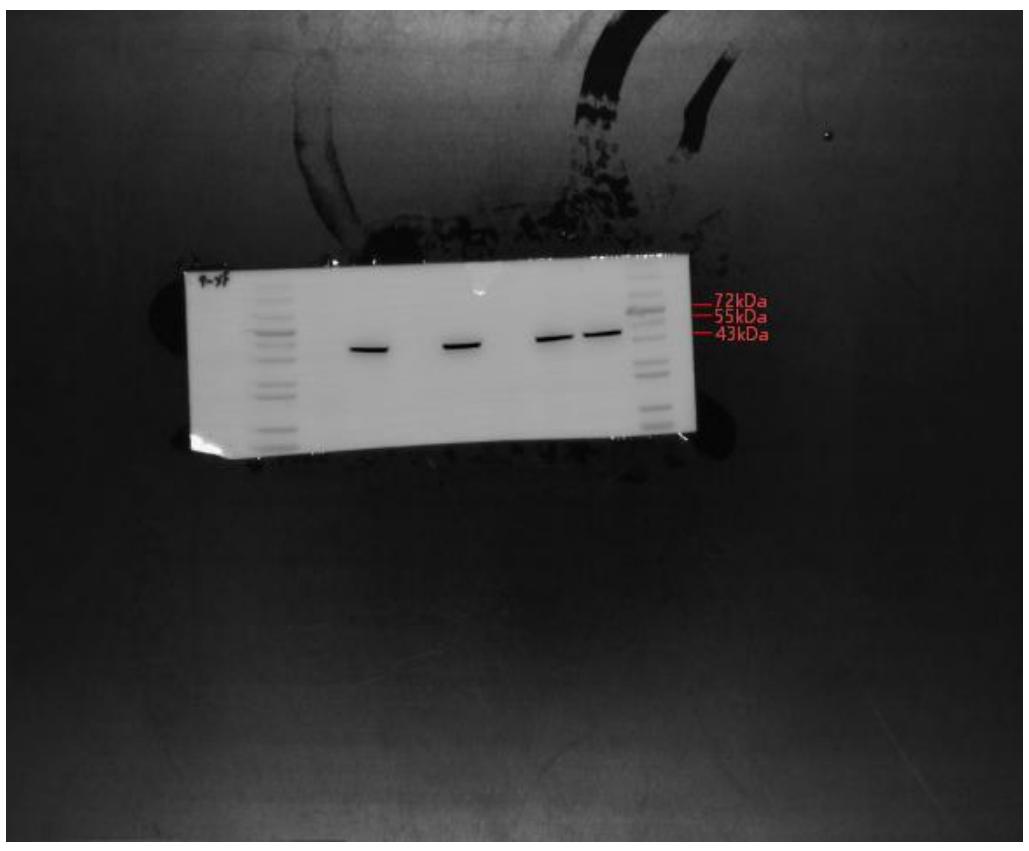

HA

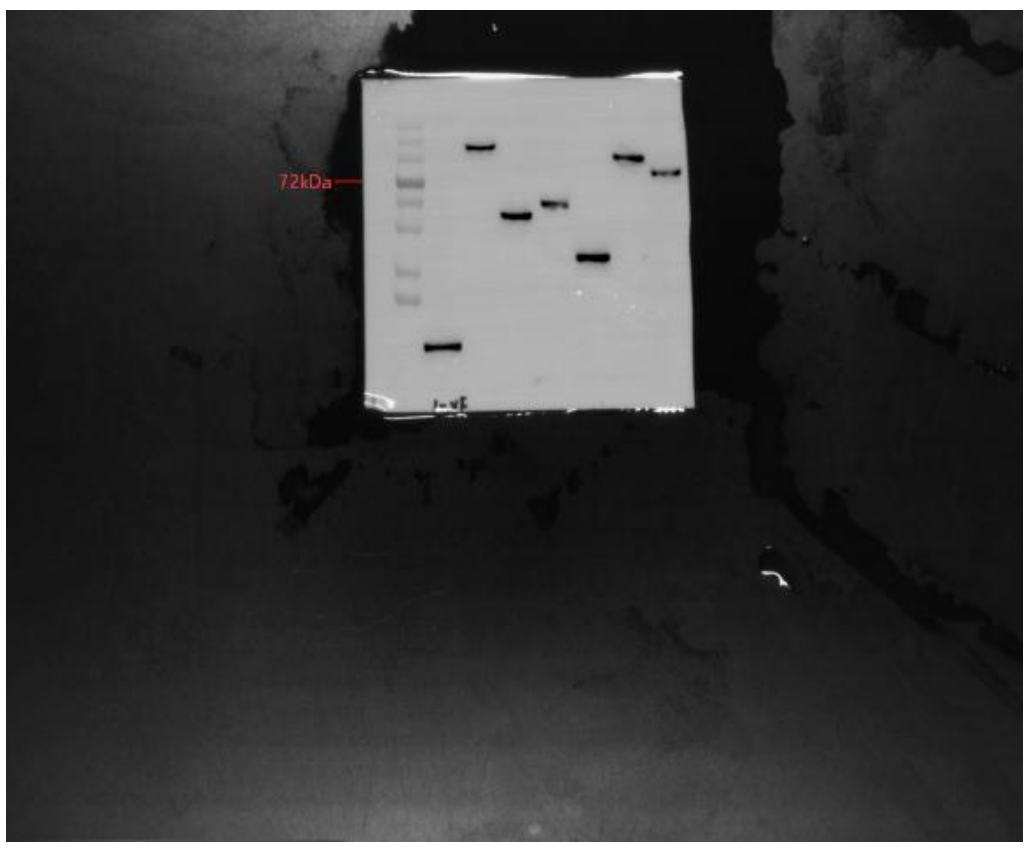

GST

Input

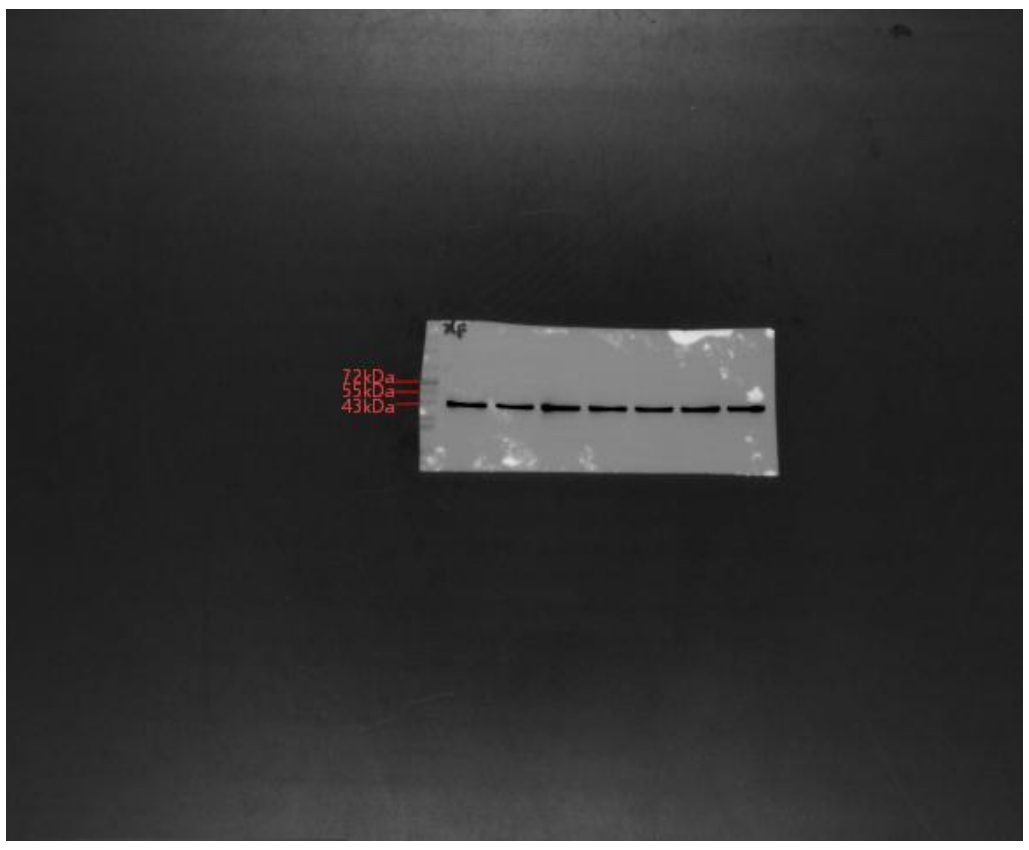

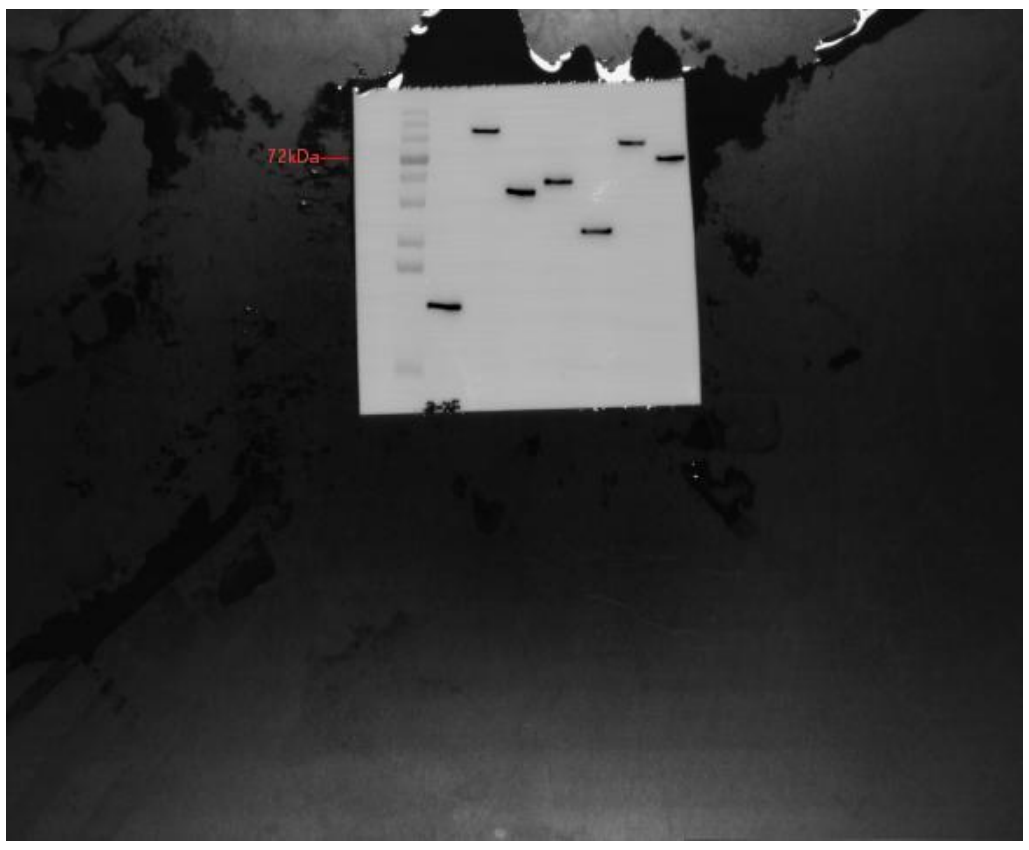

HA GST

FIG.6G

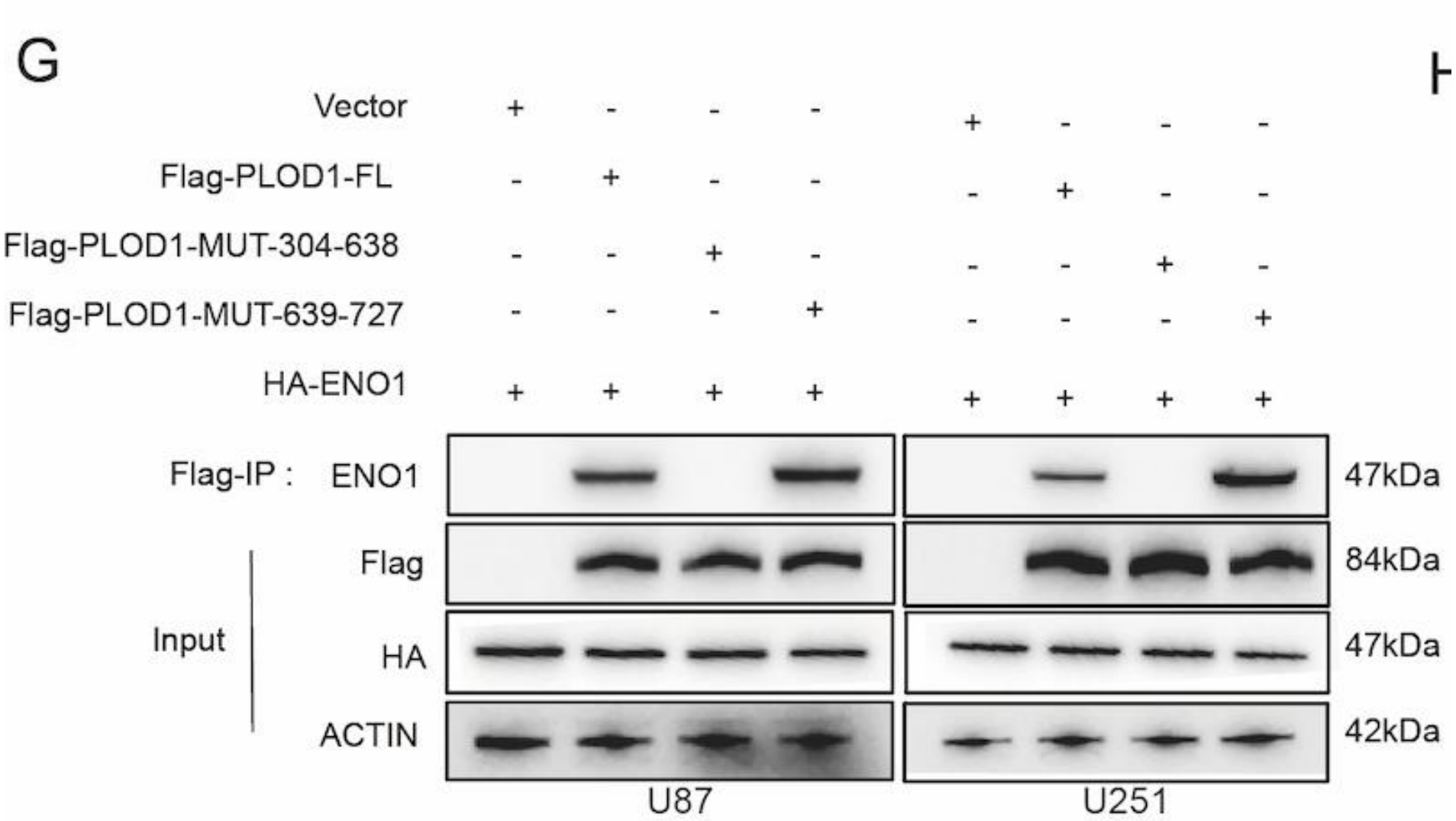

U87

IP

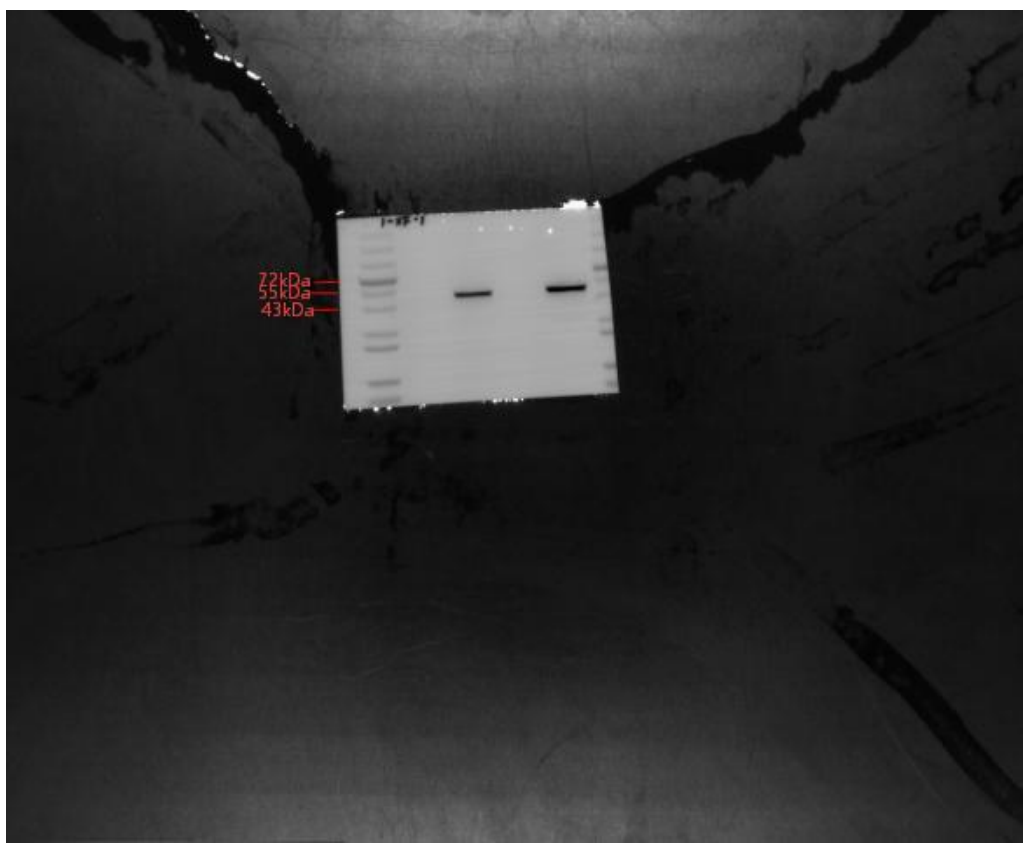

INPUT

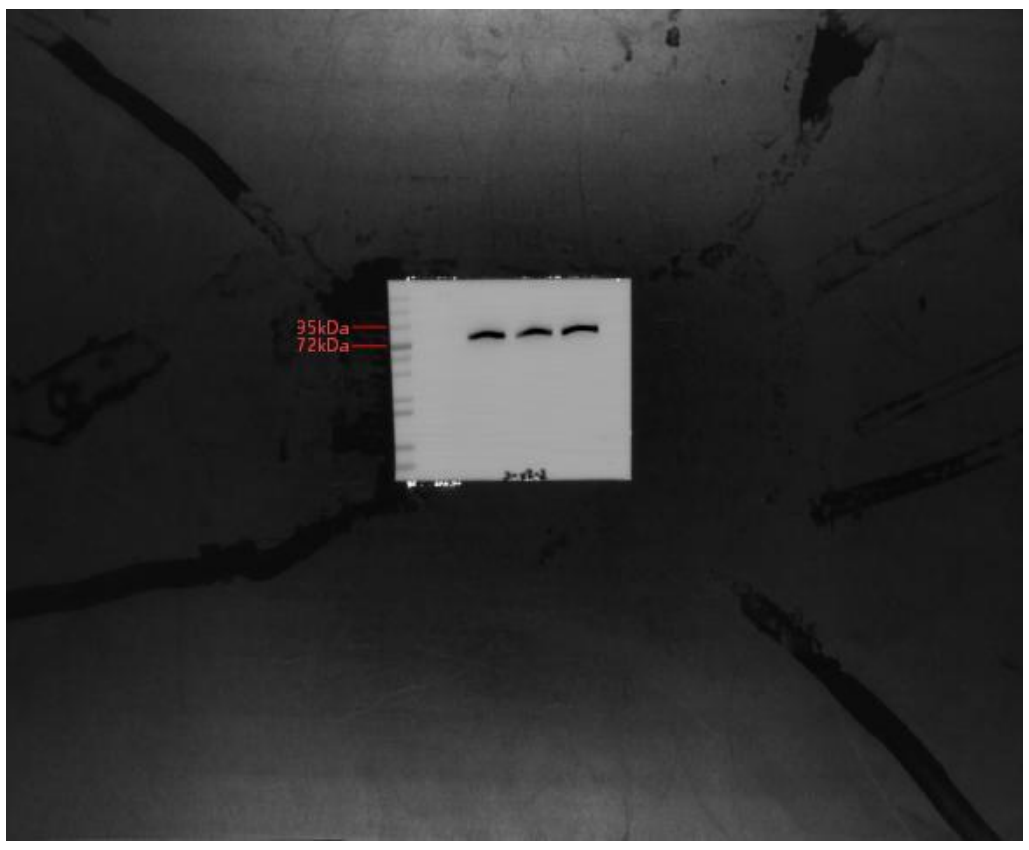

Flag

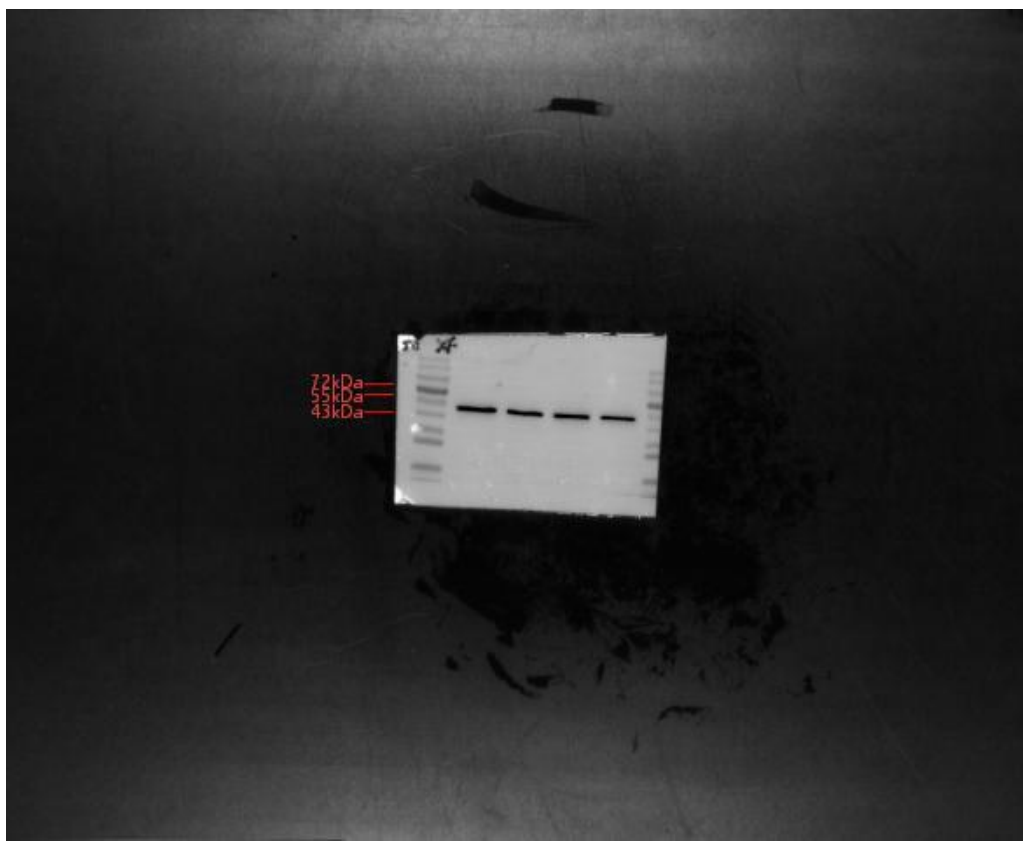

HA

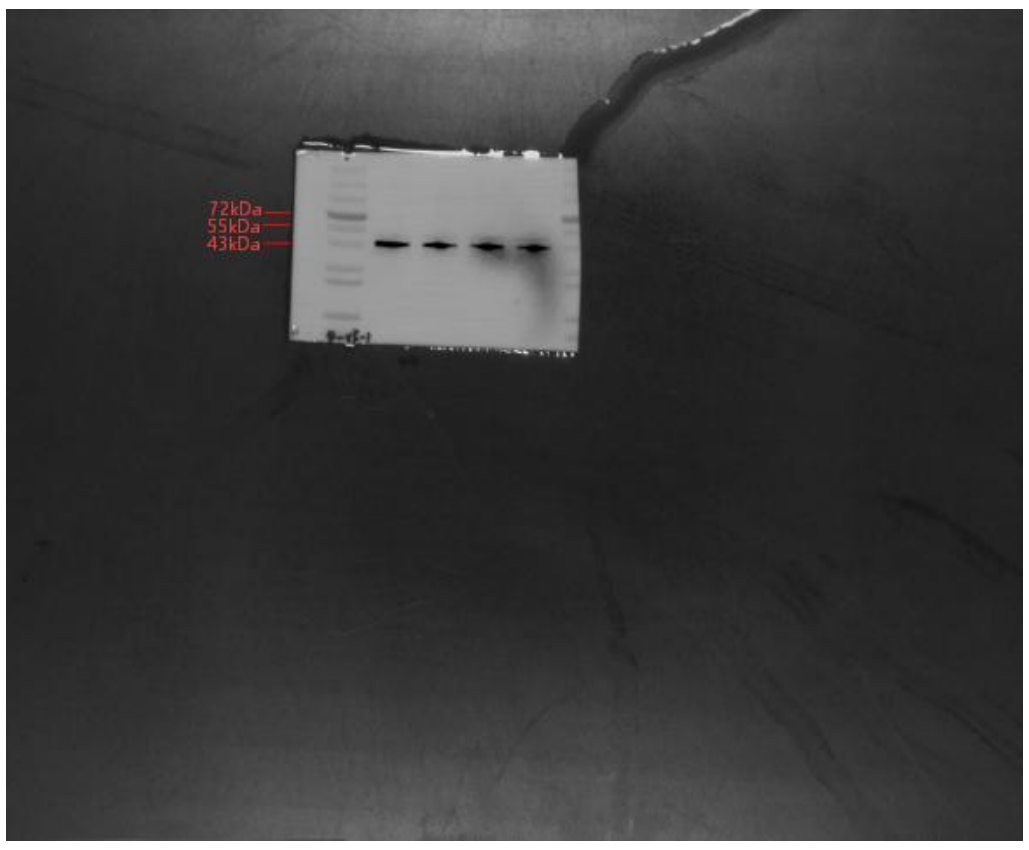

ACTIN

U251

IP

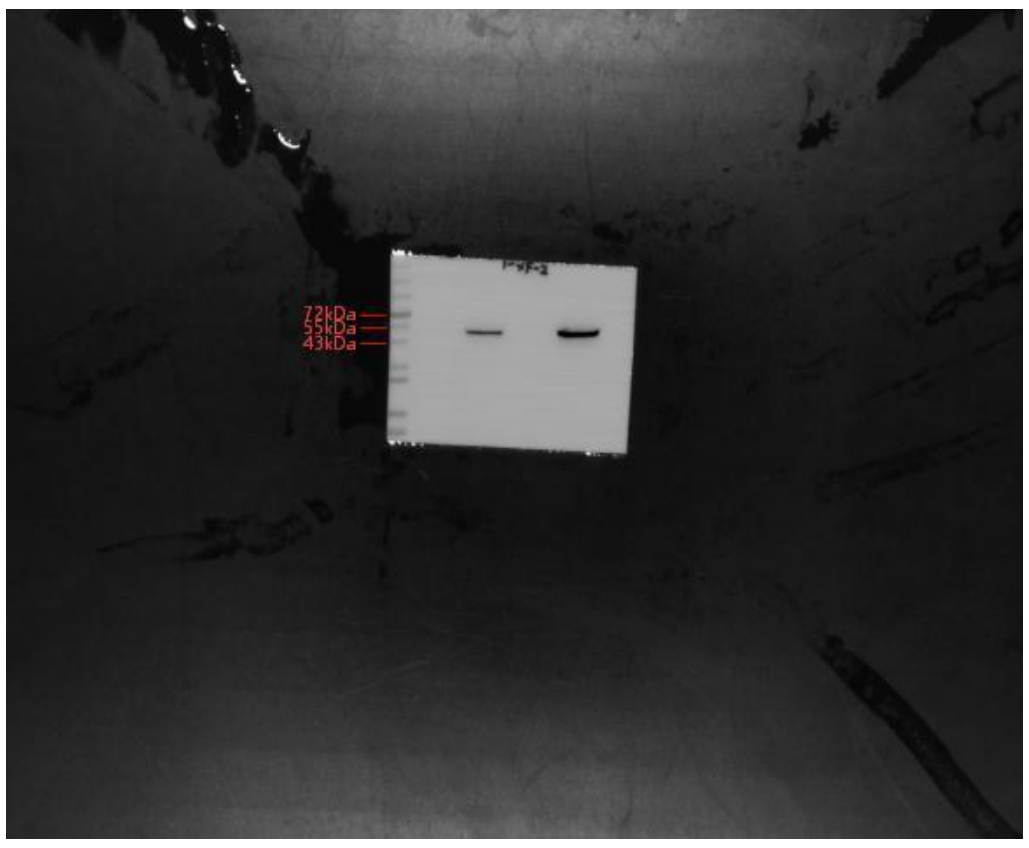

INPUT

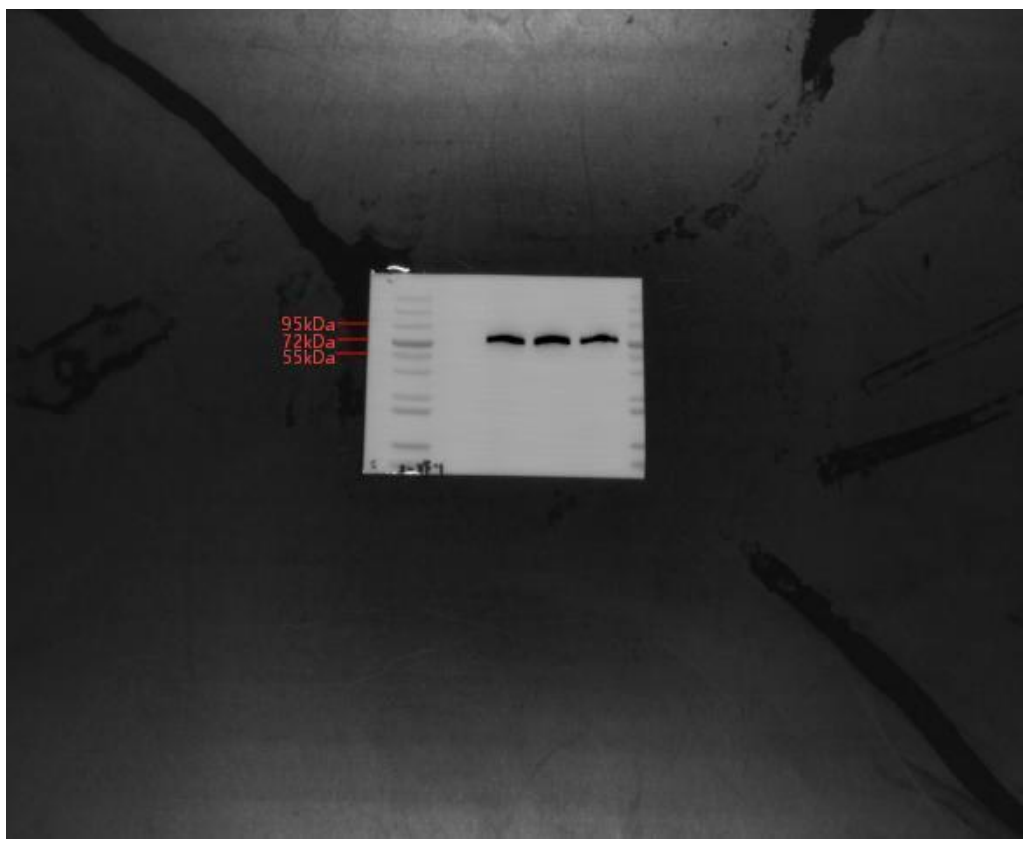

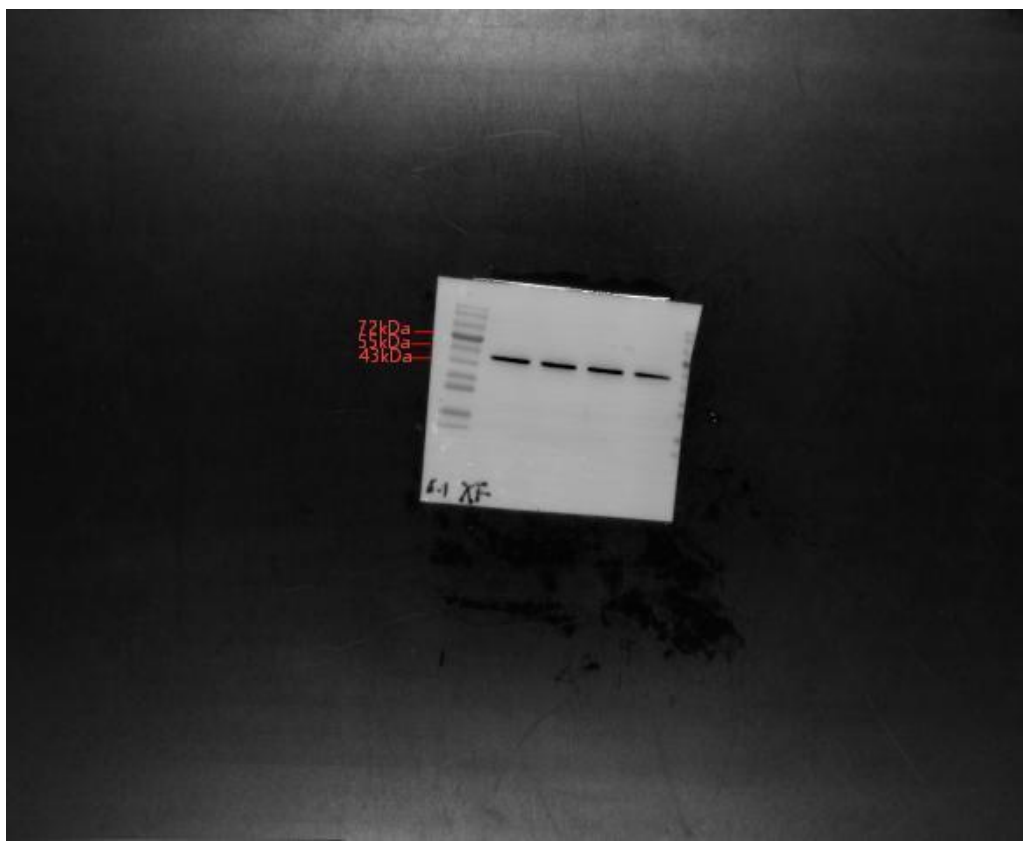

Flag

HA

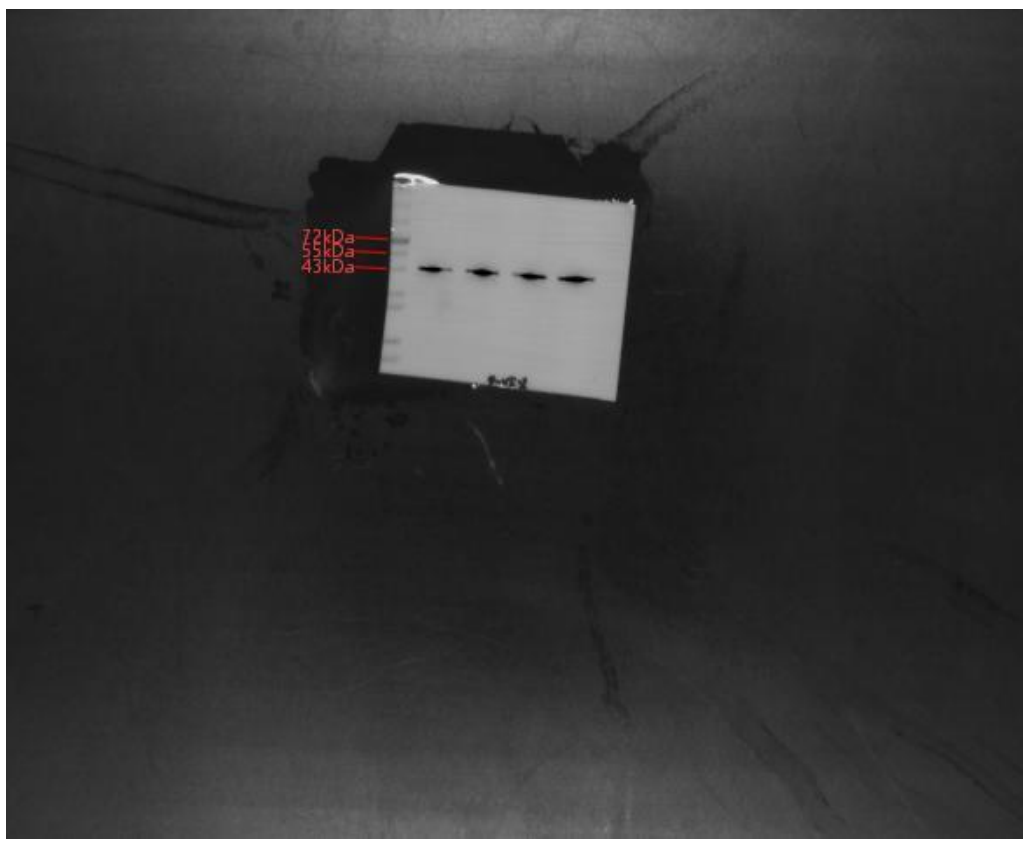

ACTIN

FIG.6H

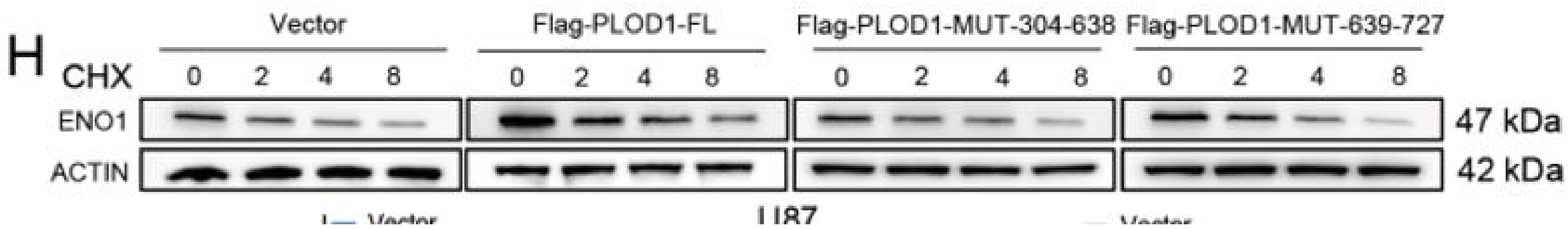

Control

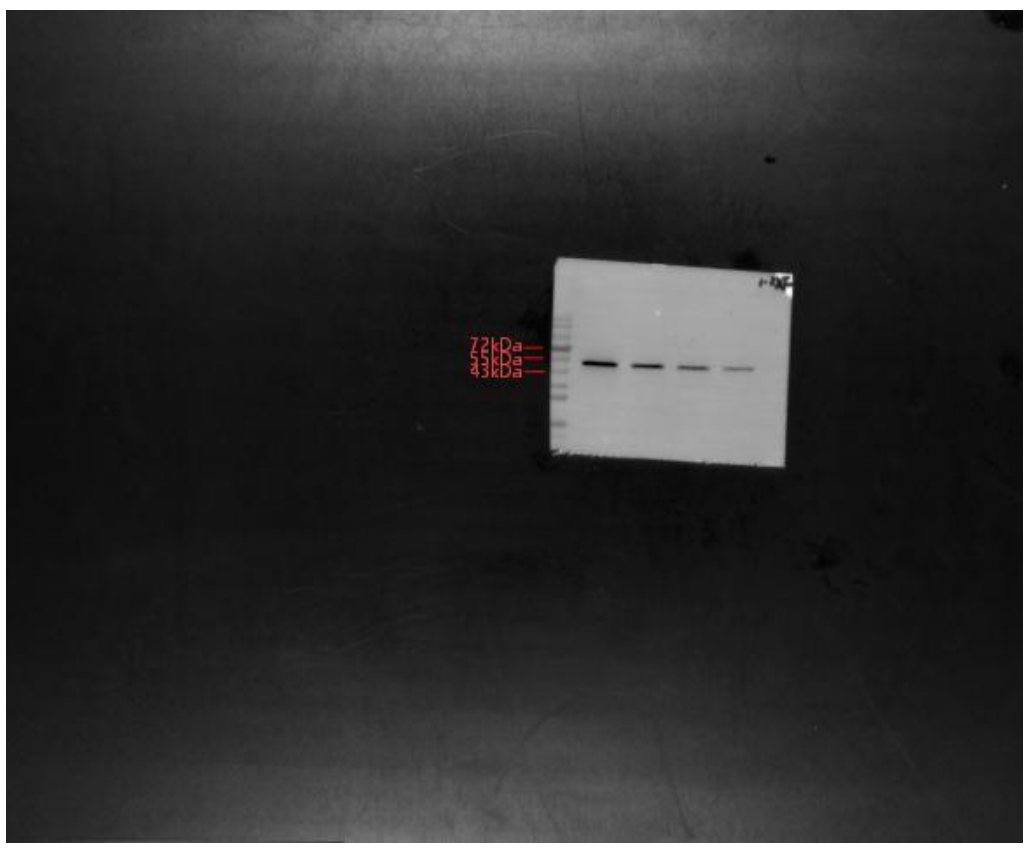

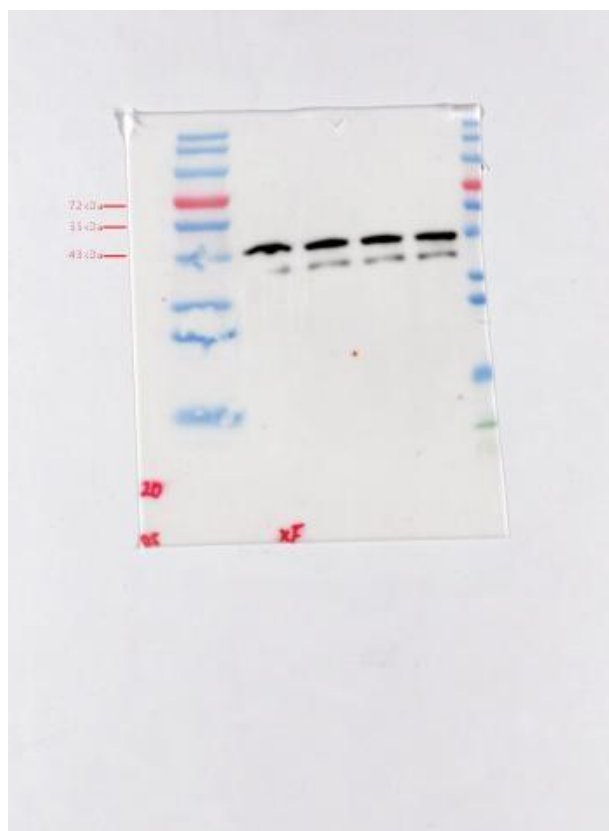

ENO1

ACTIN

WT

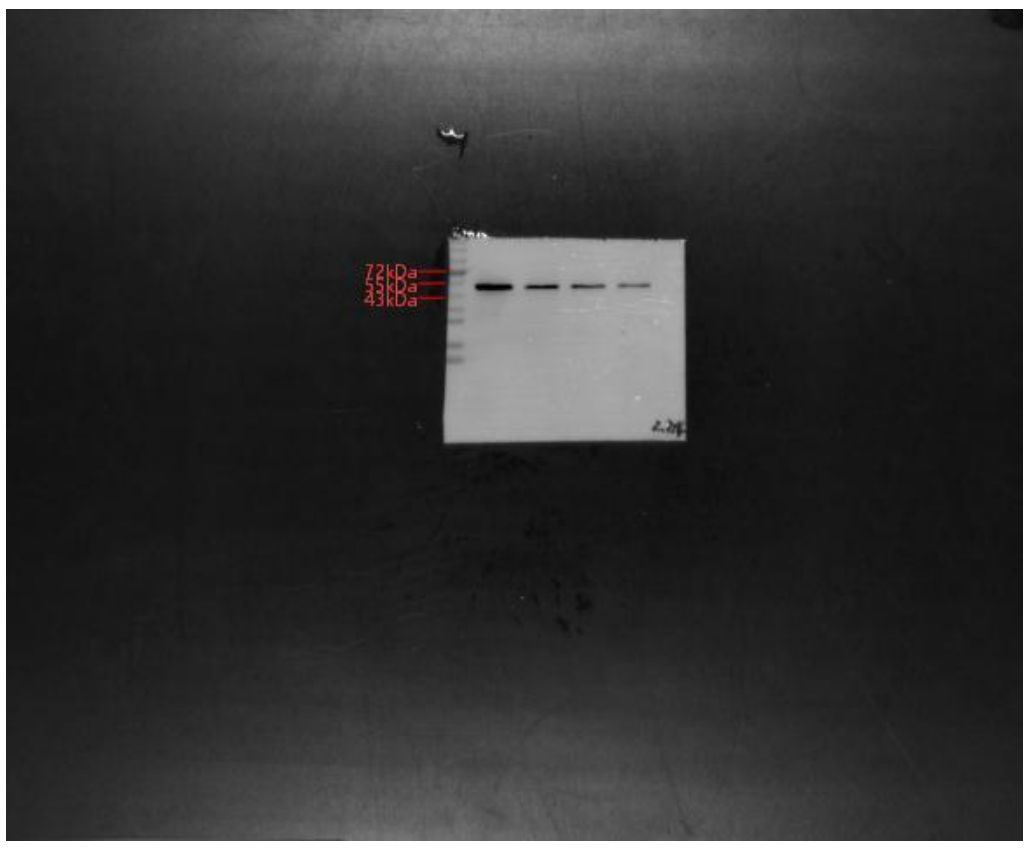

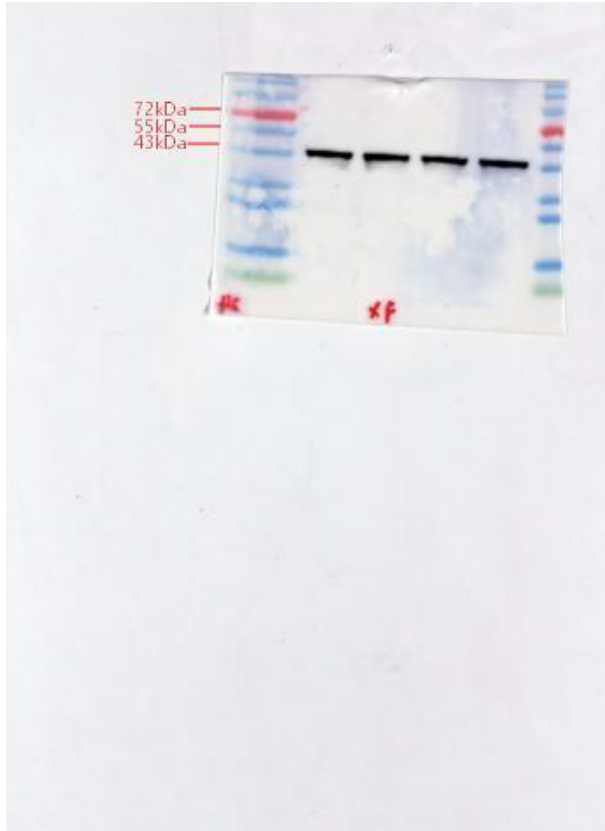

ENO1 ACTIN

MUT-304-638

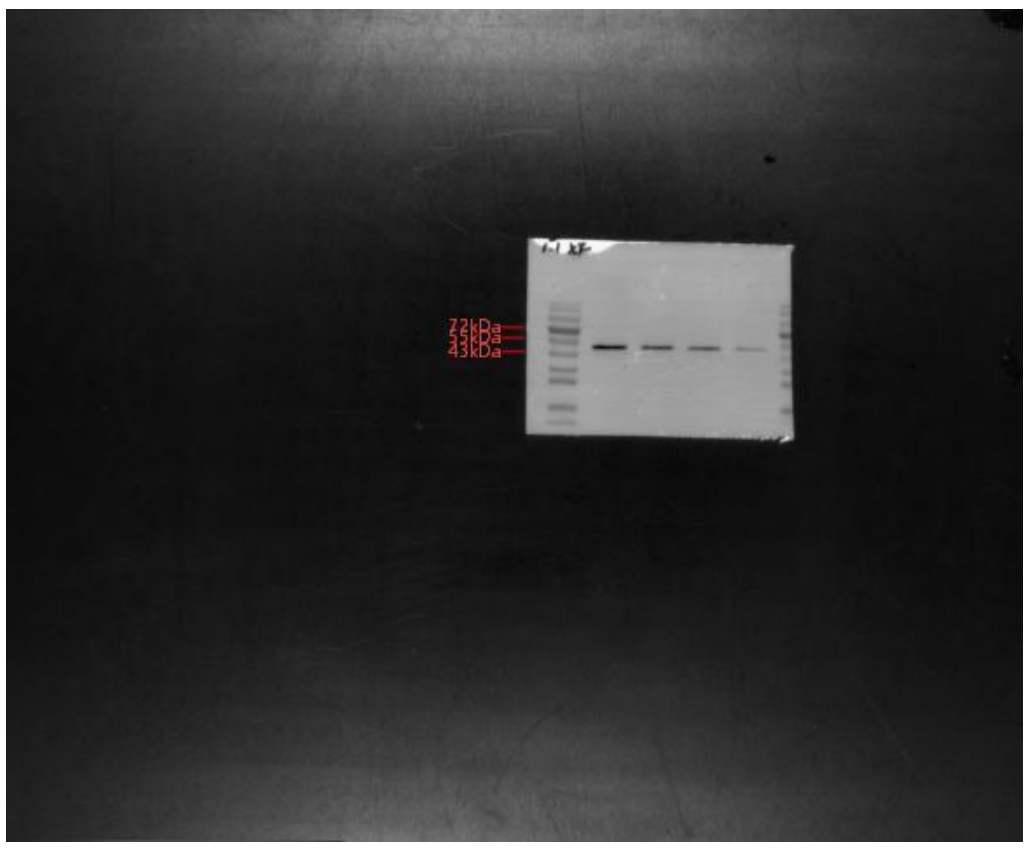

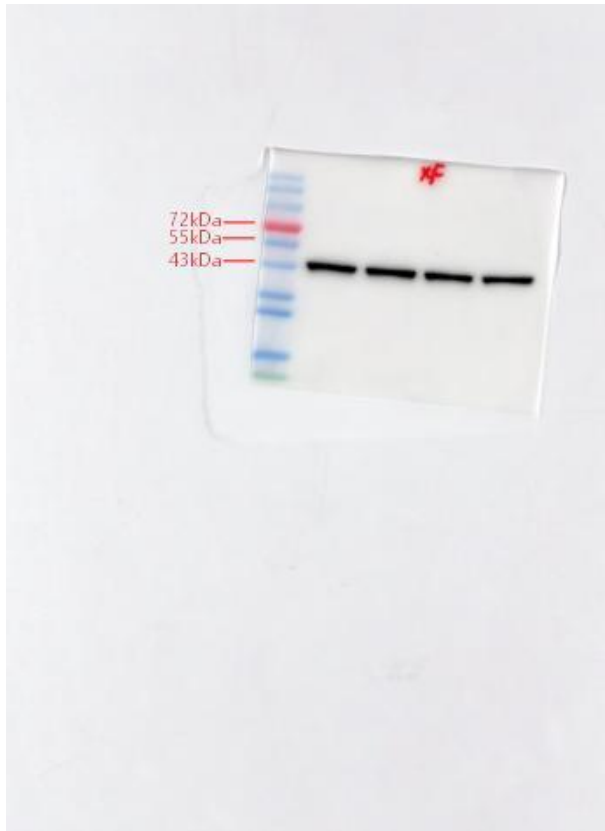

ENO1 ACTIN

MUT-639-727

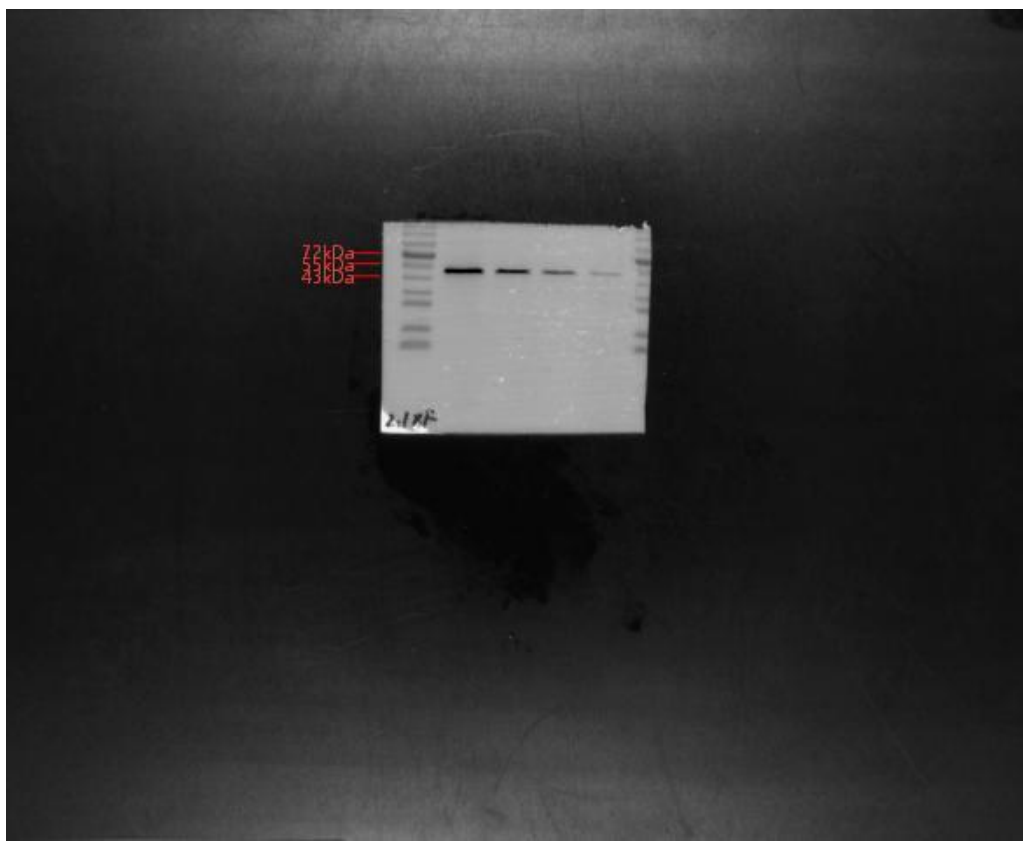

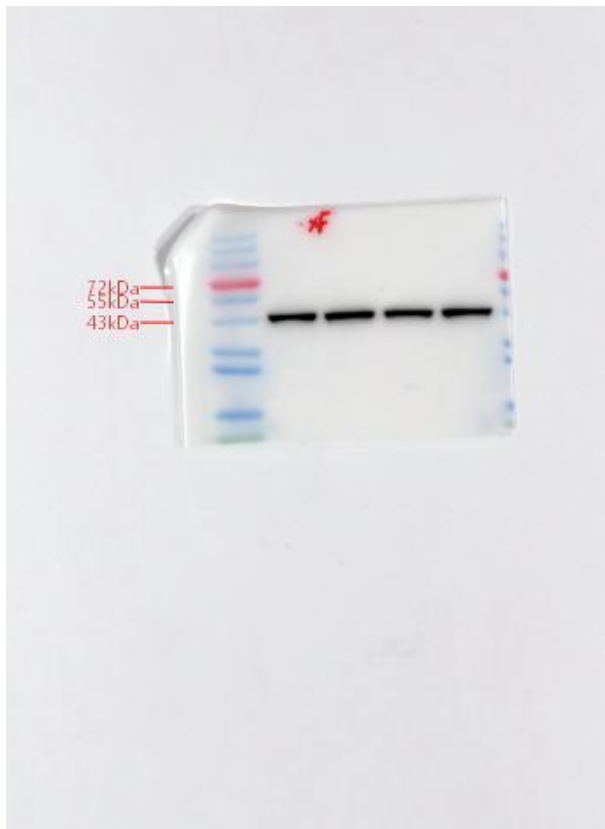

ENO1 ACTIN

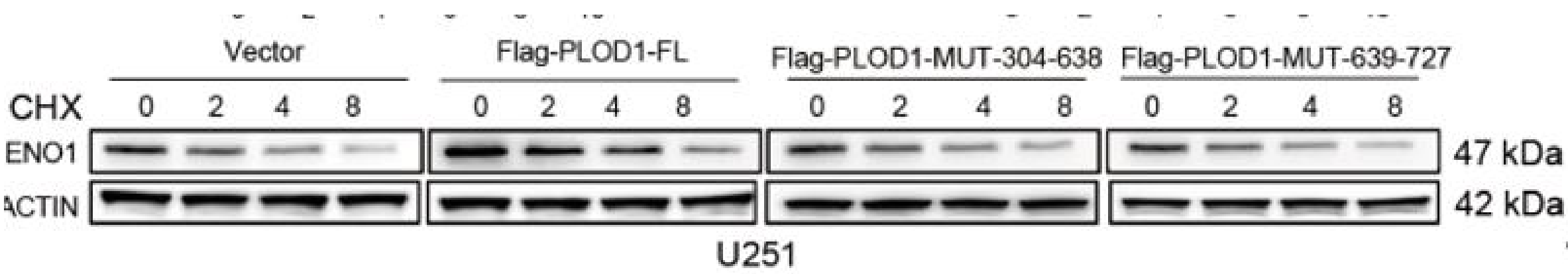

Control

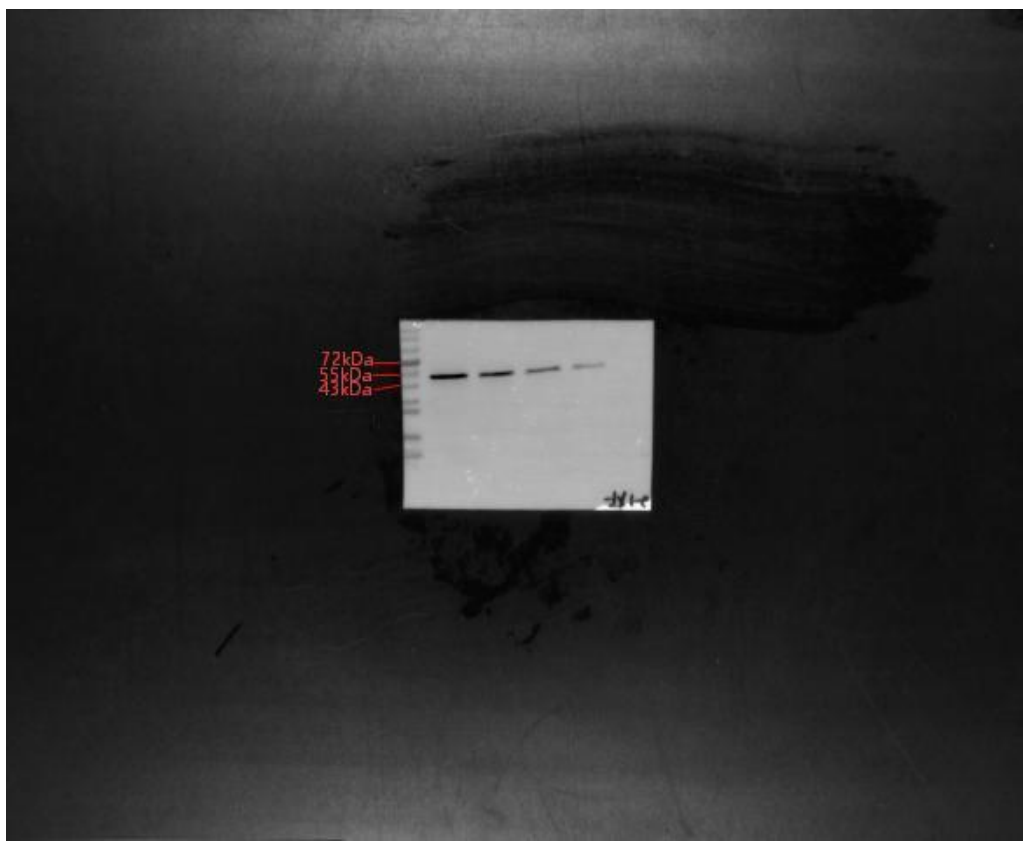

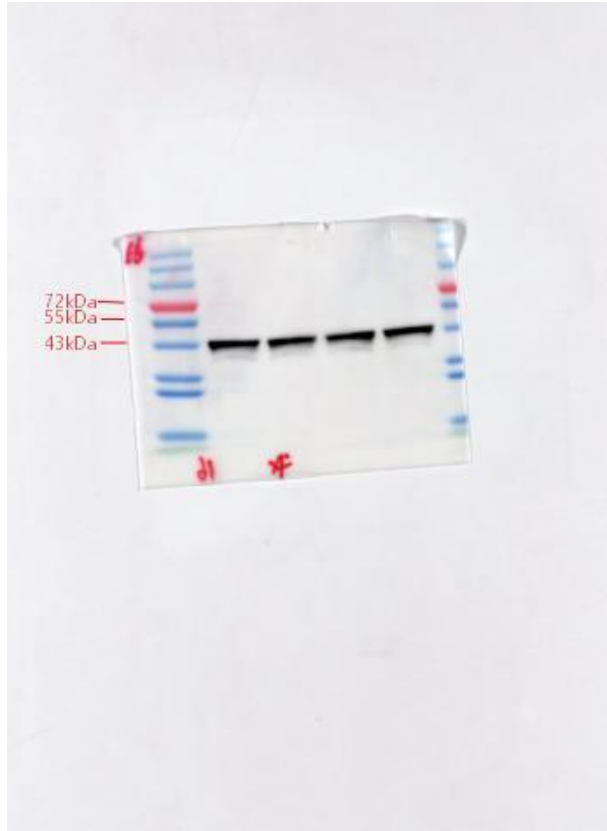

ENO1 ACTIN

WT

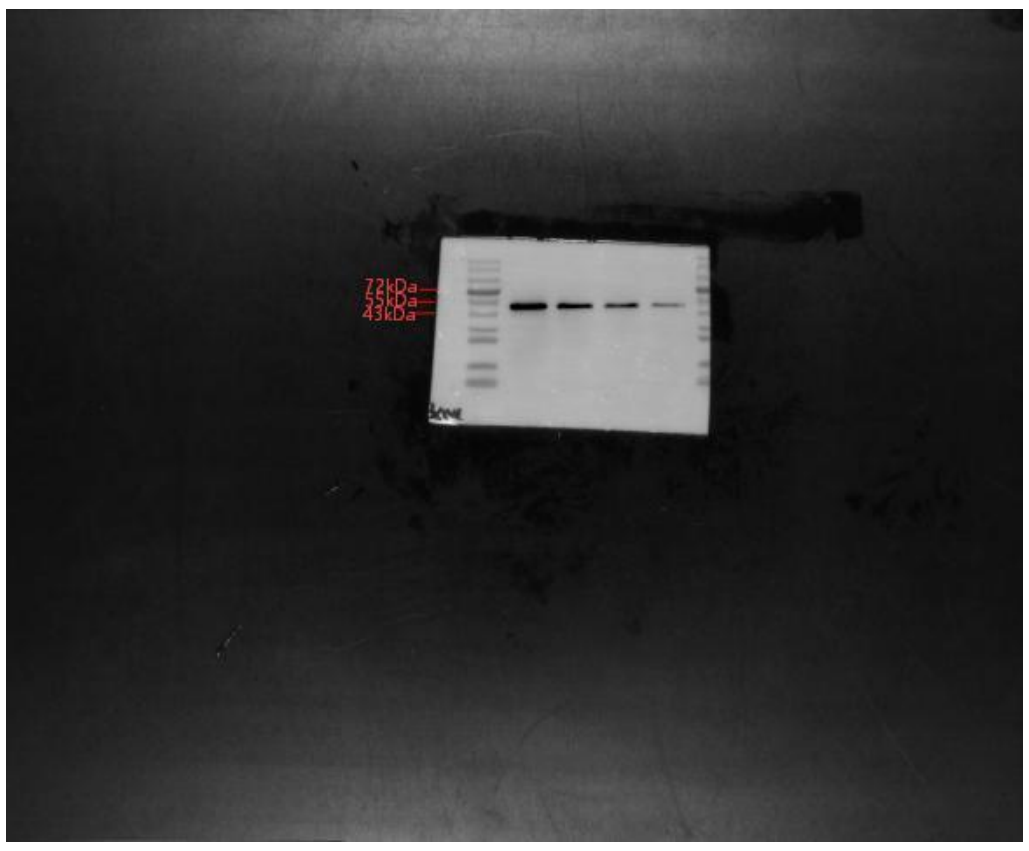

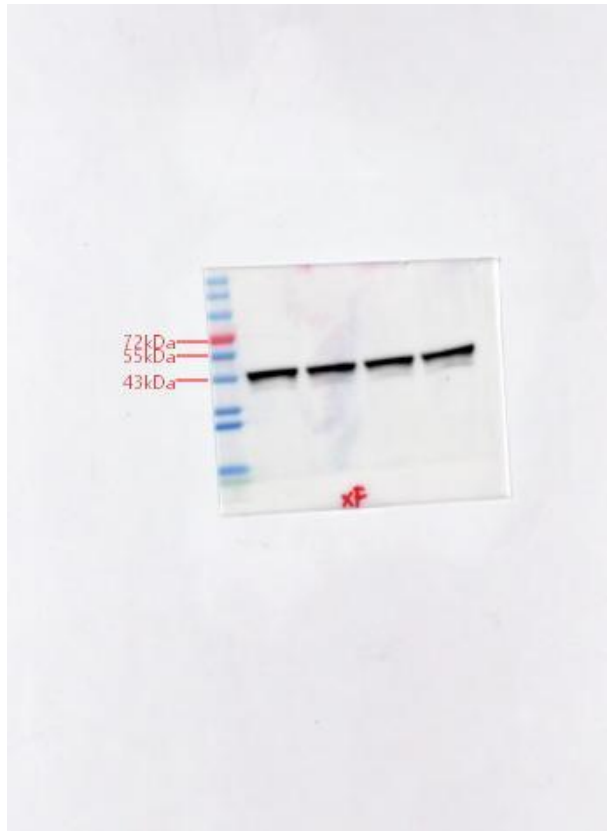

ENO1 ACTIN

MUT-304-638

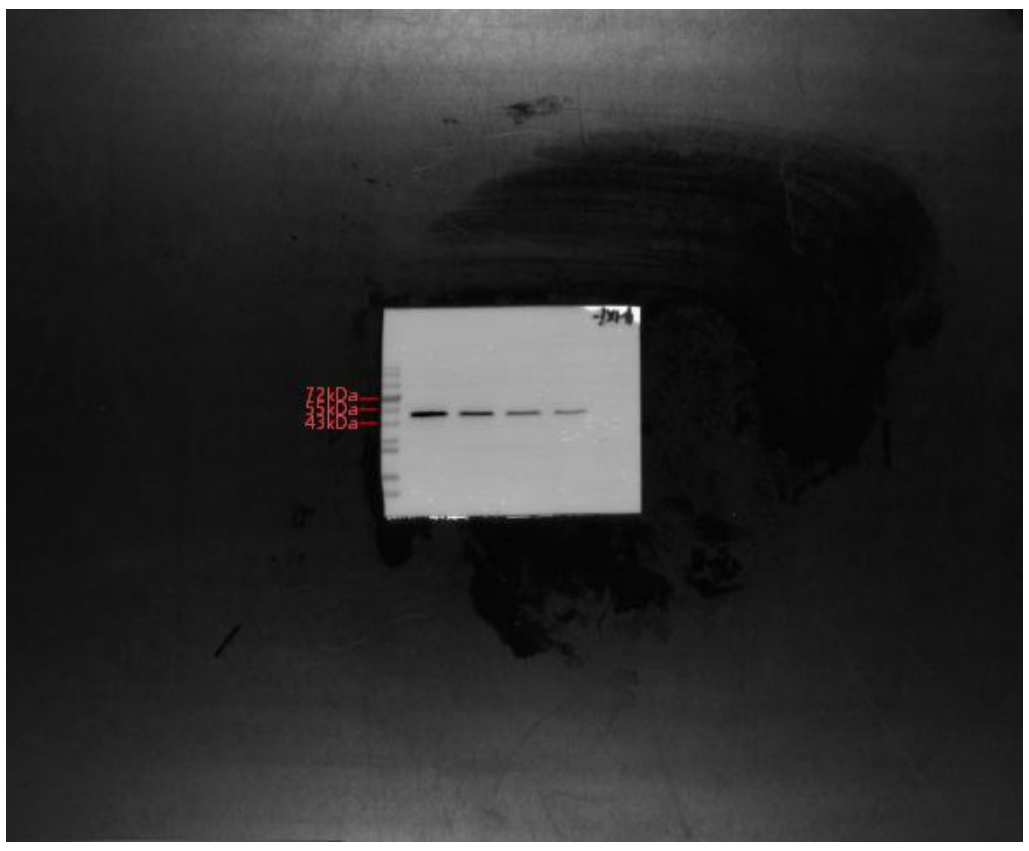

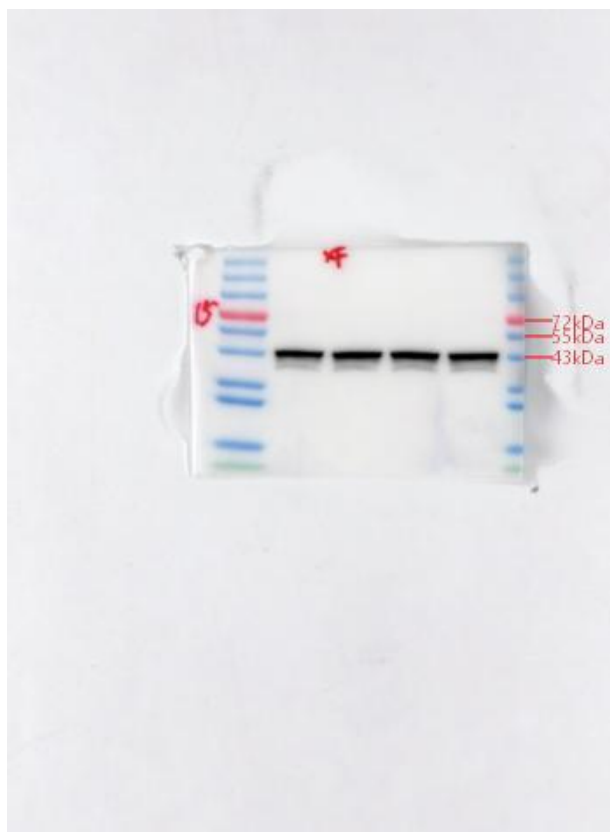

ENO1 ACTIN

MUT-639-727

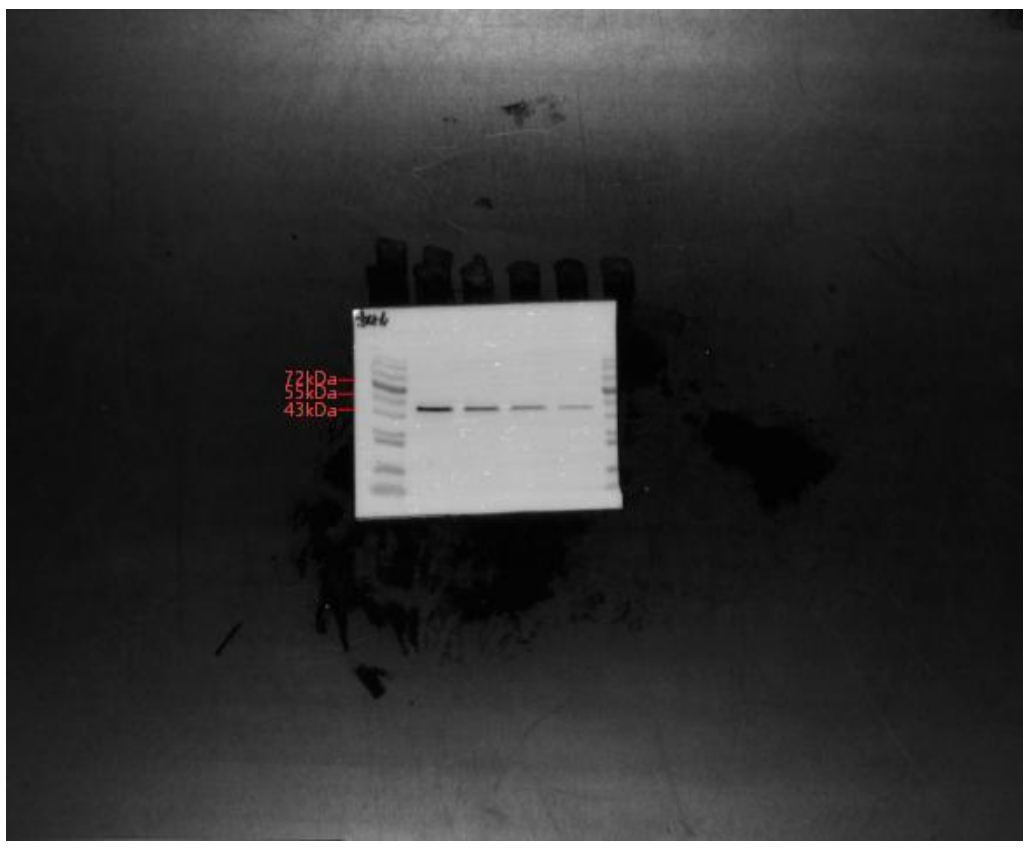

ENO1

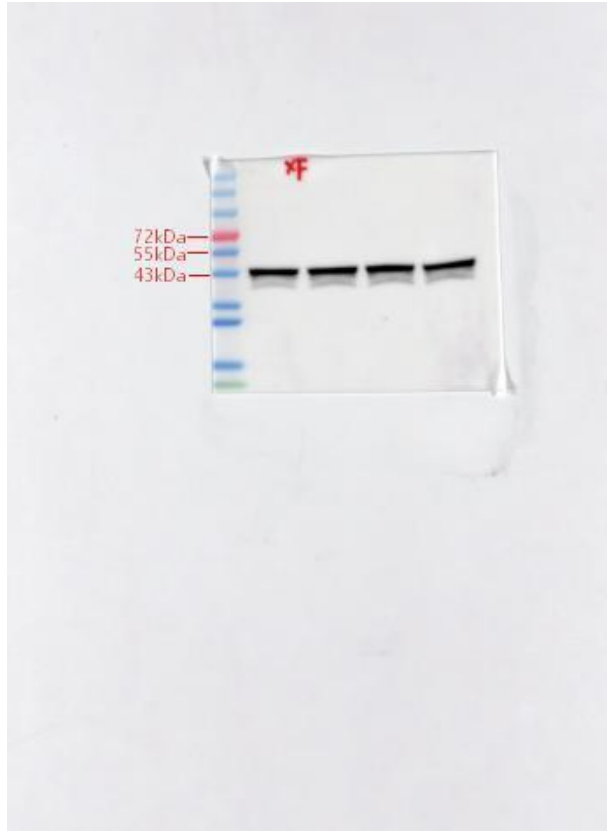

ACTIN

FIG.S1B

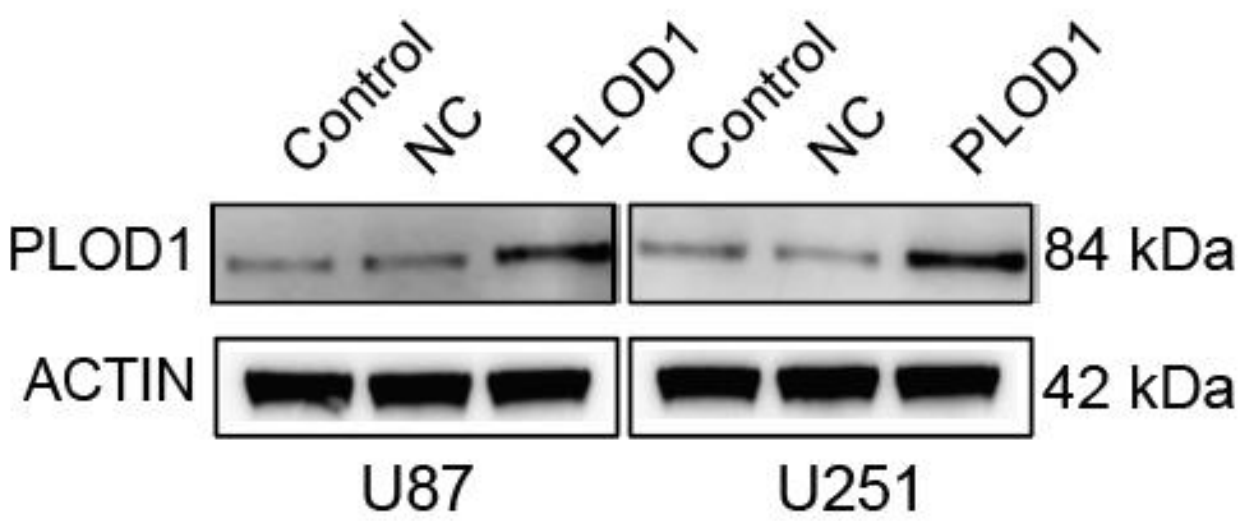

U87

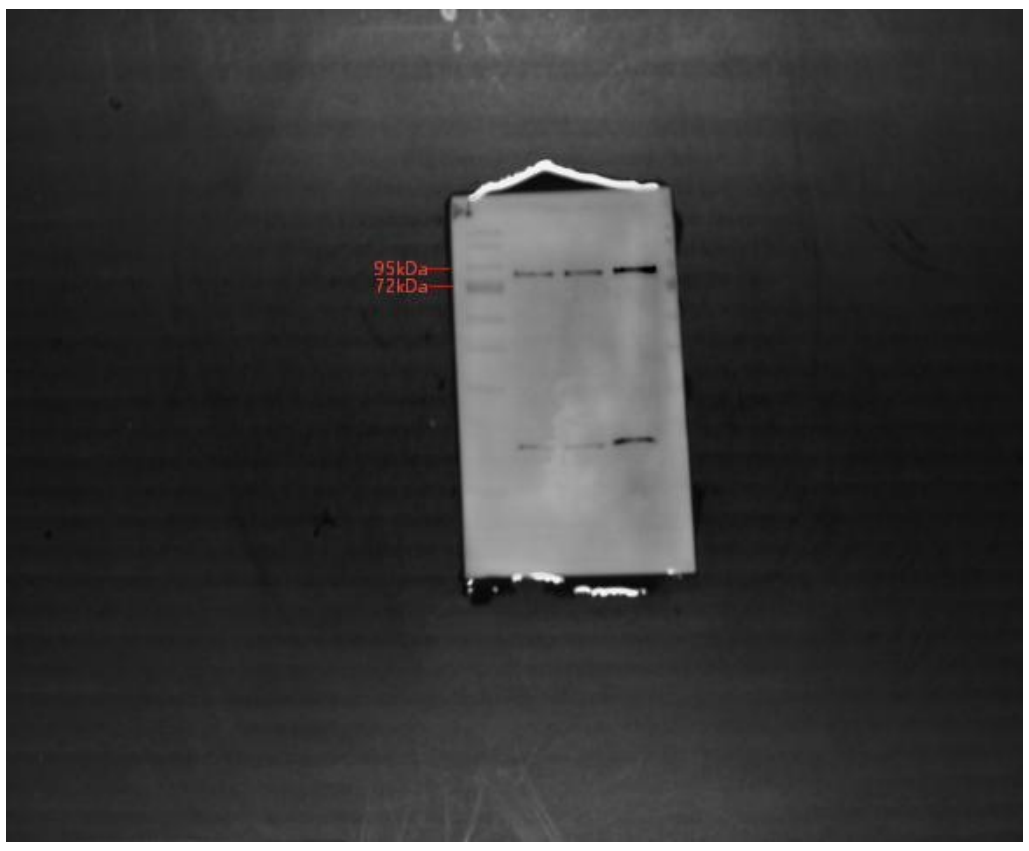

PLOD1

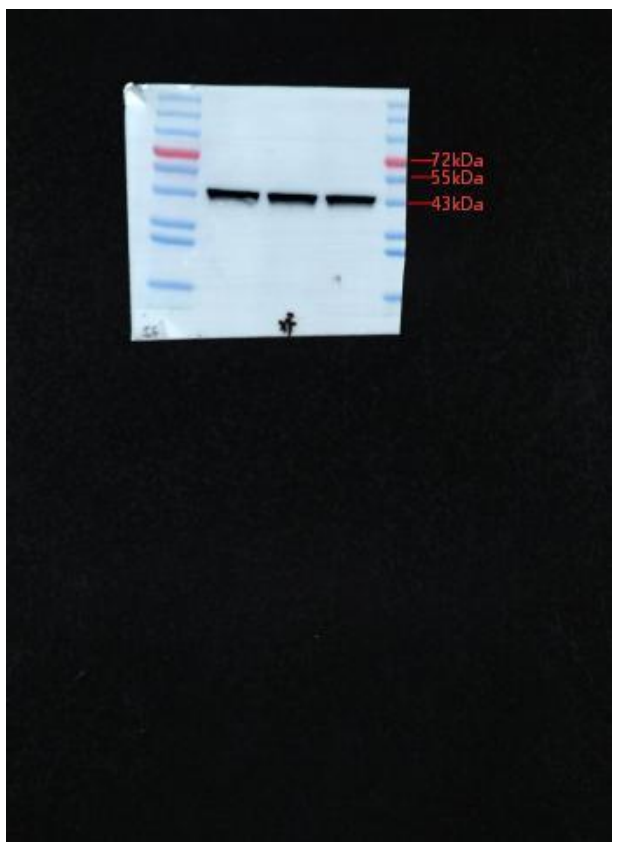

ACTIN

U251

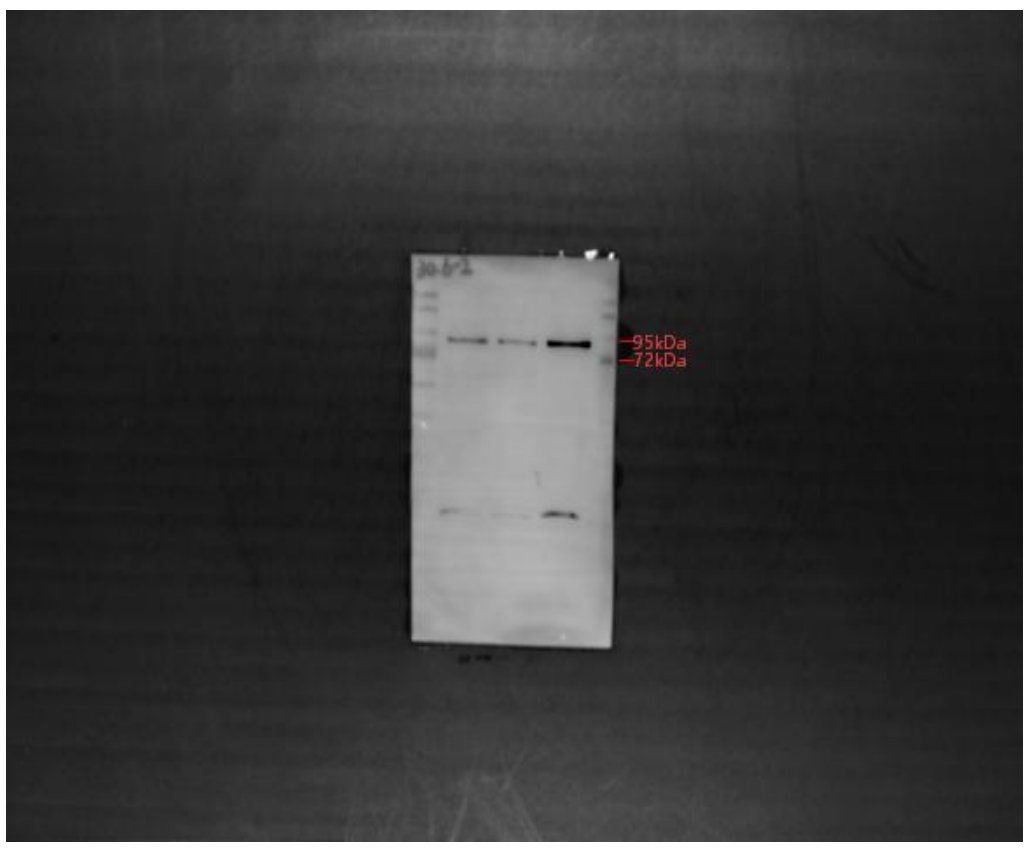

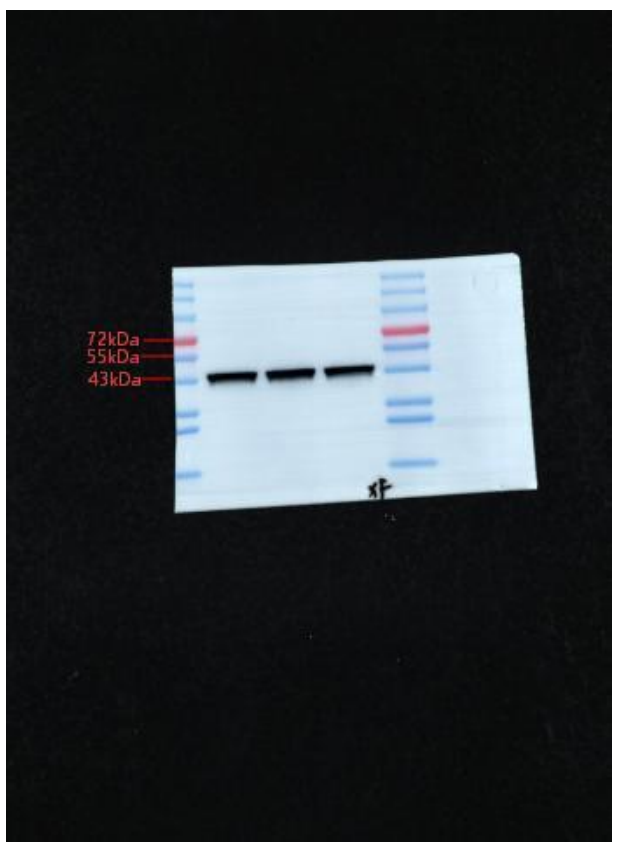

PLOD1 ACTIN

FIG.S1D

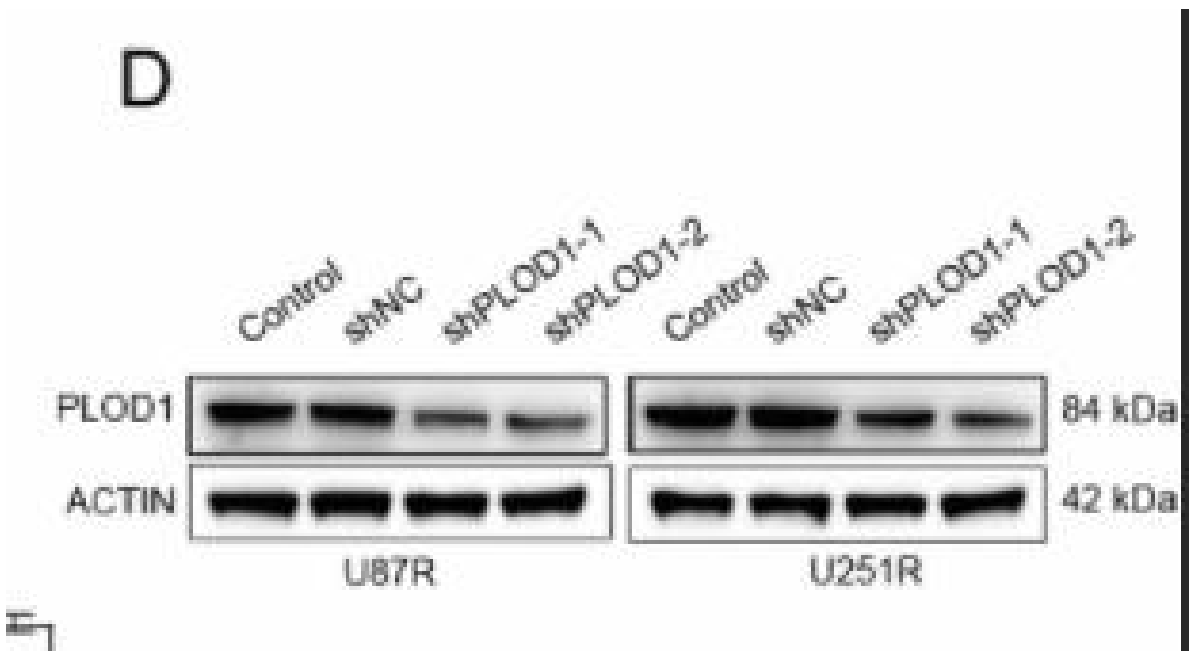

U87R

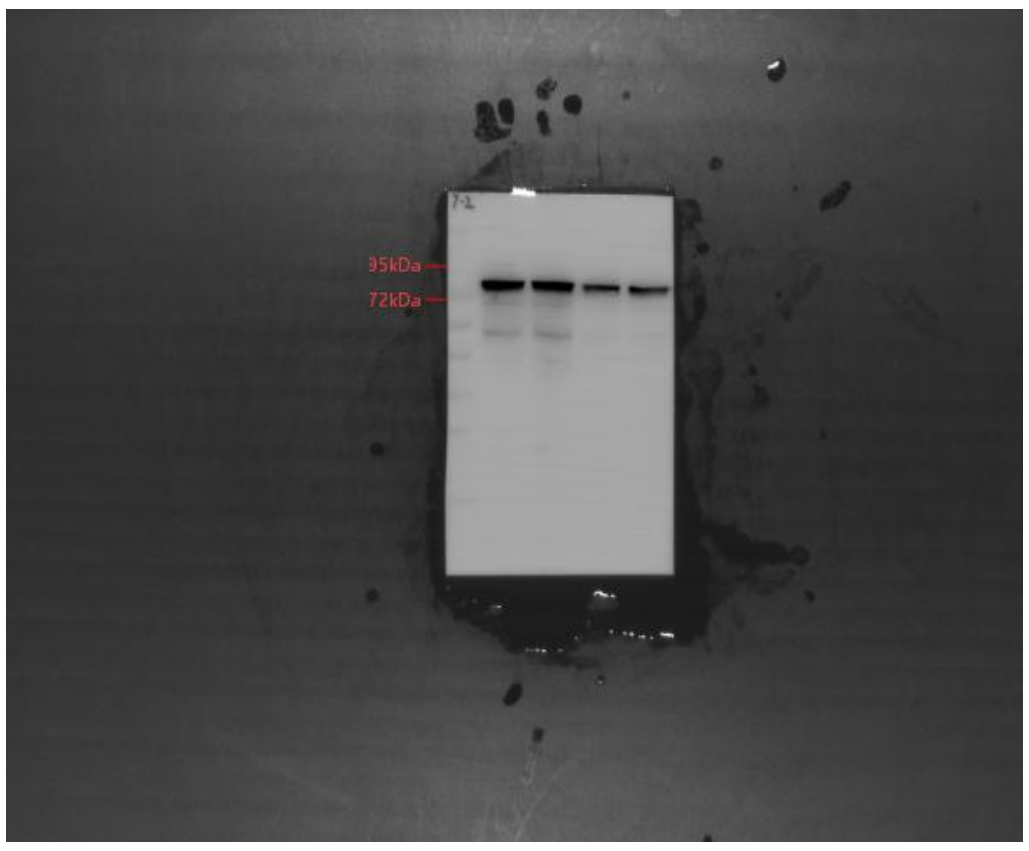

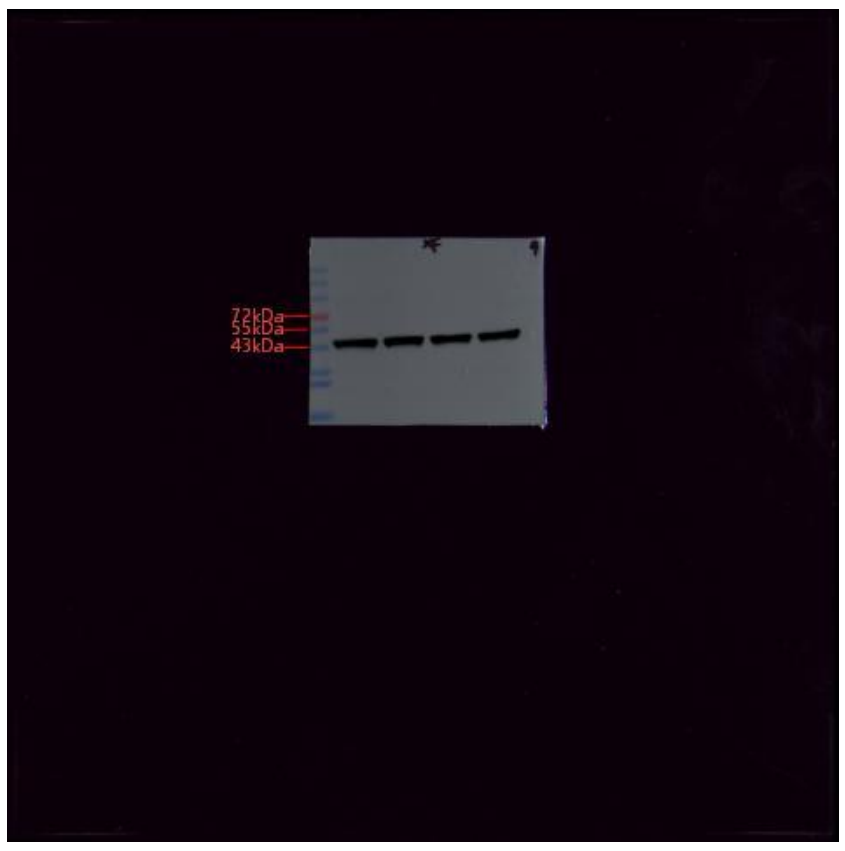

PLOD1 ACTIN

U251R

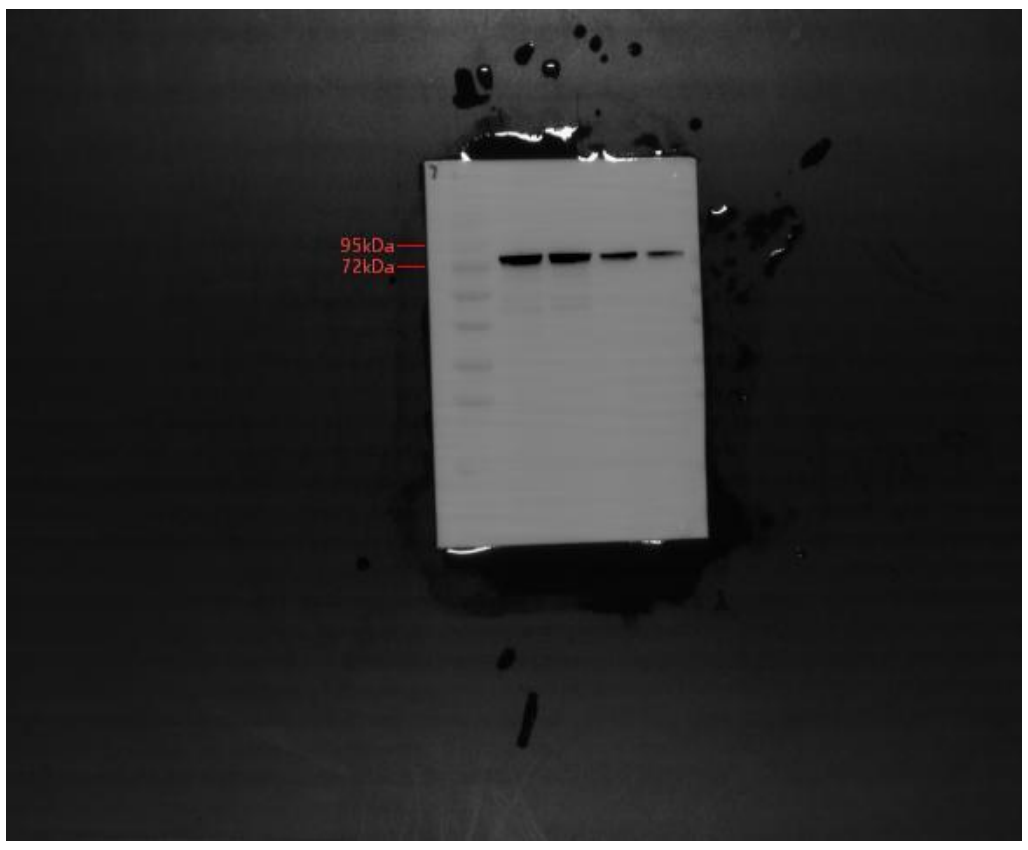

PLOD1

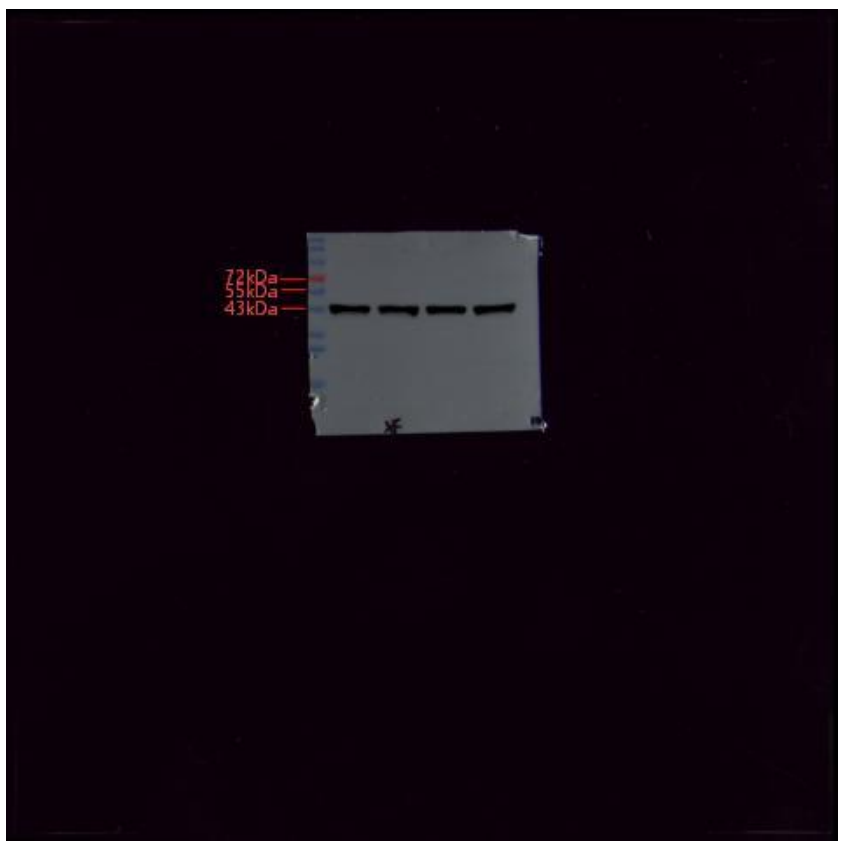

ACTIN

FIG.S1F

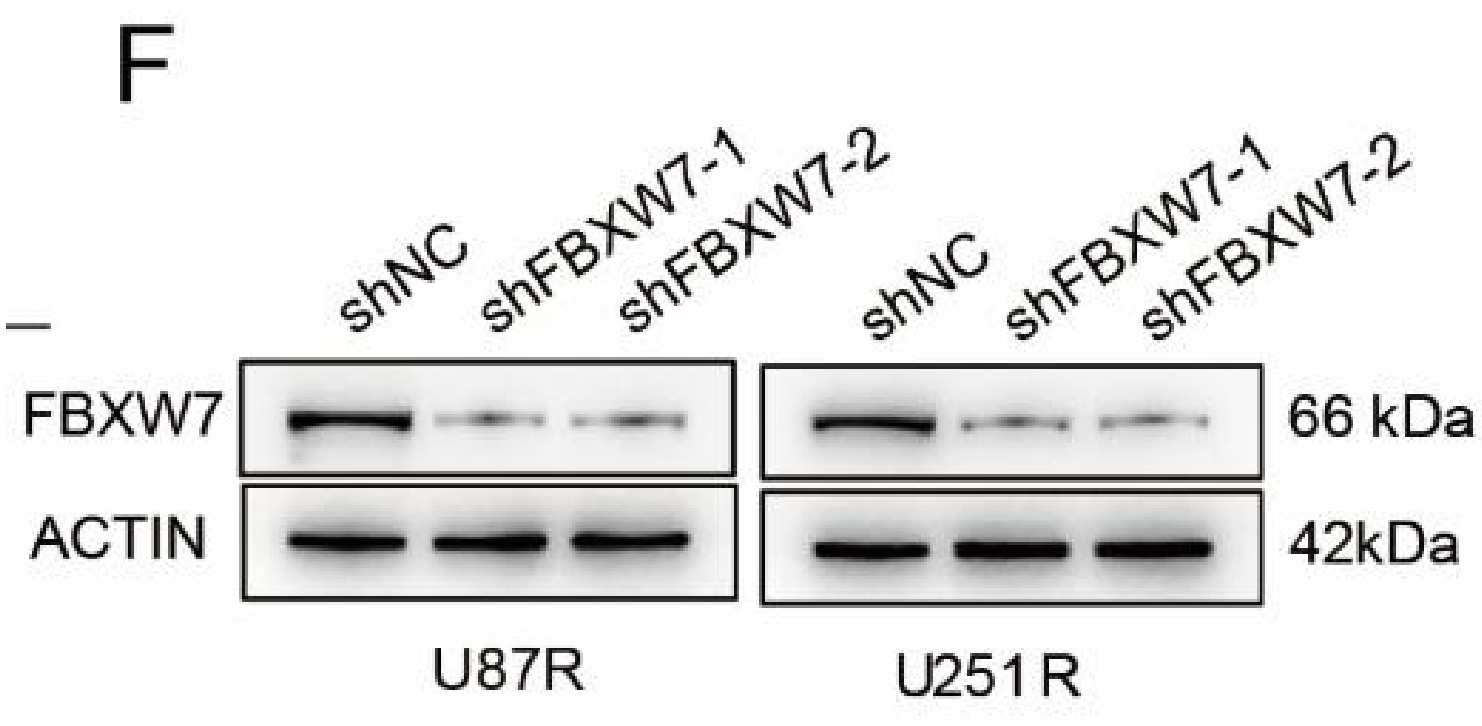

U87R

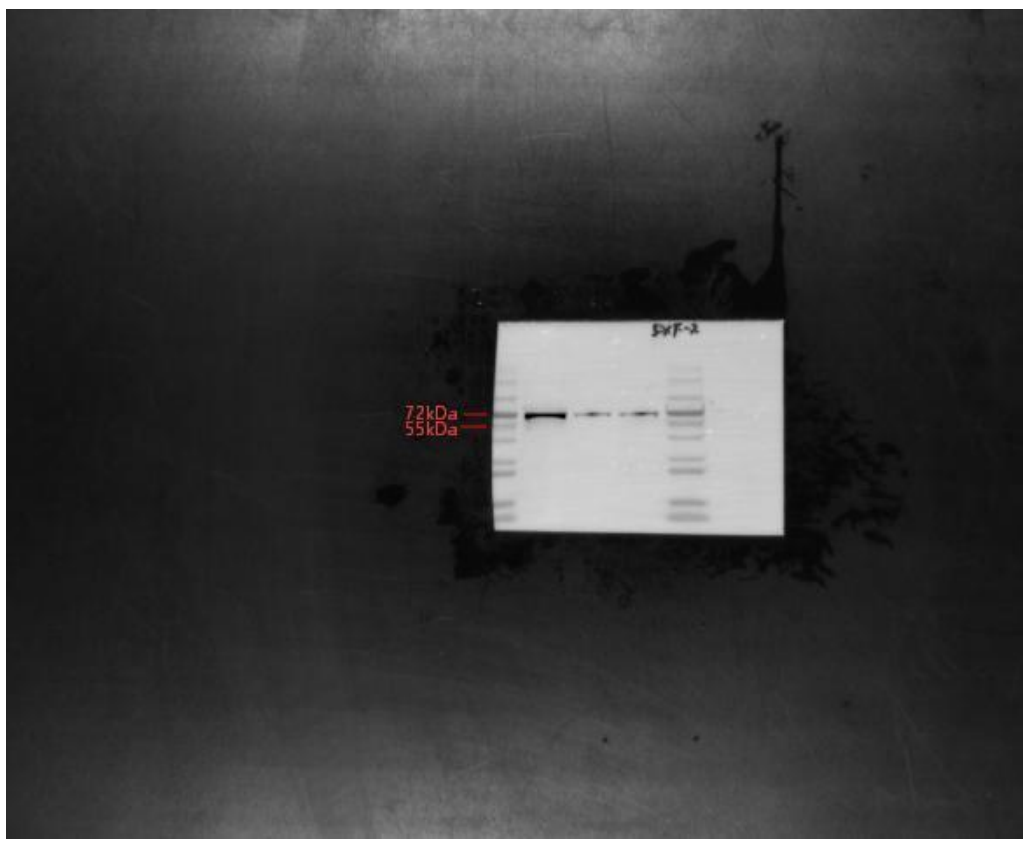

FBXW7

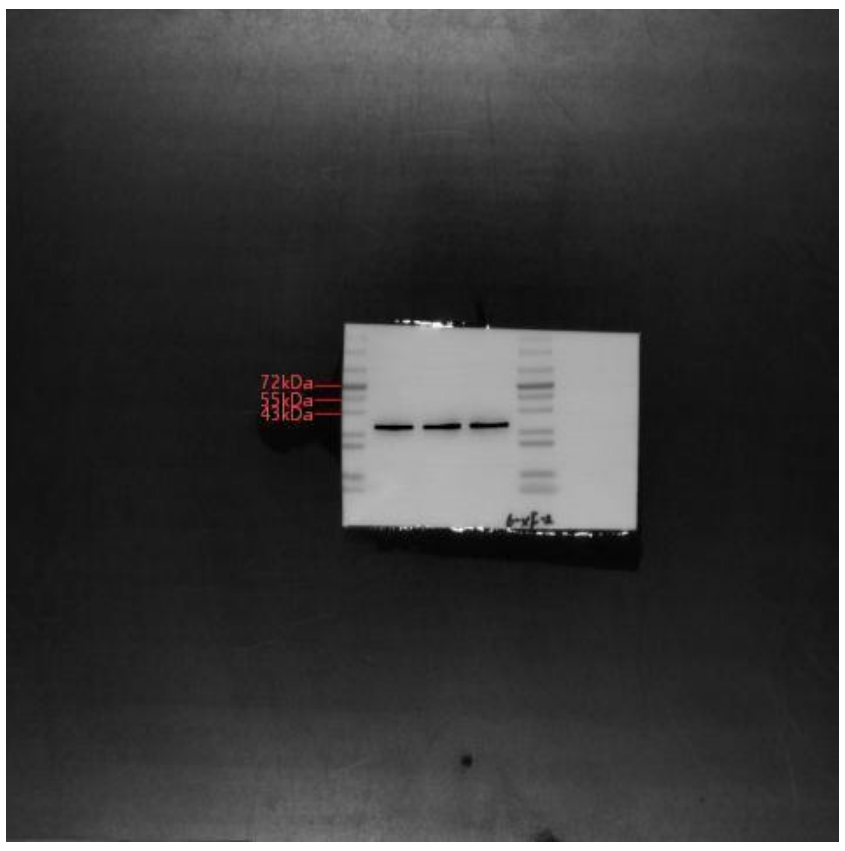

ACTIN

U251R

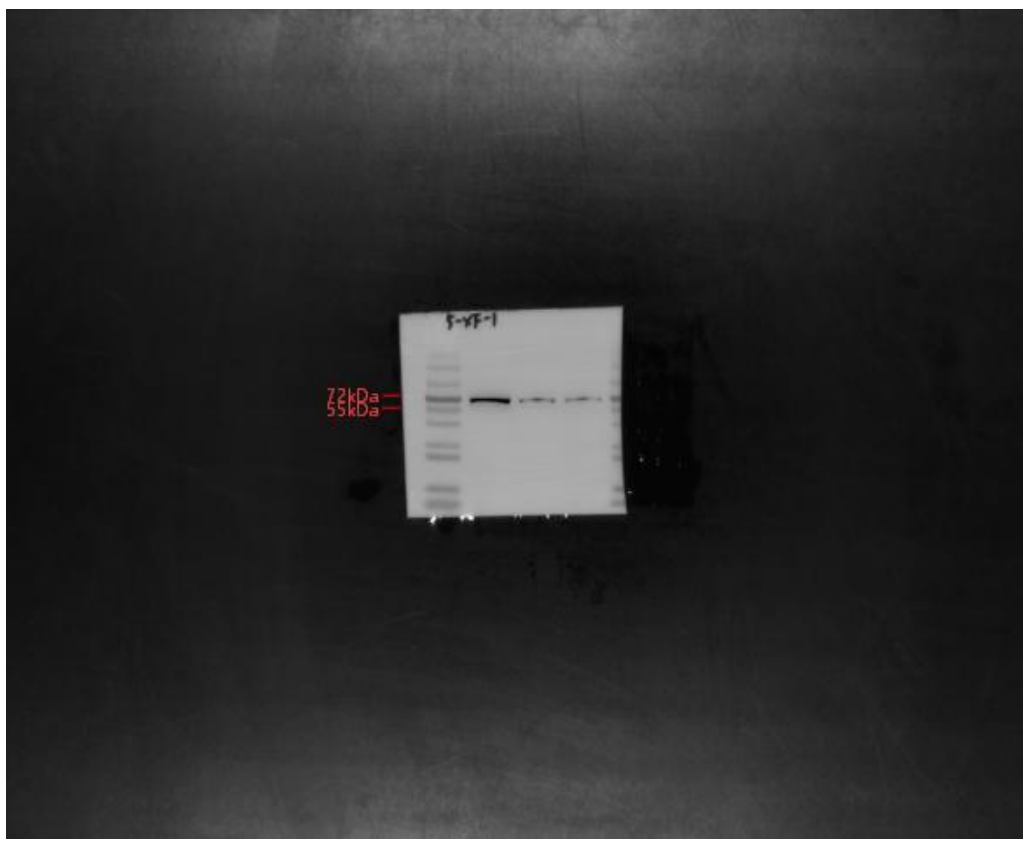

FBXW7

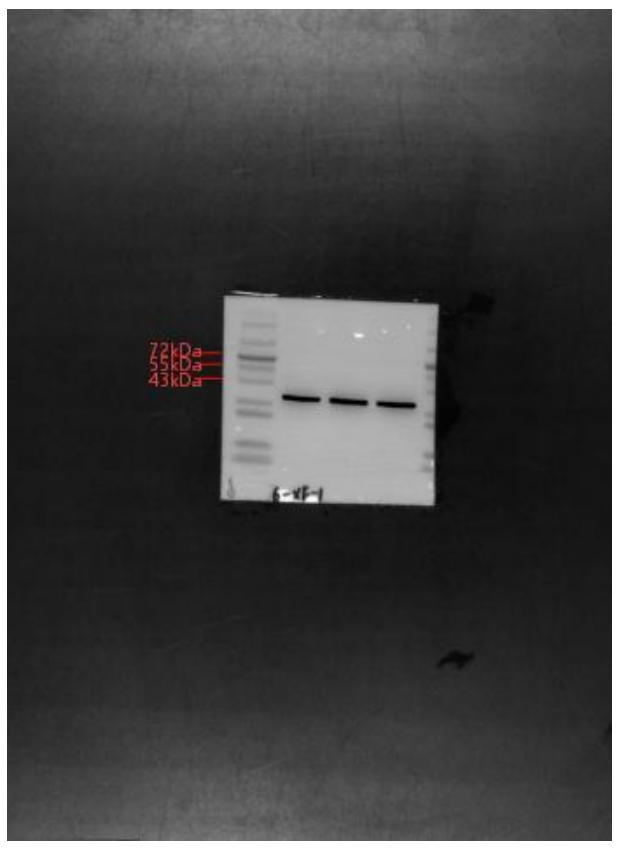

ACTIN

FIG.S1G

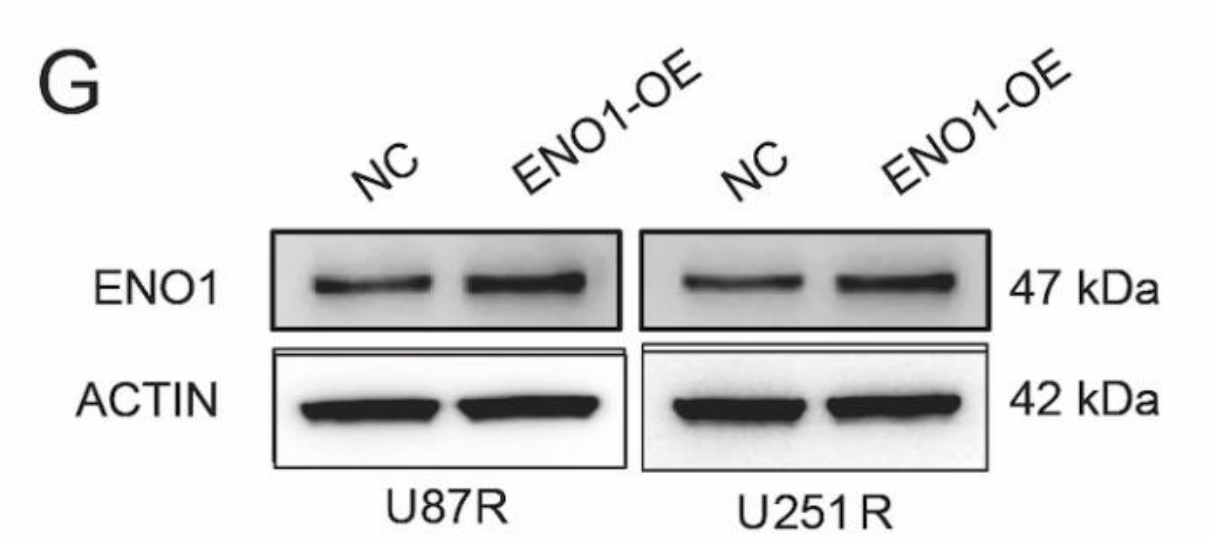

U87R

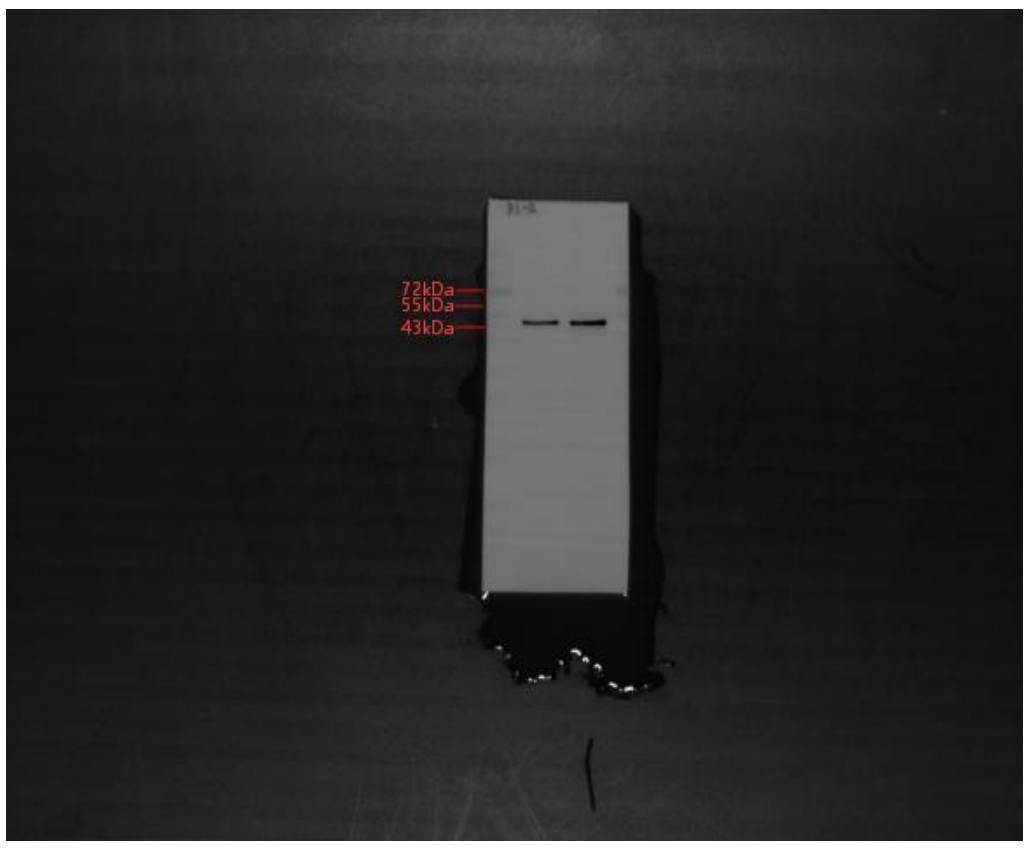

ENO1

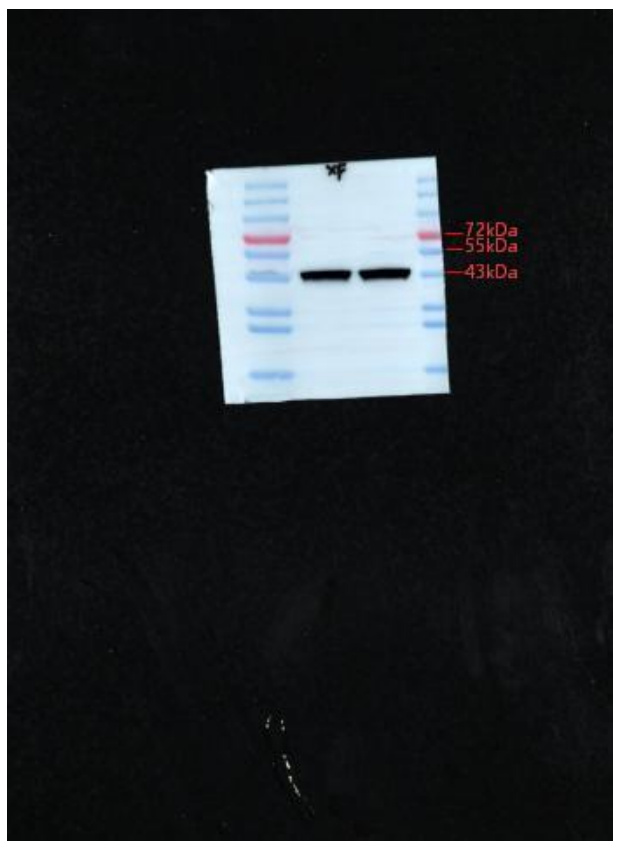

ACTIN

U251R

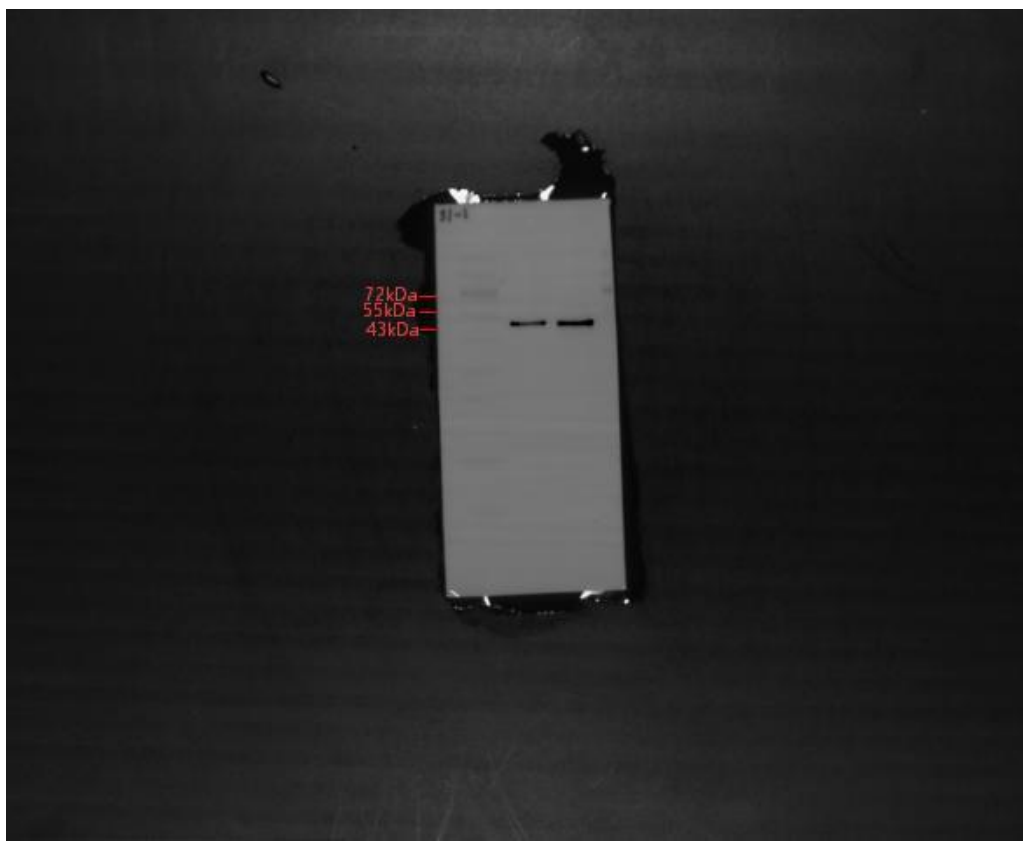

ENO1

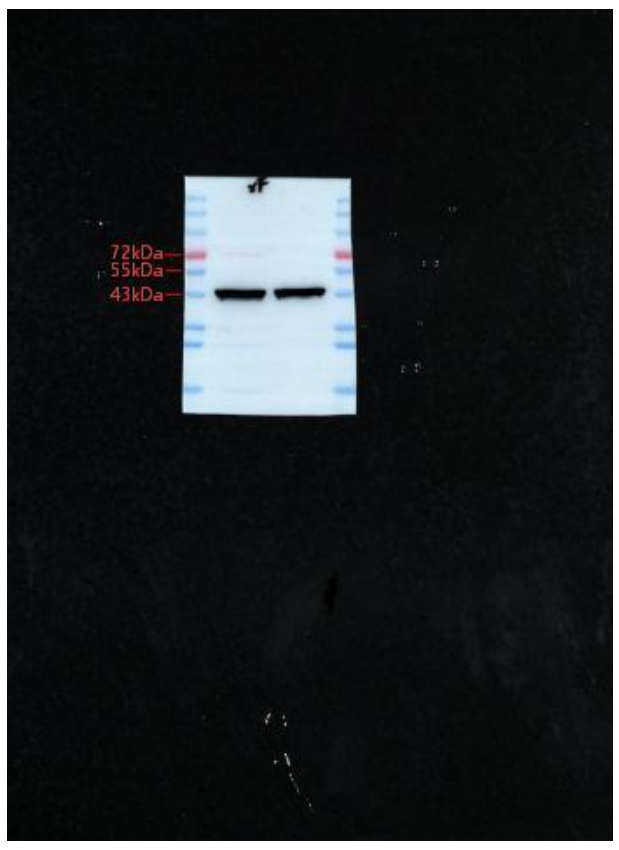

ACTIN

FIG.S1H

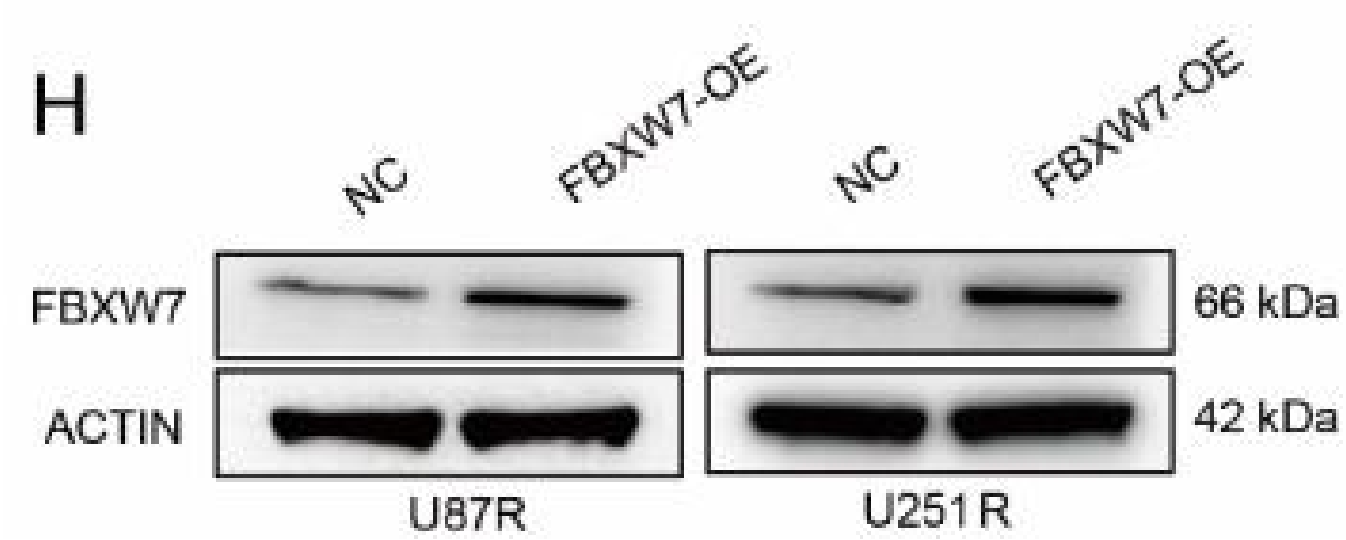

U87R

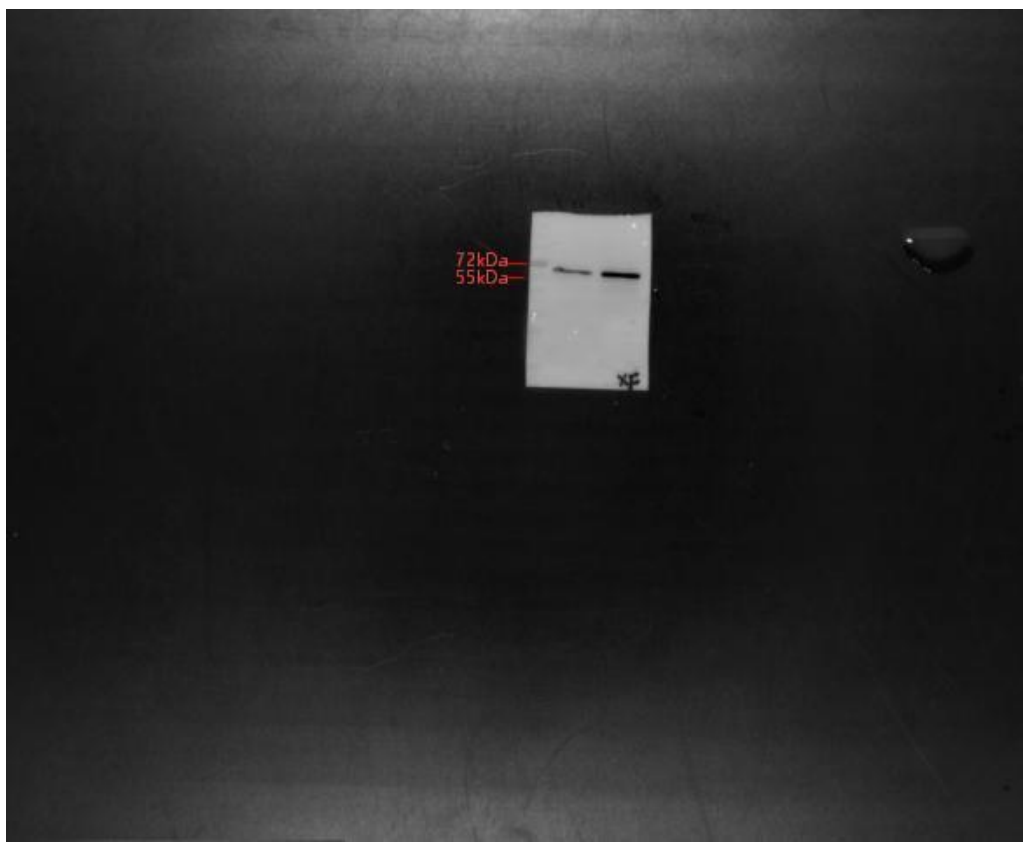

FBXW7

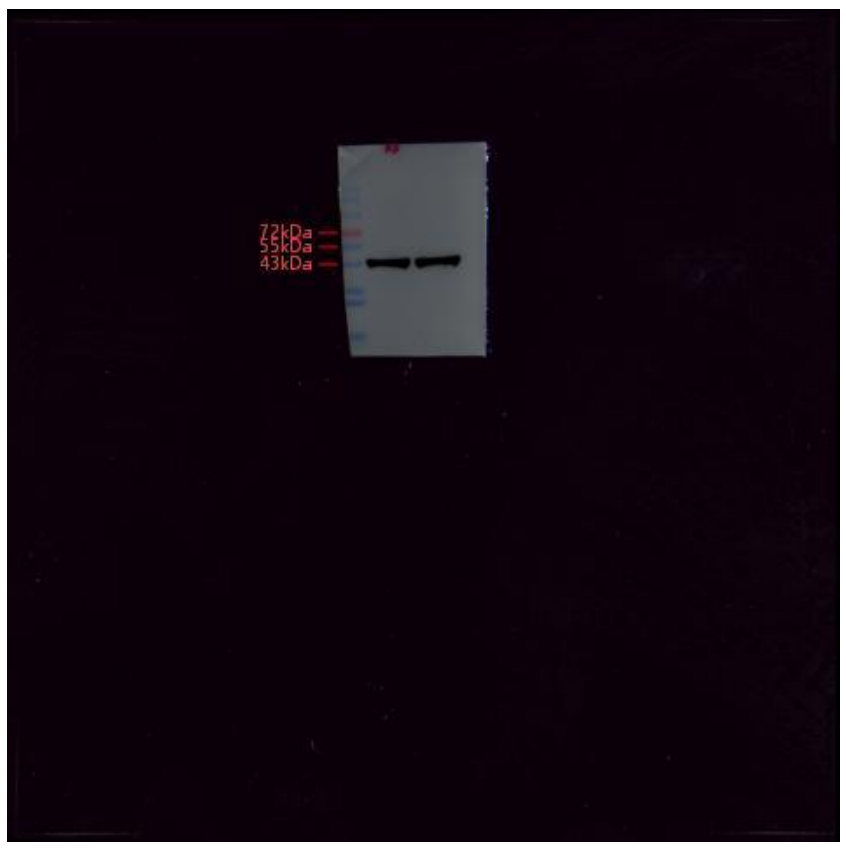

ACTIN

U251R

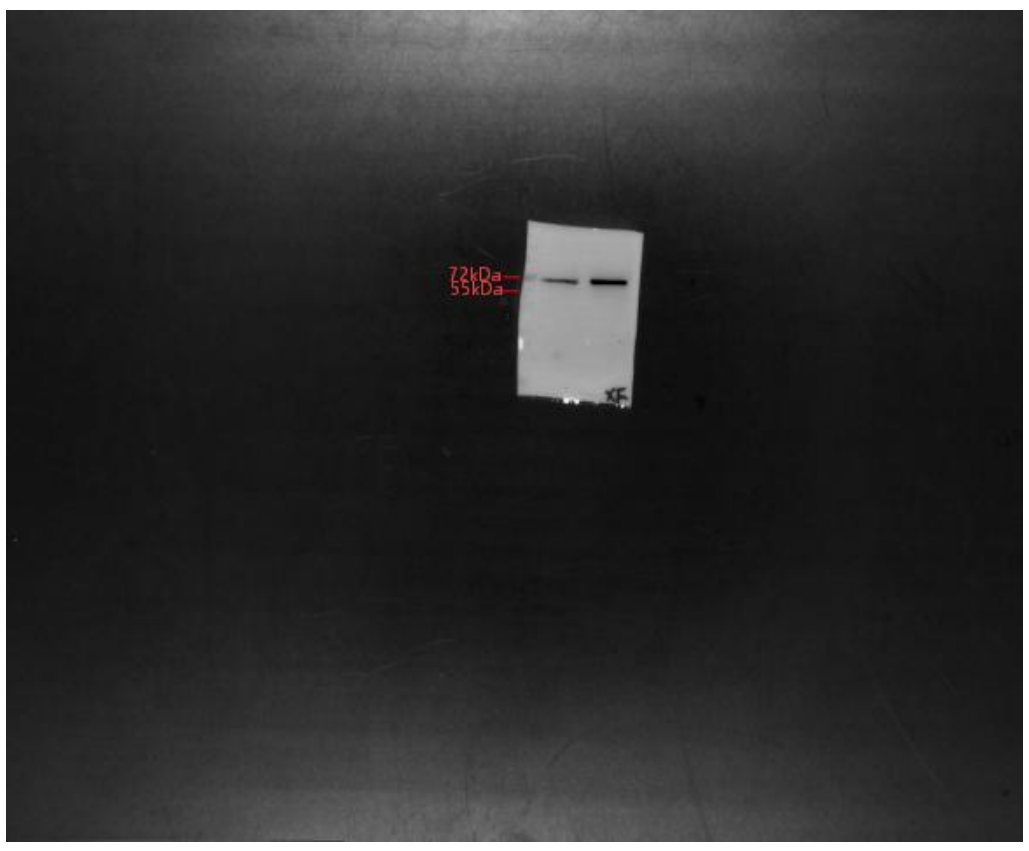

FBXW7

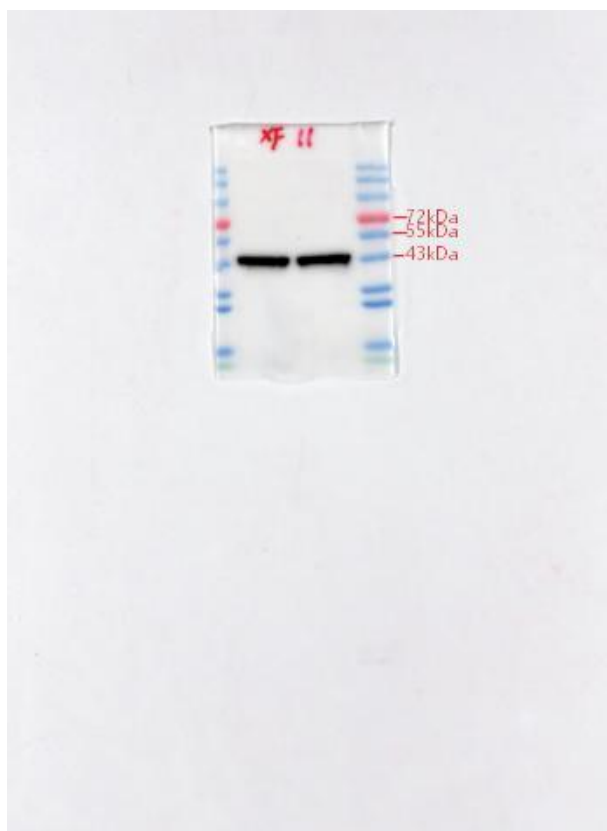

ACTIN
