# Supplementary material for: PLOD1 Catalytic Activity Stabilizes ENO1 by Limiting FBXW7‐dependent Degradation to Promote Glycolysis and TMZ Resistance in Glioblastoma
Source: Adv Sci (Weinh). 2026 Sep 27:e78070. Online ahead of print. doi: 10.1002/advs.78070 (PMC13616334; doi:10.1002/advs.78070)

Figure2H

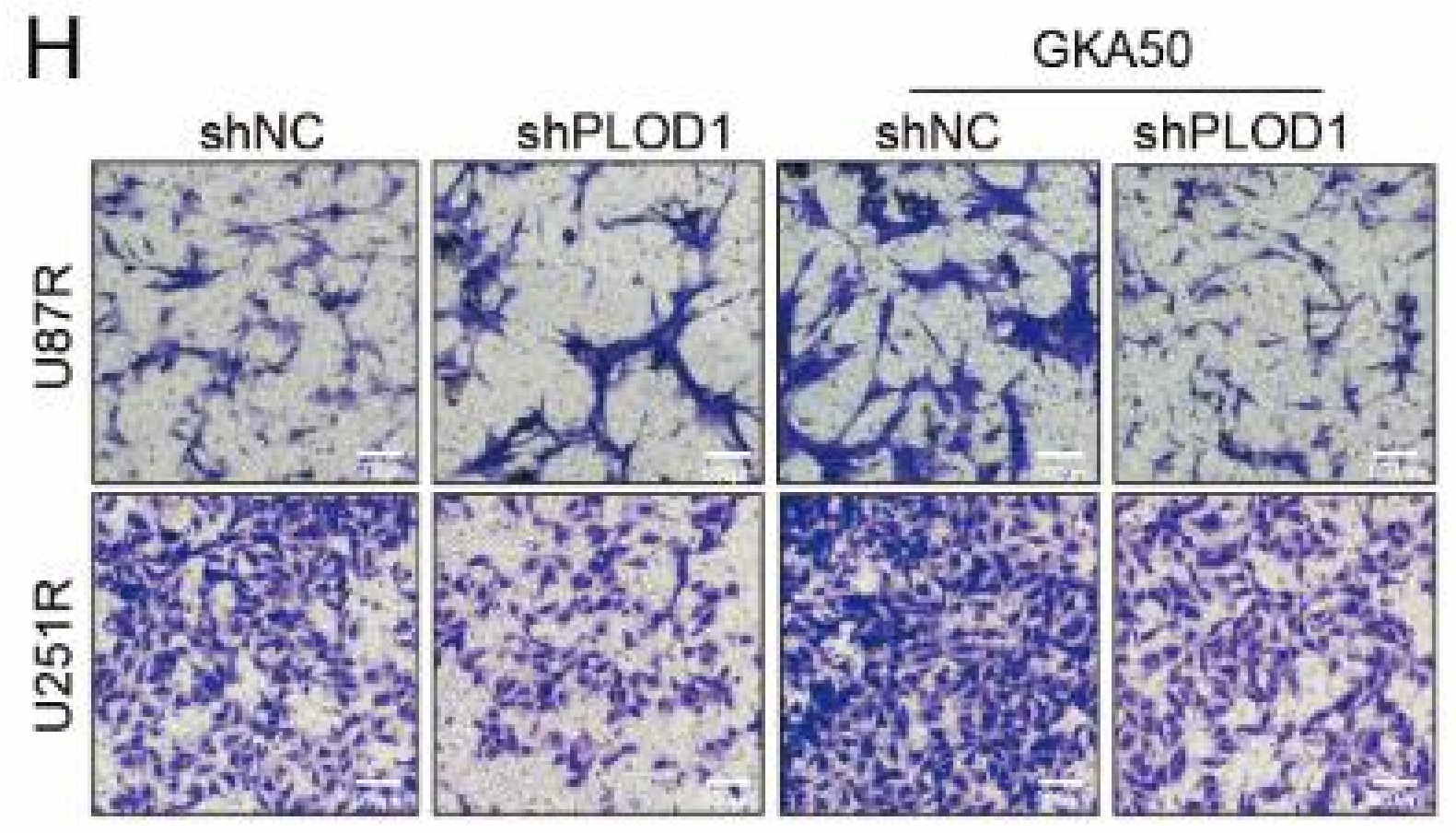

U87R

ShNC

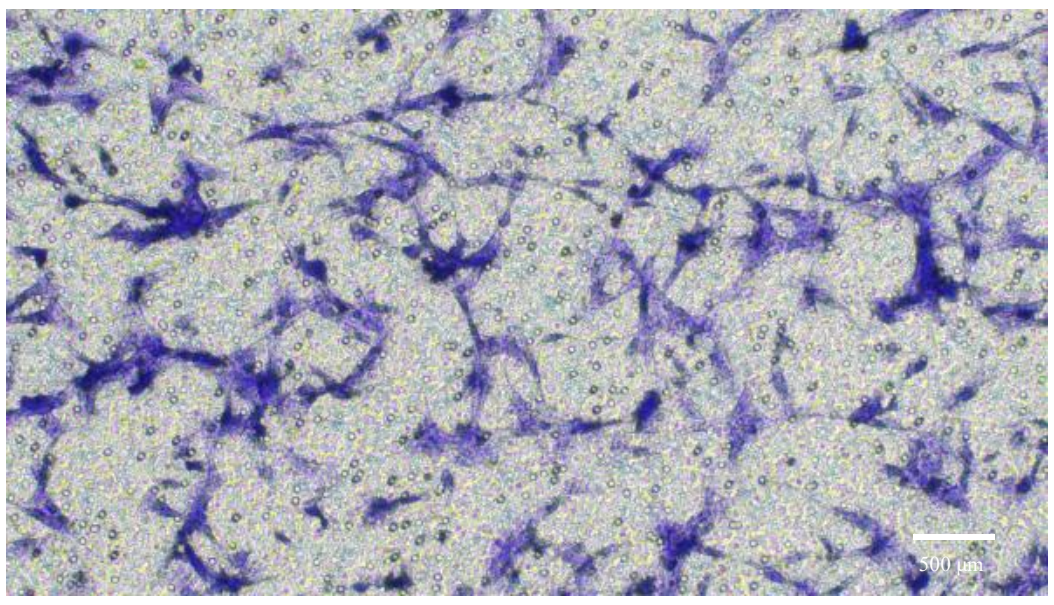

ShPLOD1

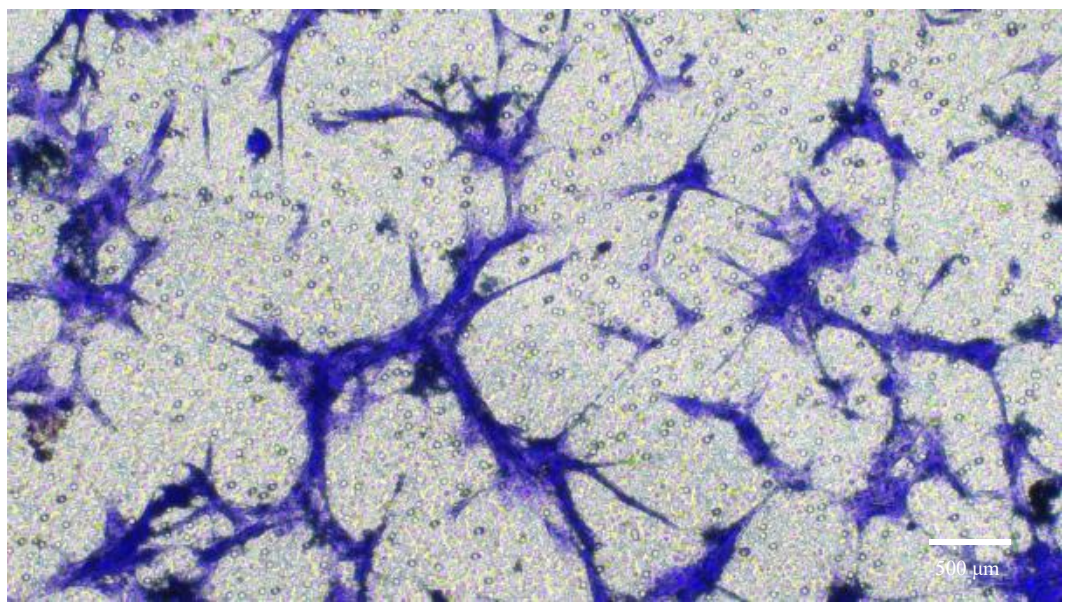

ShNC+GKA50

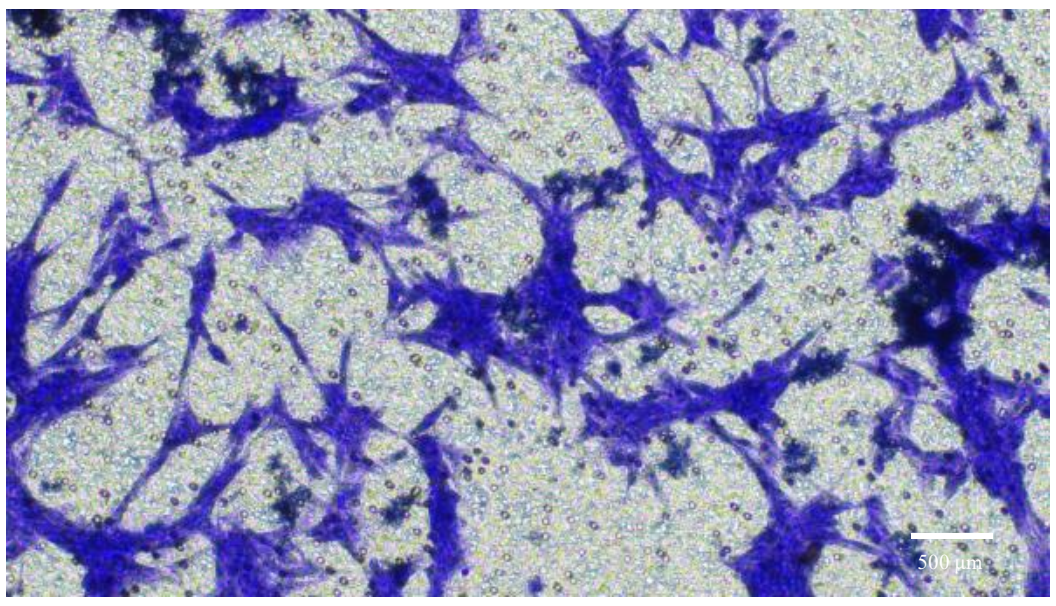

ShPLOD1+GKA50

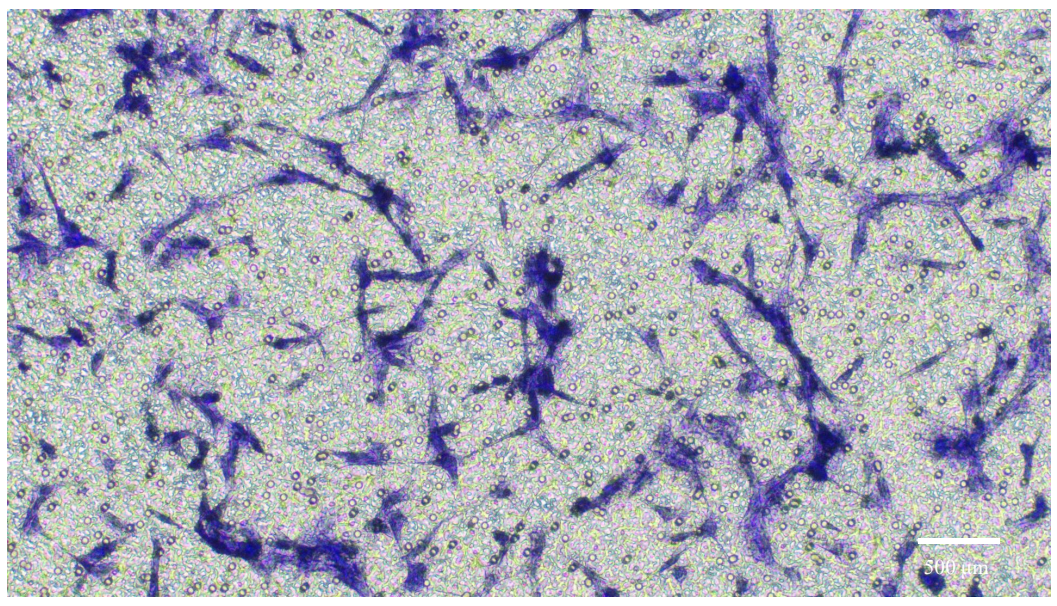

U251R

ShNC

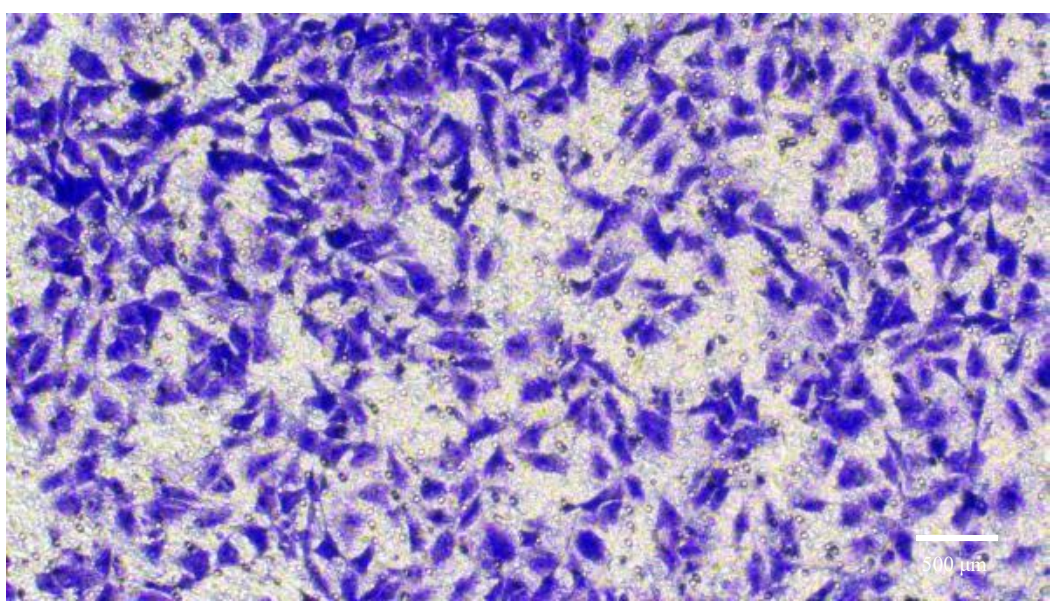

ShPLOD1

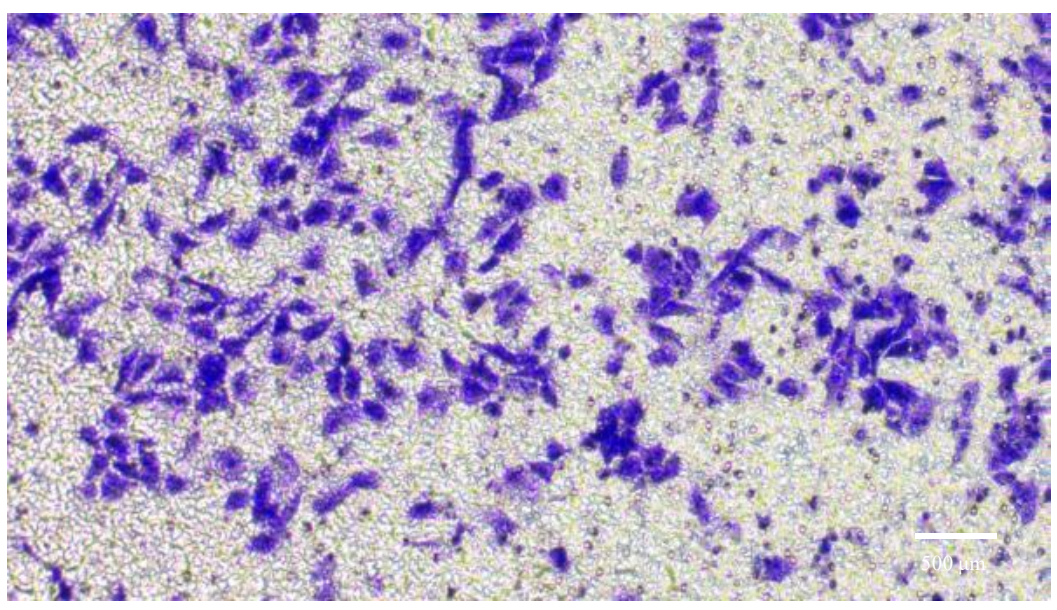

ShNC+GKA50

ShPLOD1+GKA50

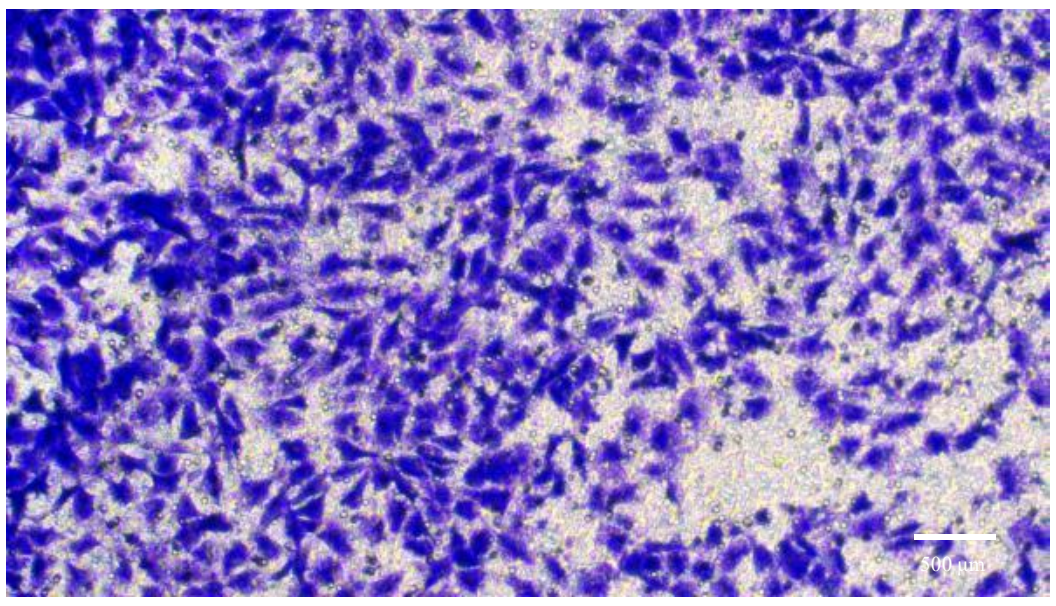

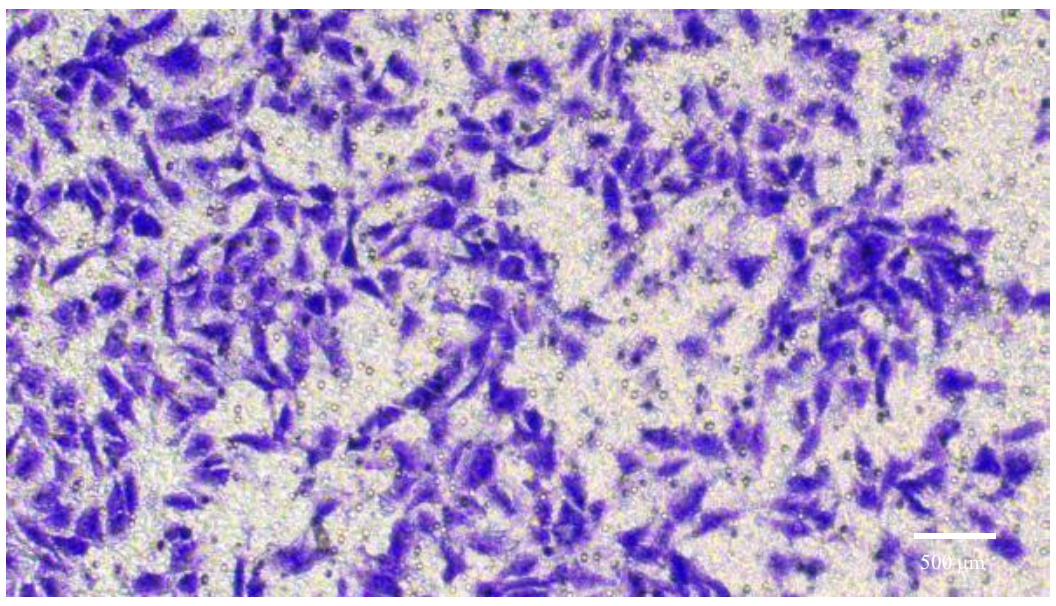

Figure3D

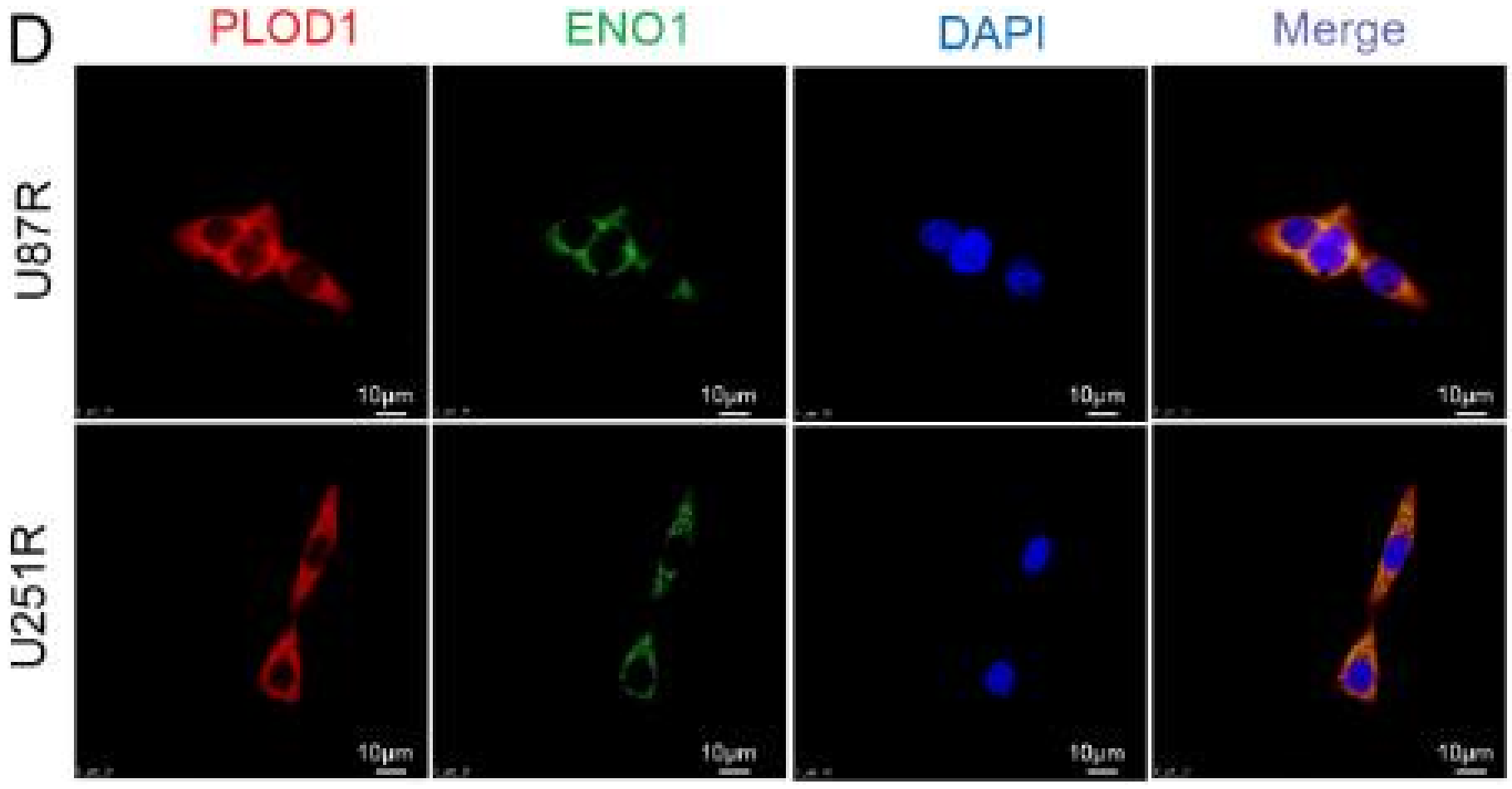

U87R

PLOD1

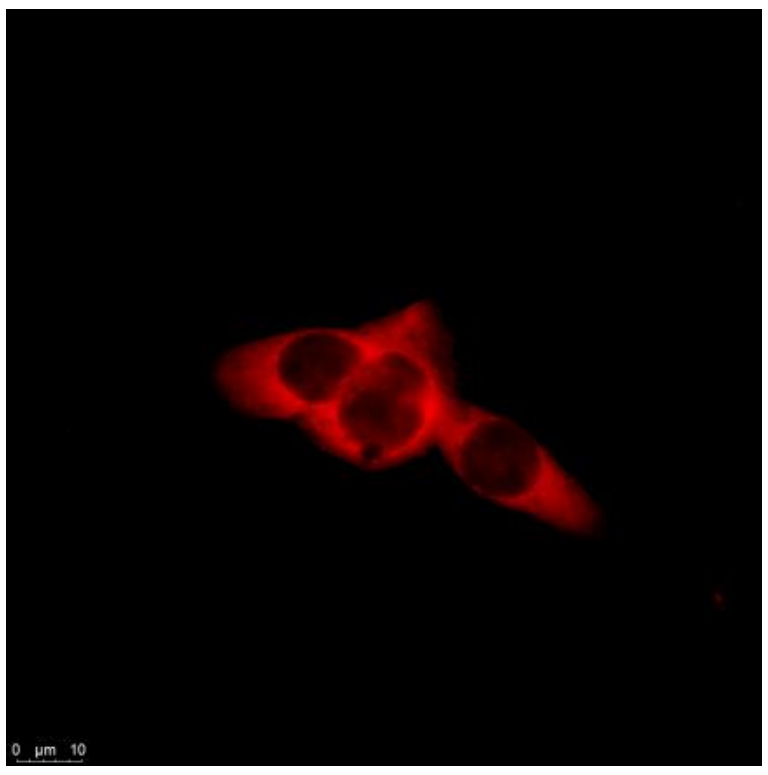

ENO1

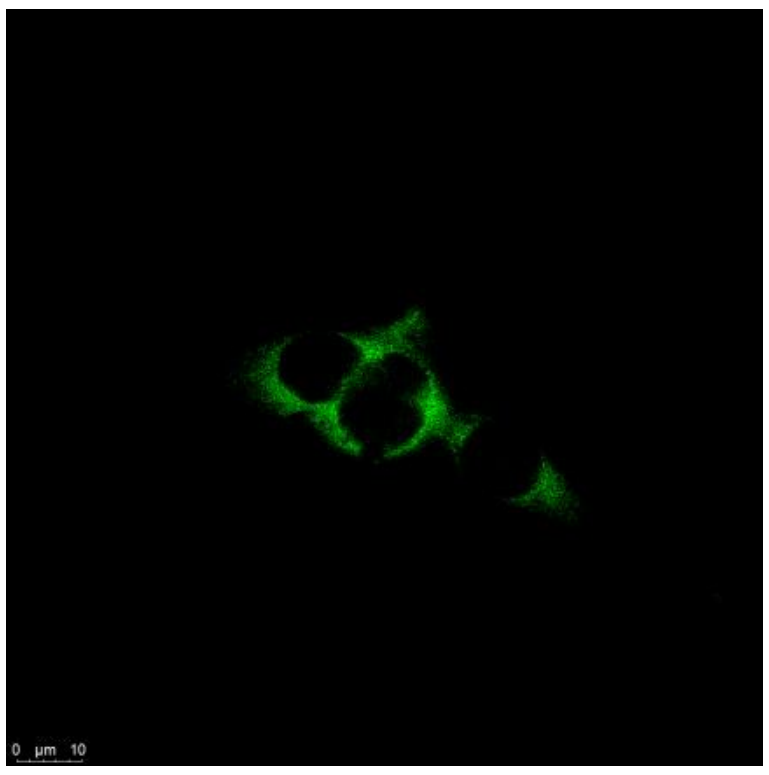

DAPI

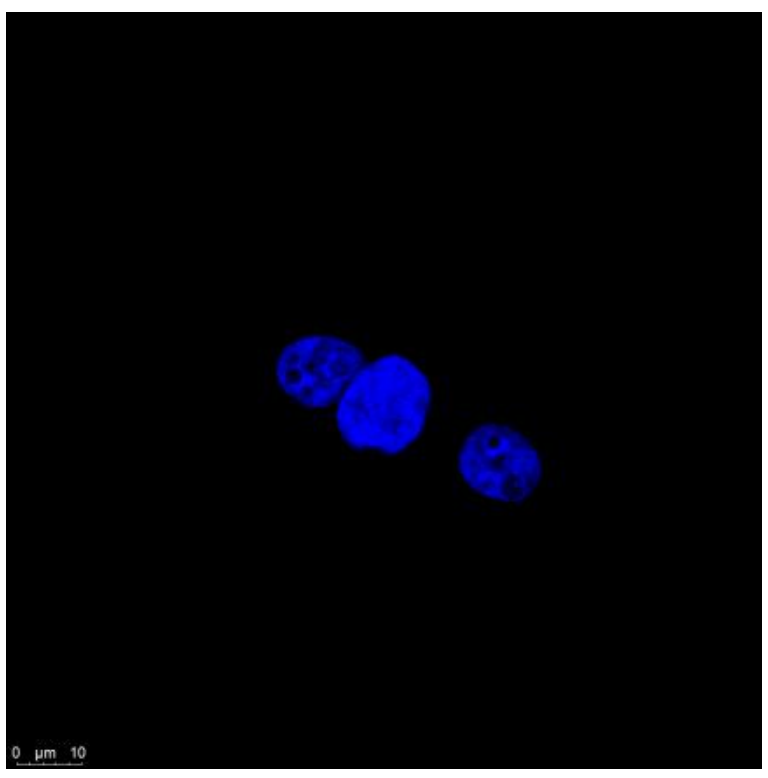

Merge

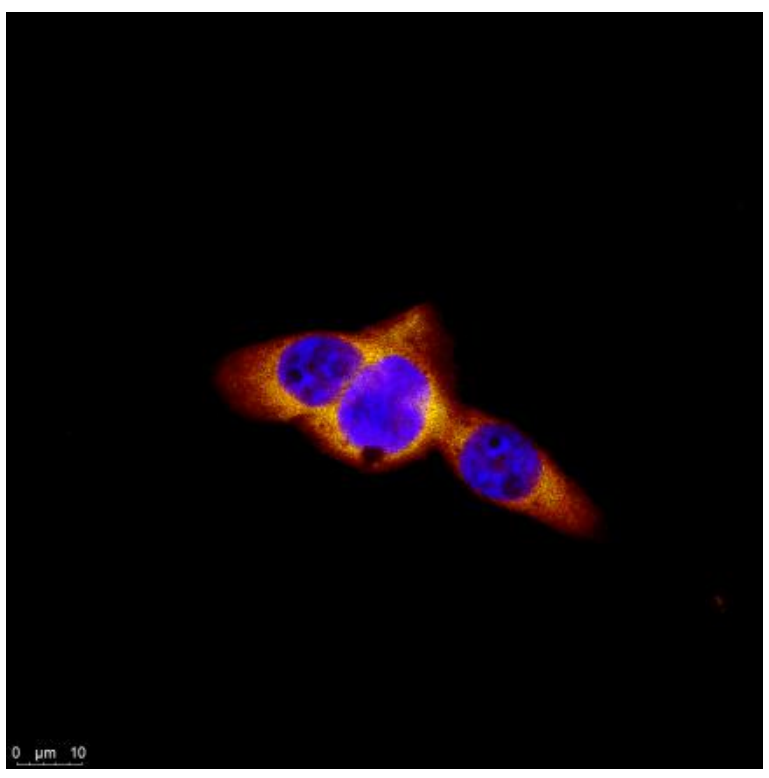

U251R

PLOD1 ENO1

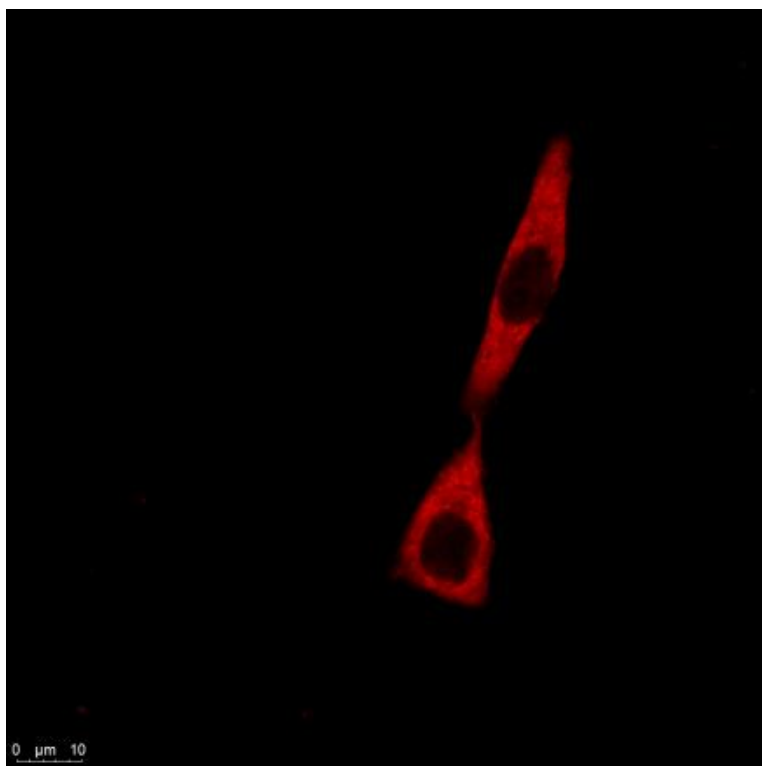

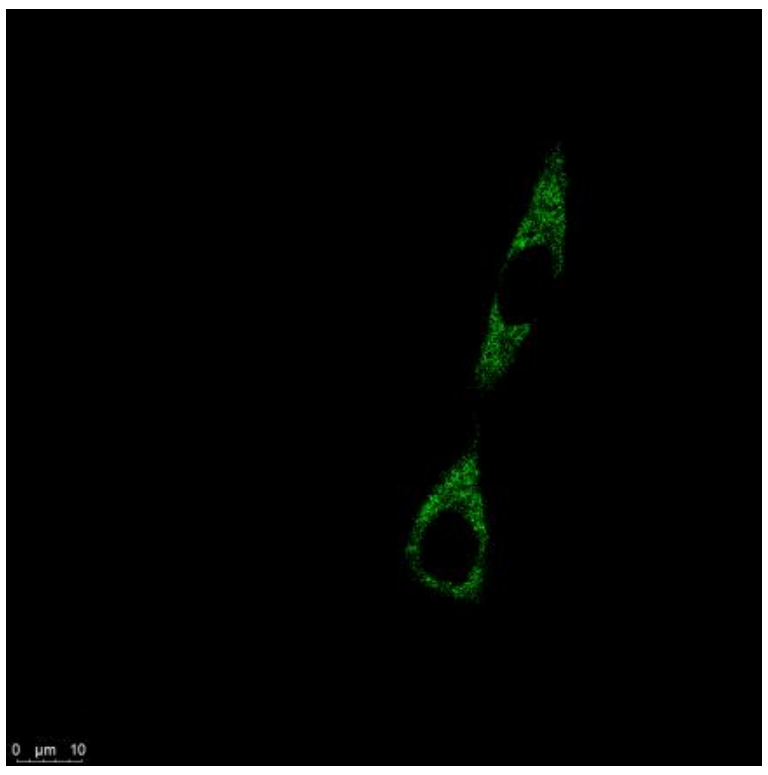

DAPI Merge

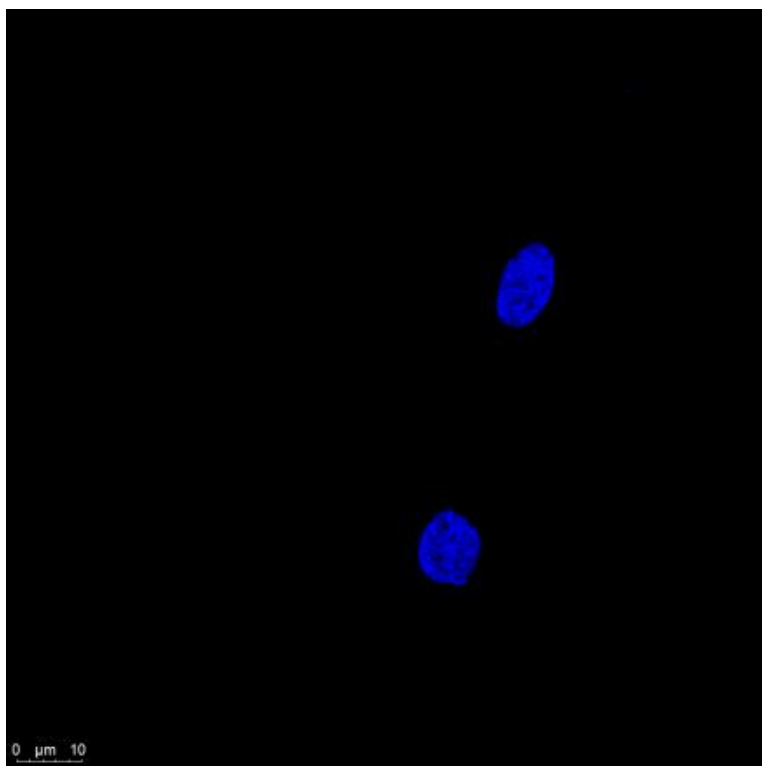

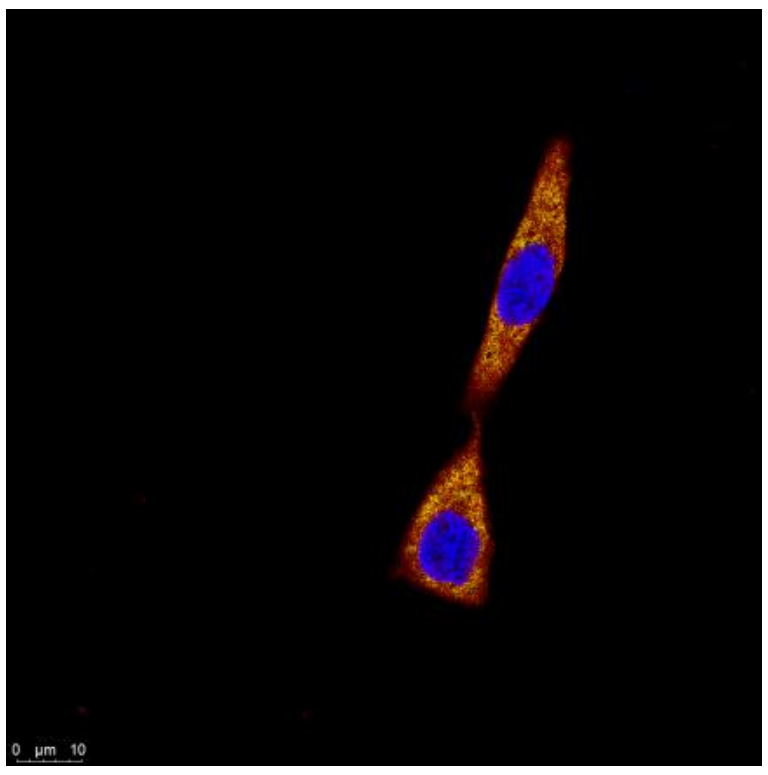

Figure3M

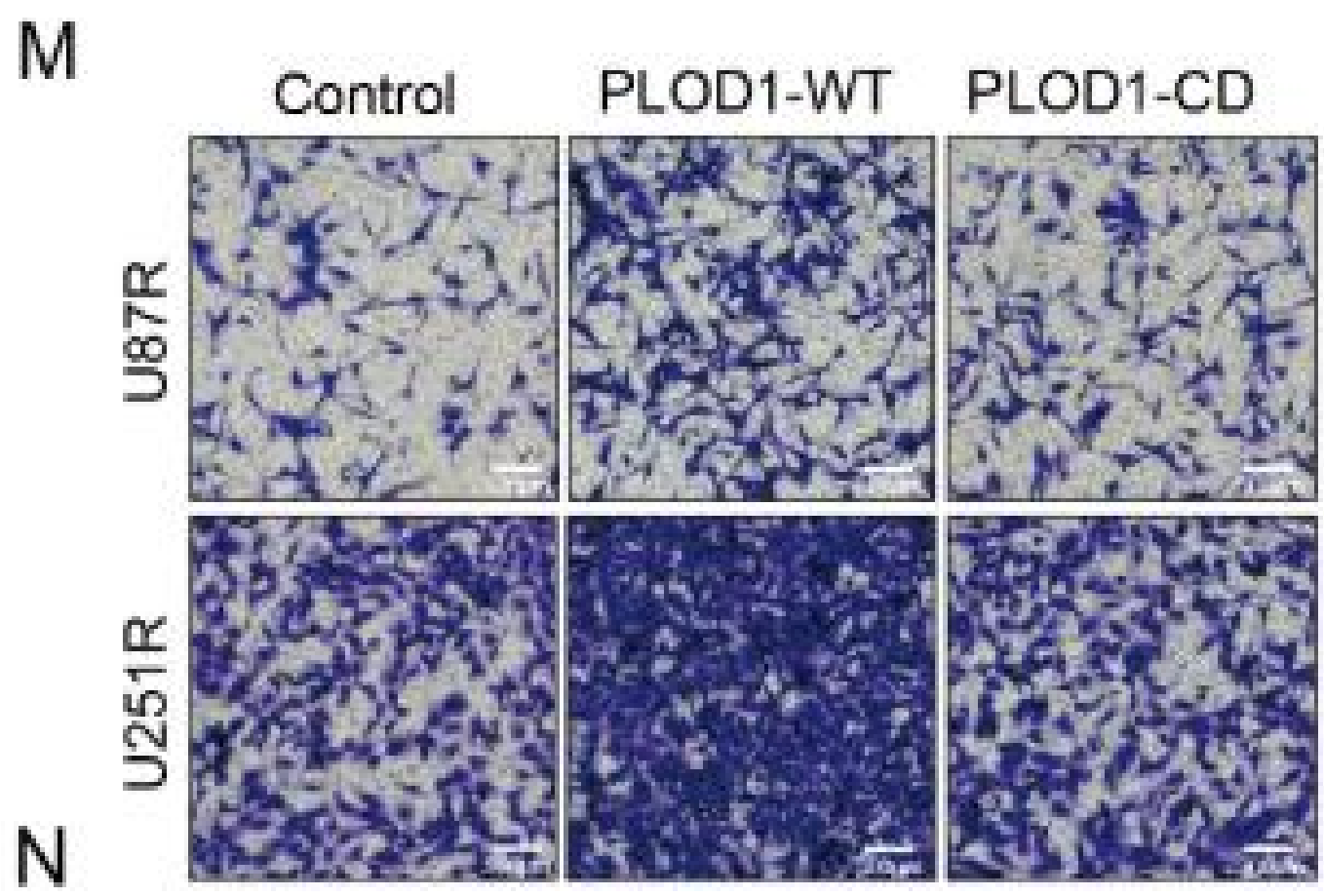

U87R

Control PLOD1-WT

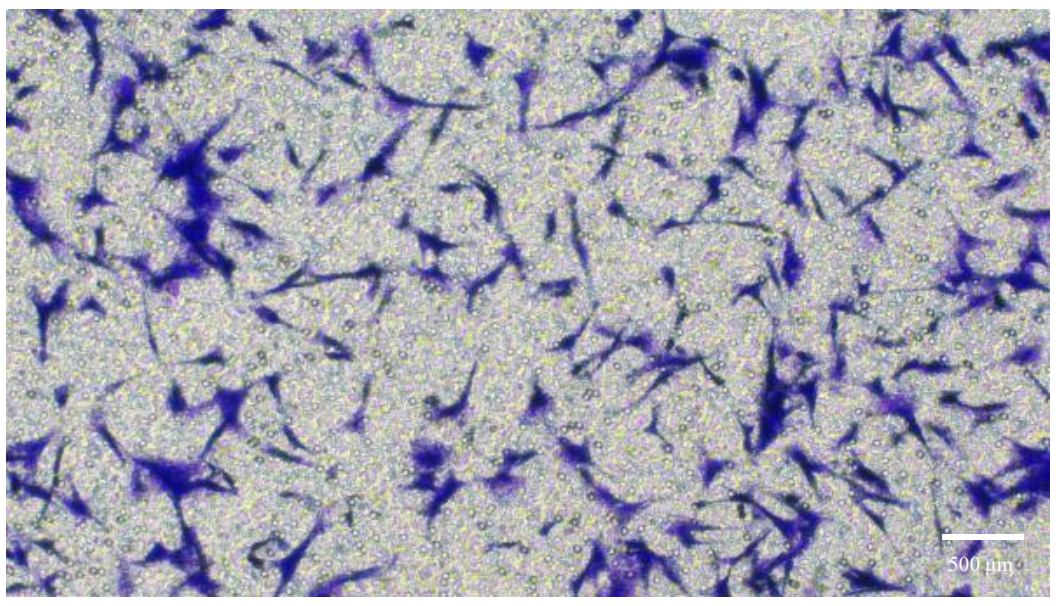

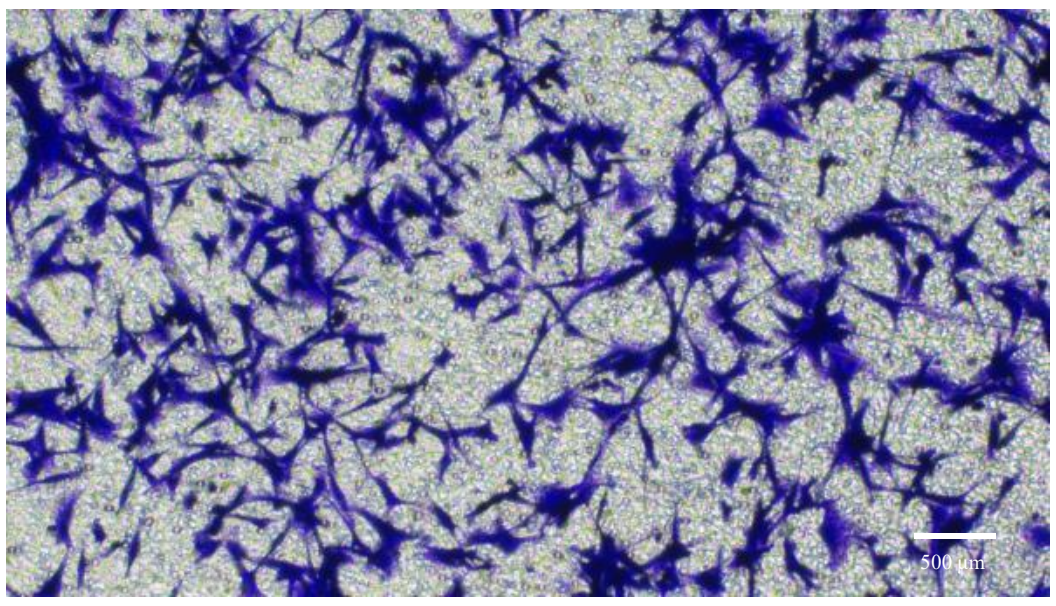

PLOD1-CD

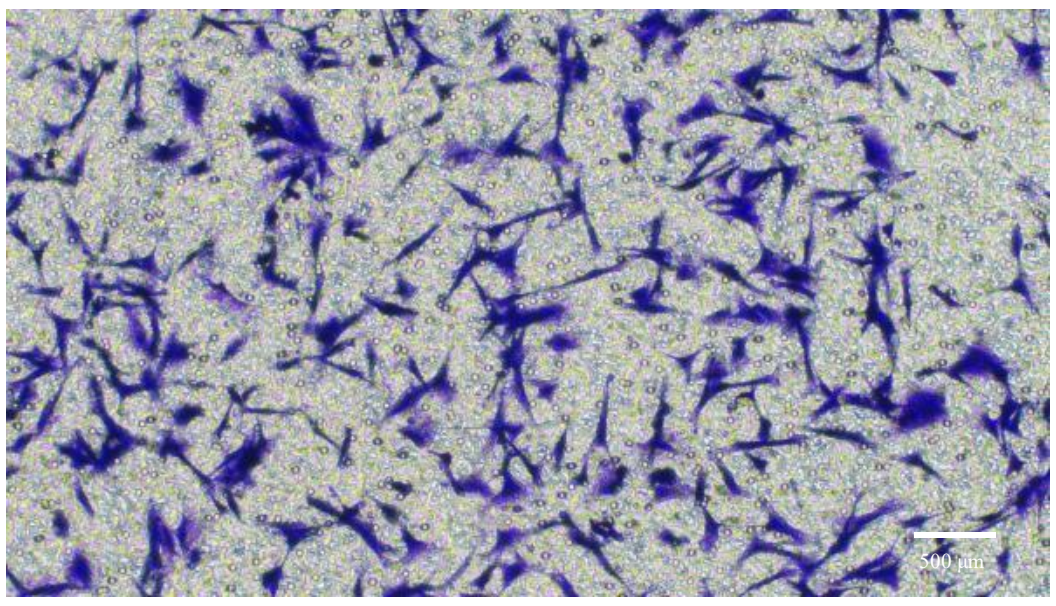

U251R

Control

PLOD1-WT

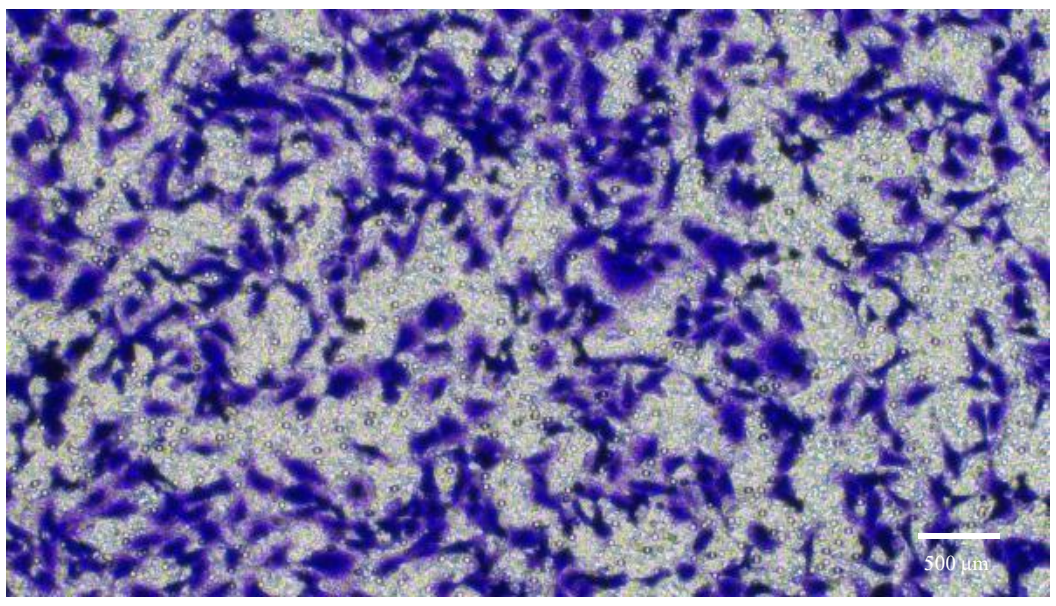

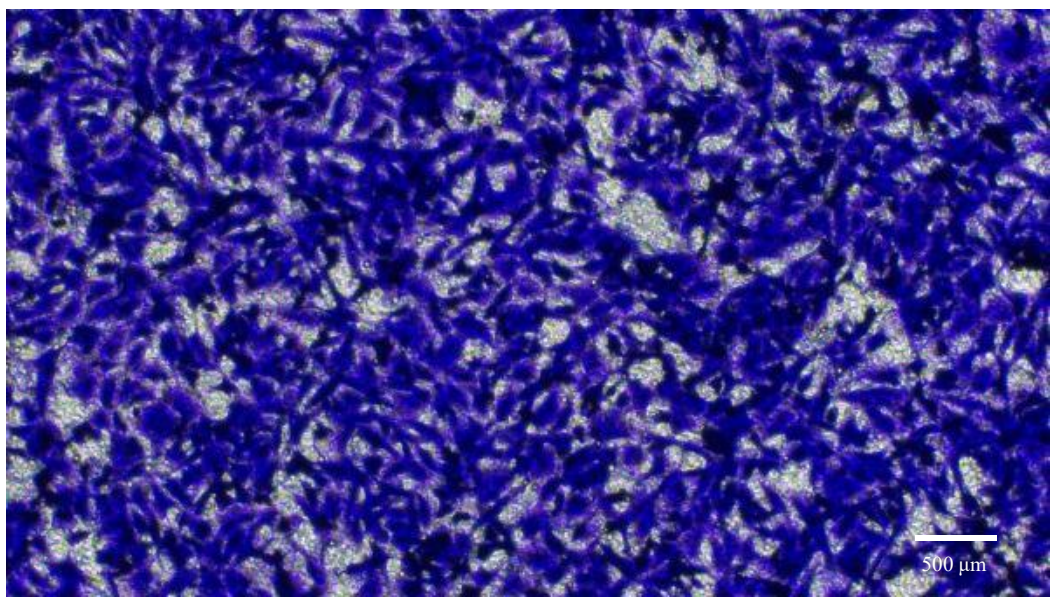

PLOD1-CD

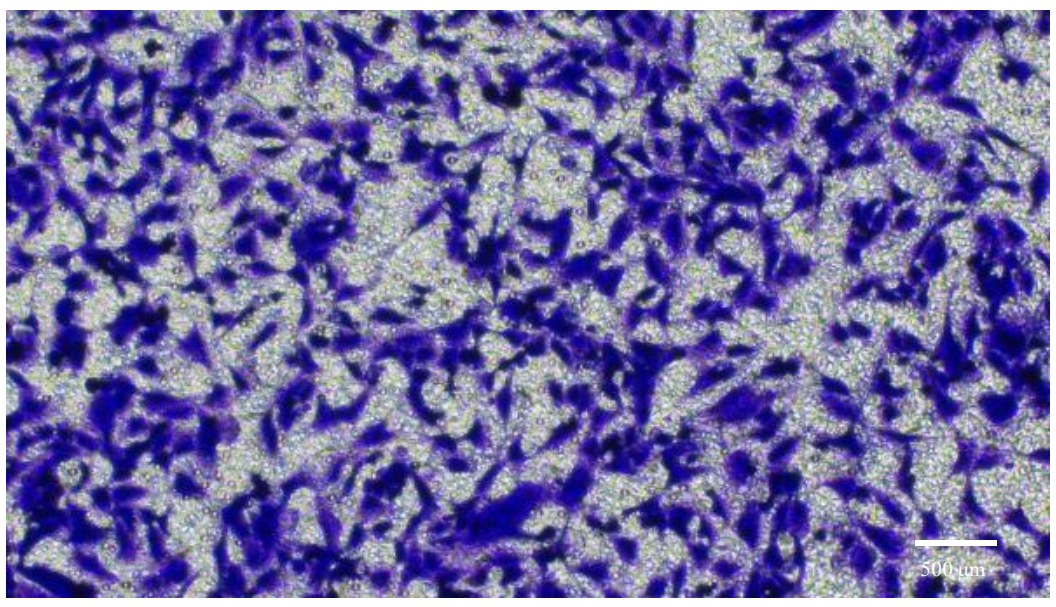

Figure 7E

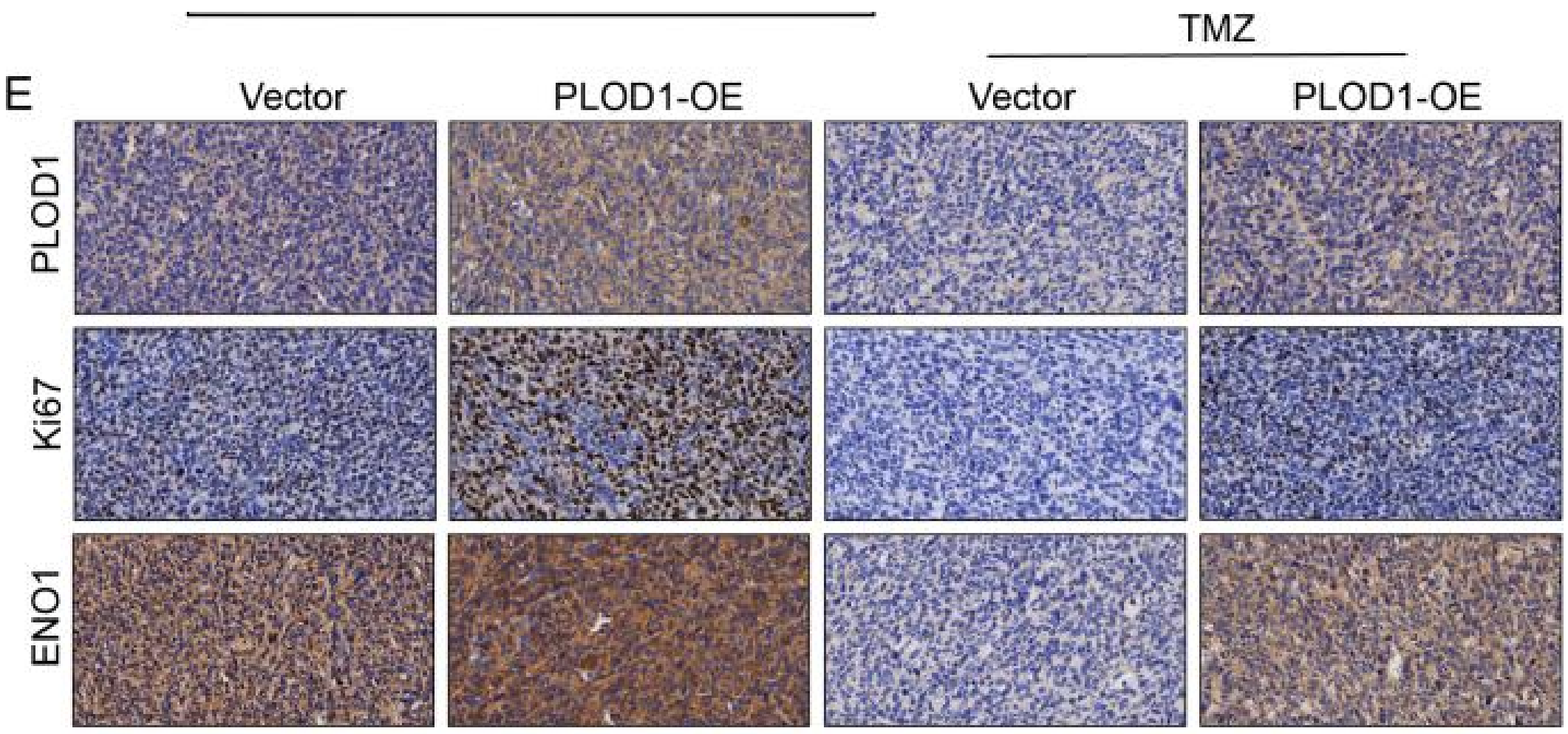

PLOD1

Vector

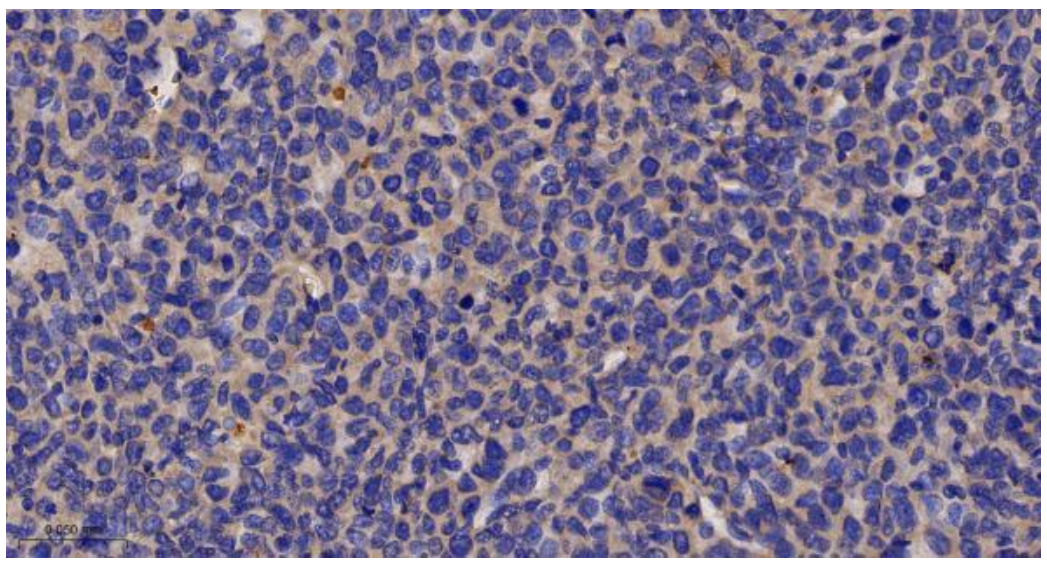

PLOD1-OE

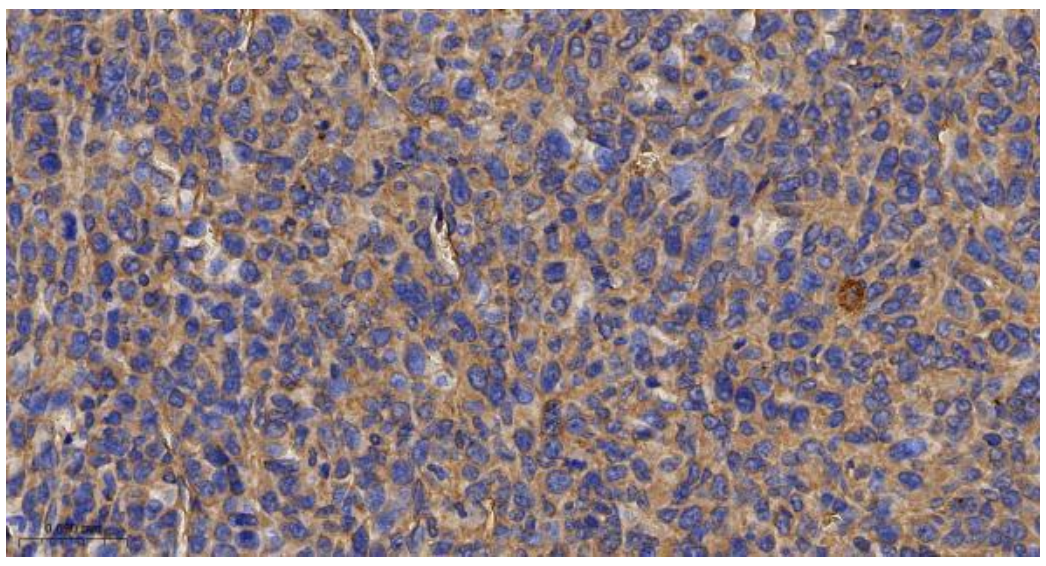

Vector-TMZ

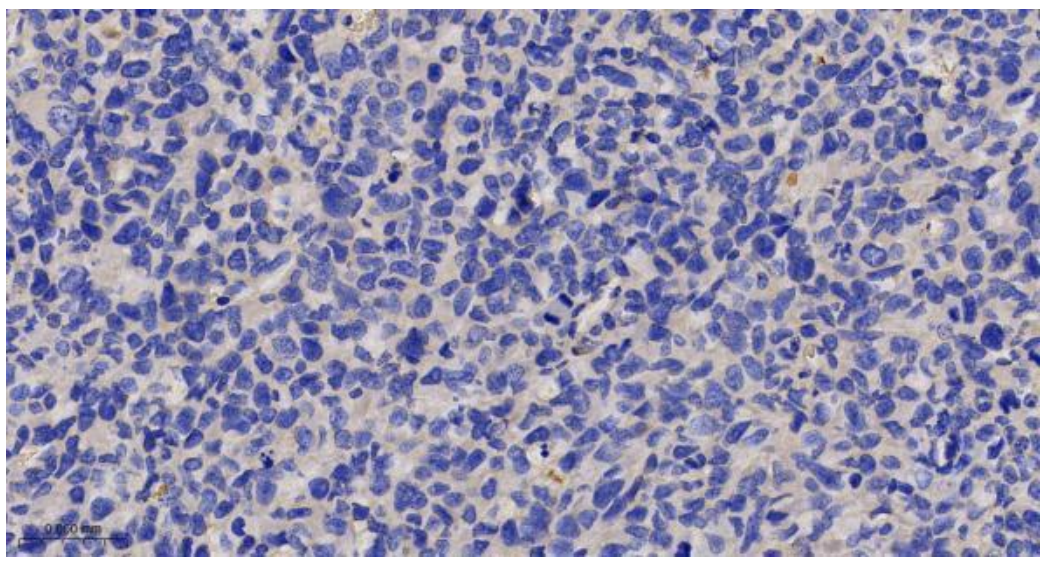

PLOD1-OE-TMZ

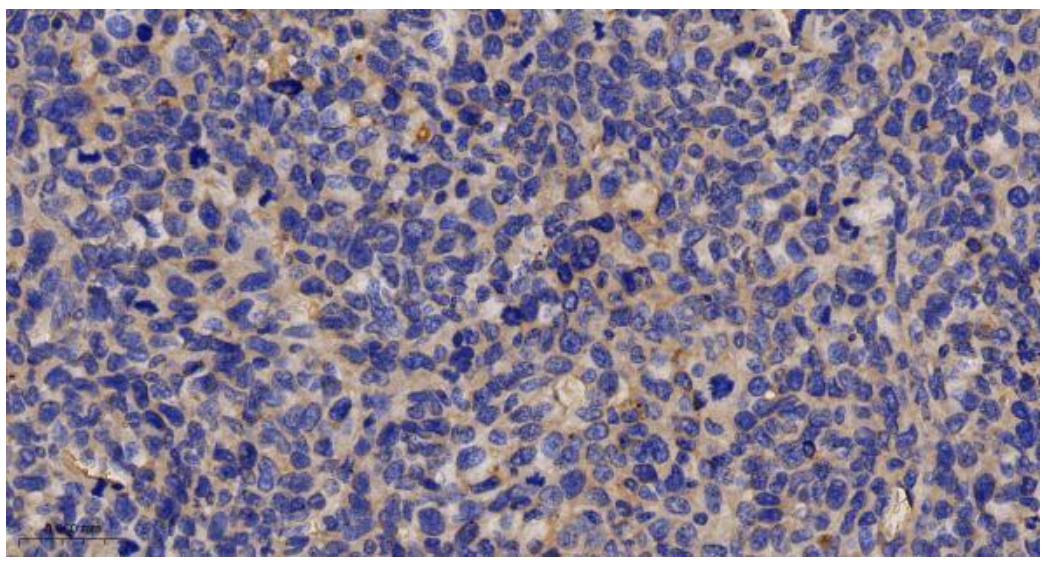

Ki67

Vector

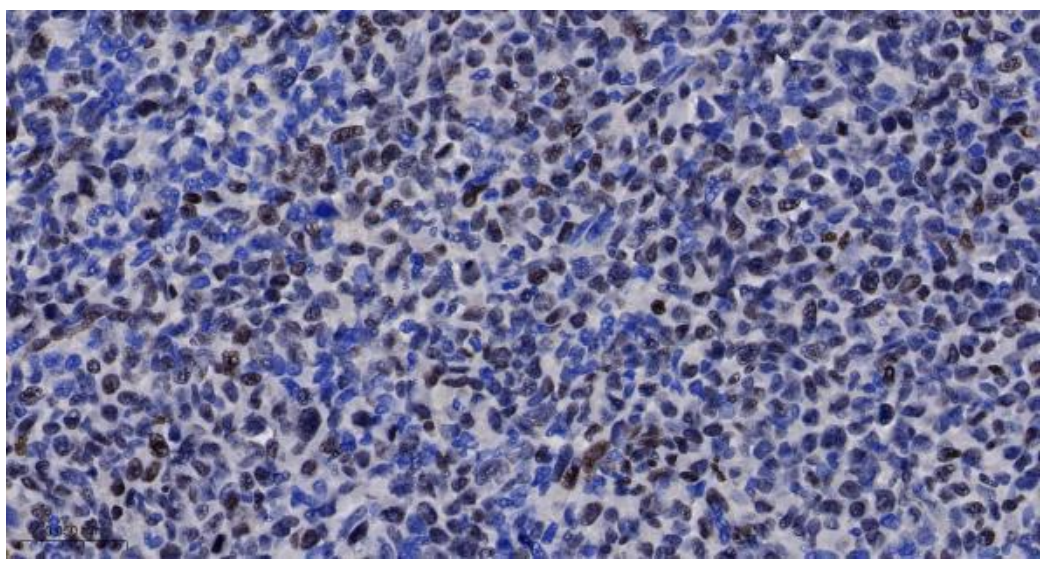

PLOD1-OE

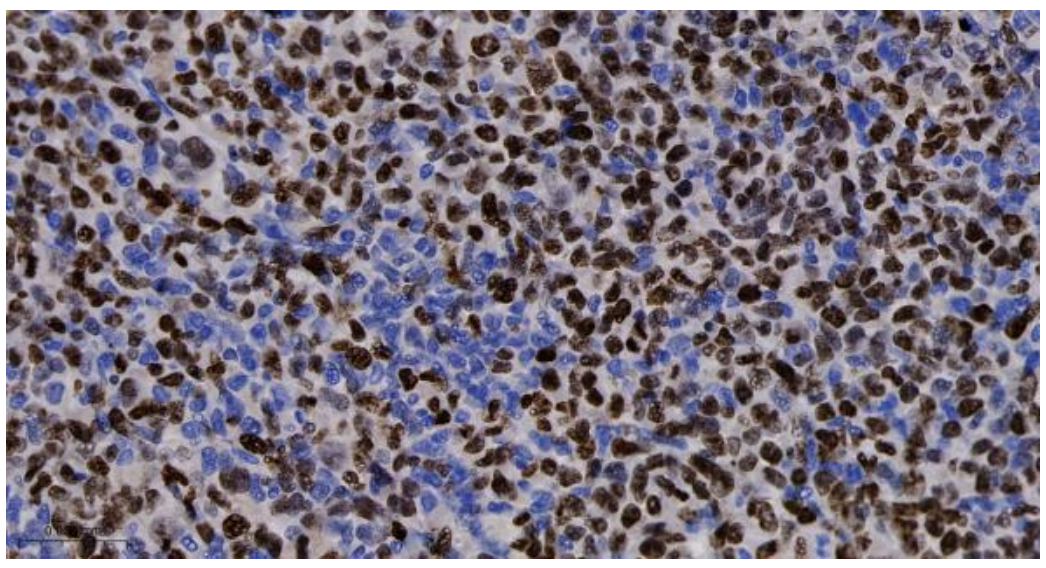

Vector-TMZ

PLOD1-OE-TMZ

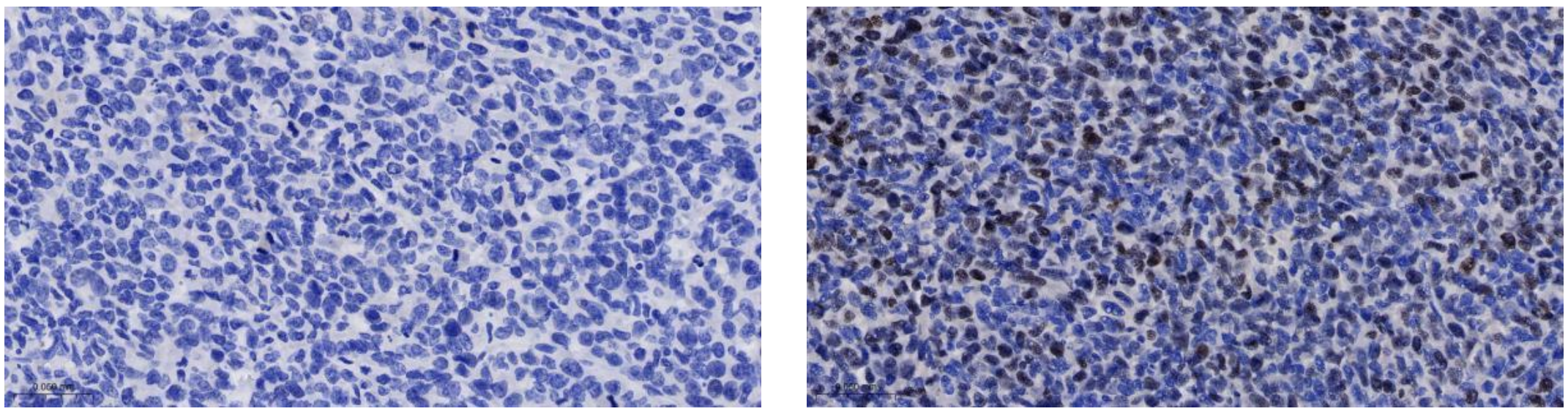

ENO1

Vector PLOD1-OE

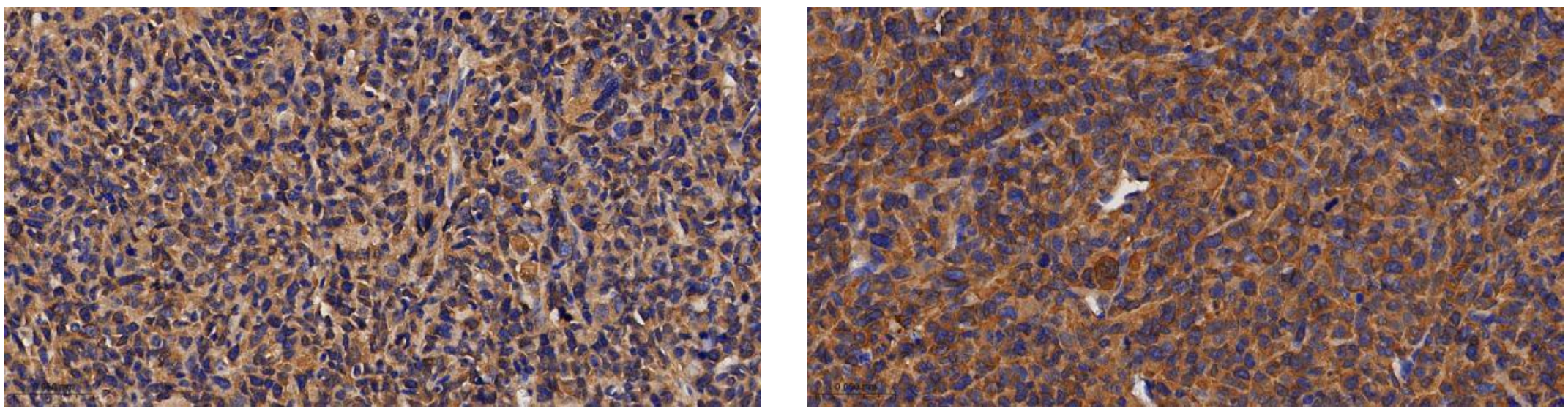

Vector-TMZ PLOD1-OE-TMZ

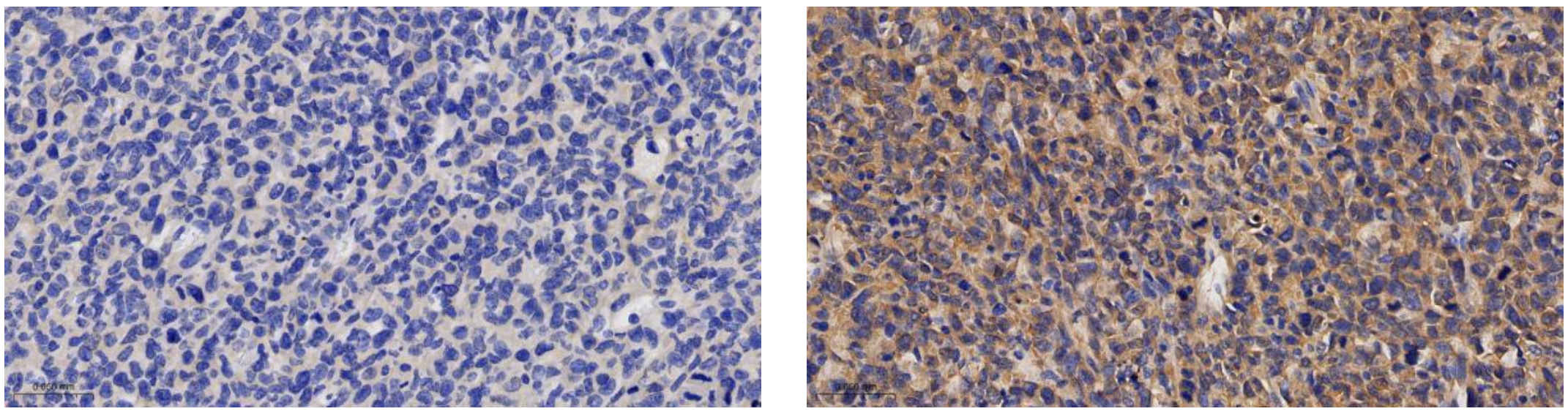

Figure 7L

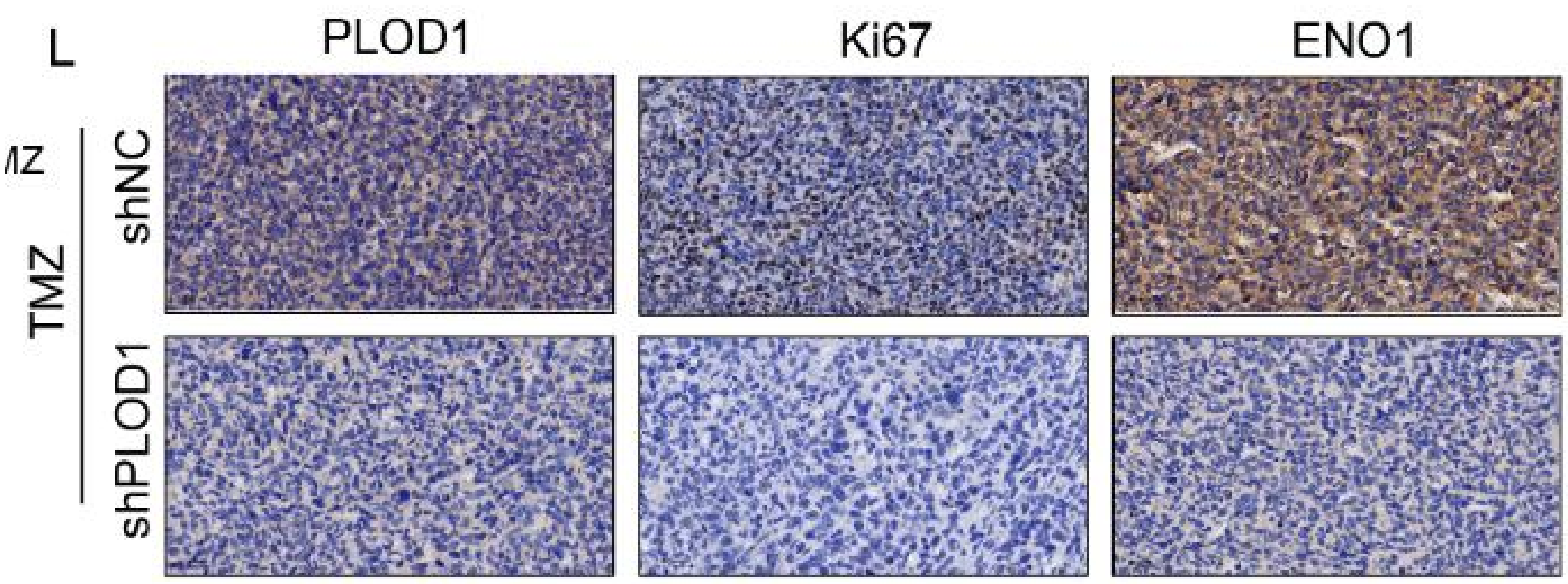

TMZ

ShNC

PLOD1 Ki67

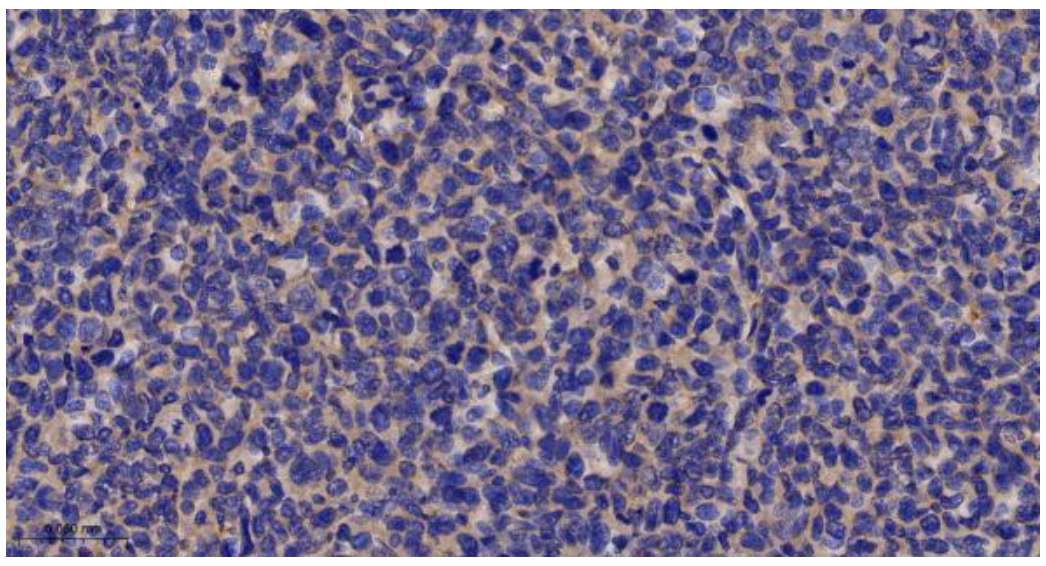

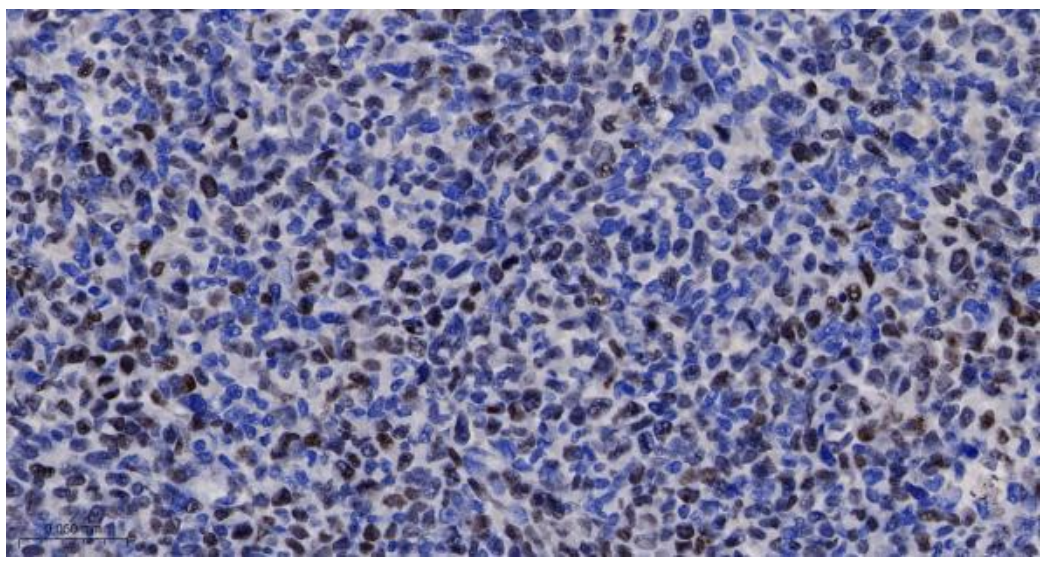

ENO1

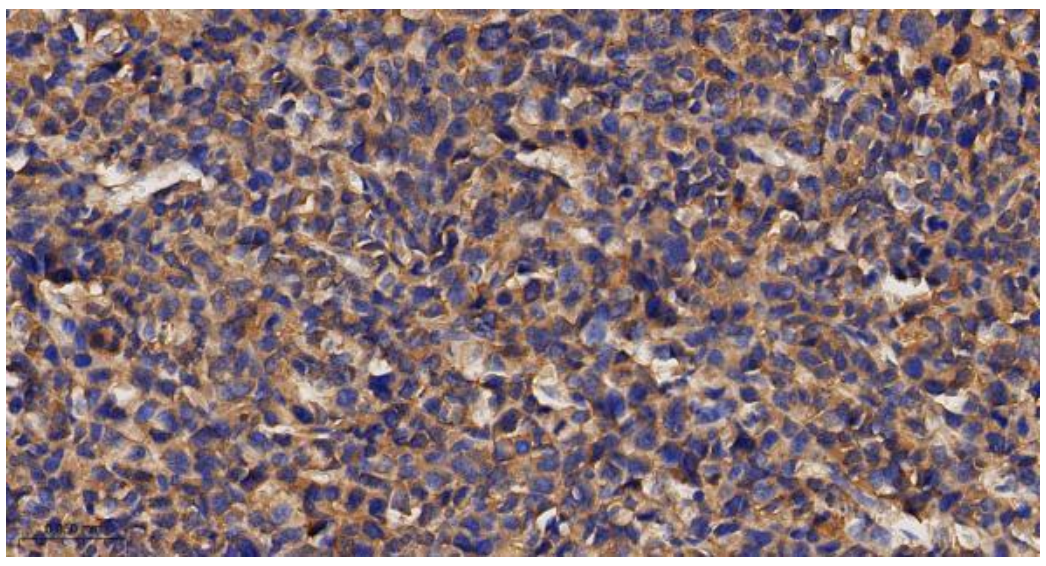

ShPLOD1

PLOD1

Ki67

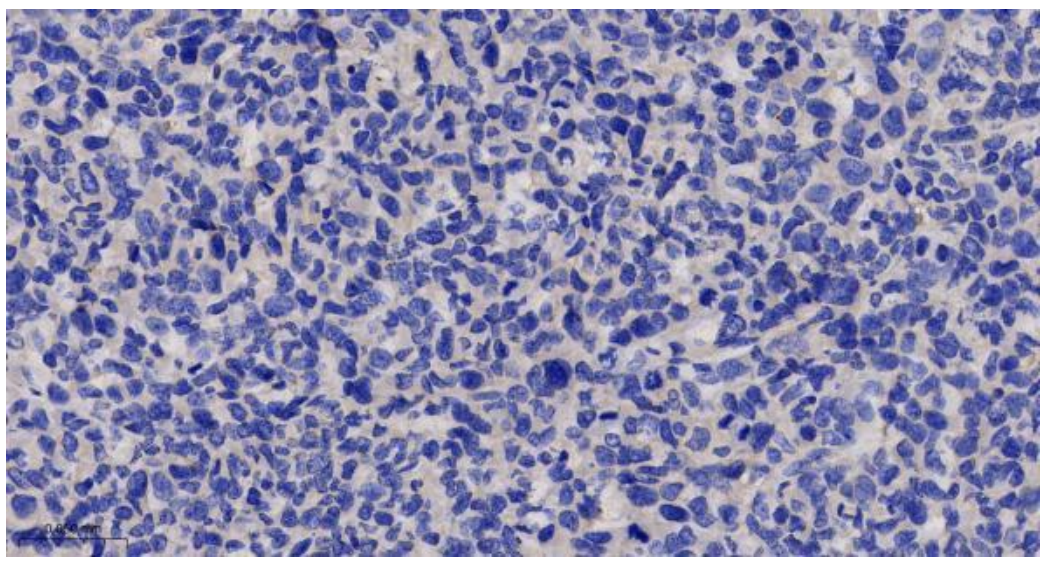

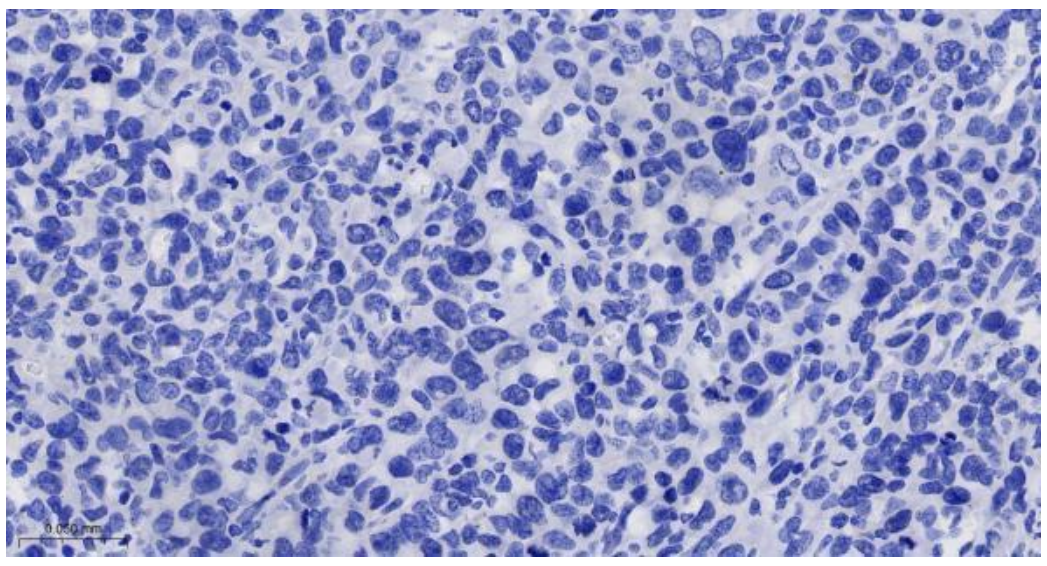

ENO1

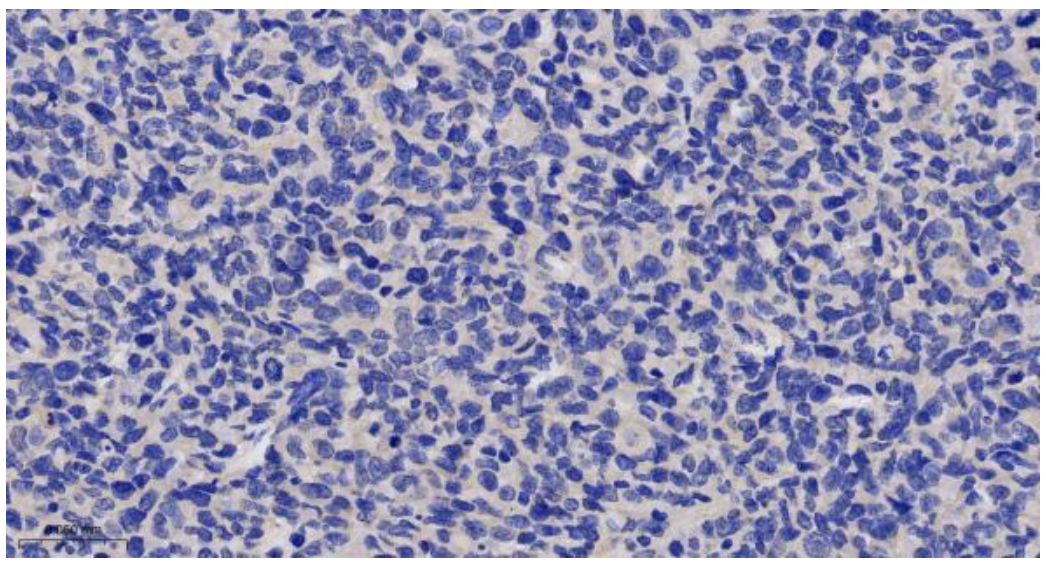

Figure 7Q

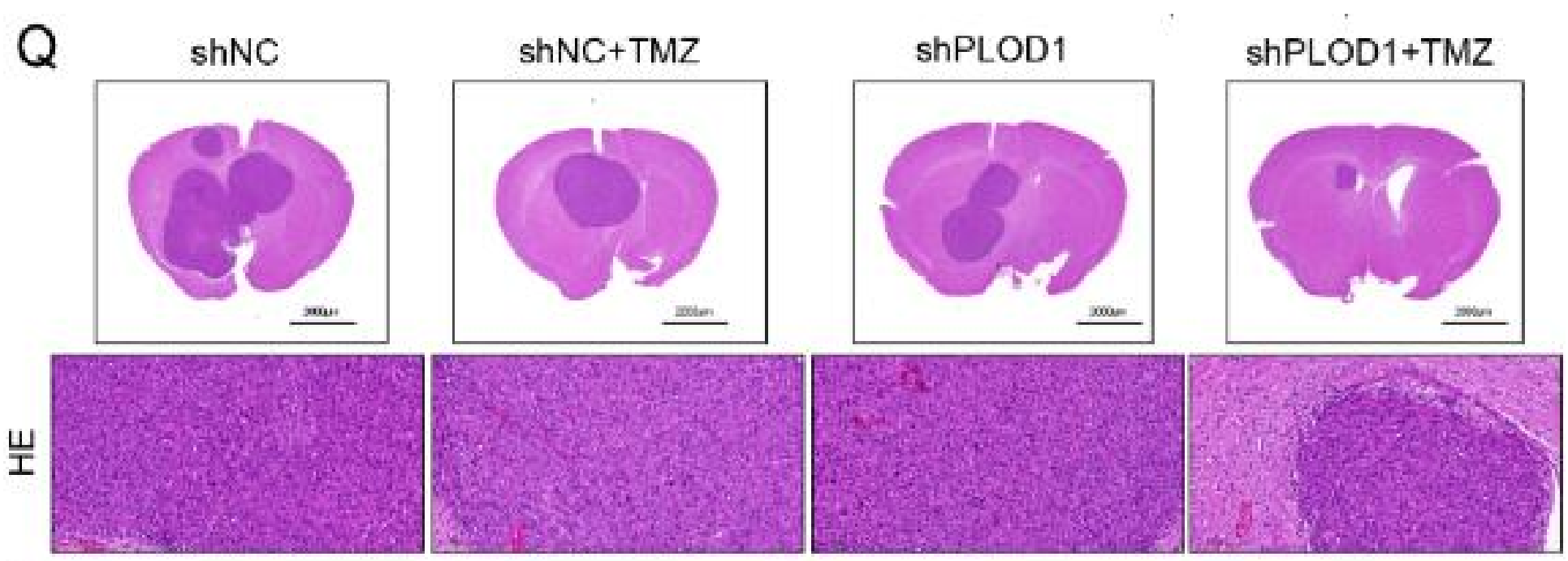

ShNC

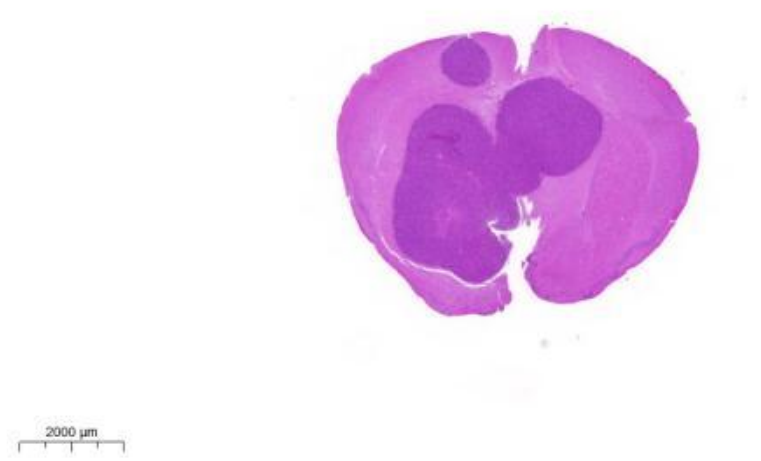

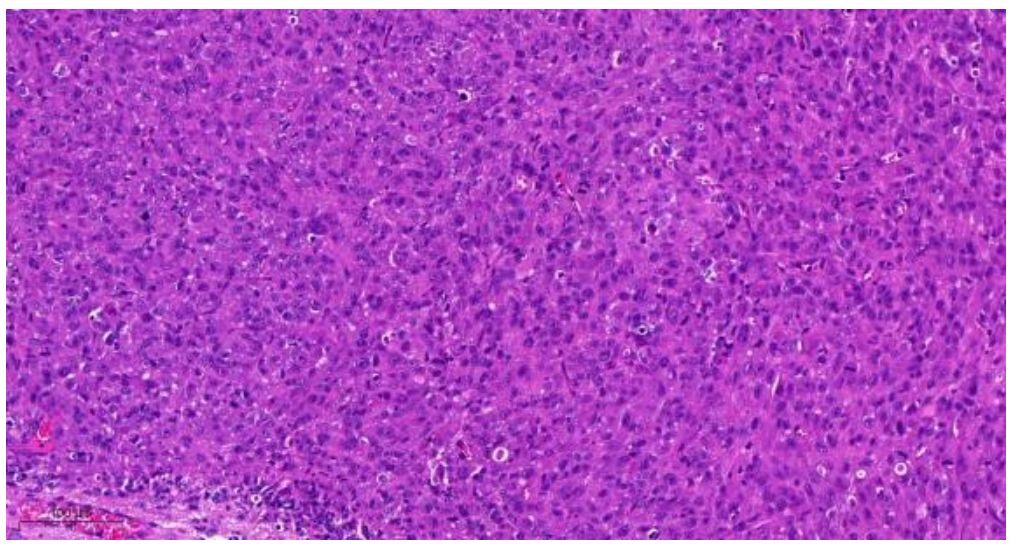

shNC+TMZ

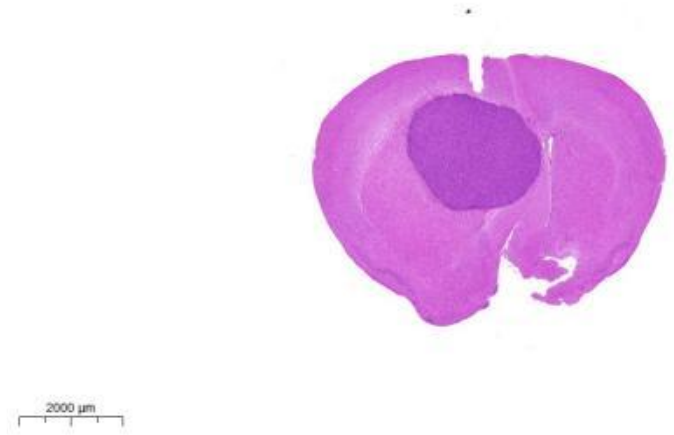

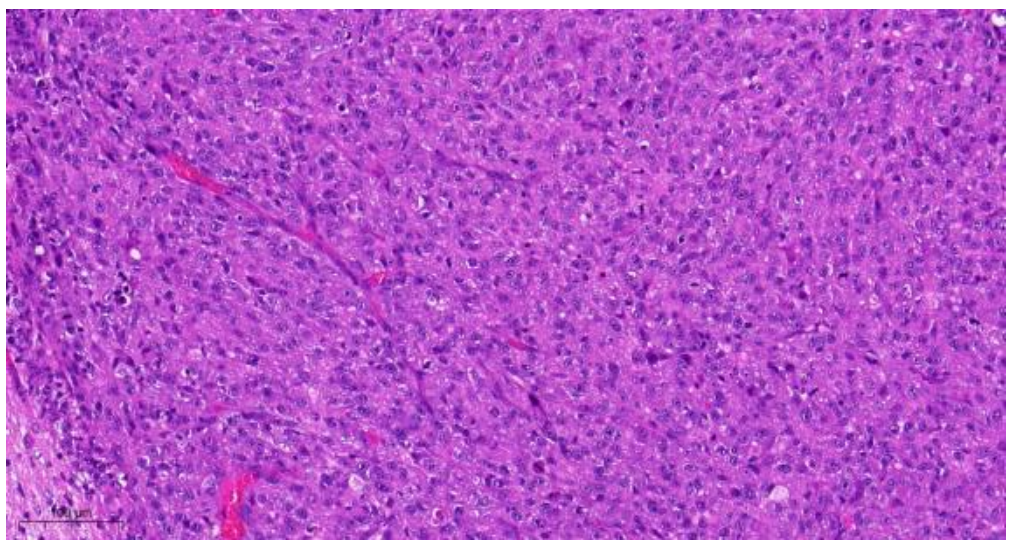

shPLOD1

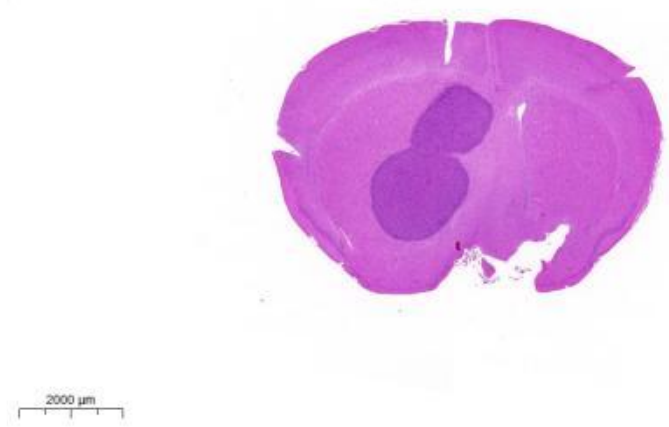

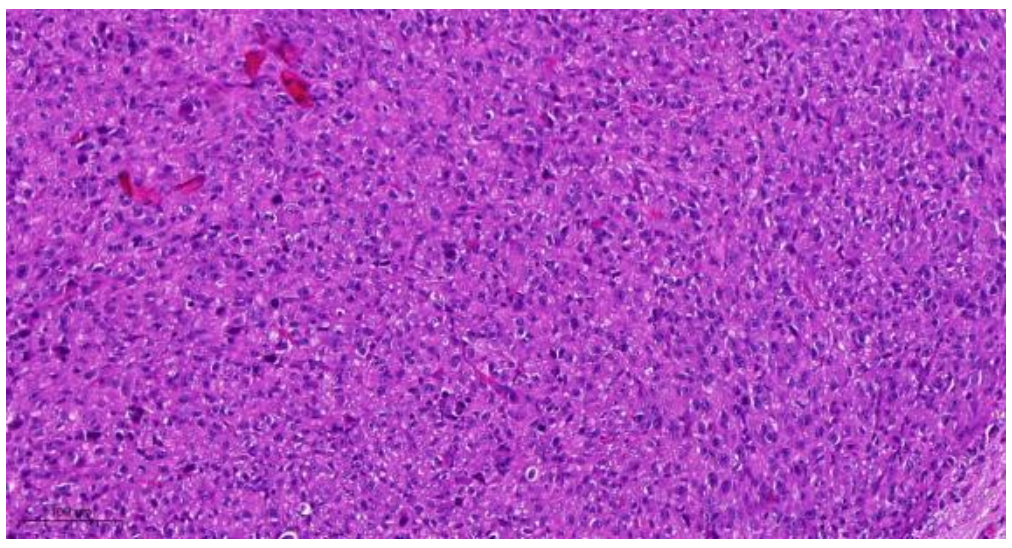

shPLOD1+TMZ

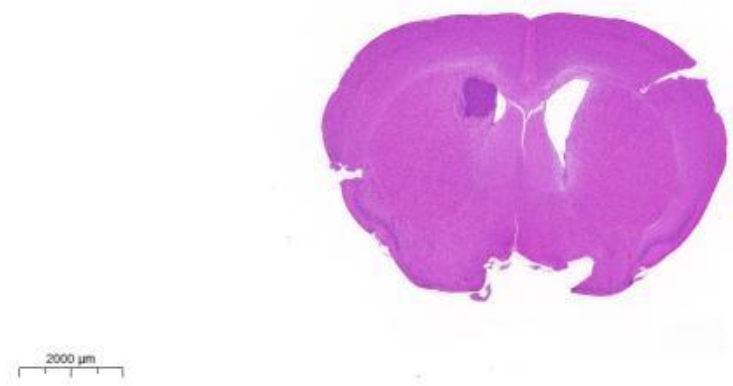

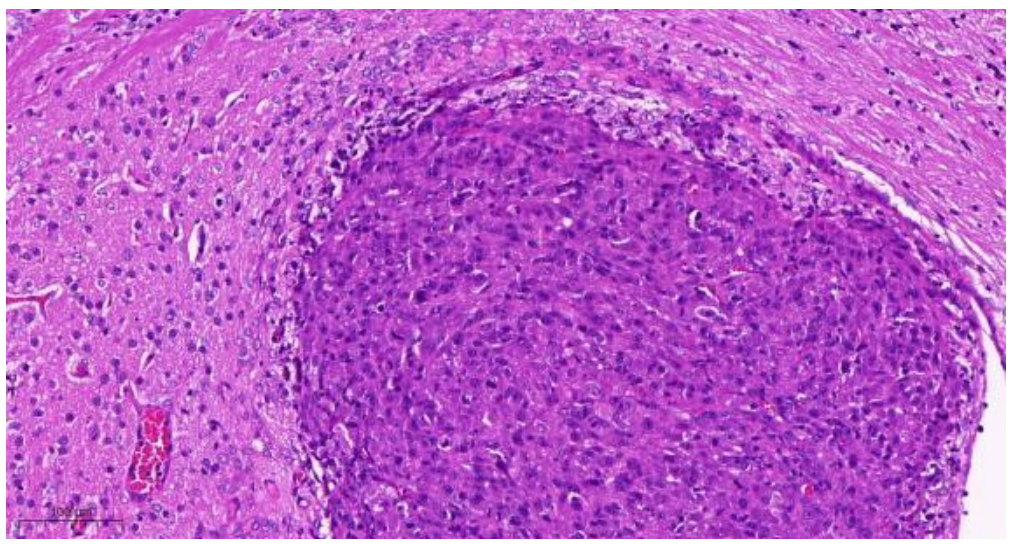

Figure 7R

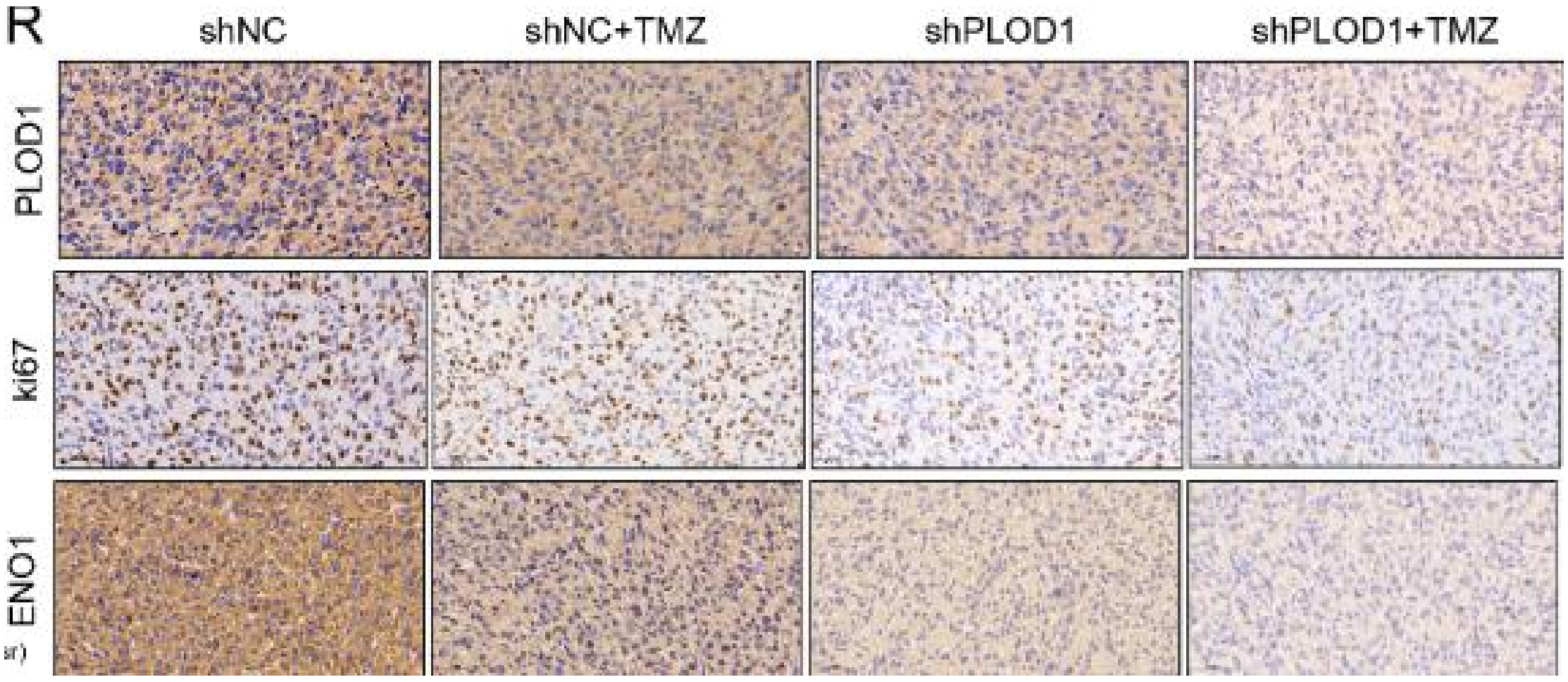

PLOD1

ShNC shNC+TMZ

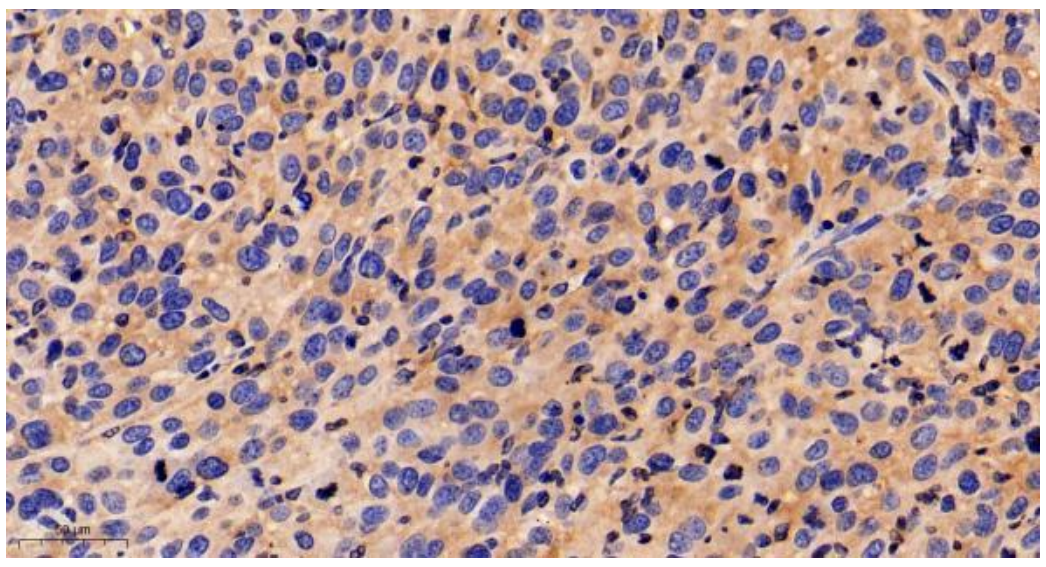

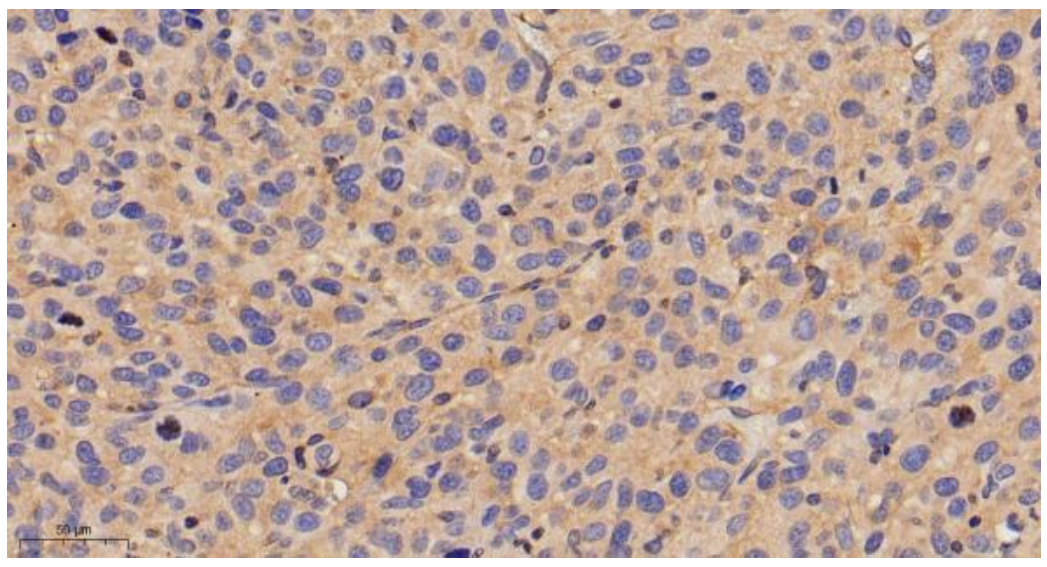

ShPLOD1 shPLOD1+TMZ

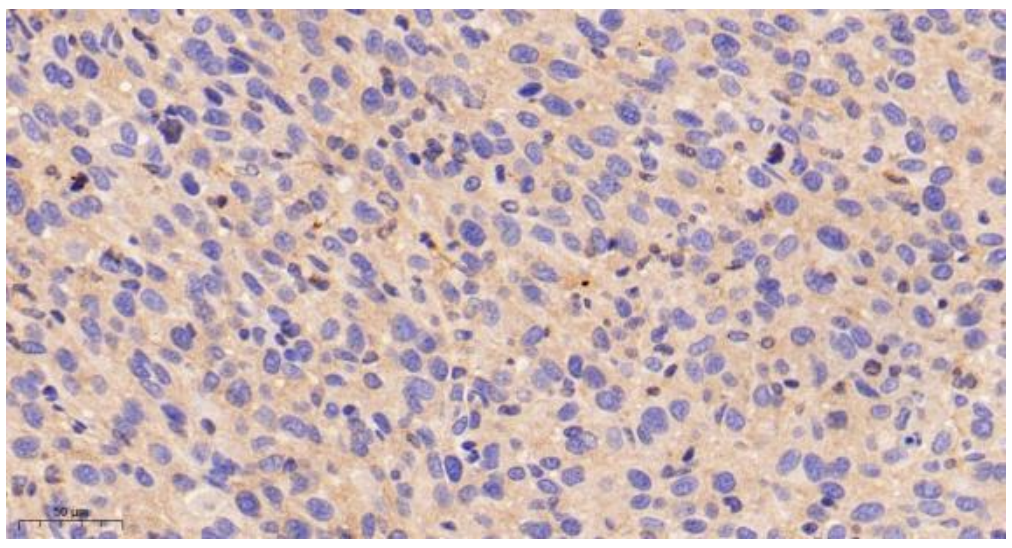

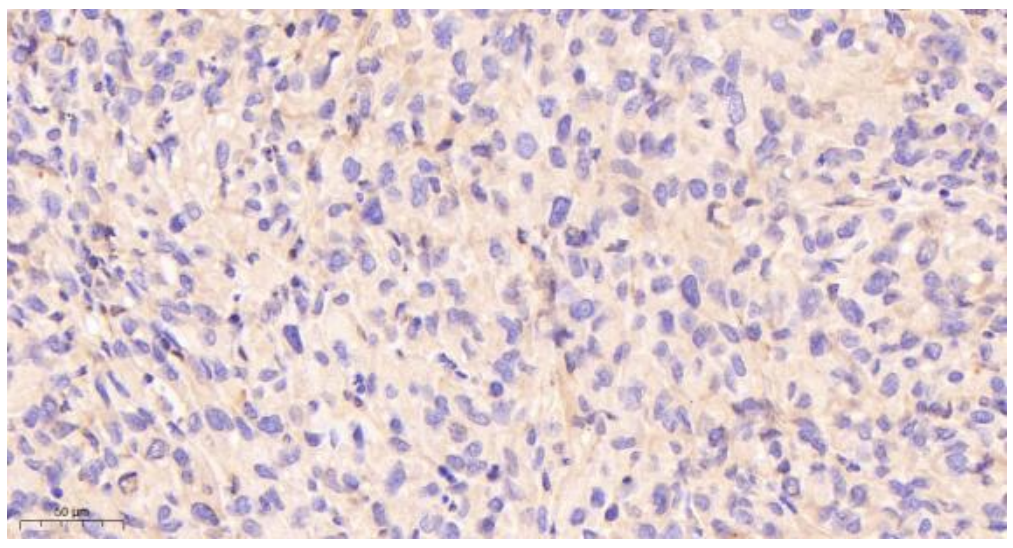

Ki67

ShNC shNC+TMZ

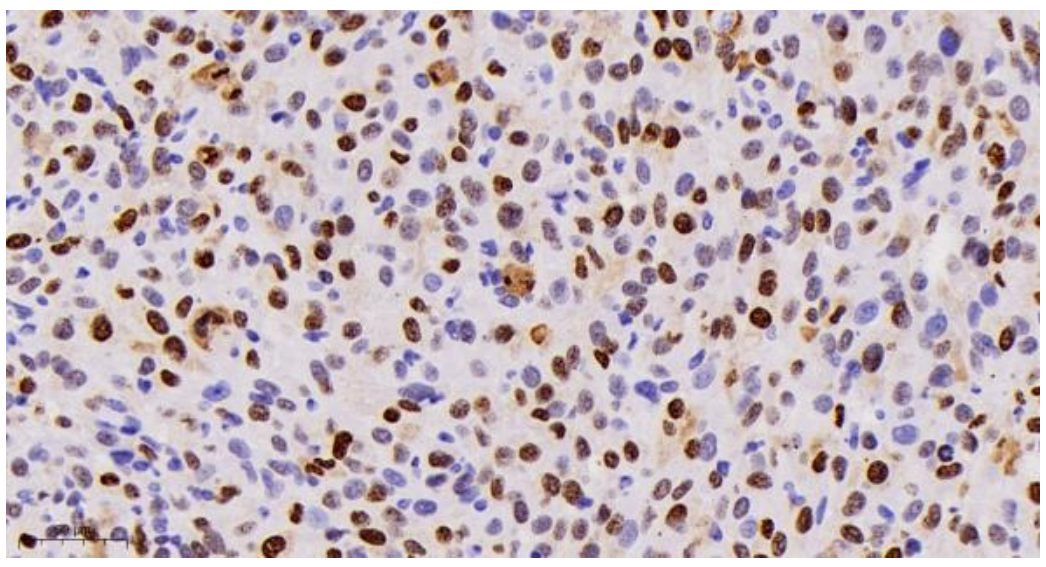

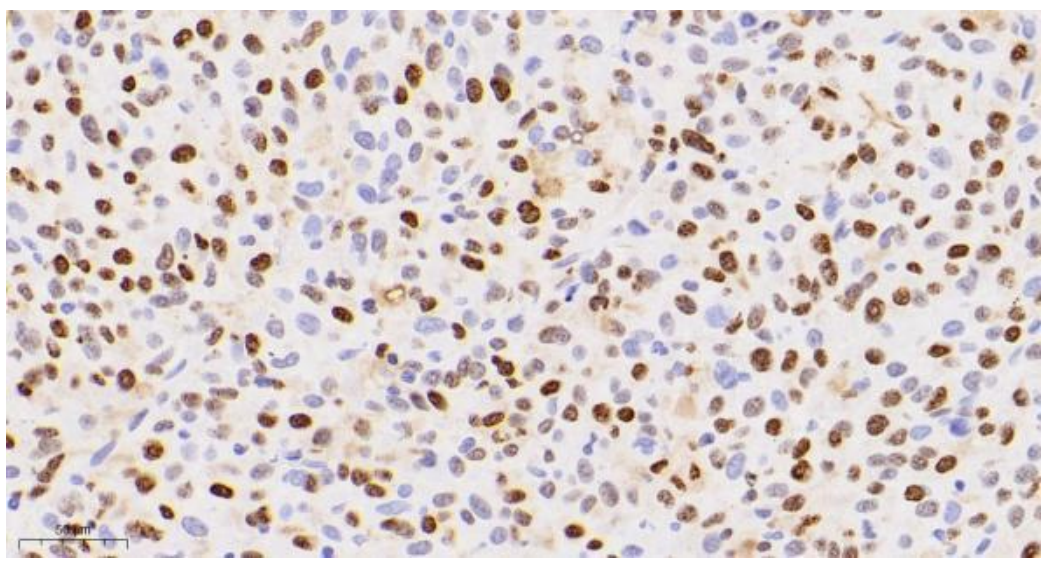

ShPLOD1 shPLOD1+TMZ

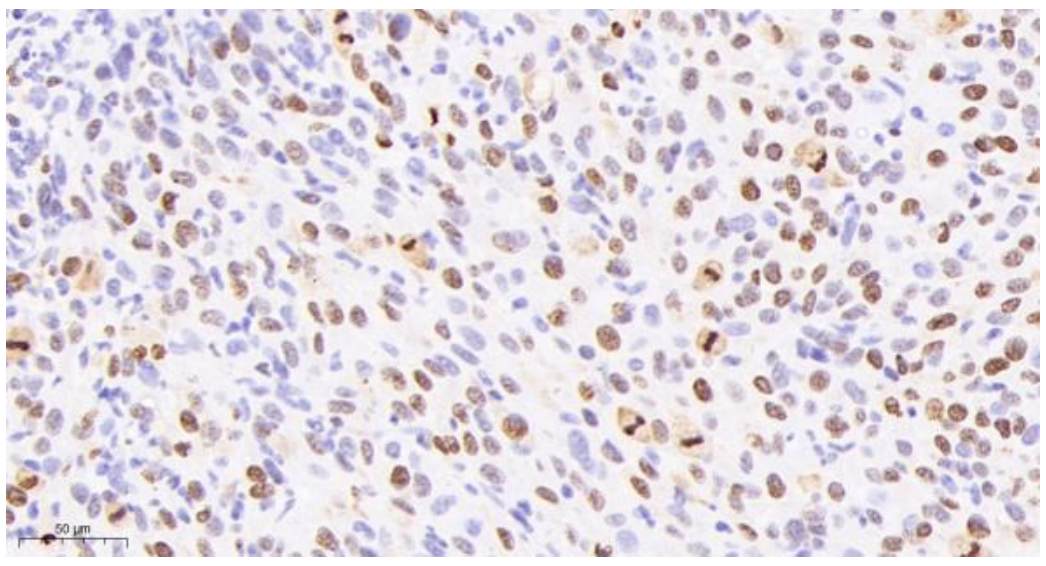

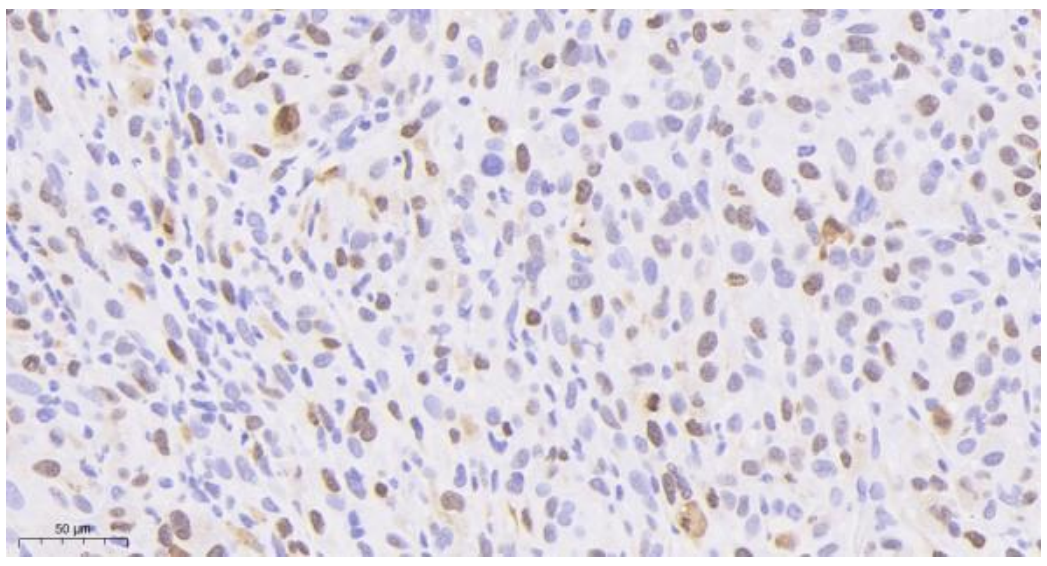

ENO1

ShNC

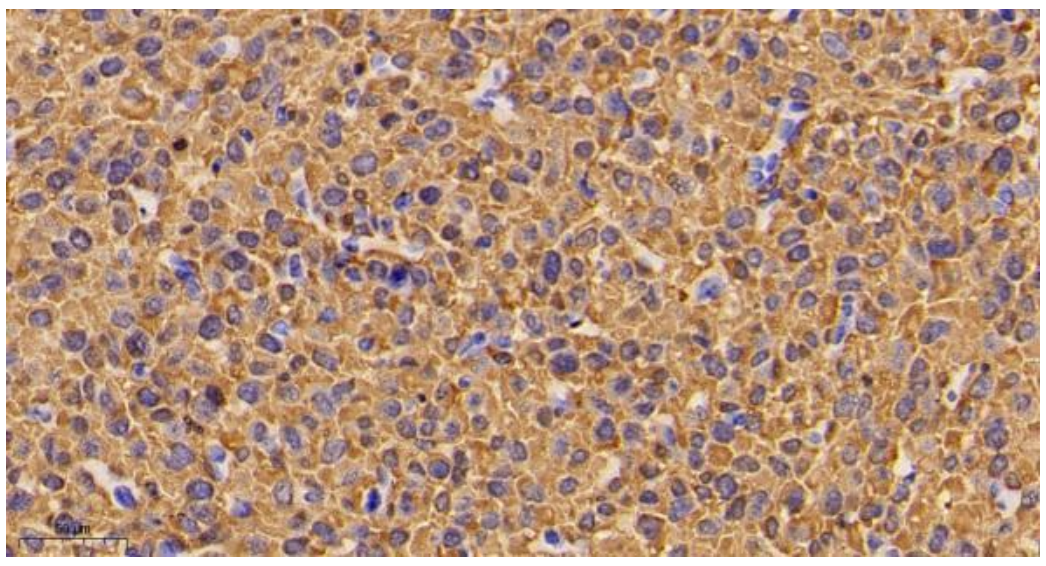

shNC+TMZ

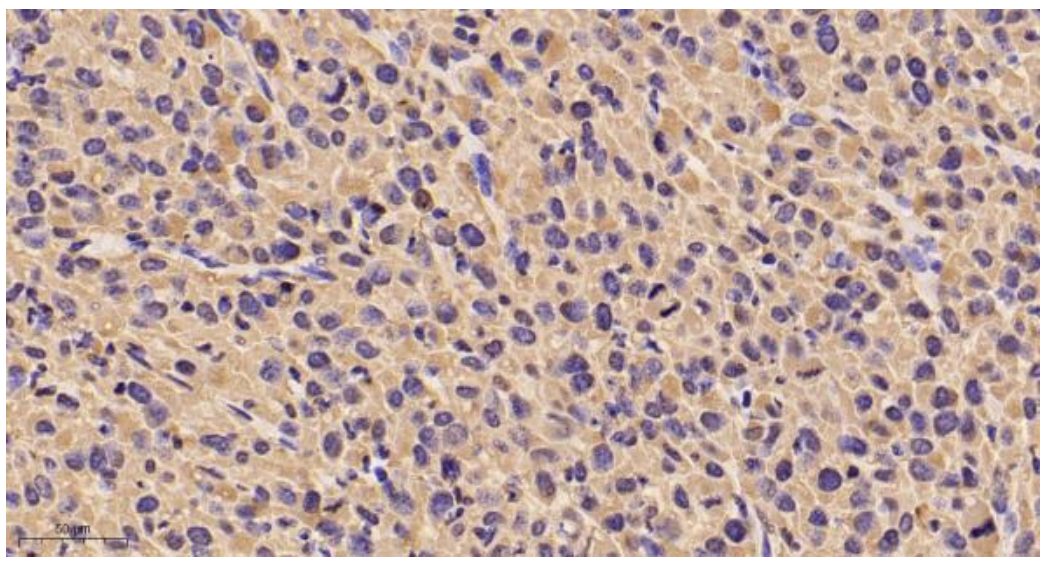

ShPLOD1

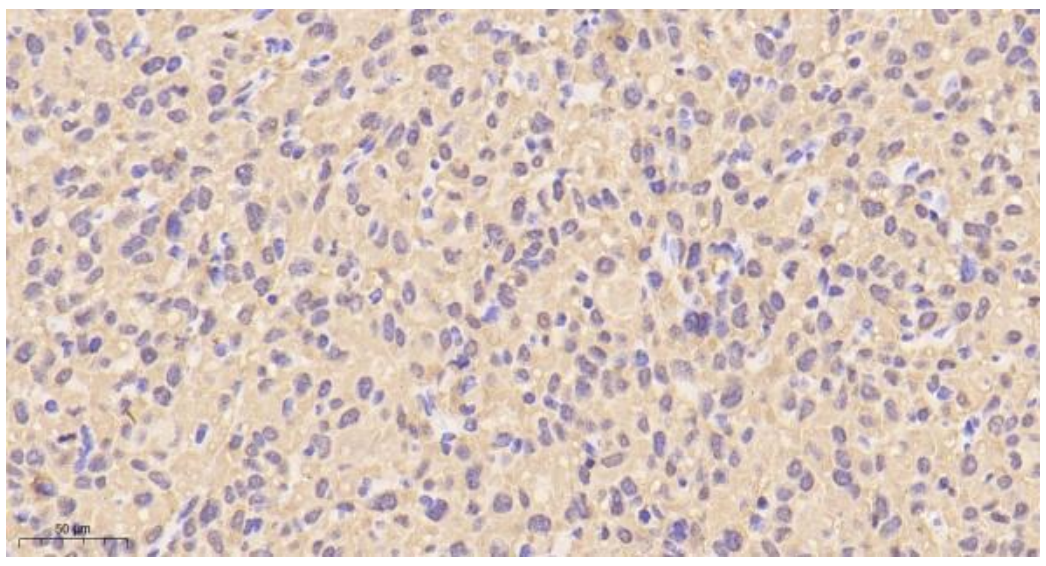

shPLOD1+TMZ

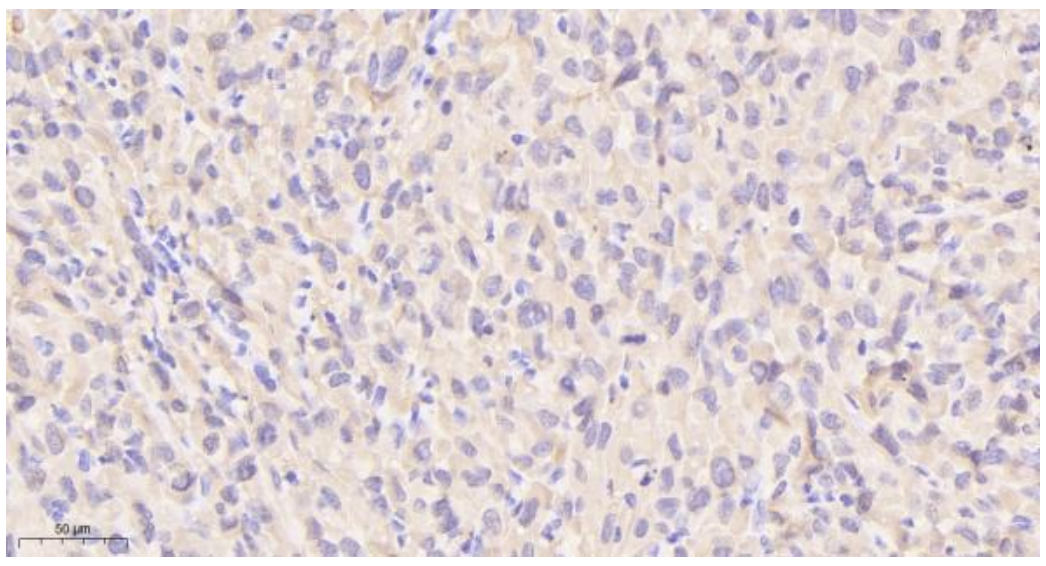

Figure 8A

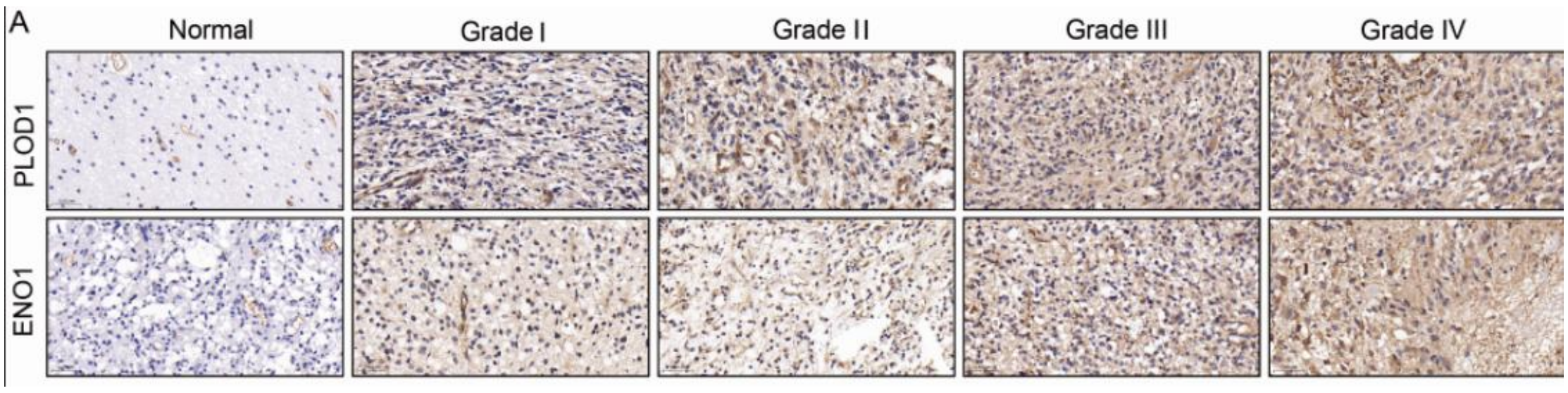

PLOD1

N

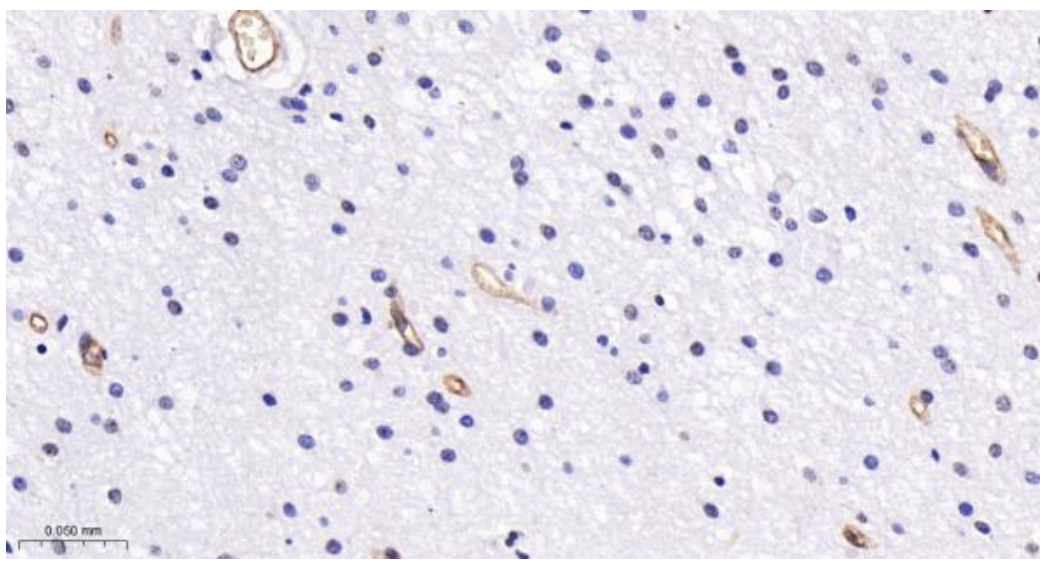

Grade I

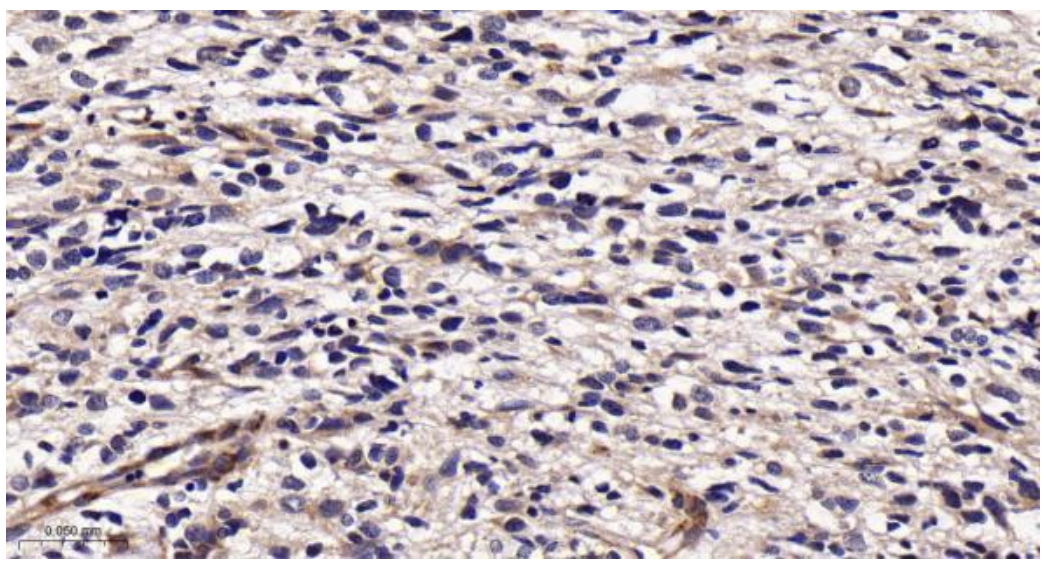

Grade II

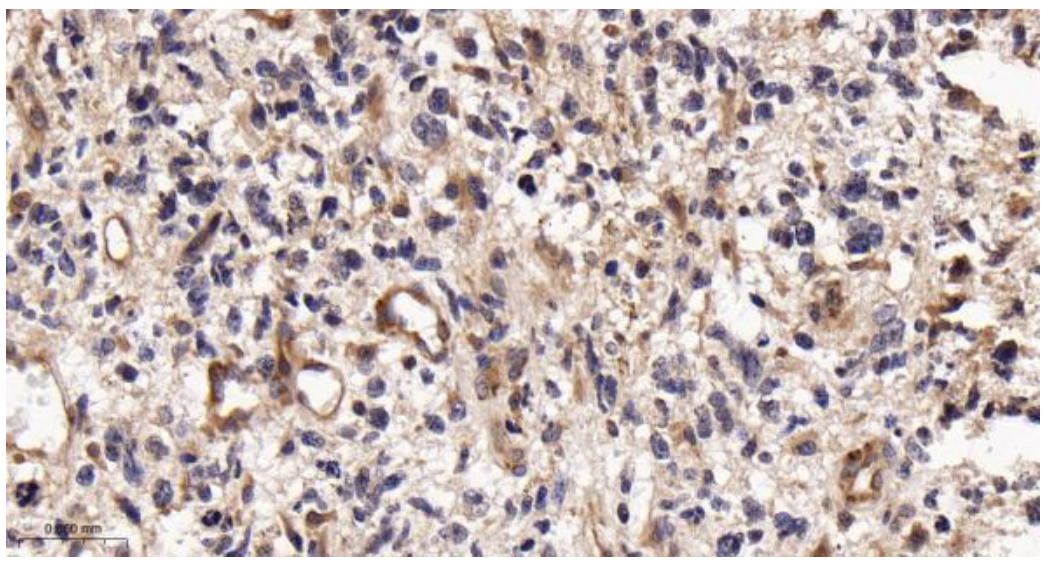

Grade III

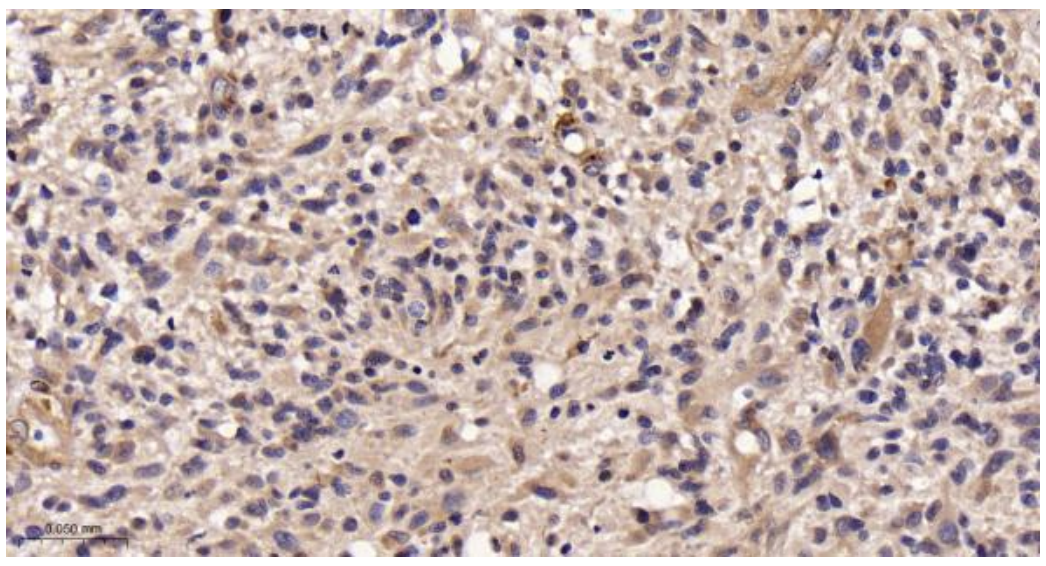

Grade IV

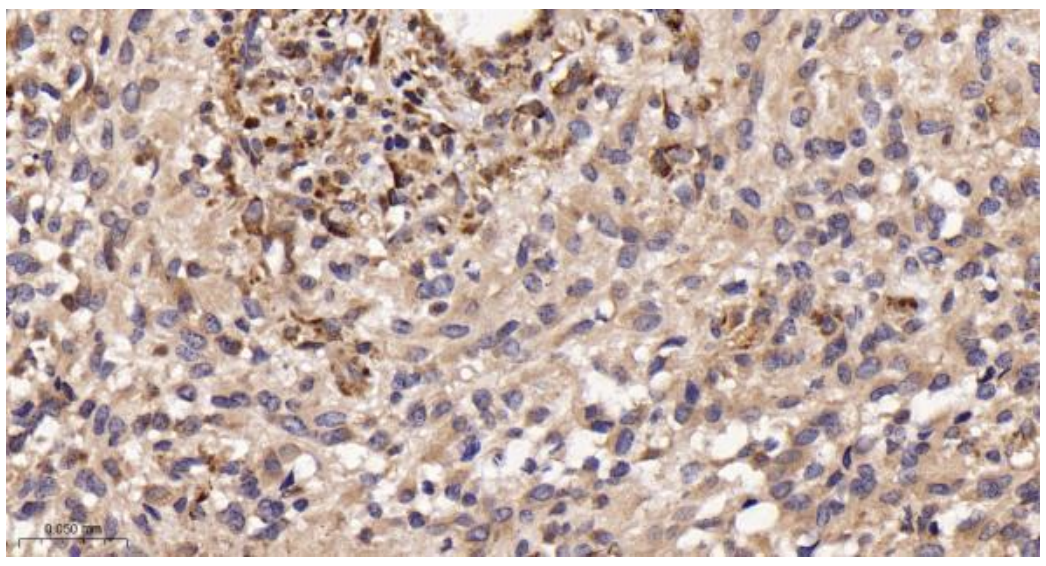

ENO1

N

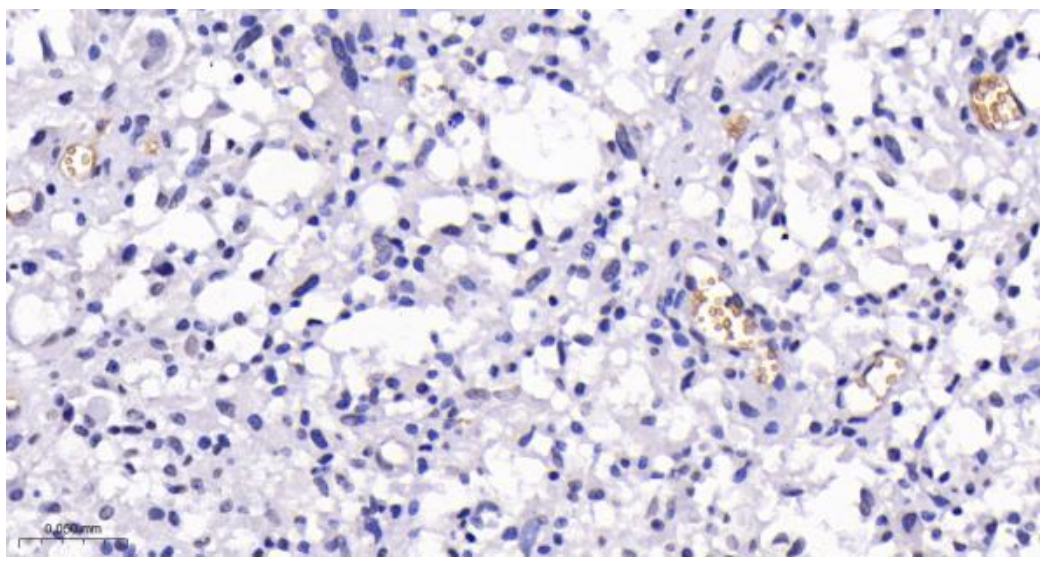

Grade I

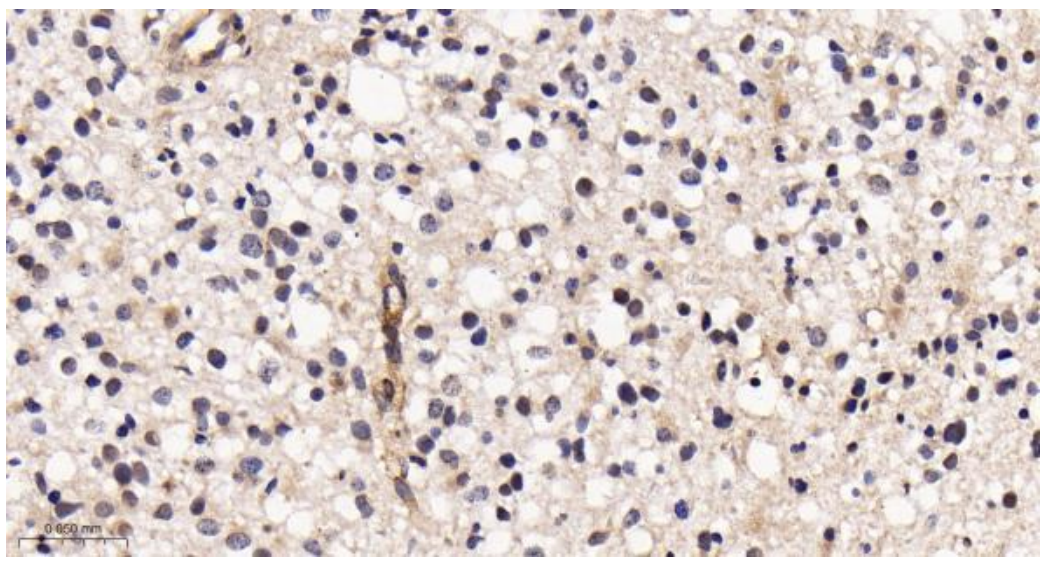

Grade II

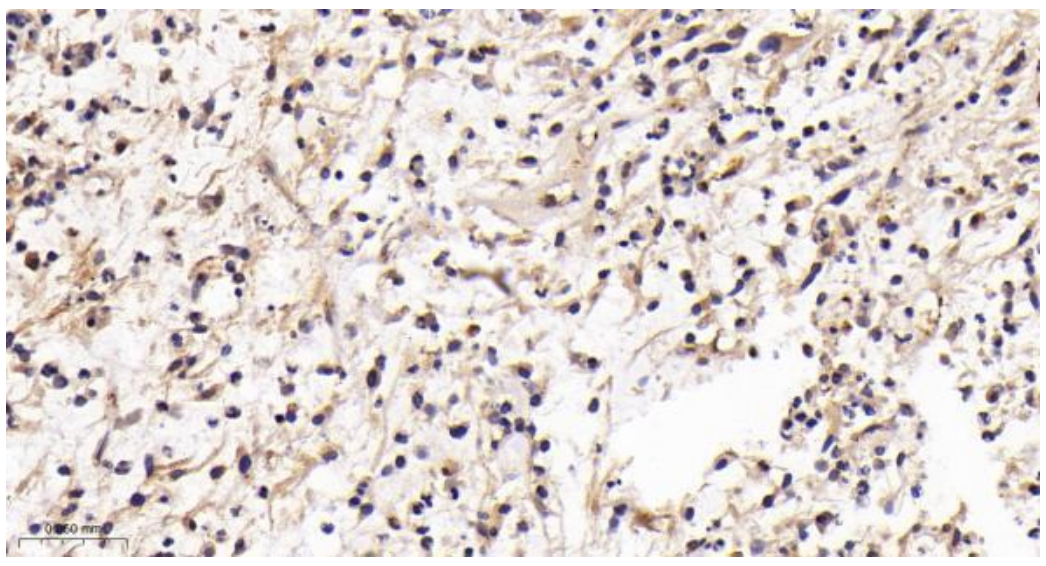

Grade III

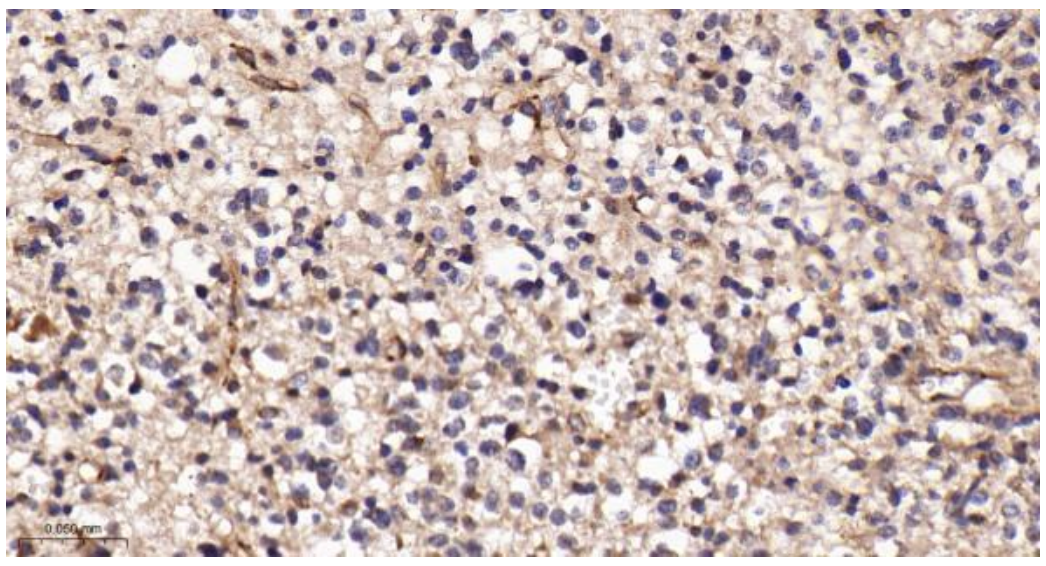

Grade IV

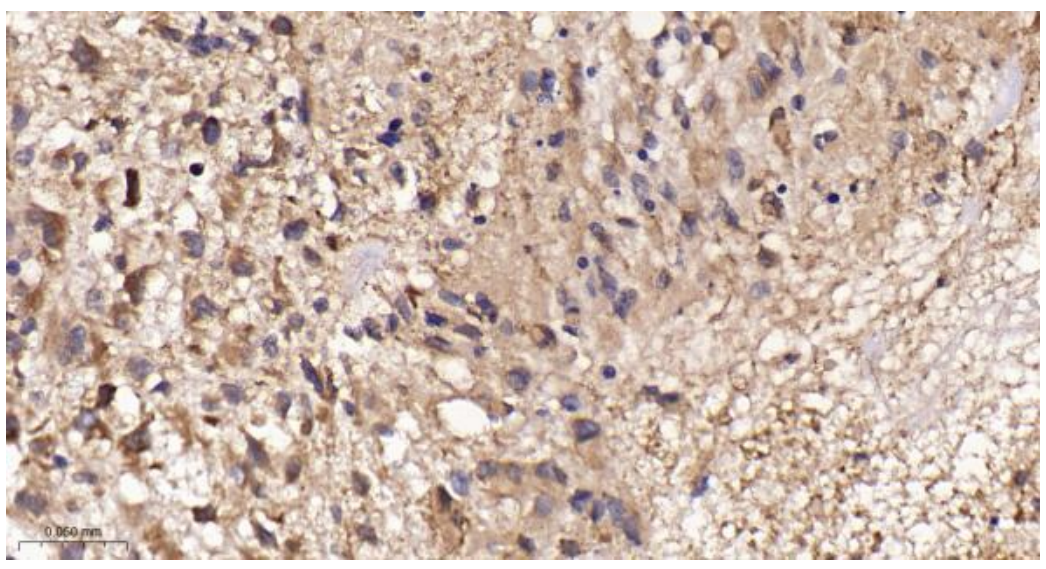

Figure S2C

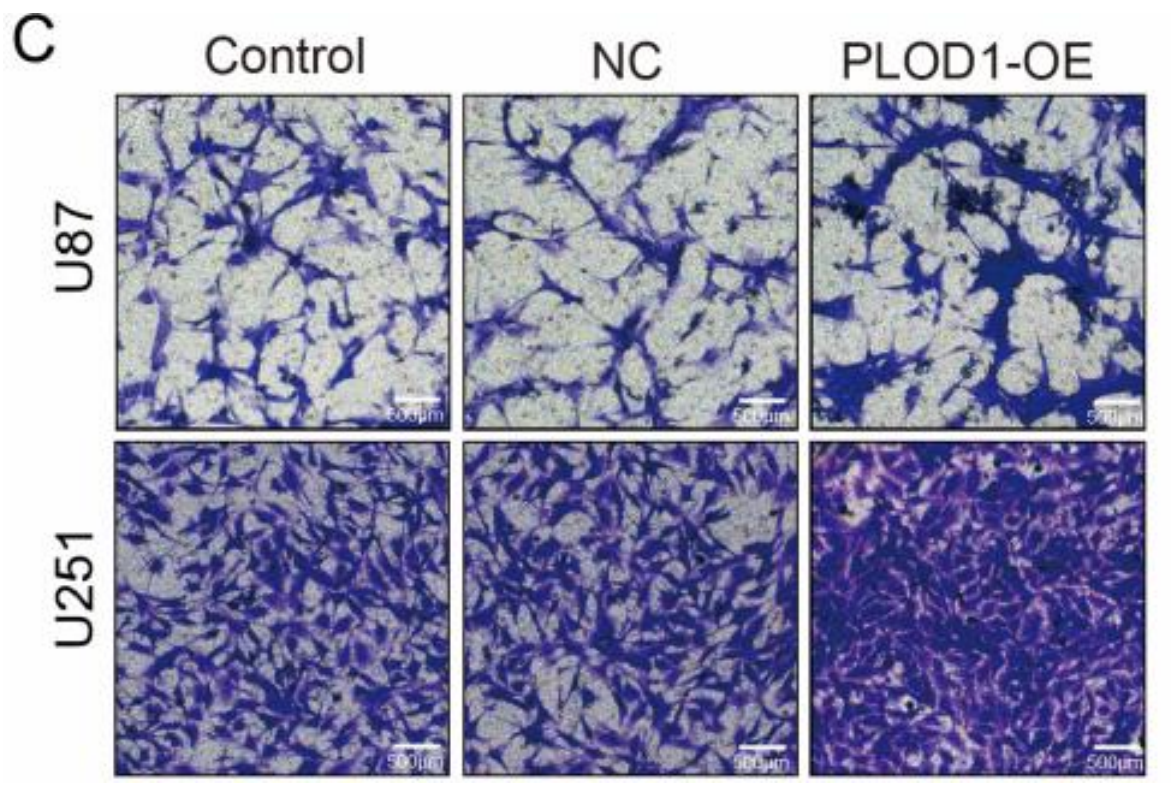

U87

Control

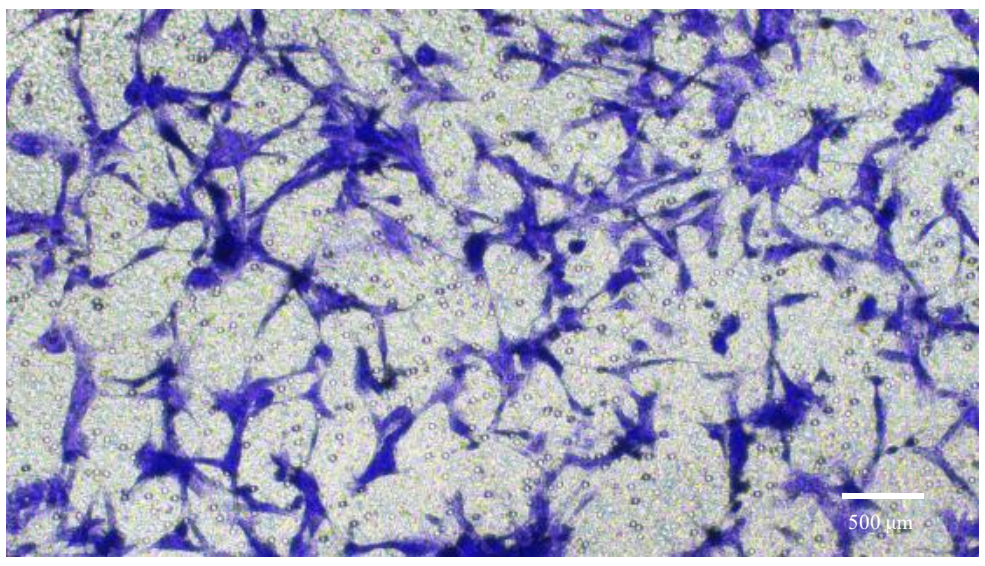

NC

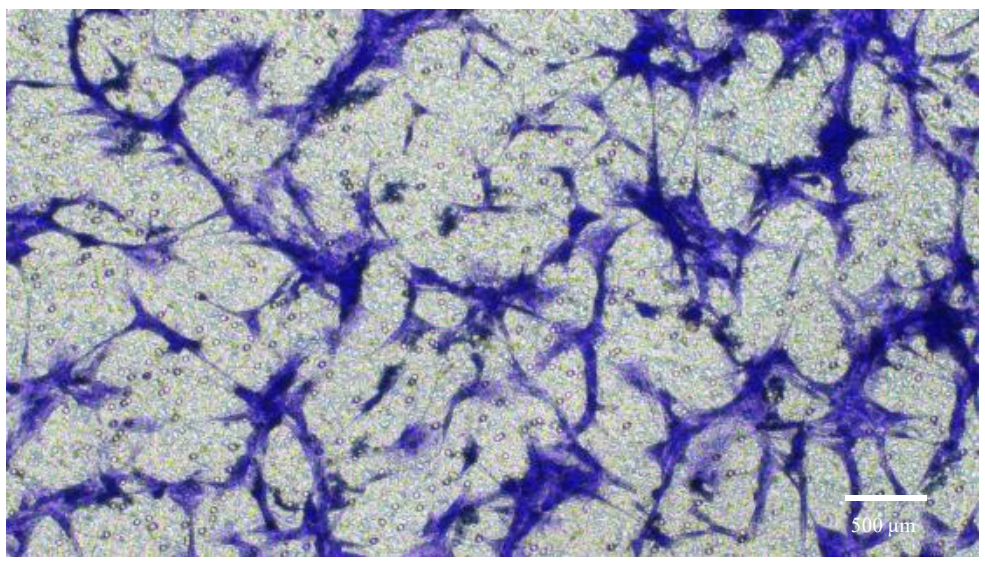

PLOD1-OE

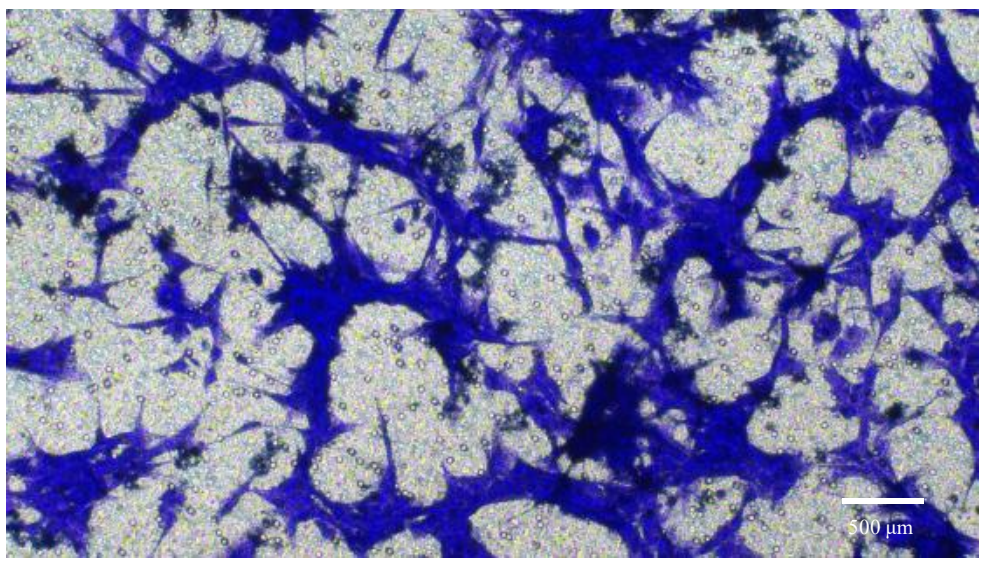

U251

Control

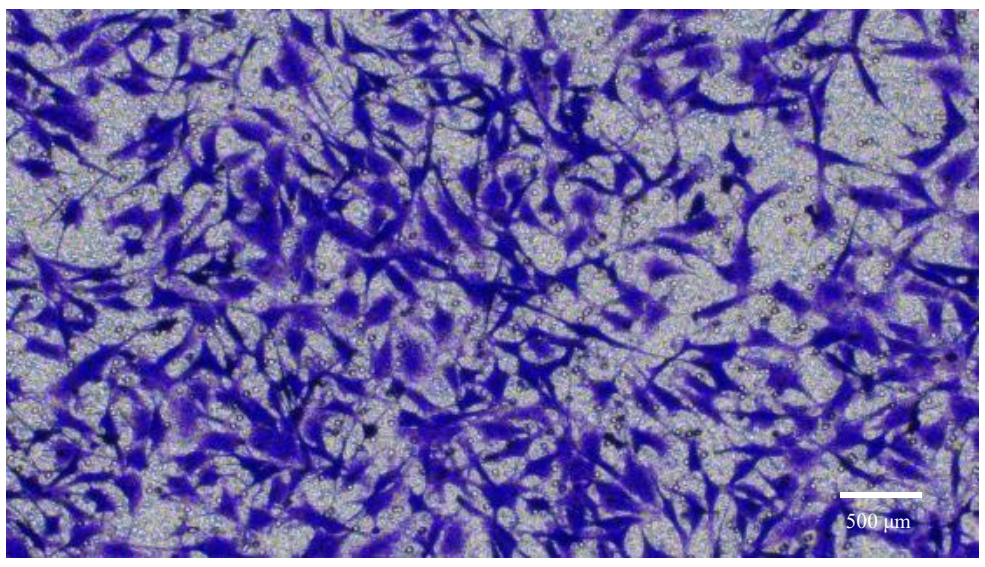

NC

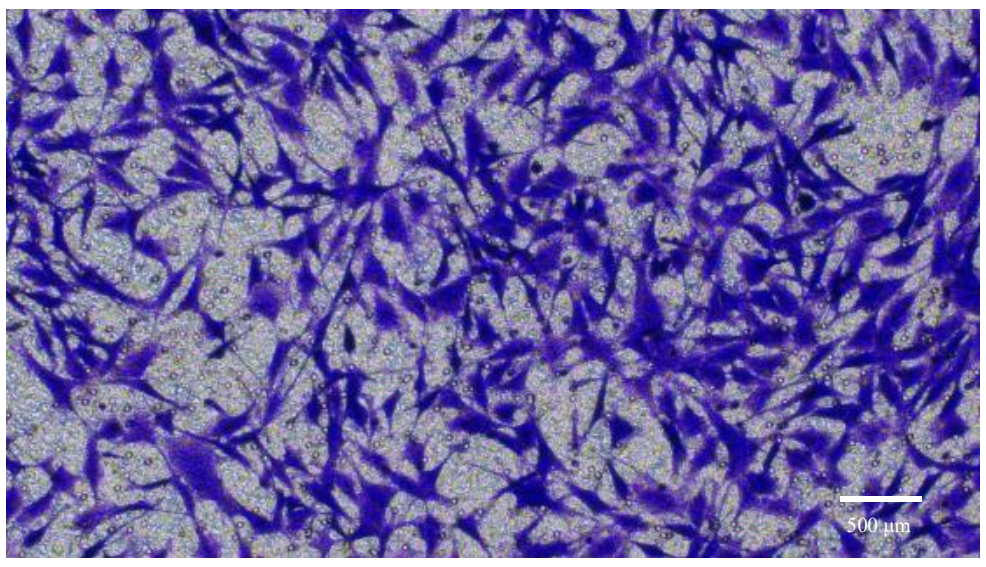

PLOD1-OE

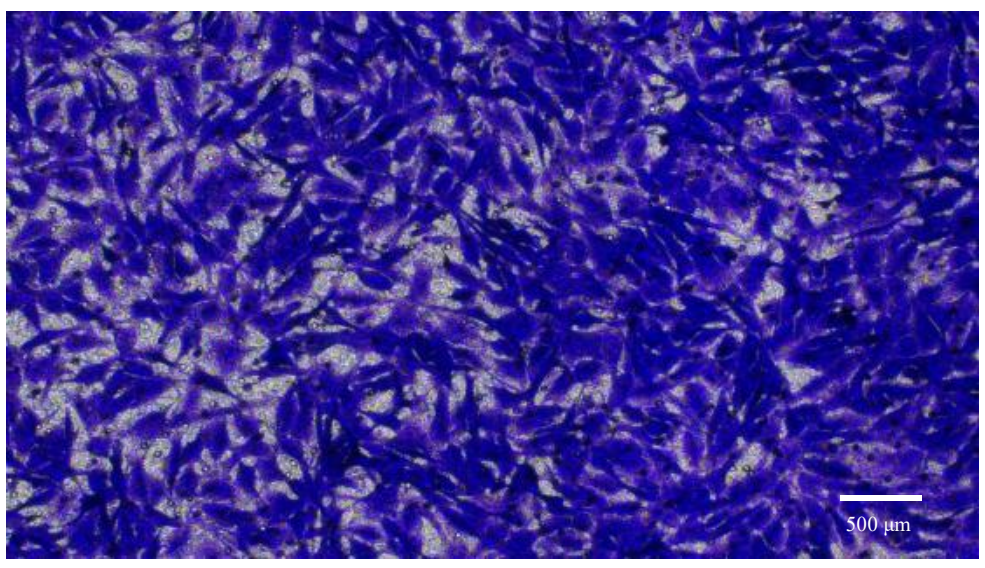

Figure S2D

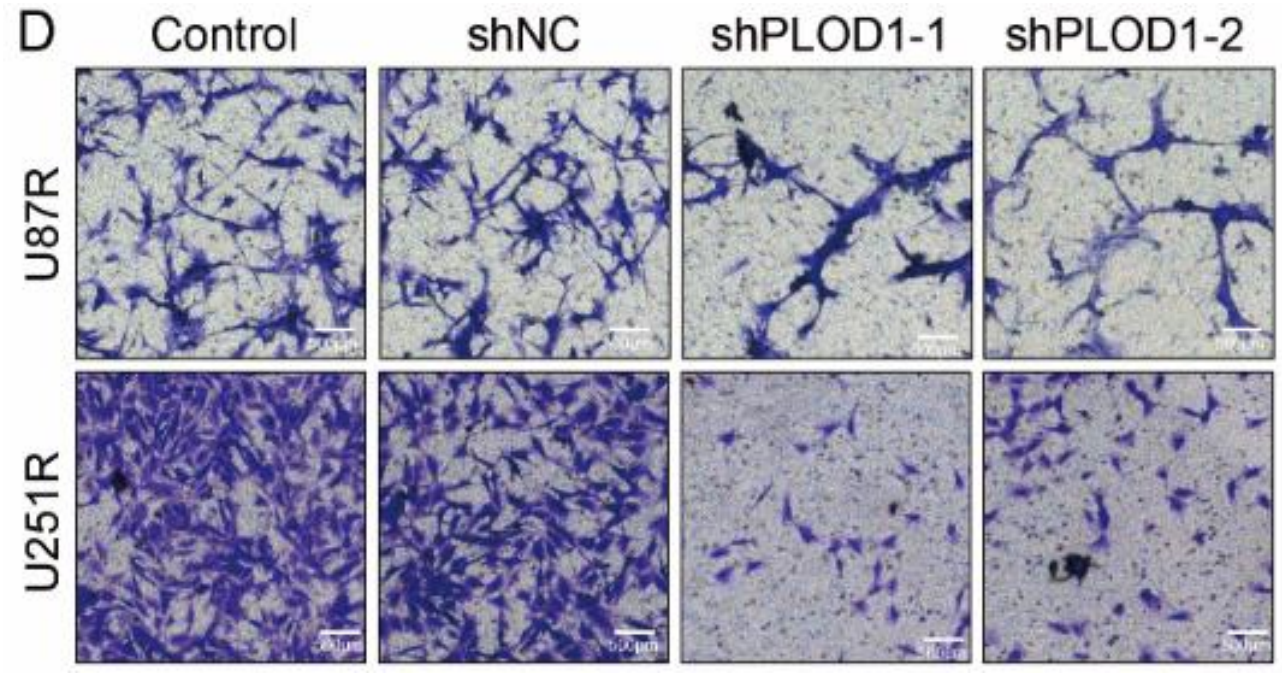

U87R

Control | shNC

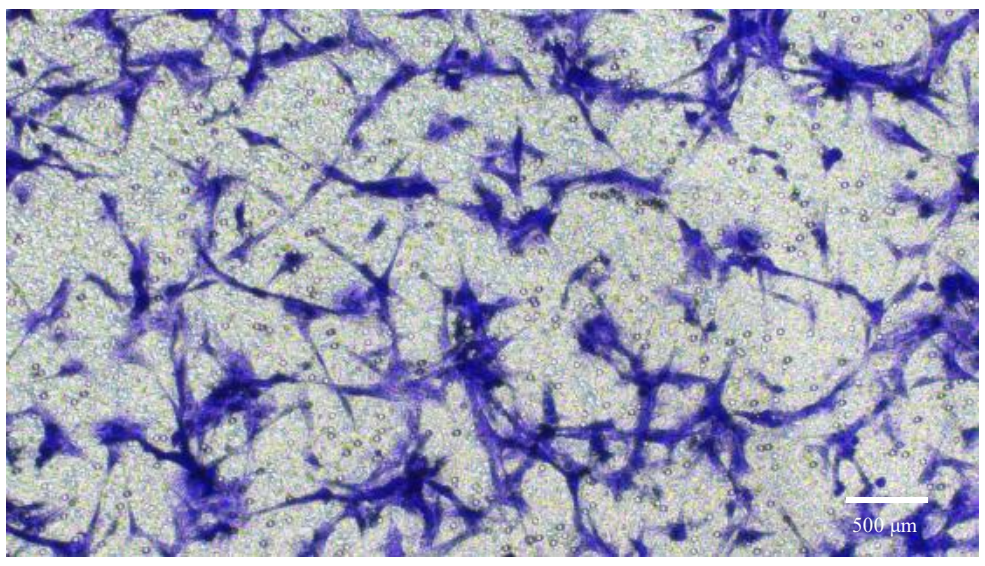
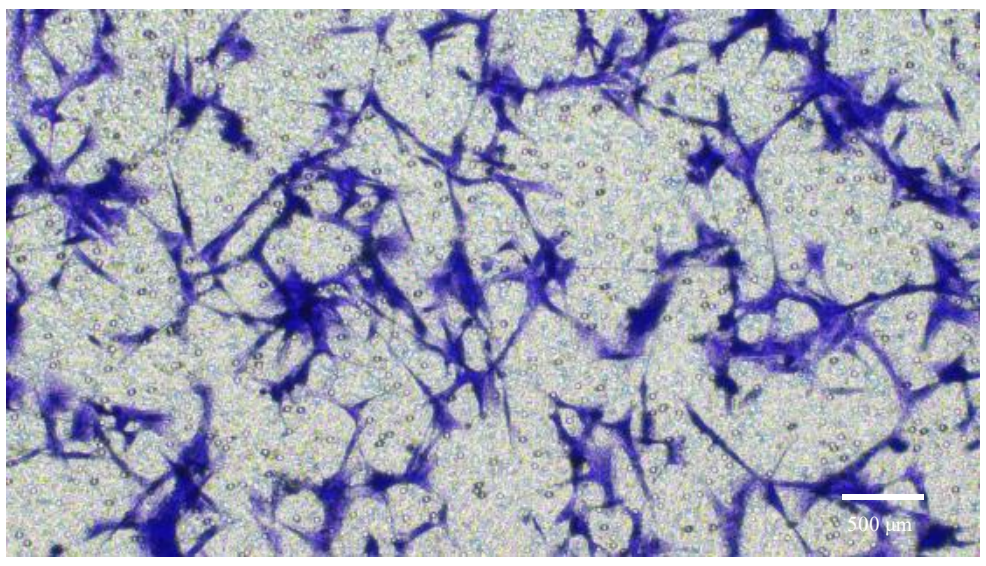

ShPLOD1-1 | ShPLOD1-2

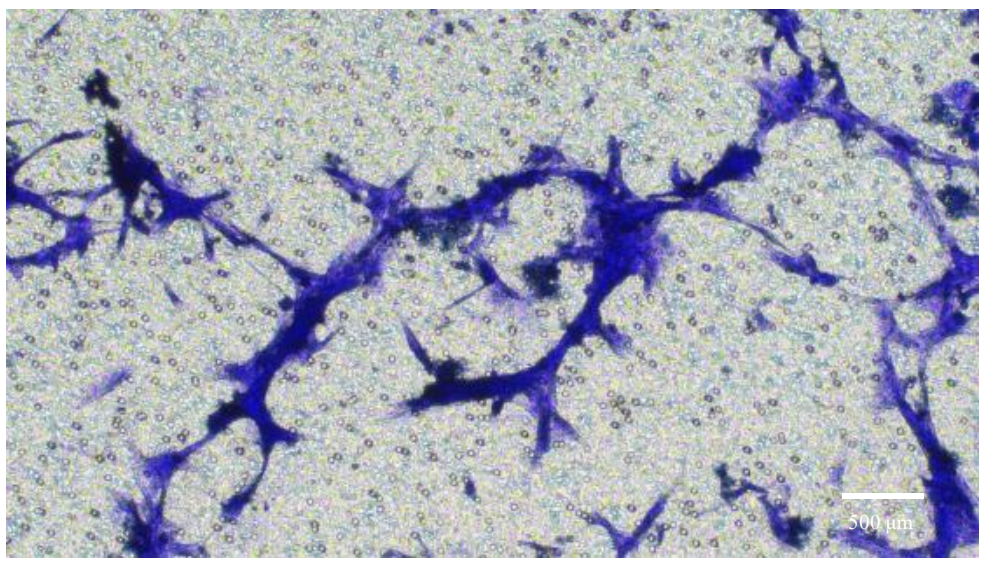
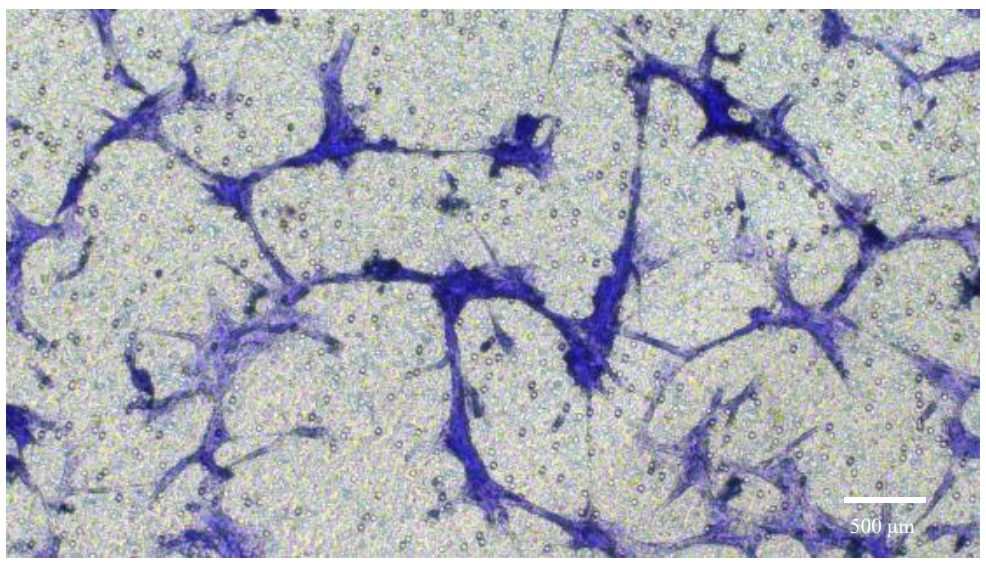

U251R

Control | shNC

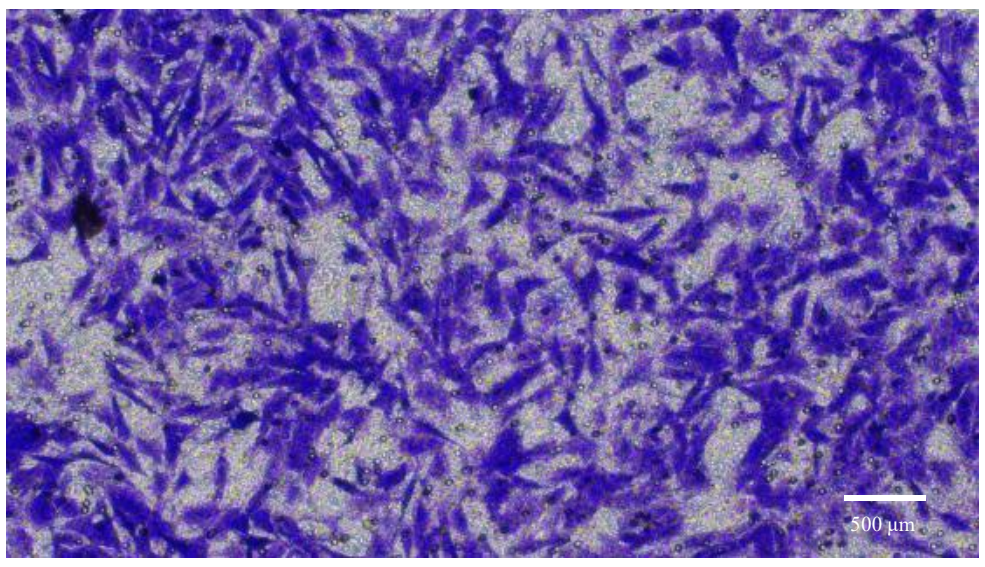
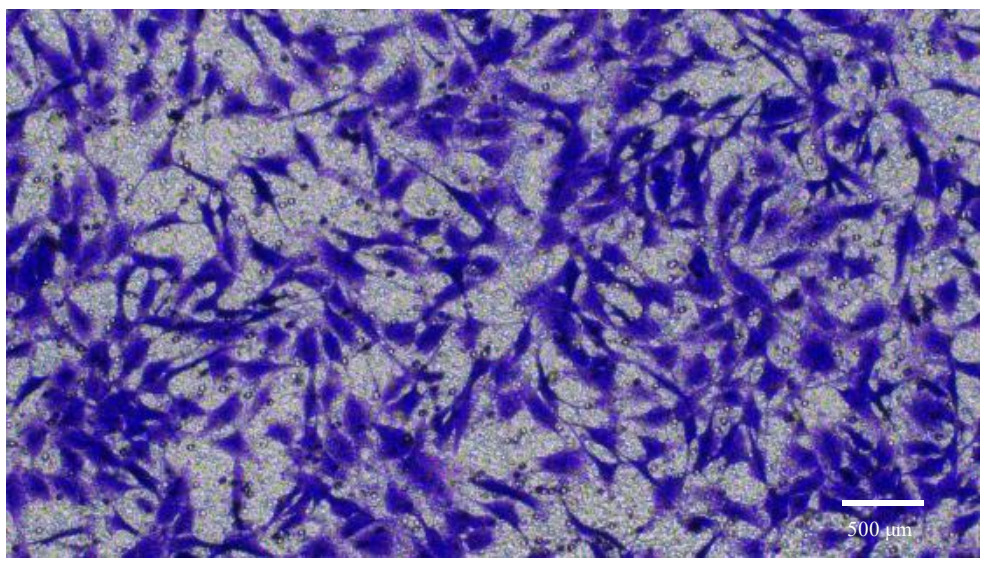

ShPLOD1-1 | ShPLOD1-2

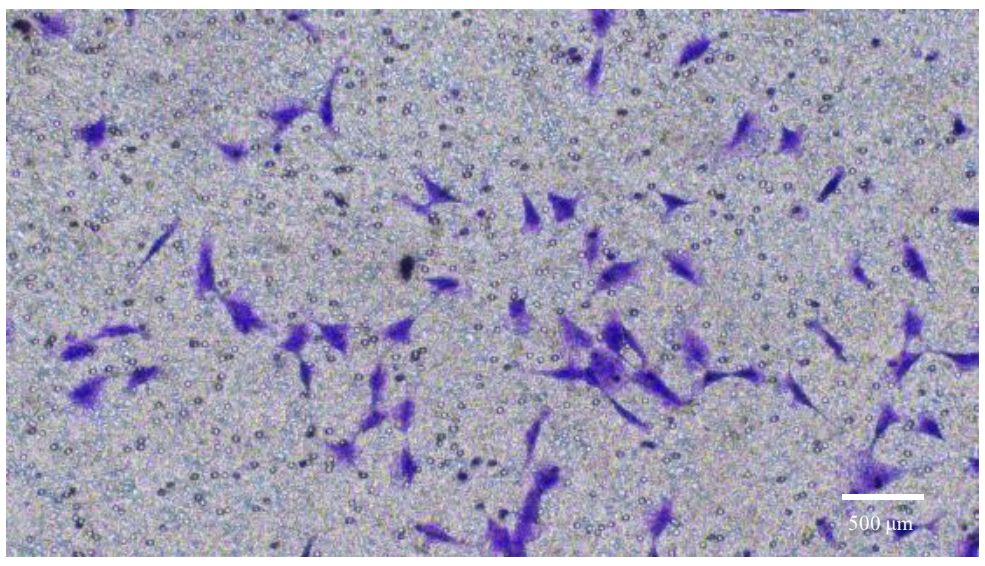

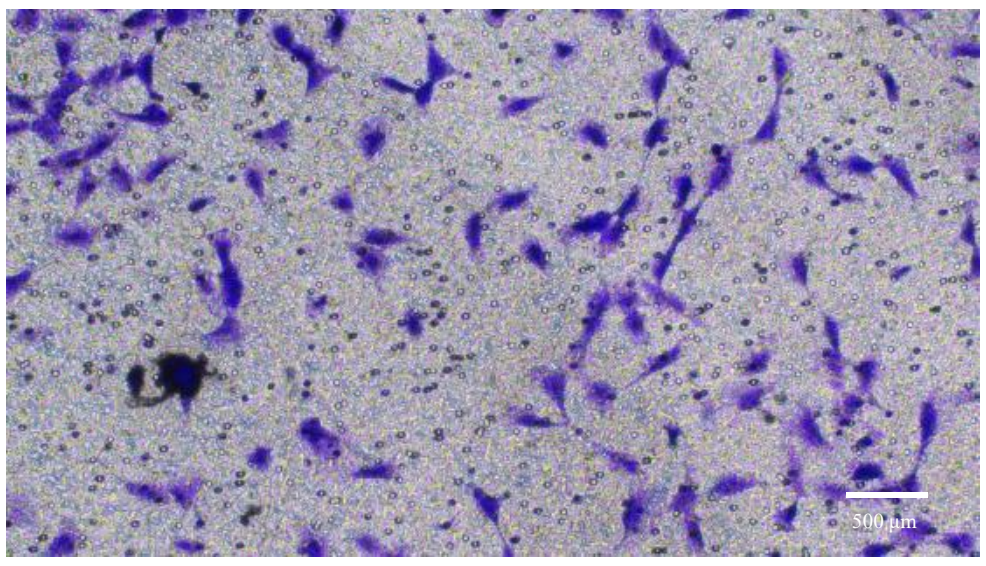

Figure S4H

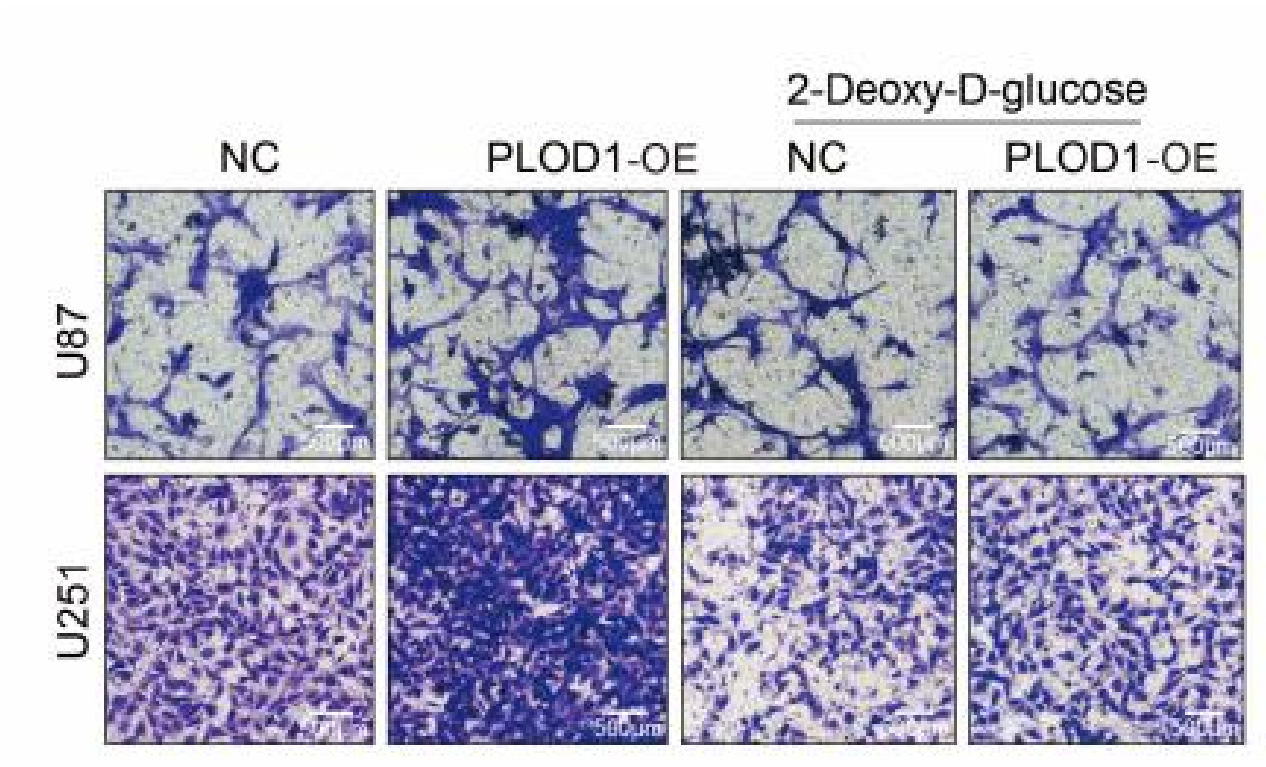

U87

NC

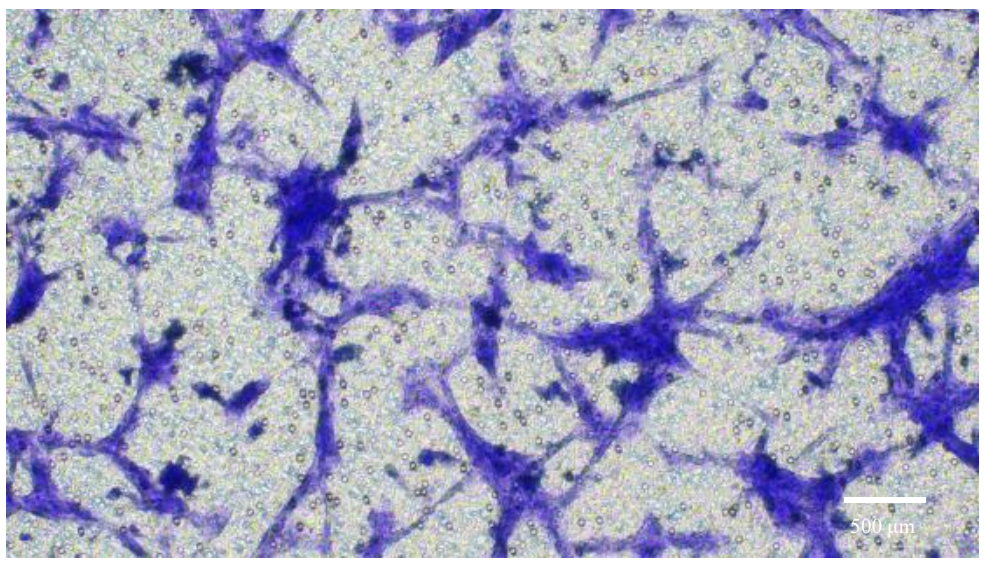

PLOD1-OE

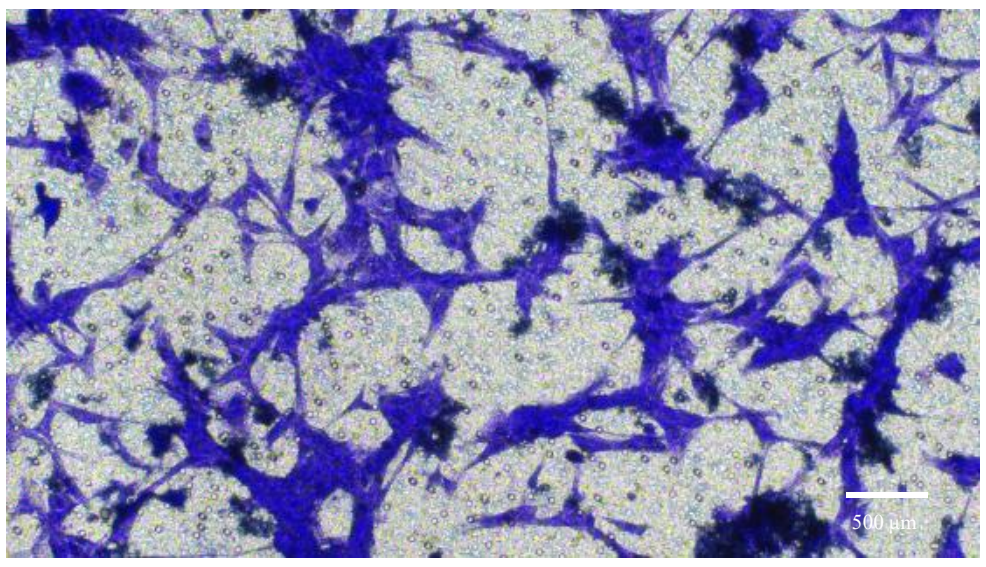

NC+2-Deoxy-D-glucose

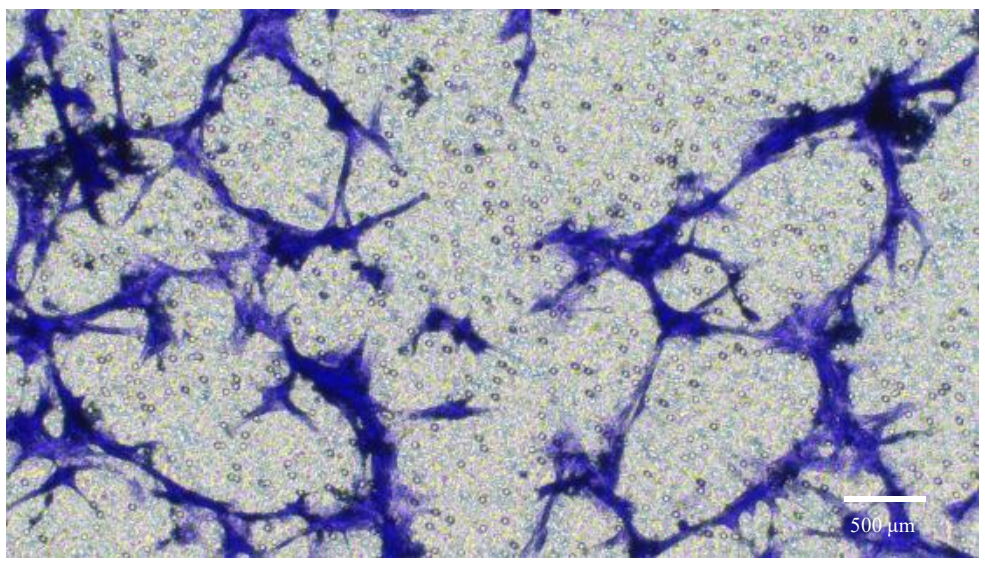

PLOD1-OE+2-Deoxy-D-glucose

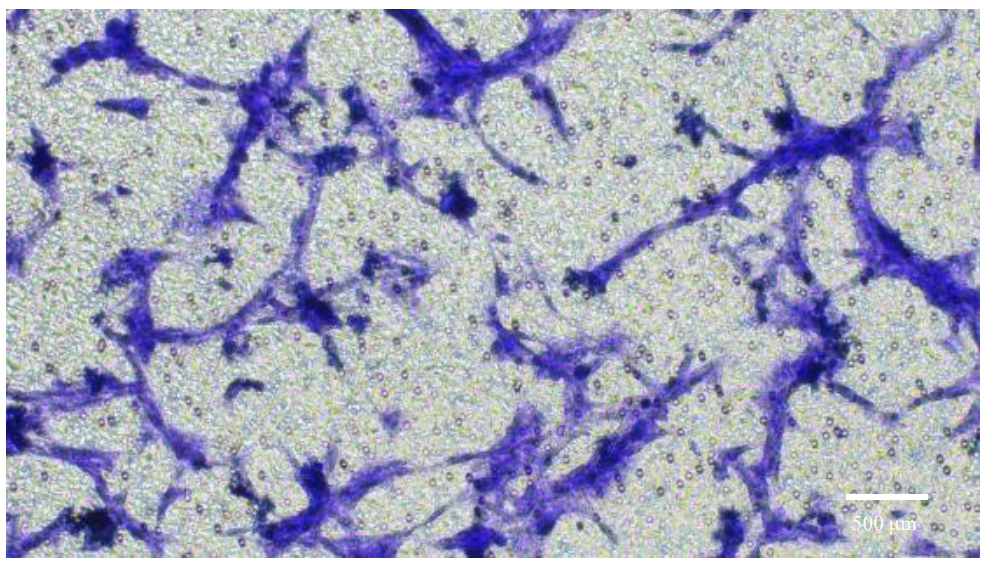

U251

NC

PLOD1-OE

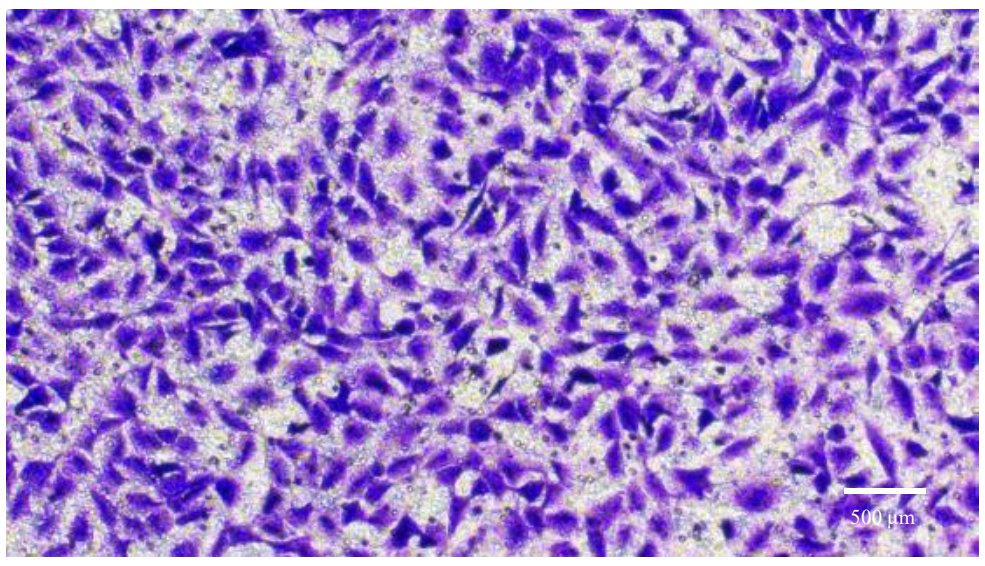

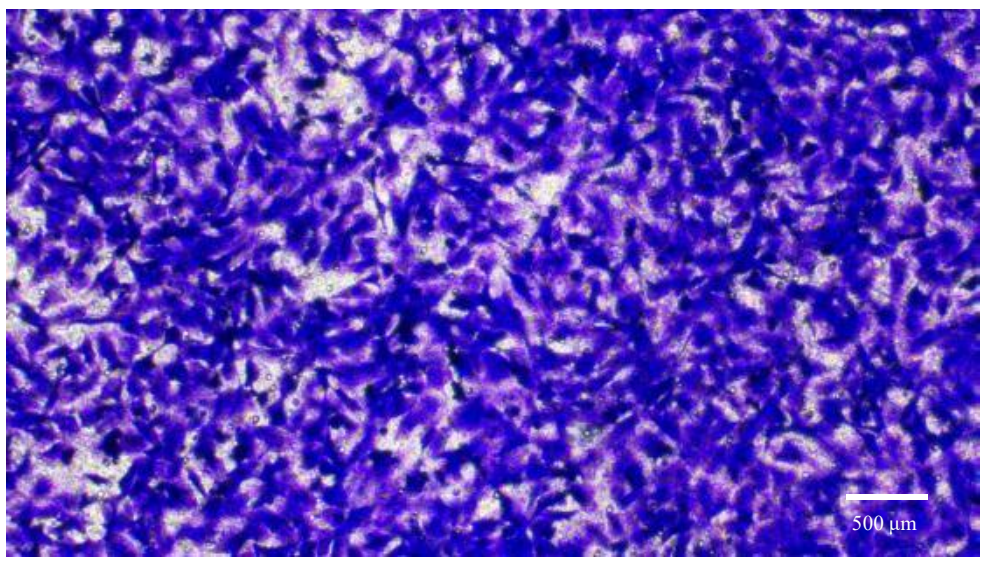

NC+2-Deoxy-D-glucose

PLOD1-OE+2-Deoxy-D-glucose

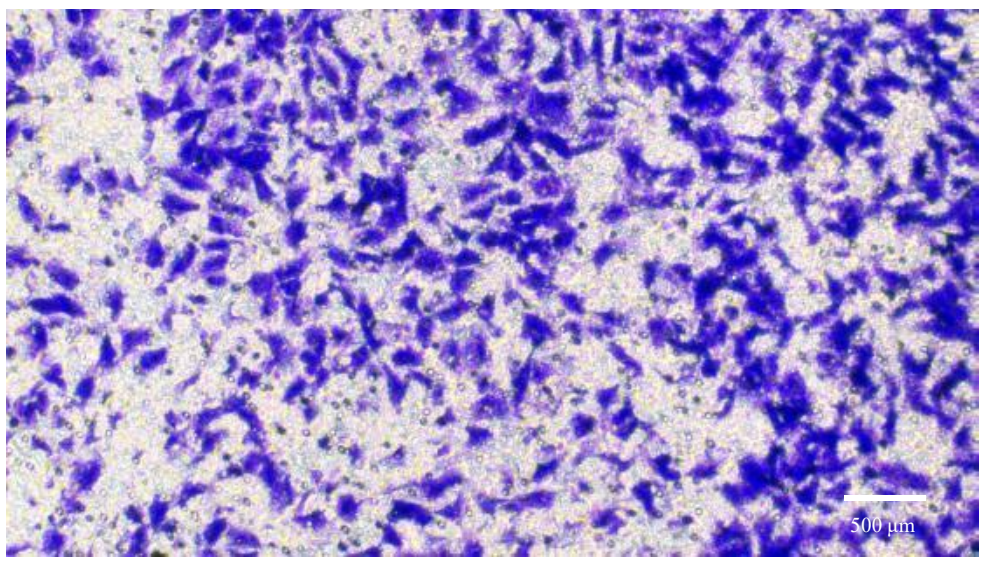

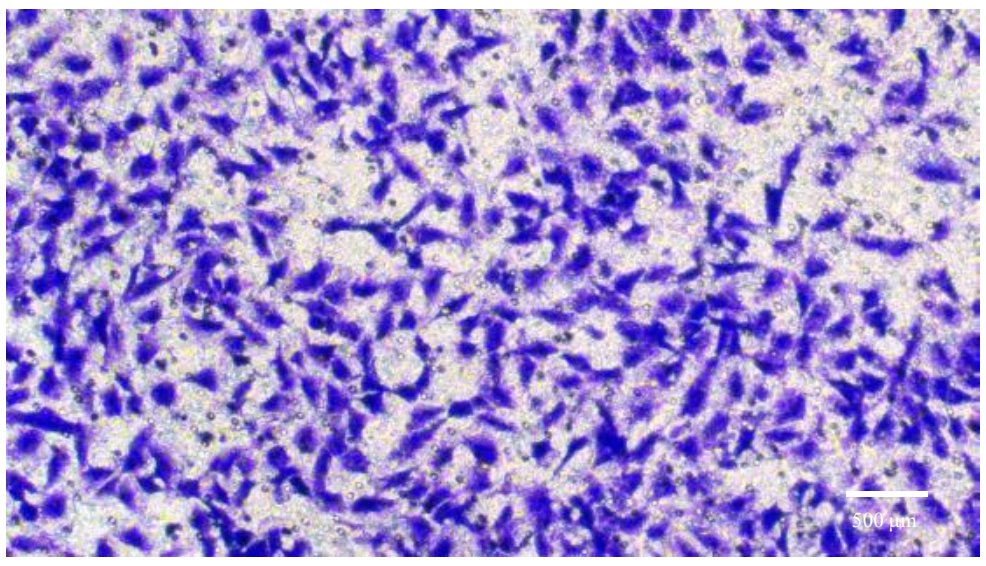

Figure S7A

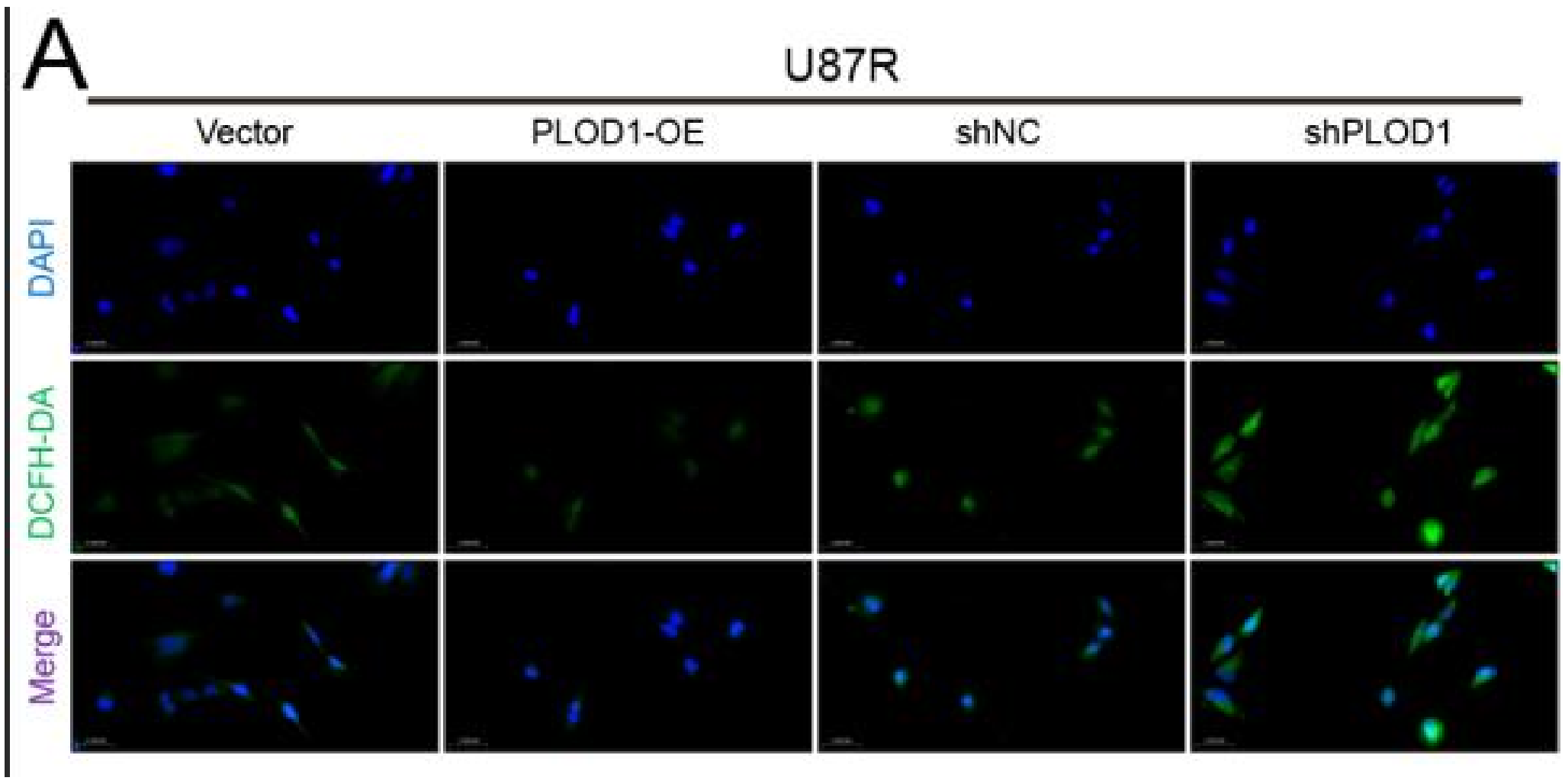

U87R

Vector

DAPI

DCFH-DA

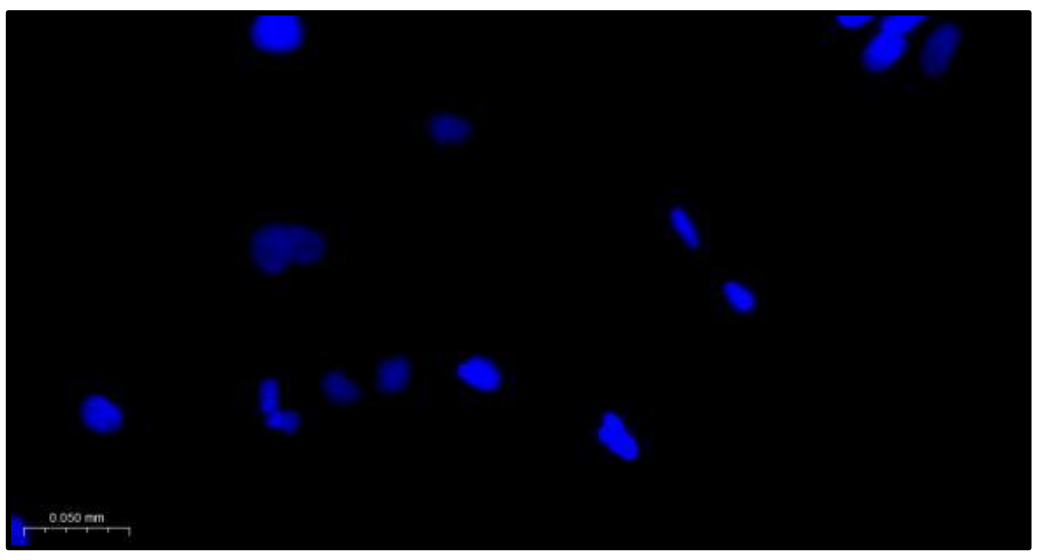

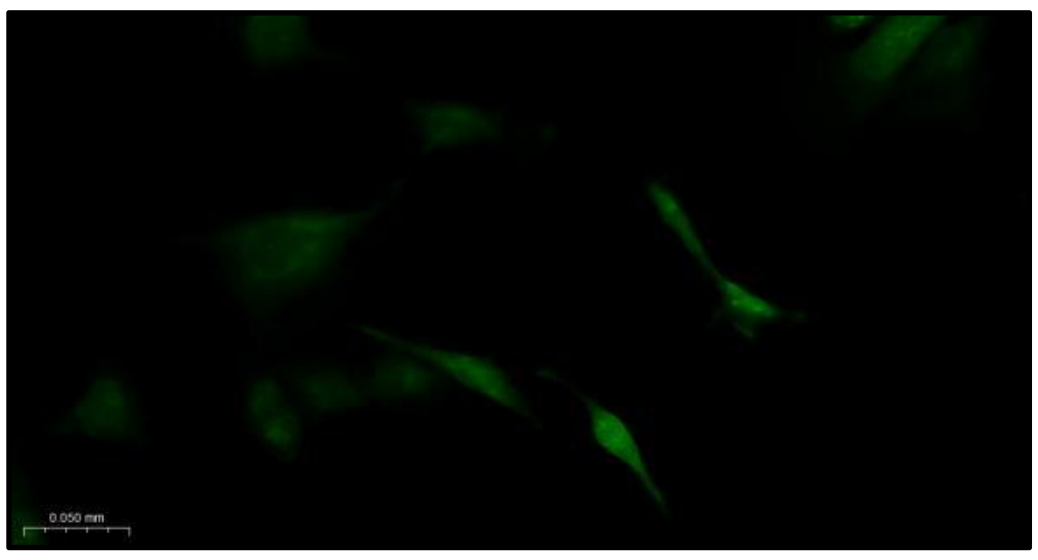

Merge

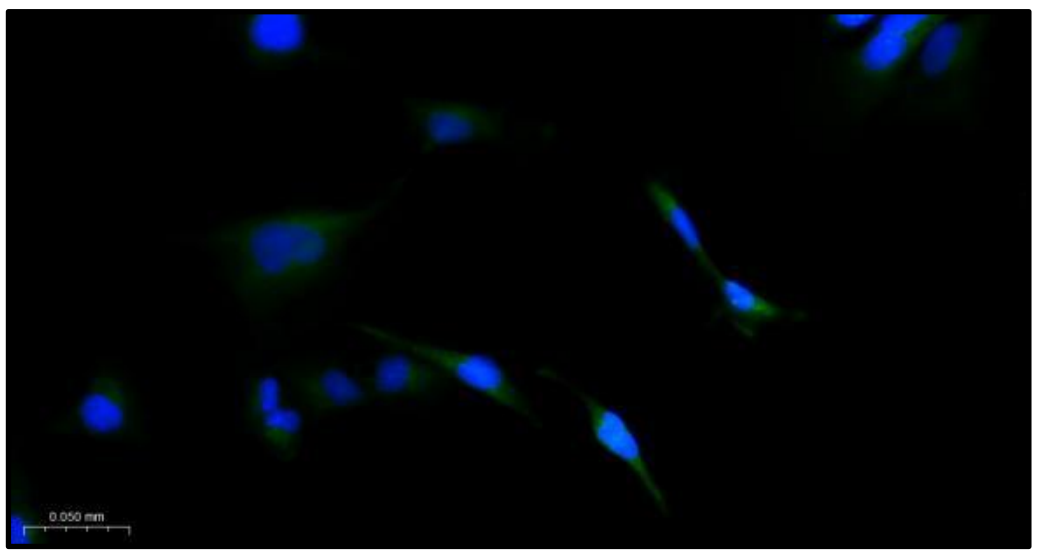

PLOD1-OE

DAPI

DCFH-DA

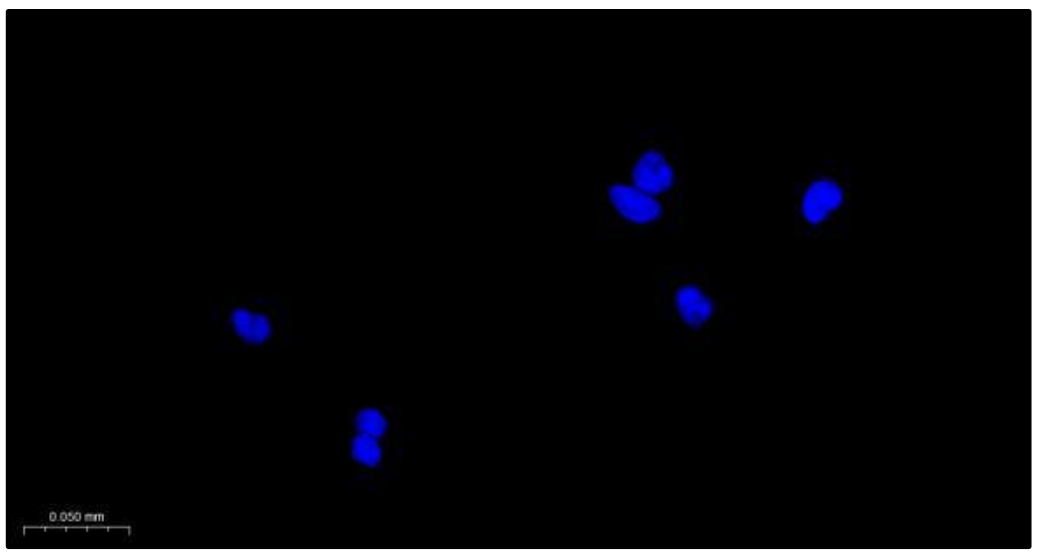

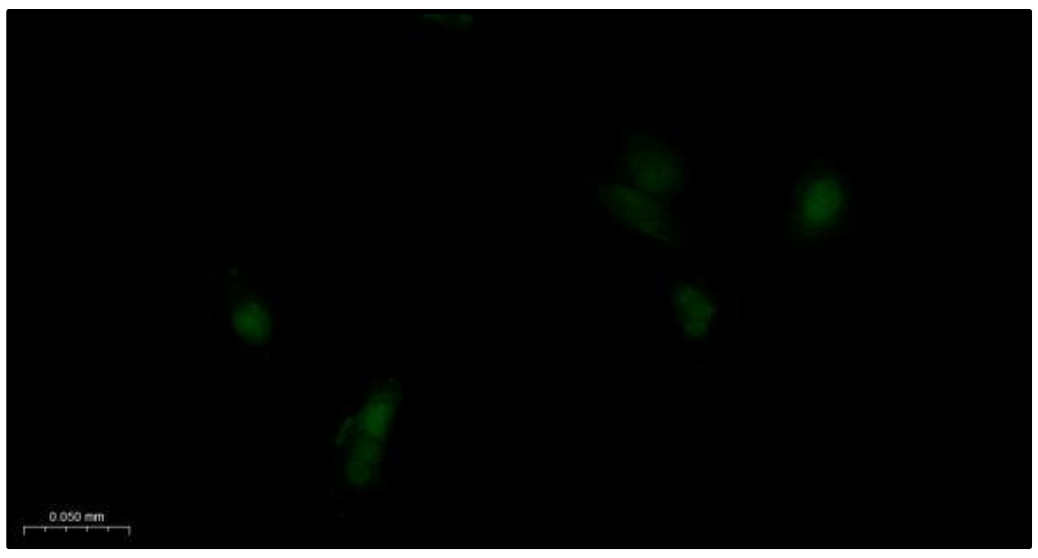

Merge

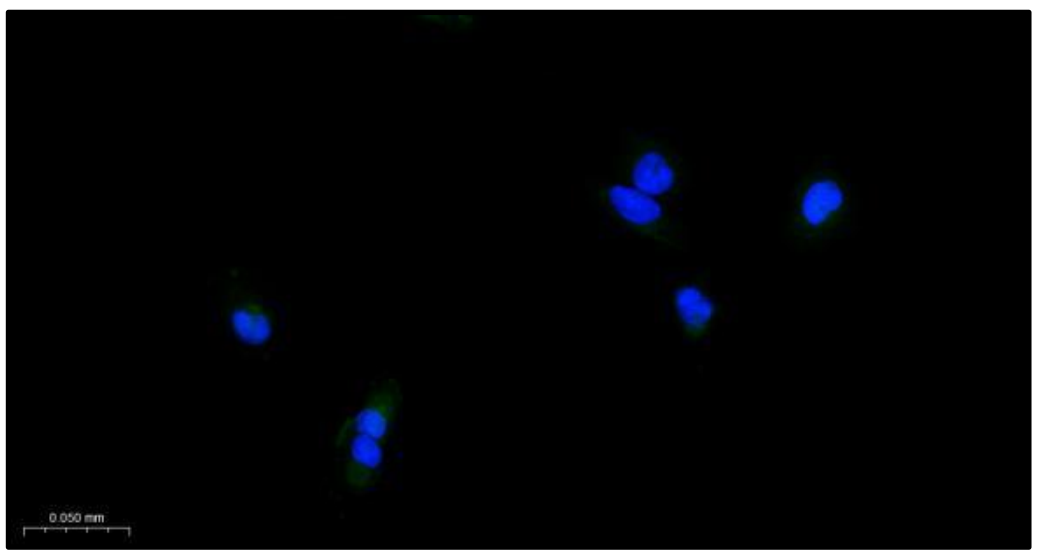

ShNC

DAPI

DCFH-DA

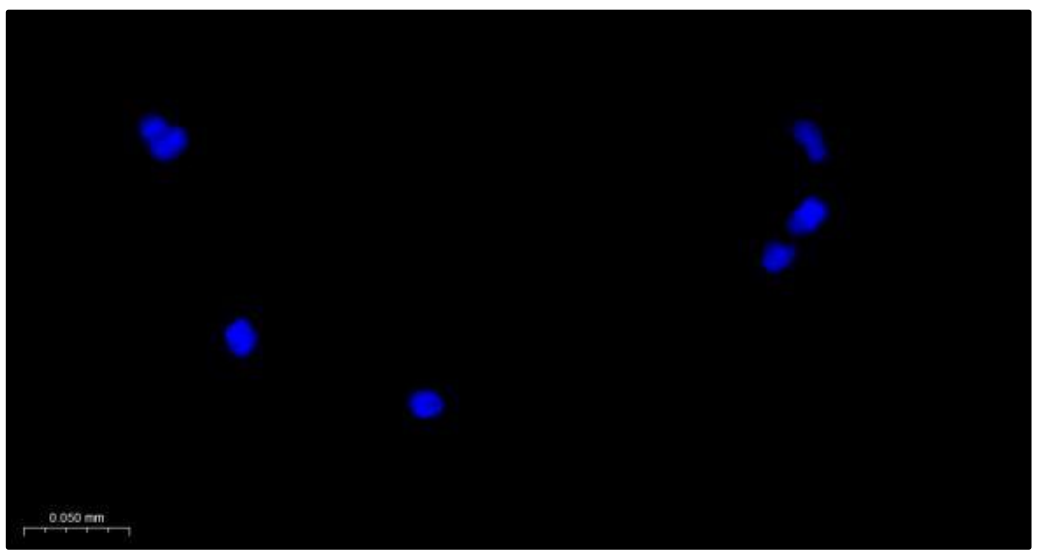

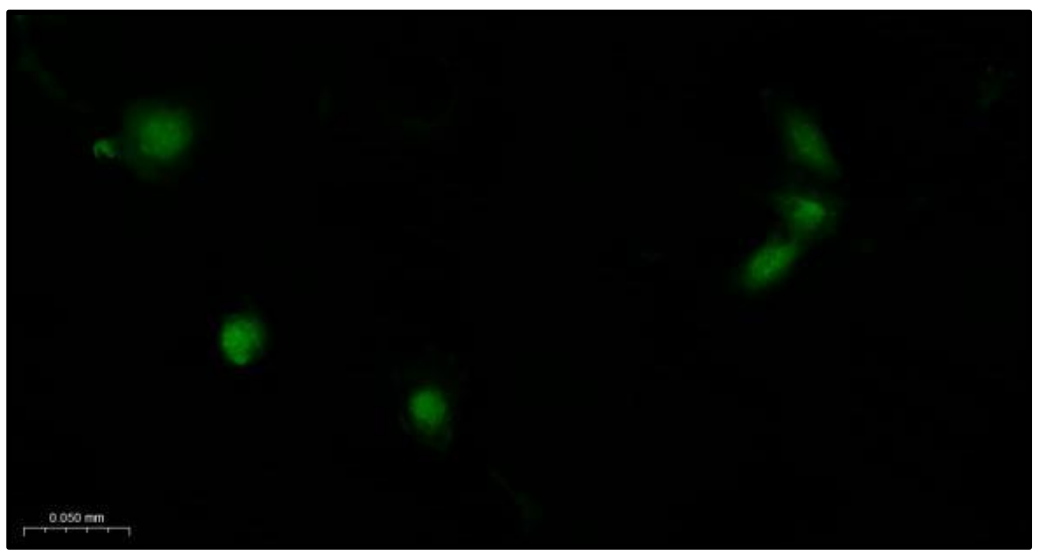

Merge

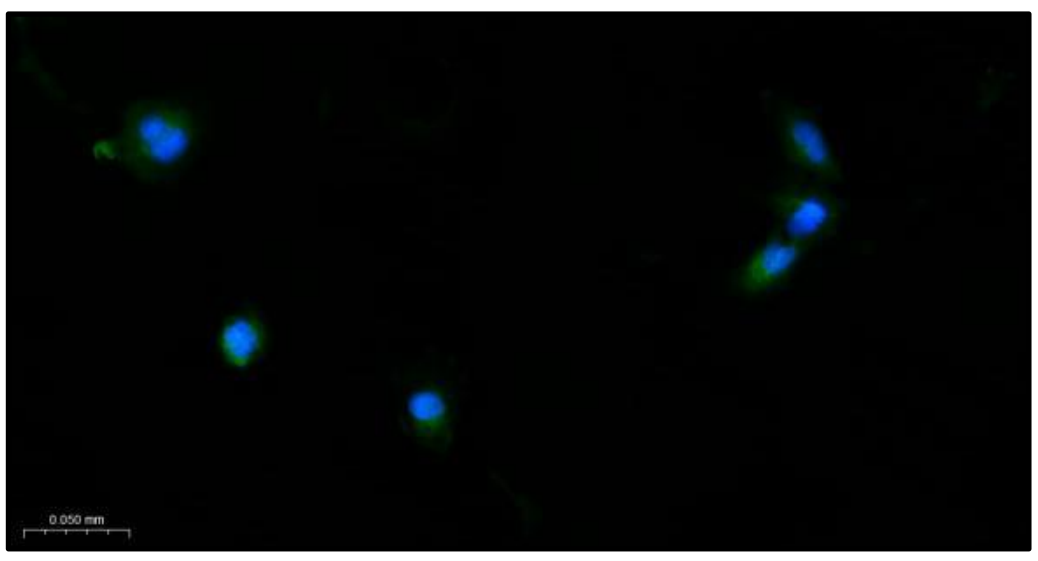

ShPLOD1

DAPI

DCFH-DA

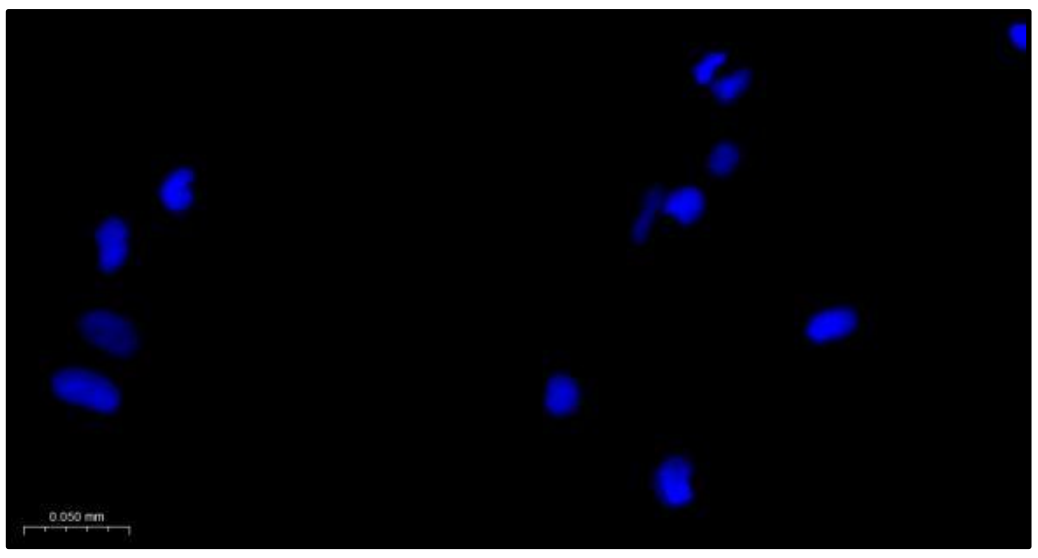
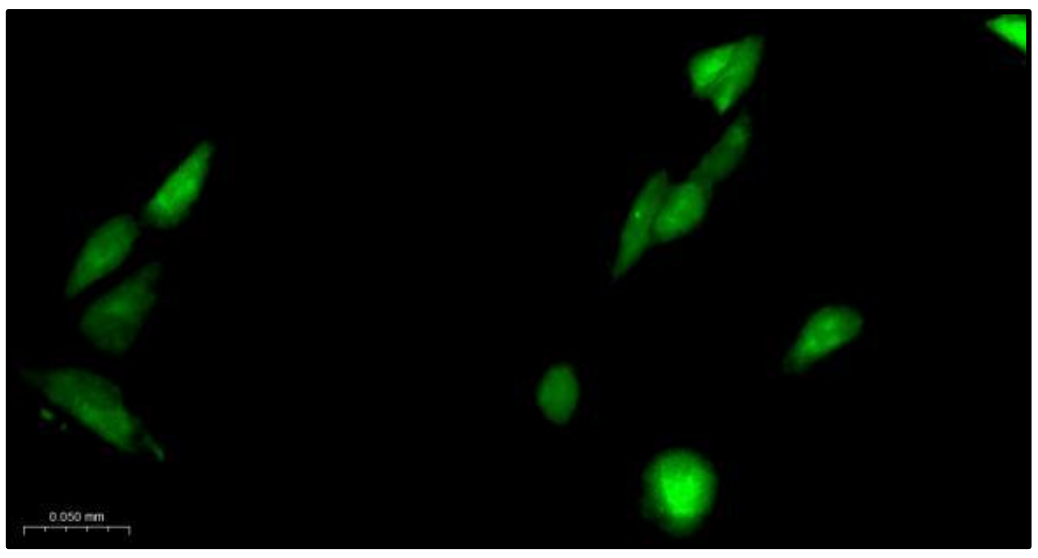

Merge

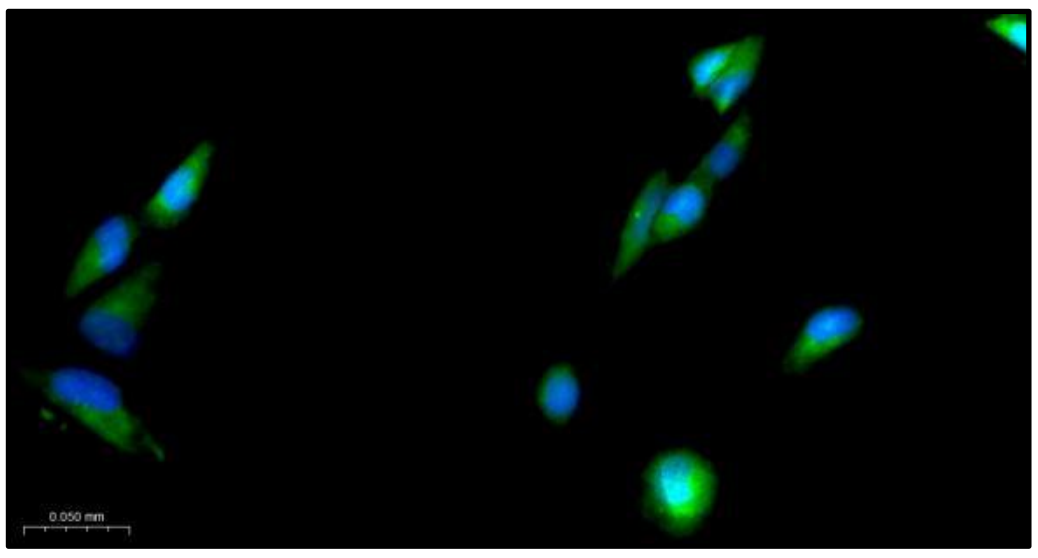

Figure S7A

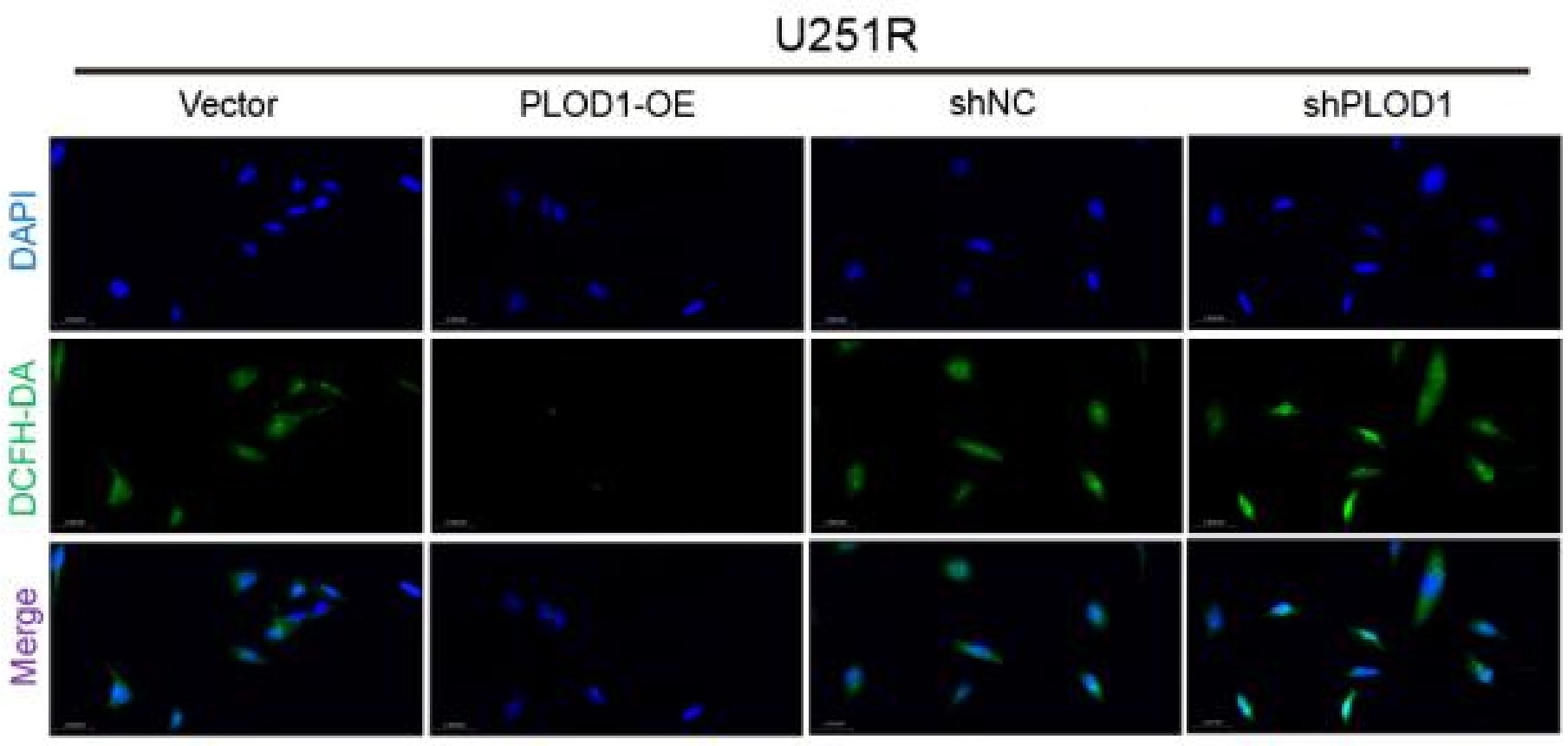

U251R

Vector

DAPI

DCFH-DA

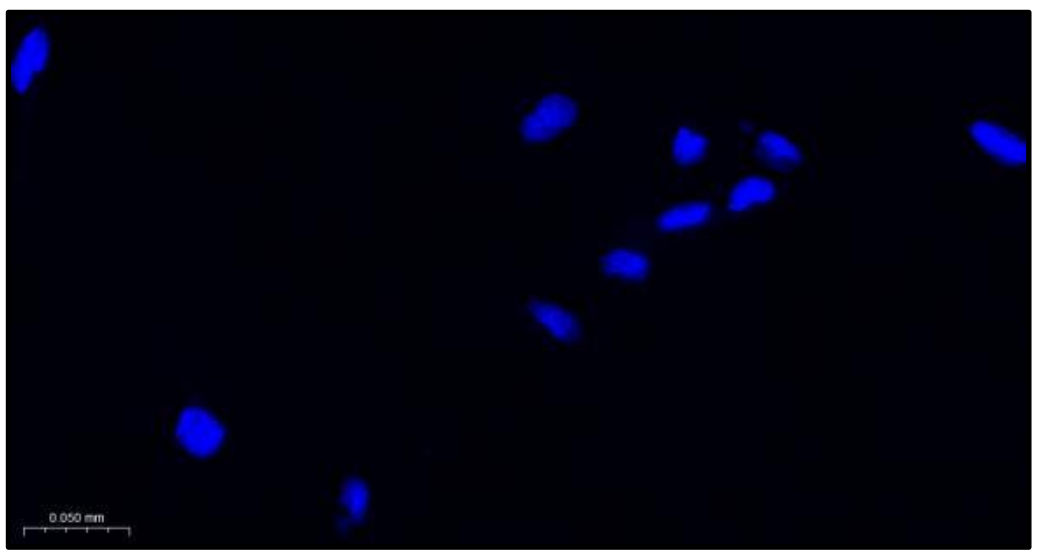
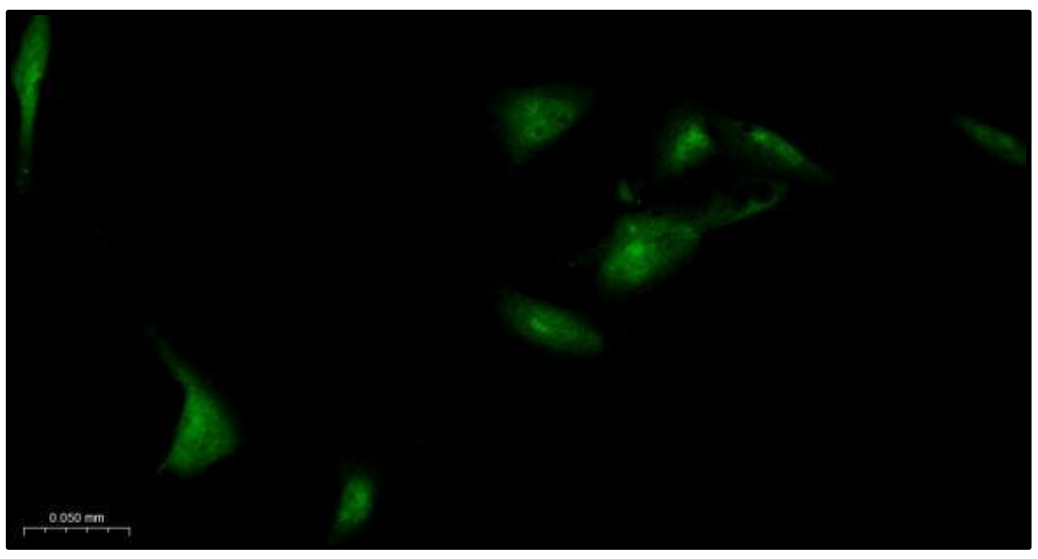

Merge

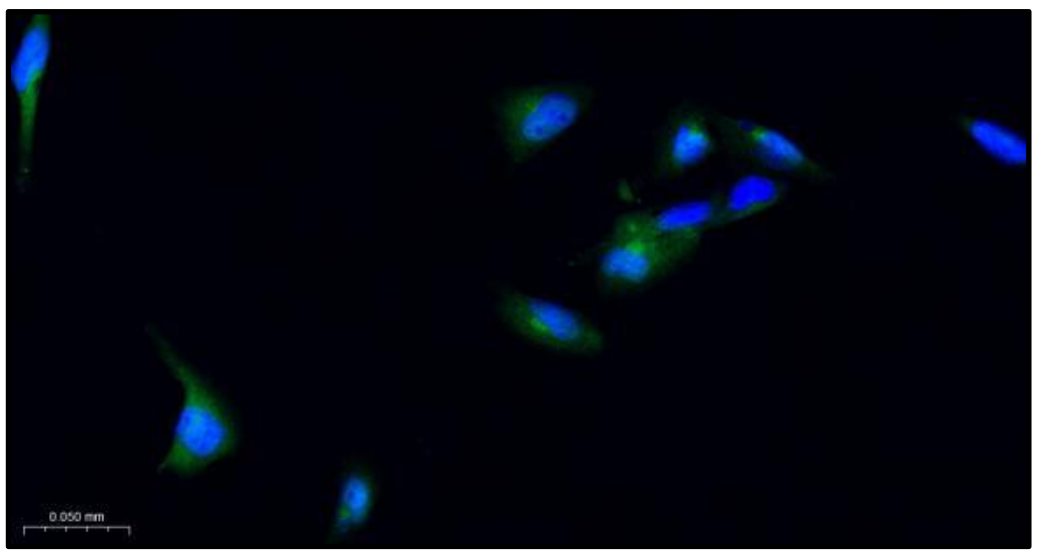

PLOD1-OE

DAPI

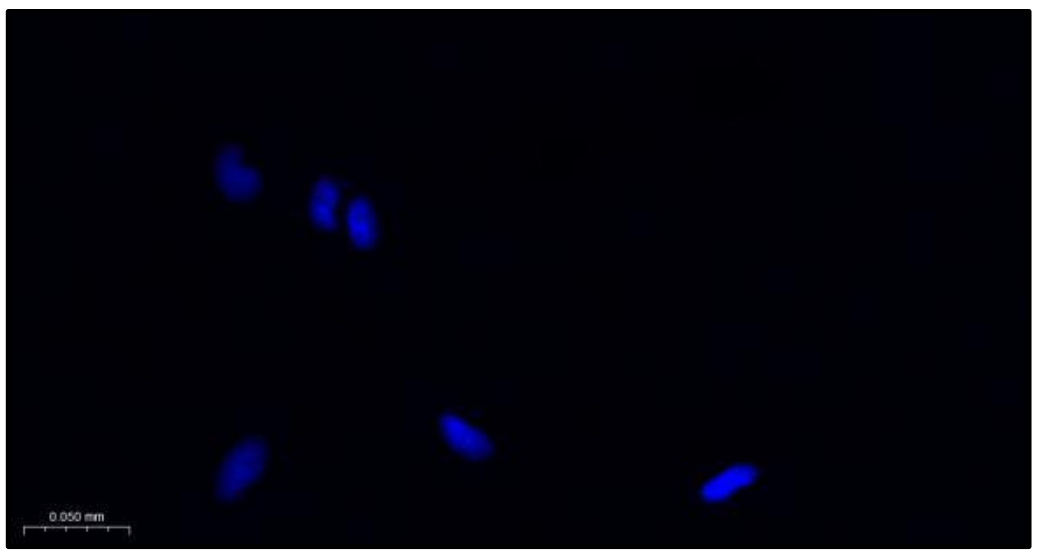

DCFH-DA

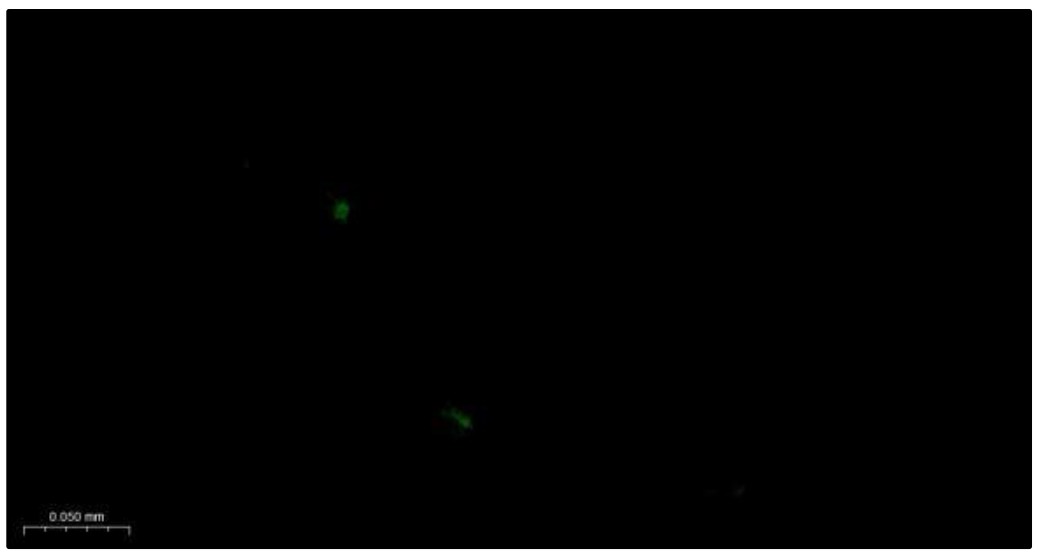

Merge

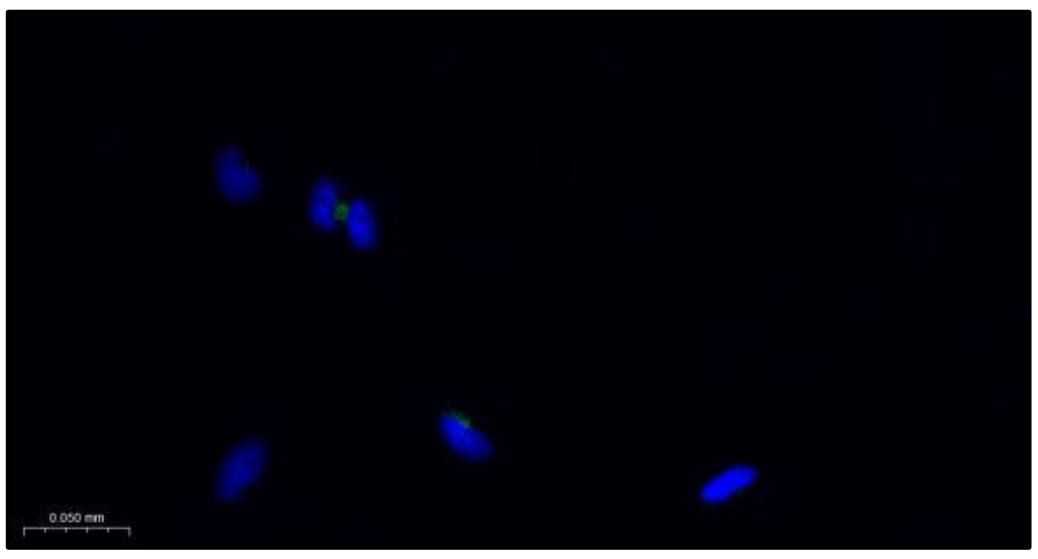

ShNC

DAPI

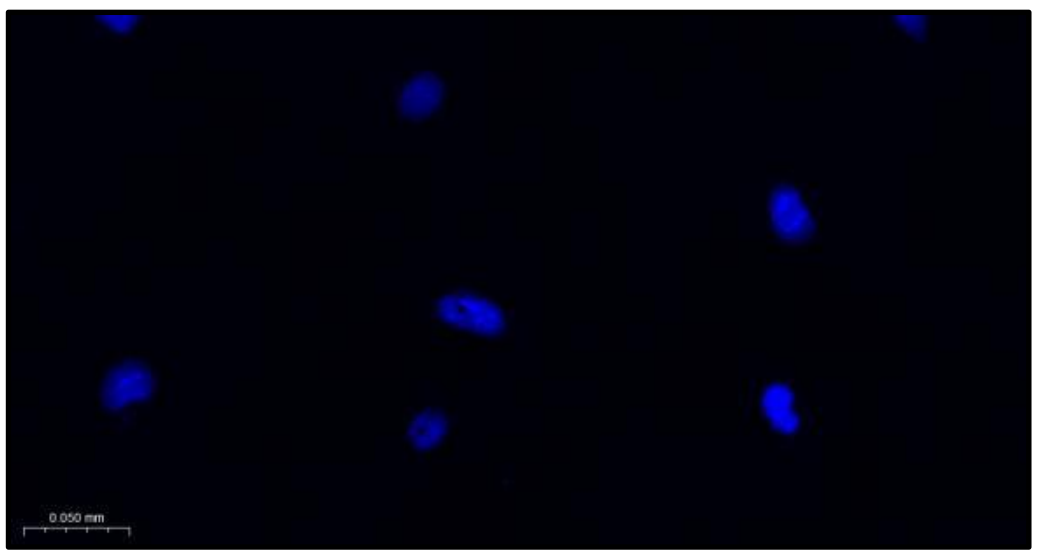

DCFH-DA

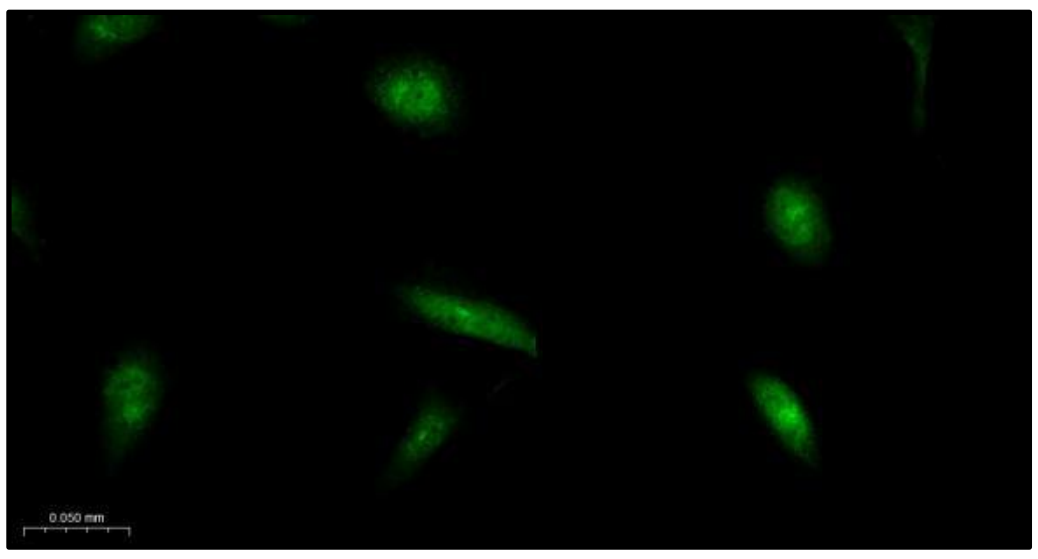

Merge

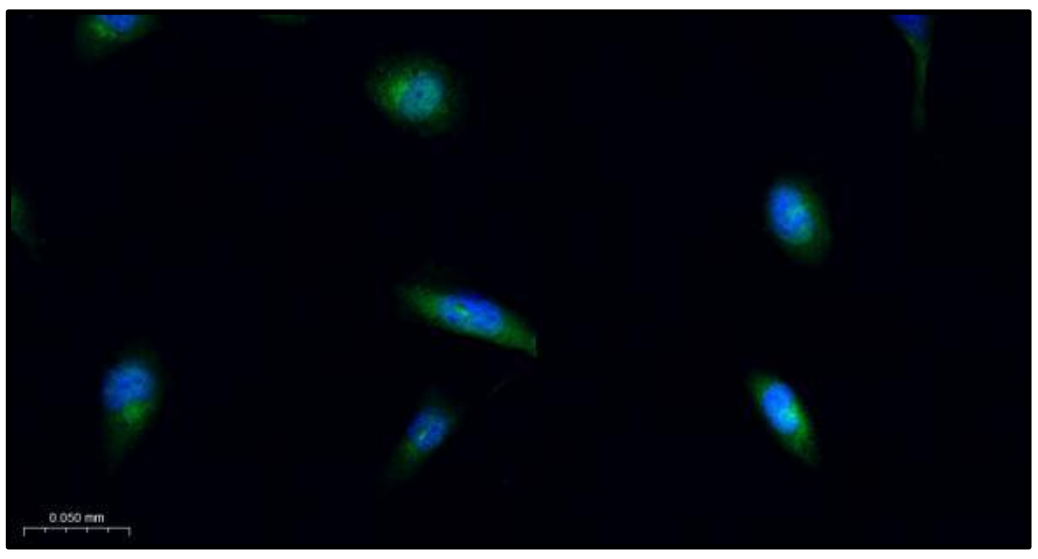

ShPLOD1

DAPI

DCFH-DA

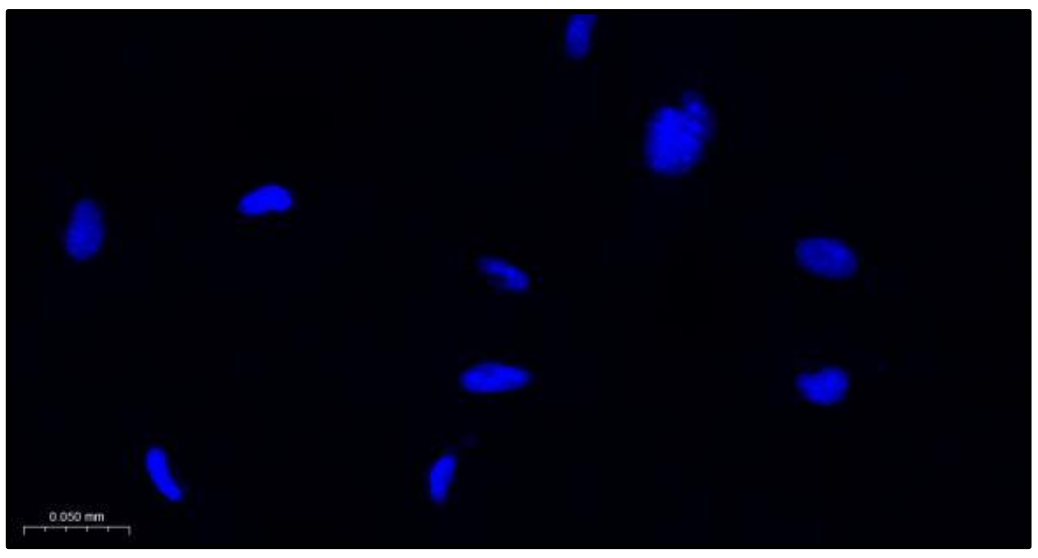

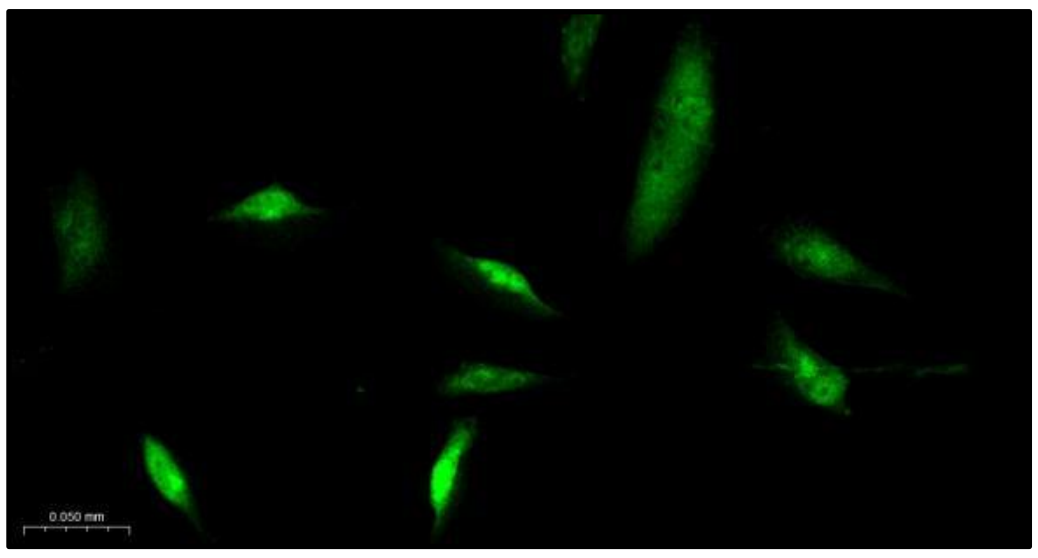

Merge

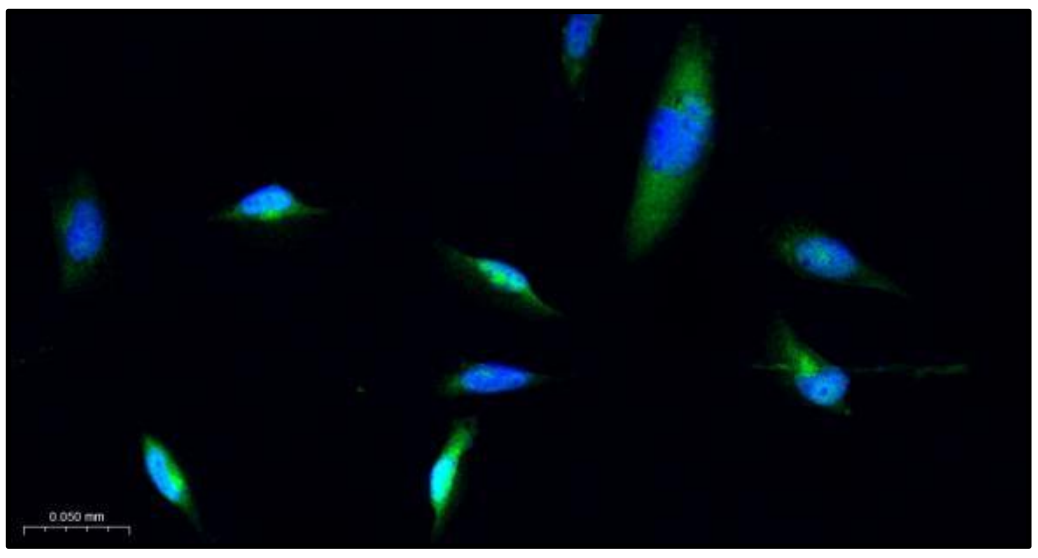

Figure S7B

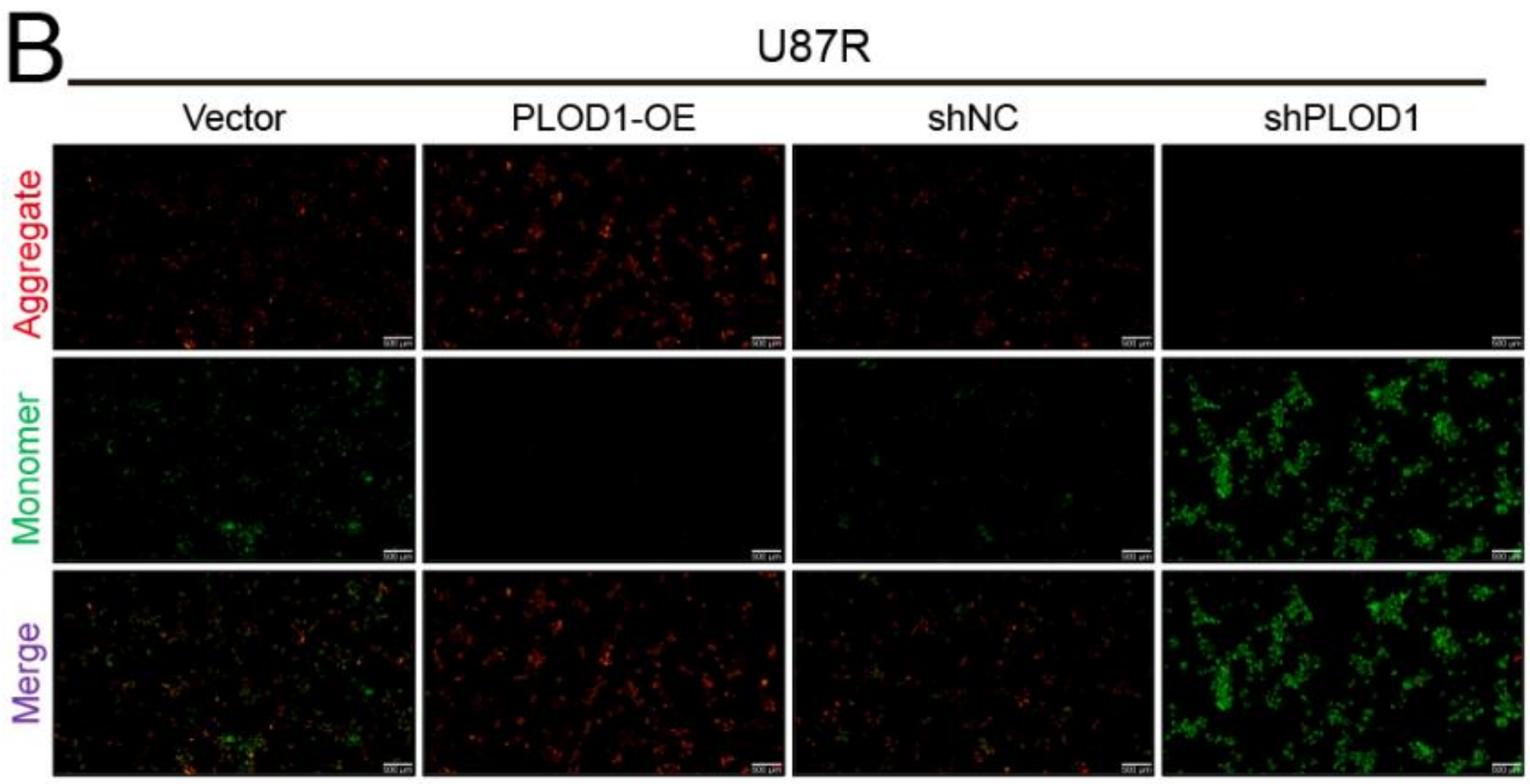

U87R

Vector

Aggregate

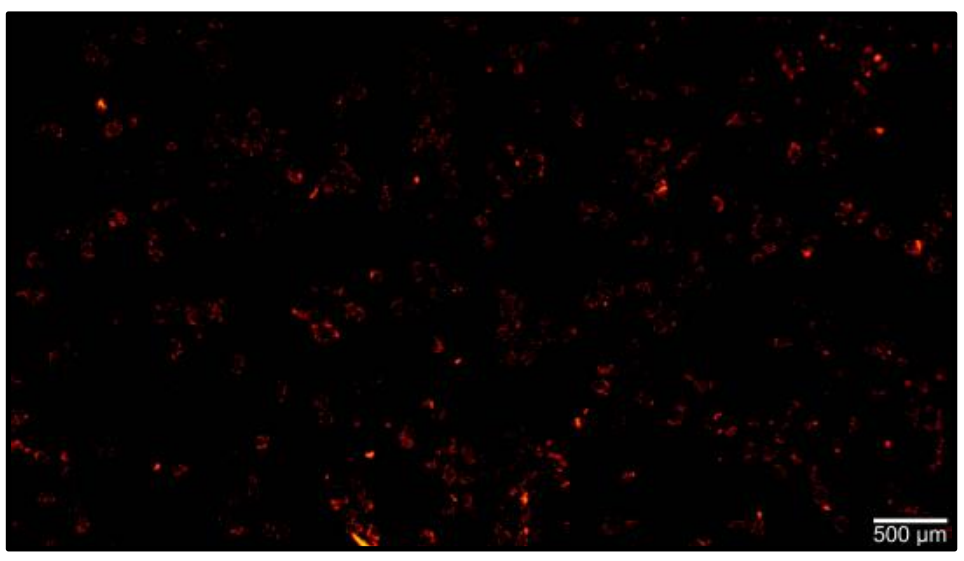

Monomers

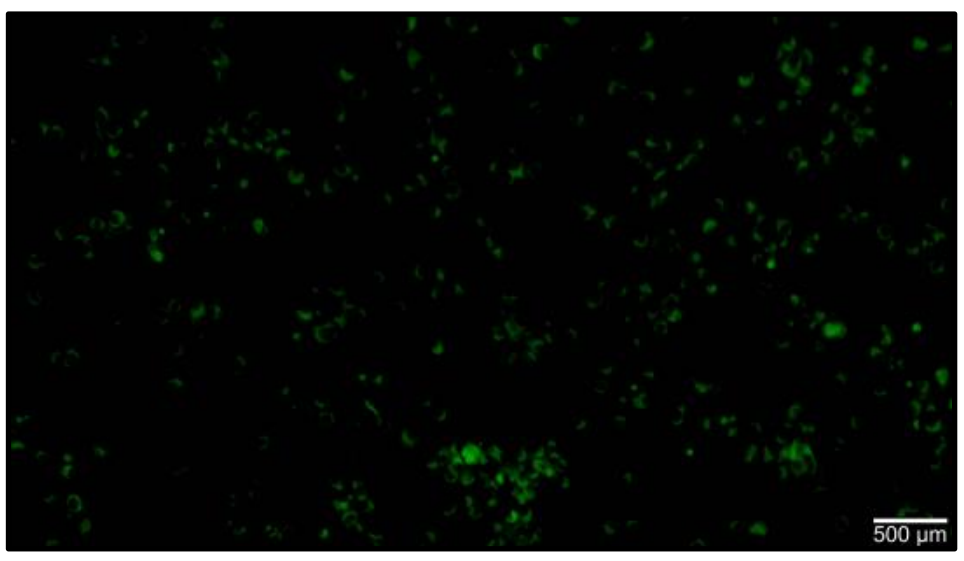

Merge

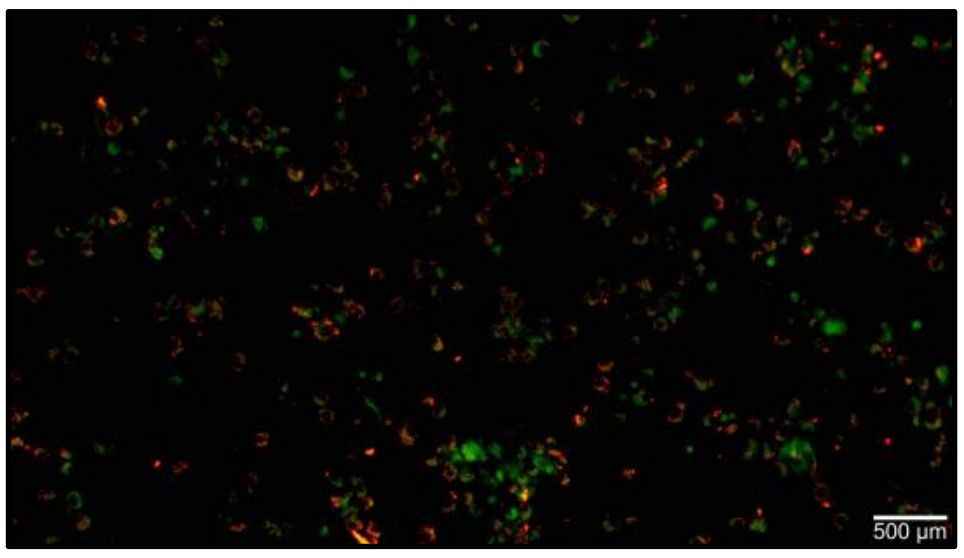

PLOD1-OE

Aggregate Monomers

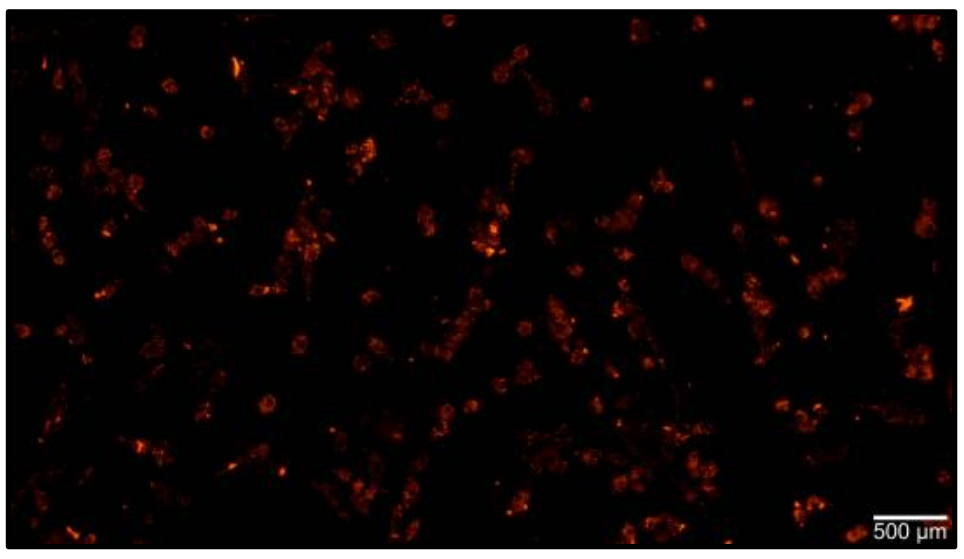

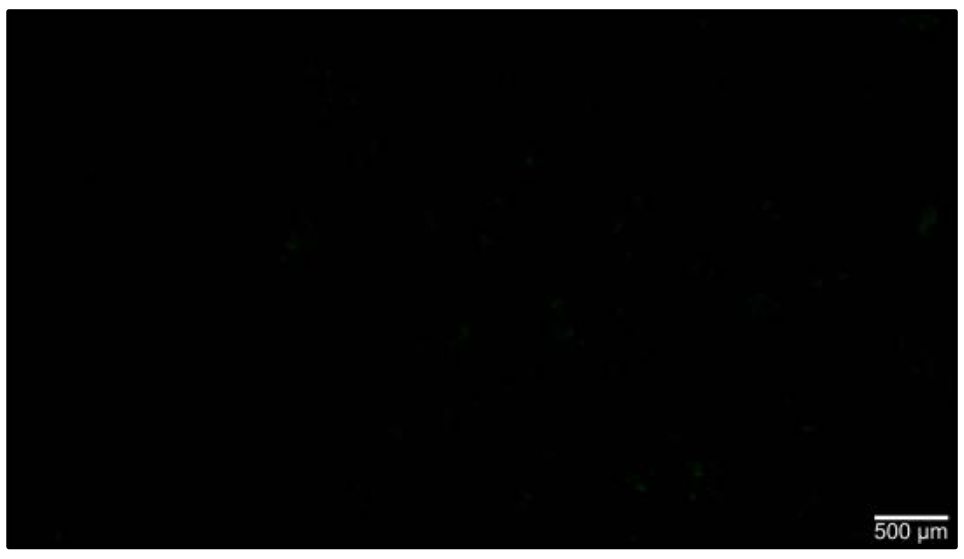

Merge

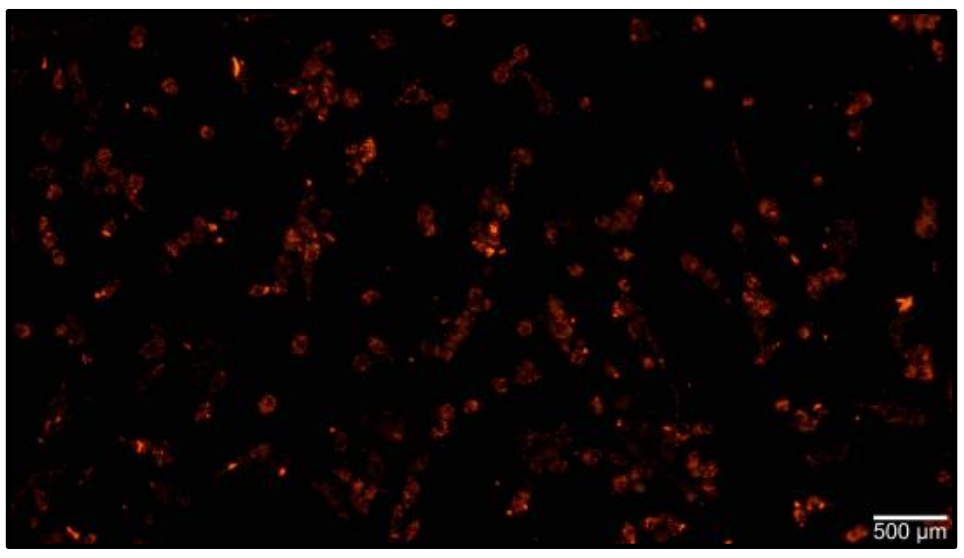

ShNC

Aggregate Monomers

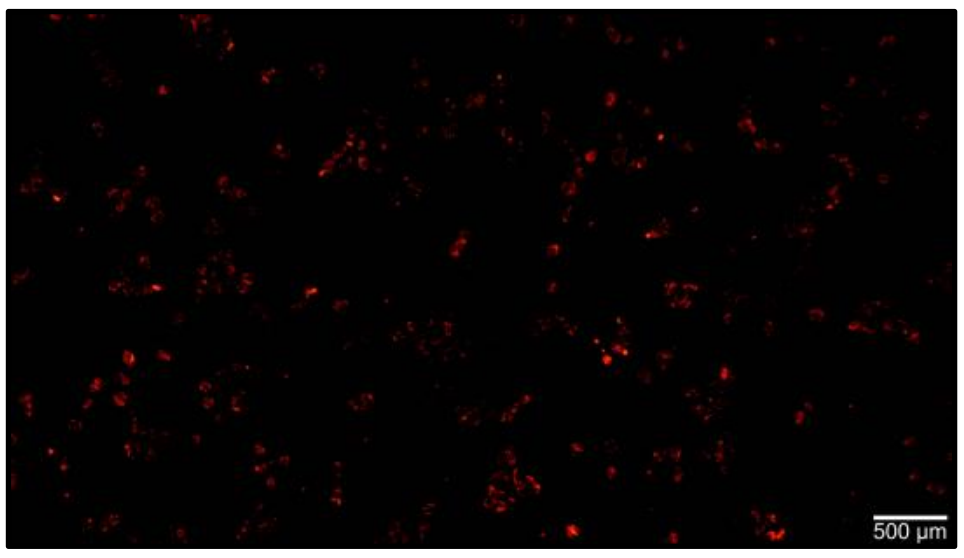

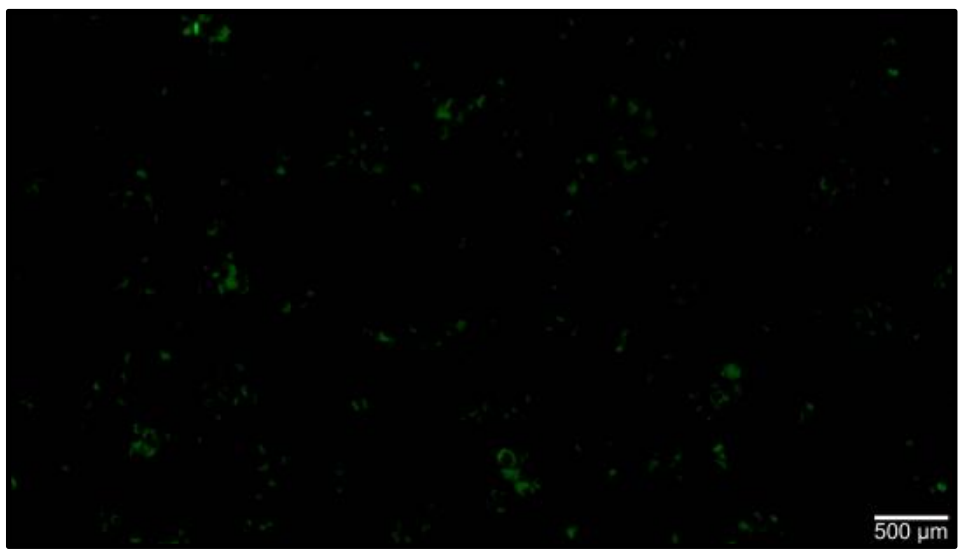

Merge

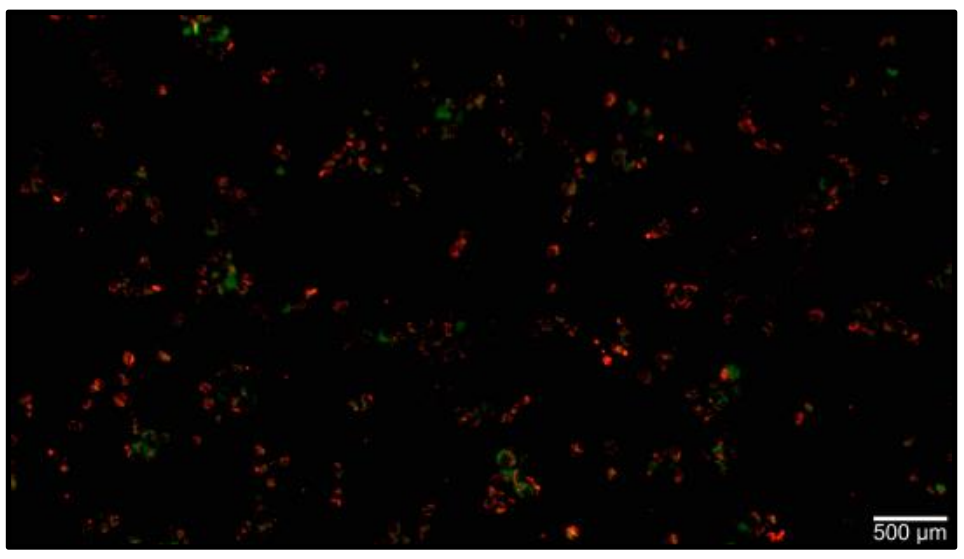

ShPLOD1

Aggregate Monomers

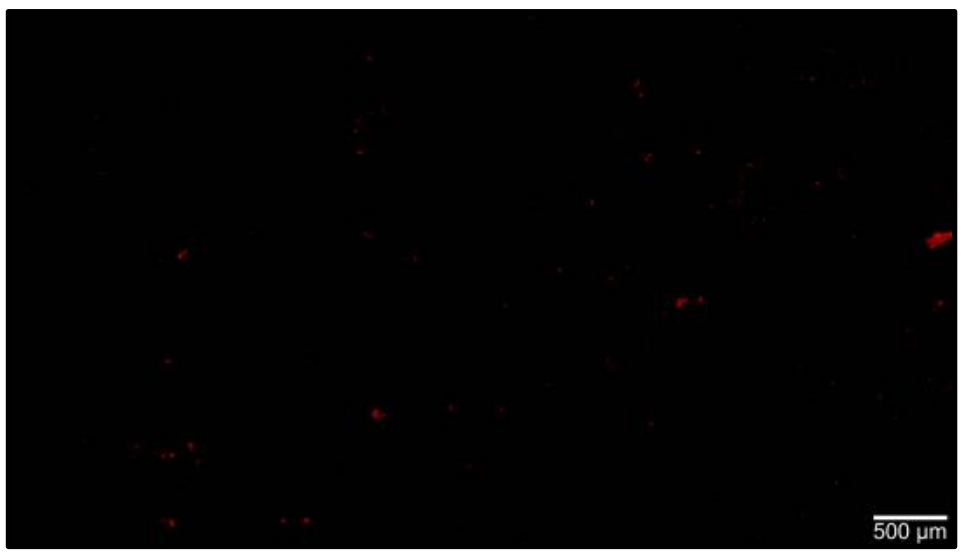

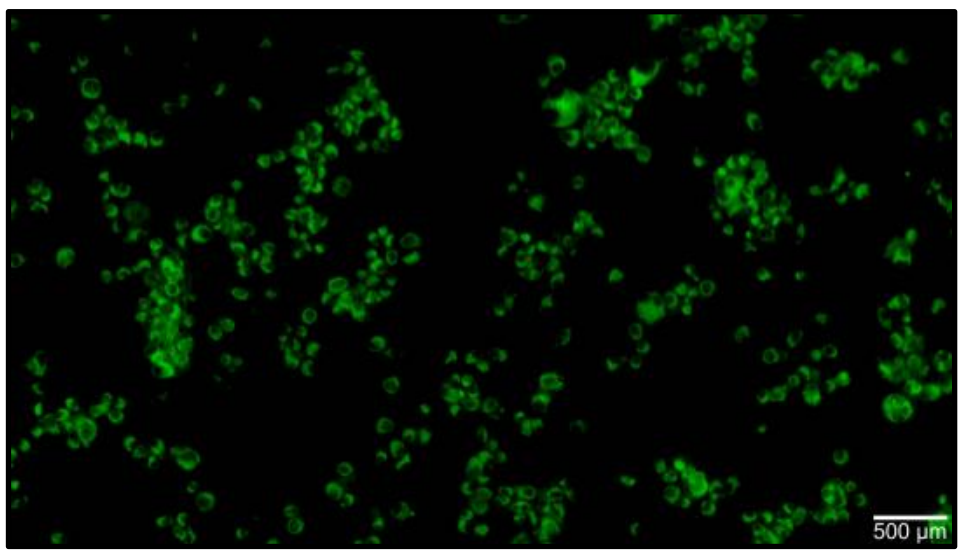

Merge

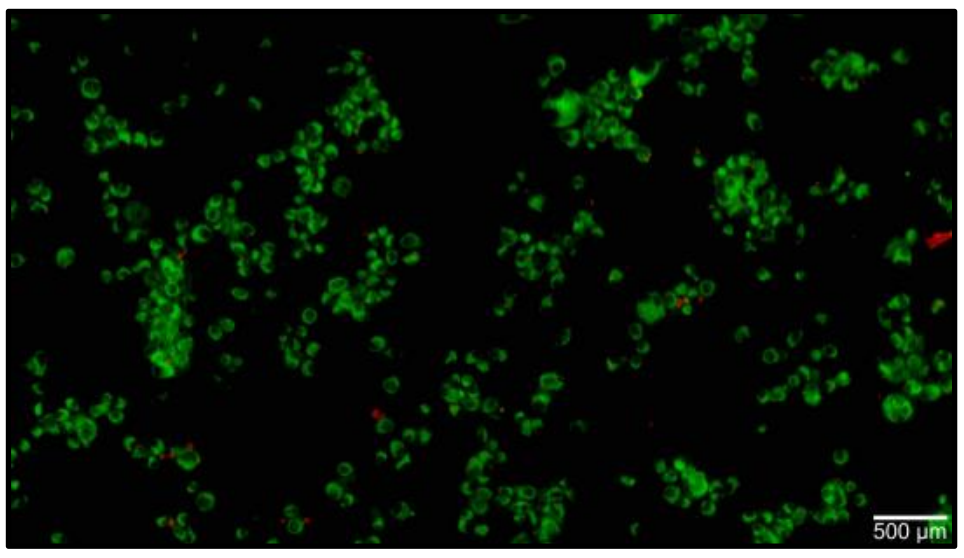

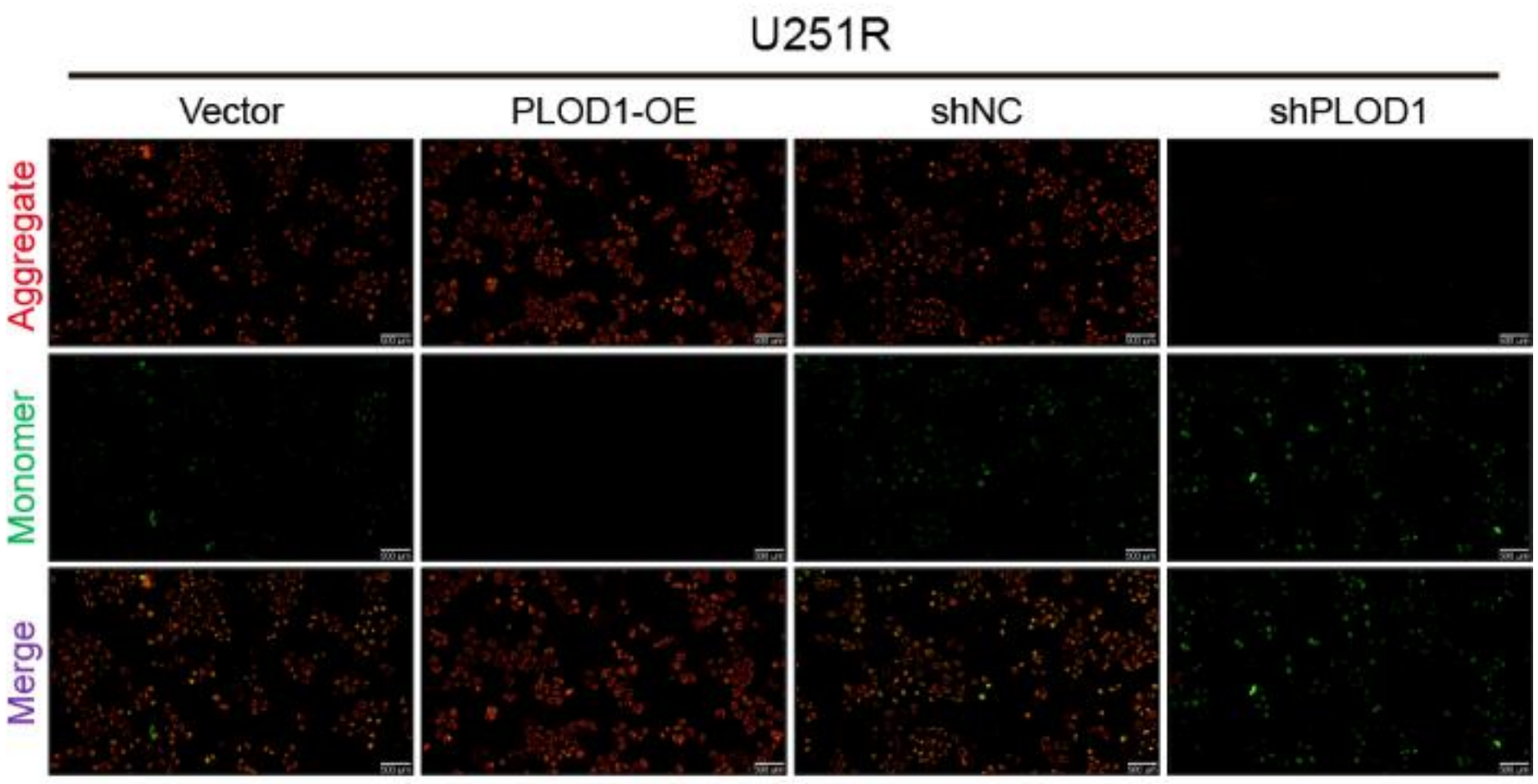

U251R

Vector

Aggregate

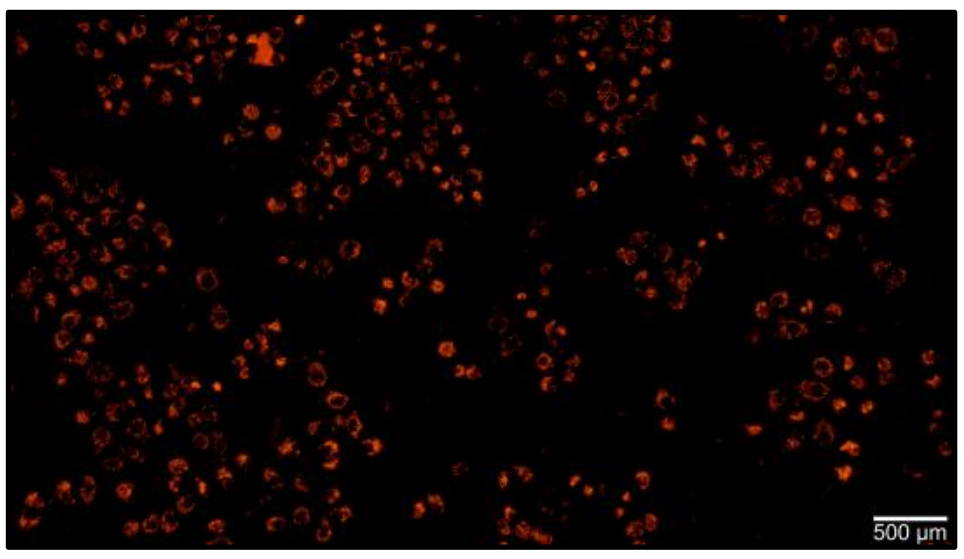

Monomers

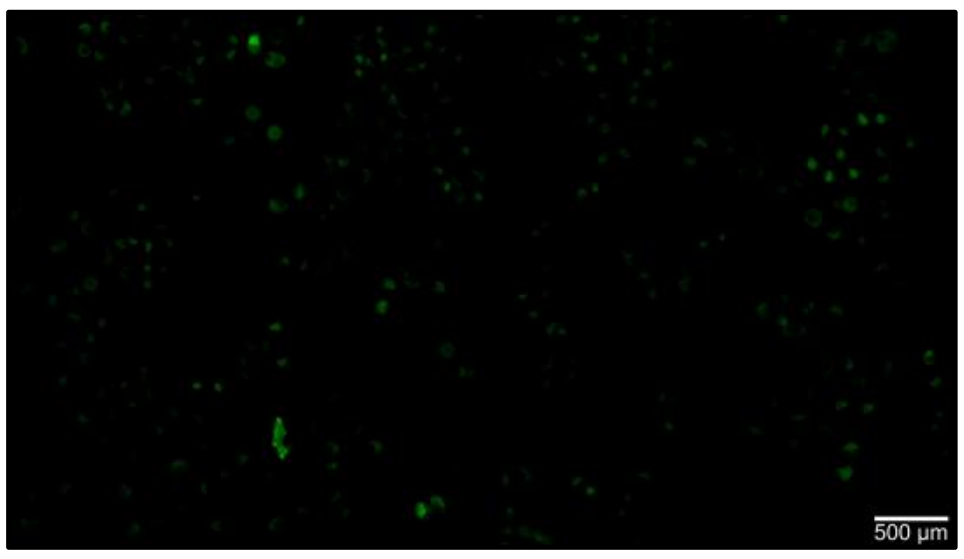

Merge

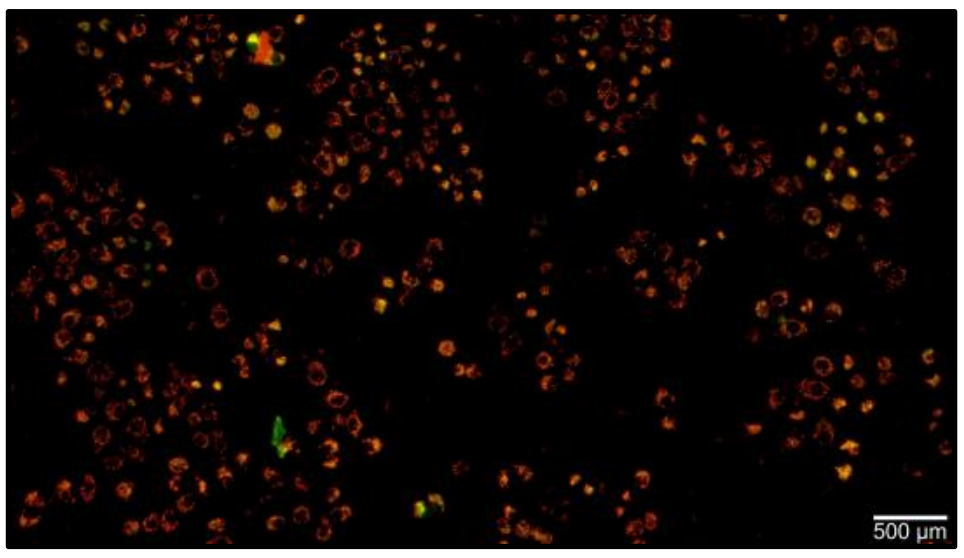

PLOD1-OE

Aggregate

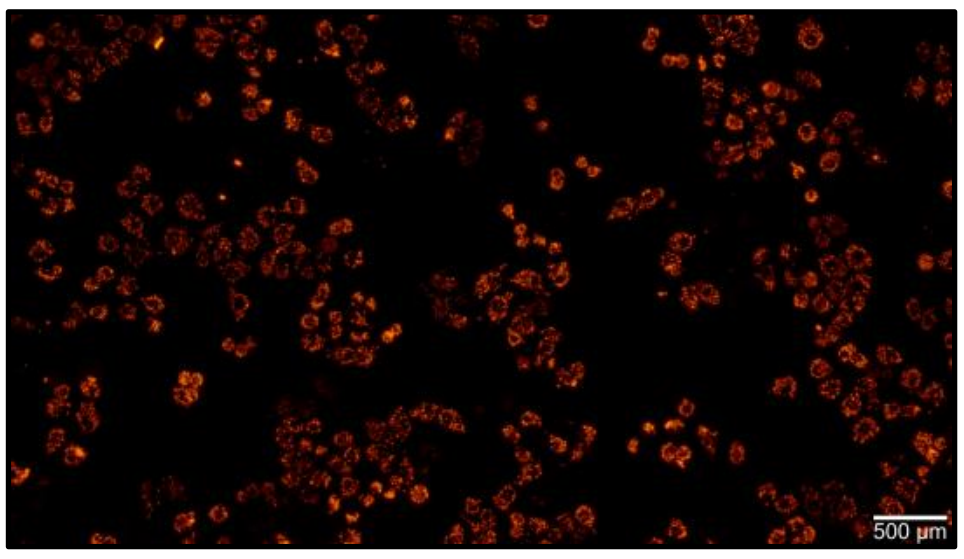

Monomers

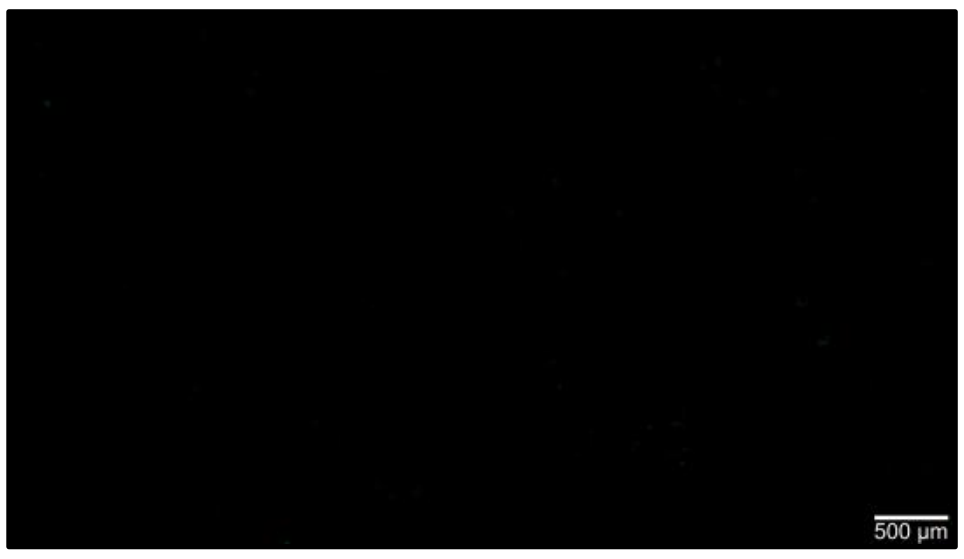

Merge

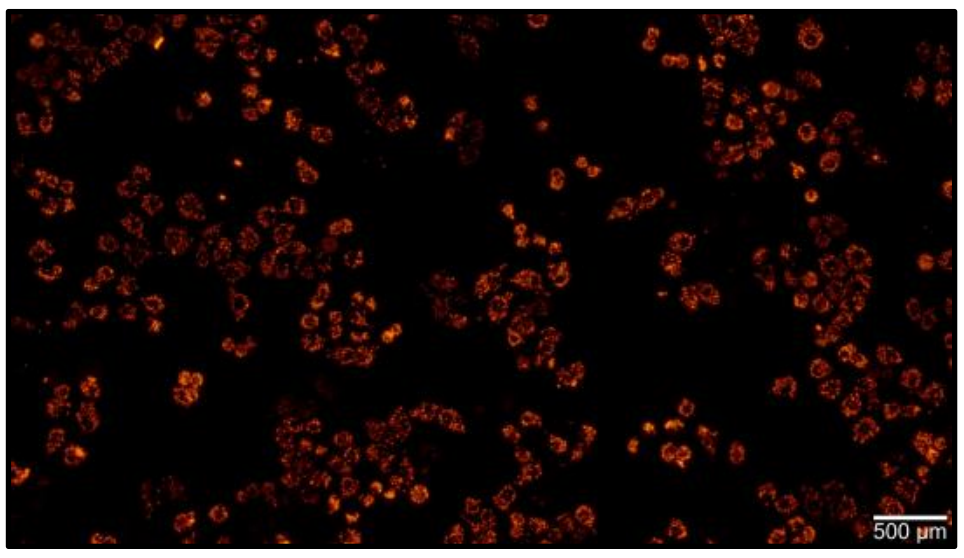

ShNC

Aggregate

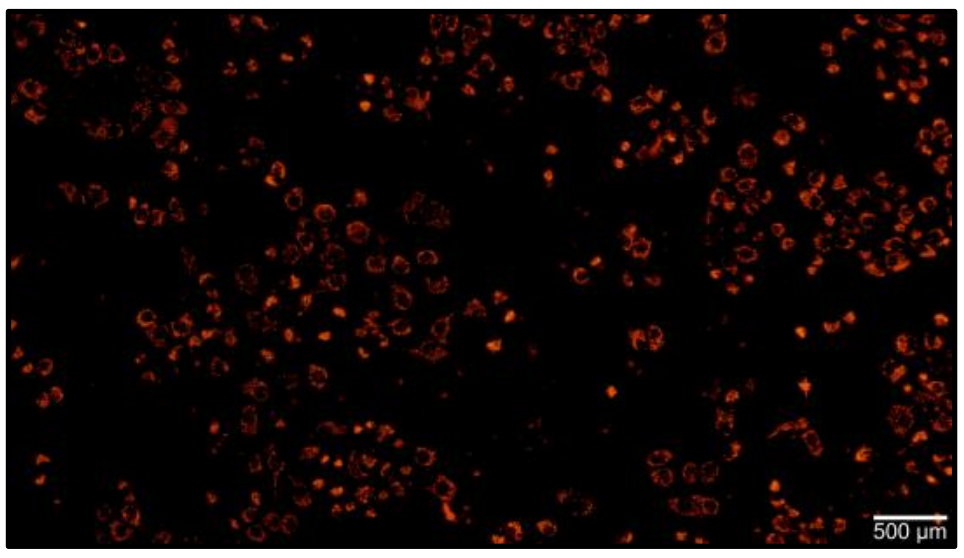

Monomers

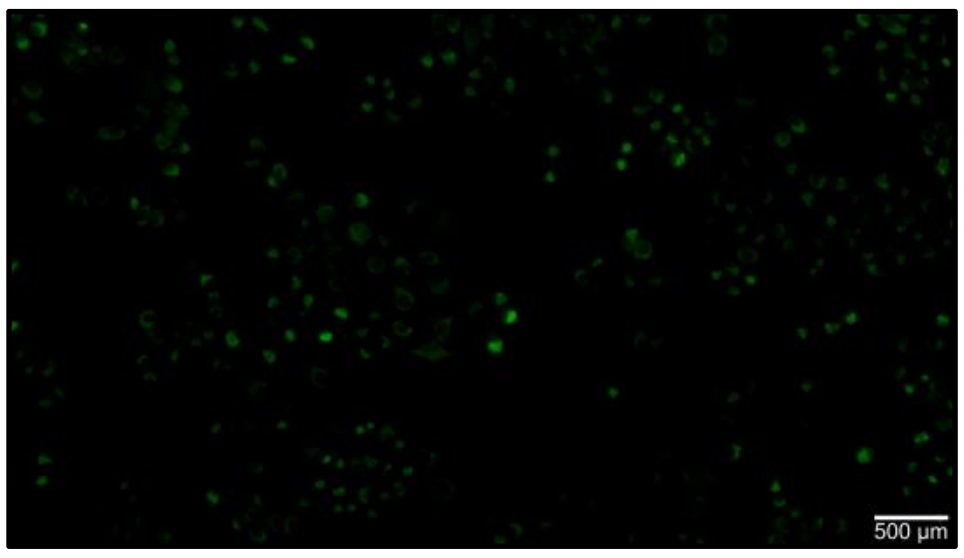

Merge

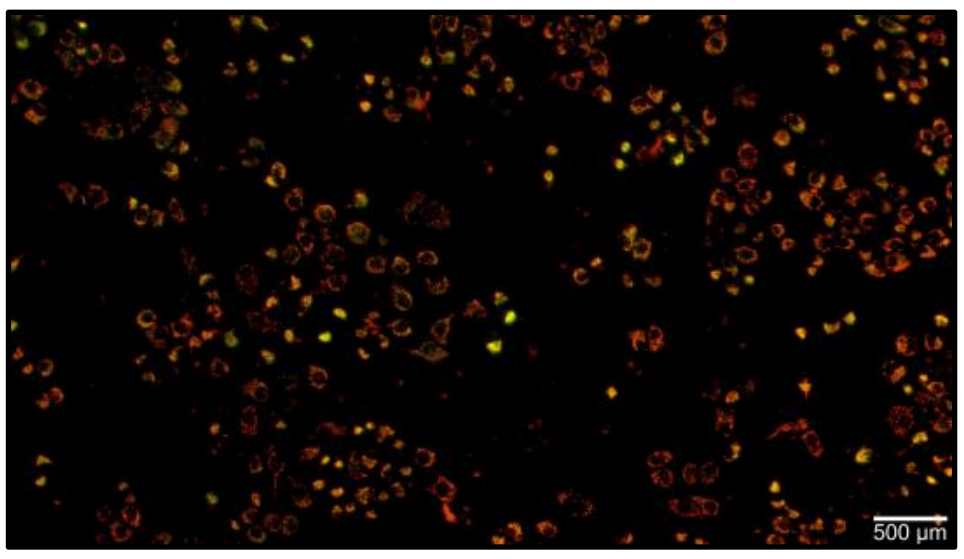

ShPLOD1

Aggregate

Monomers

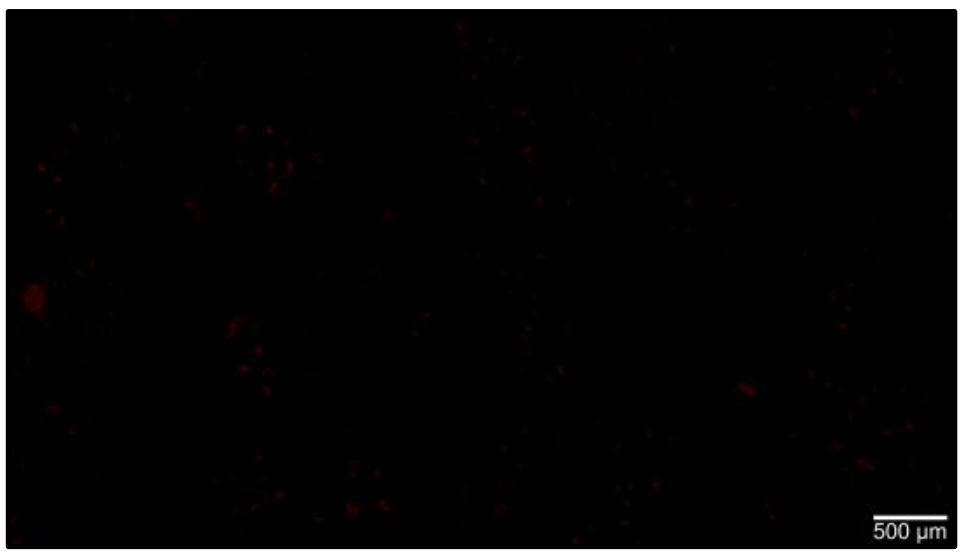

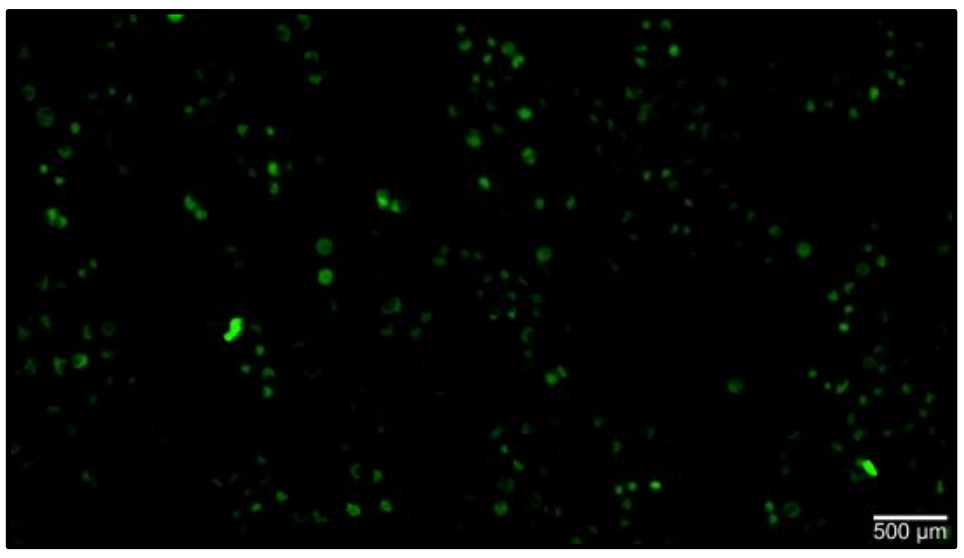

Merge

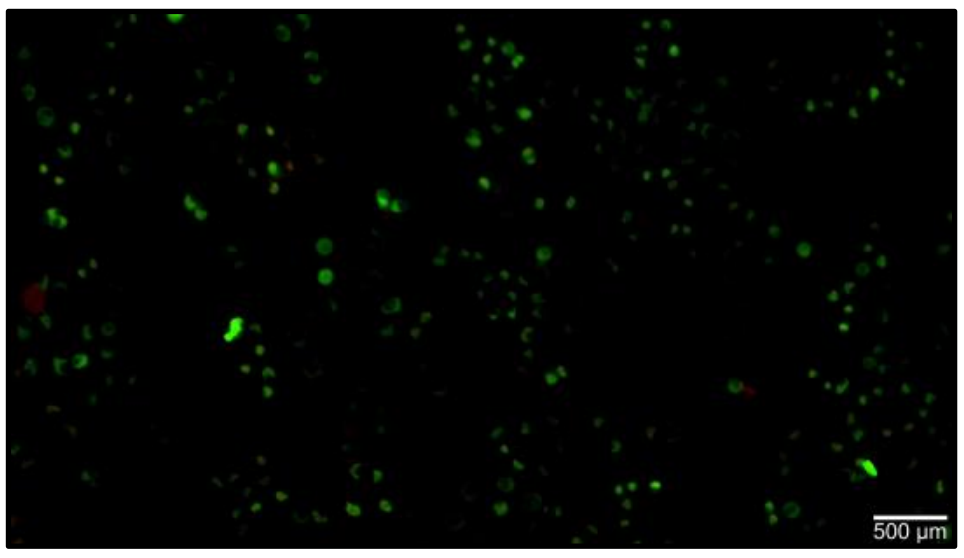

Supplement: Supplementary file 2 [file ADVS-9999-e78070-s004.pdf]
