# Supplementary material for: PLOD1 Catalytic Activity Stabilizes ENO1 by Limiting FBXW7‐dependent Degradation to Promote Glycolysis and TMZ Resistance in Glioblastoma
Source: Adv Sci (Weinh). 2026 Sep 27:e78070. Online ahead of print. doi: 10.1002/advs.78070 (PMC13616334; doi:10.1002/advs.78070)
Supplement: Supplementary file 3 [file ADVS-9999-e78070-s001.docx]

**Supplementary Figures and Figure legends**

**PLOD1 Catalytic Activity Stabilizes ENO1 by Limiting FBXW7-dependent Degradation to Promote Glycolysis and TMZ Resistance in Glioblastoma**

Fen Xue^1,2,3,4#^, Dingkong Liang^5#^, Xin Chen^6#^, Fei Fan^7*^, Xiayun He^1,2,3,4*^

^#^Fen Xue, Dingkong Liang and Xin Chen contributed equally to this work.

*Corresponding authors

**Affiliations:**

^1^ Department of Radiation Oncology, Fudan University Shanghai Cancer Center, Shanghai 200032, China

^2^ Department of Oncology, Shanghai Medical College, Fudan University, Shanghai 200032, China

^3^ Shanghai Clinical Research Center for Radiation Oncology

^4^ Shanghai Key Laboratory of Radiation Oncology, Shanghai 200032, China

^5^ Department of General Surgery, Longhua Hospital Shanghai University of Traditional Chinese Medicine, Shanghai 200032, China

^6^ Department of Neurosurgery, Fudan University Shanghai Cancer Center, Shanghai 200032, China.

^7^ Department of Neurosurgery, Union Hospital, Tongji Medical College, Huazhong University of Science and Technology, Wuhan 430000, China

**Corresponding Authors:**

Xiayun He — Email: hexiayun1962@163.com

Fei Fan — Email: fanfei@hust.edu.cn

**Keywords: Glioblastoma; Temozolomide resistance; PLOD1; ENO1; Metabolic reprogramming**

**Supplementary Figure S1. Validation of genetic manipulations.** (A, B) qRT-PCR (A) and Western blot (B) analysis confirming the successful overexpression of PLOD1 in parental U87 and U251 cells. (C, D) qRT-PCR (C) and Western blot (D) analysis demonstrating efficient knockdown of PLOD1 by two independent shRNAs in TMZ-resistant U87-R and U251-R cells. (E, F) qRT-PCR (E) and Western blot (F) analysis validating the knockdown efficiency of two independent shRNAs targeting FBXW7 in TMZ-resistant cells. (G) Western blot confirming overexpression of ENO1 in TMZ-resistant cells. (H) Western blot confirming overexpression of FBXW7 in TMZ-resistant cells. For all qRT-PCR data, results are presented as mean ± SD from three independent experiments (n = 3). Statistical significance in (A, C, and E) was determined using one-way ANOVA followed by Tukey’s multiple-comparisons test. All statistical tests were two-sided. ***P < 0.01*, and ****P < 0.001*; ns indicates no statistically significant difference.

**Supplementary Figure S2. PLOD1 orchestrates a malignant phenotype in GBM cells.** (A, B) Proliferation of parental U87 and U251 cells following PLOD1 overexpression (A) and of TMZ-resistant U87-R and U251-R cells following PLOD1 knockdown (B), as measured by CCK-8 assay over five days. (C, D) Transwell migration assays of parental cells overexpressing PLOD1 (C) and resistant cells with PLOD1 knockdown (D) Representative images of migrated cells and corresponding quantification per field are shown. Scale bar = 500 μm. For quantitative in vitro experiments, data are presented as mean ± SD from three independent experiments (n = 3). Statistical significance was determined using two-way ANOVA followed by Tukey’s multiple-comparisons test for the time-course proliferation assays in (A, B), and one-way ANOVA followed by Tukey’s multiple-comparisons test for the migration assays in (C, D). All statistical tests were two-sided. **P < 0.05*, ***P < 0.01*, and ****P < 0.001*; ns indicates no statistically significant difference.

**Supplementary Figure S3. TMZ-resistant GBM cells display a hyper-glycolytic phenotype.**  (A-C) Quantification of lactate production (A), glucose consumption (B), and ATP levels (C) in parental versus resistant cells. (D, E) Real-time metabolic flux analysis showing ECAR (D) and OCR (E). Data are presented as mean ± SD from three independent experiments (n = 3). Statistical significance for the comparisons in (A–C) was determined using a two-tailed Student’s t-test. **P < 0.05*, ***P < 0.01*, and ****P < 0.001*.

**Supplementary Figure S4. Glycolytic reprogramming is required for PLOD1-mediated malignancy.** (A-C) Quantification of relative lactate production (A), glucose consumption (B), and ATP levels (C) in parental GBM cells following PLOD1 overexpression. (D, E) Real-time metabolic flux analysis showing ECAR (D) and OCR (E) in parental cells overexpressing PLOD1. (F-I) Functional reversal experiments in PLOD1-overexpressing parental cells treated with or without the glycolysis inhibitor 2-DG (5 mM). (F) qRT-PCR shows that 2-DG partly lowers PLOD1 mRNA in all groups, but PLOD1-OE cells still retain higher expression than controls. (G) Proliferation. (H) Migration. Scale bar=500 μm. (I) TMZ sensitivity, with IC_50_ values indicated. Data are presented as mean ± SD (n = 3). Statistical significance was determined using two-tailed Student’s t-test for (A–C), one-way ANOVA with Tukey’s post hoc test for (F, H), and two-way ANOVA with Tukey’s post hoc test for (G). IC50 values in (I) were calculated by nonlinear regression. **P < 0.05*, ***P < 0.01*, ****P < 0.001*.

**Supplementary Figure S5. Pharmacological activation of glycolysis rescues metabolic defects from PLOD1 knockdown.** (A-C) Quantification of lactate production (A), glucose consumption (B), and ATP levels (C) in PLOD1-knockdown resistant cells treated with or without the glucokinase activator GKA50 (10 μM). Data are presented as mean ± SD from three independent experiments (n = 3). Statistical significance was determined using one-way ANOVA followed by Tukey’s post hoc test. **P < 0.05*, ***P < 0.01*, and ****P < 0.001*; ns, not significant.

**Supplementary Figure S6. Pharmacological inhibition of glycolysis suppresses PLOD1-induced metabolic reprogramming.** (A-C) Quantification of lactate production (A), glucose consumption (B), and ATP levels (C) in PLOD1-overexpressing parental cells treated with or without the glycolysis inhibitor 2-DG (5 mM). Data are presented as mean ± SD from three independent experiments (n = 3). Statistical significance was determined using one-way ANOVA followed by Tukey’s post hoc test. *P < 0.05, **P < 0.01, and ***P < 0.001.

**Supplementary Figure S7. PLOD1 preserves redox balance and mitochondrial membrane potential in GBM cells.** (A) Representative DCFH-DA fluorescence images showing intracellular reactive oxygen species (ROS) levels in U87R and U251R cells following PLOD1 overexpression or knockdown. PLOD1 overexpression reduced intracellular ROS accumulation, whereas PLOD1 depletion increased ROS levels. Scale bar, 50 μm.(B) Representative JC-1 staining images and quantitative analysis of JC-1 aggregate (red) and monomer (green) fluorescence in U87R and U251R cells. PLOD1 overexpression increased the proportion of JC-1 aggregates and reduced JC-1 monomers, whereas PLOD1 knockdown produced the opposite effect, indicating mitochondrial membrane depolarization. Scale bar, 500 μm. Data are presented as mean ± SD (n = 3). Statistical significance in (B) was determined using one-way ANOVA followed by Tukey’s post hoc test. **P < 0.05*, ***P < 0.01*, ****P < 0.001*.

**Supplementary Figure S8. LC-MS/MS screening and structural prioritization of candidate PLOD1-regulated lysine residues on ENO1.** (A) LC-MS/MS summary showing successful identification of ENO1 in immunoprecipitated samples from control (NC) and PLOD1-overexpressing (PLOD1 OE) cells. ENO1 peptide groups, peptide-spectrum matches (PSMs), and sequence coverage are presented. (B) Quantification of lysine oxidation-containing peptide groups detected in the LC-MS/MS datasets. Although lysine oxidation events were detected globally, no ENO1-derived lysine oxidation peptide was confidently identified. (C) Sequence coverage of lysine residues across ENO1 in NC and PLOD1 OE samples. Covered lysine residues are indicated, whereas no confidently localized lysine oxidation site was detected. (D) Structural mapping of candidate lysine residues on the ENO1 crystal structure based on sequence motif prediction and structural accessibility analysis. Canonical PLOD-like recognition sites (K28, K60, and K126), extended candidate residues, and the previously reported K281 post-translational modification hotspot are highlighted.

**Supplementary Figure S9. The association between PLOD1 and ENO1 is independent of clinicopathological variables and is maintained across WHO grade strata.**

(A) Rank-based partial Spearman correlation analyses demonstrating that the positive association between PLOD1 and ENO1 expression remains significant after adjustment for WHO grade or multivariable clinical covariates in the TCGA-GBMLGG, CGGA mRNAseq_693, and CGGA mRNAseq_325 cohorts. Partial correlation coefficients and corresponding P values are indicated.

(B) Multivariable linear regression analyses showing that PLOD1 expression remains independently associated with ENO1 expression after adjustment for clinicopathological variables. Standardized regression coefficients (β) and corresponding P values are shown. (C) Correlation analyses of PLOD1 and ENO1 mRNA expression within individual WHO grade II, III, and IV gliomas from the TCGA-GBMLGG, CGGA mRNAseq_693, and CGGA mRNAseq_325 cohorts. Spearman correlation coefficients (rho), sample sizes (n), and corresponding P values are indicated. (D) Multivariable Cox proportional hazards regression analysis evaluating the independent prognostic value of the PLOD1–ENO1 signature in the TCGA-GBMLGG cohort. The model simultaneously included the PLOD1–ENO1 signature, age, sex, and WHO grade. Hazard ratios (HRs), 95% confidence intervals (CIs), and corresponding P values are shown.

**Supplementary Figure S10. Body-weight changes of mice during the subcutaneous xenograft experiments.**
(A) Body-weight changes in mice from the Vector, PLOD1-OE, Vector+TMZ, and PLOD1-OE+TMZ groups during the experimental period (n = 5). (B) Body-weight changes in mice from the shNC+TMZ and shPLOD1+TMZ groups during the experimental period (n = 6). Data are presented as mean ± SD. Statistical analysis was performed using two-way repeated-measures ANOVA followed by Tukey’s multiple-comparisons test. No significant differences in body weight were observed among the experimental groups (P > 0.05).
