# Supplementary material for: PLOD1 Catalytic Activity Stabilizes ENO1 by Limiting FBXW7‐dependent Degradation to Promote Glycolysis and TMZ Resistance in Glioblastoma
Source: Adv Sci (Weinh). 2026 Sep 27:e78070. Online ahead of print. doi: 10.1002/advs.78070 (PMC13616334; doi:10.1002/advs.78070)
Supplement: Supplementary file 4 [file ADVS-9999-e78070-s003.docx]

**Supplementary Table**

Fen Xue^1,2,3,4#^, Dingkong Liang^5#^, Xin Chen^6#^, Fei Fan^7*^, Xiayun He^1,2,3,4*^

^#^Fen Xue, Dingkong Liang and Xin Chen contributed equally to this work.

*Corresponding authors

**Affiliations:**

^1^ Department of Radiation Oncology, Fudan University Shanghai Cancer Center, Shanghai 200032, China

^2^ Department of Oncology, Shanghai Medical College, Fudan University, Shanghai 200032, China

^3^ Shanghai Clinical Research Center for Radiation Oncology

^4^ Shanghai Key Laboratory of Radiation Oncology, Shanghai 200032, China

^5^ Department of General Surgery, Longhua Hospital Shanghai University of Traditional Chinese Medicine, Shanghai 200032, China

^6^ Department of Neurosurgery, Fudan University Shanghai Cancer Center, Shanghai 200032, China.

^7^ Department of Neurosurgery, Union Hospital, Tongji Medical College, Huazhong University of Science and Technology, Wuhan 430000, China

**Corresponding Authors:**

Fei Fan — Email: fanfei@hust.edu.cn

Xiayun He — Email: hexiayun1962@163.com

**Keywords: Glioblastoma; Temozolomide resistance; PLOD1; ENO1; Metabolic reprogramming**

Table S1. List of antibodies used in this study.

| Antibody Target | Source (Vendor) | Catalog No. | Species | Application (Dilution) |
| --- | --- | --- | --- | --- |
| **Primary Antibodies** |  |  |  |  |
| ENO1 | Cell Signaling Technology (CST) | #3810 | Rabbit | WB (1:1000), IHC(1:50) |
| ENO1 | Proteintech | 67187-1-Ig | Mouse | IF(1:400) |
| ENO1 | Abcam | Ab227978 | Rabbit | IP(3ug) |
| PLOD1 | Proteintech | 29480-1-AP | Rabbit | WB (1:3000), IF (1:200),  IHC(1:100)  IP(3ug) |
| His-tag | Cell Signaling Technology (CST) | #12698 | Rabbit | WB (1:1000) |
| Myc-tag | Cell Signaling Technology (CST) | #2276 | Mouse | WB (1:1000) |
| HA-tag | Cell Signaling Technology (CST) | #3724 | Rabbit | WB (1:1000)  IP (3ug) |
| Flag-tag | Cell Signaling Technology (CST) | #14793 | Rabbit | WB (1:1000)  IP(3ug) |
| GST-tag | Cell Signaling Technology (CST) | #2624 | Mouse | WB (1:1000) |
| HK2 | Cell Signaling Technology (CST) | #2867 | Rabbit | WB (1:1000) |
| PKM2 | Cell Signaling Technology (CST) | #4053 | Rabbit | WB (1:1000) |
| Lamin B1 | HUABIO | ET1606-27 | Rabbit | 1:5000 |
| Calnexin | proteintech | 10427-2-AP | Rabbit | 1:5000 |
| LDHA | Cell Signaling Technology (CST) | #3582 | Rabbit | WB (1:1000) |
| Ki67 | Abcam | ab16667 | Rabbit | IHC (1:500) |
| β-actin | Abcam | ab6276 | Mouse | WB (1:5000) |
| β-actin | Abcam | ab8227 | Rabbit | WB (1:5000) |
| FBXW7 | Proteintech | 28424-1-AP | Rabbit | WB (1:2000)  IP(4ug) |
| anti-Hyp antibody | MyBioSource | MBS2045108 | Rabbit | WB(1:1000) |
| **Secondary Antibodies** |  |  |  |  |
| Goat anti-Rabbit IgG (H+L)-HRP | Abcam | ab205718 | Goat | WB (1:5000) |
| Goat anti-Mouse IgG (H+L)-HRP | Abcam | ab205719 | Goat | WB (1:5000) |
| Goat anti-Mouse IgG (H+L) Highly Cross-Adsorbed Secondary Antibody, Alexa Fluor™ Plus 488 | Invitrogen | A32723TR | Goat | IF (4µg/mL) |
| Goat anti-Rabbit IgG (H+L) Cross-Adsorbed Secondary Antibody, Alexa Fluor™ 594 | Invitrogen | A-11012 | Goat | IF (4µg/mL) |

Table S2. List of Primer sequences used in this study

| Gene | Species | Forward | Reverse |
| --- | --- | --- | --- |
| PRDX6 | Human | GTTGCCACCCCAGTTGATTG | TGAAGACTCCTTTCGGGAAAAGT |
| NMNAT1 | Human | AGGAAGGTACACAGTTGTCAAAG | GATGACCCGGTGATAGGCAG |
| NUDT5 | Human | AAACAGTTCCGACCACCAATG | GTCCCCTTTGTAGCCAGTTTC |
| PLOD1 | Human | AACAGAACAAGAACGTCATT | TGTTTGAAATATAGGGCACA |
| UPP1 | Human | TCATCCGCATTGGCACTTCT | GCAGAACACAGCAACAGCTC |
| HEXB | Human | CCGGAGAACTTCTACATCAG | GGGTGATTGAGACAAGAAGT |
| PLCE1 | Human | GCTTCTTAACACGGGACTTGG | CTTCAAGGGCATTGTGCTCTC |
| SUV39H1 | Human | CATCTGGGACGCATCACTGTA | TCACCAACACGGTACTCATTG |
| LDHA | Human | TTGACCTACGTGGCTTGGAAG | GGTAACGGAATCGGGCTGAAT |
| PPAT | Human | AATTGTCAGCCCTTCGTTGTT | CCTTAATCGAGCAGCATTTACCA |
| ACOT1 | Human | GTGGGCGTCAACAGAAATCG | AACAGGAAGGTGCTCTCTGC |
| ALDH3B1 | Human | GCCCTGGAACTATCCGCTG | CGTTCTTGCTAATCTCCGATGG |
| B4GALT4 | Human | ACAATGGTCTTCCACACTAGAGA | AGACTCGTGACACTTGGTGTA |
| P4HA1 | Human | AAACGTCTGAATACTGAGTG | CTGGAAGATTACCCTTTGAG |
| GPX1 | Human | CAGTCGGTGTATGCCTTCTCG | GAGGGACGCCACATTCTCG |
| DSEL | Human | AAAGTCTCGTGCAAGCCATTT | GTTGGGTTGGACAGCATAACT |
| ENO1 | Human | GGGAAAGATGCCACCAATGT | GCCGATGACCACCTTATCAG |
| FBXW7 | Human | AGAAATGTCAAAAGACCAGCCA | TATGACCGTTTGCTTGTTGGG |
| ACTB | Human | GACATTAAGGAGAAGCTGTG | CATGATGGAGTTGAAGGTAG |

Table S3. Sixteen metabolism-related candidate genes prioritized by integrative analysis of TMT proteomics, GSE193957 and TCGA datasets

| **Gene** | **GSE logFC**  **(U87-R vs U87)** | **GSE adj.P** | **TCGA log_2_FC**  **(Tumor vs Normal)** | **TCGA adj.P** | **TMT Ratio**  **(U251-R vs U251)** |  | **TMT P** | **Validation** |
| --- | --- | --- | --- | --- | --- | --- | --- | --- |
| ACOT1 | 0.971348722 | 4.19e-06 | 0.0121960677849144 | 0.984470813063229 | 1.466 |  | 3.93090000000429e-05 | Fig.1D |
| ALDH3B1 | 1.2923705 | 2.71e-05 | 1.50727455862757 | 0.000431353357678518 | 0.666 |  | 0.001675 | Fig.1D |
| B4GALT4 | 0.7370335 | 2.13e-05 | 1.1468543944605 | 2.85625059138498e-08 | 0.711 |  | 0.000137371 | Fig.1D |
| DSEL | 2.229331778 | 1.02e-05 | 0.767184552562041 | 0.0507384099199691 | 1.766 |  | 0.00167639999999991 | Fig.1D |
| GPX1 | 0.0438025 | 0.556824524 | 1.77821945443267 | 0.0000252628828618332 | 0.648 |  | 0.0035578 | Fig.1D |
| HEXB | 0.179165611 | 0.111849283 | 1.59344541516654 | 5.38581852528964E-07 | 0.705 |  | 0.00078335 | Fig.1D |
| LDHA | 0.621269389 | 0.000246167 | 1.02456666002386 | 0.00741217074653959 | 1.325 |  | 0.00268449999999998 | Fig.1D |
| NMNAT1 | 0.633849444 | 9.69e-05 | 0.291978140574198 | 0.252756953359149 | 1.624 |  | 0.00270320000000002 | Fig.1D |
| NUDT5 | 0.486808444 | 0.000202925 | 0.569728137497433 | 0.00713983296345067 | 1.36 |  | 0.0028842 | Fig.1D |
| P4HA1 | 1.364296944 | 5.45e-06 | 1.48400573222098 | 4.36275591320336e-05 | 1.407 |  | 0.000696849999999971 | Fig.1D |
| PLCE1 | 2.148431556 | 8.96e-07 | 0.445950104518381 | 0.16332102725678 | 1.386 |  | 0.0103222000000001 | Fig.1D |
| **PLOD1** | **0.656238111** | **0.000991498** | **1.71030346480028** | **3.72298031002445e-07** | **1.35** |  | **0.000762660000000026** | **Selected for subsequent investigation** |
| PPAT | 0.141701444 | 0.050559142 | 0.361631251126316 | 0.185473913019695 | 0.645 |  | 3.83e-06 | Fig.1D |
| PRDX6 | 0.063599944 | 0.341749161 | 0.783961911653675 | 0.0110816329474892 | 1.318 |  | 0.00046214 | Fig.1D |
| SUV39H1 | 0.182839944 | 0.031297135 | 0.273738587884525 | 0.267318499429276 | 0.666 |  | 0.00027936 | Fig.1D |
| UPP1 | 0.064633667 | 0.453376525 | 1.40123837631081 | 0.00453580665796987 | 0.59 |  | 1.73e-05 | Fig.1D |

Note: TMT DEPs were defined using FC >1.3 or FC <1/1.3 with p <0.05. Candidate genes were obtained by integrating metabolism-related TMT DEPs with genes identified from GSE193957 and TCGA datasets. The GSE193957 and TCGA datasets were employed as independent transcriptomic resources to cross-reference expression profiles and prioritize biologically relevant candidates.
