# Supplementary material for: PLOD1 Catalytic Activity Stabilizes ENO1 by Limiting FBXW7‐dependent Degradation to Promote Glycolysis and TMZ Resistance in Glioblastoma
Source: Adv Sci (Weinh). 2026 Sep 27:e78070. Online ahead of print. doi: 10.1002/advs.78070 (PMC13616334; doi:10.1002/advs.78070)
Supplement: Supplementary file 5 [file ADVS-9999-e78070-s005.docx]

Cell line

**1. U87 MG**

• Acquisition date/year: ___2023.5.18_______________

• Passage range used in this study: P___3__ to P___20__

• Highest passage used: P__20___

• Latest passage used for reported experiments: P__10___

• Mycoplasma test closest to latest experimental passage — date: ___2026.4.20_______ ; passage: P__10___ ; result: ___Negative_______

U87 MG (ATCC HTB-14; RRID: CVCL_0022) is recognized in Cellosaurus as being distinct from the original U-87 MG cell line established at Uppsala University. STR profiling confirmed that the U87 MG cells used in this study completely matched the current ATCC U-87 MG reference profile (EV = 1.0). Therefore, this cell line is explicitly reported herein as ATCC-derived U87 MG and was authenticated against the corresponding ATCC reference profile.

•STR:U87 MG cells (ATCC HTB-14; RRID: CVCL_0022) were authenticated by short tandem repeat (STR) profiling. The STR profile showed a complete match to the U-87MG ATCC reference profile in the EXPASY database, with an evaluation value (EV) of 1.0. No multiple alleles were detected. The original STR authentication report is provided below. The original report was issued in Chinese by the testing laboratory.

•Mycoplasma test:

First:date:2025.3.15

Latest test date:2026.4.20

**2. U251 MG**

• Acquisition date/year: ________2023.5.18___________

• Passage range used in this study: P___3__ to P___18__

• Highest passage used: P____18_

• Latest passage used for reported experiments: P__9___

• Mycoplasma test closest to latest experimental passage — date: ___2026.4.20_____ ; passage: P___9__ ; result: ___Negative _____

•STR:U251 MG cells (Sigma-Aldrich, #09063001; RRID: CVCL_0021) were authenticated by short tandem repeat (STR) profiling. The STR profile showed a basic match to the U-251 MG (KO) reference profile in the DSMZ database, with an evaluation value (EV) of 0.94. No multiple alleles were detected. The original STR authentication report is provided below. The original report was issued in Chinese by the testing laboratory.

•Mycoplasma test:

First:date:2025.3.15

Latest test date:2026.4.20

**3. U87-R / U87MG-TMZ**

• Date TMZ-resistance establishment was completed: _________20240321___________

• Confirm parental origin: U87 MG

• Passage range after establishment used in this study: P_5____ to P_51___

• Highest passage used: P__51___

• Mycoplasma assay method: _PCR / qPCR ___________________

• Mycoplasma test date: _______2026.4.20________

• Passage tested: P__51___

• Result: Negative

•Mycoplasma test:

First:date:2025.3.15

Latest test date:2026.4.20

**4. U251-R / U251MG-TMZ**

• Date TMZ-resistance establishment was completed: ________20240324____________

• Confirm parental origin: U251 MG

• Passage range after establishment used in this study: P____4_ to P_63_

• Highest passage used: P__63___

• Mycoplasma assay method: _____PCR/qPCR_______________

• Mycoplasma test date: ______2026.4.20__________________

• Passage tested: P__63___

• Result: Negative

•Mycoplasma test:

First:date:2025.6.1

Latest test date:2026.4.20

**5. HEK293T**

• Acquisition date/year: ___20220214_________________

• Passage range used in this study: P_2____ to P__17___

• Highest passage used: P__17___

• Latest passage used for reported experiments: P__13___

• Mycoplasma test closest to latest experimental passage — date: _____2026.4.20______ ; passage: P__17___ ; result: ____Negative______

•STR:HEK293T cells (ATCC CRL-3216; RRID: CVCL_0063) were authenticated by short tandem repeat (STR) profiling. The STR profile was consistent with the HEK293T reference profile. The original STR authentication report and electropherogram are provided below. The original authentication report was issued in Chinese by the testing laboratory.

•Mycoplasma test:

First:date:2025.3.15

Latest test date:2026.4.20
